# Introduction of a 7-aza-6-MeO-indoline auxiliary in Lewis-acid/photoredox cooperative catalysis: highly enantioselective aminomethylation of α,β-unsaturated amides†

**DOI:** 10.1039/d0sc01890b

**Published:** 2020-04-27

**Authors:** Santosh K. Pagire, Naoya Kumagai, Masakatsu Shibasaki

**Affiliations:** Institute of Microbial Chemistry (BIKAKEN) 3-14-23, Kamiosaki Shinagawa-ku Tokyo 141-0021 Japan mshibasa@bikaken.or.jp nkumagai@bikaken.or.jp

## Abstract

An efficient cooperative chiral Lewis acid/photoredox catalytic system for engaging highly reactive radicals in highly enantioselective conjugate addition to α,β-unsaturated carbonyls is highly desirable. Direct photoexcitation of unbound substrates typically induces undesired background pathways for racemic products and remains a formidable challenge to be addressed in the area of enantioselective photocatalysis. Herein, we report a cooperative catalytic system comprising a chiral Cu(i) complex and an Ir(iii) photocatalyst fueled by visible-light irradiation that allows for seamless integration of the catalytic formation of α-amino alkyl radicals and subsequent enantioselective addition to α,β-unsaturated amides. A 7-aza-6-MeO-indoline attachment on the amide substrates plays a pivotal role in suppressing the undesired pathways, resulting in excellent enantioselectivity and enabling expedited access to valuable γ-aminobutyramides. The indoline amide was readily diversified with full recovery of the azaindoline attachment, highlighting the synthetic utility of this cooperative catalytic system.

## Abstract 2

An efficient cooperative chiral Lewis acid and photoredox catalytic system towards the highly enantioselective radical conjugate addition of α-amino radicals to α,β-unsaturated amides is developed with the implementation of unique auxiliaries.

## Graphical abstract

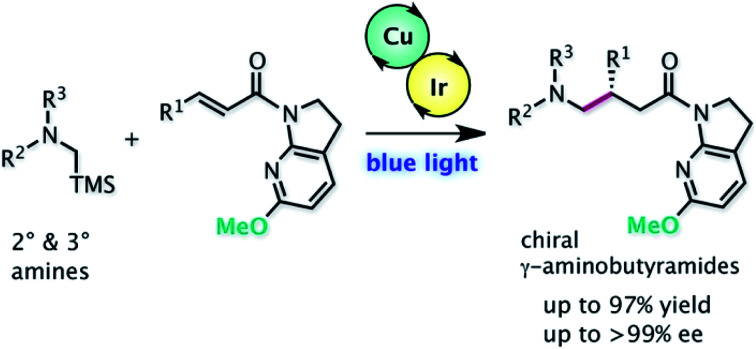

## Introduction

Over the past decade, the field of visible-light photoredox catalysis has rapidly advanced as a sustainable methodology for exerting orthogonal activation modes to manipulate small organic molecules,^1,2^ in which photoinduced single-electron transfer triggers the *in situ* generation of active chemical species from organic substrates, thereby enabling a variety of unique chemical events to facilitate C–C and C–heteroatom bond formation.^3–12^ Recently, reaction promotion by combining photoexcitation and concomitant stereocontrol with the aid of catalytic chiral sources such as chiral Lewis acids or organocatalysts has become a topic of great interest and prompted the development of enantioselective photocatalysis.^13–23^ Meggers *et al.* developed chiral-at-metal Λ-configured or Δ-configured Ir(iii) or Rh(iii) photoactive complexes for a broad range of asymmetric photocatalytic transformations.^24,25^ Although several enantioselective photoredox reactions are now well established, a number of fundamental reactions such as Giese reactions^26,27^ could benefit from improved catalytic systems.^28^ In this particular case, the α,β-unsaturated carbonyl substrates may undergo direct [2 + 2]-photocycloaddition^29–33^ or *E*/*Z*-isomerization,^34,35^ which is often difficult to suppress.^36^ To overcome such shortcomings, elaborated catalytic systems were devised to streamline the desired catalytic scenario. In the late 20^th^ century, Sibi and Porter described the Lewis acid-promoted enantioselective radical addition to the electron-deficient α,β-unsaturated compounds.^37^ Stoichiometric amounts of reagents were required, however, likely due to the undesired background reaction of the unbound substrate. Yet, this reaction set the standard for the future development of numerous enantioselective conjugate radical addition reactions.^38–41^ It was later demonstrated that visible light photocatalysts could be utilized to generate α-amino radicals, thereby obviating the mandatory use of external reagents.^42–50^ Catalytic enantioselective photocatalytic intramolecular conjugate addition of α-amino radicals to the quinolone skeleton was first disclosed by Bach's group using a chiral hydrogen-bonding photosensitizer.^51^ The work of Pandey *et al.*^52–56^ and others^57–62^ revealed that α-silylamines have a pivotal role in the photogeneration of α-amino radical species, which are integrated into a subsequent conjugate addition in an intermolecular fashion. Yoon *et al.* developed a dual catalytic system comprising a chiral Sc(iii) complex and a Ru-based photocatalyst that promotes conjugate addition to form the chiral products with excellent enantiocontrol.^63^ Subsequently, Gong *et al.* and Meggers *et al.* independently provided alternative protocols for conjugate additions using ingeniously designed chiral photocatalysts such as Ni(ii)-DBFOX^64^ and Δ-RhO,^65^ respectively. With a similar platform, few other groups attempted analogous reactions, and the dominant racemic background reaction could be overcome to the great extent.^66,67^

Our recent efforts focusing on asymmetric catalysis led us to identify that 7-azaindoline amides are privileged substrates that drive a number of highly stereocontrolled C–C bond-forming reactions in the context of Cu(i) catalysis.^68,69^ However, the 7-azaindoline auxiliary had not been implemented in the field of photocatalysis and the use of inexpensive copper complexes is rarely explored in asymmetric photocatalysis.^23,70–73^ We reasoned that this auxiliary could also serve as a potential stereo-controlling unit in radical reactions and provide a powerful alternative to several challenging photocatalytic transformations. Herein, we established a photocatalytic additive-free protocol exerted by Cu(i)/Ir(iii) dual catalysis to engage α,β-unsaturated 7-azaindoline amides and α-silylamines in a highly enantioselective radical conjugate addition. The intriguing substituent effect of the 7-azaindoline unit was first revealed by systematic studies and comparisons of the crystal structures of Cu(i)/amide complexes. The broad substrate generality is supported by divergent transformation of the 7-azaindoline moiety of the enantioenriched products and highlights the synthetic utility of the present catalytic protocol.

## Results and discussion

We initiated our investigation with the prototypical 7-azaindoline amide **1a** and α-silylamine **2a** as an electrophile and radical source, respectively, where 1 mol% of [Ir(ppy)_2_(dtb-bpy)]PF_6_ was used as a photocatalyst under blue-light irradiation (*λ*_max_ 448/455 nm). The initial attempt using a Cu(i)/(*S*,*S*)-^*t*^Bu-PyBox (**L1**) complex as a typical chiral Lewis acid in acetonitrile at room temperature resulted in poor conversion to give the desired product **3aa** (Table 1, entry 1). This is likely due to the decomposition of the Cu(i) complex as indicated by the deeply colored reaction mixture, preventing photoexcitation of the Ir(iii) photocatalyst. In contrast, Cu(i) phosphine complexes emerged as a compatible chiral Lewis acid under blue-light irradiation, affording **3aa** in moderate yield (entries 2–6). Among the chiral phosphines tested, a commercially available biaryl-type (*R*)-DM-Segphos (**L2**) afforded the highest enantioselectivity (89% ee), which was sensitive to the reaction temperature (entries 2–4). A protic solvent like ethanol was beneficial for increasing the yield to 73% (*vide infra*, see mechanistic discussion), but a reduction in solubility hampered the smooth progress of the reaction at −20 °C (entries 7 and 8). Aiming to attain homogeneity, we next evaluated a mixed solvent system, leading to the identification of EtOH : DME (1 : 3) as the optimal reaction media to give **3aa** in 83% yield with 89% ee (entries 9–12). Lowering the reaction temperature to −30 °C did not further enhance the enantioselectivity, and the reaction became more sluggish (entry 13). No reaction occurred in the absence of a photocatalyst or light source, confirming that the photocatalytic activation of **2a** is essential to the cooperative catalysis (entries 14 and 15). Conditions lacking a Cu(i) catalyst gave no product **3aa**, indicating that the reaction system does not undergo a non-stereoselective background reaction (entry 16).

**Table 1:** Screening of the reaction conditions^a^

Entry	Ligand	Solvent	Temp (°C)	Yield^b^ (%)	ee^c^ (%)
1	**L1**	MeCN	23	10	ND
2	**L2**	MeCN	23	72	30
3	**L2**	MeCN	5	61	70
4	**L2**	MeCN	−20	62	89
5	**L3**	MeCN	5	49	52
6	**L4**	MeCN	5	43	−44
7	**L2**	EtOH	5	73	74
8	**L2**	EtOH	−20	Trace	ND
9	**L2**	EtOH : MeCN (1 : 1)	−20	68	66
10	**L2**	EtOH : DME (1 : 1)	−20	77	76
11	**L2**	EtOH : DME (3 : 1)	−20	49	85
**12**	**L2**	**EtOH** **:** **DME (1** **:** **3)**	**−20**	**83(80)** d	**89**
13	**L2**	EtOH : DME (1 : 3)	−30	67	89
14^e^	**L2**	EtOH : DME (1 : 3)	−20	Trace	ND
15^f^	**L2**	EtOH : DME (1 : 3)	−20	Trace	ND
16^g^	**L2**	EtOH : DME (1 : 3)	−20	Trace	ND
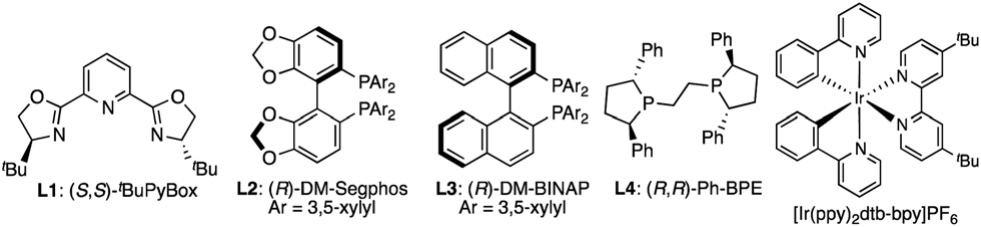					

a **1a**: 0.1 mmol, **2a**: 0.25 mmol, blue light (*λ*_max_ 448/455 nm).

b Determined by ^1^H NMR analysis of the crude reaction mixture with 1,1,2,2-tetrachloroethane as an internal standard.

c Determined by chiral stationary phase HPLC analysis.

d Isolated yield.

e Without a photocatalyst.

f Reaction in the dark.

g Without a Cu(i) catalyst.

The necessity of the 7-azaindoline auxiliary was confirmed in a series of reactions using distinct α,β-unsaturated amides as acceptors of the in situ-generated radical (Table 2). Minute changing in the unsaturation of the heterocycle to 7-azaindole **4** resulted in no reaction. Isomeric 4-azaindoline **5** or indoline derivative **6** exhibited no reactivity, indicating that the coordination capability of the nitrogen atom at the 7-position is essential. Of note, a potentially chelating acyclic *N*-(2-pyridyl)amide **7** failed to promote the reaction, highlighting the exclusive nature of 7-azaindoline to engage the α,β-unsaturated carbonyl units in an asymmetric radical reaction. As expected, a Weinreb amide **8** and dimethyl amide **9** were incompatible substrates. Based on the apparent pivotal role of 7-azaindoline to control stereoselection *via* strong bidentate coordination to the Cu(i) complex, we conducted a systematic study of substituent effects (Table 3). Installation of non-coordinating methyl or phenyl groups at the 6-position, the most biasing position to interfere with the coordination to Cu(i), significantly decreased the enantioselectivity (**3ba**, **3ca**). In sharp contrast, upon introducing a potentially coordinative substituent, *e.g.*, methoxy or chloro substituents, at the same position enhanced the yield and improved enantioselectivity up to 98% ee (**3da**, **3ea**).
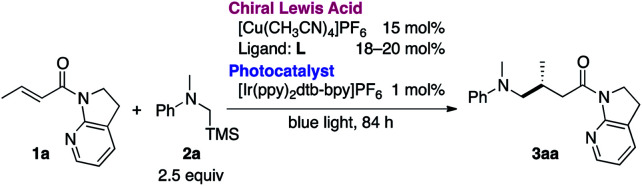


**Table 2:** Necessity of the 7-azaindoline auxiliary^a^

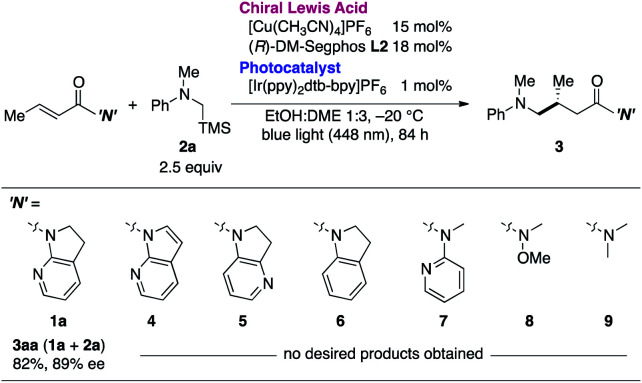

a Amide: 0.1 mmol, **2a**: 0.25 mmol. Yields were determined by ^1^H NMR analysis of the crude reaction mixture with 1,1,2,2-tetrachloroethane as an internal standard.

**Table 3:** Substituent effect of the 7-azaindoline auxiliary^a^

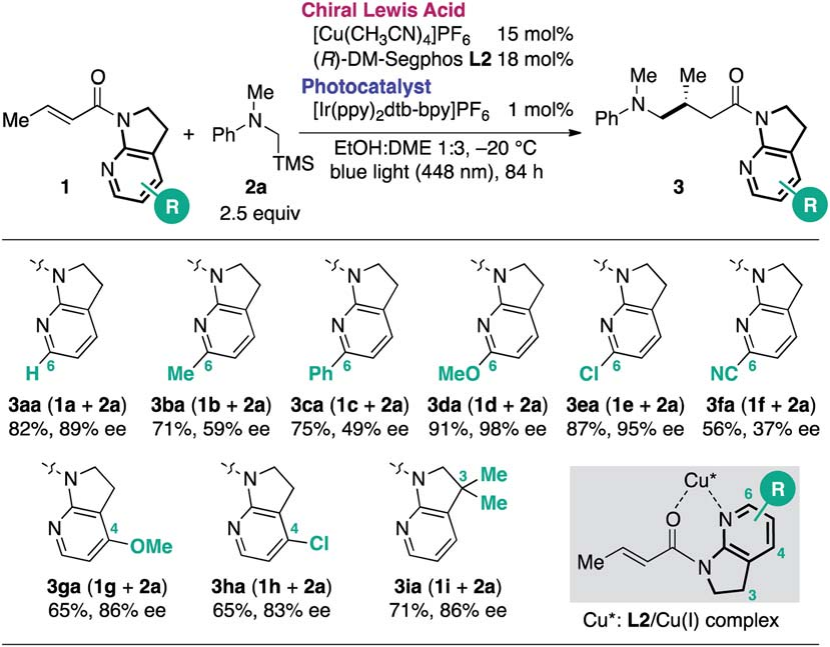

a **1**: 0.1 mmol, **2a**: 0.25 mmol. Yields were determined by ^1^H NMR analysis of the crude reaction mixture with 1,1,2,2-tetrachloroethane as an internal standard.

This finding was further supported by the negative effect of the 6-cyano substituent (**3fa**). This observation can be ascribed to the preferential end-on coordination mode of the cyano functionality, which hindered the coordination ability of the neighboring pyridyl nitrogen. Electronic effects on the pyridyl nitrogen were excluded on the basis of the minimal effect of methoxy or chloro substituents at the 4-position on the enantioselectivity (**3ga**, **3ha**). Similarly, sterics at the 3-position had little effect on the reaction outcome (**3ia**), implying that delicate steric factors with coordination capabilities near the Cu(i) center are crucial toward achieving excellent enantioselectivity.

**Scheme 1:**
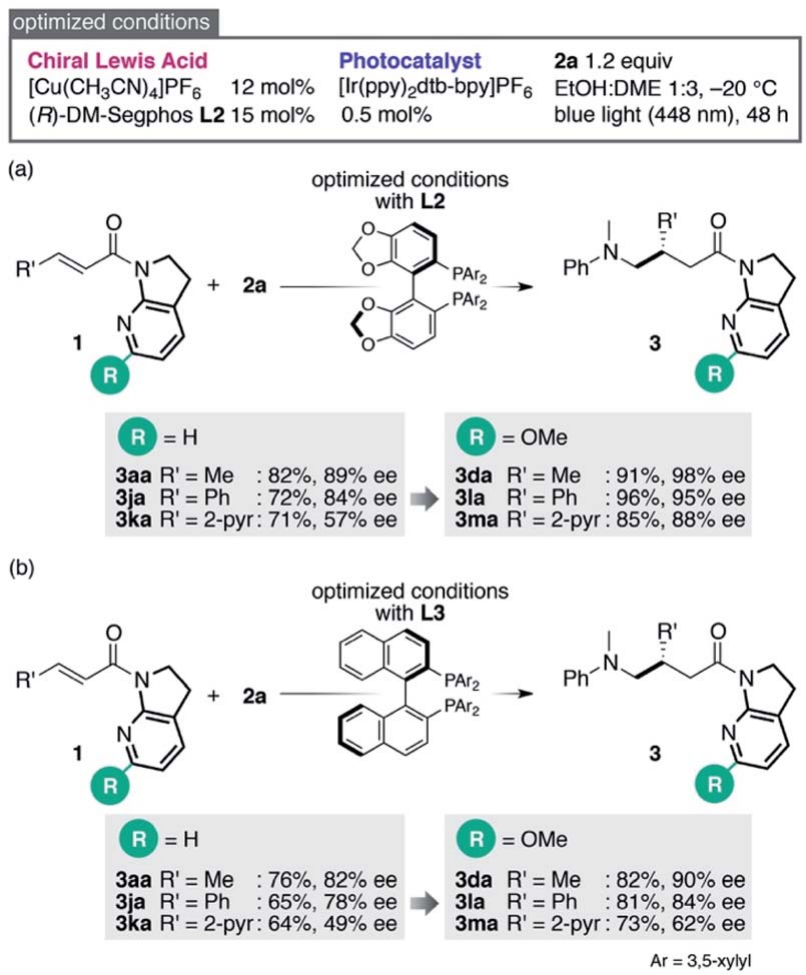
Optimized conditions and enhancement of stereoselection with a 6-MeO substituent.

**Fig. 1:**
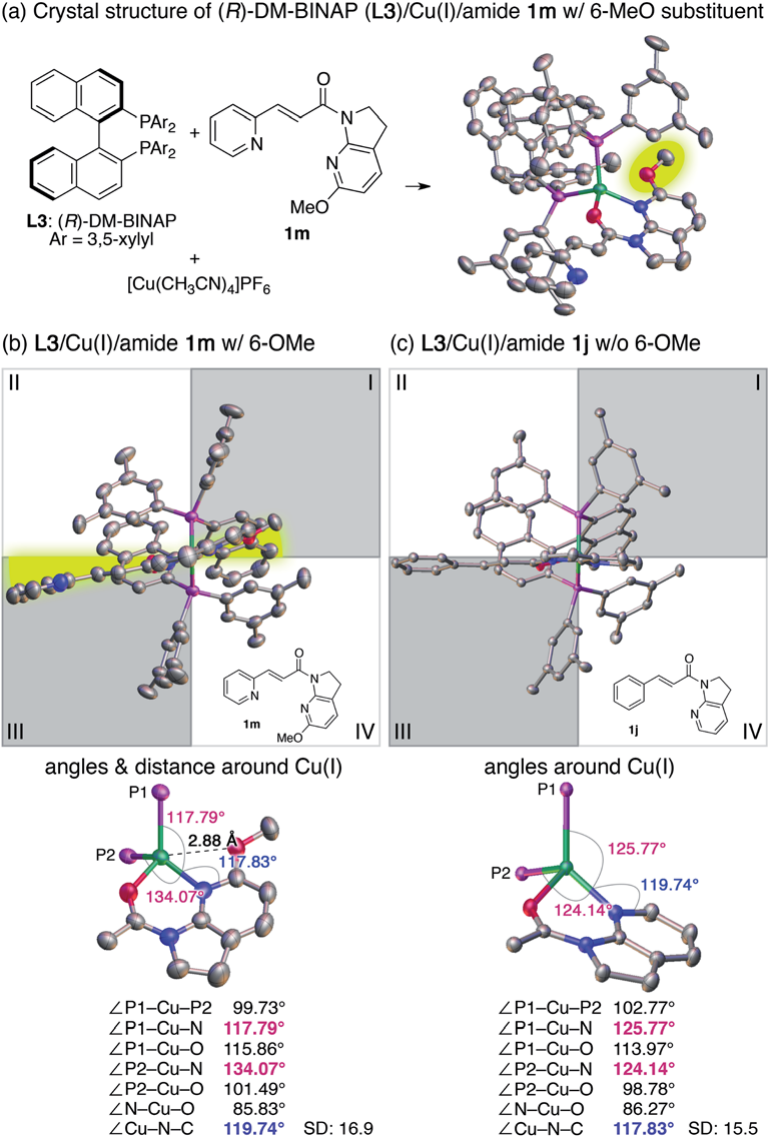
Crystal structures of Cu(i)/amide complexes. (a) Crystal structure of a (*R*)-DM-BINAP (**L3**)/Cu(i)/amide **1m** (with 6-MeO substituent) complex. (b) Front view of the **L3**/Cu(i)/amide **1m** complex (with 6-MeO substituent). (c) Front view of the **L3**/Cu(i)/amide **1j** complex (without 6-MeO substituent). The quadrant representation is applied to structures in (b and c), where the shaded area of the first and third quadrants represents the shielded area by 3,5-xylyl groups on the phosphorus. SD: standard deviation. Thermal ellipsoids are drawn at the 50% probability level. Hydrogens are omitted for clarity. Color code: gray, carbon; blue, nitrogen; red, oxygen; purple, phosphorus; green, copper.

Reinvestigation of the reaction conditions with the proficient 7-aza-6-MeO-indoline attachment revealed that both the catalyst loading and the requisite amount of **2a** could be decreased (Scheme 1). The stereoselectivity enhancement was maintained with α,β-unsaturated amides possessing different β-substituents, indicating that perturbation around the coordination sphere of the Cu(i) centre influenced the stereoselection at the β-position (Scheme 1a). Intriguingly, the beneficial effect of the 6-MeO substituent was also validated in the reaction using a different biaryl ligand, (*R*)-DM-BINAP **L3**, armed with identical aromatic biasing groups (3,5-xylyl) on the phosphorus atom (Scheme 1b). To gain more insight into this notable substituent effect, single crystals of **L3**/Cu(i)/7-azaindoline amide complexes were grown to determine the structural differences induced by the absence or presence of the 6-MeO substituent (Fig. 1). Consistent with the previous observations, the 7-azaindoline amide moiety adopted the *Z*-conformation for bidentate coordination to Cu(i), even in the presence of the 6-MeO substituent, as revealed by X-ray crystallographic analysis of the **L3**/Cu(i)/amide **1m** complex (Fig. 1a).

In-depth studies of the crystal structures of **L3**/Cu(i)/**1m** (with the 6-MeO substituent) and **L3**/Cu(i)/**1j** (without the 6-MeO substituent)^74^ elucidated the structural differences that may account for the positive effect of the 6-MeO substituent. In the absence of the 6-MeO substituent, the front view of the **L3**/Cu(i)/**1j** complex exhibited the ideal tetrahedral coordination around Cu(i), in which the amide **1i** located perpendicular to the P–Cu–P fragment (Fig. 1c). Biasing 3,5-xylyl groups of **L3** exist in the first and third quadrants, and the incoming radical predominantly approaches from the second quadrant to give the product with the observed absolute configuration (see the X-ray crystal structure of **3dd** in Table 4). On the other hand, amide **1m** with the 6-MeO substituent in the **L3**/Cu(i)/**1m** complex slightly rotated counterclockwise (Fig. 1b). Comparative analysis of the bond angles of the two complexes revealed a highly skewed tetrahedral coordination for the **L3**/Cu(i)/**1m** complex with deviated ∠P1–Cu–N (117.79°) and ∠P2–Cu–N (134.07°) angles. While the distance between Cu(i) and the 6-MeO substituent (2.88 Å) exceeded the sum of the van der Waals radii, the narrower ∠Cu–N–C(6) angle (117.83°) compared with that of the **L3**/Cu(i)/**1j** complex (119.74°) suggested that the weak attractive interaction between Cu(i) and the 6-MeO group results from the skewed coordination mode. Indeed, a competitive binding study using an equimolar mixture of the **L3**/Cu(i) complex, **1j**, and **1m** resulted in a nearly 1 : 1 mixture of **L3**/Cu(i)/**1j** and **L3**/Cu(i)/**1m** complexes, suggesting that the 6-MeO group is not merely imparting a steric bias to disfavor complexation (see Section 9 in the ESI for ^1^H-NMR analyses of these complexes†).

**Table 4:** Substrate generality^a^

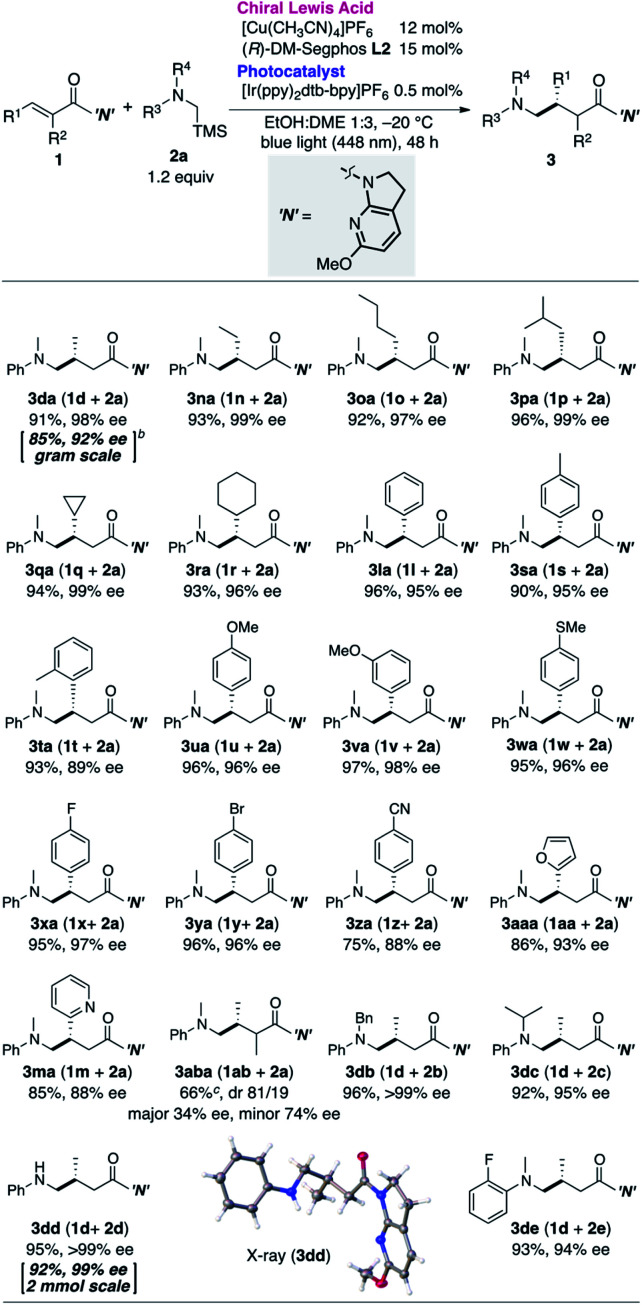

a **1a**: 0.1 mmol, **2a**: 0.12 mmol.

b The reaction was run for 96 h with reduced catalyst loading (Cu: 10 mol%, **L2**: 12 mol%, photocatalyst 0.3 mol%).

c Combined yield of diastereomers. Color code: white, hydrogen; gray, carbon; blue, nitrogen; red, oxygen.

The deviation of the position of amide **1m** from the horizontal line rendered the β-position of the amide more likely to be shielded by the 3,5-xylyl group at the third quadrant, leading to higher enantioselectivity. We believe this mechanism is also operative with 6-Cl-substituted 7-azaindoline amide **1e**, in which the enantioselectivity was similarly increased despite the smaller magnitude of enhancement (Table 3, **3ea**).

Having determined the optimal cooperative catalytic system and incorporating the newly generated the 7-aza-6-MeO-indoline attachment, we next investigated the substrate scope of the catalytic asymmetric aminomethylation (Table 4). The reaction of archetypal β-Me-substituted unsaturated amide could be performed on a gram scale with lower catalyst loading (**3da**, Cu: 10 mol%, Ir: 0.3 mol%), albeit with a marginal loss in enantioselectivity. Linear, branched, and cyclic β-alkyl substituents were largely accommodated to deliver the corresponding products in high yield and with high enantioselectivity (**3na–3ra**). β-Aromatic amides were successfully accommodated as tractable substrates to deliver the corresponding γ-aminomethylated products with high enantioselectivity, irrespective of the presence of electron-donating or -withdrawing substituents (**3la**, **3sa–3za**). Potentially coordinative heteroaromatic units did not interfere with the catalysis (**3ma**, **3aaa**), although the reaction was complicated by the presence of an α-Me substituent on the amide substrate, giving a mixture of diastereomers (81 : 19) in moderate yield with moderate enantioselectivity (**3aba**). Sterically more (*N*-Bn, ^*i*^Pr) or less (*N*-H) demanding α-silylamines **2b–d** as well as **2e** bearing an *o*-F substituent were compatible, affording the desired γ-amino amides (**3db–3de**) in high yield with high enantioselectivity.

**Fig. 2:**
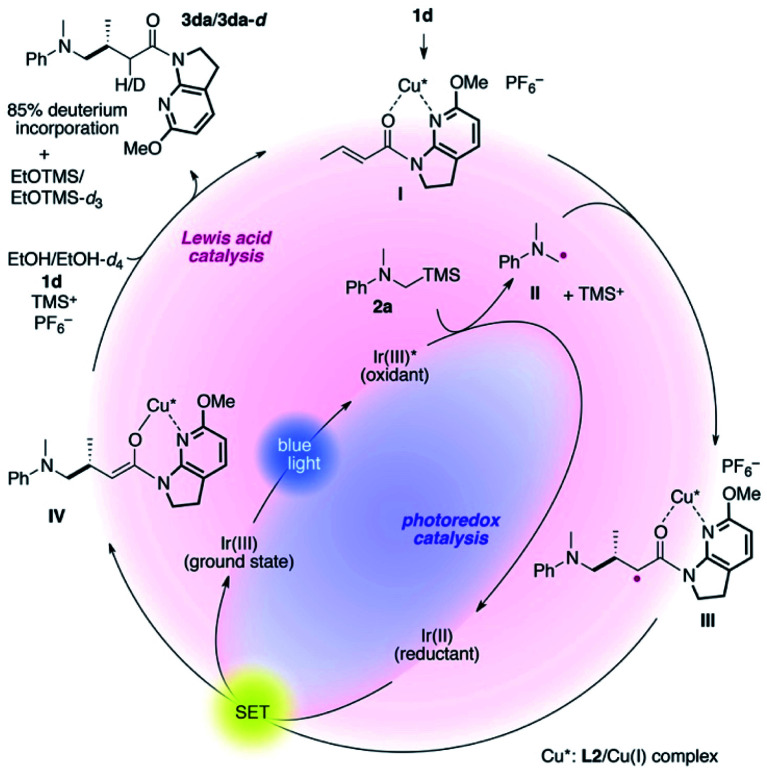
Plausible catalytic cycle.

The proposed catalytic cycle is delineated in Fig. 2. 7-Aza-6-MeO-indoline amide **1d** readily interacts with the **L2**/Cu(i) complex to form complex **I**, mitigating the direct excitation of **1d** for [2 + 2]-photocycloaddition. Independently, α-silylamine **2a** is oxidized by the photoexcited Ir(iii)* complex generated by blue-light irradiation, catalytically producing α-amino radical **II** and TMS cations. At low temperature, the coordinated amide in complex **I** is sufficiently more electrophilic than non-ligated **1d**, and undergoes coupling with radical **II** with excellent stereocontrol, as described in Fig. 1.

**Scheme 2:**
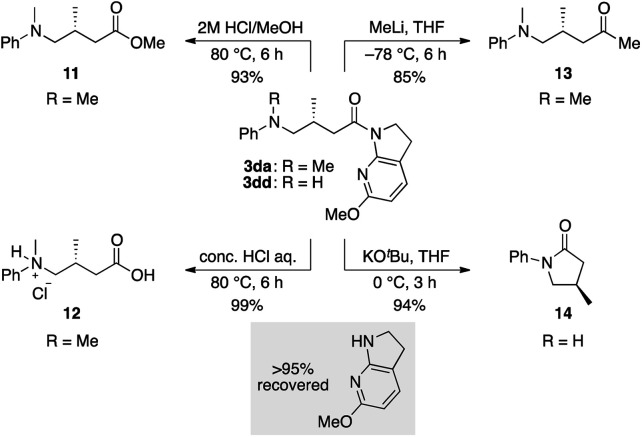
Transformation of the products.

The thus-formed α-amide radical **III** was quenched with the Ir(ii) complex *via* single-electron transfer to give amide enolate **IV** while concomitantly regenerating the ground state Ir(iii) complex to close the photocatalytic cycle. The co-solvent EtOH protonated the amide enolate to liberate γ-amino amide product **3da**, as evidenced by the 85% deuterium incorporation (see Fig. S4 and S5 in the ESI for HRMS and ^1^H NMR analyses, respectively†) when using EtOH-*d*_4_ in place of EtOH. The 6-MeO-7-azaindoline attachment was readily removed after taming the reactivity of the cooperative asymmetric catalysis, allowing for diverse functional group transformations (Scheme 2). Simple acidic hydrolysis of product **3da** gave Me ester **11** or acid **12** depending on the acid concentration and reaction media. This class of compounds provides potentially useful intermediates for γ-aminobutyric acid derivatives, a key structural motif shared in several marketed therapeutics for central nervous system disorders.^75,76^ The 7-azaindoline unit served to stabilize the tetrahedral intermediate formed upon reaction with an organolithium reagent, delivering methyl ketone **13** without over-alkylated product. For secondary amine product **3dd**, simple treatment with KO^*t*^Bu at 0 °C afforded lactam **14**. In all cases, the 6-MeO-7-azaindoline attachment was recovered in over 95% yield.

## Conclusions

In conclusion, we developed a new protocol for a highly enantioselective catalytic conjugate addition of α-amino radicals fueled by visible light irradiation. A chiral Cu(i) catalyst and an Ir-based photocatalyst exert their influence orthogonally to promote the smooth reaction, where the 7-azaindoline unit of the α,β-unsaturated amides plays a key role in both the reaction progress and stereoselection. Substituents at the 6-position of the 7-azaindoline attachment had a significant effect on the reaction, shedding light on the stereocontrol of the privileged Cu(i)/7-azaindoline combination in asymmetric catalysis. The substrate generality and functional group transformation of the products will be highly applicable to practical organic synthesis.

## Conflicts of interest

There are no conflicts to declare.

## Supplementary Material

SC-011-D0SC01890B-s001

SC-011-D0SC01890B-s002
