# Supplementary material for: Introduction of a 7-aza-6-MeO-indoline auxiliary in Lewis-acid/photoredox cooperative catalysis: highly enantioselective aminomethylation of α,β-unsaturated amides
Source: Chem Sci. 2020 Apr 27;11(20):5168–74. doi: 10.1039/d0sc01890b (PMC8159422; doi:10.1039/d0sc01890b)

Electronic Supplementary Material (ESI) for Chemical Science.
This journal is © The Royal Society of Chemistry 2020

Supporting Information

Introduction of a 7-Aza-6-MeO-indoline auxiliary in Lewis-acid/photoredox cooperative catalysis: Highly enantioselective aminomethylation of α,β-unsaturated amides

Santosh K. Pagire, Naoya Kumagai,* and Masakatsu Shibasaki*

Institute of Microbial Chemistry (BIKAKEN), Tokyo, Japan

mshibasa@bikaken.or.jp, nkumagai@bikaken.or.jp

Table of Contents

1. General 2
2. Instrumentation 2
3. Materials 2
4. General procedures for the synthesis of starting materials 3
4.1. Preparation of amides from Crotonoyl chlorides (Procedure A) 3
4.2. Preparation of amides from Cinnamoyl chlorides (Procedure B) 7
4.3. Preparation of amides from Carboxylic acids (Procedure C) 8
4.4. Preparation of amides from 10 using Horner–Wadsworth–Emmons (HWE) reaction (Procedure D) 12
4.5. Synthesis of Amine derivatives (Procedure E) 15
5. Photochemical reactions 16
5.1: Reaction optimization 16
5.2: Standard Procedure 1: Variation of 7-azaindolines 23
5.3: Standard Procedure 2: Variation at the α,β,-positions 30
5.4: Standard Procedure 2: Variation of Amines 45
6. Gram-Scale Reaction 48
7. Transformations of the products 49
7.1. Esterification af amide (Procedure 49
7.2. Hydrolysis of amide 49
7.3. Addition of MeLi to amide 50
7.4. Lactam formation 50
8. Deuterium exchange experiment 51
9. Copper complexation experiments 52
10. Crystal Structures: 59
10.1. Solid state structure of 1w 59
10.2. Solid state structure of 3dd: (determination of absolute configuration) 60
10.3. Solid state structure of C1 61
11. References 62
12. NMR Spectra: 63

1. General

All the photocatalytic reactions were performed in a flame-dried 10 mL glass Schenk test tube with a Teflon-coated magnetic stirring bar unless otherwise noted. The two necked test tubes were equipped with a LED and rubber septum. The reactions were run under Ar atmosphere. Air- and moisture-sensitive liquids were transferred via a gas-tight syringe and a stainless-steel needle. All work-up and purification procedures were carried out with reagent-grade solvents under ambient atmosphere. Flash column chromatography was performed using Biotage Isolera One system.

2. Instrumentation

Unless otherwise stated, all the NMR spectras were recorded in $CDCl_3$ on Bruker AVANCE III HD400 or 500 NMR spectrometer. Chemical shifts for proton are reported in parts per million downfield from tetramethylsilane and are referenced to residual protium in the NMR solvent ($CDCl_3$: δ 7.26 ppm). For ^{13}C NMR, chemical shifts were reported in the scale relative to NMR solvent ($CDCl_3$: δ 77.16 ppm) as an internal reference. NMR data are reported as follows: chemical shifts, multiplicity (s: singlet, d: doublet, t: triplet, q: quartet, m: multiplet, bs: broad signal), coupling constant (Hz), and integration. Infrared (IR) spectra were recorded on a HORIBA FT210 Fourier transform infrared spectrophotometer. Single-crystal X-ray data were collected on a Rigaku R-AXIS RAPID II imaging plate area detector with graphite-monochromated Cu-Kα radiation. Optical rotation was measured using a 1 mL cell with a 1.0 dm path length on a JASCO polarimeter P-1030. High-resolution mass spectra (ESI TOF (+)) were measured on Thermo Fisher Scientific LTQ Orbitrap XL. Preparative HPLC was conducted on a JASCO HPLC system equipped with Daicel chiral-stationary-phase columns (ϕ 20 mm x 250 mm).
The photochemical reactions are performed either by using Micro Photochemical Reactor (purchased from Sigma-Aldrich), blue LED lights AC / DC input 100 V / 240 V AC (**ALDKIT001-1EA**:
Link: <https://www.sigmaaldrich.com/catalog/product/aldrich/aldkit001?lang=ja&region=JP>)
or by LED_{448} **PER-AMP** produced by Techno Sigma (Link: <http://www.techno-sigma.co.jp/ts2006/file_pdf/PER-AMP.pdf>).

3. Materials

Unless otherwise noted, all the required materials and chemicals were purchased from commercial suppliers and were used without further purification. DME was dried and purified by passing through a solvent purification system (Glass Contour). Dry Ethanol was purchased from Wako chemicals ltd. Metals and Ligands were purchased from Tokyo Chemical Industry (TCI) or Strem Chemical, Inc. or Sigma-Aldrich and used as received (opened and handled in the glove box). All the 7-azaindolines are either purchased from the commercial sources (TCI / Wako / Sigma-Aldrich-Merck) or readily prepared from corresponding indoles by already known procedures.[1]
Column chromatography was performed with silica gel Merck 60 (230-400 mesh ASTM), silica gel 60 N (spherical, neutral, 40-50 μm) from Kanto Chemical Co., Inc.
Preparative Thin Layer Chromatography (PTLC) plates (Silica gel 60, F_{254}, 0.5 mm, 20 x 20 cm, 1.05744.0001) were purchased from Merck, Germany.

4. General procedures for the synthesis of starting materials

4.1. Preparation of amides from Crotonoyl chlorides (Procedure A)

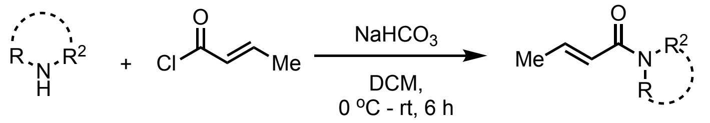

To a solution of the corresponding 2° amines (3 mmol, 1.0 equiv) and $NaHCO_3$ (9 mmol, 3 equiv) in dry DCM (30 mL) was added Crotonyl chloride (193 µL, 6 mmol, 2.0 equiv) at 0 °C and the mixture was stirred at RT for 6 h. The reaction mixture was diluted with water (50 mL) and the product was extracted into DCM (3 x 20 mL). Combined organic layers were dried over Na_2SO_4 and concetrated under reduced pressure. The crude residue was purified by automated flash column chromatography (Biotage Isolera One) with pre-packed silica gel column using Ethyl acetate/Hexane (2/8) eluents. All the products were subsequently recrystallized from DCM/Hexane.

(*E*)-1-(2,3-dihydro-1*H*-pyrrolo[2,3-*b*]pyridin-1-yl)but-2-en-1-one (1a):

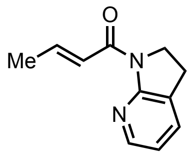

The reaction performed according to the general procedure A afforded 395 mg (70%).

Colorless solid (m.p. 65–67 °C).

^{1}H NMR (400 MHz, 300 K, CDCl$_3$): δ 8.12 (d, *J* = 5.2, 1.3 Hz, 1H), 7.79 (dq, *J* = 15.3, 1.7 Hz, 1H), 7.44 (dq, *J* = 7.4, 1.4 Hz, 1H), 7.17 – 7.04 (m, 1H), 6.85 (dd, *J* = 7.3, 5.1 Hz, 1H), 4.19 – 4.09 (m, 2H), 3.08 – 3.00 (m, 2H), 1.97 (dd, *J* = 7.0, 1.7 Hz, 3H).

^{13}C NMR (101 MHz, 300 K, CDCl$_3$): δ 165.6, 156.4, 146.4, 142.9, 133.7, 126.6, 124.8, 118.1, 46.1, 24.5, 18.6.

The obtained data is in accordance with the literature data.[2]

(*E*)-1-(6-methyl-2,3-dihydro-1*H*-pyrrolo[2,3-*b*]pyridin-1-yl)but-2-en-1-one (1b):

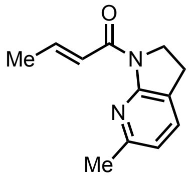

The reaction performed according to the general procedure A afforded 406 mg (67%).

Colorless solid (m.p. 67–69 °C).

^{1}H NMR (400 MHz, 300 K, CDCl$_3$): δ 7.90 (dq, *J* = 15.3, 1.7 Hz, 1H), 7.32 (dd, *J* = 7.6, 1.3 Hz, 1H), 7.17 – 7.04 (m, 1H), 6.71 (d, *J* = 7.5 Hz, 1H), 4.17 – 4.09 (m, 2H), 3.03 – 2.94 (m, 2H), 2.47 (s, 3H), 1.97 (dd, *J* = 6.9, 1.7 Hz, 3H).

^{13}C NMR (101 MHz, 300 K, CDCl$_3$): δ 165.5, 155.8, 155.6, 142.3, 133.7, 125.0, 123.0, 117.2, 46.2, 24.3, 24.1, 18.5.

IR (thin film): $\tilde{\nu}$ 2913, 1660, 1624, 1592, 1447, 1415, 1385, 1341, 1290, 1255, 1221, 1085, 974, 899, 771, 678, 640 cm^{-1}.

HRMS (ESI): *m*/*z* calculated for $C_{12}H_{14}N_2ONa$ [M+Na]$^+$: 225.0998, found: 225.1003.

(*E*)-1-(6-phenyl-2,3-dihydro-1*H*-pyrrolo[2,3-*b*]pyridin-1-yl)but-2-en-1-one (1c):

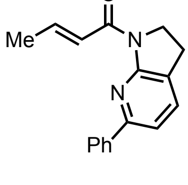

The reaction performed according to the general procedure A afforded 657 mg (83%).

Colorless solid (m.p. 161–163 °C).

^{1}H NMR (400 MHz, 300 K, CDCl$_3$): δ 8.06 (dd, *J* = 15.3, 1.7 Hz, 1H), 8.04 – 7.97 (m, 2H), 7.55 – 7.43 (m, 3H), 7.45 – 7.36 (m, 1H), 7.36 (d, *J* = 7.7 Hz, 1H), 7.19 – 7.06 (m, 1H), 4.26 – 4.14 (m, 2H), 3.12 – 2.99 (m, 2H), 2.02 (dd, *J* = 6.9, 1.7 Hz, 3H).

^{13}C NMR (101 MHz, 300 K, CDCl$_3$): δ 165.7, 156.2, 154.5, 142.0, 139.1, 134.2, 129.0, 128.9, 126.7, 125.5, 125.0, 114.5, 46.2, 24.2, 18.7.

IR (thin film): $\tilde{\nu}$ 2965, 1661, 1628, 1590, 1575, 1499, 1483, 1456, 1446, 1433, 1415, 1376, 1342, 1295, 1242, 1222, 1186, 1112, 1030, 976, 916, 870, 851, 838, 774, 743, 695, 671, 638 cm^{-1}.

HRMS (ESI): *m*/*z* calculated for $C_{17}H_{16}N_2ONa$ [M+Na]$^+$: 287.1155, found: 287.1159.

(*E*)-1-(6-methoxy-2,3-dihydro-1*H*-pyrrolo[2,3-*b*]pyridin-1-yl)but-2-en-1-one (1d):

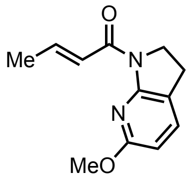

The reaction performed according to the general procedure A afforded 405 mg (62%).
Colorless solid (m.p. 104–106 °C).

^{1}H NMR (400 MHz, 300 K, CDCl$_3$): δ 7.74 (dd, *J* = 15.3, 1.7 Hz, 1H), 7.29 (dd, *J* = 8.1, 1.0 Hz, 1H), 7.04 – 6.92 (m, 1H), 6.26 (d, *J* = 8.1 Hz, 1H), 4.11 – 4.01 (m, 2H), 3.85 (s, 3H), 2.94 – 2.84 (m, 2H), 1.88 (dd, *J* = 6.9, 1.7 Hz, 3H).

^{13}C NMR (101 MHz, 300 K, CDCl$_3$): δ 164.9, 162.9, 153.6, 141.3, 136.0, 125.0, 116.9, 103.4, 53.5, 46.5, 23.5, 18.5.

IR (thin film): $\tilde{\nu}$ 2954, 1659, 1609, 1590, 1465, 1440, 1419, 1386, 1343, 1319, 12191, 1254, 1224, 1192, 1160, 1100, 1024, 962, 902, 822, 777, 668 cm^{-1}.

HRMS (ESI): *m*/*z* calculated for $C_{12}H_{14}N_2O_2Na$ [M+Na]$^+$: 241.0947, found: 241.0951.

(*E*)-1-(6-chloro-2,3-dihydro-1*H*-pyrrolo[2,3-*b*]pyridin-1-yl)but-2-en-1-one (1e):

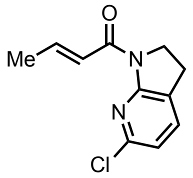

The reaction performed according to the general procedure A afforded 425 mg (64%).
Colorless solid (m.p. 108–110 °C).

^{1}H NMR (400 MHz, 300 K, CDCl$_3$): δ 7.61 (dd, *J* = 15.2, 1.7 Hz, 1H), 7.45 – 7.24 (m, 1H), 7.22 – 6.92 (m, 1H), 6.80 (d, *J* = 7.7 Hz, 1H), 4.19 – 4.00 (m, 2H), 3.11 – 2.84 (m, 2H), 1.92 (dd, *J* = 6.9, 1.7 Hz, 3H).

^{13}C NMR (101 MHz, 300 K, CDCl$_3$): δ 165.1, 155.7, 147.8, 143.3, 135.4, 124.7, 124.1, 117.4, 46.4, 23.6, 18.4.

IR (thin film): $\tilde{\nu}$ 2914, 1661, 1624, 1600, 1575, 1475, 1422, 1379, 1339, 1290, 1250, 1196, 1113, 1071, 970, 918, 875, 809, 772, 716, 677, 638 cm^{-1}.

HRMS (ESI): *m*/*z* calculated for $C_{11}H_{11}N_2OClNa$ [M+Na]$^+$: 245.0452, found: 245.0457.

(*E*)-1-(but-2-enoyl)-2,3-dihydro-1*H*-pyrrolo[2,3-*b*]pyridine-6-carbonitrile (1f):

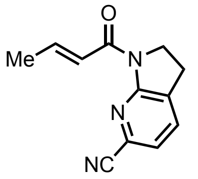

The reaction performed according to the general procedure A afforded 492 mg (77%).
Colorless solid (m.p. 156–158 °C).

^{1}H NMR (400 MHz, 300 K, CDCl$_3$): δ 7.66 (dq, *J* = 15.2, 1.7 Hz, 1H), 7.53 (dt, *J* = 7.4, 1.4 Hz, 1H), 7.28 (d, *J* = 7.5 Hz, 1H), 7.17 (dq, *J* = 15.2, 6.9 Hz, 1H), 4.19 (dd, *J* = 9.2, 8.0 Hz, 2H), 3.13 (ddd, *J* = 9.5, 7.9, 1.4 Hz, 2H), 2.00 (dd, *J* = 6.9, 1.7 Hz, 3H).

^{13}C NMR (101 MHz, 300 K, CDCl$_3$): δ 165.6, 157.0, 144.7, 133.8, 132.0, 129.7, 124.1, 123.4, 117.6, 46.1, 24.5, 18.6.

IR (thin film): $\tilde{\nu}$ 3073, 2969, 2942, 2231, 1629, 1601, 1576, 1478, 1438, 1381, 1341, 1288, 1268, 1250, 1233, 1216, 1080, 1032, 975, 921, 893, 849, 832, 772, 749, 679, 655, 501 cm^{-1}.

HRMS (ESI): *m*/*z* calculated for $C_{12}H_{12}N_3O$ [M+H]$^+$: 214.0975, found: 214.0975.

(*E*)-1-(4-methoxy-2,3-dihydro-1*H*-pyrrolo[2,3-*b*]pyridin-1-yl)but-2-en-1-one (1g):

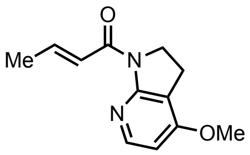

The reaction performed according to the general procedure A afforded 379 mg (58%).
Colorless solid (m.p. 131–133 °C).

^{1}H NMR (400 MHz, 300 K, CDCl$_3$): δ 8.06 (d, *J* = 5.9 Hz, 1H), 7.80 (dd, *J* = 15.3, 1.8 Hz, 1H), 7.18 – 7.02 (m, 1H), 6.48 (d, *J* = 6.0, 0.8 Hz, 1H), 4.18 – 4.07 (m, 2H), 3.86 (s, 3H), 3.00 – 2.85 (m, 2H), 1.96 (dd, *J* = 6.9, 0.9 Hz, 3H).

^{13}C NMR (101 MHz, 300 K, CDCl$_3$): δ 165.4, 162.8, 157.8, 148.7, 142.4, 124.7, 112.5, 102.4, 55.6, 46.36, 21.4, 18.5.

IR (thin film): $\tilde{\nu}$ 2927, 2362, 1656, 1603, 1442, 1414, 1383, 1353, 1289, 1244, 1111, 1024, 981, 825, 678, 613 cm^{-1}.

HRMS (ESI): *m*/*z* calculated for $C_{12}H_{15}N_2O_2$ [M+H]$^+$: 219.1128, found: 219.1131.

(*E*)-1-(4-chloro-2,3-dihydro-1*H*-pyrrolo[2,3-*b*]pyridin-1-yl)but-2-en-1-one (1h):

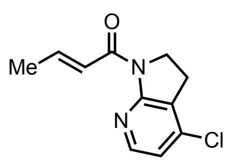

The reaction performed according to the general procedure A afforded 453 mg (68%).
Colorless solid (m.p. 124–126 °C).
^{1}H NMR (400 MHz, 300 K, $CDCl_3$): δ 7.98 (d, *J* = 5.6 Hz, 1H), 7.72 – 7.65 (m, 1H), 7.14 – 7.03 (m, 1H), 6.82 (d, *J* = 5.6 Hz, 1H), 4.17 – 4.05 (m, 2H), 3.11 – 2.95 (m, 2H), 1.93 (dd, *J* = 7.0, 1.7 Hz, 3H).
^{13}C NMR (101 MHz, 300 K, $CDCl_3$): δ 165.3, 157.1, 147.2, 143.3, 140.8, 125.1, 124.3, 118.2, 45.6, 23.5, 18.4.
IR (thin film): $\tilde{\nu}$ 2907, 1661, 1624, 1597, 1484, 1434, 1409, 1349, 1240, 1081, 974, 804, 640 cm^{-1}.
HRMS (ESI): *m*/*z* calculated for $C_{11}H_{11}N_2OClNa$ $[M+Na]^+$: 245.0452, found: 245.0457.

(*E*)-1-(3,3-dimethyl-2,3-dihydro-1*H*-pyrrolo[2,3-*b*]pyridin-1-yl)but-2-en-1-one (1i):

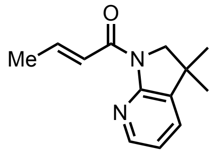

The reaction performed according to the general procedure A afforded 382 mg (59%).
Colorless solid (m.p. 64–66 °C).
^{1}H NMR (400 MHz, 300 K, $CDCl_3$): δ 8.15 (dd, *J* = 5.1, 1.6 Hz, 1H), 7.79 (dd, *J* = 15.3, 1.7 Hz, 1H), 7.41 (dd, *J* = 7.4, 1.7 Hz, 1H), 7.20 – 7.06 (m, 1H), 6.90 (dd, *J* = 7.4, 5.1 Hz, 1H), 3.89 (s, 2H), 1.98 (dd, *J* = 6.9, 1.7 Hz, 3H), 1.32 (s, 6H).
^{13}C NMR (101 MHz, 300 K, $CDCl_3$): δ 165.5, 154.9, 146.5, 142.9, 135.6, 131.1, 124.8, 118.4, 60.5, 36.7, 28.5, 18.5.
IR (thin film): $\tilde{\nu}$ 2960, 1663, 1624, 1598, 1418, 1377, 1349, 1294, 1221, 1138, 1065, 972, 916, 777, 694, 641 cm^{-1}.
HRMS (ESI): *m*/*z* calculated for $C_{13}H_{17}N_2O$ $[M+H]^+$: 217.1335, found: 217.1338.

(*E*)-1-(1*H*-pyrrolo[2,3-*b*]pyridin-1-yl)but-2-en-1-one (4):

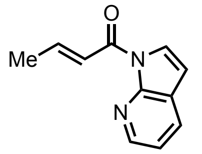

The reaction performed according to the general procedure A afforded 424 mg (76%).
Colorless solid (m.p. 54–56 °C).
^{1}H NMR (400 MHz, 300 K, $CDCl_3$): δ 8.35 (dd, *J* = 4.8, 1.7 Hz, 1H), 8.25 (dq, *J* = 15.3, 1.7 Hz, 1H), 8.03 (d, *J* = 4.1 Hz, 1H), 7.84 (dt, *J* = 7.8, 1.2 Hz, 1H), 7.39 (dq, *J* = 15.3, 7.0 Hz, 1H), 7.15 (ddd, *J* = 7.8, 4.8, 0.7 Hz, 1H), 6.57 (dd, *J* = 4.1, 0.7 Hz, 1H), 2.07 (dd, *J* = 7.0, 1.7 Hz, 3H).
^{13}C NMR (101 MHz, 300 K, $CDCl_3$): δ 163.9, 147.9, 147.4, 143.6, 129.3, 126.1, 124.6, 124.1, 118.6, 105.9, 18.9.
IR (thin film): $\tilde{\nu}$ 3073, 2971, 1692, 1643, 1595, 1579, 1530, 1471, 1443, 1406, 1373, 1331, 1294, 1282, 1262, 1210, 1129, 1114, 1100, 1060, 1045, 1024, 971, 925, 889, 832, 803, 777, 754, 735, 680, 661, 597, 501 cm^{-1}.
HRMS (ESI): *m*/*z* calculated for $C_{11}H_{11}N_2O$ $[M+H]^+$: 187.0866, found: 187.0867.

(*E*)-1-(2,3-dihydro-1*H*-pyrrolo[3,2-*b*]pyridin-1-yl)but-2-en-1-one (5):

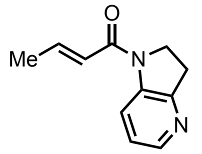

The reaction performed according to the general procedure A afforded 445 mg (79%).
Colorless solid (m.p. 65–67 °C).
^{1}H NMR (400 MHz, DMSO-*d*$_6$): δ 8.12 (dd, *J* = 5.2, 1.5 Hz, 1H), 7.79 (dd, *J* = 15.3, 1.7 Hz, 1H), 7.63 (dd, *J* = 7.4, 1.5 Hz, 1H), 6.97 (dd, *J* = 7.4, 5.1 Hz, 1H), 6.91 (dt, *J* = 15.3, 6.9 Hz, 1H), 4.00 (dd, *J* = 9.1, 7.8 Hz, 2H), 3.08 – 2.99 (m, 2H), 1.91 (dd, *J* = 6.9, 1.7 Hz, 3H).
^{13}C NMR (101 MHz, DMSO-*d*$_6$): δ 163.8, 155.6, 145.7, 141.5, 134.0, 126.7, 124.6, 118.1, 45.7, 23.6, 18.0.
IR (thin film): $\tilde{\nu}$ 2909, 1660, 1615, 1600, 1587, 1475, 1443, 1419, 1383, 1352, 1323, 1289, 1240, 1165, 972, 916, 773, 692 cm^{-1}.
HRMS (ESI): *m*/*z* calculated for $C_{11}H_{13}N_2O$ $[M+H]^+$: 189.1022, found: 189.1025.

(*E*)-1-(indolin-1-yl)but-2-en-1-one (6):

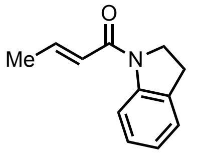

The reaction performed according to the general procedure A afforded 449 mg (80%).

Colorless solid.

^{1}H NMR (400 MHz, 300 K, $CDCl_3$): δ 8.27 (bs, 1H), 7.23 – 7.12 (m, 2H), 7.14 – 6.95 (m, 2H), 6.25 (d, *J* = 15.1 Hz, 1H), 4.13 (dd, *J* = 9.0, 8.0 Hz, 2H), 3.17 (s, 2H), 1.94 (dd, *J* = 6.9, 1.7 Hz, 3H).

^{13}C NMR (101 MHz, 300 K, $CDCl_3$): ^{13}C NMR (101 MHz, $CDCl_3$) δ 164.4, 143.2, 142.8, 131.6, 127.6, 124.6, 123.7, 117.5, 48.1, 28.1, 18.4.

The obtained data is in accordance with the literature data.[2]

(*E*)-*N*-methyl-*N*-(pyridin-2-yl)but-2-enamide (7):

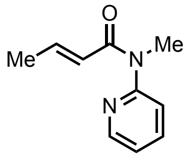

The reaction performed according to the general procedure A afforded 386 mg (73%).

Colorless oil.

^{1}H NMR (400 MHz, 300 K, $CDCl_3$): δ 8.51 (ddd, *J* = 4.7, 2.0, 1.0 Hz, 1H), 7.73 (ddd, *J* = 8.0, 7.4, 2.0 Hz, 1H), 7.19 (ddd, *J* = 7.4, 5.3, 1.0 Hz, 2H), 6.97 (dt, *J* = 15.0, 6.9 Hz, 1H), 5.93 (dt, *J* = 15.0, 1.7 Hz, 1H), 3.44 (s, 3H), 1.80 (dd, *J* = 6.9, 1.7 Hz, 3H).

^{13}C NMR (101 MHz, 300 K, $CDCl_3$): δ 166.6, 156.2, 149.2, 142.0, 138.1, 123.4, 121.6, 121.2, 35.4, 18.2.

The obtained data is in accordance with the literature data.[2]

(*E*)-*N*-methoxy-*N*-methylbut-2-enamide (8):

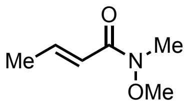

The reaction performed according to the general procedure A afforded 290 mg (75%).

Colorless oil.

^{1}H NMR (400 MHz, 300 K, $CDCl_3$): δ 6.91 (ddt, *J* = 15.5, 6.8, 3.1 Hz, 1H), 6.36 (dq, *J* = 15.4, 2.3, 1.8 Hz, 1H), 3.69 – 3.61 (m, 3H), 3.22 – 3.14 (m, 3H), 1.89 – 1.82 (m, 3H).

^{13}C NMR (101 MHz, 300 K, $CDCl_3$): δ 167.0, 142.9, 120.2, 61.7, 32.3, 18.2.

IR (thin film): $\tilde{\nu}$ 2968, 2938, 2915, 1669, 1637, 1448, 1412, 1381, 1297, 1179, 1152, 1124, 1083, 1045, 1009, 966, 913, 826, 815, 675, 620, 516, 441 cm^{-1}.

HRMS (ESI): *m*/*z* calculated for $C_6H_{12}NO_2$ $[M+H]^+$: 130.0863, found: 130.0863.

(*E*)-*N*,*N*-dimethylbut-2-enamide (9):

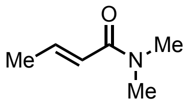

The reaction performed according to the general procedure A afforded 227 mg (67%).

Colorless solid.

^{1}H NMR (400 MHz, 300 K, $CDCl_3$): δ 6.79 (dq, *J* = 15.0, 6.8 Hz, 1H), 6.21 (dq, *J* = 15.0, 1.7 Hz, 1H), 3.00 (s, 3H), 2.92 (s, 3H), 1.81 (dd, *J* = 6.9, 1.7 Hz, 3H).

^{13}C NMR (101 MHz, 300 K, $CDCl_3$): δ 166.8, 141.0, 121.8, 37.3, 35.6, 18.1.

The obtained data is in accordance with the literature data.[2]

4.2. Preparation of amides from Cinnamoyl chlorides (Procedure B)

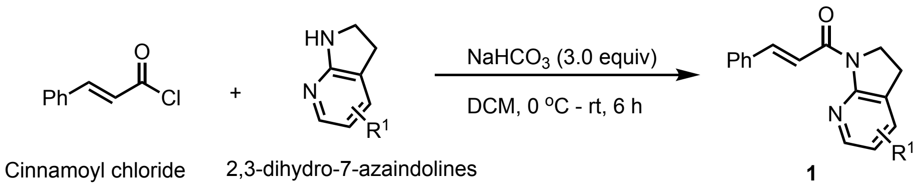

To a solution of the corresponding 2,3-dihydro-7-azaindole derivative (3 mmol, 1.0 equiv) and $NaHCO_3$ (9 mmol, 3 equiv in dry DCM (30 mL) was added Cinnamoyl chloride (1.0 g, 6 mmol, 2.0 equiv) at 0 °C and the mixture was stirred at RT for 6 h. The reaction mixture was diluted with water (50 mL) and the product was extracted into DCM (3 x 20 mL). Combined organic layers were dried over Na_2SO_4 and concetrated under reduced pressure. The crude residue was purified by automated flash column chromatography (Biotage Isolera One) with pre-packed silica gel column using Ethyl acetate/Hexane (2/8) eluents. All the products were subsequently recrystallized from DCM/Hexane.

(*E*)-1-(2,3-dihydro-1*H*-pyrrolo[2,3-*b*]pyridin-1-yl)-3-phenylprop-2-en-1-one (1j):

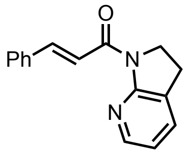

The reaction performed according to the general procedure B afforded 622 mg (83%).
Colorless solid (m.p. 101–103 °C).
^{1}H NMR (400 MHz, 300 K, $CDCl_3$): δ 8.57 (d, *J* = 15.7 Hz, 1H), 8.25 – 8.12 (m, 1H), 7.85 (d, *J* = 15.8 Hz, 1H), 7.72 – 7.59 (m, 2H), 7.48 (dd, *J* = 7.3, 1.5 Hz, 1H), 7.46 – 7.31 (m, 3H), 6.89 (dd, *J* = 7.3, 5.0 Hz, 1H), 4.34 – 4.05 (m, 2H), 3.16 – 2.98 (m, 2H).
^{13}C NMR (101 MHz, 300 K, $CDCl_3$): δ 165.6, 156.4, 146.5, 142.9, 135.9, 133.7, 129.7, 128.8, 128.4, 126.5, 121.0, 118.2, 46.2, 24.4.
IR (thin film): $\tilde{\nu}$ 3080, 1650, 1600, 1587, 1476, 1442, 1419, 1381, 1355, 1300, 1240, 1222, 1020, 983, 763, 703, 567 cm^{-1}.
HRMS (ESI): *m/z* calculated for $C_{16}H_{15}N_2O$ [M+H]$^+$: 251.1179, found: 251.1180.

(*E*)-1-(6-methoxy-2,3-dihydro-1*H*-pyrrolo[2,3-*b*]pyridin-1-yl)-3-phenylprop-2-en-1-one (1l):

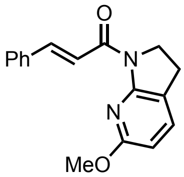

The reaction performed according to the general procedure B afforded 672 mg (80%).
Pale yellow solid (m.p. 126–128 °C).
^{1}H NMR (400 MHz, 300 K, $CDCl_3$): δ 8.52 (dd, *J* = 15.7, 1.1 Hz, 1H), 7.82 (d, *J* = 15.8 Hz, 1H), 7.63 – 7.57 (m, 2H), 7.40 – 7.32 (m, 4H), 6.37 (d, *J* = 8.1 Hz, 1H), 4.26 – 4.18 (m, 2H), 3.99 (s, 3H), 3.01 – 2.96 (m, 2H).
^{13}C NMR (101 MHz, 300 K, $CDCl_3$): δ 165.0, 163.2, 153.8, 142.3, 136.3, 135.9, 129.7, 128.9, 128.0, 120.9, 117.3, 104.0, 53.8, 47.0, 23.7.
IR (thin film): $\tilde{\nu}$ 2935, 2359, 1649, 1613, 1588, 1473, 1448, 1418, 1387, 1344, 1317, 1288, 1253, 1220, 1196, 1160, 1094, 1029, 980, 917, 807, 760, 702, 679, 569, 545 cm^{-1}.
HRMS (ESI): *m/z* calculated for $C_{17}H_{16}N_2O_2Na$ [M+Na]$^+$: 303.1104, found: 303.1112.

4.3. Preparation of amides from Carboxylic acids (Procedure C)

To a solution of the corresponding carboxylic acid (3.6 mmol, 1.2 equiv.), 2,3-dihydro-7-azaindole (360 mg, 3 mmol, 1 equiv.) or 6-Methoxy-2,3-dihydro-7-azaindole (450 mg, 3 mmol, 1 equiv.) and DMAP (184 mg, 1.5 mmol, 50 mol %) in dry DCM (30 mL) was added solid EDCI (695 mg, 3.6 mmol, 1.2 equiv) in one portion and the mixture was stirred at RT 24 h. The reaction mixture was diluted with saturated $NaHCO_3$ (50 mL) and the product was extracted into DCM (3 x 20 mL). Combined organic layers were dried over Na_2SO_4 and concetrated under reduced pressure. The crude residue was purified by automated flash column chromatography (Biotage Isolera One) with pre-packed silica gel column using Ethyl acetate/Hexane (2/8) eluents. All the products were subsequently recrystallized from DCM/Hexane.

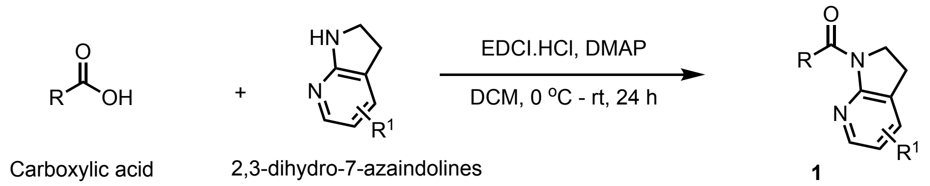

(*E*)-1-(2,3-dihydro-1*H*-pyrrolo[2,3-*b*]pyridin-1-yl)-3-(pyridin-2-yl)prop-2-en-1-one (1k):

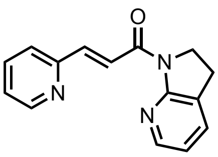

The reaction performed according to the general procedure C afforded 640 mg (85%).
Colorless solid (m.p. 91–93 °C).
^{1}H NMR (400 MHz, 300 K, $CDCl_3$): δ 8.86 (d, *J* = 15.5 Hz, 1H), 8.67 (dd, *J* = 4.8, 1.5 Hz, 1H), 8.21 (d, *J* = 4.9 Hz, 1H), 7.84 (d, *J* = 15.5 Hz, 1H), 7.69 (td, *J* = 7.7, 1.8 Hz, 1H), 7.53 (dd, *J* = 7.9, 1.1 Hz, 1H), 7.47 (dd, *J* = 7.3, 1.5 Hz, 1H), 7.26 – 7.19 (m, 1H), 6.88 (dd, *J* = 7.3, 5.2 Hz, 1H), 4.22 (t, *J* = 8.4 Hz, 2H), 3.09 (td, *J* = 8.4, 8.0, 1.3 Hz, 2H).
^{13}C NMR (101 MHz, 300 K, $CDCl_3$): δ 165.2, 156.2, 154.4, 150.2, 146.7, 141.9, 136.7, 133.7, 126.4, 124.8, 124.1, 123.7, 118.8, 46.2, 24.5.
IR (thin film): $\tilde{\nu}$ 3075, 2965, 1651, 1618, 1599, 1587, 1564, 1467, 1419, 1382, 1351, 1299, 1241, 1223, 1164, 1018, 991, 779, 745, 697, 597, 576 cm^{-1}.
HRMS (ESI): *m/z* calculated for $C_{15}H_{14}N_3O$ [M+H]$^+$: 252.1131, found: 252.1132.

(*E*)-1-(6-methoxy-2,3-dihydro-1*H*-pyrrolo[2,3-*b*]pyridin-1-yl)-3-(pyridin-2-yl)prop-2-en-1-one (1m):

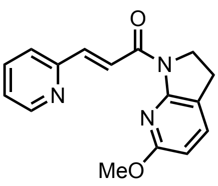

The reaction performed according to the general procedure C afforded 725 mg (86%).
Yellow solid (m.p. 150–152 °C).
^{1}H NMR (400 MHz, 300 K, $CDCl_3$): δ 9.06 (d, *J* = 15.4 Hz, 1H), 8.58 – 8.55 (m, 1H), 7.74 (d, *J* = 15.4 Hz, 1H), 7.69 – 7.62 (m, 1H), 7.42 – 7.34 (m, 2H), 7.22 – 7.16 (m, 1H), 6.35 (dd, *J* = 8.1, 0.9 Hz, 1H), 4.24 – 4.17 (m, 2H), 4.03 (s, 3H), 3.01 – 2.94 (m, 2H).
^{13}C NMR (101 MHz, 300 K, $CDCl_3$): δ 164.8, 163.3, 154.3, 153.6, 150.0, 140.6, 136.6, 136.3, 125.4, 124.2, 123.8, 116.9, 104.2, 54.1, 46.8, 23.8.
IR (thin film): $\tilde{\nu}$ 2931, 2862, 2360, 1650, 1613, 1589, 1563, 1476, 1461, 1418, 1395, 1346, 1320, 1291, 1258, 1224, 1198, 1165, 1101, 1031, 992, 916, 812, 780, 742, 584, 547 cm^{-1}.
HRMS (ESI): *m/z* calculated for $C_{16}H_{15}N_3O_2Na$ [M+Na]$^+$: 304.1056, found: 304.1059.

(*E*)-1-(6-methoxy-2,3-dihydro-1*H*-pyrrolo[2,3-*b*]pyridin-1-yl)-3-(*p*-tolyl)prop-2-en-1-one (1s):

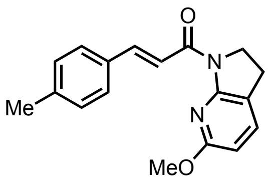

The reaction performed according to the general procedure C afforded 767 mg (87%).
Pale yellow solid (m.p. 140–142 °C).
^{1}H NMR (400 MHz, 300 K, $CDCl_3$): δ 8.49 (d, *J* = 15.8 Hz, 1H), 7.81 (d, *J* = 15.7 Hz, 1H), 7.51 (d, *J* = 8.2 Hz, 2H), 7.40 (d, *J* = 8.2 Hz, 1H), 7.18 (d, *J* = 7.9 Hz, 2H), 6.38 (d, *J* = 8.1 Hz, 1H), 4.29 – 4.19 (m, 2H), 4.01 (s, 3H), 3.00 (td, *J* = 8.3, 1.1 Hz, 2H), 2.37 (s, 3H).
^{13}C NMR (101 MHz, 300 K, $CDCl_3$): δ 165.3, 163.2, 153.9, 142.4, 140.0, 136.3, 133.3, 129.7, 128.0, 119.9, 117.3, 104.0, 53.9, 47.0, 23.8, 21.6.
IR (thin film): $\tilde{\nu}$ 2970, 1645, 1604, 1590, 1514, 1472, 1420, 1389, 1342, 1319, 1287, 1259, 1245, 1220, 1198, 1161, 1093, 1031, 1000, 916, 810, 799, 772, 733, 694, 545, 519, 502 cm^{-1}.
HRMS (ESI): *m*/*z* calculated for $C_{18}H_{19}N_2O_2$ $[M+H]^+$: 295.1441, found: 295.1442.

(*E*)-1-(6-methoxy-2,3-dihydro-1*H*-pyrrolo[2,3-*b*]pyridin-1-yl)-3-(*o*-tolyl)prop-2-en-1-one (1t):

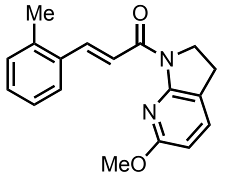

The reaction performed according to the general procedure C afforded 794 mg (90%).
Pale yellow solid (m.p. 136–138 °C).
^{1}H NMR (400 MHz, 300 K, $CDCl_3$): δ 8.49 (d, *J* = 15.6 Hz, 1H), 8.13 (d, *J* = 15.6 Hz, 1H), 7.74 – 7.65 (m, 1H), 7.40 (dd, *J* = 8.2, 1.2 Hz, 1H), 7.27 – 7.12 (m, 3H), 6.38 (d, *J* = 8.1 Hz, 1H), 4.32 – 4.22 (m, 2H), 3.96 (s, 3H), 3.06 – 2.97 (m, 2H), 2.48 (s, 3H).
^{13}C NMR (101 MHz, 300 K, $CDCl_3$): δ 165.2, 163.2, 153.9, 140.0, 137.9, 136.4, 135.0, 130.9, 129.5, 126.2, 126.0, 121.9, 117.3, 104.1, 53.9, 47.1, 23.8, 20.0.
IR (thin film): $\tilde{\nu}$ 2963, 1648, 1612, 1591, 1474, 1448, 1419, 1389, 1342, 1291, 1254, 1219, 1196, 1161, 1094, 1028, 977, 918, 707, 771, 670, 550, 507, 483, cm^{-1}.
HRMS (ESI): *m*/*z* calculated for $C_{18}H_{19}N_2O_2$ $[M+H]^+$: 295.1441, found: 295.1440.

(*E*)-1-(6-methoxy-2,3-dihydro-1*H*-pyrrolo[2,3-*b*]pyridin-1-yl)-3-(4-methoxyphenyl)prop-2-en-1-one (1u):

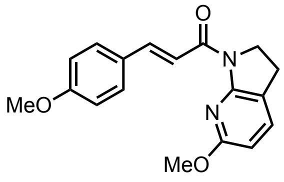

The reaction performed according to the general procedure C afforded 285 mg (92%).
Pale yellow solid (m.p. 153–155 °C).
^{1}H NMR (400 MHz, 300 K, $CDCl_3$): δ 8.41 (d, *J* = 15.7 Hz, 1H), 7.79 (d, *J* = 15.7 Hz, 1H), 7.61 – 7.51 (m, 2H), 7.39 (dt, *J* = 8.1, 1.0 Hz, 1H), 6.94 – 6.81 (m, 2H), 6.37 (d, *J* = 8.0 Hz, 1H), 4.27 – 4.16 (m, 2H), 4.01 (s, 3H), 3.84 (s, 3H), 3.04 – 2.92 (m, 2H).
^{13}C NMR (101 MHz, 300 K, $CDCl_3$): δ 165.4, 163.2, 161.0, 154.0, 142.1, 136.3, 129.6, 128.8, 118.6, 117.3, 114.4, 103.8, 55.5, 53.9, 47.0, 23.8.
IR (thin film): $\tilde{\nu}$ 2936, 1648, 1601, 1511, 1473, 1445, 1419, 1386, 1344, 1303, 1290, 1253, 1196, 1171, 1094, 1029, 983, 919, 825, 808, 549 cm^{-1}.
HRMS (ESI): *m*/*z* calculated for $C_{18}H_{18}N_2O_3Na$ $[M+Na]^+$: 333.1210, found: 333.1215.

(*E*)-1-(6-methoxy-2,3-dihydro-1*H*-pyrrolo[2,3-*b*]pyridin-1-yl)-3-(3-methoxyphenyl)prop-2-en-1-one (1v):

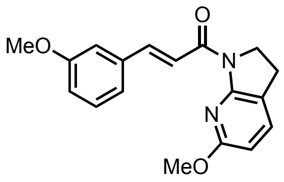

The reaction performed according to the general procedure C afforded 827 mg (89%).
Pale yellow solid (m.p. 129–131 °C).
^{1}H NMR (400 MHz, 300 K, $CDCl_3$): δ 8.51 (d, *J* = 15.7 Hz, 1H), 7.80 (d, *J* = 15.8 Hz, 1H), 7.40 (dt, *J* = 8.1, 1.0 Hz, 1H), 7.31 – 7.25 (m, 1H), 7.22 – 7.19 (m, 1H), 7.14 (t, *J* = 2.0 Hz, 1H), 6.93 – 6.89 (m, 1H), 6.38 (dd, *J* = 8.0, 0.7 Hz, 1H), 4.26 – 4.20 (m, 2H), 4.01 (s, 3H), 3.82 (s, 3H), 3.04 – 2.97 (m, 2H).
^{13}C NMR (101 MHz, 300 K, $CDCl_3$): δ 165.0, 163.2, 160.0, 153.8, 142.4, 137.4, 136.4, 129.9, 121.2, 120.8, 117.3, 115.6, 113.1, 104.1, 55.3, 53.9, 47.1, 23.8.
IR (thin film): $\tilde{\nu}$ 2969, 2932, 1647, 1614, 1587, 1488, 1475, 1464, 1421, 1397, 1346, 1320, 1299, 1273, 1259, 1233, 1219, 1201, 1186, 1167, 1100, 1027, 983, 913, 842, 784, 773, 730, 698, 675, 647, 573, 543, 506 cm^{-1}.
HRMS (ESI): *m*/*z* calculated for $C_{18}H_{19}N_2O_3$ $[M+H]^+$: 311.1390, found: 311.1393.

(*E*)-1-(6-methoxy-2,3-dihydro-1*H*-pyrrolo[2,3-*b*]pyridin-1-yl)-3-(4-(methylthio)phenyl)prop-2-en-1-one (1w):

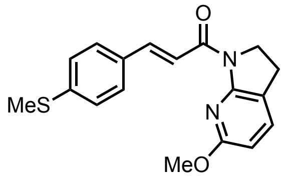

The reaction performed according to the general procedure C afforded 309 mg (95%).
Yellow crystals (m.p. 147–149 °C).
^{1}H NMR (400 MHz, 300 K, $CDCl_3$): δ 8.47 (dd, *J* = 15.7, 1.0 Hz, 1H), 7.77 (d, *J* = 15.7 Hz, 1H), 7.51 (d, *J* = 8.3 Hz, 2H), 7.38 (dq, *J* = 8.2, 0.8 Hz, 1H), 7.24 – 7.17 (m, 2H), 6.37 (dd, *J* = 8.1, 0.9 Hz, 1H), 4.28 – 4.16 (m, 2H), 3.99 (s, 3H), 3.04 – 2.95 (m, 2H), 2.50 (s, 3H).
^{13}C NMR (101 MHz, 300 K, $CDCl_3$): δ 165.1, 163.2, 153.8, 141.8, 141.0, 136.3, 132.6, 128.4, 126.2, 120.0, 117.3, 104.0, 53.9, 47.0, 23.8, 15.4.
IR (thin film): $\tilde{\nu}$ 2919, 1648, 1611, 1590, 1553, 1492, 1473, 1445, 1418, 1386, 1343, 1317, 1292, 1252, 1221, 1196, 1184, 1160, 1115, 1094, 1028, 983, 919, 809, 769, 751, 642, 546 cm^{-1}.
HRMS (ESI): *m/z* calculated for $C_{18}H_{18}N_2O_2SNa$ [M+Na]$^+$: 349.0981, found: 349.0982.

(*E*)-3-(4-fluorophenyl)-1-(6-methoxy-2,3-dihydro-1*H*-pyrrolo[2,3-*b*]pyridin-1-yl)prop-2-en-1-one (1x):

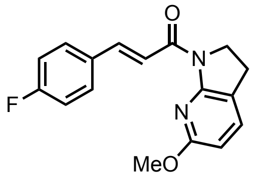

The reaction performed according to the general procedure C afforded 840 mg (94%).
Pale yellow solid (m.p. 162–164 °C).
^{1}H NMR (400 MHz, 300 K, $CDCl_3$): δ 8.45 (d, *J* = 15.7 Hz, 1H), 7.79 (d, *J* = 15.8 Hz, 1H), 7.58 (dd, *J* = 8.7, 5.5 Hz, 2H), 7.40 (dd, *J* = 8.1, 1.1 Hz, 1H), 7.06 (t, *J* = 8.6 Hz, 2H), 6.39 (d, *J* = 8.1 Hz, 1H), 4.28 – 4.19 (m, 2H), 3.99 (s, 3H), 3.01 (td, *J* = 8.4, 1.0 Hz, 2H).
^{13}C NMR (101 MHz, 300 K, $CDCl_3$): δ 164.9, 163.6 (d, *J* = 250.4 Hz), 163.2, 153.8, 141.1, 136.4, 132.3 (d, *J* = 3.4 Hz), 129.8 (d, *J* = 8.4 Hz), 120.7 (d, *J* = 2.3 Hz), 117.4, 116.0 (d, *J* = 21.9 Hz), 104.1, 53.8, 47.1, 23.8.
^{19}F NMR (376 MHz, $CDCl_3$): δ -110.82.
IR (thin film): $\tilde{\nu}$ 2962, 1645, 1611, 1590, 1510, 1473, 1422, 1392, 1343, 1318, 1294, 1259, 1219, 1197, 1162, 1028, 997, 833, 807, 772, 669, 545, 511 cm^{-1}.
HRMS (ESI): *m/z* calculated for $C_{17}H_{16}N_2O_2F$ [M+H]$^+$: 299.1190, found: 299.1191.

(*E*)-3-(4-bromophenyl)-1-(6-methoxy-2,3-dihydro-1*H*-pyrrolo[2,3-*b*]pyridin-1-yl)prop-2-en-1-one (1y):

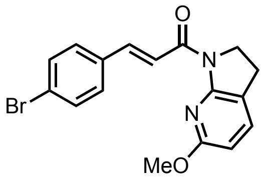

The reaction performed according to the general procedure C afforded 945 mg (88%).
Pale yellow solid (m.p. 181–183 °C).
^{1}H NMR (400 MHz, 300 K, $CDCl_3$): δ 8.51 (d, *J* = 15.7 Hz, 1H), 7.75 (d, *J* = 15.8 Hz, 1H), 7.54 – 7.44 (m, 4H), 7.43 – 7.38 (m, 1H), 6.39 (d, *J* = 8.1 Hz, 1H), 4.28 – 4.18 (m, 2H), 3.98 (s, 3H), 3.06 – 2.95 (m, 2H).
^{13}C NMR (101 MHz, 300 K, $CDCl_3$): δ 164.8, 163.2, 153.8, 140.9, 136.5, 135.0, 132.1, 129.4, 123.8, 121.7, 117.4, 104.2, 53.9, 47.1, 23.8.
IR (thin film): $\tilde{\nu}$ 2938, 1645, 1609, 1587, 1563, 1487, 1472, 1445, 1417, 1386, 1342, 1318, 1295, 1257, 1246, 1218, 1197, 1161, 1093, 1069, 1030, 1008, 916, 813, 772, 668, 618, 545, 493 cm^{-1}.
HRMS (ESI): *m/z* calculated for $C_{17}H_{16}N_2O_2Br$ [M+H]$^+$: 359.0390, found: 359.0391.

(*E*)-4-(3-(6-methoxy-2,3-dihydro-1*H*-pyrrolo[2,3-*b*]pyridin-1-yl)-3-oxoprop-1-en-1-yl)benzonitrile (1z):

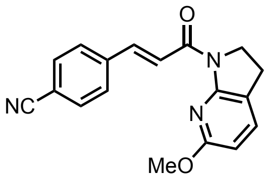

The reaction performed according to the general procedure C afforded 823 mg (90%).
Yellow solid (m.p. 200–202 °C).
^{1}H NMR (400 MHz, 300 K, $CDCl_3$): δ 8.61 (d, *J* = 15.8 Hz, 1H), 7.79 (d, *J* = 15.8 Hz, 1H), 7.67 (d, *J* = 0.9 Hz, 4H), 7.43 (dt, *J* = 8.1, 1.0 Hz, 1H), 6.42 (d, *J* = 8.1 Hz, 1H), 4.28 – 4.21 (m, 2H), 3.97 (s, 3H), 3.03 (td, *J* = 8.3, 1.1 Hz, 2H).
^{13}C NMR (101 MHz, 300 K, $CDCl_3$): δ 164.1, 163.3, 153.6, 140.4, 139.8, 136.6, 132.7, 128.3, 124.5, 118.7, 117.5, 112.8, 104.5, 53.9, 47.1, 23.8.
IR (thin film): $\tilde{\nu}$ 2962, 2928, 2225, 1648, 1608, 1587, 1506, 1473, 1417, 1395, 1342, 1293, 1255, 1245, 1198, 1097, 1011, 957, 915, 825, 815, 772, 545, 509 cm^{-1}.
HRMS (ESI): *m/z* calculated for $C_{18}H_{15}N_3O_2Na$ [M+Na]$^+$: 328.1056, found: 328.1058.

(*E*)-1-(6-methoxy-2,3-dihydro-1*H*-pyrrolo[2,3-*b*]pyridin-1-yl)-2-methylbut-2-en-1-one (1ab):

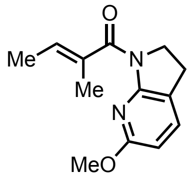

The reaction performed according to the general procedure C afforded 507 mg (73%).

Colorless solid (m.p. 50–52 °C).

^{1}H NMR (400 MHz, 300 K, CDCl$_3$): δ 7.33 (dd, *J* = 8.1, 0.9 Hz, 1H), 6.30 (dd, *J* = 8.0, 0.8 Hz, 1H), 5.84 – 5.75 (m, 1H), 4.14 – 4.02 (m, 2H), 3.80 (s, 3H), 3.03 – 2.92 (m, 2H), 1.95 (s, 3H), 1.73 (dd, *J* = 6.9, 1.1 Hz, 3H).

^{13}C NMR (101 MHz, 300 K, CDCl$_3$): δ 171.8, 163.1, 153.6, 135.8, 134.7, 126.5, 116.4, 103.9, 53.4, 47.2, 24.2, 14.4, 13.4.

IR (thin film): $\tilde{\nu}$ 2915, 1638, 1608, 1590, 1473, 1416, 1385, 1342, 1325, 1309, 1292, 1253, 1192, 1155, 1091, 1024, 917, 808, 742, 658, 565 cm^{-1}.

HRMS (ESI): *m/z* calculated for $C_{13}H_{16}N_2O_2Na^+$ [M+Na]$^+$: 255.1104, found: 255.1106.

Diethyl (2-(6-methoxy-2,3-dihydro-1*H*-pyrrolo[2,3-*b*]pyridin-1-yl)-2-oxoethyl)phosphonate (10):

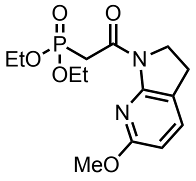

The reaction performed according to the general procedure C (30 mmol scale, DCM: 300 mL) afforded 8.06 g (82%).

Colorless solid (m.p. 44–46 °C).

^{1}H NMR (400 MHz, 300 K, CDCl$_3$): δ 7.38 (dd, *J* = 8.2, 1.0 Hz, 1H), 6.36 (dd, *J* = 8.2, 1.0 Hz, 1H), 4.20 (s, 1H), 4.16 – 4.05 (m, 7H), 3.92 (s, 3H), 3.05 – 2.85 (m, 2H), 1.24 (t, *J* = 7.1 Hz, 6H).

^{13}C NMR (101 MHz, 300 K, CDCl$_3$): δ 163.5 (d, *J* = 6.8 Hz), 163.0, 153.0, 136.5, 116.8, 104.5, 62.3 (d, *J* = 6.3 Hz), 54.1, 46.9, 35.1 (d, *J* = 133.4 Hz), 23.5, 16.4 (d, *J* = 6.3 Hz).

^{31}P NMR (162 MHz, 300 K, CDCl$_3$): δ 21.79.

IR (thin film): $\tilde{\nu}$ 3476, 2982, 2938, 1656, 1611, 1589, 1526, 1477, 1422, 1335, 1314, 1294, 1255, 1197, 1151, 1095, 1054, 1024, 967, 908, 810, 782, 670, 635, 603 cm^{-1}.

HRMS (ESI): *m/z* calculated for $C_{14}H_{22}N_2O_5P$ [M+H]$^+$: 329.1261, found: 329.1259.

*4.4. Preparation of amides from **10** using Horner–Wadsworth–Emmons (HWE) reaction (Procedure D)*

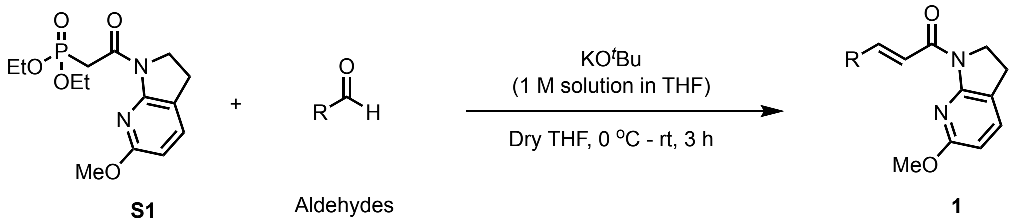

Potassium tert-butoxide solution (1 M solution in THF, 1.0 mL, 1.0 mmol, 1.0 equiv.) was slowly added to diethyl (2-(6-methoxy-2,3-dihydro-1*H*-pyrrolo[2,3-*b*]pyridin-1-yl)-2-oxoethyl)phosphonate **10** (1.0 mmol, 1.0 equiv) in anhydrous THF (15 mL) under a positive argon atmosphere at 0 °C. The resulting mixture allowed stirring for 1 h, followed by slow addition of desired aldehyde (1.5 mmol, 1.5 equiv) in 3 mL THF over 5 min, the resulting mixture stirred under an inert atmosphere for 2 h at 0 °C. The progress of the reaction monitored by TLC analysis (staining with 2, 4-DNP / $KMnO_4$); after complete consumption of the starting material, the reaction was quenched by adding saturated ammonium chloride solution (10 mL) and extracted with ethyl acetate (15 mL x 3). Combined organic layer was washed with brine (20 mL), dried over anhydrous Na_2SO_4, and concentrated *in vacuo*. Purification of the crude product by automated flash column chromatography using Hexanes/EtOAc (8/2) solvent system afforded pure α, β-unsaturated amides **1**. All the products were subsequently recrystallized from DCM/Hexane.

(*E*)-1-(6-methoxy-2,3-dihydro-1*H*-pyrrolo[2,3-*b*]pyridin-1-yl)pent-2-en-1-one (1n):

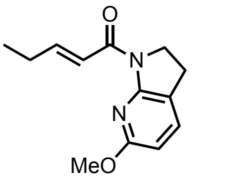

The reaction performed according to the general procedure D afforded 200 mg (86%).
Colorless solid (m.p. 80–82 °C).
^{1}H NMR (400 MHz, 300 K, $CDCl_3$): δ 7.81 (dt, *J* = 15.4, 1.8 Hz, 1H), 7.37 (dq, *J* = 8.1, 0.9 Hz, 1H), 7.16 (dt, *J* = 15.4, 6.1 Hz, 1H), 6.34 (dd, *J* = 8.1, 0.7 Hz, 1H), 4.22 – 4.12 (m, 2H), 3.93 (s, 3H), 2.97 (td, *J* = 8.4, 1.0 Hz, 2H), 2.31 (qdd, *J* = 7.5, 6.0, 1.8 Hz, 2H), 1.13 (t, *J* = 7.4 Hz, 3H).
^{13}C NMR (101 MHz, 300 K, $CDCl_3$): δ 165.4, 163.2, 153.9, 148.2, 136.2, 122.5, 117.2, 103.7, 53.8, 46.9, 25.9, 23.8, 12.6.
IR (thin film): $\tilde{\nu}$ 2971, 2942, 2905, 1659, 1610, 1590, 1479, 1469, 1455, 1419, 1388, 1375, 1335, 1292, 1253, 1221, 1194, 1163, 1103, 1027, 978, 914, 857, 829, 774, 732, 654, 548, 522 cm^{-1}.
HRMS (ESI): *m*/*z* calculated for $C_{13}H_{17}N_2O_2$ $[M+H]^+$: 233.1285, found: 233.1286.

(*E*)-1-(6-methoxy-2,3-dihydro-1*H*-pyrrolo[2,3-*b*]pyridin-1-yl)hept-2-en-1-one (1o):

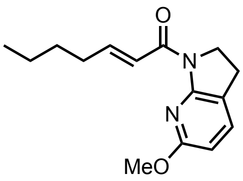

The reaction performed according to the general procedure D afforded 231 mg (89%).
Colorless solid (m.p. 37–39 °C).
^{1}H NMR (400 MHz, 300 K, $CDCl_3$): δ 7.81 (dt, *J* = 15.4, 1.6 Hz, 1H), 7.36 (dt, *J* = 8.0, 1.0 Hz, 1H), 7.14 – 7.03 (m, 1H), 6.33 (d, *J* = 8.1 Hz, 1H), 4.20 – 4.11 (m, 2H), 3.92 (s, 3H), 3.02 – 2.90 (m, 2H), 2.31 – 2.22 (m, 2H), 1.56 – 1.45 (m, 2H), 1.42 – 1.29 (m, 2H), 0.90 (t, *J* = 7.3 Hz, 3H).
^{13}C NMR (101 MHz, 300 K, $CDCl_3$): δ 165.3, 163.1, 153.9, 146.9, 136.2, 123.4, 117.1, 103.7, 53.8, 46.8, 32.7, 30.5, 23.7, 22.4, 14.0.
IR (thin film): $\tilde{\nu}$ 2955, 2929, 2860, 1659, 1624, 1590, 1473, 1418, 1387, 1345, 1292, 1253, 1196, 1160, 1093, 1025, 985, 808, 640 cm^{-1}.
HRMS (ESI): *m*/*z* calculated for $C_{15}H_{20}N_2O_2Na$ $[M+Na]^+$: 283.1417, found: 283.1427.

(*E*)-1-(6-methoxy-2,3-dihydro-1*H*-pyrrolo[2,3-*b*]pyridin-1-yl)-5-methylhex-2-en-1-one (1p):

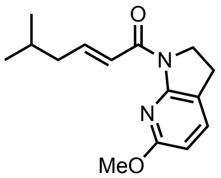

The reaction performed according to the general procedure D afforded 234 mg (90%).

Colorless solid (m.p. 69–71 °C).

^{1}H NMR (400 MHz, 300 K, $CDCl_3$): δ 7.79 (dt, *J* = 15.3, 1.5 Hz, 1H), 7.36 (dt, *J* = 8.2, 1.0 Hz, 1H), 7.16 – 6.99 (m, 1H), 6.34 (d, *J* = 8.1 Hz, 1H), 4.24 – 4.02 (m, 2H), 3.91 (s, 3H), 3.02 – 2.91 (m, 2H), 2.22 – 2.09 (m, 2H), 1.88 – 1.74 (m, 1H), 0.94 (d, *J* = 6.7 Hz, 6H).

^{13}C NMR (101 MHz, 300 K, $CDCl_3$): δ 165.2, 163.1, 153.9, 145.6, 136.2, 124.6, 117.1, 103.7, 53.8, 46.8, 42.4, 28.1, 23.8, 22.6.

IR (thin film): $\tilde{\nu}$ 2955, 1659, 1624, 1590, 1474, 1418, 1387, 1336, 1292, 1253, 1160, 1093, 1025, 983, 806 cm^{-1}.

HRMS (ESI): *m*/*z* calculated for *m*/*z* calculated for $C_{15}H_{20}N_2O_2Na$ [M+Na]$^+$: 283.1417, found: 283.1424.

(*E*)-3-cyclopropyl-1-(6-methoxy-2,3-dihydro-1*H*-pyrrolo[2,3-*b*]pyridin-1-yl)prop-2-en-1-one (1q):

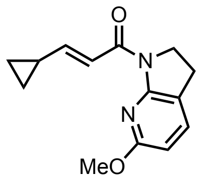

The reaction performed according to the general procedure D afforded 231 mg (95%).

Colorless solid (m.p. 117–119 °C).

^{1}H NMR (400 MHz, 300 K, $CDCl_3$): δ 7.94 (d, *J* = 15.2 Hz, 1H), 7.37 (dt, *J* = 8.2, 1.1 Hz, 1H), 6.60 (dd, *J* = 15.2, 9.9 Hz, 1H), 6.34 (d, *J* = 8.1 Hz, 1H), 4.19 – 4.12 (m, 2H), 3.95 (s, 3H), 3.00 – 2.93 (m, 2H), 1.69 – 1.57 (m, 1H), 0.97 – 0.90 (m, 2H), 0.71 – 0.65 (m, 2H).

^{13}C NMR (101 MHz, $CDCl_3$): δ 165.5, 163.1, 154.0, 151.6, 136.2, 120.4, 117.2, 103.5, 53.82, 46.8, 23.8, 15.1, 8.8.

IR (thin film): $\tilde{\nu}$ 2916, 1653, 1615, 1590, 1473, 1422, 1399, 1367, 1330, 1317, 1286, 1259, 1200, 1187, 1159, 1100, 1020, 982, 940, 871, 808, 792, 940, 871, 808, 792, 754, 703, 665, 643, 547 cm^{-1}.

HRMS (ESI): *m*/*z* calculated for *m*/*z* calculated for $C_{14}H_{16}N_2O_2Na$ [M+Na]$^+$: 267.1104, found: 267.1110.

(*E*)-3-cyclohexyl-1-(6-methoxy-2,3-dihydro-1*H*-pyrrolo[2,3-*b*]pyridin-1-yl)prop-2-en-1-one (1r):

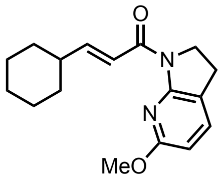

The reaction performed according to the general procedure D afforded 249 mg (87%).

Colorless solid (m.p. 87–89 °C).

^{1}H NMR (400 MHz, 300 K, $CDCl_3$): δ 7.77 (dd, *J* = 15.5, 1.6 Hz, 1H), 7.36 (dt, *J* = 8.1, 1.0 Hz, 1H), 7.08 (dd, *J* = 15.5, 6.2 Hz, 1H), 6.34 (d, *J* = 8.1 Hz, 1H), 4.22 – 4.10 (m, 2H), 3.93 (s, 3H), 3.00 – 2.91 (m, 2H), 2.25 – 2.14 (m, 1H), 1.91 – 1.81 (m, 2H), 1.80 – 1.73 (m, 2H), 1.71 – 1.64 (m, 1H), 1.38 – 1.11 (m, 5H).

^{13}C NMR (101 MHz, 300 K, $CDCl_3$): δ 165.6, 163.1, 153.9, 152.1, 136.2, 120.9, 117.2, 103.7, 53.9, 46.9, 40.8, 32.2, 26.2, 26.0, 23.7.

IR (thin film): $\tilde{\nu}$ 2924, 2851, 1657, 1621, 1590, 1473, 1448, 1417, 1385, 1341, 1293, 1252, 1196, 1160, 1093, 1027, 986, 808, 640 cm^{-1}.

HRMS (ESI): *m*/*z* calculated for $C_{17}H_{22}N_2O_2Na$ [M+Na]$^+$: 309.1573, found: 309.1578.

(*E*)-3-(furan-2-yl)-1-(6-methoxy-2,3-dihydro-1*H*-pyrrolo[2,3-*b*]pyridin-1-yl)prop-2-en-1-one (1aa):

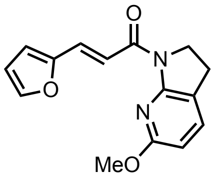

The reaction performed according to the general procedure D afforded 216 mg (80%).
Yellow solid (m.p. 108–110 °C).

^{1}H NMR (400 MHz, 300 K, $CDCl_3$): δ 8.50 (d, *J* = 15.5 Hz, 1H), 7.56 (d, *J* = 15.6 Hz, 1H), 7.42 (d, *J* = 1.8 Hz, 1H), 7.38 (dd, *J* = 8.2, 1.1 Hz, 1H), 6.58 (d, *J* = 3.4 Hz, 1H), 6.48 – 6.44 (m, 1H), 6.37 (d, *J* = 8.1 Hz, 1H), 4.25 – 4.18 (m, 2H), 4.03 (s, 3H), 3.03 – 2.97 (m, 2H).

^{13}C NMR (101 MHz, 300 K, $CDCl_3$): δ 165.0, 163.2, 153.8, 152.5, 144.0, 136.3, 128.8, 118.9, 117.0, 113.9, 112.3, 104.0, 54.0, 46.9, 23.8.

IR (thin film): $\tilde{\nu}$ 2938, 2359, 1649, 1611, 1557, 1477, 1418, 1383, 1338, 1317, 1290, 1248, 1197, 1162, 1095, 1017, 977, 916, 882, 802, 751, 717, 670, 643, 594 cm^{-1}.

HRMS (ESI): *m*/*z* calculated for $C_{15}H_{14}N_2O_3Na$ $[M+Na]^+$: 293.0897, found: 293.0899.

4.5. Synthesis of Amine derivatives (Procedure E)

To a solution of amine (9.3 mmol, 1 equiv) in dry THF (0.3 M) under Argon at –78 °C was added a solution of ^{n}BuLi in hexanes (2.6 M, 4 mL, 10.23 mmol, 1.1 equiv) over the period of ~3 min. The solution was then allowed to warm to room temperature for 24 h. Then, (iodomethyl)trimethylsilane (10.2 mmol, 1.6 mL, 1.1 equiv) was added slowly, and the resulting solution was stirred for additional 12 h. The reaction was then quenched by slow addition of a saturated solution of NH_4Cl (10 mL) and extracted with EtOAc (3 x 20 mL). The combined organic layers were dried over Na_2SO_4 and concentrated *in vacuo* to afford a crude product. That was purified by automated flash column chromatography (Biotage Isolera One) with pre-packed silica gel column using Hexane/EtOAc (99/01) as eluents.

***N*-methyl-*N*-((trimethylsilyl)methyl)aniline (2a):**

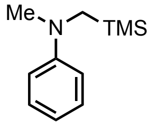

The reaction performed according to the general procedure E afforded 1.40 g (78%).
Pale yellow oil.
^{1}H NMR (400 MHz, $CDCl_3$): δ 7.37 – 7.10 (m, 2H), 6.92 – 6.48 (m, 3H), 2.98 (s, 3H), 2.91 (s, 2H), 0.15 (s, 9H).
^{13}C NMR (101 MHz, $CDCl_3$): δ 150.7, 129.1, 115.3, 112.0, 44.1, 40.3, -1.0.
The obtained data is in accordance with the literature data.[3]

***N*-benzyl-*N*-((trimethylsilyl)methyl)aniline (2b):**

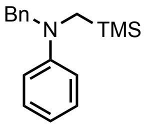

The reaction performed according to the general procedure E afforded 1.90 g (76%).
Pale yellow oil.
^{1}H NMR (400 MHz, $CDCl_3$): δ 7.36 – 7.29 (m, 2H), 7.27 – 7.15 (m, 5H), 6.77 – 6.51 (m, 3H), 4.56 (s, 2H), 3.00 (s, 2H), 0.10 (s, 9H).
^{13}C NMR (101 MHz, $CDCl_3$): δ 149.7, 138.8, 129.1, 128.7, 126.8, 126.8, 115.5, 112.2, 56.0, 42.4, -0.9.
The obtained data is in accordance with the literature data.[3]

***N*-isopropyl-*N*-((trimethylsilyl)methyl)aniline (2c):**

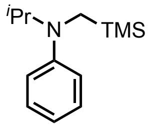

The reaction performed according to the general procedure E afforded 1.50 g (73%).
Pale yellow oil.
^{1}H NMR (400 MHz, $CDCl_3$): δ 7.27 – 7.18 (m, 2H), 6.83 – 6.76 (m, 2H), 6.75 – 6.67 (m, 1H), 4.11 – 3.99 (m, 1H), 2.62 (s, 2H), 1.15 (d, *J* = 6.6 Hz, 6H), 0.05 (s, 9H).
^{13}C NMR (101 MHz, $CDCl_3$): δ 150.6, 128.8, 116.6, 115.6, 51.3, 33.8, 19.7, -0.9.
The obtained data is in accordance with the literature data.[3]

***N*-((trimethylsilyl)methyl)aniline (2d):**

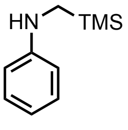

The reaction performed according to the general procedure E afforded 1.16 g (70%).
Pale yellow oil.
^{1}H NMR (400 MHz, $CDCl_3$): δ 7.26 – 7.15 (m, 2H), 6.80 – 6.62 (m, 3H), 3.49 (bs, 1H), 2.54 (s, 2H), 0.18 (d, *J* = 1.1 Hz, 9H).
^{13}C NMR (101 MHz, $CDCl_3$): δ 150.7, 129.3, 117.1, 112.5, 33.7, -2.6.
The obtained data is in accordance with the literature data.[3]

2-fluoro-*N*-methyl-*N*-((trimethylsilyl)methyl)aniline (2e):

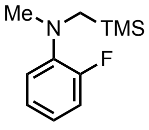

The reaction performed according to the general procedure E afforded 1.45 g (74%).
Pale yellow oil.
^{1}H NMR (400 MHz, $CDCl_3$): δ 7.06 – 6.90 (m, 3H), 6.85 – 6.77 (m, 1H), 2.84 (s, 3H), 2.71 (s, 2H), 0.07 (s, 9H).
^{13}C NMR (101 MHz, $CDCl_3$): δ 155.4 (d, *J* = 244.7 Hz), 142.5 (d, *J* = 8.4 Hz), 124.2 (d, *J* = 3.4 Hz), 120.7 (d, *J* = 7.8 Hz), 119.0 (d, *J* = 3.5 Hz), 116.2 (d, *J* = 21.0 Hz), 47.3 (d, *J* = 4.0 Hz), 43.5 (d, *J* = 2.8 Hz), -1.12.
^{19}F NMR (376 MHz, $CDCl_3$): δ -121.92.
The obtained data is in accordance with the literature data.[3]

5. Photochemical reactions

5.1: Reaction optimization

I: Reaction optimization at room temperature / 5 °C (placed in a cold storage room):

A dry 4 mL tube was charged with a photocatalyst **PC** (0.01 equiv., 1.0 mol%), Metal salt (0.15 equiv., 15 mol%), Ligand (0.20 equiv., 20 mol%) and TBACl (8.35 mg, 0.30 equiv., 30 mol%) in the Glove box. To the mixture was added anhydrous solvent(s) (2 mL) via syringe with a stainless-steel needle at room temperature under positive Argon pressure. The reaction mixture was charged with the acceptor **1a** (18.8 mg, 1.0 equiv, 0.1 mmol) and stirred for 30 min. The reaction was carefully degassed by 3 freeze/pump/thaw cycles under Argon in the dark. Then, the amine **2a** (50 μL, 2.5 equiv., 0.25 mmol) was added slowly to the reaction mixture and irradiated by blue light (λ_{max} = 455 nm). The yield was determined by ^{1}H NMR spectroscopy of the crude product using 1,1,2,2-tetrachloroethane (100 μL, 1M solution in $CDCl_3$) as an internal standard. The purification was done by using Preparative Thin Layer Chromatography (PTLC) using Hexane/EtOAc (~85/15) eluents. The pure product **3** was obtained by filtering through glass frit funnel using DCM as an eluent. The enantiomeric excess (*ee*) was determined by chiral HPLC analysis.

II: Reaction optimization at -20 °C:

An oven dried 10 mL Schlenk tube was charged with Photocatalyst **PC** (0.01 equiv., 1.0 mol%), Metal salt (0.15 equiv., 15 mol%), Ligand (0.20 equiv., 20 mol%) in the Glove box. To the mixture was added anhydrous solvent(s) (2 mL) via syringe with a stainless-steel needle at room temperature under positive Argon pressure. The resulting solution was stirred for 30 min and transferred to another Schlenk tube containing the acceptor **1a** (18.8 mg, 1.0 equiv, 0.1 mmol). The reaction was carefully degassed by 3 freeze/pump/thaw cycles under Argon in the dark. The resulting reaction mixture was then allowed to stir for additional 30 min at room temperature before cooling down to -20 °C. Then, the amine **2a** (50 μL, 2.5 equiv., 0.25 mmol) was added slowly to the reaction mixture and allowed to equilibrate for 15 min at -20 °C. This mixture was then irradiated by blue light (λ_{max} = 448 nm). The yield was determined by ^{1}H NMR spectroscopy of the crude product using 1,1,2,2-tetrachloroethane (100 μL, 1M solution in $CDCl_3$) as an internal standard. The purification was done by using Preparative Thin Layer Chromatography (PTLC) using Hexane/EtOAc (~85/15) eluents. The pure product **3** was obtained by filtering through glass frit funnel using $CHCl_3$ as an eluent. The enantiomeric excess (*ee*) was determined by chiral HPLC analysis.

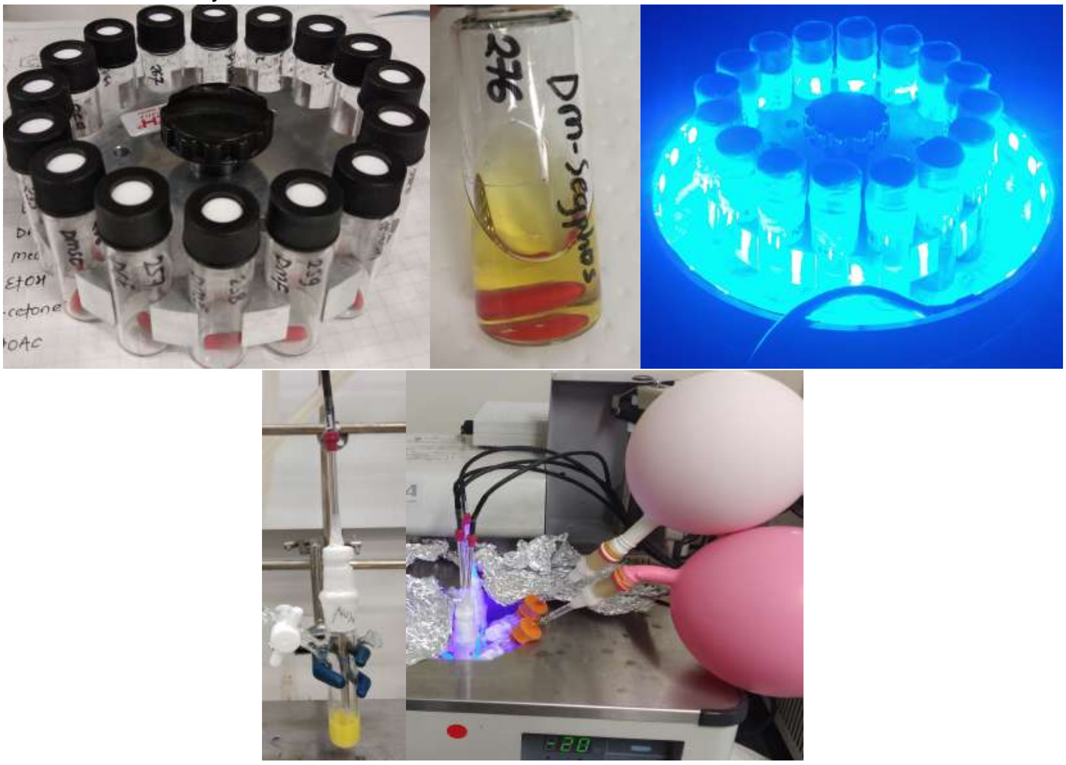

Fig. S1: Photochemical reaction set-up for room temperature / 5 °C (top (λ_{max} = 455 nm) and -20 °C (bottom (λ_{max} = 448 nm).

[A] Metal and Ligand optimization at room temperature (~23 °C)

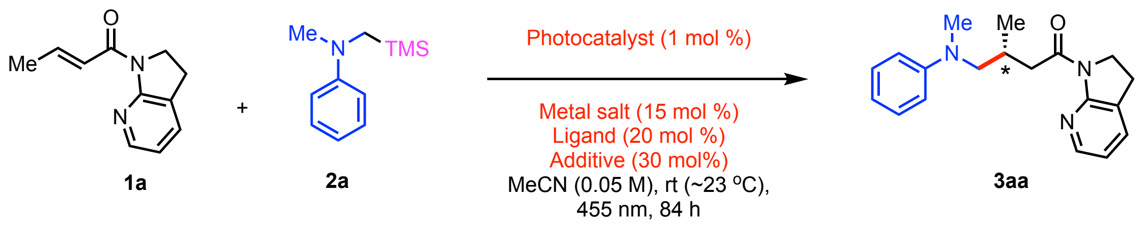

Entry	Metal salt	Ligand	Photocatalyst	PTC (30 mol%)	NMR Yield (%)[b]	*ee* (%)[c]
01	None	None	$[Ru(bpy)_3Cl_2$ (**PC1**)	TBACl	28	ND
02	$Sc(OTf)_3$	(*S*,*S*)-^{t}Bu-PyBox (**L1**)	none	TBACl	10	0
03	none	none	$[Ru(bpy)_3Cl_2$ (**PC1**)	none	26	ND
04	$Sc(OTf)_3$	(*S*,*S*)-^{t}Bu-PyBox (**L1**)	$[Ru(bpy)_3Cl_2$ (**PC1**)	TBACl	85	0
05	$Sc(OTf)_3$	(*S*,*S*)-^{t}Bu-PyBox (**L1**)	$[Ru(bpy)_3Cl_2$ (**PC1**)	none	83	0
06	none	(*S*,*S*)-^{t}Bu-PyBox (**L1**)	$[Ru(bpy)_3Cl_2$ (**PC1**)	TBACl	27	ND
07	$Sc(OTf)_3$	none	$[Ru(bpy)_3Cl_2$ (**PC1**)	TBACl	35	ND
08	$[Cu(MeCN)_4]PF_6$	(*S*,*S*)-^{t}Bu-PyBox (**L1**)	$[Ru(bpy)_3Cl_2$ (**PC1**)	none	traces	ND
**09**	**$[Cu(MeCN)_4]PF_6$**	**(*R*)-DM-Segphos (L2)**	**$[Ir(ppy)_2dtb\text{-}bpy]PF_6$ (PC2)**	**none**	**72**	**30**

Unless otherwise stated, these are the common conditions maintained for all the optimizations: [a]*1a: 0.1 mmol, 2a: 0.25 mmol, Metal salt (15 mol %), Ligand (20 mol %), Photocatalyst (1 mol %), MeCN (0.05 M), rt (~23 °C), 84 h. [b] Determined by ^{1}H-NMR analysis of the crude reaction mixture with 1,1,2,2-tetrachloroethane as an internal standard. [c] Determined by chiral stationary phase HPLC analysis. (Abbreviations: ee = enantiomeric excess; ND = Not determined; PTC = Phase Transfer Catalyst; TBACl = Tetrabutyl Ammonium Chloride).*

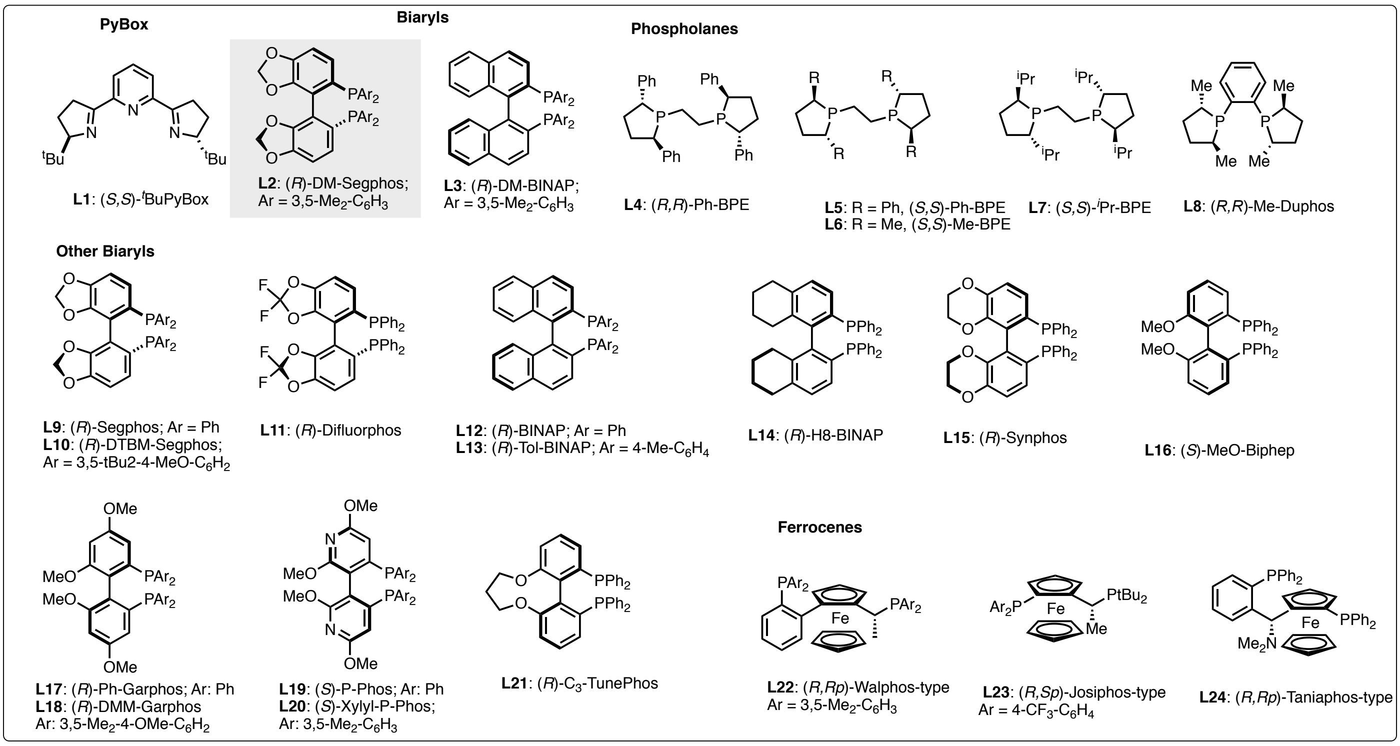

[B] Ligand optimization at low temperature (~5 °C)

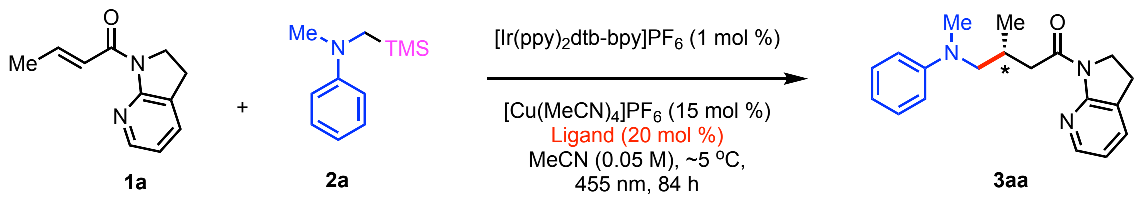

Entry	Ligand	NMR Yield (%)	*ee* (%)
**01**	**(*R*)-DM-Segphos (L2)**	**61**	**70**
02	(*R*)-DM-BINAP (**L3**)	49	52
03	(*R,R*)-Ph-BPE (**L4**)	43	-44
04	(*S,S*)-Ph-BPE (**L5**)	40	50
05	(*S,S*)-Me-BPE (**L6**)	10	3
06	(*S,S*)-^{i}Pr-BPE (**L7**)	10	8
07	(*R,R*)-Me-Duphos (**L8**)	10	-5
08	(*R*)-Segphos (**L9**)	68	67
09	(*R*)-DTBM-Segphos (**L10**)	10	3
10	(*R*)-Difluorphos (**L11**)	40	38
11	(*R*)-BINAP (**L12**)	45	45
12	(*R*)-Tol-BINAP (**L13**)	28	63
13	(*R*)-H8-BINAP (**L14**)	25	4
14	(*R*)-Synphos (**L15**)	39	29
15	(*S*)-MeO-Biphep (**L16**)	49	-29
16	(*R*)-Ph-Garphos (**L17**)	63	42
17	(*R*)-DMM-Garphos (**L18**)	41	36
18	(*S*)-P-Phos (**L19**)	55	-39
19	(*S*)-Xylyl-P-Phos (**L20**)	53	-35
20	(*R*)-C3-Tunephos (**L21**)	48	31
21	(*R,R$_p$*)-Walphos-type (**L22**)	13	25
22	(*R,S$_p$*)-Josiphos-type (**L23**)	traces	ND
23	(*R,R$_p$*)-Taniaphos-type (**L24**)	traces	ND

[C] Solvent optimization at low temperature (~5 °C)

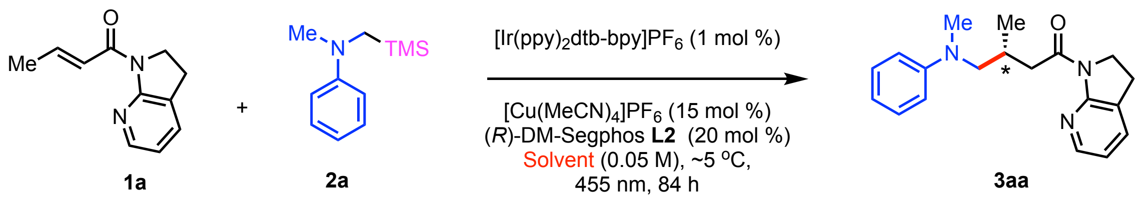

Entry	Solvent (0.05 M)	NMR Yield (%)	*ee* (%)
**01**	**MeCN**	**61**	**70**
02	DCM	10	ND
03	DCE	10	ND
04	$CHCl_3$	traces	ND
05	1,4-Dioxane	traces	ND
06	DMSO	traces	ND
07	DMF	traces	ND
08	MeOH	10	ND
**09**	**EtOH**	**73**	**74**
10	Acetone	49	53
11	EtOAc	18	ND
12	^{i}PrOAc	22	ND
13	THF	68	20
14	Diethyl Ether	8	ND

[D] Solvent mixture optimization at low temperature (~5 °C)

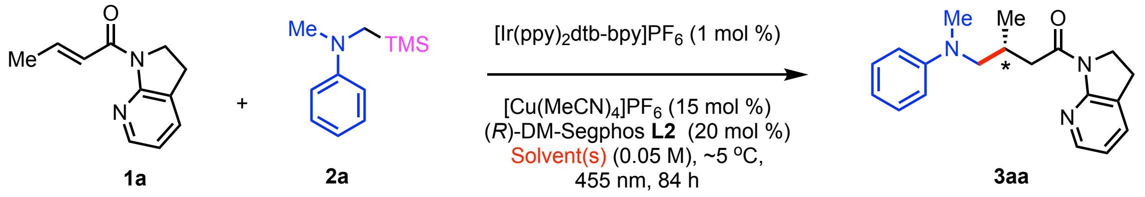

Entry	Solvent(s) (1 / 1, 0.05 M)	NMR Yield (%)	*ee* (%)
**01**	**MeCN**	**62**	**89**
02*	EtOH	traces	ND
03	EtOH / MeCN	68	66
04	EtOH / MeOH	67	63
05	EtOH / THF	75	73
06	EtOH / DMF	44	46
**07**	**EtOH / DME**	**77**	**76**
08	EtOH / Diethyl Ether	65	77
09	EtOH / Water	10	71
10	EtOH / Acetone	80	74
11	EtOH / EtOAc	79	74
12	EtOH / DMSO	45	72

*Reaction mixture was turbid (solubility issues, Fig. S2)

Left: Clear reaction mixture in MeCN at -20 °C (after 1 h)

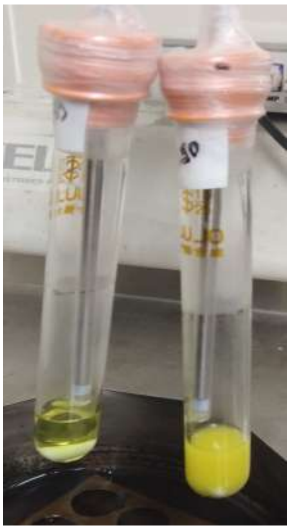

Right: Turbid reaction mixture in EtOH at -20 °C (after 1 h).

Fig. S2. Photochemical reaction in MeCN and EtOH at -20 °C (after 1 h)

[E] Solvent mixture ratio optimization at low temperature (-20 °C)

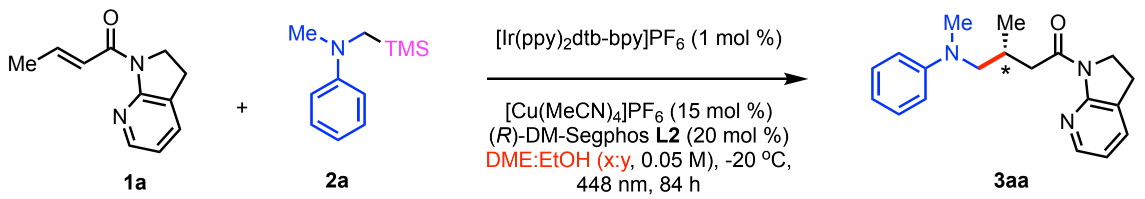

Entry	EtOH / DME (x / y, 0.05 M)	NMR Yield (%)	*ee* (%)
**01**	**1 / 3**	**80**	**89**
02	3/ 1	49	85

[F] Photocatalyst optimization

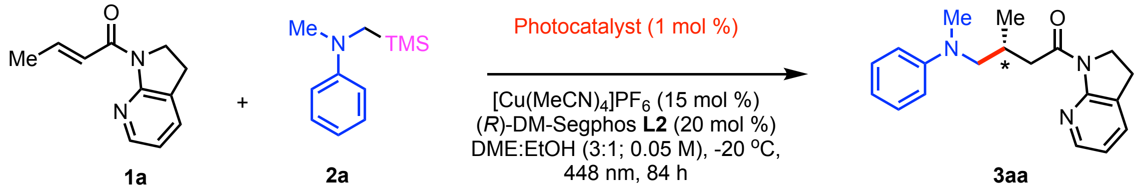

Entry	Photocatalyst	NMR Yield (%)	*ee* (%)
01	[Ru(bpy)$_3$Cl$_2$ (**PC1**)	56	88
**02**	**[Ir(ppy)$_2$dtb-bpy]PF$_6$ (PC2)**	**83**	**89**
03	[Ir{dF(CF$_3$)ppy}$_2$dtb-bpy]PF$_6$ (**PC3**)	67	88
04	*fac*-[Ir(ppy)$_3$] (**PC4**)	78	89
05	Eosin Y (**PC5**)	0	ND
06	Acridinium dye (**PC6**)	0	ND
07	None	0	ND

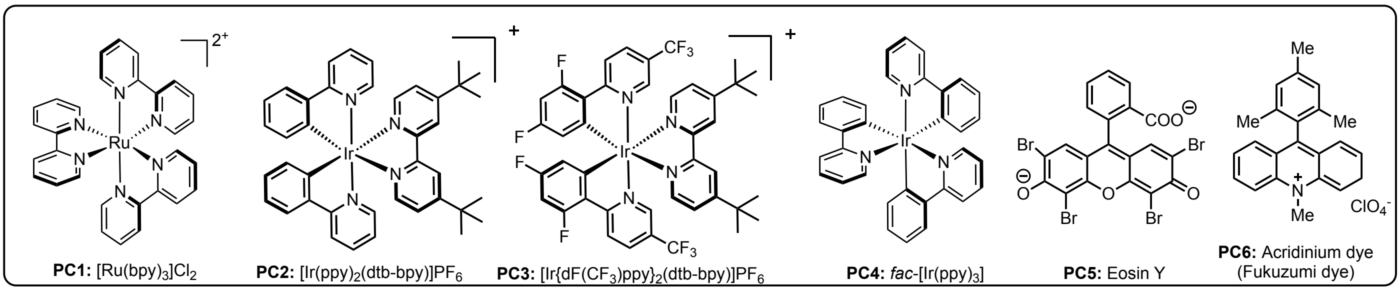

[G] Temperature optimization

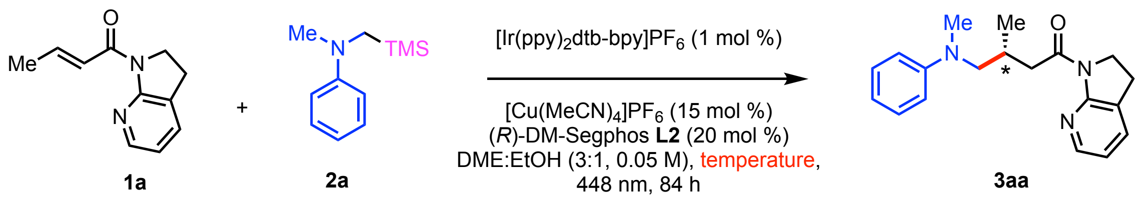

Entry	Temperature	NMR Yield (%)	*ee* (%)
01	-10	84	82
**02**	**-20**	**83**	**89**
03	-30	67	89

[H] Catalyst loading optimization

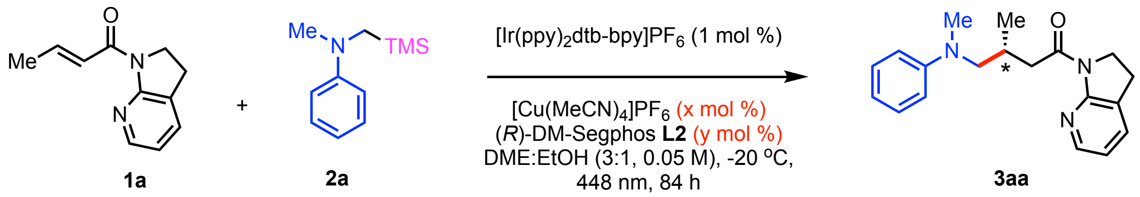

Entry	$[Cu(MeCN)_4]PF_6$ (x mol%)	(*R*)-DM-Segphos (y mol%)	NMR Yield (%)	*ee* (%)
01	5	6	66	51
02	10	12	75	82
**03**	**15**	**18**	**83**	**89**
04	20	24	83	90

5.2: Standard Procedure 1: Variation of 7-azaindolines

General procedure for asymmetric α-amino radical addition: An oven dried 10 mL Schlenk tube was charged with $[Ir(ppy)_2dtbbpy]PF_6$ (0.92 mg, 0.01 equiv., 1.0 mol %), $[Cu(MeCN)_4]PF_6$ (5.60 mg, 0.15 equiv., 15 mol %), (*R*)-DM-Segphos (13 mg, 0.18 equiv., 18 mol %) in the Glove box. To the mixture was added anhydrous DME (1.5 mL) via syringe with a stainless-steel needle at room temperature under positive Argon pressure. The resulting solution was stirred for 30 min and transferred to another Schlenk tube containing the acceptor **1** (1.0 equiv, 0.1 mmol) in EtOH (0.5 mL). The reaction was carefully degassed by 3 freeze/pump/thaw cycles under Argon in the dark. The resulting reaction mixture was then allowed to stir for additional 30 min at room temperature before cooling down to -20 °C. Then, the amine **2a** (~50 µL, 2.5 equiv., 0.25 mmol) was added slowly to the reaction mixture and allowed to equilibrate for 15 min at -20 °C. This mixture was then irradiated by blue light (λ_{max} = 448 nm) for 84 h. The crude residue loaded directly onto a Preparative Thin Layer Chromatography (PTLC) and eluted using Hexane:EtOAc (~85:15) solvent system (>2 times). If rquired, the isolated product was repurified by another PTLC with DCM. The UV-visible product band was scratched and filtered through glass frit funnel using $CHCl_3$ as an eluent to afford **3**.

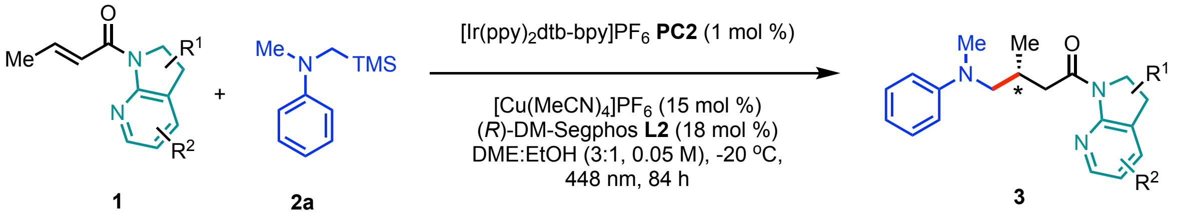

(*R*)-1-(2,3-dihydro-1*H*-pyrrolo[2,3-*b*]pyridin-1-yl)-3-methyl-4-(methyl(phenyl)amino)butan-1-one (3aa):

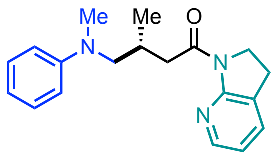

The reaction performed according to the standard procedure 1 afforded 25 mg (82%). Yellow oil.

^{1}H NMR (400 MHz, 300 K, $CDCl_3$): δ 8.06 (ddt, *J* = 5.1, 1.8, 1.0 Hz, 1H), 7.42 (dq, *J* = 7.3, 1.4 Hz, 1H), 7.20 – 7.13 (m, 2H), 6.85 (dd, *J* = 7.3, 5.1 Hz, 1H), 6.73 (dt, *J* = 8.0, 1.0 Hz, 2H), 6.62 (tt, *J* = 7.2, 1.1 Hz, 1H), 4.14 – 3.99 (m, 2H), 3.47 (dd, *J* = 14.5, 6.7 Hz, 1H), 3.43 – 3.37 (m, 1H), 3.10 (dd, *J* = 14.5, 8.3 Hz, 1H), 3.01 – 2.95 (m, 5H), 2.85 (dd, *J* = 15.0, 7.8 Hz, 1H), 2.69 – 2.58 (m, 1H), 1.04 (d, *J* = 6.7 Hz, 3H).

^{13}C NMR (101 MHz, 300 K, $CDCl_3$): δ 172.2, 156.16, 149.8, 146.2, 133.4, 129.1, 126.2, 117.9, 115.7, 112.0, 59.4, 45.8, 41.6, 39.5, 29.5, 24.3, 18.2.

IR (thin film): $\tilde{\nu}$ 2924, 1656, 1600, 1548, 1536, 1507, 1478, 1460, 1441, 1420, 1378, 1333, 1308, 1241, 1220, 1200, 1163, 1081, 1033, 991, 773, 747, 692, 565 cm^{-1}.

HRMS (ESI): *m*/*z* calculated for $C_{19}H_{24}N_3O$ $[M+H]^+$: 310.1914, found: 310.1918.

$[\alpha]_D^{26}$ -189.8 (*c* 0.41, $CHCl_3$, 89% *ee* sample).

Enantiomeric excess was determined to be 89% *ee* (*standard procedure 1, using* ***L2***) by chiral stationary phase HPLC analysis (CHIRALPAK IG3 (ϕ 0.46 cm x 25 cm), *n*-hexane/^{i}PrOH = 95/05, flow rate 1.0 mL/min, detection at 254 nm, t_R = 27.8 min (major), 34.8 min (minor).

Enantiomeric excess was determined to be 82% *ee* (*standard procedure 1, using* ***L3***) by chiral stationary phase HPLC analysis (CHIRALPAK IG3 (ϕ 0.46 cm x 25 cm), *n*-hexane/^{i}PrOH = 9/1, flow rate 1.0 mL/min, detection at 254 nm, t_R = 24.2 min (major), 29.7 min (minor).

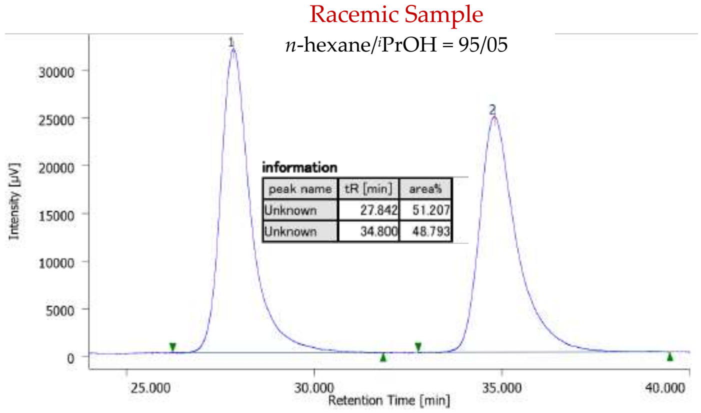

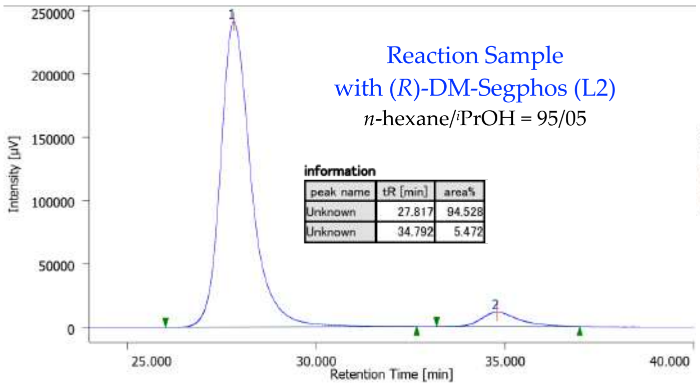

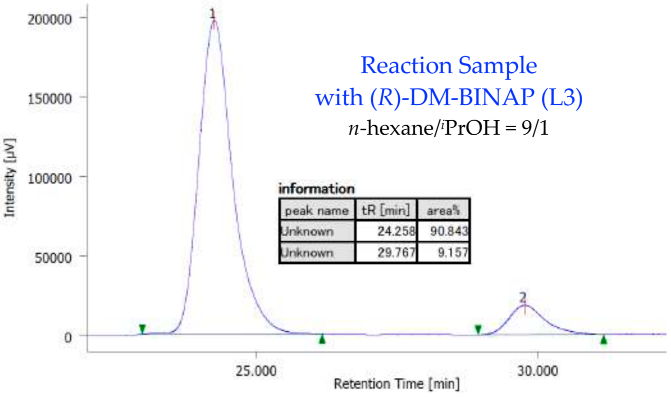

(*R*)-3-methyl-4-(methyl(phenyl)amino)-1-(6-methyl-2,3-dihydro-1*H*-pyrrolo[2,3-*b*]pyridin-1-yl)butan-1-one (3ba):

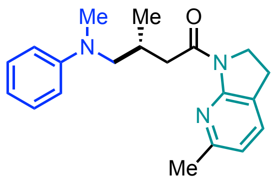

The reaction performed according to the standard procedure 1 afforded 23 mg (71%).
Yellow oil.
^{1}H NMR (400 MHz, 300 K, $CDCl_3$): δ 7.31 (dt, *J* = 7.6, 1.2 Hz, 1H), 7.20 – 7.13 (m, 2H), 6.74 – 6.69 (m, 3H), 6.65 – 6.59 (m, 1H), 4.11 – 4.02 (m, 2H), 3.46 (dd, *J* = 14.5, 6.7 Hz, 1H), 3.41 – 3.32 (m, 1H), 3.12 (dd, *J* = 14.5, 8.3 Hz, 1H), 2.97 (s, 3H), 2.96 – 2.91 (m, 3H), 2.65 – 2.53 (m, 1H), 2.45 (s, 3H), 1.05 (d, *J* = 6.7 Hz, 3H).
^{13}C NMR (101 MHz, 300 K, $CDCl_3$): δ 172.3, 155.7, 155.4, 149.9, 133.6, 129.1, 122.8, 117.0, 115.8, 112.0, 59.4, 46.0, 41.5, 39.4, 29.7, 24.3, 24.0, 18.3.
IR (thin film): $\tilde{\nu}$ 2926, 1656, 1595, 1548, 1507, 1450, 1417, 1382, 1327, 1262, 1220, 1199, 991, 772, 747, 692 cm^{-1}.
HRMS (ESI): *m*/*z* calculated for $C_{20}H_{26}N_3O$ [M+H]$^+$: 324.2070, found: 324.2073.
$[\alpha]_D^{26}$ +49.9 (*c* 1.0, $CHCl_3$, 59% *ee* sample).

Enantiomeric excess was determined to be 59% *ee* (*standard procedure 1*) by chiral stationary phase HPLC analysis (CHIRALPAK IA (ϕ 0.46 cm x 25 cm), *n*-hexane/^{i}PrOH = 95/05, flow rate 1.0 mL/min, detection at 254 nm, t_R = 9.4 min (major), 10.1 min (minor).

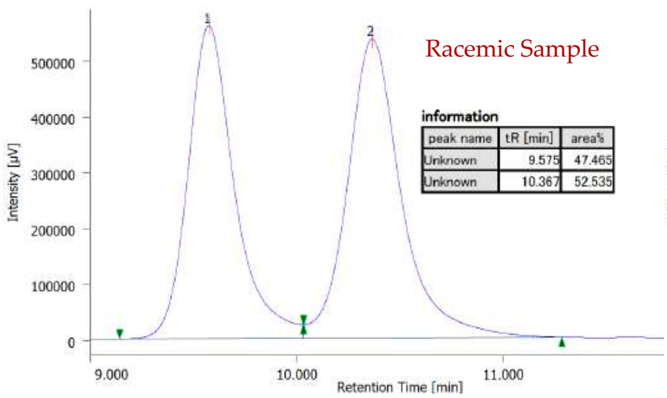

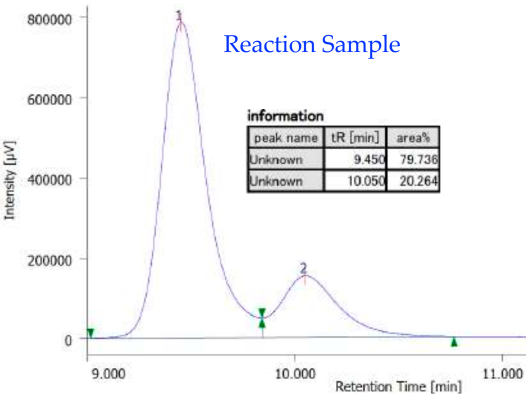

(*R*)-3-methyl-4-(methyl(phenyl)amino)-1-(6-phenyl-2,3-dihydro-1*H*-pyrrolo[2,3-*b*]pyridin-1-yl)butan-1-one (3ca):

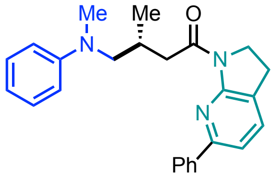

The reaction performed according to the standard procedure 1 afforded 29 mg (75%). Yellow oil.

^{1}H NMR (400 MHz, 300 K, $CDCl_3$): δ 8.02 – 7.97 (m, 2H), 7.53 – 7.44 (m, 3H), 7.43 – 7.38 (m, 1H), 7.36 (d, *J* = 7.7 Hz, 1H), 7.12 – 7.05 (m, 2H), 6.69 – 6.65 (m, 2H), 6.59 (tt, *J* = 7.1, 1.1 Hz, 1H), 4.12 (t, *J* = 8.5 Hz, 2H), 3.48 (ddd, *J* = 14.4, 11.0, 6.6 Hz, 2H), 3.22 (dd, *J* = 15.5, 7.5 Hz, 1H), 3.12 (dd, *J* = 14.5, 8.3 Hz, 1H), 3.05 – 2.99 (m, 2H), 2.92 (s, 3H), 2.76 – 2.61 (m, 1H), 1.07 (d, *J* = 6.7 Hz, 3H).

^{13}C NMR (101 MHz, 300 K, $CDCl_3$): δ 172.3, 156.1, 154.4, 149.8, 139.2, 134.1, 129.1, 129.0, 128.9, 126.7, 124.9, 115.8, 114.5, 112.15, 59.3, 46.1, 41.7, 39.4, 29.7, 24.1, 18.5.

IR (thin film): $\tilde{\nu}$ 2964, 1657, 1598, 1505, 1479, 1442, 1422, 1390, 1328, 1245, 1220, 991, 770, 747, 692 cm^{-1}.

HRMS (ESI): *m*/*z* calculated for $C_{25}H_{28}N_3O$ $[M+H]^+$: 386.2227, found: 386.2229.

$[\alpha]_D^{26}$ -42.9 (*c* 0.6, $CHCl_3$, 49% *ee* sample).

Enantiomeric excess was determined to be 49% *ee* (*standard procedure 1*) by chiral stationary phase HPLC analysis (CHIRALPAK IG3 (ϕ 0.46 cm x 25 cm), *n*-hexane/^{i}PrOH = 95/05, flow rate 1.0 mL/min, detection at 254 nm, t_R = 36.5 min (minor), 39.6 min (major).

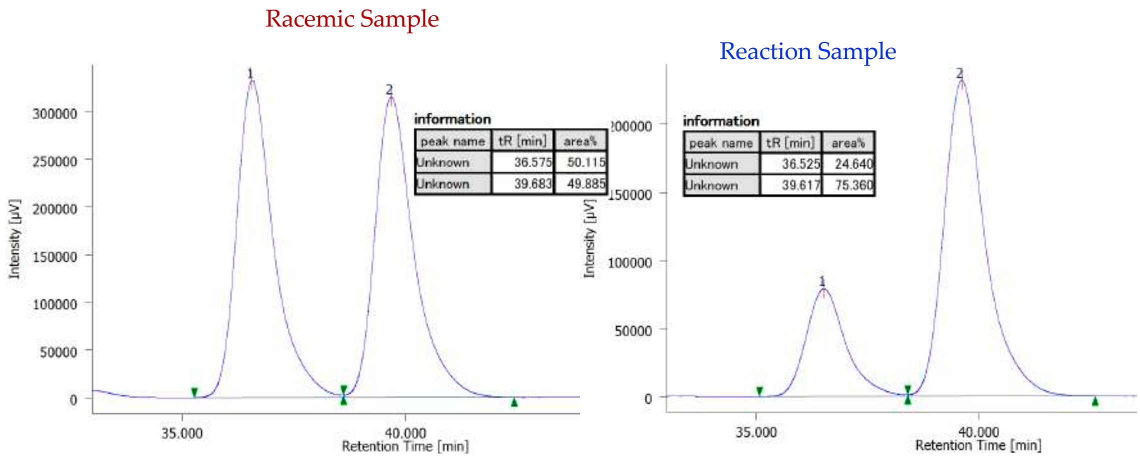

(*R*)-1-(6-methoxy-2,3-dihydro-1*H*-pyrrolo[2,3-*b*]pyridin-1-yl)-3-methyl-4-(methyl(phenyl)amino)butan-1-one (3da):

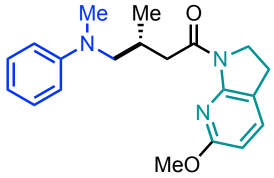

The reaction performed according to the standard procedure 1 afforded 31 mg (91%). Yellow oil.

^{1}H NMR (400 MHz, 300 K, $CDCl_3$): δ 7.35 (dt, *J* = 8.1, 1.1 Hz, 1H), 7.21 – 7.13 (m, 2H), 6.76 – 6.68 (m, 2H), 6.63 (tt, *J* = 7.2, 1.1 Hz, 1H), 6.33 (d, *J* = 8.1 Hz, 1H), 4.13 – 4.04 (m, 2H), 3.88 (s, 3H), 3.45 (dd, *J* = 14.4, 6.7 Hz, 1H), 3.30 – 3.05 (m, 3H), 2.96 (s, 3H), 2.94 – 2.87 (m, 2H), 2.73 – 2.61 (m, 1H), 1.05 (d, *J* = 6.7 Hz, 3H).

^{13}C NMR (101 MHz, 300 K, $CDCl_3$): δ 171.6, 163.0, 153.8, 149.9, 136.1, 129.1, 117.0, 115.9, 112.1, 103.4, 59.1, 53.8, 46.7, 41.1, 39.3, 29.5, 23.7, 18.6.

IR (thin film): $\tilde{\nu}$ 2928, 1656, 1598, 1548, 1507, 1473, 1418, 1392, 1293, 1252, 1220, 1196, 1159, 1092, 1220, 1196, 1159, 1092, 1026, 991, 808, 772, 748, 692, 669, 534 cm^{-1}.

HRMS (ESI): *m*/*z* calculated for $C_{20}H_{26}N_3O_2$ $[M+H]^+$: 340.2020, found: 340.2024.

$[\alpha]_D^{26}$ +53.9 (*c* 0.86, $CHCl_3$, 98% *ee* sample).

Enantiomeric excess was determined to be 98% *ee* (*standard procedure 1, using* ***L2***) by chiral stationary phase HPLC analysis (CHIRALPAK IG3 (ϕ 0.46 cm x 25 cm), *n*-hexane/^{i}PrOH = 95/05, flow rate 1.0 mL/min, detection at 254 nm, t_R = 24.1 min (major), 29.1 min (minor).

Enantiomeric excess was determined to be 90% *ee* (*standard procedure 1, using* ***L3***) by chiral stationary phase HPLC analysis (CHIRALPAK IG3 (ϕ 0.46 cm x 25 cm), *n*-hexane/^{i}PrOH = 9/1, flow rate 1.0 mL/min, detection at 254 nm, t_R = 21.4 min (major), 24.4 min (minor).

Racemic Sample

n-hexane/^{i}PrOH = 95/05

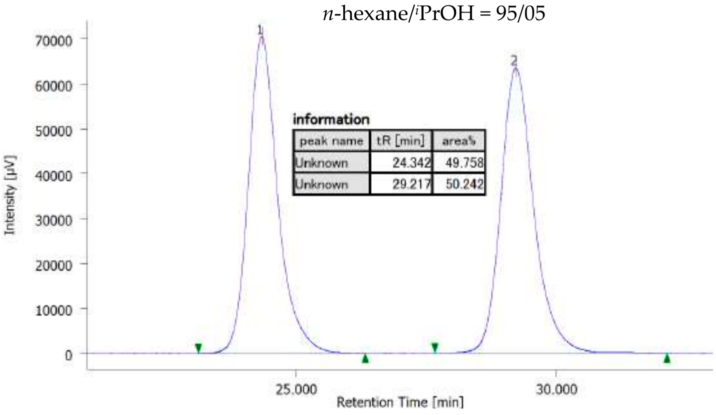

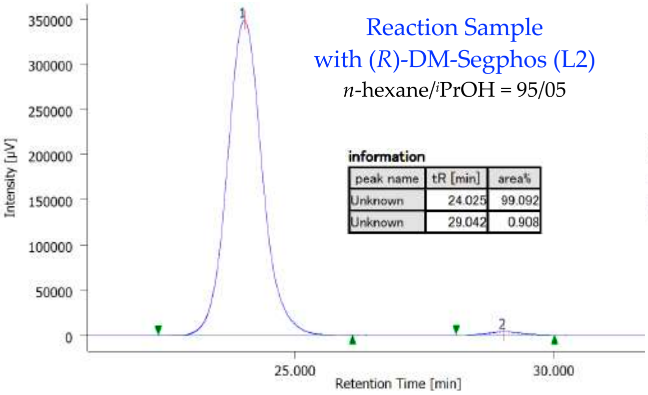

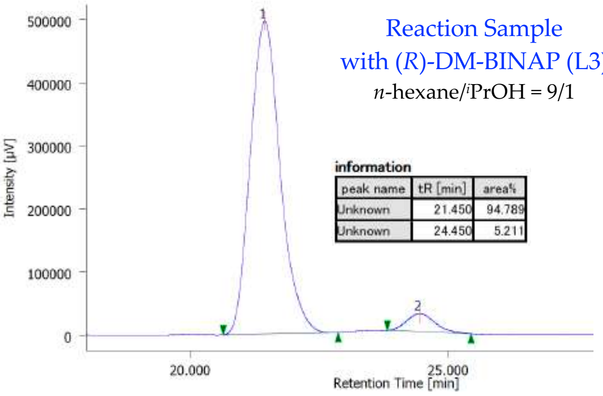

(*R*)-1-(6-chloro-2,3-dihydro-1*H*-pyrrolo[2,3-*b*]pyridin-1-yl)-3-methyl-4-(methyl(phenyl)amino)butan-1-one (3ea):

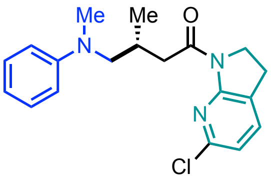

The reaction performed according to the standard procedure 1 afforded 30 mg (87%). Yellow oil.

^{1}H NMR (400 MHz, 300 K, $CDCl_3$): δ 7.93 (dt, *J* = 5.5, 1.0 Hz, 1H), 7.18 – 7.11 (m, 2H), 6.85 (d, *J* = 5.5 Hz, 1H), 6.71 (dt, *J* = 7.8, 1.0 Hz, 2H), 6.65 – 6.59 (m, 1H), 4.13 – 4.00 (m, 2H), 3.46 – 3.36 (m, 2H), 3.10 (dd, *J* = 14.5, 7.9 Hz, 1H), 3.01 – 2.97 (m, 2H), 2.96 (s, 3H), 2.80 (dd, *J* = 15.1, 7.7 Hz, 1H), 2.69 – 2.55 (m, 1H), 1.04 (d, *J* = 6.7 Hz, 3H).

^{13}C NMR (101 MHz, 300 K, $CDCl_3$): δ 172.1, 155.8, 149.7, 148.0, 135.4, 129.1, 124.6, 117.4, 115.8, 112.1, 59.3, 46.4, 41.5, 39.5, 29.6, 23.7, 18.2.

IR (thin film): $\tilde{\nu}$ 2962, 2927, 1664, 1600, 1578, 1507, 1480, 1442, 1423, 1377, 1327, 1253, 1220, 1195, 1114, 1081, 1032, 992, 869, 804, 749, 693 cm^{-1}.

HRMS (ESI): *m*/*z* calculated for $C_{19}H_{23}N_3OCl$ [M+H]$^+$: 344.1524, found: 344.1528.

$[\alpha]_D^{26}$ +136.5 (*c* 1.0, $CHCl_3$, 95% *ee* sample).

Enantiomeric excess was determined to be 95% *ee* (*standard procedure 1*) by chiral stationary phase HPLC analysis (CHIRALPAK IG3 (ϕ 0.46 cm x 25 cm), *n*-hexane/^{i}PrOH = 95/05, flow rate 1.0 mL/min, detection at 254 nm, t_R = 26.3 min (major), 34.4 min (minor).

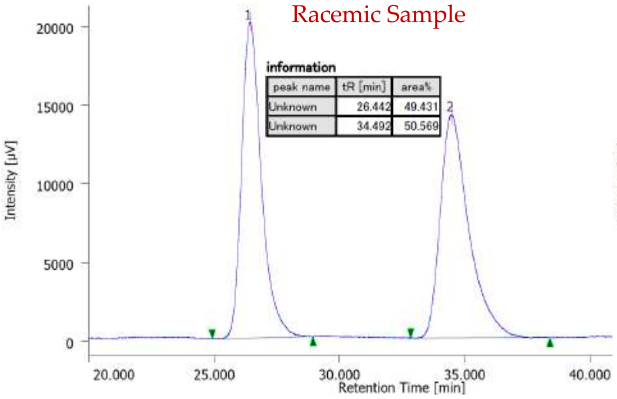

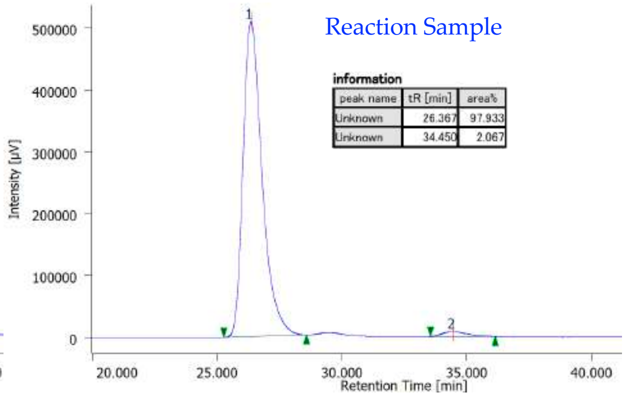

(*R*)-1-(3-methyl-4-(methyl(phenyl)amino)butanoyl)-2,3-dihydro-1*H*-pyrrolo[2,3-*b*]pyridine-6-carbonitrile (3fa):

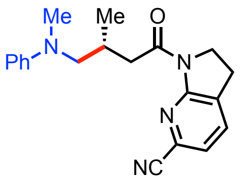

The reaction performed according to the standard procedure 1 afforded 19 mg (56%). Yellow oil.

^{1}H NMR (400 MHz, 300 K, CDCl$_3$): δ 7.51 – 7.42 (m, 1H), 7.28 – 7.24 (m, 1H), 7.19 – 7.13 (m, 2H), 6.75 – 6.67 (m, 2H), 6.60 (tt, *J* = 7.2, 1.1 Hz, 1H), 4.06 (td, *J* = 8.7, 1.5 Hz, 2H), 3.47 – 3.36 (m, 1H), 3.28 (dd, *J* = 15.9, 6.3 Hz, 1H), 3.13 (dd, *J* = 14.5, 7.3 Hz, 1H), 3.04 – 2.98 (m, 2H), 2.96 (s, 3H), 2.89 (dd, *J* = 15.9, 7.3 Hz, 1H), 2.63 (dq, *J* = 13.9, 7.0 Hz, 1H), 1.07 (d, *J* = 6.7 Hz, 3H).

^{13}C NMR (101 MHz, 300 K, CDCl$_3$): δ 172.3, 156.8, 149.7, 133.6, 131.8, 129.5, 129.1, 123.3, 117.7, 115.9, 112.1, 59.2, 45.9, 41.8, 39.4, 29.5, 24.4, 18.4.

IR (thin film): $\tilde{\nu}$ 2959, 2870, 2231, 1654, 1596, 1576, 1505, 1479, 1437, 1418, 1373, 1317, 1295, 1263, 1218, 1190, 1082, 990, 833, 772, 746, 691, 670, 654, 506, 492 cm^{-1}.

HRMS (ESI): *m/z* calculated for $C_{20}H_{23}N_4O$ [M+H]$^+$: 335.1866, found: 335.1871.

$[\alpha]_D^{26}$ +16.3 (*c* 0.7, CHCl$_3$, 37% *ee* sample).

Enantiomeric excess was determined to be 37% *ee* (*standard procedure 1*) by chiral stationary phase HPLC analysis (CHIRALPAK IG3 (ϕ 0.46 cm x 25 cm), *n*-hexane/^{i}PrOH = 95/05, flow rate 1.0 mL/min, detection at 254 nm, t_R = 108.3 min (major), 149.0 min (minor).

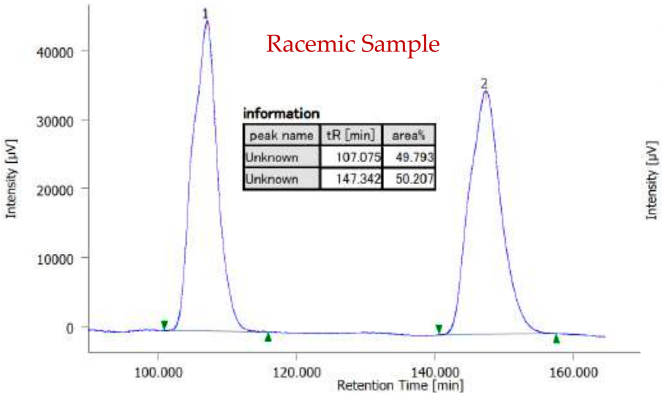

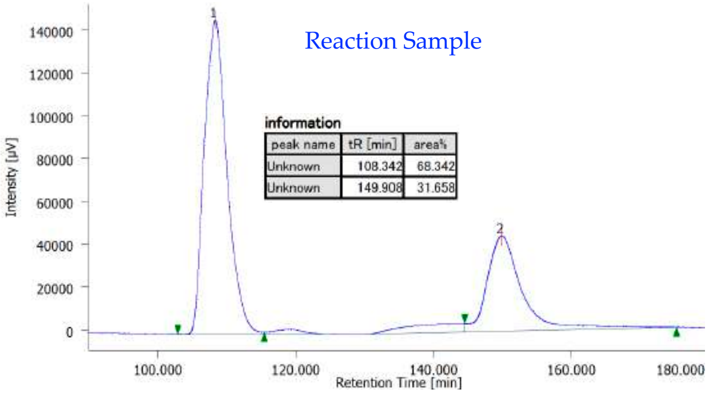

(*R*)-1-(4-methoxy-2,3-dihydro-1*H*-pyrrolo[2,3-*b*]pyridin-1-yl)-3-methyl-4-(methyl(phenyl)amino)butan-1-one (3ga):

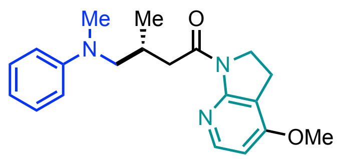

The reaction performed according to the standard procedure 1 afforded 22 mg (65%). Colorless sticky oil.

^{1}H NMR (400 MHz, 300 K, CDCl$_3$): δ 8.00 (dt, *J* = 5.9, 1.0 Hz, 1H), 7.20 – 7.13 (m, 2H), 6.76 – 6.71 (m, 2H), 6.64 – 6.59 (m, 1H), 6.47 (d, *J* = 5.9 Hz, 1H), 4.14 – 3.99 (m, 2H), 3.87 (s, 3H), 3.46 (dd, *J* = 14.5, 6.5 Hz, 1H), 3.43 – 3.34 (m, 1H), 3.08 (dd, *J* = 14.5, 8.4 Hz, 1H), 2.97 (s, 3H), 2.93 – 2.78 (m, 3H), 2.61 (dq, *J* = 14.4, 7.3 Hz, 1H), 1.03 (d, *J* = 6.7 Hz, 3H).

^{13}C NMR (101 MHz, 300 K, CDCl$_3$): δ 172.2, 162.7, 157.6, 149.8, 148.6, 129.1, 115.6, 112.3, 112.0, 102.4, 59.4, 55.6, 46.2, 41.4, 39.5, 29.6, 21.4, 18.2.

IR (thin film): $\tilde{\nu}$ 2922, 2358, 1656, 1588, 1508, 1417, 1376, 1288, 1200, 1114, 1014, 798, 747, 692, 658, 644, 618, 567, 551, 538 cm^{-1}.

HRMS (ESI): *m/z* calculated for $C_{20}H_{26}N_3O_2$ [M+H]$^+$: 340.2020, found: 340.2020.

$[\alpha]_D^{26}$ -50.1 (*c* 0.1, CHCl$_3$, 86% *ee* sample).

Enantiomeric excess was determined to be 86% *ee* (*standard procedure 1*) by chiral stationary phase HPLC analysis (CHIRALPAK IG3 (ϕ 0.46 cm x 25 cm), *n*-hexane/ethanol = 95/05, flow rate 1.0 mL/min, detection at 254 nm, t_R = 37.0 min (major), 42.8 min (minor).

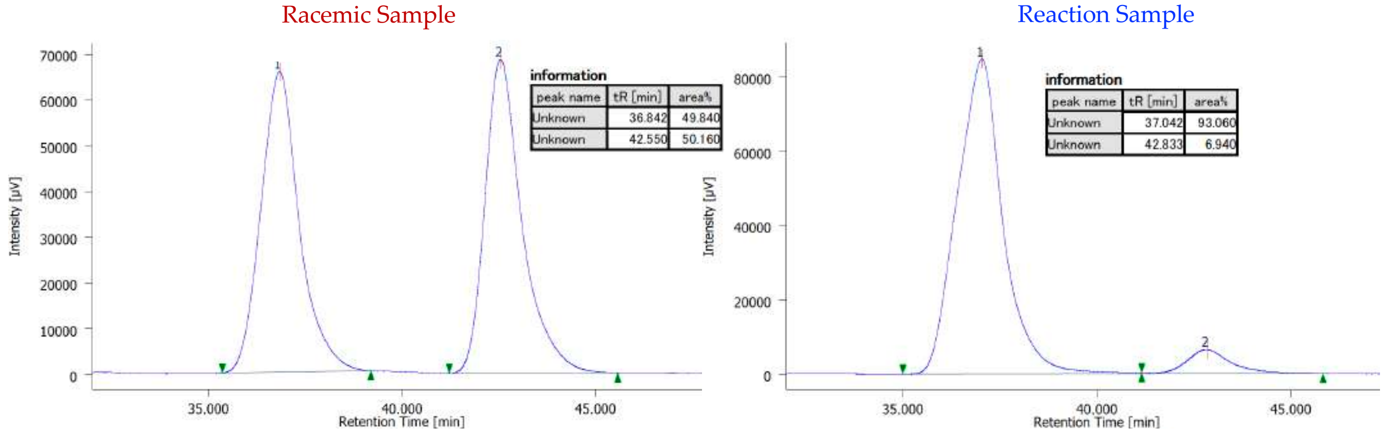

(*R*)-1-(4-chloro-2,3-dihydro-1*H*-pyrrolo[2,3-*b*]pyridin-1-yl)-3-methyl-4-(methyl(phenyl)amino)butan-1-one (3ha):

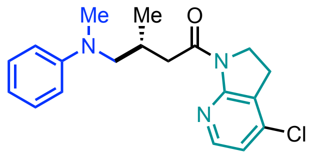

The reaction performed according to the standard procedure 1 afforded 23 mg (65%). Yellow oil.

^{1}H NMR (400 MHz, 300 K, CDCl$_3$): δ 7.93 (dt, *J* = 5.5, 1.0 Hz, 1H), 7.18 – 7.12 (m, 2H), 6.85 (d, *J* = 5.5 Hz, 1H), 6.71 (dt, *J* = 7.8, 1.0 Hz, 2H), 6.65 – 6.59 (m, 1H), 4.13 – 4.00 (m, 2H), 3.41 (ddd, *J* = 15.1, 10.9, 6.5 Hz, 2H), 3.10 (dd, *J* = 14.5, 7.9 Hz, 1H), 3.02 – 2.97 (m, 2H), 2.96 (s, 3H), 2.80 (dd, *J* = 15.1, 7.7 Hz, 1H), 2.71 – 2.55 (m, 1H), 1.04 (d, *J* = 6.7 Hz, 3H).

^{13}C NMR (101 MHz, 300 K, CDCl$_3$): δ 172.3, 157.0, 149.7, 147.2, 140.7, 129.1, 125.0, 118.3, 115.8, 112.0, 59.3, 45.5, 41.5, 39.5, 29.5, 23.5, 18.2.

IR (thin film): $\tilde{\nu}$ 2956, 2916, 1662, 1598, 1571, 1507, 1440, 1410, 1375, 1357, 1330, 1244, 1200, 1161, 991, 815, 747, 692, 644, 593 cm^{-1}.

HRMS (ESI): *m/z* calculated for $C_{19}H_{23}N_3OCl$ [M+H]$^+$: 344.1524, found: 344.1527.

$[\alpha]_D^{26}$ +51.2 (*c* 1.0, CHCl$_3$, 83% *ee* sample).

Enantiomeric excess was determined to be 83% *ee* (*standard procedure 1*) by chiral stationary phase HPLC analysis (CHIRALPAK IG3 (ϕ 0.46 cm x 25 cm), *n*-hexane/^{i}PrOH = 95/05, flow rate 1.0 mL/min, detection at 254 nm, t_R = 16.9 min (major), 20.2 min (minor).

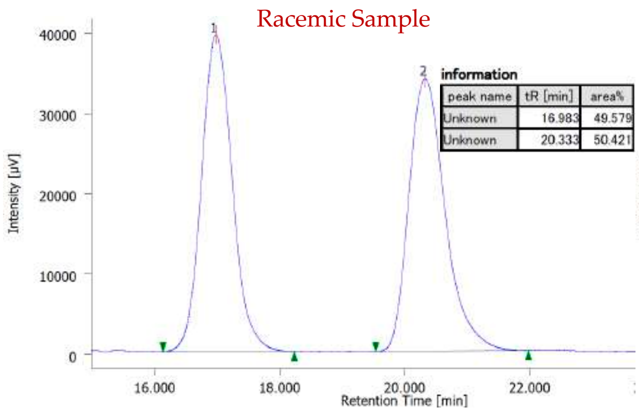

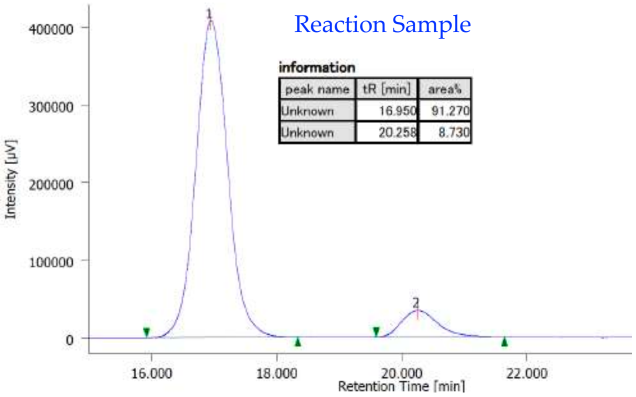

(*R*)-1-(3,3-dimethyl-2,3-dihydro-1*H*-pyrrolo[2,3-*b*]pyridin-1-yl)-3-methyl-4-(methyl(phenyl)amino)butan-1-one (3ia):

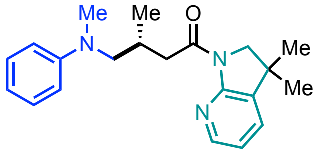

The reaction performed according to the standard procedure 1 afforded 24 mg (71%). Yellow oil.

^{1}H NMR (400 MHz, 300 K, CDCl$_3$): δ 8.08 (dd, *J* = 5.1, 1.7 Hz, 1H), 7.39 (dd, *J* = 7.3, 1.7 Hz, 1H), 7.22 – 7.15 (m, 2H), 6.89 (dd, *J* = 7.4, 5.1 Hz, 1H), 6.75 (dt, *J* = 7.8, 1.0 Hz, 2H), 6.63 (tt, *J* = 7.2, 1.0 Hz, 1H), 3.88 – 3.78 (m, 2H), 3.48 (dd, *J* = 14.5, 6.4 Hz, 1H), 3.40 (dd, *J* = 14.9, 5.8 Hz, 1H), 3.10 (dd, *J* = 14.5, 8.5 Hz, 1H), 2.98 (s, 3H), 2.90 (dd, *J* = 15.0, 8.0 Hz, 1H), 2.71 – 2.57 (m, 1H), 1.31 (s, 3H), 1.29 (s, 3H), 1.05 (d, *J* = 6.7 Hz, 3H).

^{13}C NMR (101 MHz, 300 K, CDCl$_3$): δ 172.3, 154.7, 149.8, 146.4, 135.3, 130.9, 129.1, 118.3, 115.8, 112.1, 60.3, 59.5, 41.6, 39.6, 36.5, 29.6, 28.7, 28.6, 18.2.

IR (thin film): $\tilde{\nu}$ 2957, 2920, 2869, 1660, 1599, 1507, 1466, 1420, 1375, 1335, 1308, 1260, 1201, 992, 796, 747, 692, 644, 617, 566, 540 cm^{-1}.

HRMS (ESI): *m/z* calculated for $C_{21}H_{28}N_3O$ [M+H]$^+$: 338.2227, found: 338.2225.

$[\alpha]_D^{26}$ +18.9 (*c* 0.34, CHCl$_3$, 86% *ee* sample).

Enantiomeric excess was determined to be 86% *ee* (*standard procedure 1*) by chiral stationary phase HPLC analysis (CHIRALPAK OJ-3 (ϕ 0.46 cm x 25 cm), *n*-hexane/^{i}PrOH = 95/05, flow rate 1.0 mL/min, detection at 254 nm, t_R = 12.5 min (major), 15.3 min (minor).

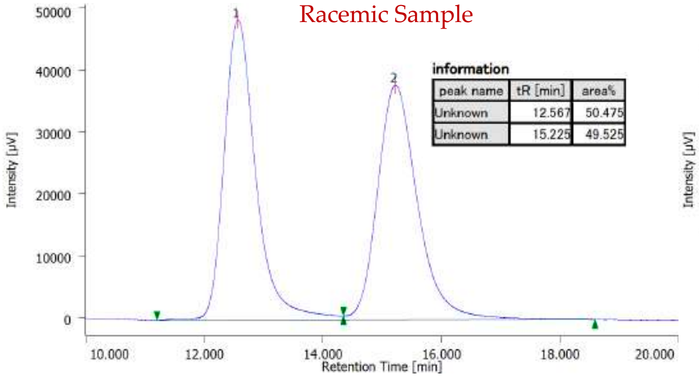

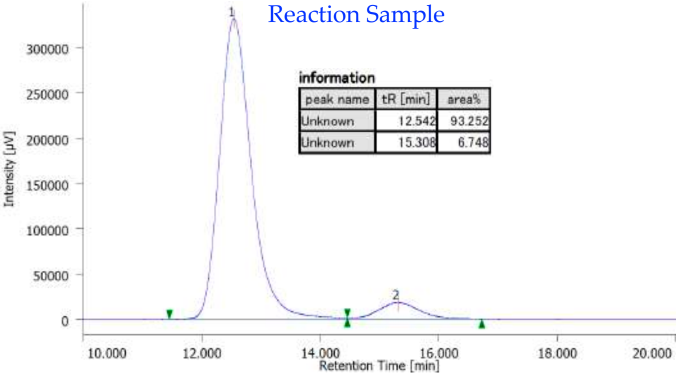

5.3: Standard Procedure 2: Variation at the α,β,-positions

General procedure for asymmetric α-amino radical addition: An oven dried 10 mL Schlenk tube was charged with [Ir(ppy)$_2$dtbbpy]PF$_6$ (0.46 mg, 0.005 equiv., 0.5 mol %), [Cu(MeCN)$_4$]PF$_6$ (4.48 mg, 0.12 equiv., 12 mol %), (*R*)-DM-Segphos (10.8 mg, 0.15 equiv., 15 mol %) in the Glove box. To the mixture was added anhydrous DME (1.5 mL) via syringe with a stainless-steel needle at room temperature under positive Argon pressure. The resulting solution was stirred for 30 min and transferred to another Schlenk tube containing the acceptor **1** (1.0 equiv, 0.1 mmol) in EtOH (0.5 mL). The reaction was carefully degassed by 3 freeze/pump/thaw cycles under Argon in the dark. The resulting reaction mixture was then allowed to stir for additional 30 min at room temperature before cooling down to -20 °C. Then, the amine **2a** (~25 µL, 1.2 equiv., 0.12 mmol) was added slowly to the reaction mixture and allowed to equilibrate for 15 min at -20 °C. This mixture was then irradiated by blue light (λ_{max} = 448 nm) for 48 h. The crude residue loaded directly onto a Preparative Thin Layer Chromatography (PTLC) and eluted using Hexane:EtOAc (~85:15) solvent system (>2 times). If rquired, the isolated product was repurified by another PTLC with DCM. The UV-visible product band was scratched and filtered through glass frit funnel using CHCl$_3$ as an eluent to afford **3**.

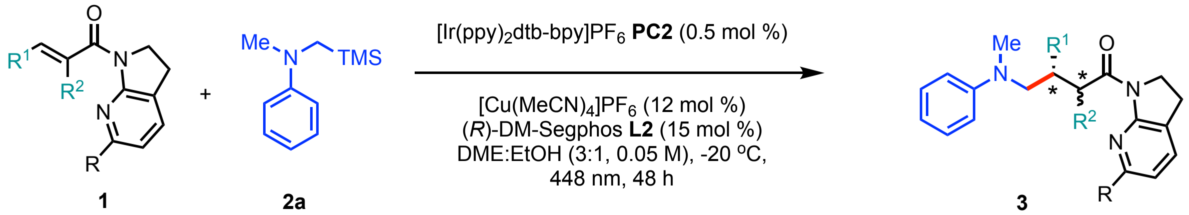

(*R*)-1-(2,3-dihydro-1*H*-pyrrolo[2,3-*b*]pyridin-1-yl)-4-(methyl(phenyl)amino)-3-phenylbutan-1-one (3ja):

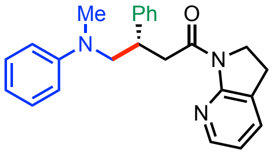

The reaction performed according to the standard procedure 2 afforded 27 mg (72%).
Yellow oil.
^{1}H NMR (400 MHz, 300 K, CDCl$_3$): δ 8.10 – 8.07 (m, 1H), 7.41 – 7.37 (m, 1H), 7.29 – 7.22 (m, 4H), 7.21 – 7.12 (m, 3H), 6.84 (dd, *J* = 7.3, 5.1 Hz, 1H), 6.72 – 6.68 (m, 2H), 6.61 (tt, *J* = 7.2, 1.0 Hz, 1H), 4.00 – 3.94 (m, 2H), 3.86 – 3.77 (m, 2H), 3.72 – 3.49 (m, 2H), 3.38 – 3.29 (m, 1H), 2.96 – 2.87 (m, 2H), 2.64 (s, 3H).
^{13}C NMR (101 MHz, 300 K, CDCl$_3$): δ 171.4, 156.1, 149.2, 146.2, 143.3, 133.4, 129.1, 128.5, 128.3, 126.6, 126.2, 118.0, 115.8, 112.0, 59.6, 45.8, 40.5, 40.0, 39.3, 24.3.
IR (thin film): $\tilde{\nu}$ 2925, 1657, 1599, 1548, 1529, 1504, 1480, 1441, 1421, 1391, 1346, 1305, 1241, 1218, 772, 748, 698 cm^{-1}.
HRMS (ESI): *m/z* calculated for $C_{24}H_{26}N_3O$ [M+H]$^+$: 372.2070, found: 372.2073.
$[\alpha]_D^{26}$ -56.4 (*c* 0.43, CHCl$_3$, 84% *ee* sample).

Enantiomeric excess was determined to be 84% *ee* (*standard procedure 2, using* ***L2***) by chiral stationary phase HPLC analysis (CHIRALPAK IA (*ϕ* 0.46 cm x 25 cm), *n*-hexane/^{i}PrOH = 9/1, flow rate 1.0 mL/min, detection at 254 nm, t_R = 10.1 min (major), 15.6 min (minor).

Enantiomeric excess was determined to be 78% *ee* (*standard procedure 2, using* ***L3***) by chiral stationary phase HPLC analysis (CHIRALPAK IA (*ϕ* 0.46 cm x 25 cm), *n*-hexane/^{i}PrOH = 9/1, flow rate 1.0 mL/min, detection at 254 nm, t_R = 10.1 min (major), 15.6 min (minor).

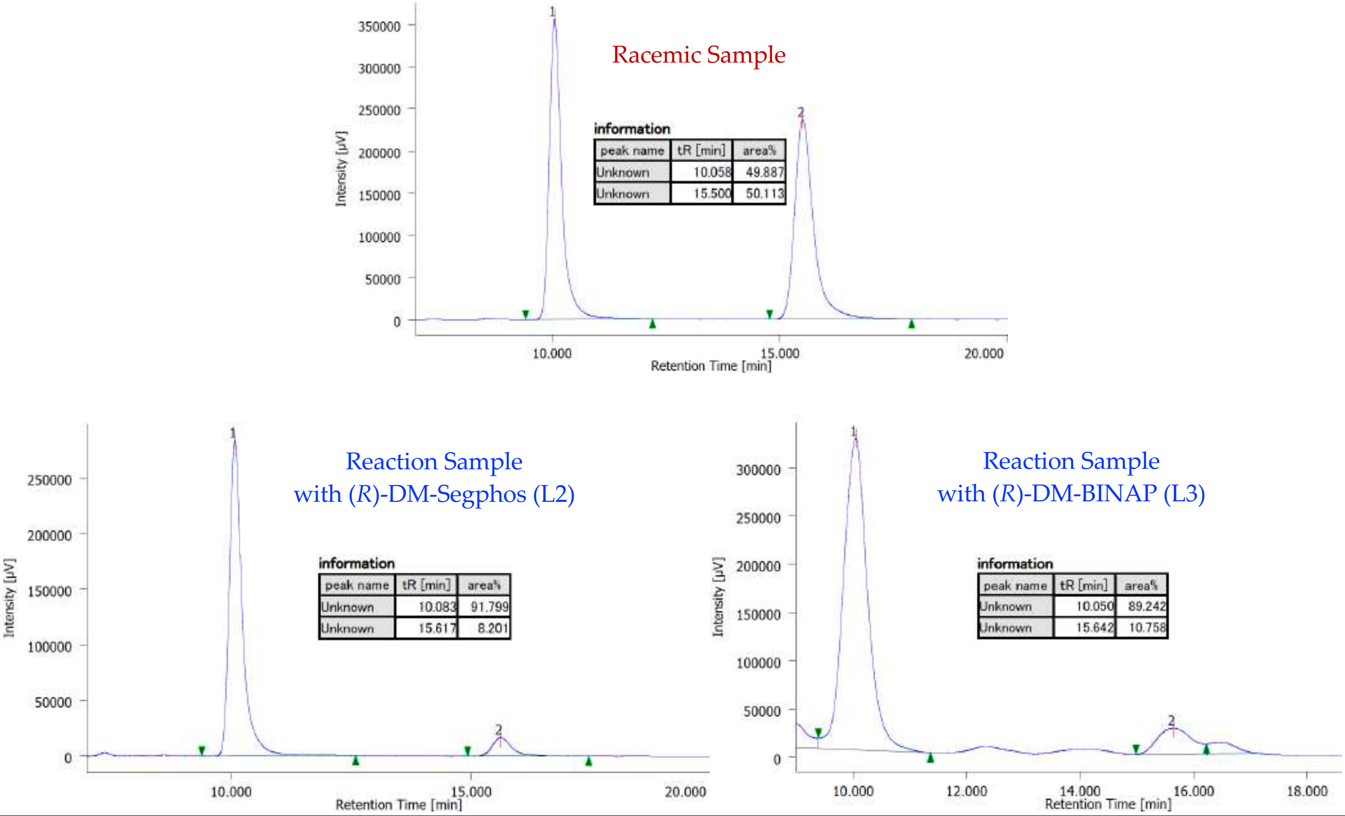

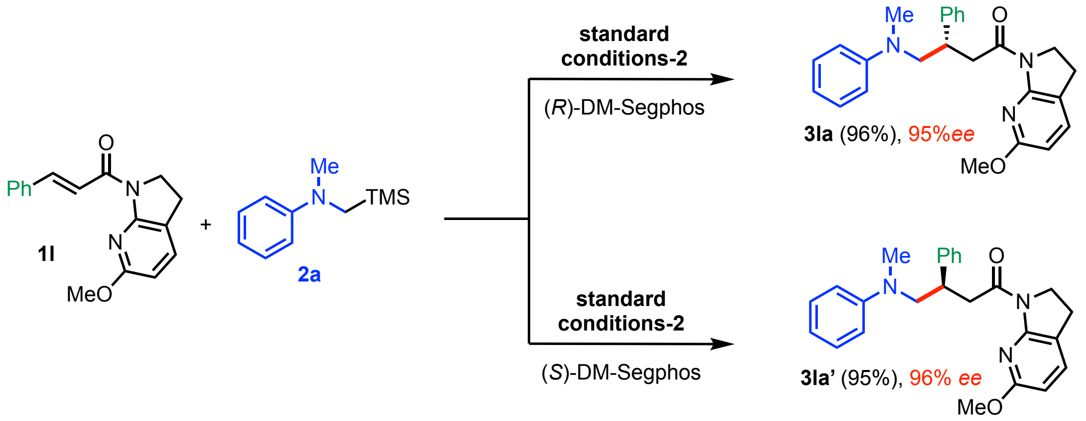

(*R*)-1-(6-methoxy-2,3-dihydro-1*H*-pyrrolo[2,3-*b*]pyridin-1-yl)-4-(methyl(phenyl)amino)-3-phenylbutan-1-one (3la):

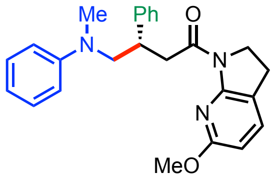

The reaction performed according to the standard procedure 2 afforded 38.5 mg (96%) from **1l** [*E*-isomer/(*R*)-DM-Segphos].

Yellow oil.

^{1}H NMR (400 MHz, 300 K, CDCl$_3$): δ 7.45 – 7.39 (m, 1H), 7.34 (dt, *J* = 8.1, 1.1 Hz, 1H), 7.31 – 7.22 (m, 4H), 7.22 – 7.14 (m, 2H), 6.71 (dt, *J* = 7.8, 1.0 Hz, 2H), 6.63 (tt, *J* = 7.2, 1.0 Hz, 1H), 6.33 (d, *J* = 8.1 Hz, 1H), 4.05 (td, *J* = 8.8, 1.8 Hz, 2H), 3.90 (dd, *J* = 14.2, 6.5 Hz, 1H), 3.84 (s, 3H), 3.83 – 3.77 (m, 1H), 3.74 – 3.54 (m, 2H), 3.28 (dd, *J* = 14.1, 8.1 Hz, 1H), 2.92 – 2.85 (m, 2H), 2.63 (s, 3H).

^{13}C NMR (101 MHz, 300 K, CDCl$_3$): δ 170.9, 163.0, 153.8, 149.3, 143.8, 136.2, 129.8, 129.2, 128.5, 128.1, 126.7, 122.5, 117.0, 115.9, 112.0, 103.6, 59.6, 53.8, 46.7, 40.1, 39.8, 39.3, 23.7.

IR (thin film): $\tilde{\nu}$ 2986, 2906, 1654, 1595, 1507, 1473, 1449, 1420, 1347, 1293, 1252, 1196, 1159, 1116, 1092, 1025, 992, 904, 865, 809, 749, 698, 670, 644 cm^{-1}.

HRMS (ESI): *m*/*z* calculated for $C_{25}H_{28}N_3O_2$ [M+H]$^+$: 402.2176, found: 402.2178.

$[\alpha]_D^{26}$ -91.6 (*c* 1.0, CHCl$_3$, 95% *ee* sample).

Enantiomeric excess was determined to be 95% *ee* (*standard procedure 2, using* ***L2***) by chiral stationary phase HPLC analysis (CHIRALPAK IE (ϕ 0.46 cm x 25 cm), *n*-hexane/^{i}PrOH = 95/05, flow rate 1.0 mL/min, detection at 254 nm, t_R = 32.5 min (major), 36.6 min (minor).

Enantiomeric excess was determined to be 84% *ee* (*standard procedure 2, using* ***L3***) by chiral stationary phase HPLC analysis (CHIRALPAK IE (ϕ 0.46 cm x 25 cm), *n*-hexane/^{i}PrOH = 95/05, flow rate 1.0 mL/min, detection at 254 nm, t_R = 33.4 min (major), 36.1 min (minor).

(*S*)-1-(6-methoxy-2,3-dihydro-1*H*-pyrrolo[2,3-*b*]pyridin-1-yl)-4-(methyl(phenyl)amino)-3-phenylbutan-1-one (3la'):

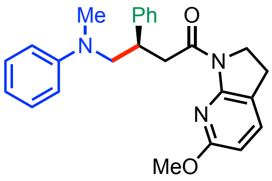

The reaction performed according to the standard procedure 2 afforded 38 mg (95%) from **1l** [*E*-isomer/(*S*)-DM-Segphos].

Yellow oil.

^{1}H NMR (400 MHz, 300 K, CDCl$_3$): δ 7.46 – 7.40 (m, 1H), 7.35 (dt, *J* = 8.1, 1.0 Hz, 1H), 7.32 – 7.24 (m, 3H), 7.23 – 7.16 (m, 3H), 6.73 (dt, *J* = 7.8, 1.0 Hz, 2H), 6.65 (tt, *J* = 7.2, 1.1 Hz, 1H), 6.35 (d, *J* = 8.1 Hz, 1H), 4.11 – 4.02 (m, 2H), 3.91 (dd, *J* = 14.2, 6.5 Hz, 1H), 3.85 (s, 3H), 3.84 – 3.79 (m, 1H), 3.76 – 3.56 (m, 2H), 3.30 (dd, *J* = 14.2, 8.1 Hz, 1H), 2.90 (ddd, *J* = 9.8, 7.0, 1.1 Hz, 2H), 2.64 (s, 3H).

^{13}C NMR (101 MHz, 300 K, CDCl$_3$): δ 170.9, 163.0, 153.8, 149.3, 143.8, 136.2, 129.8, 129.2, 128.5, 128.0, 126.7, 122.5, 117.0, 115.9, 112.0, 103.6, 59.6, 53.8, 46.7, 40.1, 39.8, 39.3, 23.7.

IR (thin film): $\tilde{\nu}$ 3025, 2905, 1654, 1596, 1507, 1473, 1449, 1420, 1400, 1347, 1293, 1252, 1193, 1159, 1116, 1092, 1024, 992, 948, 904, 866, 809, 785, 748, 699, 670, 644 cm^{-1}.

HRMS (ESI): *m*/*z* calculated for $C_{25}H_{28}N_3O_2$ [M+H]$^+$: 402.2176, found: 402.2178.

$[\alpha]_D^{26}$ +92.5 (*c* 1.0, CHCl$_3$, 96% *ee* sample).

Enantiomeric excess was determined to be 96% *ee* (*standard procedure 2, using* ***L2'***) by chiral stationary phase HPLC analysis (CHIRALPAK IE (ϕ 0.46 cm x 25 cm), *n*-hexane/^{i}PrOH = 95/05, flow rate 1.0 mL/min, detection at 254 nm, t_R = 34.7 min (minor), 37.0 min (major).

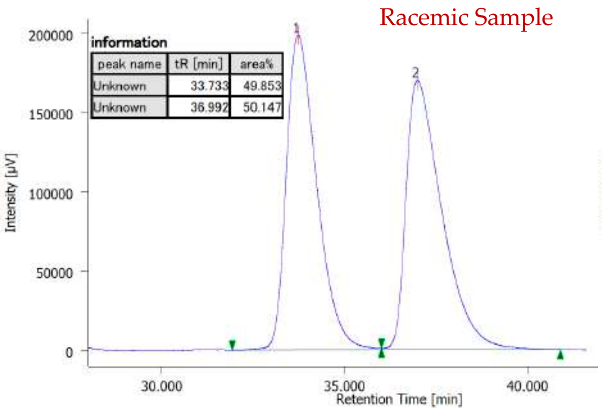

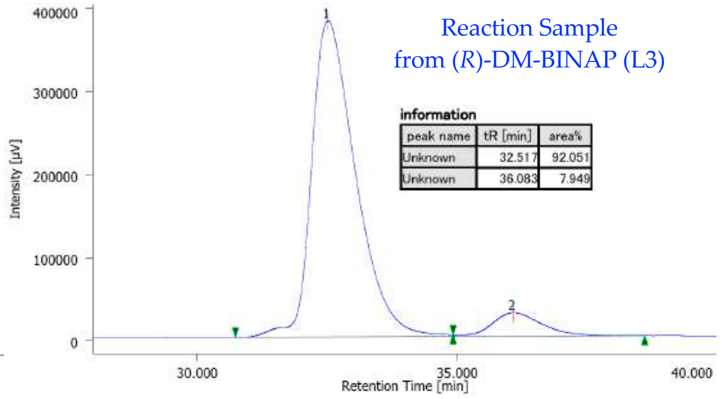

Reaction Sample
from (*R*)-DM-Segphos (L2)

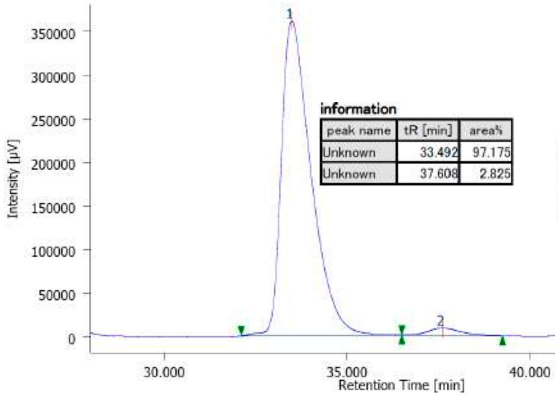

Reaction Sample
from (*S*)-DM-Segphos (L2')

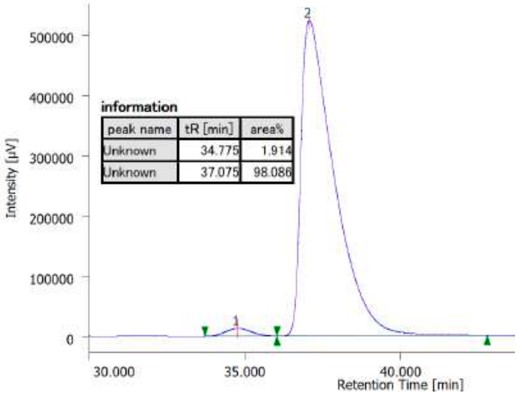

(*R*)-1-(2,3-dihydro-1*H*-pyrrolo[2,3-*b*]pyridin-1-yl)-4-(methyl(phenyl)amino)-3-(pyridin-2-yl)butan-1-one (3ka):
The reaction performed according to the standard procedure 2 afforded 26 mg crude product (71%).

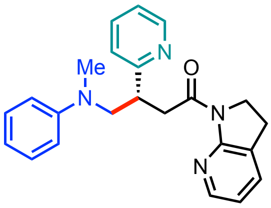

Yellow oil. (Note: *Very unstable compound, diificult to handle*).

^{1}H NMR (500 MHz, 300 K, CDCl$_3$): δ 8.58 (d, *J* = 4.9 Hz, 1H), 8.11 (d, *J* = 5.2 Hz, 1H), 7.64 (s, 1H), 7.46 – 7.38 (m, 1H), 7.17 (dd, *J* = 8.8, 7.2 Hz, 1H), 7.15 – 7.06 (m, 2H), 6.87 – 6.83 (m, 1H), 6.74 – 6.69 (m, 1H), 6.63 (t, *J* = 7.2 Hz, 2H), 6.45 – 6.40 (m, 1H), 4.01 (t, *J* = 8.5 Hz, 2H), 3.93 (d, *J* = 7.7 Hz, 1H), 3.86 (dd, *J* = 14.6, 6.1 Hz, 1H), 3.70 (s, 1H), 3.65 (t, *J* = 11.6 Hz, 2H), 3.01 – 2.96 (m, 2H), 2.65 (s, 3H).

^{13}C NMR (151 MHz, 300 K, CDCl$_3$): δ 171.3, 149.3, 146.2, 137.2, 133.2, 132.5, 131.9, 131.5, 129.0, 121.5, 117.8, 115.7, 111.9, 107.7, 101.0, 58.1, 45.6, 41.9, 39.0, 29.7, 22.7, 21.1.

IR (thin film): $\tilde{\nu}$ 2960, 2923, 2853, 1655, 1598, 1507, 1461, 1421, 1377, 1260, 1104, 1011, 941, 845, 803, 747, 693, 665 cm^{-1}.

HRMS (ESI): *m/z* calculated for $C_{23}H_{25}N_4O$ [M+H]$^+$: 373.2023, found: 373.2028.

$[\alpha]_D^{26}$ +2.5 (*c* 0.1, CHCl$_3$, 57% *ee* sample).

Enantiomeric excess was determined to be 57% *ee* (*standard procedure 2, using* ***L2***) by chiral stationary phase HPLC analysis (CHIRALPAK IA (ϕ 0.46 cm x 25 cm), *n*-hexane/^{i}PrOH = 9/1, flow rate 1.0 mL/min, detection at 254 nm, t$_R$ = 15.8 min (major), 22.8 min (minor).

Enantiomeric excess was determined to be 49% *ee* (*standard procedure 2, using* ***L3***) by chiral stationary phase HPLC analysis (CHIRALPAK IA (ϕ 0.46 cm x 25 cm), *n*-hexane/^{i}PrOH = 9/1, flow rate 1.0 mL/min, detection at 254 nm, t$_R$ = 15.8 min (major), 22.8 min (minor).

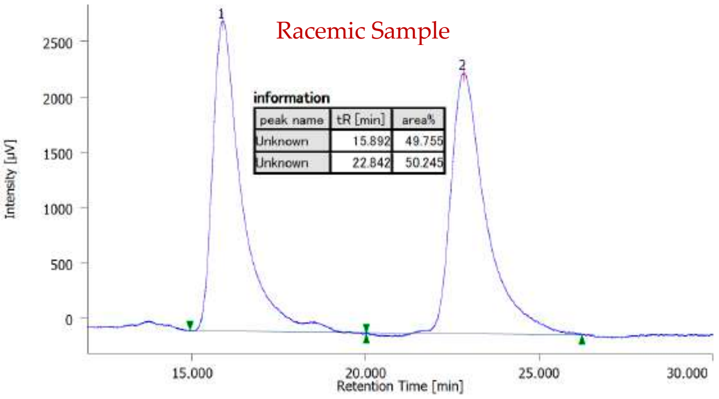

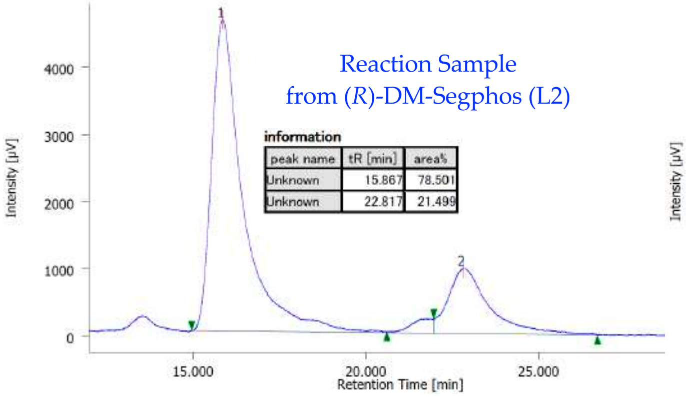

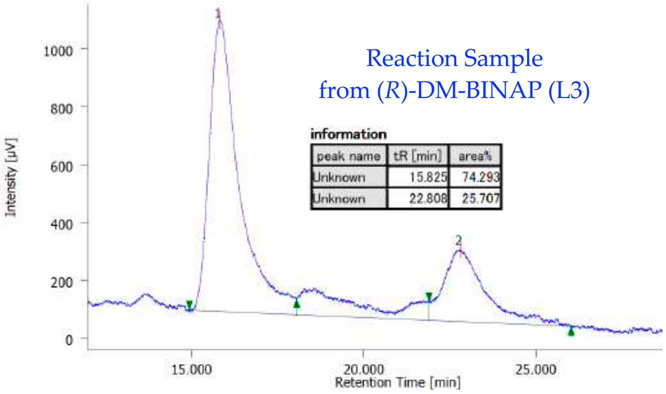

(*R*)-1-(6-methoxy-2,3-dihydro-1*H*-pyrrolo[2,3-*b*]pyridin-1-yl)-4-(methyl(phenyl)amino)-3-(pyridin-2-yl)butan-1-one (3ma):

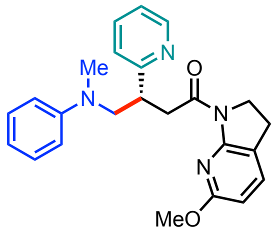

The reaction performed according to the standard procedure 2 afforded 34 mg (85%). Yellow oil.

^{1}H NMR (400 MHz, 300 K, $CDCl_3$): δ 8.54 (ddd, *J* = 4.8, 1.8, 0.9 Hz, 1H), 7.50 (td, *J* = 7.6, 1.9 Hz, 1H), 7.33 (dt, *J* = 8.1, 1.0 Hz, 1H), 7.22 – 7.13 (m, 2H), 7.12 (dt, *J* = 7.7, 1.1 Hz, 1H), 7.12 – 7.04 (m, 1H), 6.74 – 6.67 (m, 2H), 6.64 (tt, *J* = 7.3, 1.0 Hz, 1H), 6.32 (d, *J* = 8.1 Hz, 1H), 4.13 – 3.99 (m, 2H), 3.96 – 3.88 (m, 2H), 3.87 (s, 3H), 3.81 – 3.64 (m, 2H), 3.62 – 3.52 (m, 1H), 2.95 – 2.88 (m, 2H), 2.62 (s, 3H).

^{13}C NMR (101 MHz, 300 K, $CDCl_3$): δ 171.2, 163.1, 163.1, 153.9, 149.4, 149.3, 136.2, 136.0, 129.2, 124.7, 121.6, 116.7, 116.0, 112.1, 103.5, 58.2, 54.0, 46.6, 41.8, 39.4, 39.0, 23.8.

IR (thin film): $\tilde{\nu}$ 2918, 2851, 1652, 1591, 1558, 1540, 1507, 1473, 1456, 1419, 1507, 1473, 1456, 1419, 1293, 1253, 1196, 1159, 1093, 1022, 907, 808, 748, 693, 507 cm^{-1}.

HRMS (ESI): *m*/*z* calculated for $C_{24}H_{27}N_4O_2$ $[M+H]^+$: 403.2129, found: 403.2136.

$[\alpha]_D^{26}$ -9.6 (*c* 0.1, $CHCl_3$, 88% *ee* sample).

Enantiomeric excess was determined to be 88% *ee* (*standard procedure 2, using* ***L2***) by chiral stationary phase HPLC analysis (CHIRALPAK IA (*ϕ* 0.46 cm x 25 cm), *n*-hexane/^{i}PrOH = 9/1, flow rate 1.0 mL/min, detection at 254 nm, t_R = 10.6 min (major), 16.2 min (minor).

Enantiomeric excess was determined to be 62% *ee* (*standard procedure 2, using* ***L3***) by chiral stationary phase HPLC analysis (CHIRALPAK IA (*ϕ* 0.46 cm x 25 cm), *n*-hexane/^{i}PrOH = 9/1, flow rate 1.0 mL/min, detection at 254 nm, t_R = 10.6 min (major), 15.5 min (minor).

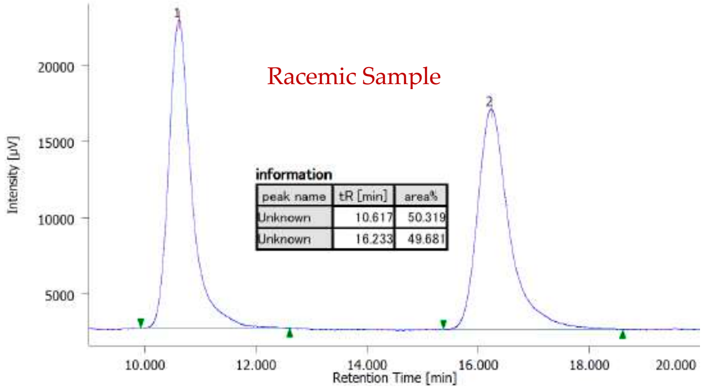

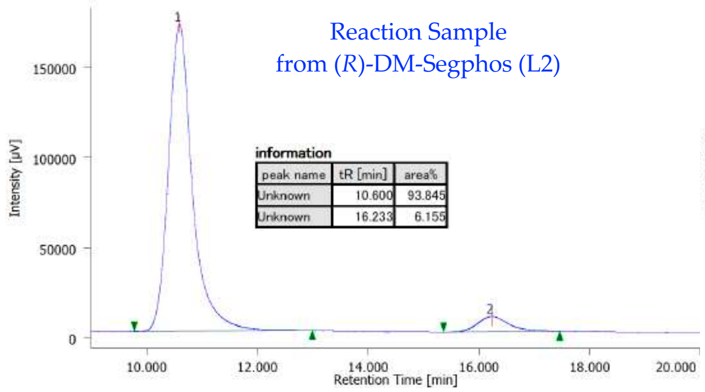

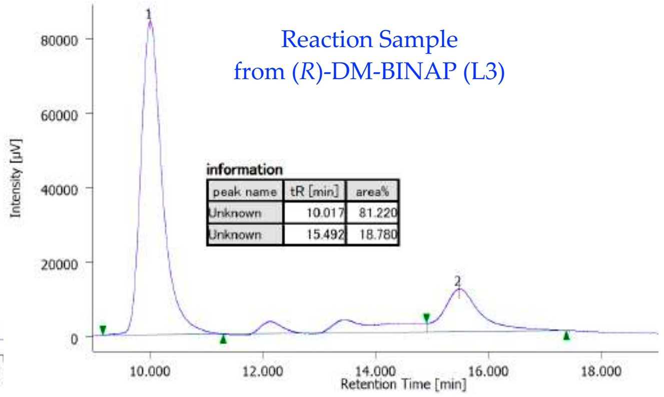

(*R*)-1-(6-methoxy-2,3-dihydro-1*H*-pyrrolo[2,3-*b*]pyridin-1-yl)-3-((methyl(phenyl)amino)methyl)pentan-1-one (3na):

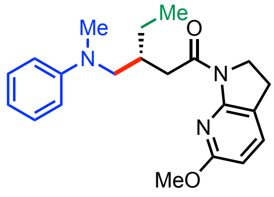

The reaction performed according to the standard procedure 2 afforded 33 mg (93%). Colorless oil.

^{1}H NMR (400 MHz, 300 K, CDCl$_3$): δ 7.33 (dt, *J* = 8.1, 1.1 Hz, 1H), 7.18 – 7.12 (m, 2H), 6.73 (dt, *J* = 7.9, 1.0 Hz, 2H), 6.63 – 6.58 (m, 1H), 6.32 (d, *J* = 8.1 Hz, 1H), 4.10 – 4.02 (m, 2H), 3.88 (s, 3H), 3.42 (dd, *J* = 14.4, 7.2 Hz, 1H), 3.32 – 3.12 (m, 3H), 2.93 (s, 3H), 2.88 (dddd, *J* = 9.0, 7.9, 4.0, 1.1 Hz, 1H), 2.56 (tt, *J* = 7.4, 5.5 Hz, 1H), 1.62 – 1.49 (m, 2H), 1.42 (dt, *J* = 14.1, 7.2 Hz, 1H), 0.97 (t, *J* = 7.5 Hz, 3H).

^{13}C NMR (101 MHz, 300 K, CDCl$_3$): δ 172.0, 163.0, 153.9, 150.1, 136.1, 129.1, 117.0, 115.9, 112.2, 103.4, 56.9, 53.8, 46.8, 39.1, 38.2, 35.5, 25.4, 23.7, 11.3.

IR (thin film): $\tilde{\nu}$ 2960, 2934, 2874, 1654, 1596, 1559, 1507, 1473, 1448, 1418, 1395, 292, 1251, 1219, 1195, 1159, 1092, 1027, 992, 808, 772, 747, 692, 670, 535, 506 cm^{-1}.

HRMS (ESI): *m/z* calculated for $C_{21}H_{28}N_3O_2$ [M+H]$^+$: 354.2176, found: 354.2182.

$[\alpha]_D^{26}$ +15.1 (*c* 0.4, CHCl$_3$, 99% *ee* sample).

Enantiomeric excess was determined to be 99% *ee* (*standard procedure* 2) by chiral stationary phase HPLC analysis (CHIRALPAK IG3 (*ϕ* 0.46 cm x 25 cm), *n*-hexane/^{i}PrOH = 95/05, flow rate 1.0 mL/min, detection at 254 nm, t_R = 17.1 min (major), 20.0 min (minor).

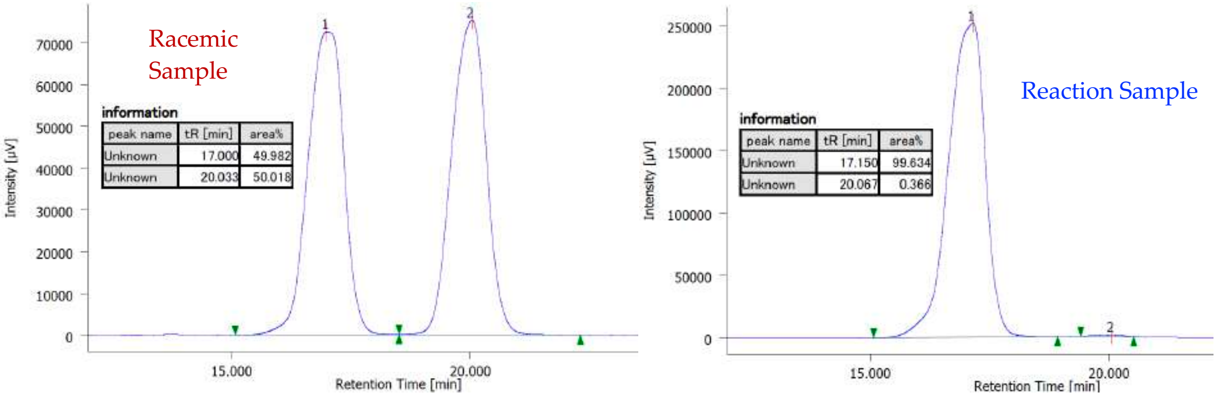

(*R*)-1-(6-methoxy-2,3-dihydro-1*H*-pyrrolo[2,3-*b*]pyridin-1-yl)-3-((methyl(phenyl)amino)methyl)heptan-1-one (3oa):

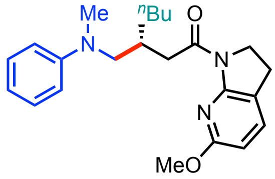

The reaction performed according to the standard procedure 2 afforded 35 mg (92%). Colorless oil.

^{1}H NMR (400 MHz, 300 K, CDCl$_3$): δ 7.33 (d, *J* = 8.1 Hz, 1H), 7.15 (dd, *J* = 8.9, 7.2 Hz, 2H), 6.72 (dt, *J* = 7.7, 1.1 Hz, 2H), 6.65 – 6.57 (m, 1H), 6.32 (d, *J* = 8.1 Hz, 1H), 4.10 – 4.00 (m, 2H), 3.88 (s, 3H), 3.42 (dd, *J* = 14.4, 7.5 Hz, 1H), 3.28 (dd, *J* = 16.6, 7.7 Hz, 1H), 3.22 – 3.09 (m, 2H), 2.93 (s, 3H), 2.92 – 2.82 (m, 2H), 2.70 – 2.54 (m, 1H), 1.66 – 1.21 (m, 6H), 0.87 (t, *J* = 7.1 Hz, 3H).

^{13}C NMR (101 MHz, 300 K, CDCl$_3$): δ 171.9, 162.9, 153.9, 150.1, 136.1, 129.1, 117.0, 115.9, 112.2, 103.3, 57.3, 53.8, 46.7, 39.1, 38.7, 34.1, 32.6, 29.2, 23.7, 23.2, 14.2.

IR (thin film): $\tilde{\nu}$ 2953, 2927, 2857, 1656, 1597, 1507, 1473, 1448, 1418, 1394, 1292, 1251, 1195, 1092, 1027, 990, 808, 747, 692 cm^{-1}.

HRMS (ESI): *m/z* calculated for $C_{23}H_{32}N_3O_2$ [M+H]$^+$: 382.2489, found: 382.2491.

$[\alpha]_D^{26}$ +53.8 (*c* 1.0, CHCl$_3$, 97% *ee* sample).

Enantiomeric excess was determined to be 97% *ee* (*standard procedure* 2) by chiral stationary phase HPLC analysis (CHIRALPAK IG3 (*ϕ* 0.46 cm x 25 cm), *n*-hexane/^{i}PrOH = 95/05, flow rate 1.0 mL/min, detection at 254 nm, t_R = 14.1 min (major), 20.6 min (minor).

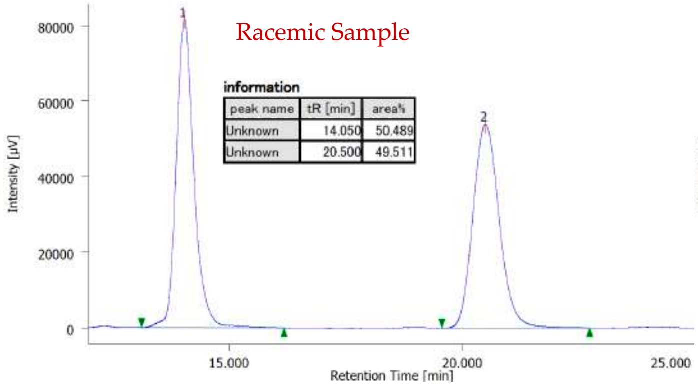

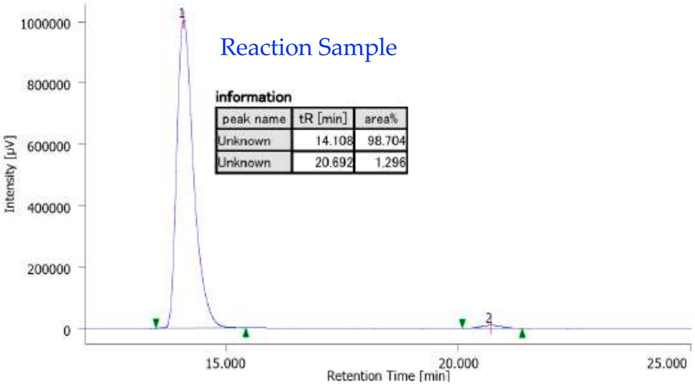

(*R*)-1-(6-methoxy-2,3-dihydro-1*H*-pyrrolo[2,3-*b*]pyridin-1-yl)-5-methyl-3-((methyl(phenyl)amino)methyl)hexan-1-one (3pa):

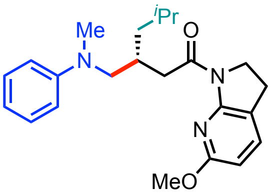

The reaction performed according to the standard procedure 2 afforded 37 mg (96%). Colorless oil.

^{1}H NMR (400 MHz, 300 K, CDCl$_3$): δ 7.32 (dt, *J* = 8.1, 1.1 Hz, 1H), 7.17 – 7.10 (m, 2H), 6.71 (dt, *J* = 7.9, 1.0 Hz, 2H), 6.60 (tt, *J* = 7.2, 1.0 Hz, 1H), 6.31 (d, *J* = 8.1 Hz, 1H), 4.04 (ddd, *J* = 9.3, 8.1, 1.6 Hz, 2H), 3.87 (s, 3H), 3.43 – 3.28 (m, 2H), 3.21 – 3.06 (m, 2H), 2.92 (s, 3H), 2.85 (dddd, *J* = 9.1, 8.0, 5.9, 1.1 Hz, 2H), 2.72 – 2.59 (m, 1H), 1.70 (dt, *J* = 13.4, 6.7 Hz, 1H), 1.35 – 1.22 (m, 2H), 0.93 (d, *J* = 6.6 Hz, 3H), 0.87 (d, *J* = 6.6 Hz, 3H).

^{13}C NMR (101 MHz, 300 K, CDCl$_3$): δ 171.9, 162.9, 153.9, 150.3, 136.1, 129.1, 117.0, 115.9, 112.2, 103.3, 57.7, 53.8, 46.7, 42.7, 39.0, 38.8, 32.1, 25.8, 23.7, 23.4, 22.8.

IR (thin film): $\tilde{\nu}$ 2953, 2866, 1655, 1597, 1507, 1473, 1449, 1419, 1394, 1292, 1251, 1195, 1159, 1092, 1028, 990, 808, 747, 692, 670 cm^{-1}.

HRMS (ESI): *m*/*z* calculated for $C_{23}H_{32}N_3O_2$ [M+H]$^+$: 382.2489, found: 382.2493.

$[\alpha]_D^{26}$ +25.6 (*c* 1.0, CHCl$_3$, 99% *ee* sample).

Enantiomeric excess was determined to be 99% *ee* (*standard procedure 2*) by chiral stationary phase HPLC analysis (CHIRALPAK IG3 (ϕ 0.46 cm x 25 cm), *n*-hexane/^{i}PrOH = 95/05, flow rate 1.0 mL/min, detection at 254 nm, t_R = 10.6 min (major), 12.8 min (minor).

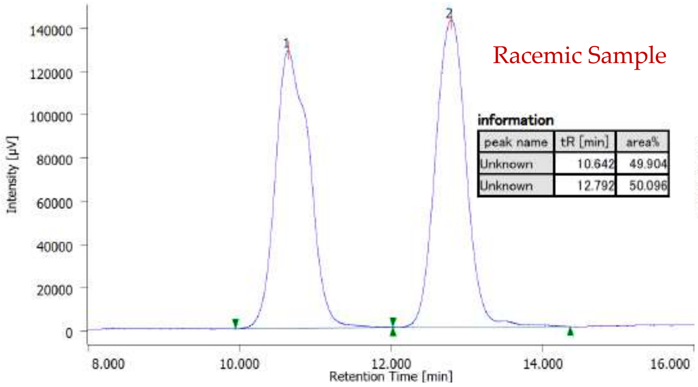

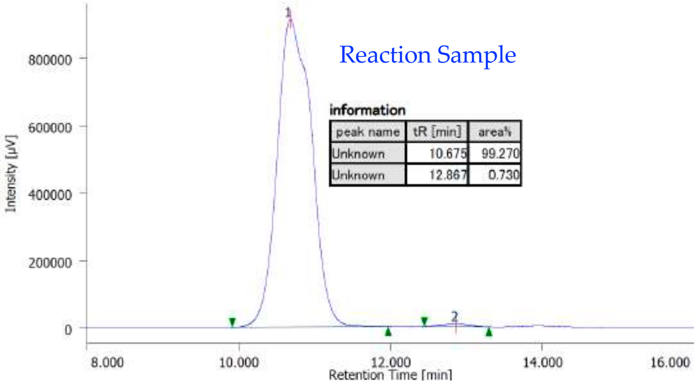

(*R*)-3-cyclopropyl-1-(6-methoxy-2,3-dihydro-1*H*-pyrrolo[2,3-*b*]pyridin-1-yl)-4-(methyl(phenyl)amino)butan-1-one (3qa):

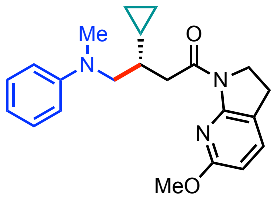

The reaction performed according to the standard procedure 2 afforded 34 mg (94%). Colorless oil.

^{1}H NMR (400 MHz, 300 K, CDCl$_3$): δ 7.34 (dt, *J* = 8.1, 1.1 Hz, 1H), 7.18 – 7.12 (m, 2H), 6.74 – 6.69 (m, 2H), 6.59 (tt, *J* = 7.2, 1.1 Hz, 1H), 6.33 (d, *J* = 8.1 Hz, 1H), 4.07 (t, *J* = 8.5 Hz, 2H), 3.89 (s, 3H), 3.65 (dd, *J* = 14.7, 6.5 Hz, 1H), 3.35 (d, *J* = 6.8 Hz, 2H), 3.29 (dd, *J* = 14.7, 7.6 Hz, 1H), 2.97 (s, 3H), 2.90 (tdd, *J* = 8.3, 3.3, 1.1 Hz, 2H), 1.86 – 1.72 (m, 1H), 0.78 – 0.65 (m, 1H), 0.49 – 0.32 (m, 2H), 0.20 – 0.06 (m, 2H).

^{13}C NMR (101 MHz, 300 K, CDCl$_3$): δ 171.8, 163.0, 153.9, 149.7, 136.2, 129.1, 117.1, 115.6, 111.8, 103.4, 57.7, 53.9, 46.8, 39.9, 39.4, 39.2, 23.7, 14.9, 4.5, 3.5.

IR (thin film): $\tilde{\nu}$ 2924, 1655, 1597, 1508, 1474, 1447, 1419, 1395, 1353, 1293, 1252, 1195, 1158, 1113, 1093, 1024, 992, 918, 896, 862, 809, 747, 693, 671, 535 cm^{-1}.

HRMS (ESI): *m/z* calculated for $C_{22}H_{28}N_3O_2$ [M+H]$^+$: 366.2182, found: 366.2175.

$[\alpha]_D^{26}$ -7.9 (*c* 0.12, CHCl$_3$, 98% *ee* sample).

Enantiomeric excess was determined to be 98% *ee* (*standard procedure 2*) by chiral stationary phase HPLC analysis (CHIRALPAK IG3 (ϕ 0.46 cm x 25 cm), *n*-hexane/^{i}PrOH = 95/05, flow rate 1.0 mL/min, detection at 254 nm, t_R = 20.2 min (major), 30.7 min (minor).

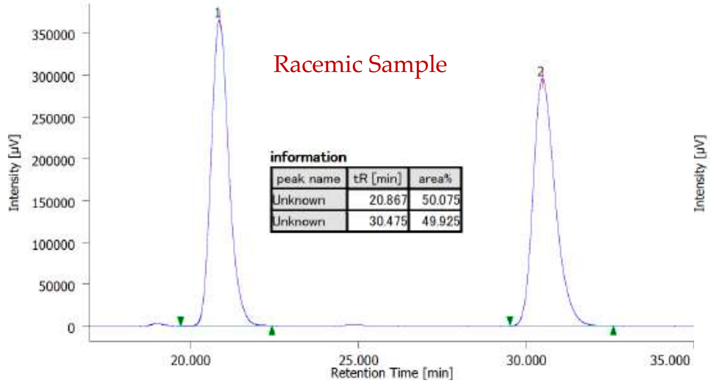

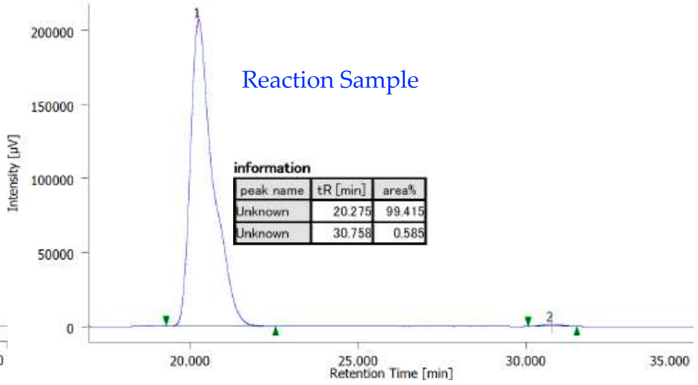

(*R*)-3-cyclohexyl-1-(6-methoxy-2,3-dihydro-1*H*-pyrrolo[2,3-*b*]pyridin-1-yl)-4-(methyl(phenyl)amino)butan-1-one (3ra):

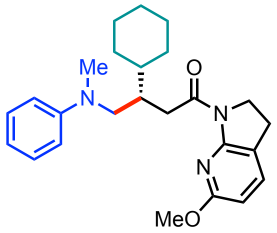

The reaction performed according to the standard procedure 2 afforded 38 mg (93%). Colorless oil.

^{1}H NMR (400 MHz, 300 K, CDCl$_3$): δ 7.33 – 7.30 (m, 1H), 7.14 (dd, *J* = 8.9, 7.2 Hz, 2H), 6.74 (dt, *J* = 7.8, 1.0 Hz, 2H), 6.59 (tt, *J* = 7.2, 1.0 Hz, 1H), 6.31 (d, *J* = 8.1 Hz, 1H), 4.03 (ddd, *J* = 9.5, 7.8, 2.0 Hz, 2H), 3.92 (s, 3H), 3.48 – 3.38 (m, 1H), 3.31 – 3.06 (m, 3H), 2.91 (s, 3H), 2.89 – 2.77 (m, 2H), 2.66 – 2.55 (m, 1H), 1.86 – 1.48 (m, 6H), 1.32 – 1.04 (m, 5H).

^{13}C NMR (101 MHz, 300 K, CDCl$_3$): δ 172.2, 162.9, 154.0, 150.2, 136.0, 129.0, 117.0, 115.8, 112.2, 103.3, 54.6, 53.9, 46.8, 39.2, 38.7, 38.6, 35.5, 31.1, 29.2, 27.0, 27.0, 26.9, 23.6.

IR (thin film): $\tilde{\nu}$ 2922, 2850, 1655, 1597, 1507, 1473, 1447, 1419, 1396, 1347, 1292, 1251, 1196, 1159, 1092, 1029, 998, 907, 808, 747, 693, 538 cm^{-1}.

HRMS (ESI): *m/z* calculated for $C_{25}H_{34}N_3O_2$ [M+H]$^+$: 408.2646, found: 408.2646.

$[\alpha]_D^{26}$ +21.4 (*c* 1.0, CHCl$_3$, 96% *ee* sample).

Enantiomeric excess was determined to be 96% *ee* (*standard procedure 2*) by chiral stationary phase HPLC analysis (CHIRALPAK IG3 (ϕ 0.46 cm x 25 cm), *n*-hexane/^{i}PrOH = 95/05, flow rate 1.0 mL/min, detection at 254 nm, t_R = 15.9 min (major), 25.8 min (minor).

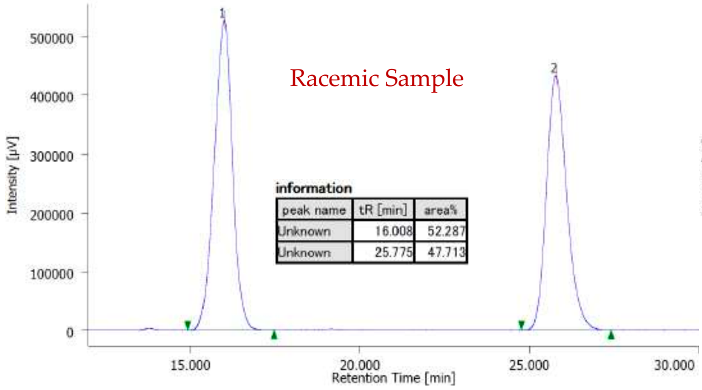

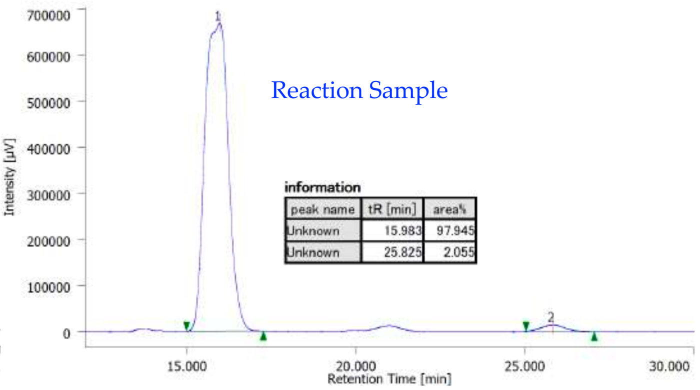

(*R*)-1-(6-methoxy-2,3-dihydro-1*H*-pyrrolo[2,3-*b*]pyridin-1-yl)-4-(methyl(phenyl)amino)-3-(*p*-tolyl)butan-1-one (3sa):

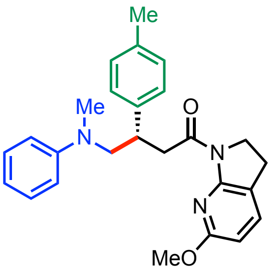

The reaction performed according to the standard procedure 2 afforded 37 mg (90%). Colorless oil.

^{1}H NMR (400 MHz, 300 K, CDCl$_3$): δ 7.33 (dt, *J* = 8.1, 1.1 Hz, 1H), 7.21 – 7.10 (m, 4H), 7.09 – 7.04 (m, 2H), 6.71 (dt, *J* = 7.7, 1.0 Hz, 2H), 6.63 (tt, *J* = 7.2, 1.0 Hz, 1H), 6.33 (d, *J* = 8.1 Hz, 1H), 4.08 – 4.01 (m, 2H), 3.91 – 3.85 (m, 1H), 3.84 (s, 3H), 3.83 – 3.74 (m, 1H), 3.69 – 3.54 (m, 2H), 3.25 (dd, *J* = 14.0, 7.8 Hz, 1H), 2.92 – 2.82 (m, 2H), 2.64 (s, 3H), 2.30 (s, 3H).

^{13}C NMR (101 MHz, 300 K, CDCl$_3$): δ 171.0, 163.0, 153.8, 149.3, 140.7, 136.1, 136.1, 129.2, 129.2, 127.9, 117.0, 115.9, 112.0, 103.6, 59.6, 53.8, 46.7, 39.9, 39.7, 39.3, 23.7, 21.2.

IR (thin film): $\tilde{\nu}$ 2935, 2905, 2862, 1653, 1591, 1507, 1473, 1419, 1394, 1375, 1347, 1294, 1251, 1219, 1195, 1159, 1118, 1092, 1031, 994, 948, 907, 865, 812, 771, 748, 693, 669, 539, 506 cm^{-1}.

HRMS (ESI): *m*/*z* calculated for $C_{26}H_{30}N_3O_2$ [M+H]$^+$: 416.2333, found: 416.2338.

$[\alpha]_D^{26}$ -32.2 (*c* 0.4, CHCl$_3$, 95% *ee* sample).

Enantiomeric excess was determined to be 95% *ee* (*standard procedure* 2) by chiral stationary phase HPLC analysis (CHIRALPAK IC (ϕ 0.46 cm x 25 cm), *n*-hexane/^{i}PrOH = 95/05, flow rate 1.0 mL/min, detection at 254 nm, t_R = 45.2 min (major), 50.2 min (minor).

Racemic Sample

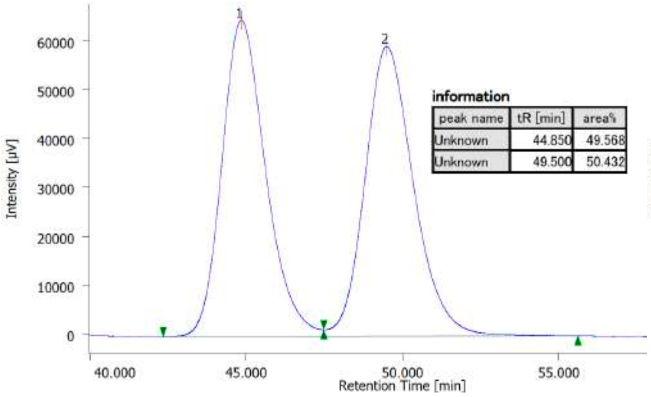

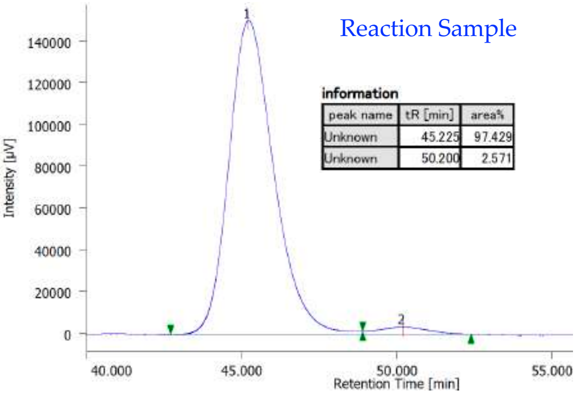

(*R*)-1-(6-methoxy-2,3-dihydro-1*H*-pyrrolo[2,3-*b*]pyridin-1-yl)-4-(methyl(phenyl)amino)-3-(*o*-tolyl)butan-1-one (3ta):

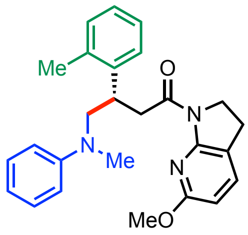

The reaction performed according to the standard procedure 2 afforded 38 mg (93%). Colorless oil.

^{1}H NMR (400 MHz, 300 K, CDCl$_3$): δ 7.37 – 7.28 (m, 2H), 7.21 – 7.11 (m, 3H), 7.11 – 7.05 (m, 2H), 6.75 – 6.68 (m, 2H), 6.67 – 6.58 (m, 1H), 6.33 (d, *J* = 8.1 Hz, 1H), 4.19 – 4.07 (m, 1H), 4.03 (ddd, *J* = 8.3, 7.0, 2.5 Hz, 2H), 3.90 (dd, *J* = 14.5, 6.5 Hz, 1H), 3.83 (s, 3H), 3.73 – 3.54 (m, 2H), 3.26 (dd, *J* = 14.5, 8.4 Hz, 1H), 2.88 (ddd, *J* = 9.8, 7.1, 1.1 Hz, 2H), 2.62 (s, 3H), 2.20 (s, 3H).

^{13}C NMR (101 MHz, 300 K, CDCl$_3$): δ 171.1, 163.0, 153.8, 149.4, 142.5, 137.1, 136.2, 130.6, 129.2, 126.2, 126.1, 117.0, 116.0, 111.9, 103.6, 59.5, 53.8, 46.7, 40.2, 39.1, 35.0, 23.7, 19.9.

IR (thin film): $\tilde{\nu}$ 2926, 2855, 1653, 1593, 1558, 1507, 1489, 1473, 1456, 1418, 1395, 1374, 1362, 1339, 1292, 1250, 1217, 1193, 1159, 1092, 1031, 993, 906, 809, 773, 746, 727, 692, 670, 560, 530, 518, 506 cm^{-1}.

HRMS (ESI): *m/z* calculated for $C_{26}H_{30}N_3O_2$ [M+H]$^+$: 416.2333, found: 416.2337.

$[\alpha]_D^{26}$ -44.6 (*c* 0.6, CHCl$_3$, 89% *ee* sample).

Enantiomeric excess was determined to be 89% *ee* (*standard procedure 2*) by chiral stationary phase HPLC analysis (CHIRALPAK IC (ϕ 0.46 cm x 25 cm), *n*-hexane/^{i}PrOH = 95/05, flow rate 1.0 mL/min, detection at 254 nm, t_R = 29.6 min (major), 39.5 min (minor).

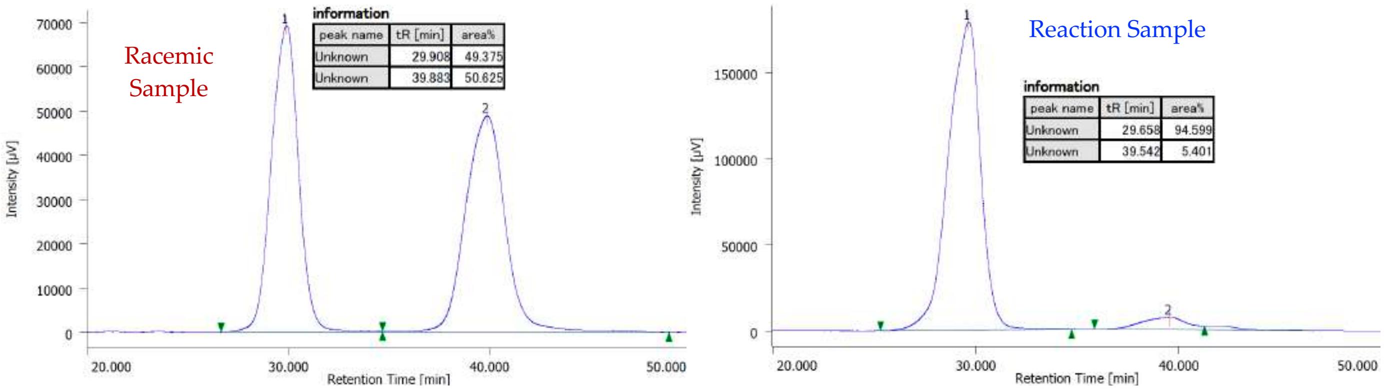

(*R*)-1-(6-methoxy-2,3-dihydro-1*H*-pyrrolo[2,3-*b*]pyridin-1-yl)-3-(4-methoxyphenyl)-4-(methyl(phenyl)amino)butan-1-one (3ua):

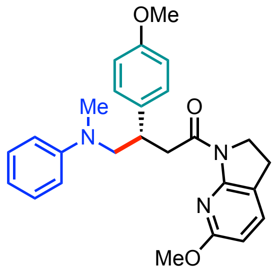

The reaction performed according to the standard procedure 2 afforded 41 mg (96%). Yellow oil.

^{1}H NMR (400 MHz, 300 K, CDCl$_3$): δ 7.34 (dt, *J* = 8.1, 1.1 Hz, 1H), 7.21 – 7.13 (m, 4H), 6.82 – 6.78 (m, 2H), 6.72 – 6.69 (m, 2H), 6.67 – 6.60 (m, 1H), 6.33 (d, *J* = 8.1 Hz, 1H), 4.04 (td, *J* = 8.5, 1.1 Hz, 2H), 3.90 – 3.82 (m, 1H), 3.84 (s, 3H), 3.77 (s, 3H), 3.80 – 3.72 (m, 1H), 3.71 – 3.51 (m, 2H), 3.23 (dd, *J* = 14.2, 8.2 Hz, 1H), 2.94 – 2.84 (m, 2H), 2.63 (s, 3H).

^{13}C NMR (101 MHz, 300 K, CDCl$_3$): δ 171.0, 163.0, 158.4, 153.8, 149.3, 136.2, 135.8, 129.2, 128.9, 117.0, 115.9, 113.9, 112.0, 103.6, 59.6, 55.4, 53.8, 46.8, 40.0, 39.3, 39.3, 23.7.

IR (thin film): $\tilde{\nu}$ 2922, 2853, 1653, 1607, 1509, 1473, 1419, 1396, 1294, 1251, 1177, 1093, 1032, 993, 907, 828, 809, 749, 694, 538 cm^{-1}.

HRMS (ESI): *m/z* calculated for $C_{26}H_{30}N_3O_3$ [M+H]$^+$: 432.2282, found: 432.2284.

$[\alpha]_D^{26}$ -26.2 (*c* 0.1, CHCl$_3$, 96% *ee* sample).

Enantiomeric excess was determined to be 96% *ee* (*standard procedure 2*) by chiral stationary phase HPLC analysis (CHIRALPAK IA (ϕ 0.46 cm x 25 cm), *n*-hexane/^{i}PrOH = 95/05, flow rate 1.0 mL/min, detection at 254 nm, t_R = 20.4 min (major), 37.3 min (minor).

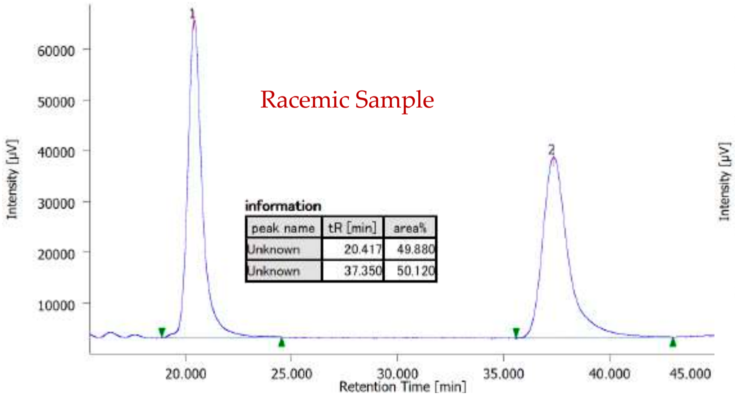

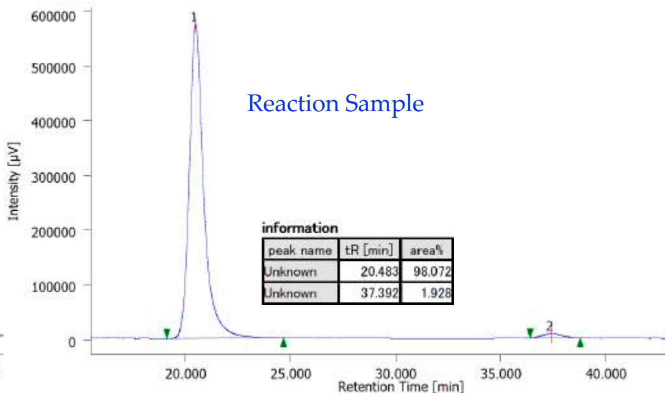

(*R*)-1-(6-methoxy-2,3-dihydro-1*H*-pyrrolo[2,3-*b*]pyridin-1-yl)-3-(3-methoxyphenyl)-4-(methyl(phenyl)amino)butan-1-one (3va):

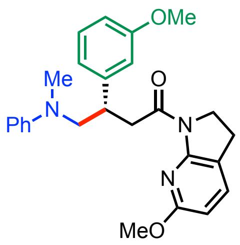

The reaction performed according to the standard procedure 2 afforded 42 mg (97%).
Yellow oil.
^{1}H NMR (400 MHz, 300 K, CDCl$_3$): δ 7.34 (dt, *J* = 8.1, 1.1 Hz, 1H), 7.17 (ddd, *J* = 8.1, 7.6, 1.7 Hz, 3H), 6.84 (dt, *J* = 7.6, 1.3 Hz, 1H), 6.78 (dd, *J* = 2.6, 1.6 Hz, 1H), 6.76 – 6.69 (m, 3H), 6.66 – 6.60 (m, 1H), 6.33 (d, *J* = 8.0 Hz, 1H), 4.05 (td, *J* = 8.4, 1.0 Hz, 2H), 3.91 – 3.86 (m, 1H), 3.85 (s, 3H), 3.84 – 3.75 (m, 1H), 3.73 (s, 3H), 3.72 – 3.52 (m, 2H), 3.34 – 3.24 (m, 1H), 2.91 – 2.85 (m, 2H), 2.66 (s, 3H).
^{13}C NMR (101 MHz, 300 K, CDCl$_3$): δ 170.9, 163.0, 159.8, 153.8, 149.3, 145.5, 136.2, 129.5, 129.2, 120.4, 117.0, 116.0, 113.9, 112.1, 112.0, 103.6, 59.5, 55.3, 53.8, 46.8, 40.2, 39.7, 39.3, 23.7.
IR (thin film): $\tilde{\nu}$ 2962, 2933, 2903, 2865, 1653, 1595, 1568, 1558, 1541, 1508, 1473, 1419, 1396, 1375, 1292, 1257, 1219, 1196, 1159, 1093, 1016, 802, 773, 747, 698, 669, 518, 506 cm^{-1}.
HRMS (ESI): *m*/*z* calculated for $C_{26}H_{30}N_3O_3$ [M+H]$^+$: 432.2282, found: 432.2282.
$[\alpha]_D^{26}$ -2.5 (*c* 0.1, CHCl$_3$, 98% *ee* sample).

Enantiomeric excess was determined to be 98% *ee* (*standard procedure* 2) by chiral stationary phase HPLC analysis (CHIRALPAK IE (ϕ 0.46 cm x 25 cm), *n*-hexane/^{i}PrOH = 95/05, flow rate 1.0 mL/min, detection at 254 nm, t_R = 52.5 min (major), 60.1 min (minor).

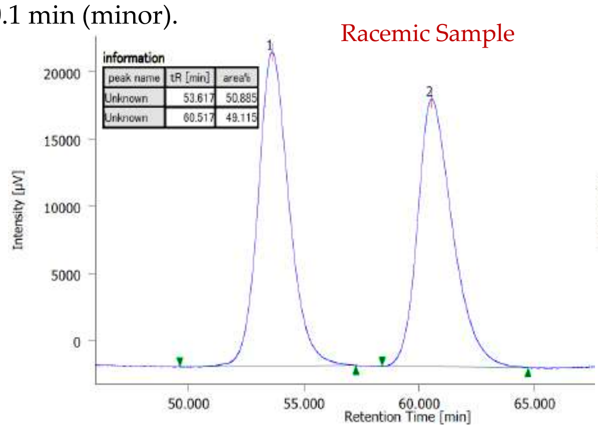

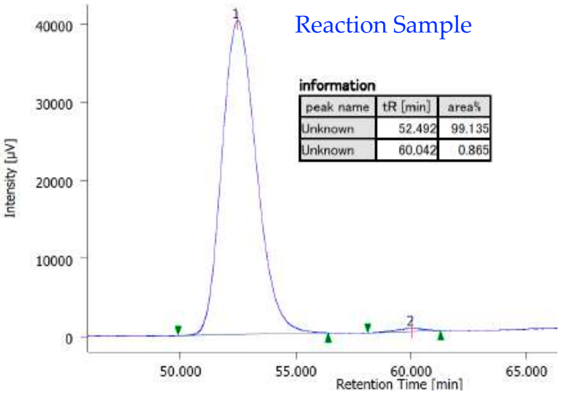

(*R*)-1-(6-methoxy-2,3-dihydro-1*H*-pyrrolo[2,3-*b*]pyridin-1-yl)-4-(methyl(phenyl)amino)-3-(4-(methylthio)phenyl)butan-1-one (3wa):

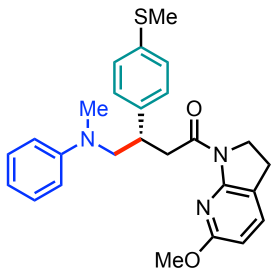

The reaction performed according to the standard procedure 2 afforded 44 mg (96%). Yellow oil.

^{1}H NMR (400 MHz, 300 K, $CDCl_3$): δ 7.34 (dt, *J* = 8.1, 1.0 Hz, 1H), 7.21 – 7.14 (m, 6H), 6.73 – 6.69 (m, 2H), 6.64 (tt, *J* = 7.2, 1.0 Hz, 1H), 6.34 (d, *J* = 8.1 Hz, 1H), 4.08 – 4.01 (m, 2H), 3.90 – 3.85 (m, 1H), 3.84 (s, 3H), 3.79 (tt, *J* = 8.1, 6.2 Hz, 1H), 3.72 – 3.50 (m, 2H), 3.26 (dd, *J* = 14.1, 8.0 Hz, 1H), 2.89 (td, *J* = 8.4, 1.0 Hz, 2H), 2.64 (s, 3H), 2.45 (s, 3H).

^{13}C NMR (101 MHz, 300 K, $CDCl_3$): δ 170.8, 163.0, 153.7, 149.2, 140.8, 136.3, 136.2, 129.2, 128.6, 127.0, 117.0, 116.0, 112.1, 103.6, 59.5, 53.8, 46.8, 39.7, 39.7, 39.4, 23.7, 16.2.

IR (thin film): $\tilde{\nu}$ 2987, 2954, 2907, 1653, 1596, 1540, 1507, 1473, 1447, 1396, 1293, 1251, 1219, 1196, 1159, 1093, 1024, 994, 813, 772, 749, 693, 670 cm^{-1}.

HRMS (ESI): *m/z* calculated for $C_{26}H_{30}N_3O_2S$ $[M+H]^+$: 448.2053, found: 448.2053.

$[\alpha]_D^{26}$ -174.0 (*c* 0.73, $CHCl_3$, 96% *ee* sample).

Enantiomeric excess was determined to be 96% *ee* (*standard procedure* 2) by chiral stationary phase HPLC analysis (CHIRALPAK IC3 (ϕ 0.46 cm x 25 cm), *n*-hexane/^{i}PrOH = 95/05, flow rate 1.0 mL/min, detection at 254 nm, t_R = 60.9 min (major), 79.9 min (minor).

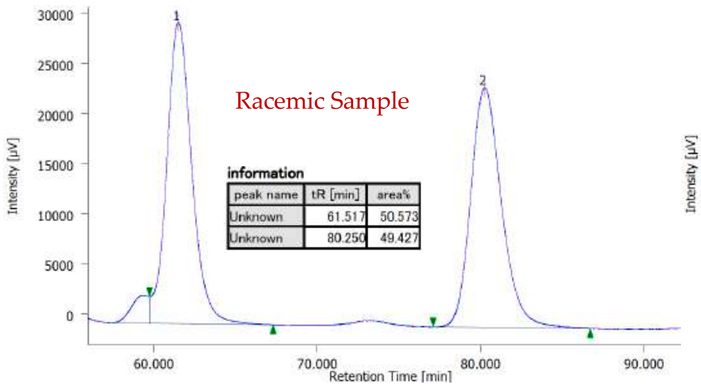

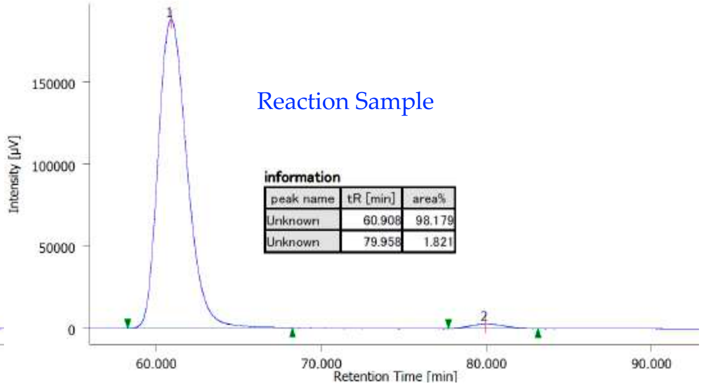

(*R*)-3-(4-fluorophenyl)-1-(6-methoxy-2,3-dihydro-1*H*-pyrrolo[2,3-*b*]pyridin-1-yl)-4-(methyl(phenyl)amino)butan-1-one (3xa):

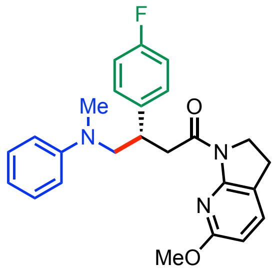

The reaction performed according to the standard procedure 2 afforded 39 mg (95%). Colorless oil.

^{1}H NMR (400 MHz, 300 K, $CDCl_3$): δ 7.35 (dt, *J* = 8.1, 1.0 Hz, 1H), 7.22 – 7.14 (m, 4H), 6.99 – 6.91 (m, 2H), 6.70 (dt, *J* = 7.9, 1.0 Hz, 2H), 6.65 (tt, *J* = 7.1, 1.0 Hz, 1H), 6.34 (d, *J* = 8.1 Hz, 1H), 4.04 (ddt, *J* = 11.0, 8.0, 2.6 Hz, 2H), 3.87 (dd, *J* = 14.0, 6.3 Hz, 1H), 3.84 (s, 3H), 3.85 – 3.75 (m, 1H), 3.74 – 3.48 (m, 2H), 3.25 (dd, *J* = 14.0, 8.1 Hz, 1H), 2.90 (ddd, *J* = 9.6, 6.9, 1.1 Hz, 2H), 2.63 (s, 3H).

^{13}C NMR (101 MHz, 300 K, $CDCl_3$): δ 170.7, 163.0, 161.8 (d, *J* = 244.3 Hz), 153.7, 149.2, 139.4 (d, *J* = 3.1 Hz), 136.3, 129.4 (d, *J* = 7.8 Hz), 129.2, 117.0, 116.1, 115.3 (d, *J* = 21.0 Hz), 112.1, 103.7, 59.6, 53.8, 46.8, 39.9, 39.4, 39.3, 23.72.

^{19}F NMR (376 MHz, $CDCl_3$): δ -116.60.

IR (thin film): $\tilde{\nu}$ 2926, 2904, 2858, 2833, 1652, 1597, 1558, 1507, 1473, 1418, 1394, 1373, 1348, 1292, 1250, 1218, 1194, 1158, 1092, 1031, 1023, 992, 947, 906, 832, 810, 773, 747, 692, 670, 529, 507, 491 cm^{-1}.

HRMS (ESI): *m/z* calculated for $C_{25}H_{27}N_3O_2F$ $[M+H]^+$: 420.2082, found: 420.2089.

$[\alpha]_D^{26}$ -33.5 (*c* 0.4, $CHCl_3$, 97% *ee* sample).

Enantiomeric excess was determined to be 97% *ee* (*standard procedure* 2) by chiral stationary phase HPLC analysis (CHIRALPAK IC (ϕ 0.46 cm x 25 cm), *n*-hexane/^{i}PrOH = 95/05, flow rate 1.0 mL/min, detection at 254 nm, t_R = 32.6 min (major), 43.1 min (minor).

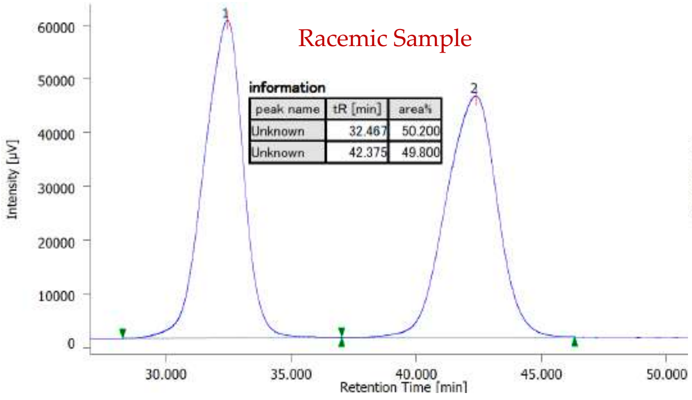

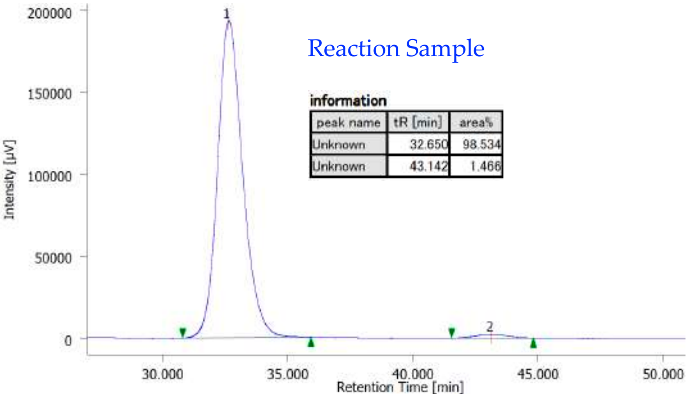

(*R*)-3-(4-bromophenyl)-1-(6-methoxy-2,3-dihydro-1*H*-pyrrolo[2,3-*b*]pyridin-1-yl)-4-(methyl(phenyl)amino)butan-1-one (3ya):

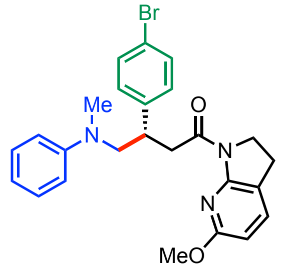

The reaction performed according to the standard procedure 2 afforded 46 mg (96%). Yellow oil.

^{1}H NMR (400 MHz, 300 K, CDCl$_3$): δ 7.41 – 7.34 (m, 3H), 7.22 – 7.16 (m, 2H), 7.14 – 7.10 (m, 2H), 6.70 (dt, *J* = 7.8, 1.0 Hz, 2H), 6.68 – 6.63 (m, 1H), 6.35 (d, *J* = 8.1 Hz, 1H), 4.08 – 4.00 (m, 2H), 3.91 – 3.85 (m, 1H), 3.84 (s, 3H), 3.80 (td, *J* = 7.5, 1.5 Hz, 1H), 3.72 – 3.48 (m, 2H), 3.25 (dd, *J* = 14.1, 8.1 Hz, 1H), 2.90 (ddd, *J* = 9.9, 7.0, 1.1 Hz, 2H), 2.64 (s, 3H).

^{13}C NMR (101 MHz, 300 K, CDCl$_3$): δ 170.5, 163.1, 153.7, 149.2, 142.8, 136.3, 131.6, 129.8, 129.3, 120.5, 117.1, 116.2, 112.1, 103.7, 59.5, 53.8, 46.7, 39.7, 39.6, 39.5, 23.7.

IR (thin film): $\tilde{\nu}$ 2925, 1653, 1590, 1559, 1507, 1487, 1473, 1419, 1396, 1292, 1250, 1219, 1195, 1054, 1032, 1008, 812, 772, 748, 693, 670, 529, 506 cm^{-1}.

HRMS (ESI): *m*/*z* calculated for $C_{25}H_{27}N_3O_2Br$ [M+H]$^+$: 480.1281, found: 480.1290.

$[\alpha]_D^{26}$ -57.8 (*c* 0.6, CHCl$_3$, 96% *ee* sample).

Enantiomeric excess was determined to be 96% *ee* (*standard procedure 2*) by chiral stationary phase HPLC analysis (CHIRALPAK IC (ϕ 0.46 cm x 25 cm), *n*-hexane/^{i}PrOH = 95/05, flow rate 1.0 mL/min, detection at 254 nm, t_R = 31.8 min (major), 41.8 min (minor).

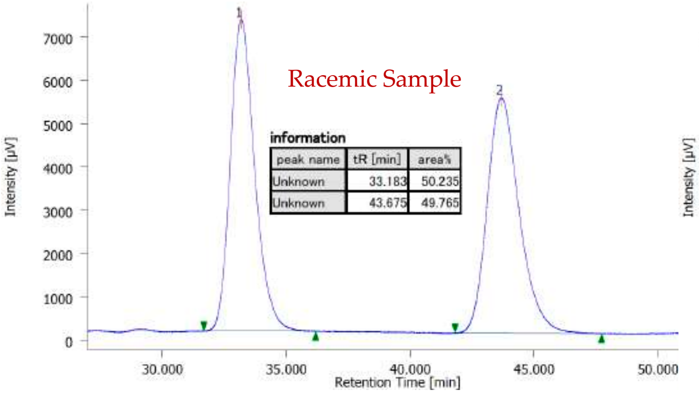

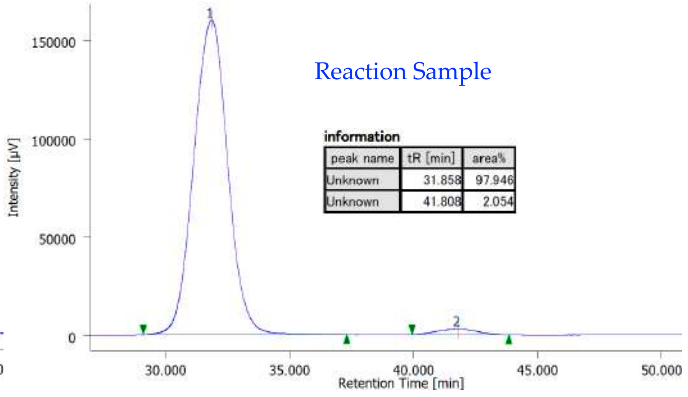

(*R*)-4-(4-(6-methoxy-2,3-dihydro-1*H*-pyrrolo[2,3-*b*]pyridin-1-yl)-1-(methyl(phenyl)amino)-4-oxobutan-2-yl)benzonitrile (3za):

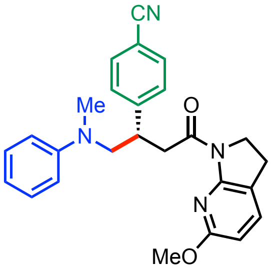

The reaction performed according to the standard procedure 2 afforded 32 mg (75%). Yellow oil.

^{1}H NMR (400 MHz, 300 K, $CDCl_3$): δ 7.58 – 7.54 (m, 2H), 7.39 – 7.34 (m, 3H), 7.22 – 7.16 (m, 2H), 6.71 – 6.65 (m, 3H), 6.36 (d, *J* = 8.1 Hz, 1H), 4.04 (ddd, *J* = 8.8, 7.4, 5.7 Hz, 2H), 3.95 – 3.86 (m, 2H), 3.85 (s, 3H), 3.77 – 3.50 (m, 2H), 3.36 – 3.26 (m, 1H), 2.96 – 2.88 (m, 2H), 2.65 (s, 3H).

^{13}C NMR (101 MHz, 300 K, $CDCl_3$): δ 170.0, 163.1, 153.6, 149.6, 149.0, 136.4, 132.4, 129.3, 128.9, 119.1, 117.1, 116.6, 112.3, 110.6, 103.8, 59.4, 53.8, 46.7, 40.4, 39.5, 39.3, 23.7.

IR (thin film): $\tilde{\nu}$ 2936, 2864, 2832, 2225, 1652, 1594, 1558, 1507, 1473, 1447, 1420, 1397, 1340, 1293, 1252, 1218, 1194, 1158, 1115, 1092, 1023, 993, 947, 906, 833, 809, 772, 748, 693, 670, 565, 551, 520, 506, 491 cm^{-1}.

HRMS (ESI): *m/z* calculated for $C_{26}H_{27}N_4O_2$ [M+H]$^{+}$: 427.2129, found: 427.2134.

$[\alpha]_D^{26}$ -8.5 (*c* 0.12, $CHCl_3$, 88% *ee* sample).

Enantiomeric excess was determined to be 88% *ee* (*standard procedure* 2) by chiral stationary phase HPLC analysis (CHIRALPAK IE (ϕ 0.46 cm x 25 cm), *n*-hexane/^{i}PrOH = 9/1, flow rate 1.0 mL/min, detection at 254 nm, t_R = 72.4 min (minor), 80.3 min (major).

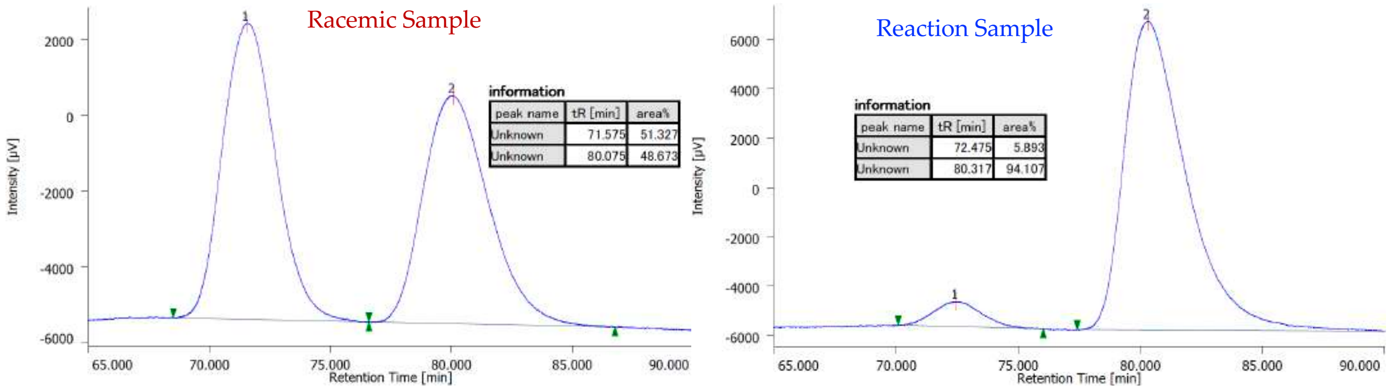

(*R*)-3-(furan-2-yl)-1-(6-methoxy-2,3-dihydro-1*H*-pyrrolo[2,3-*b*]pyridin-1-yl)-4-(methyl(phenyl)amino)butan-1-one (3aaa):

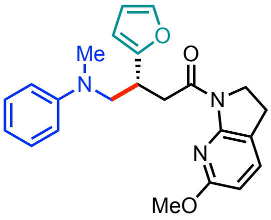

The reaction performed according to the standard procedure 2 afforded 33 mg (86%). Yellow oil.

^{1}H NMR (400 MHz, 300 K, $CDCl_3$): δ 7.35 (dt, *J* = 8.1, 1.1 Hz, 1H), 7.30 (dd, *J* = 1.9, 0.8 Hz, 1H), 7.21 – 7.12 (m, 2H), 6.73 – 6.66 (m, 2H), 6.66 – 6.58 (m, 1H), 6.33 (d, *J* = 8.1 Hz, 1H), 6.24 (dd, *J* = 3.2, 1.8 Hz, 1H), 6.02 (dt, *J* = 3.1, 0.7 Hz, 1H), 4.11 – 4.04 (m, 2H), 3.95 – 3.88 (m, 1H), 3.87 (s, 3H), 3.81 (dd, *J* = 14.4, 6.2 Hz, 1H), 3.70 – 3.53 (m, 2H), 3.46 (dd, *J* = 14.4, 8.4 Hz, 1H), 2.91 (ddd, *J* = 9.8, 7.3, 1.1 Hz, 2H), 2.71 (s, 3H).

^{13}C NMR (101 MHz, 300 K, $CDCl_3$): δ 170.5, 163.1, 156.6, 153.7, 149.2, 141.2, 136.2, 129.2, 116.9, 116.0, 112.0, 110.5, 106.3, 103.7, 56.6, 53.8, 46.7, 38.7, 38.1, 34.0, 23.8.

IR (thin film): $\tilde{\nu}$ 2915, 2848, 1652, 1591, 1558, 1540, 1507, 1473, 1456, 1420, 1293, 1252, 1219, 772, 692, 644, 510 cm^{-1}.

HRMS (ESI): *m/z* calculated for $C_{23}H_{26}N_3O_3$ [M+H]$^{+}$: 392.1969, found: 392.1971.

$[\alpha]_D^{26}$ -26.7 (*c* 0.12, $CHCl_3$, 93% *ee* sample).

Enantiomeric excess was determined to be 93% *ee* (*standard procedure* 2) by chiral stationary phase HPLC analysis (CHIRALPAK IE (ϕ 0.46 cm x 25 cm), *n*-hexane/^{i}PrOH = 95/05, flow rate 1.0 mL/min, detection at 254 nm, t_R = 33.1 min (major), 37.8 min (minor).

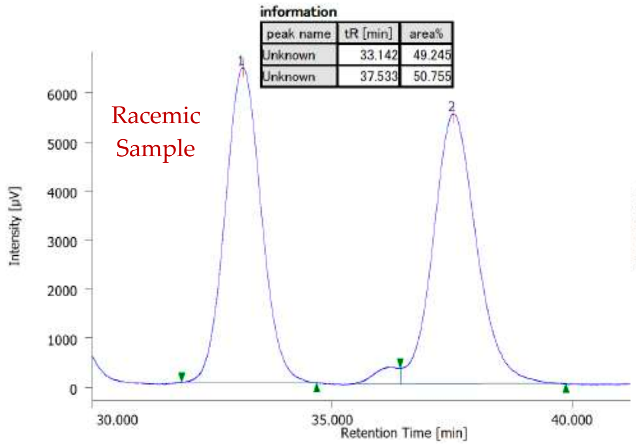

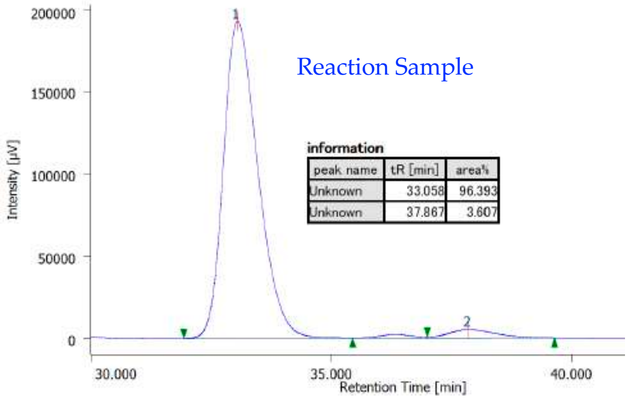

(*R*,*S*)-1-(6-methoxy-2,3-dihydro-1*H*-pyrrolo[2,3-*b*]pyridin-1-yl)-2,3-dimethyl-4-(methyl(phenyl)amino)butan-1-one (3aba):

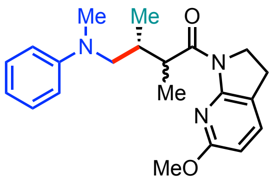

The reaction performed according to the standard procedure 2 afforded 23 mg (66%). Colorless oil. (*d.r.* 81:19)

^{1}H NMR (400 MHz, 300 K, $CDCl_3$, *major diastereomer*): δ 7.32 (dt, *J* = 8.1, 1.1 Hz, 1H), 7.13 – 7.07 (m, 2H), 6.68 – 6.64 (m, 2H), 6.60 – 6.55 (m, 1H), 6.34 (d, *J* = 8.1 Hz, 1H), 4.25 – 4.17 (m, 1H), 4.01 (dd, *J* = 9.6, 7.5 Hz, 2H), 3.91 (s, 3H), 3.89 – 3.83 (m, 2H), 3.63 (t, *J* = 8.6 Hz, 1H), 3.42 (dd, *J* = 14.3, 5.5 Hz, 1H), 3.05 – 2.96 (m, 2H), 2.88 (s, 3H), 2.86 – 2.70 (m, 2H), 2.48 (p, *J* = 8.0 Hz, 1H), 1.24 (d, *J* = 6.9 Hz, 3H), 0.98 (d, *J* = 6.8 Hz, 3H).

^{13}C NMR (101 MHz, 300 K, $CDCl_3$, *major diastereomer*): δ 176.0, 163.0, 153.8, 149.8, 136.2, 129.0, 117.4, 115.8, 112.0, 103.3, 58.3, 53.9, 46.9, 41.6, 39.1, 34.9, 23.6, 15.3, 15.1.

IR (thin film): $\tilde{\nu}$ 2929, 1655, 1598, 1508, 1473, 1417, 1397, 1293, 1252, 1221, 1158, 1093, 1030, 992, 808, 772, 747, 692, 642 cm^{-1}.

HRMS (ESI): *m*/*z* calculated for $C_{21}H_{28}N_3O_2$ $[M+H]^+$: 354.2176, found: 354.2179.

$[\alpha]_D^{26}$ -18.9 (*c* 0.21, $CHCl_3$, 34% *ee* sample, major diastereomer). *d.r.* 81:19

Enantiomeric excess was determined to be 34% *ee* (*major diastereomer*) and 74% *ee* (*minor diastereomer*) by chiral stationary phase HPLC analysis (CHIRALPAK IE (ϕ 0.46 cm x 25 cm), *n*-hexane/^{i}PrOH = 95/05, flow rate 0.5 mL/min, detection at 254 nm, t_R = 30.3 min (major, minor diatereomer), 32.1 min (minor, major diastereomer), 34.1 min (major, major diatereomer), 37.7 min (minor, minor diastereomer).

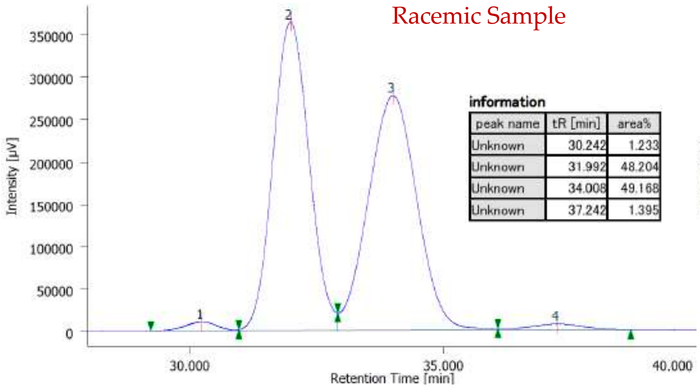

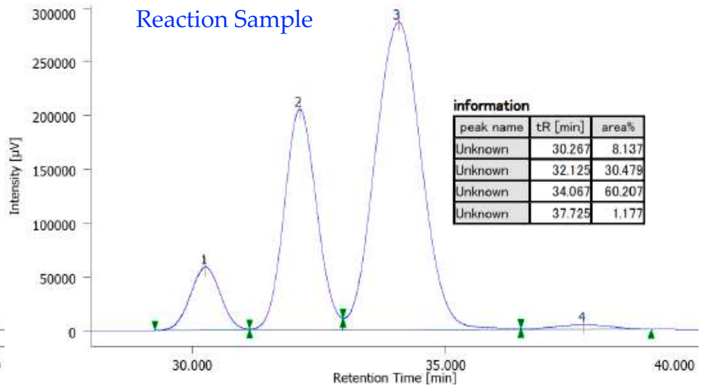

5.4: Standard Procedure 2: Variation of Amines

General procedure for asymmetric α-amino radical addition: An oven dried 10 mL Schlenk tube was charged with $[Ir(ppy)_2dtbbpy]PF_6$ (0.46 mg, 0.005 equiv., 0.5 mol%), $[Cu(MeCN)_4]PF_6$ (4.48 mg, 0.12 equiv., 12 mol%), (*R*)-DM-Segphos (10.8 mg, 0.15 equiv., 15 mol%) in the Glove box. To the mixture was added anhydrous DME (1.5 mL) via syringe with a stainless-steel needle at room temperature under positive Argon pressure. The resulting solution was stirred for 30 min and transferred to another Schlenk tube containing the acceptor **1d** (21.8 mg, 1.0 equiv, 0.1 mmol) in EtOH (0.5 mL). The reaction was carefully degassed by 3 freeze/pump/thaw cycles under Argon in the dark. The resulting reaction mixture was then allowed to stir for additional 30 min at room temperature before cooling down to -20 °C. Then, the amine **2** (1.2 equiv., 0.12 mmol) was added slowly to the reaction mixture and allowed to equilibrate for 15 min at -20 °C. This mixture was then irradiated by blue light (λ_{max} = 448 nm) for 48 h. The crude residue loaded directly onto a PLC (Silica gel 60, F_{254}, 0.5 mm, 20 x 20 cm, produced by Merck, Germany) and eluted using Hexane:EtOAc (~85:15) solvent system (2-4 times). The UV-visible product band was scratched and filtered through glass frit funnel using $CHCl_3$ as an eluent to afford **3**.

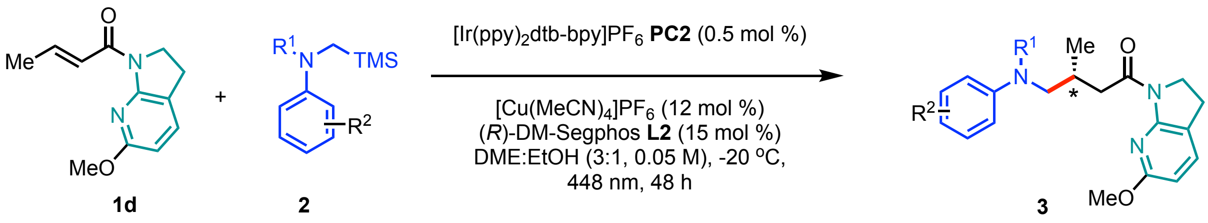

(*R*)-4-(benzyl(phenyl)amino)-1-(6-methoxy-2,3-dihydro-1*H*-pyrrolo[2,3-*b*]pyridin-1-yl)-3-methylbutan-1-one (3db):

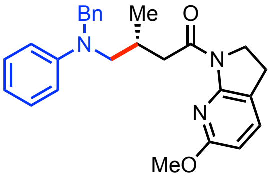

The reaction performed according to the standard procedure 2 afforded 40 mg (96%).
Yellow oil.
^{1}H NMR (400 MHz, 300 K, $CDCl_3$): δ 7.35 (dt, *J* = 8.1, 1.1 Hz, 1H), 7.29 – 7.23 (m, 2H), 7.22 – 7.09 (m, 5H), 6.74 (dt, *J* = 7.7, 1.0 Hz, 2H), 6.62 (tt, *J* = 7.2, 1.0 Hz, 1H), 6.33 (d, *J* = 8.1 Hz, 1H), 4.63 (ABq, *J* = 17.3 Hz, 2H), 4.12 – 4.04 (m, 2H), 3.85 (s, 3H), 3.61 (dd, *J* = 14.6, 6.4 Hz, 1H), 3.29 – 3.13 (m, 3H), 2.95 – 2.88 (m, 2H), 2.82 – 2.71 (m, 1H), 1.08 (d, *J* = 6.6 Hz, 3H).
^{13}C NMR (101 MHz, 300 K, $CDCl_3$): δ 171.5, 163.0, 153.8, 148.9, 138.8, 136.2, 129.2, 128.6, 126.7, 126.7, 117.0, 116.2, 112.7, 103.4, 57.7, 55.1, 53.8, 46.8, 41.3, 29.4, 23.7, 18.7.
IR (thin film): $\tilde{\nu}$ 2950, 1652, 1594, 1558, 1540, 1506, 1473, 1455, 1419, 1395, 1293, 1252, 1159, 1092, 1026, 808, 771, 748, 729, 694, 536, 512 cm^{-1}.
HRMS (ESI): *m*/*z* calculated for $C_{26}H_{30}N_3O_2$ $[M+H]^+$: 416.2333, found: 416.2336.
$[\alpha]_D^{26}$ +53.3 (*c* 1.0, $CHCl_3$, >99% *ee* sample).

Enantiomeric excess was determined to be >99% *ee* (*standard procedure 2*) by chiral stationary phase HPLC analysis (CHIRALPAK IE (ϕ 0.46 cm x 25 cm), *n*-hexane/^{i}PrOH = 95/05, flow rate 1.0 mL/min, detection at 254 nm, t_R = 27.1 min (major), 28.9 min (minor).

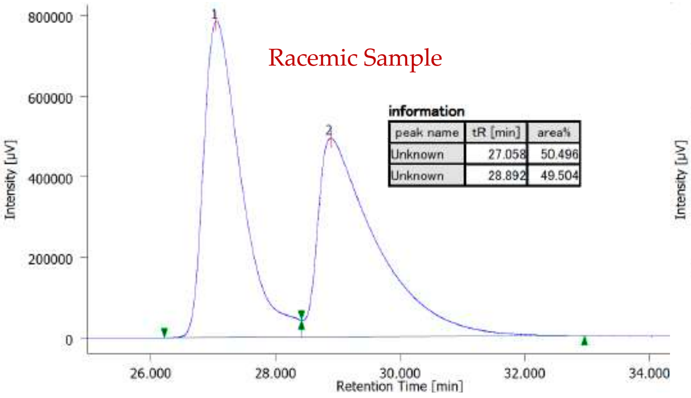

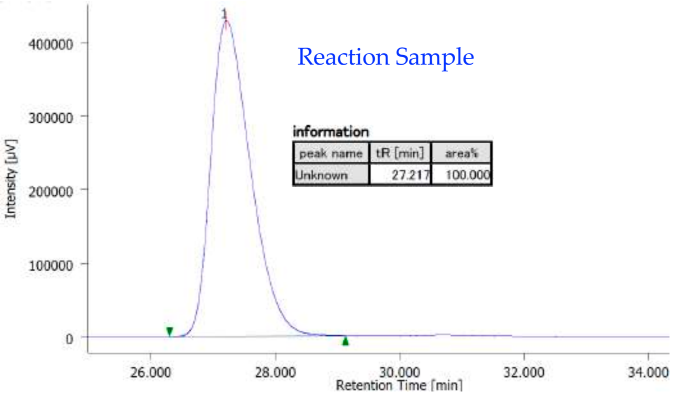

(*R*)-4-(isopropyl(phenyl)amino)-1-(6-methoxy-2,3-dihydro-1*H*-pyrrolo[2,3-*b*]pyridin-1-yl)-3-methylbutan-1-one (3dc):

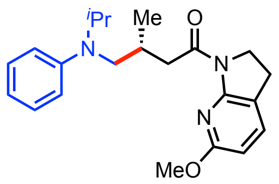

The reaction performed according to the standard procedure 2 afforded 33 mg (92%). Yellow oil.

^{1}H NMR (400 MHz, 300 K, $CDCl_3$): δ 7.37 (dt, *J* = 8.1, 1.0 Hz, 1H), 7.20 – 7.14 (m, 2H), 6.90 (dt, *J* = 7.8, 1.1 Hz, 2H), 6.70 (tt, *J* = 7.2, 1.0 Hz, 1H), 6.33 (d, *J* = 8.1 Hz, 1H), 4.15 – 4.08 (m, 2H), 4.02 – 3.90 (m, 1H), 3.88 (s, 3H), 3.26 – 3.16 (m, 3H), 2.98 – 2.92 (m, 2H), 2.88 (dd, *J* = 14.1, 8.6 Hz, 1H), 2.56 – 2.42 (m, 1H), 1.18 (d, *J* = 6.7 Hz, 3H), 1.11 (d, *J* = 6.7 Hz, 3H), 0.99 (d, *J* = 6.7 Hz, 3H).

^{13}C NMR (101 MHz, 300 K, $CDCl_3$): δ 172.0, 163.1, 153.9, 150.0, 136.1, 128.9, 117.8, 117.0, 103.4, 53.9, 52.3, 49.6, 46.8, 41.6, 28.6, 23.7, 20.6, 19.9, 18.7.

IR (thin film): $\tilde{\nu}$ 2958, 2919, 2867, 1653, 1636, 1594, 1558, 1540, 1520, 1506, 1498, 1473, 1456, 1418, 1395, 1293, 1252, 1218, 1179, 1158, 1092, 1027, 809, 771, 696 cm^{-1}.

HRMS (ESI): *m*/*z* calculated for $C_{22}H_{30}N_3O_2$ [M+H]$^+$: 368.2333, found: 368.2332.

$[\alpha]_D^{26}$ +44.8 (*c* 0.57, $CHCl_3$, 95% *ee* sample).

Enantiomeric excess was determined to be 95% *ee* (*standard procedure* 2) by chiral stationary phase HPLC analysis (CHIRALPAK IE (*ϕ* 0.46 cm x 25 cm), *n*-hexane/ethanol = 95/05, flow rate 1.0 mL/min, detection at 254 nm, t_R = 19.0 min (major), 20.6 min (minor).

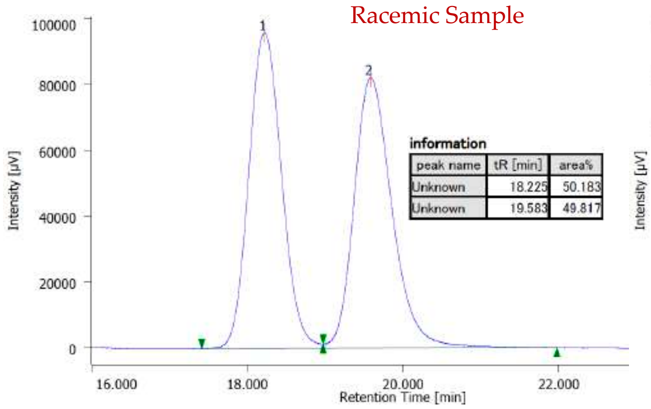

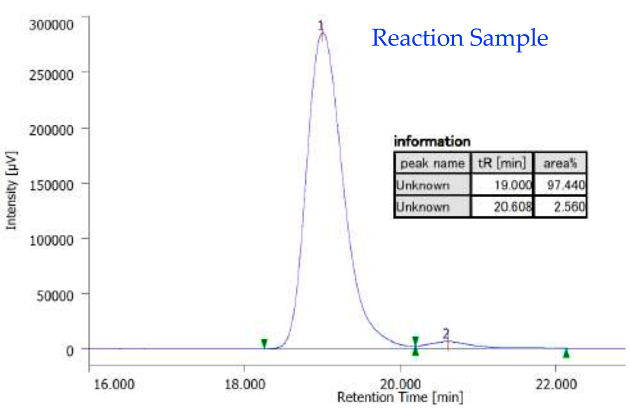

(*R*)-1-(6-methoxy-2,3-dihydro-1*H*-pyrrolo[2,3-*b*]pyridin-1-yl)-3-methyl-4-(phenylamino)butan-1-one (3dd):

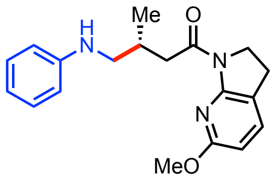

The reaction performed according to the standard procedure 2 afforded 31 mg (95%). Colorless solid (m.p. 110–112 °C).

^{1}H NMR (400 MHz, 300 K, $CDCl_3$): δ 7.36 (dt, *J* = 8.1, 1.1 Hz, 1H), 7.17 – 7.09 (m, 2H), 6.64 (tt, *J* = 7.3, 1.1 Hz, 1H), 6.61 – 6.55 (m, 2H), 6.34 (d, *J* = 8.1 Hz, 1H), 4.11 (dd, *J* = 9.2, 7.9 Hz, 2H), 3.87 (s, 3H), 3.34 (dd, *J* = 15.4, 7.2 Hz, 1H), 3.20 – 3.12 (m, 2H), 3.06 (dd, *J* = 12.2, 6.1 Hz, 1H), 2.98 – 2.88 (m, 2H), 2.60 – 2.45 (m, 1H), 1.11 (d, *J* = 6.8 Hz, 3H).

^{13}C NMR (101 MHz, 300 K, $CDCl_3$): δ 172.0, 163.1, 153.8, 148.7, 136.2, 129.3, 117.2, 116.9, 112.6, 103.3, 53.9, 50.4, 46.8, 41.8, 30.3, 23.7, 19.0.

IR (thin film): $\tilde{\nu}$ 2908, 1629, 1602, 1585, 1558, 1540, 1523, 1507, 1498, 1467, 1418, 1397, 1316, 1290, 1248, 1194, 1094, 1018, 903, 819, 772, 749, 692, 671, 536 cm^{-1}.

HRMS (ESI): *m*/*z* calculated for $C_{19}H_{24}N_3O_2$ [M+H]$^+$: 326.1863, found: 326.1866.

$[\alpha]_D^{26}$ +30.2 (*c* 1.0, $CHCl_3$, >99% *ee* sample).

Enantiomeric excess was determined to be >99% *ee* (*standard procedure* 2) by chiral stationary phase HPLC analysis (CHIRALPAK IC (*ϕ* 0.46 cm x 25 cm), *n*-hexane/^{i}PrOH = 95/05, flow rate 1.0 mL/min, detection at 254 nm, t_R = 59.8 min (major), 67.9 min (minor).

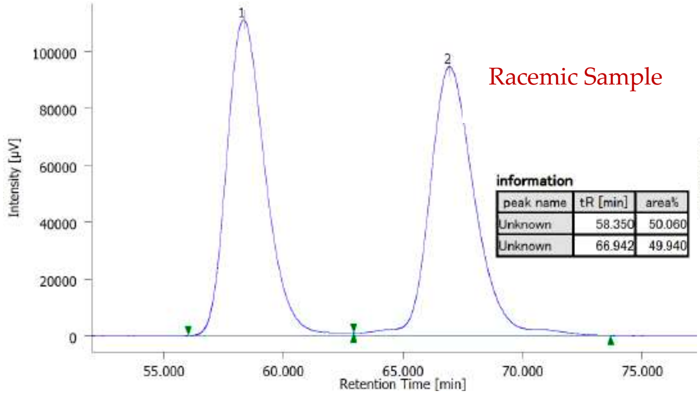

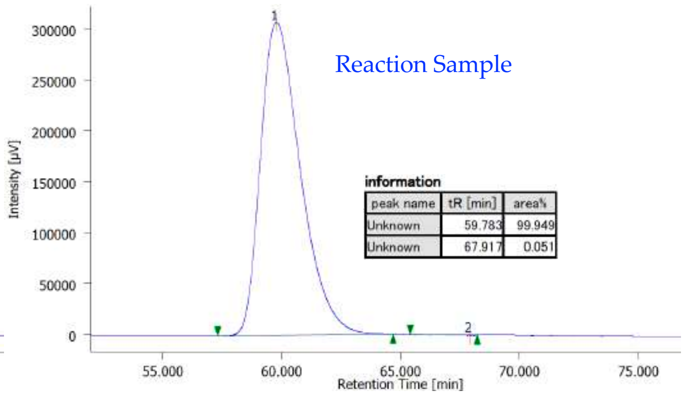

(*R*)-4-((2-fluorophenyl)(methyl)amino)-1-(6-methoxy-2,3-dihydro-1*H*-pyrrolo[2,3-*b*]pyridin-1-yl)-3-methylbutan-1-one (3de):

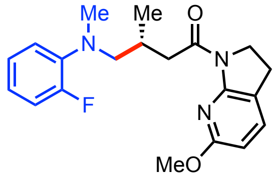

The reaction performed according to the standard procedure 2 afforded 33 mg (93%). Yellow oil.

^{1}H NMR (400 MHz, 300 K, CDCl$_3$): δ 7.34 (dt, *J* = 8.1, 1.1 Hz, 1H), 6.98 (ddd, *J* = 8.4, 7.2, 1.6 Hz, 1H), 6.97 – 6.86 (m, 2H), 6.82 – 6.72 (m, 1H), 6.30 (d, *J* = 8.1 Hz, 1H), 4.11 (dd, *J* = 9.1, 8.0 Hz, 2H), 3.82 (s, 3H), 3.29 (dd, *J* = 16.0, 5.9 Hz, 1H), 3.15 (ddd, *J* = 14.0, 7.8, 4.8 Hz, 2H), 3.03 – 2.90 (m, 3H), 2.82 (s, 3H), δ 2.60 (h, *J* = 7.2 Hz, 1H), 1.03 (d, *J* = 6.6 Hz, 3H).

^{13}C NMR (101 MHz, 300 K, CDCl$_3$): δ 172.1, 163.0, 155.1 (d, *J* = 244.3 Hz), 153.9, 140.8 (d, *J* = 8.2 Hz), 136.1, 124.4 (d, *J* = 3.5 Hz), 120.7 (d, *J* = 7.8 Hz), 119.2 (d, *J* = 3.5 Hz), 116.9, 116.2 (d, *J* = 21.2 Hz), 103.5, 61.07 (d, *J* = 4.4 Hz), 53.8 (d, *J* = 2.0 Hz), 46.7, 41.1, 40.2 (d, *J* = 2.7 Hz), 28.9, 23.7, 18.6.

^{19}F NMR (376 MHz, CDCl$_3$): δ -122.15.

IR (thin film): $\tilde{\nu}$ 2956, 1656, 1609, 1590, 1504, 1474, 1450, 1419, 1394, 1327, 1252, 1215, 1196, 1159, 1093, 1025, 974, 900, 809, 749, 535 cm^{-1}.

HRMS (ESI): *m/z* calculated for $C_{20}H_{25}N_3O_2F$ [M+H]$^+$: 358.1925, found: 358.1930.

$[\alpha]_D^{26}$ +39.2 (*c* 0.57, CHCl$_3$, 94% *ee* sample).

Enantiomeric excess was determined to be 94% *ee* (*standard procedure* 2) by chiral stationary phase HPLC analysis (CHIRALPAK IC (ϕ 0.46 cm x 25 cm), *n*-hexane/^{i}PrOH = 95/05, flow rate 1.0 mL/min, detection at 254 nm, t_R = 25.7 min (minor), 28.8 min (major).

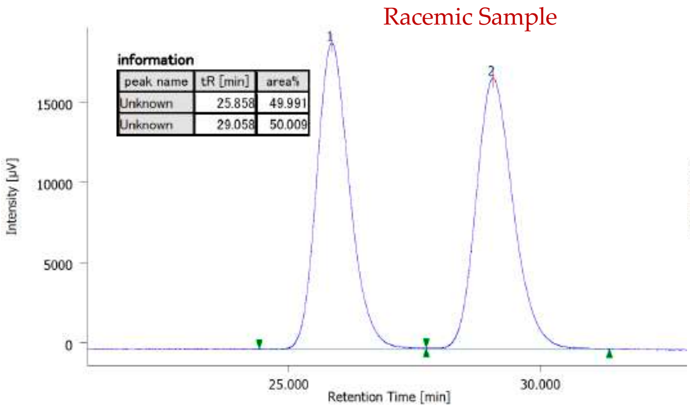

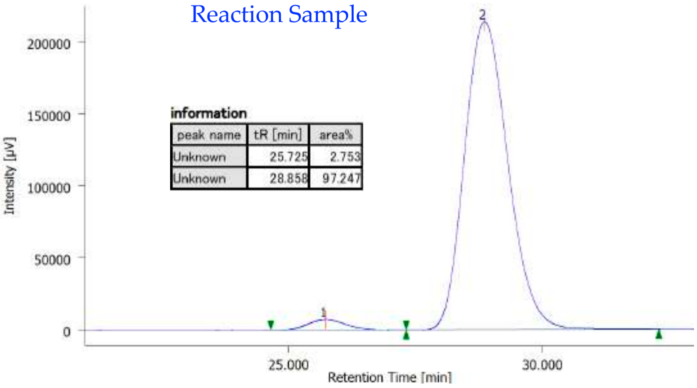

6. Gram-Scale Reaction

General procedure for asymmetric α-amino radical addition: An oven dried 200 mL three necked round bottomned flask equipped with a magnetic stirrer bar and 3 x LED (λ_{max} = 448 nm) was charged with [Ir(ppy)$_2$dtbbpy]PF$_6$ (14 mg, 0.003 equiv., 0.3 mol%), [Cu(MeCN)$_4$]PF$_6$ (187 mg, 0.10 equiv., 10 mol%), (*R*)-DM-Segphos (434 mg, 0.12 equiv., 12 mol%) under inert atmosphere. To the mixture was added anhydrous DME (75 mL) via syringe with a stainless-steel needle at room temperature under positive Argon pressure. The acceptor **1d** (1.09 g, 1.0 equiv, 5.0 mmol) was dissolved in EtOH (25 mL) and the resulting solution was transferred to 200 mL three necked round bottomned flask containing [Ir(ppy)$_2$dtbbpy]PF$_6$, [Cu(MeCN)$_4$]PF$_6$, and (*R*)-DM-Segphos. The reaction was carefully degassed by 3 freeze/pump/thaw cycles under Argon in the dark. The resulting reaction mixture was then allowed to stir for additional 30 min at room temperature before cooling down to -20 ^{o}C. Then, the amine **2a** (1.15 g, 1.2 equiv., 6.0 mmol) was added slowly to the reaction mixture and allowed to equilibrate for 30 min at -20 ^{o}C. This mixture was then irradiated by blue light (λ_{max} = 448 nm x 3) for 96 h. Purification of the crude product by automated flash column chromatography using Hexanes/EtOAc (9/1) solvent system afforded pure **3da**.
Enantiomeric excess was determined to be 92% *ee* by chiral stationary phase HPLC analysis (CHIRALPAK IG3 (ϕ 0.46 cm x 25 cm), *n*-hexane/^{i}PrOH l = 9/1, flow rate 1.0 mL/min, detection at 254 nm, t_R = 21.4 min (major), 24.1 min (minor). $[\alpha]_D^{26}$ 50.8 (*c* 1.0, CHCl$_3$, 92% *ee* sample).

Gram scale synthesis:

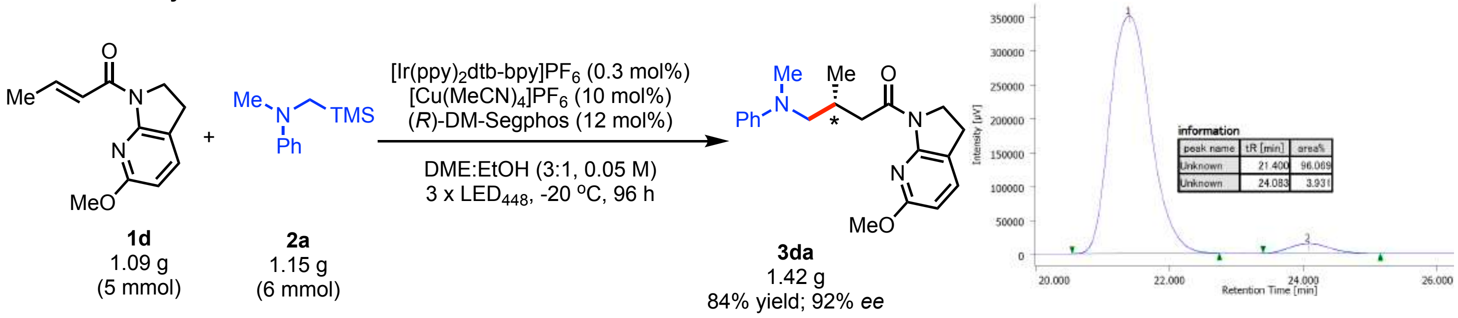

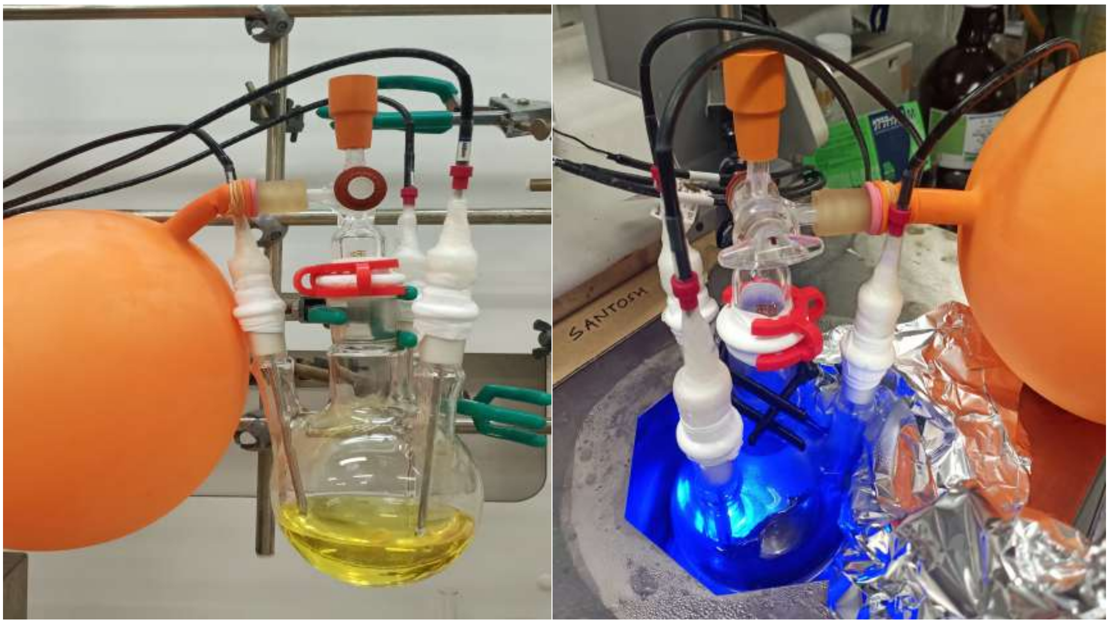

Fig. S3. Photochemical reaction set up for gram scale reaction at -20 ^{o}C

7. Transformations of the products

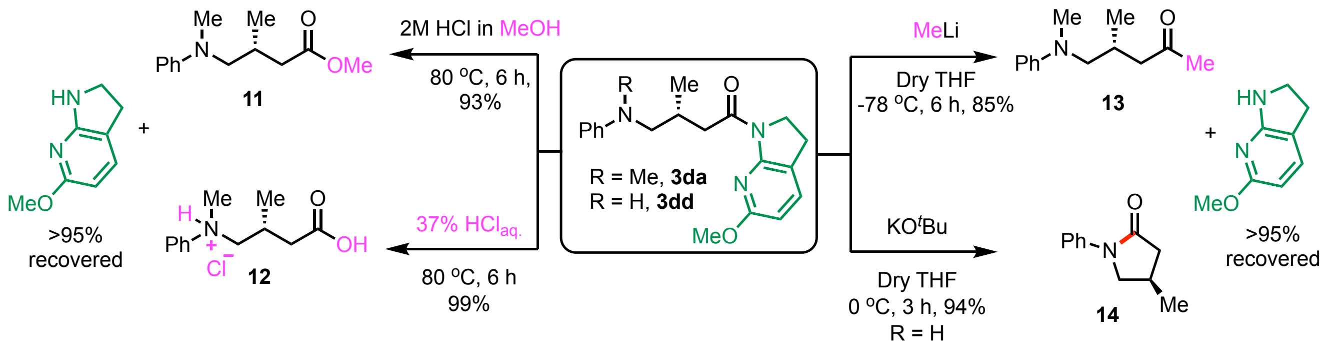

7.1. Esterification af amide (Procedure

A solution of the amide (**3da**) (115 mg, 0.34 mmol) in 2 M MeOH in HCl (5 mL) was heated in a sealed pressure tube to 80 °C for 6 h. After cooling to RT the volatiles were removed under reduced pressure and the residue was basified using saturated $NaHCO_3$ (20 mL) and extracted by EtOAc (3 x 20 mL). The combined organic layers were dried over Na_2SO_4 and purified by flash chromatography eluting with Hexane/EtOAc (9/1) to afford **11**. The 6-MeO-7-azaindoline was also recovered in excellent yields.

methyl (*R*)-3-methyl-4-(methyl(phenyl)amino)butanoate (11):

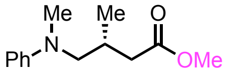

The reaction performed according to the procedure 7.1 afforded 70 mg (93%).
Yellow oil.
^{1}H NMR (400 MHz, 300 K, $CDCl_3$): δ 7.26 – 7.20 (m, 2H), 6.74 – 6.67 (m, 3H), 3.63 (s, 3H), 3.29 – 3.07 (m, 2H), 2.94 (s, 3H), 2.54 – 2.43 (m, 1H), 2.42 – 2.15 (m, 2H), 0.99 (d, *J* = 6.6 Hz, 3H).
^{13}C NMR (101 MHz, 300 K, $CDCl_3$): δ 173.4, 149.8, 129.3, 116.4, 112.9, 59.1, 51.6, 39.5, 39.3, 29.9, 18.0.
IR (thin film): $\tilde{\nu}$ 2953, 2873, 1736, 1599, 1572, 1507, 1452, 1435, 1375, 1355, 1260, 1213, 1192, 1173, 1123, 1078, 1033, 1010, 992, 970, 862, 836, 800, 749, 693 cm^{-1}.
HRMS (ESI): *m*/*z* calculated for $C_{13}H_{20}NO_2$ $[M+H]^+$: 222.1489, found: 222.1488.

7.2. Hydrolysis of amide

A solution of the amide (**3da**) (115 mg, 0.34 mmol) in 37% HCl (5 mL) was heated in a sealed pressure tube to 80 °C for 6 h. After cooling to RT the volatiles were removed under reduced pressure and the residue was purified by flash chromatography eluting with Methanol/DCM (5/95) to afford **12**. The 6-MeO-7-azaindoline was also recovered in quantitative yields.

***N*-((*R*)-3-carboxy-2-methylpropyl)-*N*-methylbenzenaminium chloride (12):**

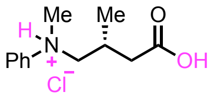

The reaction performed according to the standard procedure 7.2 afforded 81 mg (99%).
Yellow sticky oil.
^{1}H NMR (400 MHz, 300 K, MeOD): δ 7.17 – 7.11 (m, 2H), 6.75 – 6.70 (m, 2H), 6.64 – 6.58 (m, 1H), 3.33 – 3.28 (m, 1H), 3.29 – 3.04 (m, 2H), 2.92 (s, 3H), 2.46 – 2.29 (m, 2H), 2.18 – 2.09 (m, 1H), 0.96 (d, *J* = 6.6 Hz, 3H).
^{13}C NMR (101 MHz, 300 K, MeOD): δ 176.8, 151.1, 130.0, 117.3, 113.5, 59.9, 40.1, 39.8, 30.8, 18.1.
IR (thin film): $\tilde{\nu}$ 2962, 2931, 2874, 1706, 1599, 1507, 1461, 1451, 1429, 1408, 1375, 1344, 1295, 1259, 1221, 1191, 1120, 1077, 1033, 992, 970, 862, 770, 748, 693 cm^{-1}.
HRMS (ESI): *m*/*z* calculated for $C_{12}H_{17}NO_2Cl$ $[M+H]^+$: 242.0942, found: 242.0945.

7.3. Addition of MeLi to amide

To a solution of the amide (**3da**) (115 mg, 0.34 mmol) in dry THF (5 mL) was added MeLi (0.34 mL of 1 M solution in THF) at -78 °C and stirred for 6 h. Then, saturated NH_4Cl (5 mL) was added and reaction was warmed to rt. The crude product was extracted by EtOAc (3 x 20 mL) and the combined organic layers were dried over Na_2SO_4. The purification was done by flash chromatography eluting with Hexane/EtOAc (9/1) to afford **13**. The 6-MeO-7-azaindoline was also recovered in excellent yields.

(*R*)-4-methyl-5-(methyl(phenyl)amino)pentan-2-one (13):

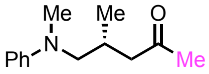

The reaction performed according to the standard procedure 7.3 afforded 59 mg (85%).
Yellow oil.
^{1}H NMR (400 MHz, 300 K, $CDCl_3$): δ 7.26 – 7.20 (m, 2H), 6.75 – 6.67 (m, 3H), 3.24 – 3.02 (m, 2H), 2.90 (s, 3H), 2.58 – 2.46 (m, 2H), 2.32 – 2.22 (m, 1H), 2.07 (s, 3H), 0.95 (d, *J* = 6.6 Hz, 3H).
^{13}C NMR (101 MHz, 300 K, $CDCl_3$): δ 208.4, 149.9, 129.3, 116.4, 112.4, 59.2, 48.8, 39.4, 30.5, 29.0, 18.7.
IR (thin film): $\tilde{\nu}$ 2959, 2928, 1711, 1599, 1572, 1548, 1505, 1496, 1462, 1451, 1373, 1355, 1260, 1219, 1168, 1124, 1079, 1033, 993, 966, 862, 771, 749, 693 cm^{-1}.
HRMS (ESI): *m*/*z* calculated for $C_{13}H_{20}NO$ [M+H]$^+$: 206.1539, found: 206.1543.

7.4. Lactam formation

To a solution of the amide (**3dd**) (100 mg, 0.3 mmol, 1.0 equiv.) in dry THF (5 mL) was added KO^{t}Bu (50 mg, 0.45 mmol, 1.5 equiv.) at 0 °C and stirred for 3 h. Then, saturated NH_4Cl (5 mL) was added and reaction was warmed to rt. The crude product was extracted by EtOAc (3 x 20 mL) and the combined organic layers were dried over Na_2SO_4. The purification was done by flash chromatography eluting with Hexane/EtOAc (9/1) to afford **14**. The 6-MeO-7-azaindoline was also recovered in excellent yields.

(*R*)-4-methyl-1-phenylpyrrolidin-2-one (14):

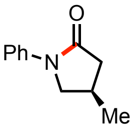

The reaction performed according to the standard procedure 7.4 afforded 50 mg (94%).
Yellow oil.
^{1}H NMR (400 MHz, 300 K, $CDCl_3$): δ 7.61 – 7.56 (m, 2H), 7.38 – 7.31 (m, 2H), 7.15 – 7.09 (m, 1H), 3.91 (dd, *J* = 9.5, 7.6 Hz, 1H), 3.42 (dd, *J* = 9.5, 6.4 Hz, 1H), 2.73 (dd, *J* = 16.7, 8.3 Hz, 1H), 2.62 – 2.46 (m, 1H), 2.23 (dd, *J* = 16.7, 7.4 Hz, 1H), 1.19 (d, *J* = 6.8 Hz, 3H).
^{13}C NMR (101 MHz, 300 K, $CDCl_3$): δ 173.8, 139.5, 128.8, 124.4, 119.9, 55.9, 41.0, 26.3, 19.5.
IR (thin film): $\tilde{\nu}$ 2962, 2873, 1698, 1598, 1498, 1457, 1395, 1353, 1296, 1282, 1221, 1175, 1159, 1122, 1100, 1067, 1031, 898, 759, 692, 667, 567 cm^{-1}.
HRMS (ESI): *m*/*z* calculated for $C_{11}H_{13}NONa$ [M+Na]$^+$: 198.0889, found: 198.0889.

8. Deuterium exchange experiment

Following the standard procedure 2, the deuterium exchange experiment was performed by using the EtOD instead of EtOH. The HRMS (Fig. S4) and NMR spectra (Fig. S5) confirms the deuterium incorporation in the product **3da-*d***.

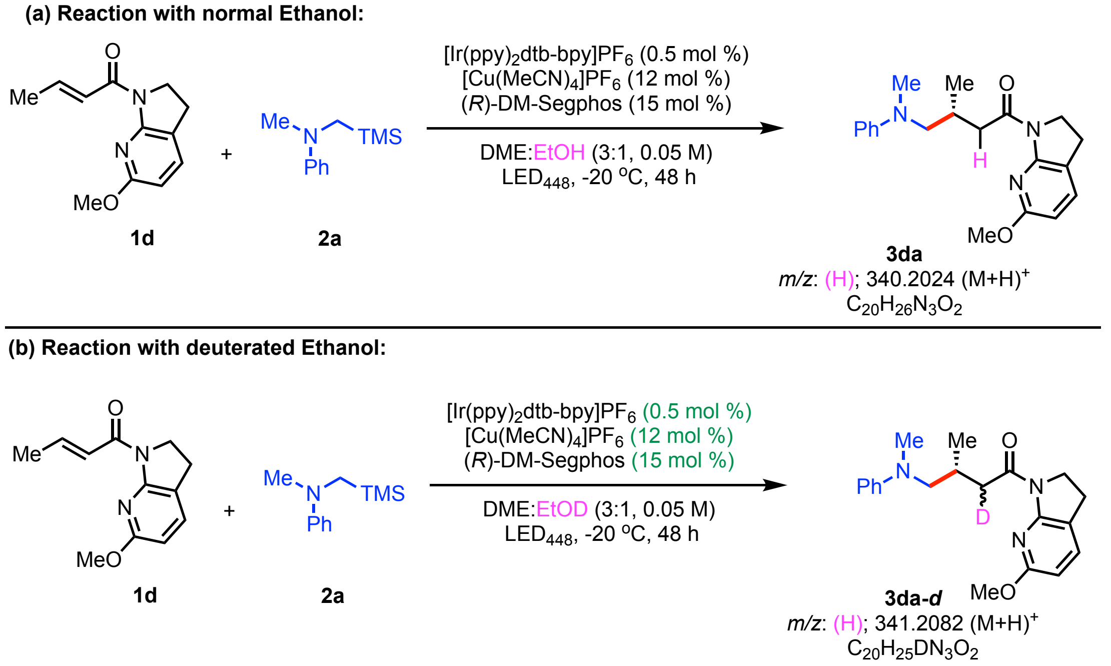

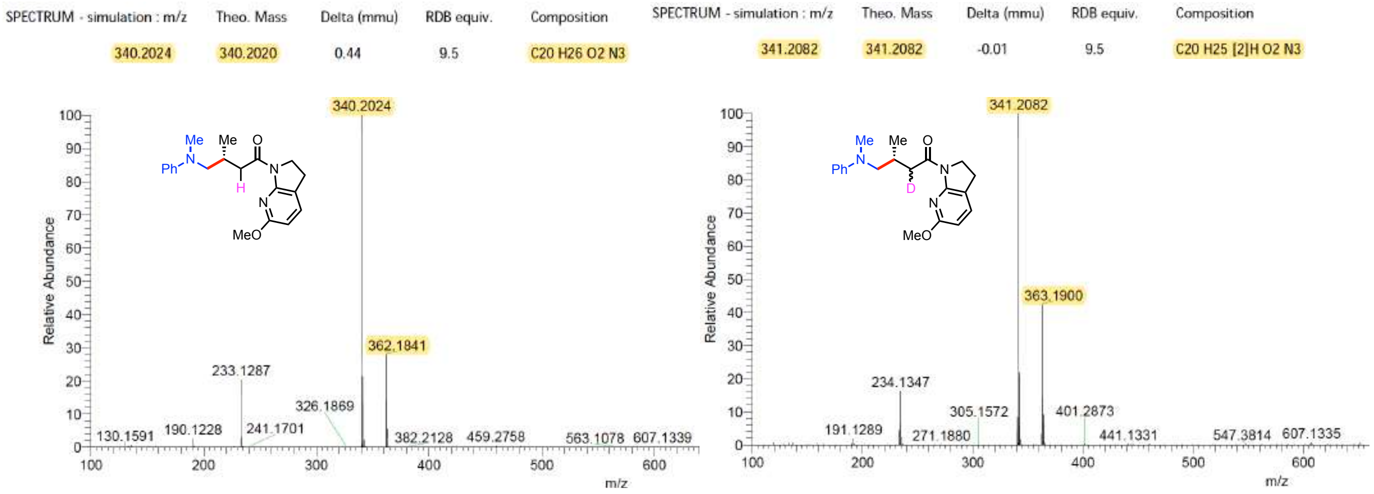

Fig S4: HRMS spectra of **3da** (left) and **3da-*d*** (right)

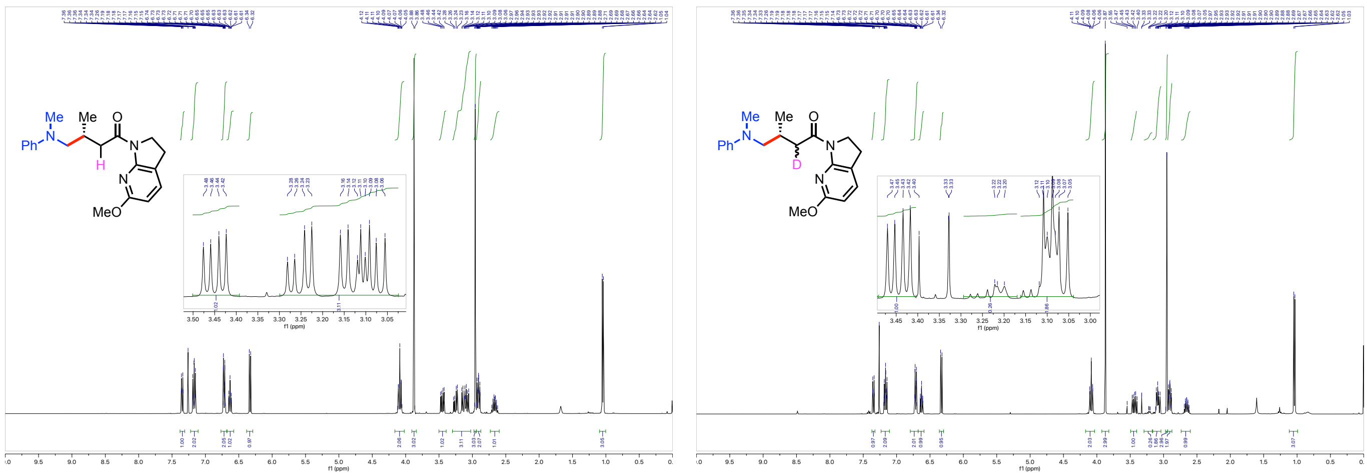

Fig S5: ^{1}H-NMR spectra of **3da** (left) and **3da-*d*** (right)

9. Copper complexation experiments

The coordination aptitude of **Cu(MeCN)₄PF₆** towards the 7-azaindoline derivative (**1j**) and 6-MeO-7-azaindoline derivative (**1m**) was separately checked by ^{1}H-NMR in MeCN-*d₃* in the presence of both chiral ligands [**L2** = (*R*)-DM-Segphos and **L3** = (*R*)-DM-BINAP]. At first, the separate NMR spectras of **1j**, **1m**, **L2**, **L3**, and their respective copper complexes were recorded. Later, a separate NMR spectras of copper/ligand complex and substrates (**1j** and **1m**) were recorded. Finally, the mixture of **1j** (1 equiv) and **1m** (1 equiv) was mixed with the copper complex (1 equiv). Surprisingly, the free substrate (either **1j** or **1m**) could not be detected, thus both the substrates were detected in a complexed form. This confirms the fast-complex exchange between **1j** and **1m**. Apparently, it also suggests that the -OMe group is not affecting (sterically) towards the efficient complexation.

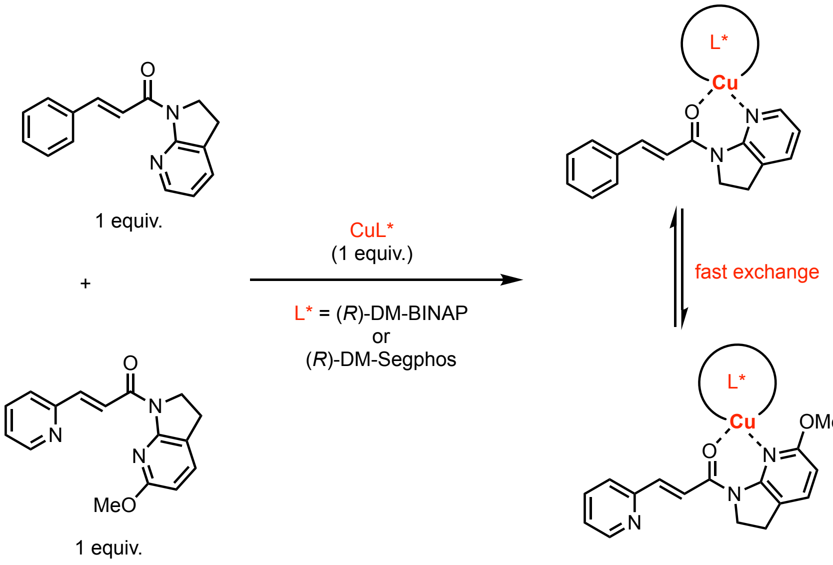

^{1}H NMR: **(*R*)-DM-BINAP** in MeCN-*d₃*

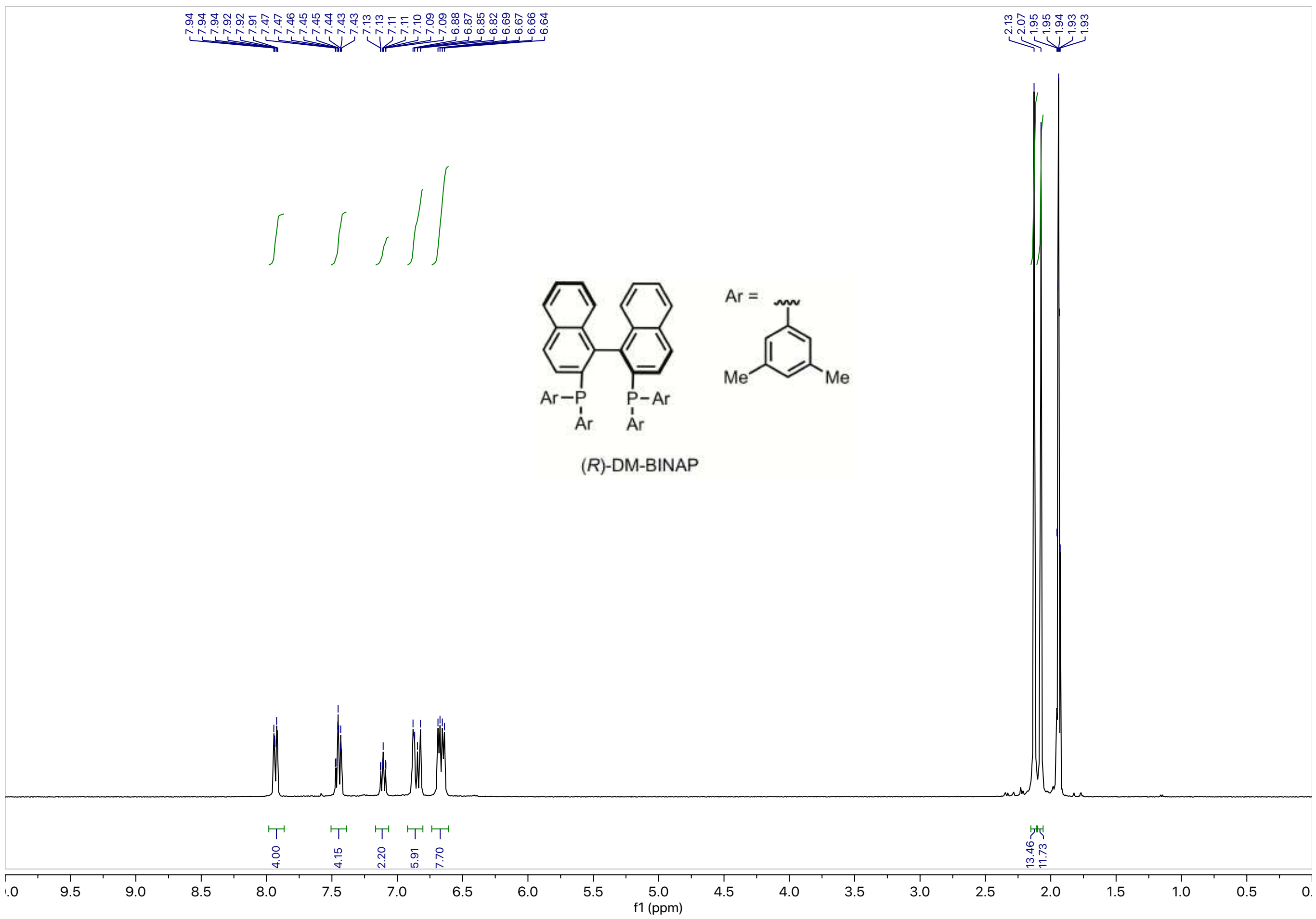

^{1}H NMR: **(*R*)-DM-BINAP** (1 equiv) **+ Cu(MeCN)$_4$PF$_6$** (1 equiv) in MeCN-d_3

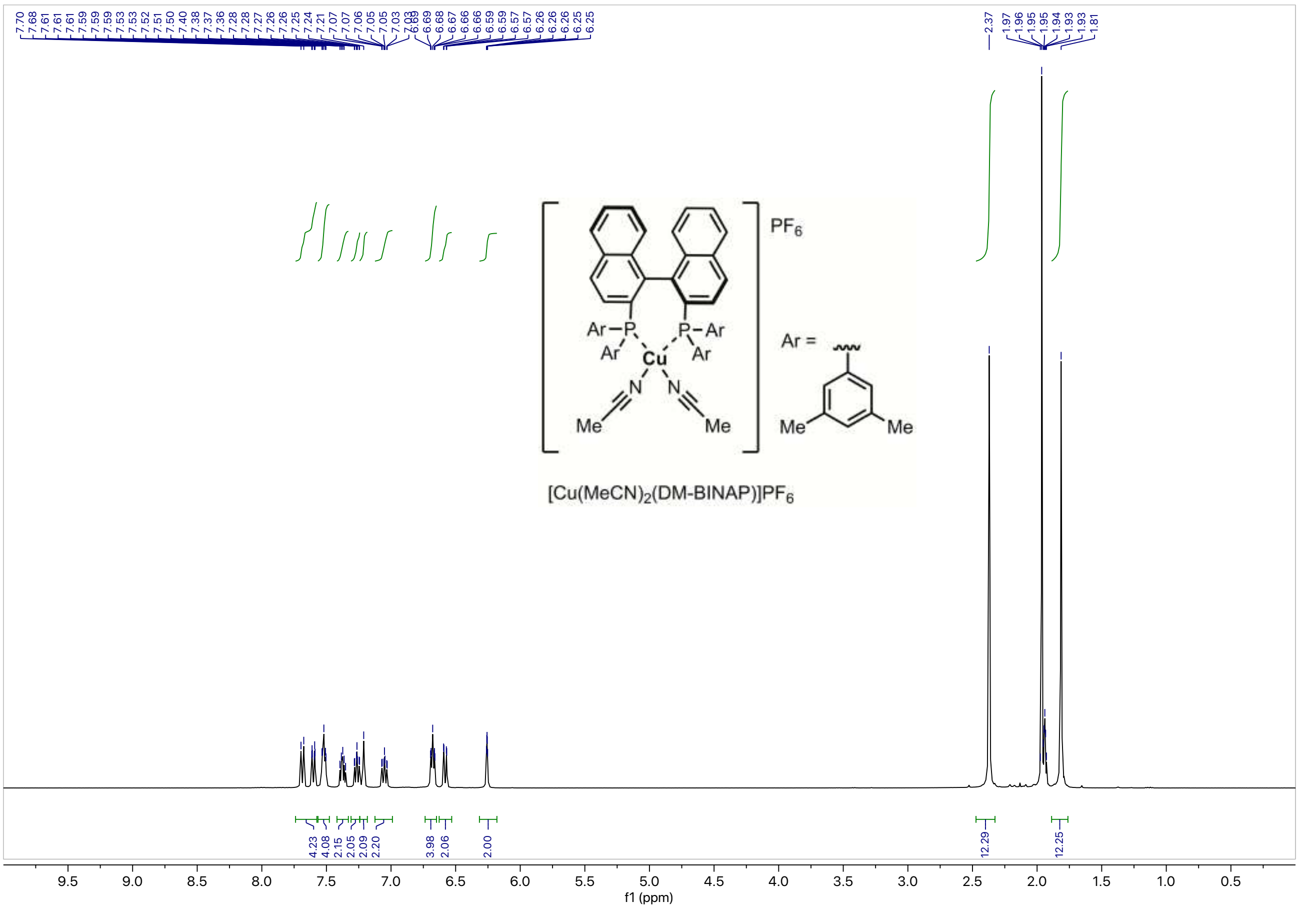

^{1}H NMR: **1j** in MeCN-d_3

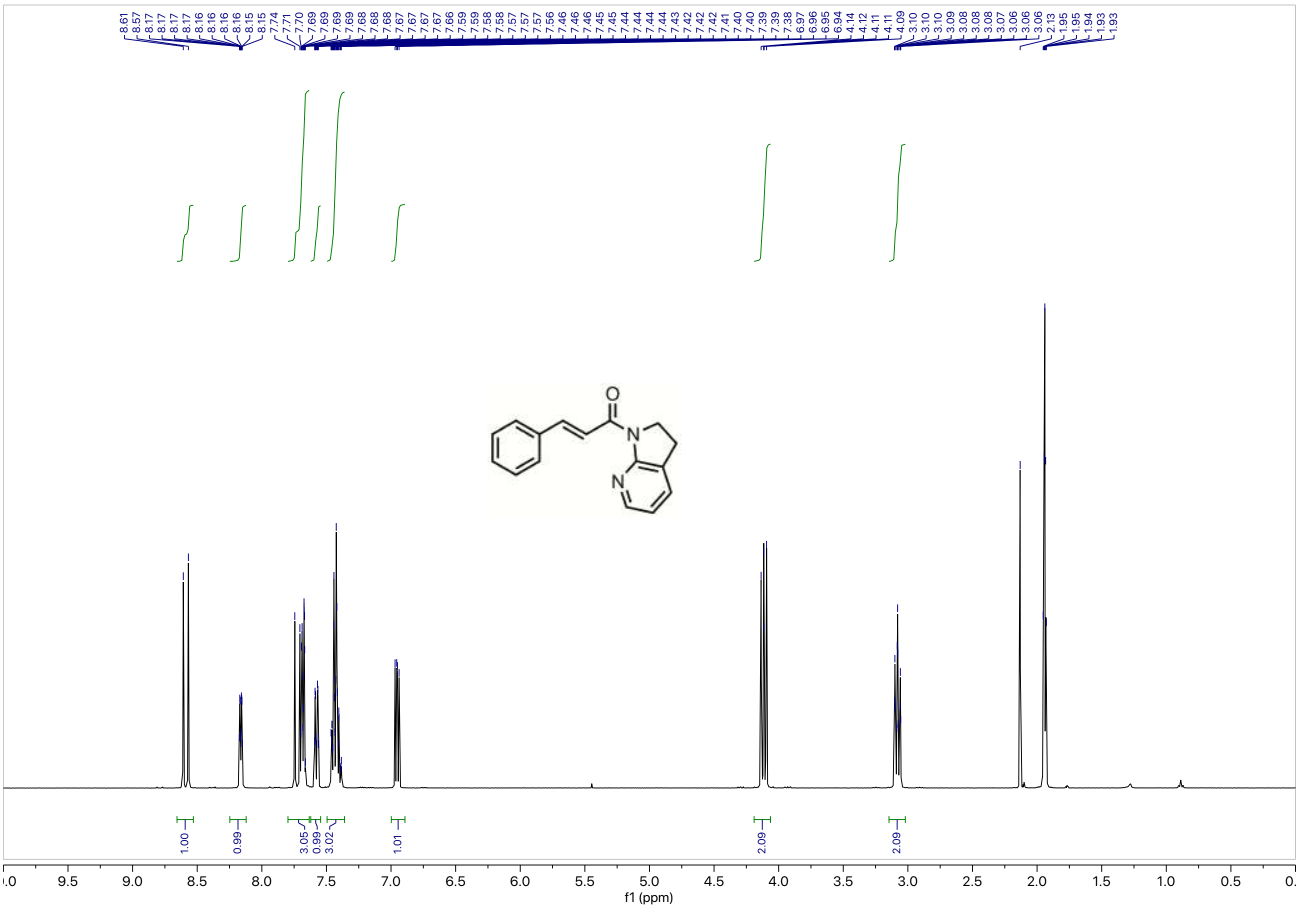

^{1}H NMR: **(*R*)-DM-BINAP** (1 equiv) + **Cu(MeCN)$_4$PF$_6$** (1 equiv) + **1j** (1 equiv) in MeCN-*d*$_3$

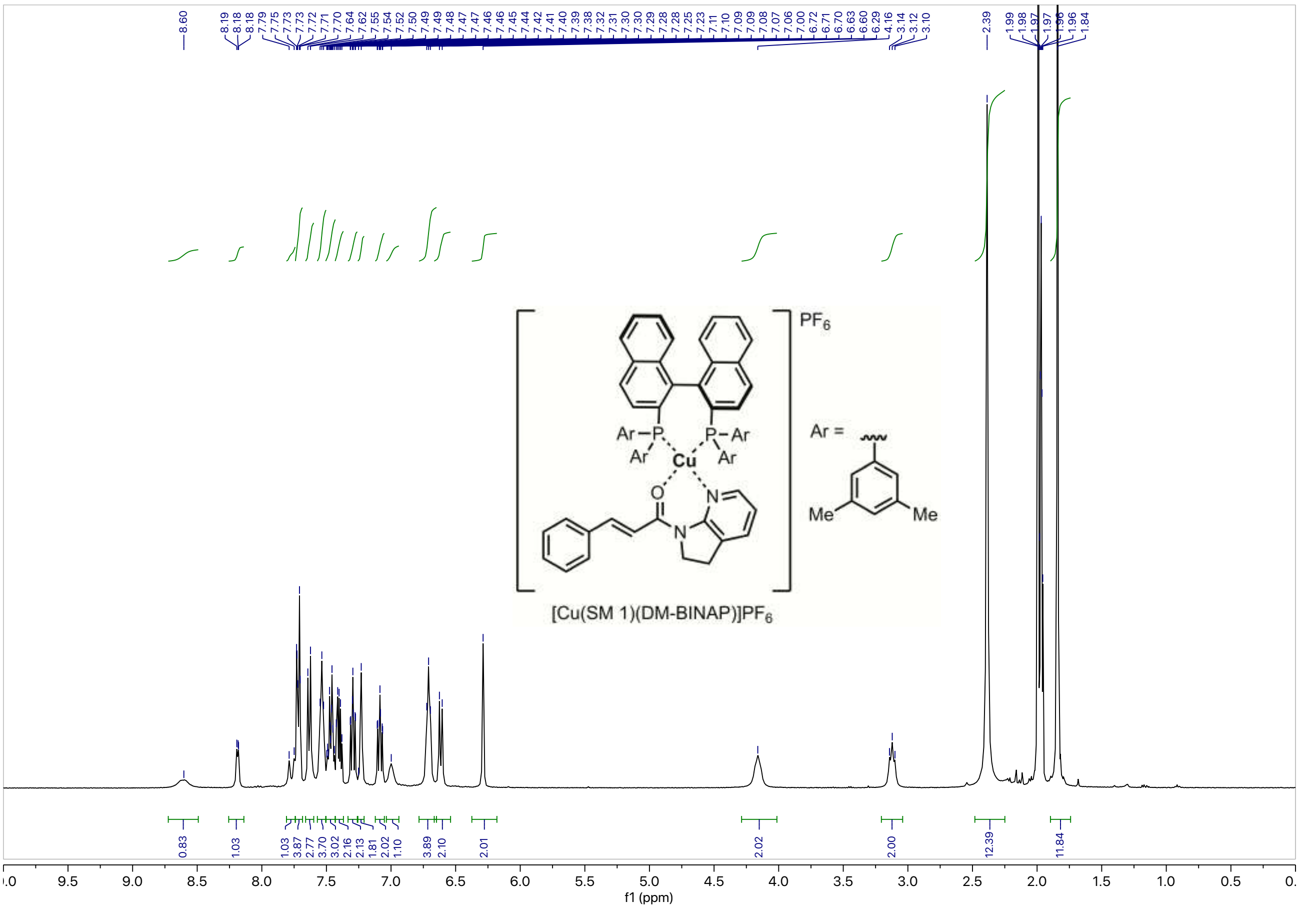

^{1}H NMR: **1m** in MeCN-*d*$_3$

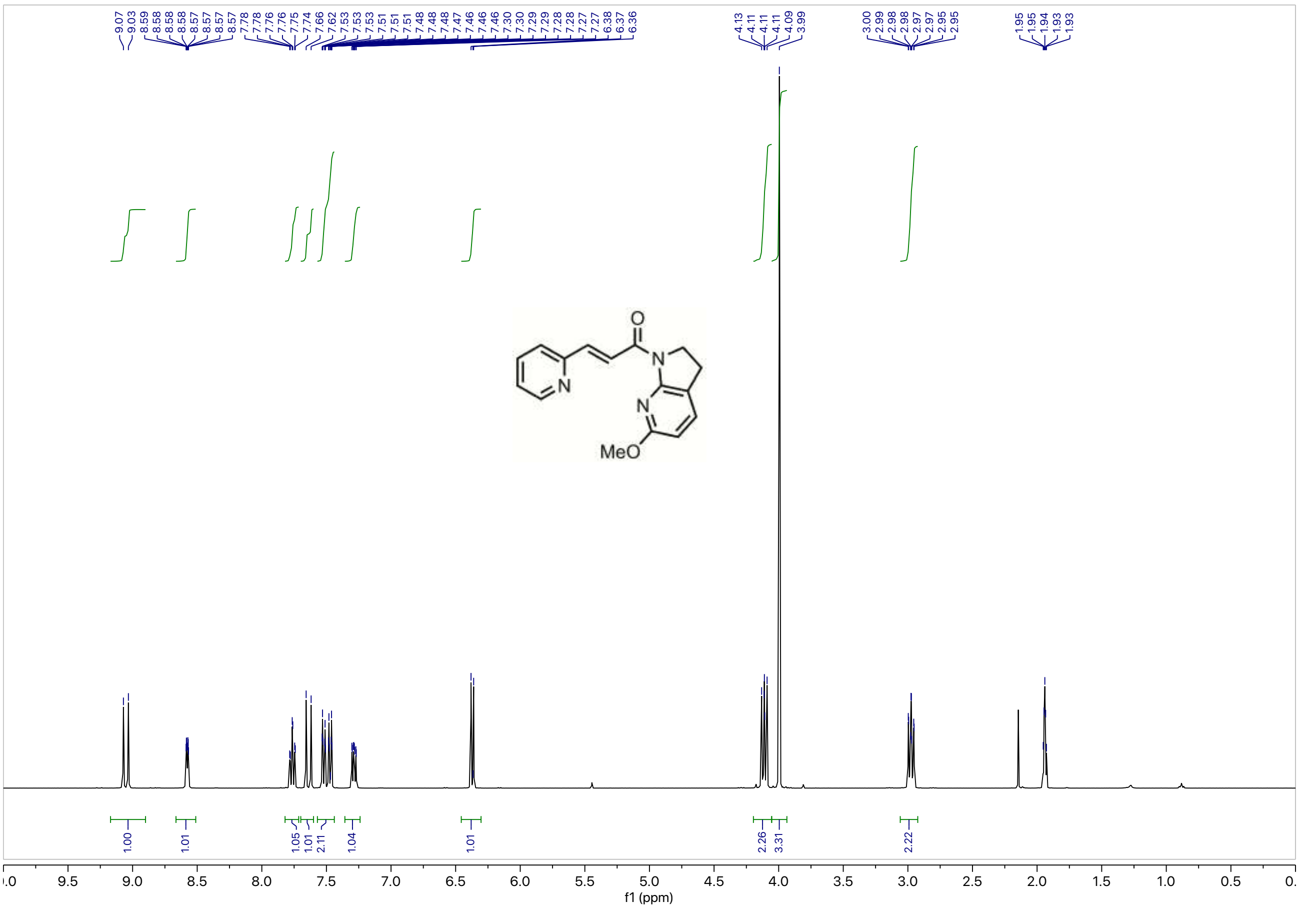

^{1}H NMR: **(*R*)-DM-BINAP** (1 equiv) **+ Cu(MeCN)$_4$PF$_6$** (1 equiv) + **1m** (1 equiv) in MeCN-*d*$_3$

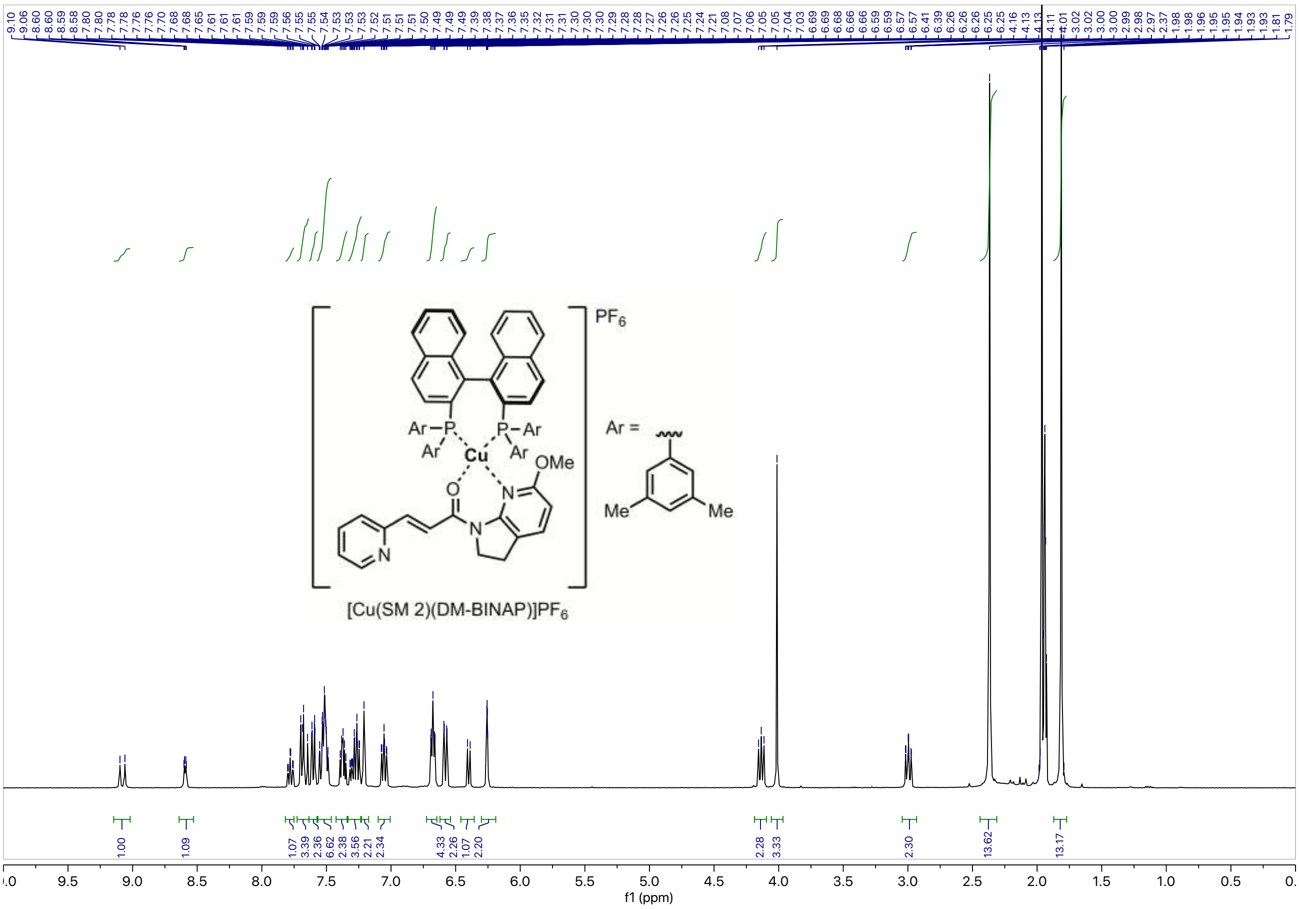

^{1}H NMR: **(*R*)-DM-BINAP** (1 equiv) **+ Cu(MeCN)$_4$PF$_6$** (1 equiv) + **1j** (1 equiv) + **1m** (1 equiv) in MeCN-*d*$_3$

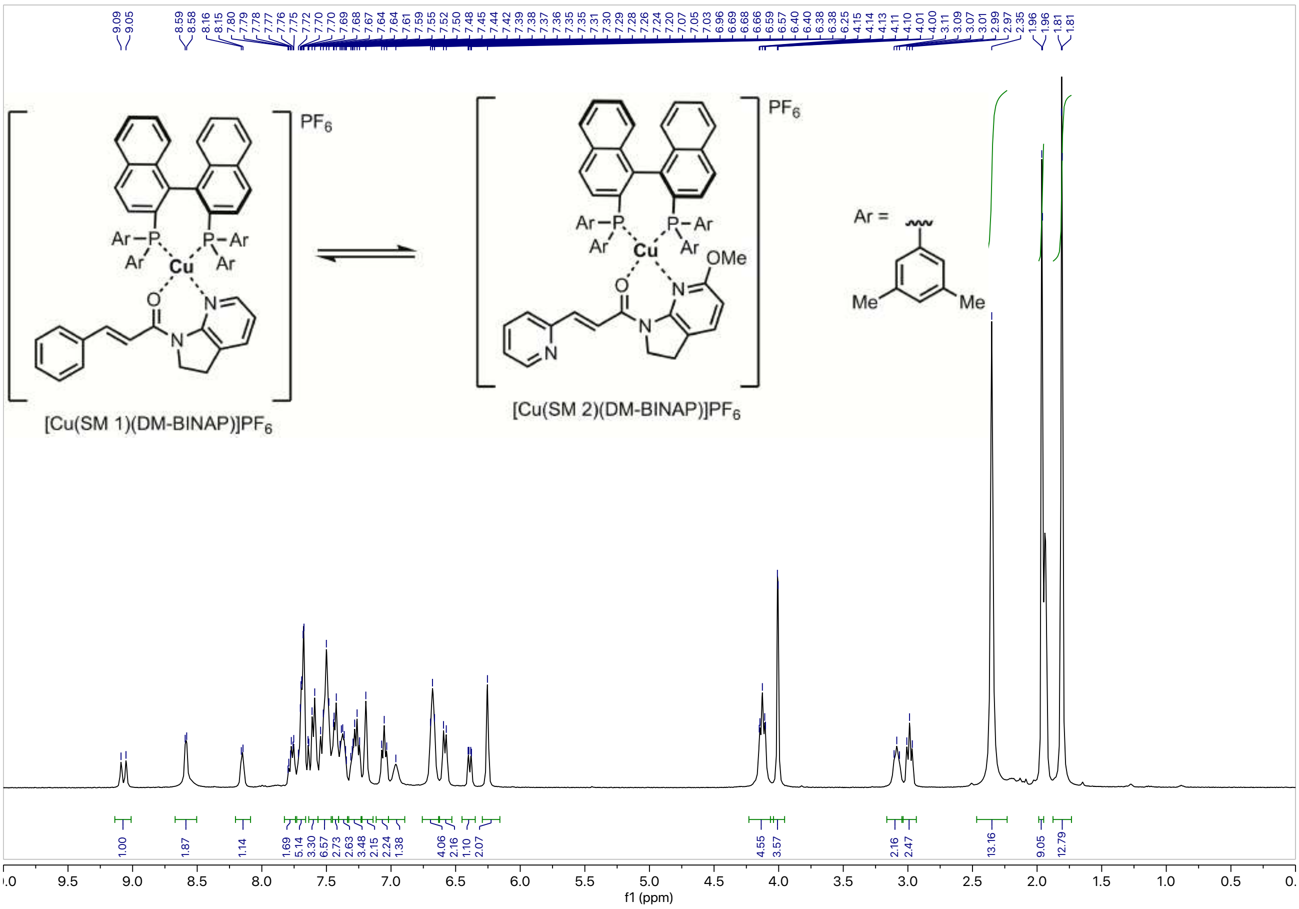

^{1}H NMR: ***(R)*-DM-Segphos** in MeCN-*d*$_3$

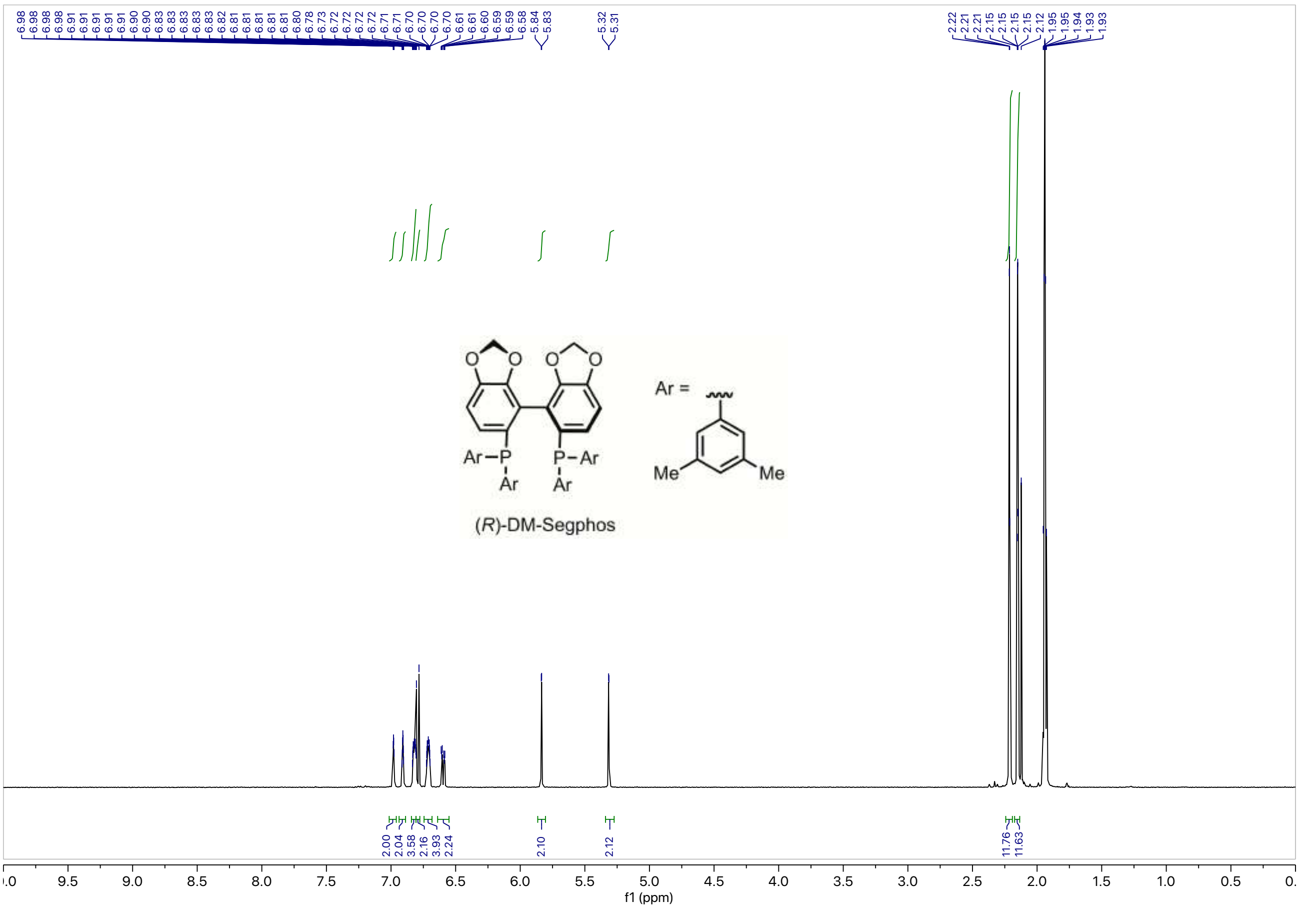

^{1}H NMR: ***(R)*-DM-Segphos** (1 equiv) **+ Cu(MeCN)$_4$PF$_6$** (1 equiv)

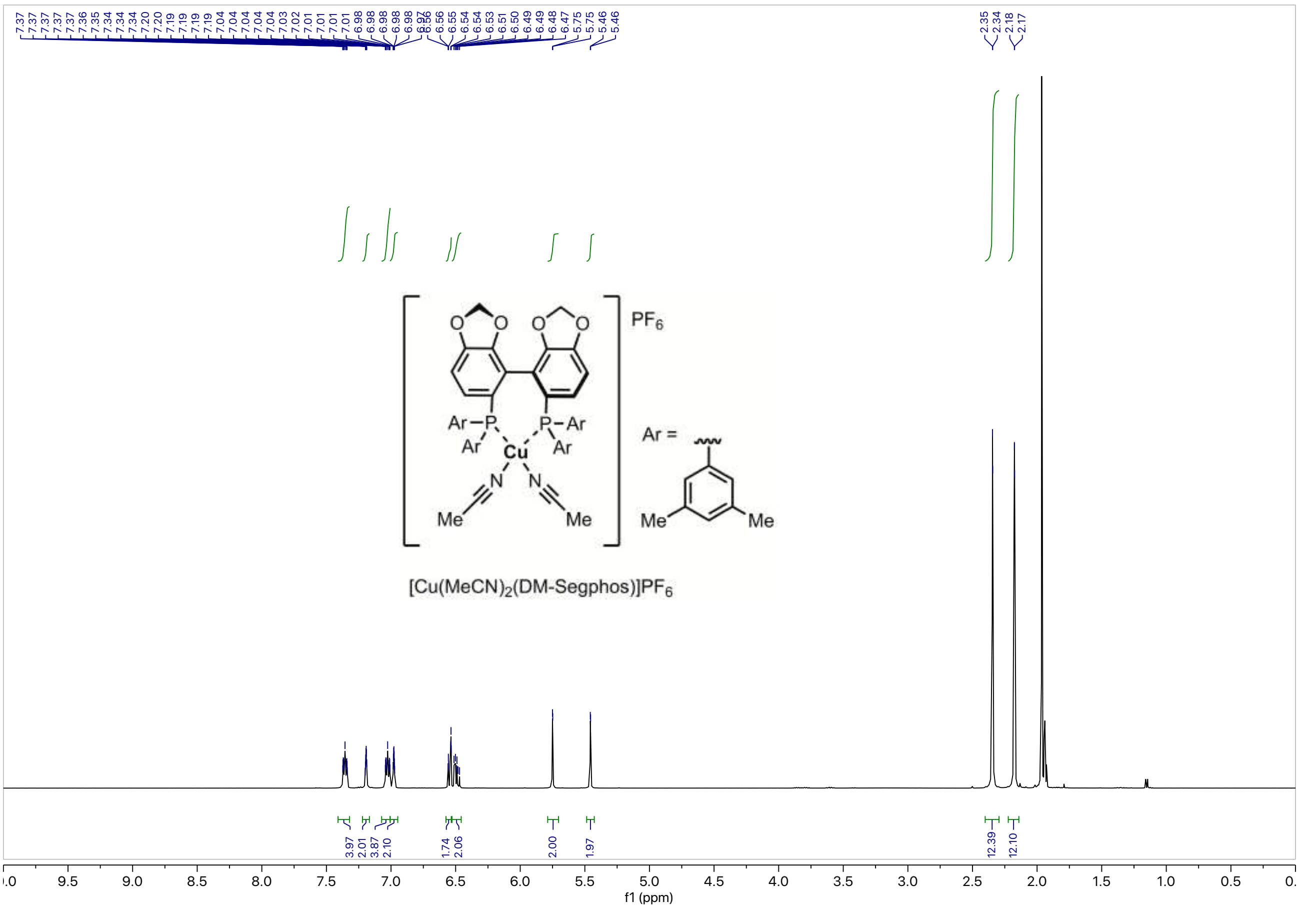

^{1}H NMR: **(*R*)-DM-Segphos** (1 equiv) **+ $Cu(MeCN)_4PF_6$** (1 equiv) + **1j** (1 equiv) in MeCN-*d*$_3$

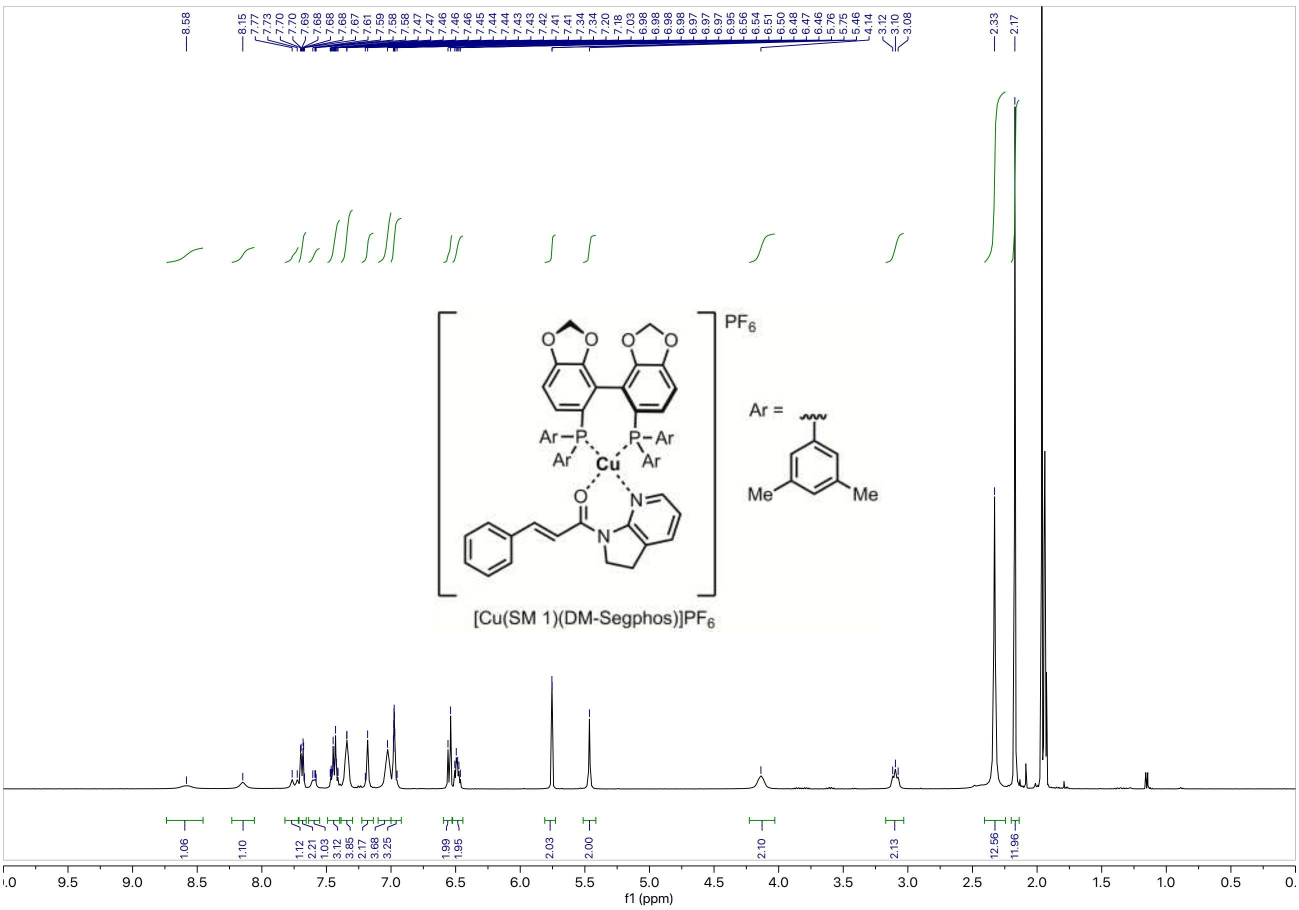

^{1}H NMR: **(*R*)-DM-Segphos** (1 equiv) **+ $Cu(MeCN)_4PF_6$** (1 equiv) + **1m** (1 equiv) in MeCN-*d*$_3$

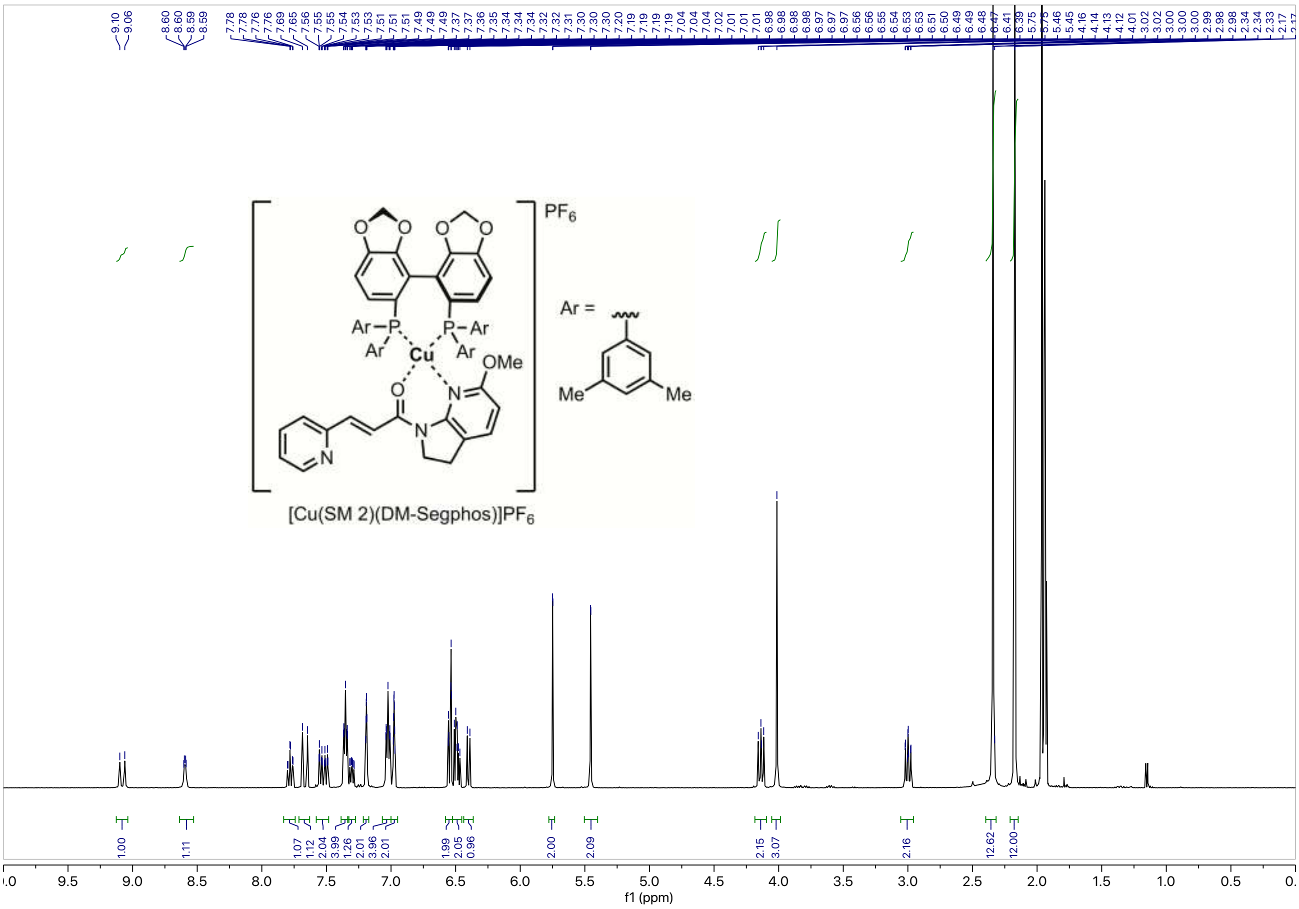

^{1}H NMR: ***(R)*-DM-Segphos** (1 equiv) **+ Cu(MeCN)$_4$PF$_6$** (1 equiv) + **1j** (1 equiv) + **1m** (1 equiv) in MeCN-*d*$_3$

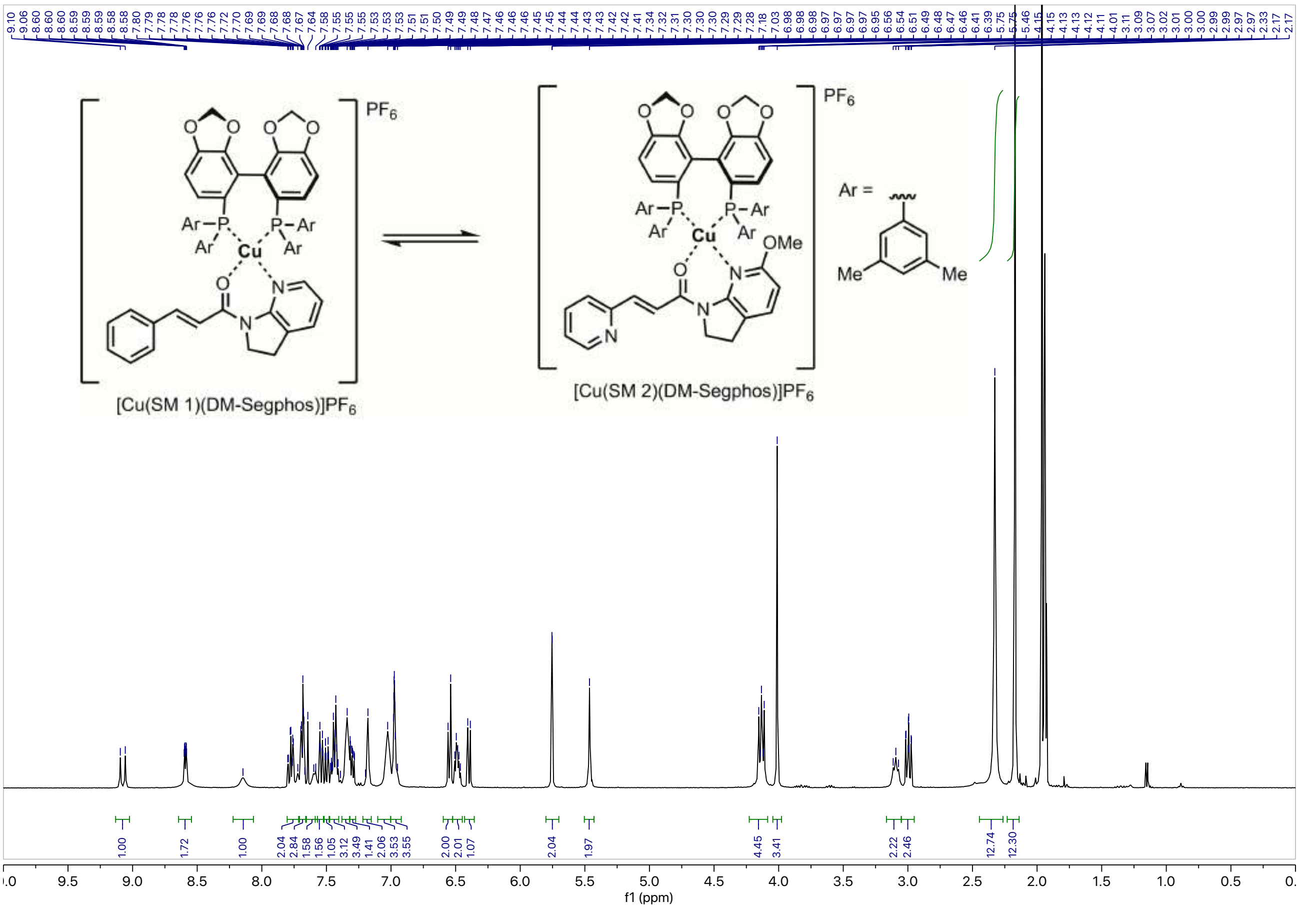

10. Crystal Structures:

*10.1. Solid state structure of **1w***

Single crystals of ***1s*** were obtained by vapor diffusion from DCM/hexane at RT. A suitable crystal was selected and the sample was measured on a Rigaku R-AXIS RAPIS II diffractometer using multi-layer mirror monochromated Cu-Kα radiation (λ = 1.54184). The data were collected at 93 K. Refined structure and crystallographic parameters are summarized in Table S1 and Fig. S6. **CCDC 1985034** contains the supplementary crystallographic data for ***1w***.

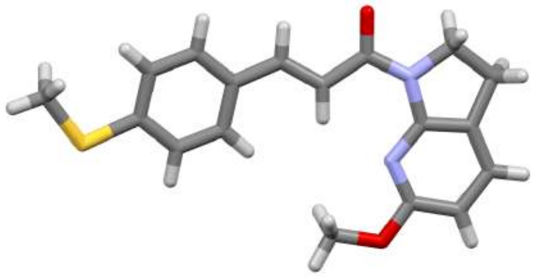

Figure S6. Structure of ***1w*** in the solid state. Color code: red: oxygen; light blue: nitrogen; gray: carbon; white: hydrogen; yellow: sulfur

Table S1. Selected crystal data of ***1w*.**

Empirical Formula		$C_{18}H_{18}N_2O_2S$
Formula Weight		326.40
Crystal Color, Habit		yellow, platelet
Crystal Dimensions		0.300 x 0.100 x 0.100 mm
Crystal System		monoclinic
Space group		$P2_1/n$
Lattice Parameters		
	a	6.60950(10) Å
	b	17.7870(2) Å
	c	14.05220(10) Å
	V	1609.03(3) Å^3
Z value		4
R_1		0.0667
wR_2		0.1739
D_{calc}		1.347 g/cm^3
F_{000}		688.00

*10.2. Solid state structure of **3dd**: (determination of absolute configuration)*

Single crystals of ***3dd*** were obtained by vapor diffusion from DCM/hexane at RT. A suitable crystal was selected and the sample was measured on a Rigaku R-AXIS RAPIS II diffractometer using multi-layer mirror monochromated Cu-Kα radiation (λ = 1.54184). The data were collected at 93 K. Refined structure and crystallographic parameters are summarized in Table S2 and Fig. S7. **CCDC 1985035** contains the supplementary crystallographic data for ***3dd***.

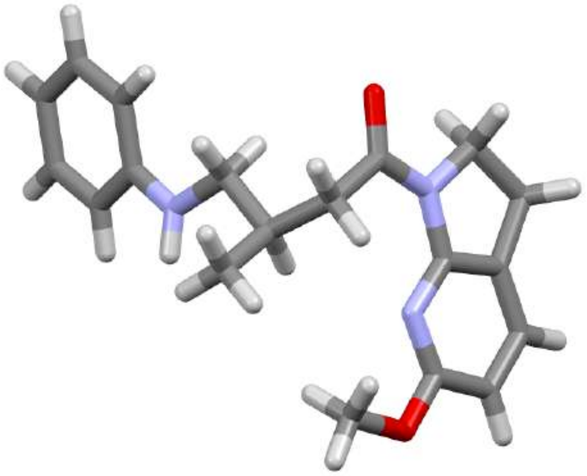

Figure S7. Structure of ***3dd*** in the solid state. Color code: red: oxygen; light blue: nitrogen; gray: carbon; white: hydrogen.

Table S2. Selected crystal data of ***3dd*.**

Empirical Formula	$C_{38}H_{46}N_6O_4$
Formula Weight	650.81
Crystal Color, Habit	colorless, needles
Crystal Dimensions	0.300 x 0.100 x 0.008 mm
Crystal System	triclinic
Space group	P1
Lattice Parameters	
a	6.05710(10) Å
b	10.16470(10) Å
c	14.3715(2) Å
V	827.01(2) Å^3
Z value	1
R_1	0.0368
wR_2	0.0966
D_{calc}	1.307 g/cm^3
F_{000}	348.00
Flack parameter	-0.02(10)

*10.3. Solid state structure of **C1***

Single crystals of ***C1*** were obtained by vapor diffusion from THF/hexane at RT. A suitable crystal was selected and the sample was measured on a Rigaku R-AXIS RAPIS II diffractometer using multi-layer mirror monochromated Cu-Kα radiation (λ = 1.54184). The data were collected at 93 K. Refined structure and crystallographic parameters are summarized in Table S3 and Fig. S8. **CCDC 1985036** contains the supplementary crystallographic data for ***C1***.

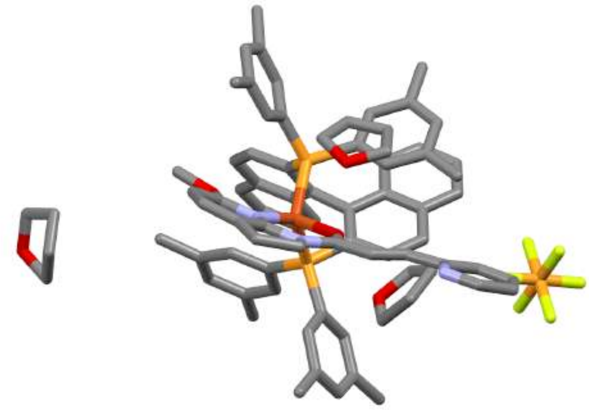

Figure S8. Structure of ***C1*** in the solid state. Color code: red: oxygen; light blue: nitrogen; gray: carbon; dark yellow: Phosphorous; orange: copper; light green: fluorine.
Note: the crystal was surrounded by THF, which was used as a solvent for the crystallization.

Table S3. Selected crystal data of ***C1*.**

Empirical Formula		$C_{80}H_{87}CuF_6N_3O_5P_3$
Formula Weight		1440.97
Crystal Color, Habit		yellow, platelet
Crystal Dimensions		0.200 x 0.100 x 0.008 mm
Crystal System		monoclinic
Space Group		*P2₁/c*
Lattice Parameters		
	a	21.7889(4) Å
	b	16.4491(4) Å
	c	24.0629(5) Å
	V	8269.0(3) Å^3
Z value		4
R_1		0.1027
wR_2		0.2118
D_{calc}		1.157 g/cm^3
F_{000}		3024.00

11. References

1. For the synthesis of 7-azaindoline derivatives, see:
(a) Brewitz, L.; Noda, H.; Kumagai, N.; Shibasaki, M. *Eur. J. Org. Chem.* **2018**, 714–722.
(b) Ito, M.; Inoue, D.; Kawasaki, R.; Kanyiva, K. S.; Shibata, T., *Heterocycles*, **2017**, *94*, 2229–2246.
(c) Hogg, J. H.; Kester, R. F.; Liang, W.; Yun, W. *WO 2014056871 A1* (Patent).
(d) Norris, D. J.; Vaccaro, W.; Debenedetto, M. V.; Degnan, A. P.; Delucca, G. V.; Deskus, J. A.; Han, W.-C.; Kumi, G. K.; Schmitz, W. D.; Starrett, J. E., Jr.; Hill, M. D.; Huang, H. *US 20160333013* A1 (Patent).
(e) Tung, Y. -S.; Coumar, M. S.; Wu, Y. -S.; Shiao, H. -Y.; Chang, J. -Y.; Liou, J. -P.; Shukla, P.; Chang, C. -W.; Chang, C. -Y.; Kuo, C. -C.; Yeh, T.-K.; Lin, C. -Y.; Wu, J. -S.; Wu, S. -Y.; Liao, C. -C.; Hsieh, H. -P. *J. Med. Chem.* **2011**, *54*, 3076-3080.
(f) Alternate method for the indole reduction, see: Tan, M.; Zhang, Y. *Tetrahedron Lett*. **2009**, *50*, 4912-4915.

2. For the synthesis of α,β-unsaturated 7-azaindoline derivatives, see:
(a) Zhang, M.; Kumagai, N.; Shibasaki, M. *Chem.Eur.J.* **2017**, *23*, 12450–12455.
(b) Zhang, M.; Kumagai, N.; Shibasaki, M. *Chem.Eur.J.* **2016**, *22*, 5525–5529.

3. For the synthesis of Amine derivatives, see:
(a) Espelt, L. R.; McPherson, I. S.; Wiensch, E. M.; Yoon T. P.; *J. Am. Chem. Soc.* **2015**, *137*, 2452–2455.
(b) Nakajima, K.; Kitagawa, M.; Ashida, Y.; Miyake, Y.; Nishibayashi, Y. *Chem. Commun.,* **2014**, *50,* 8900-8903.

12. NMR Spectra:

^{1}H NMR: **1a** (Note: *unless otherwise stated, all the NMR spectras were recorded in CDCl*$_3$).

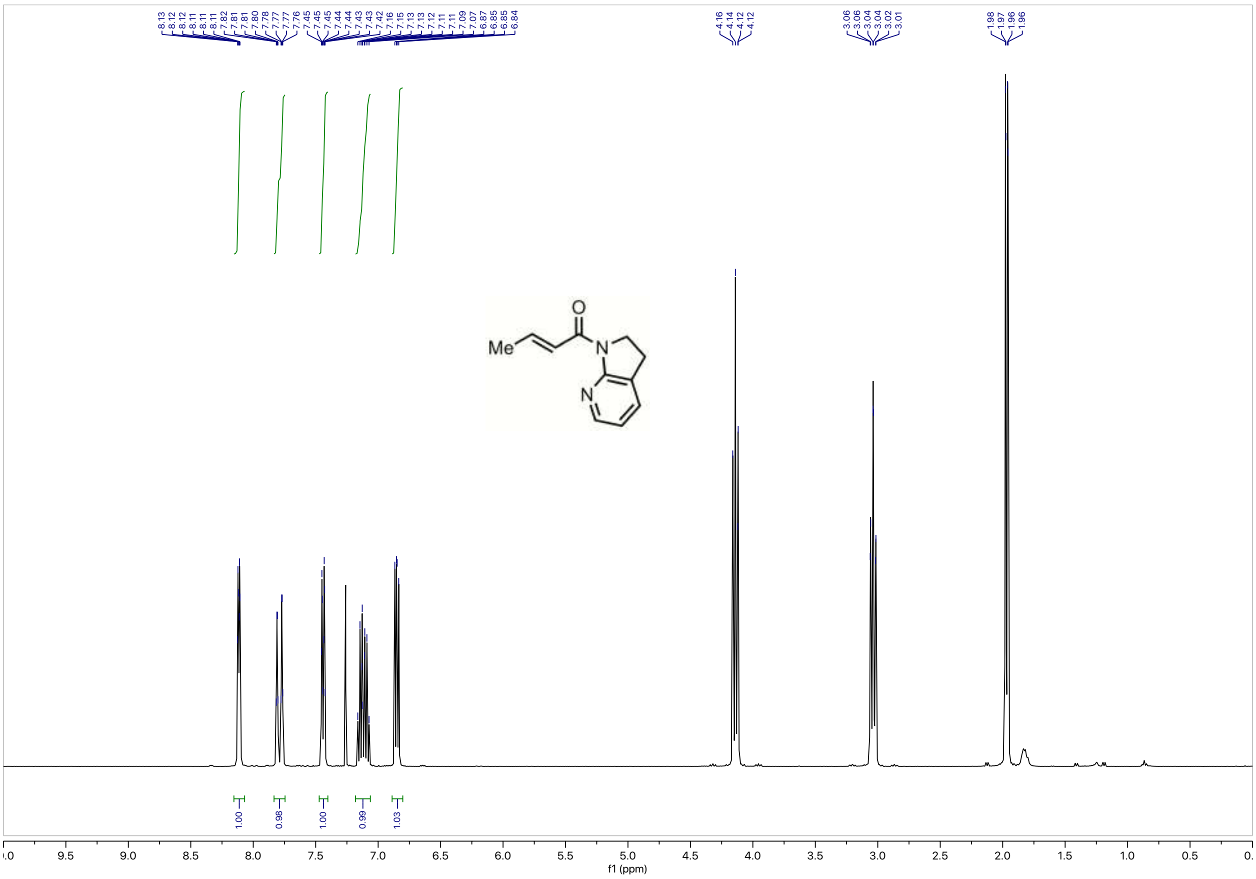

^{13}C NMR: **1a**

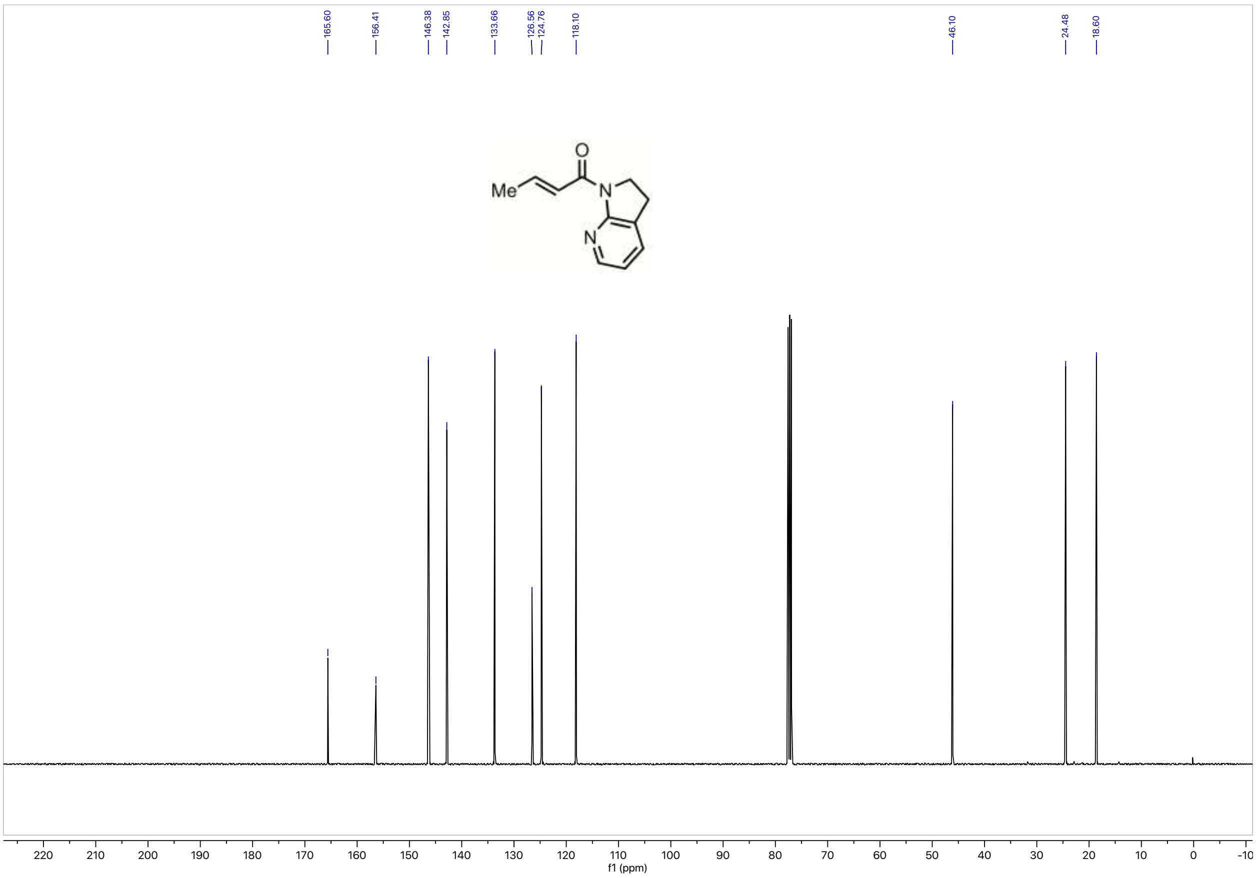

^{1}H NMR: **1b**

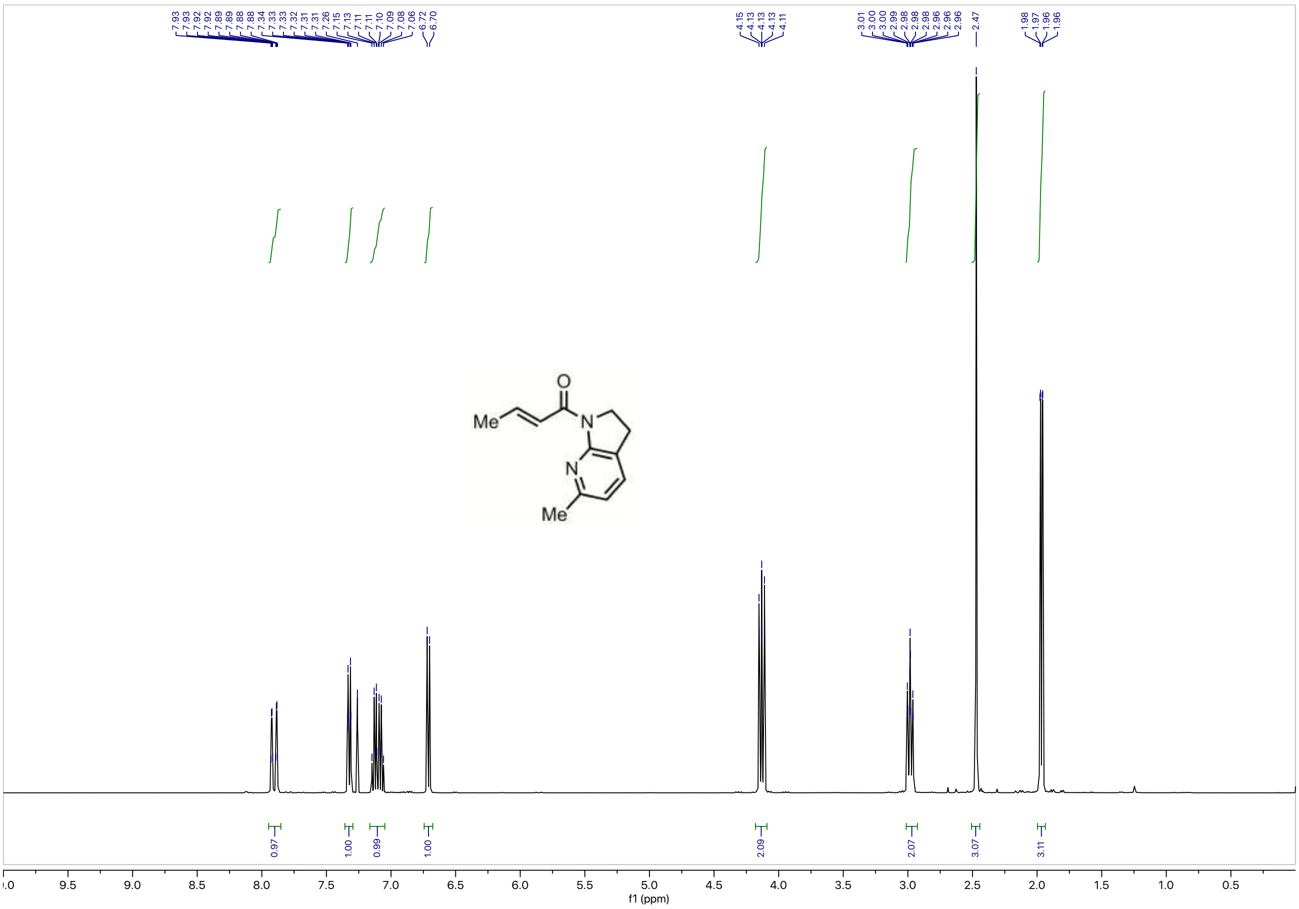

^{13}C NMR: **1b**

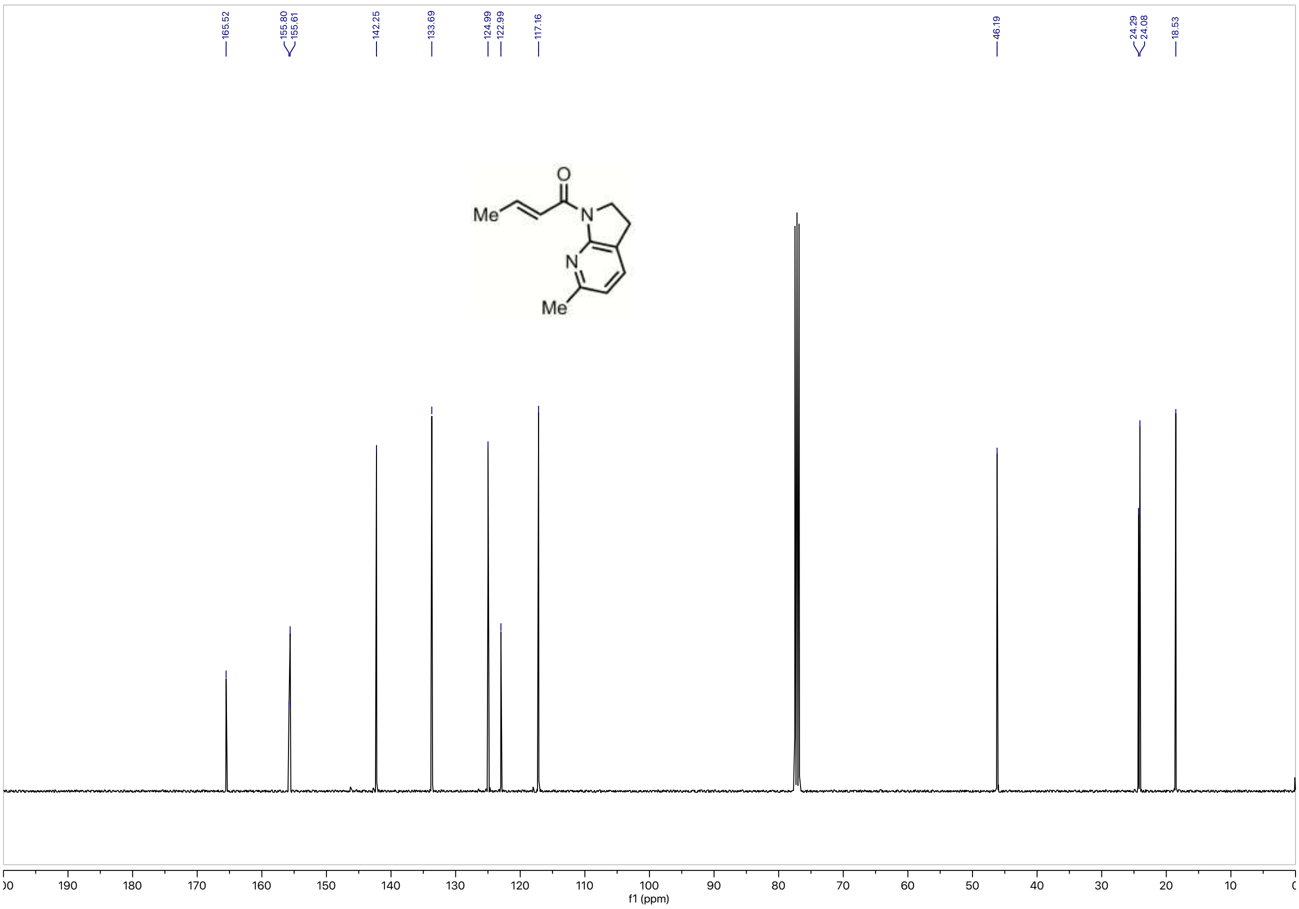

^{1}H NMR: **1c**

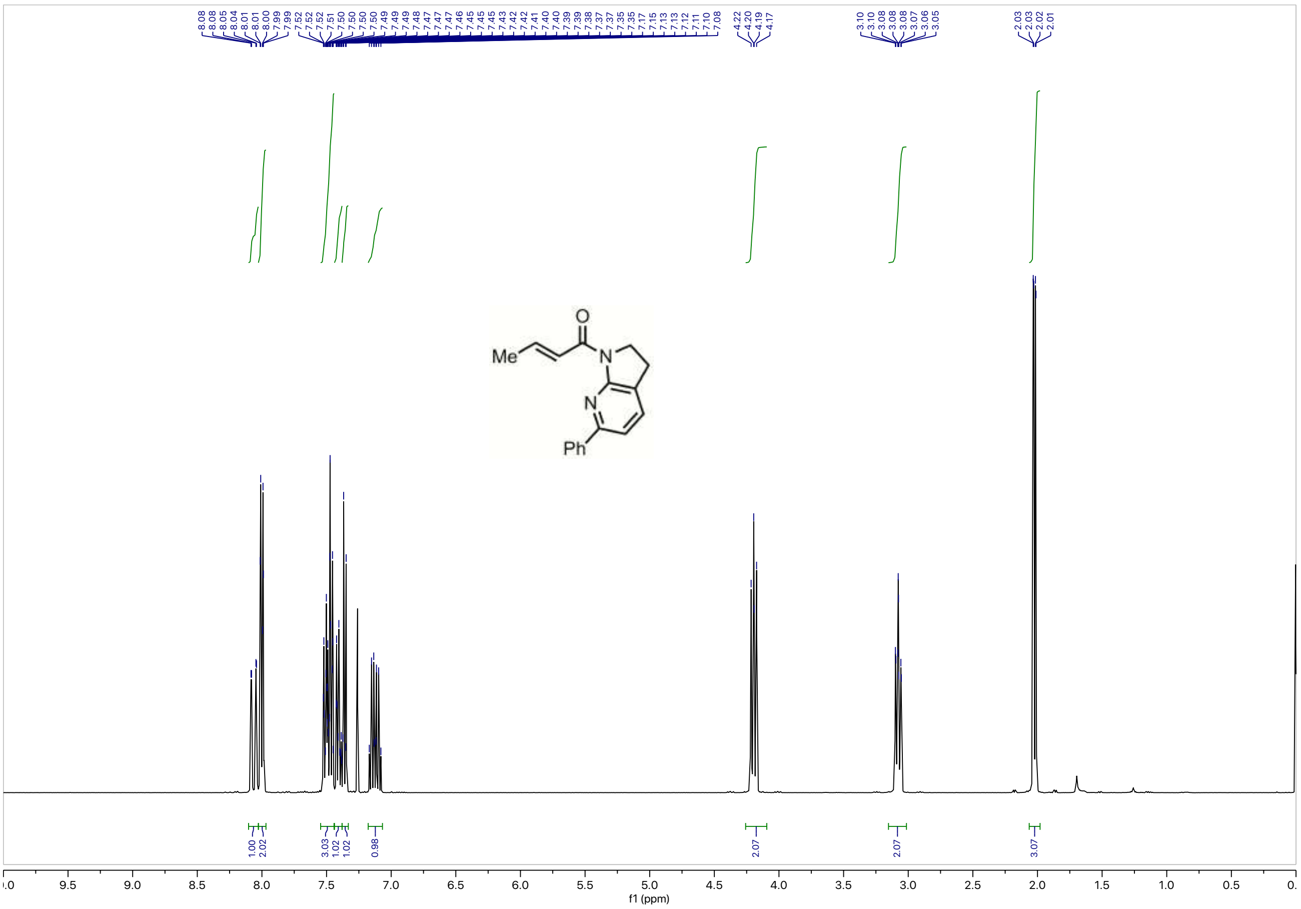

^{13}C NMR: **1c**

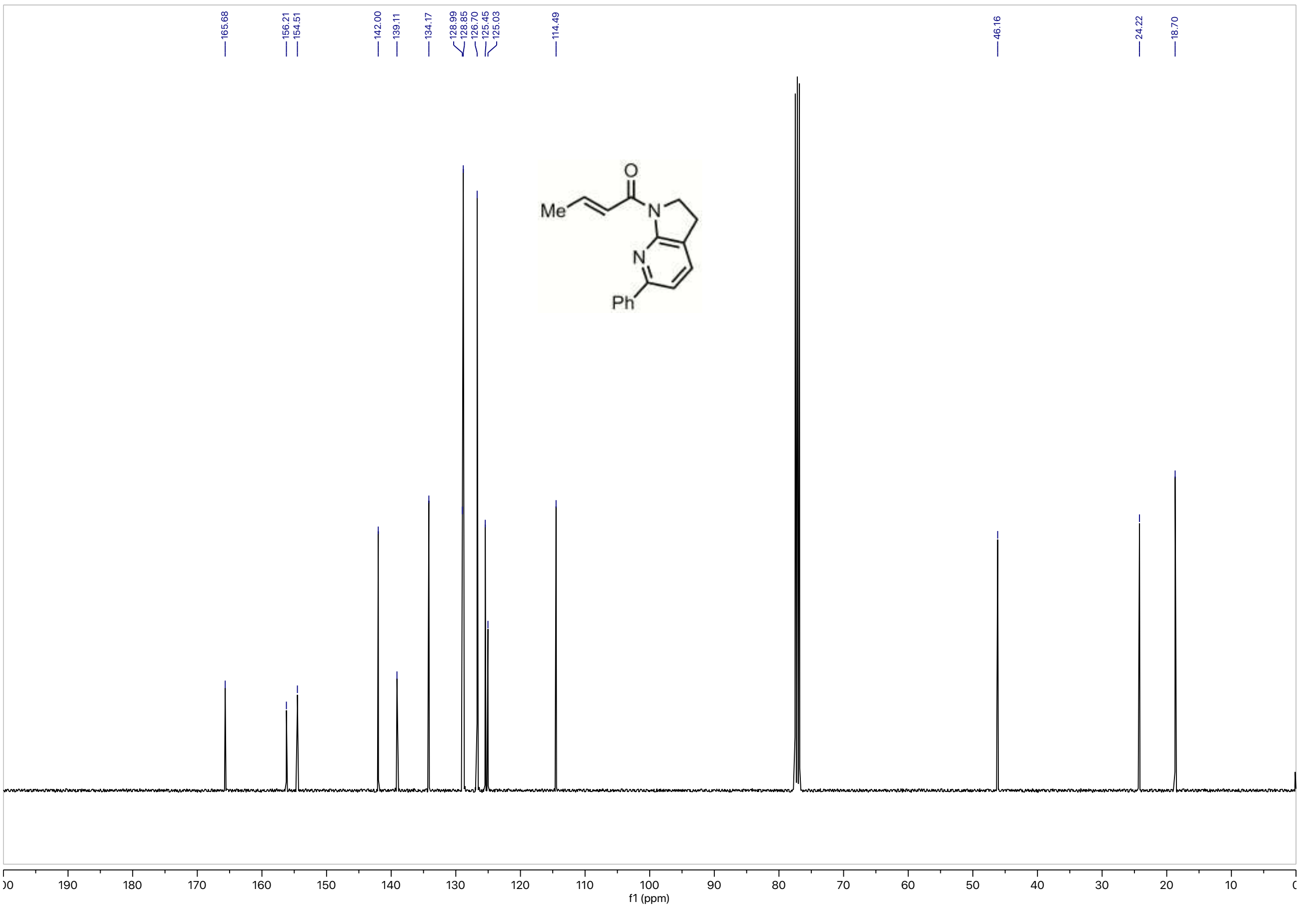

^{1}H NMR: **1d**

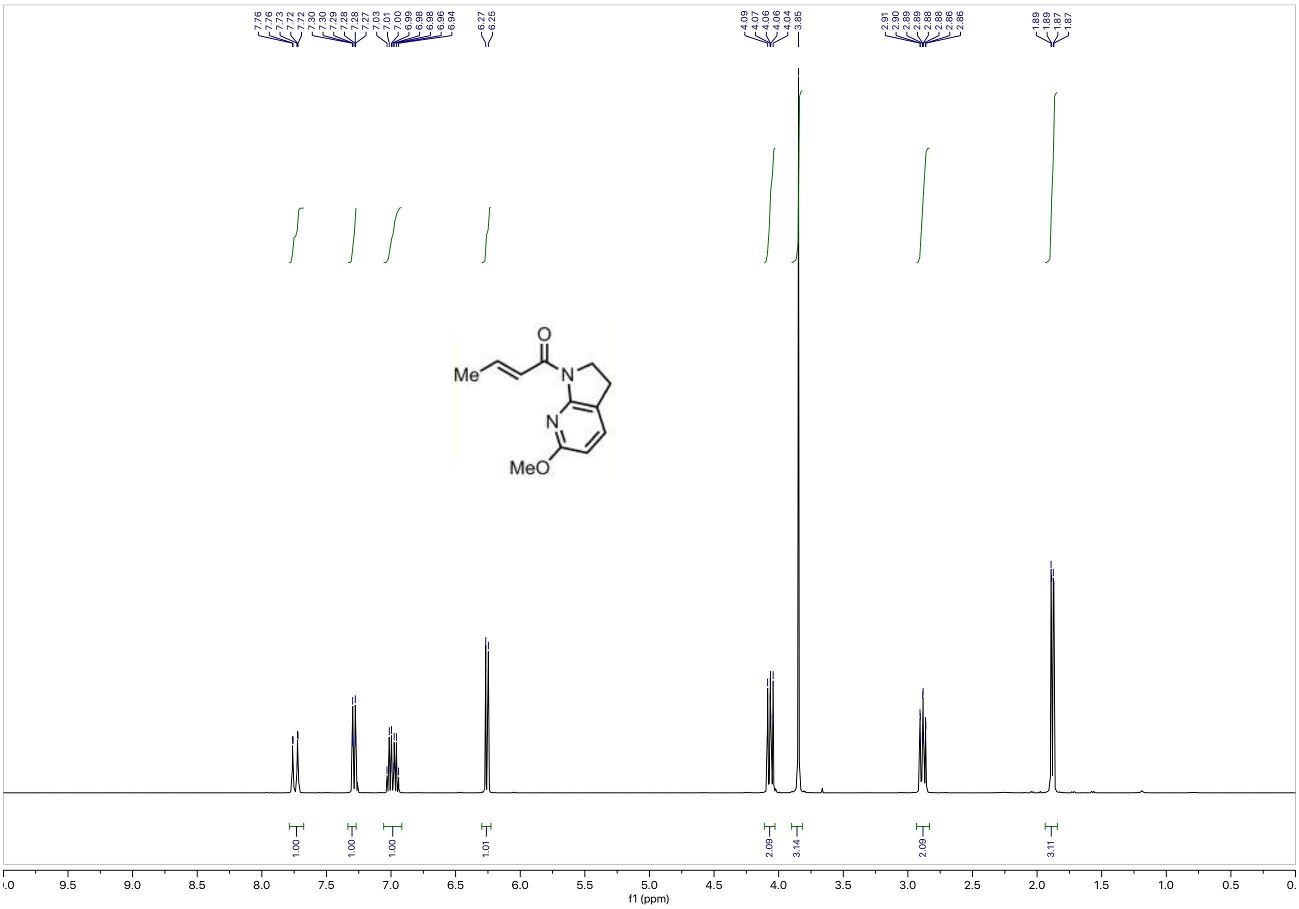

^{13}C NMR: **1d**

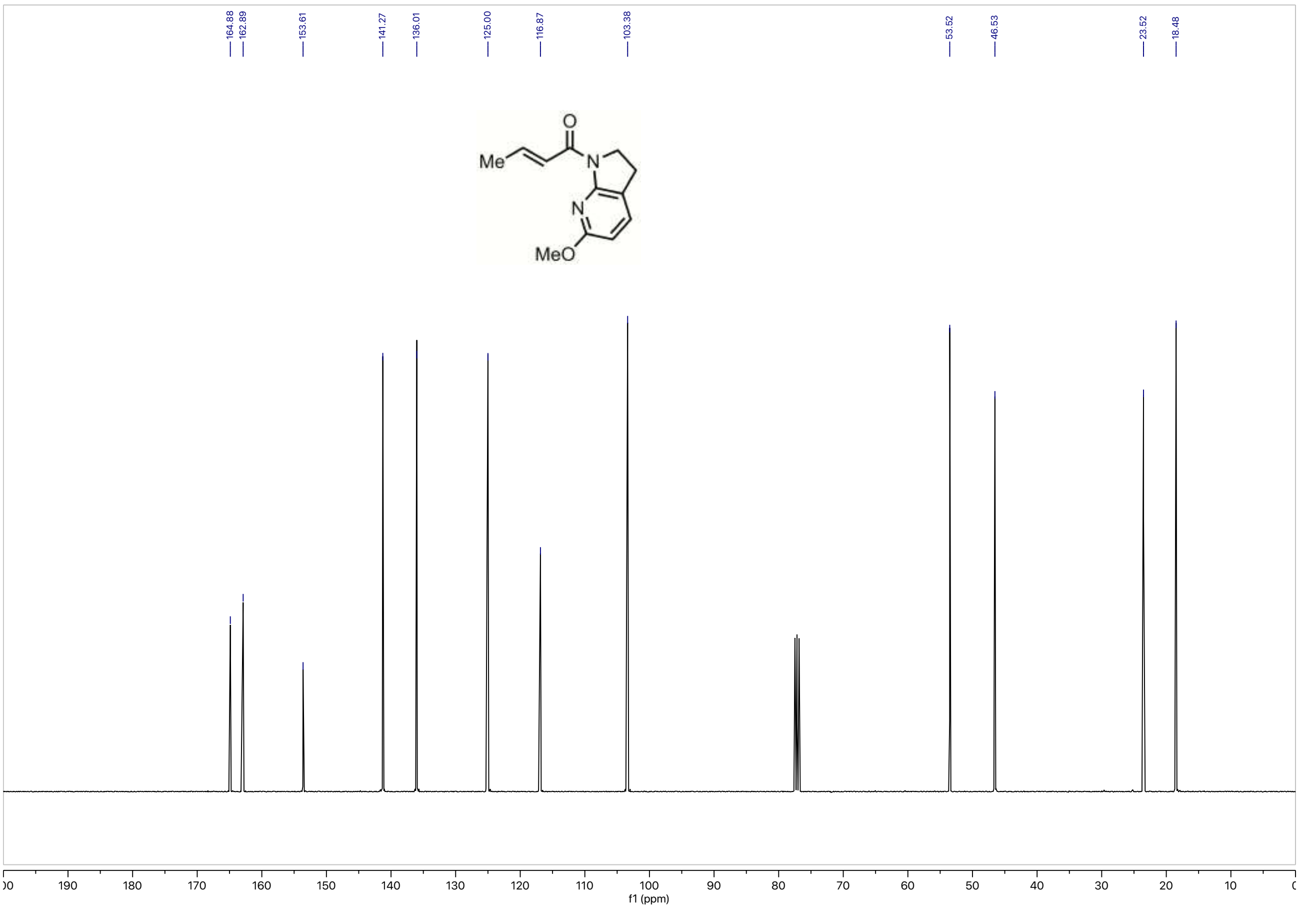

^{1}H NMR: **1e**

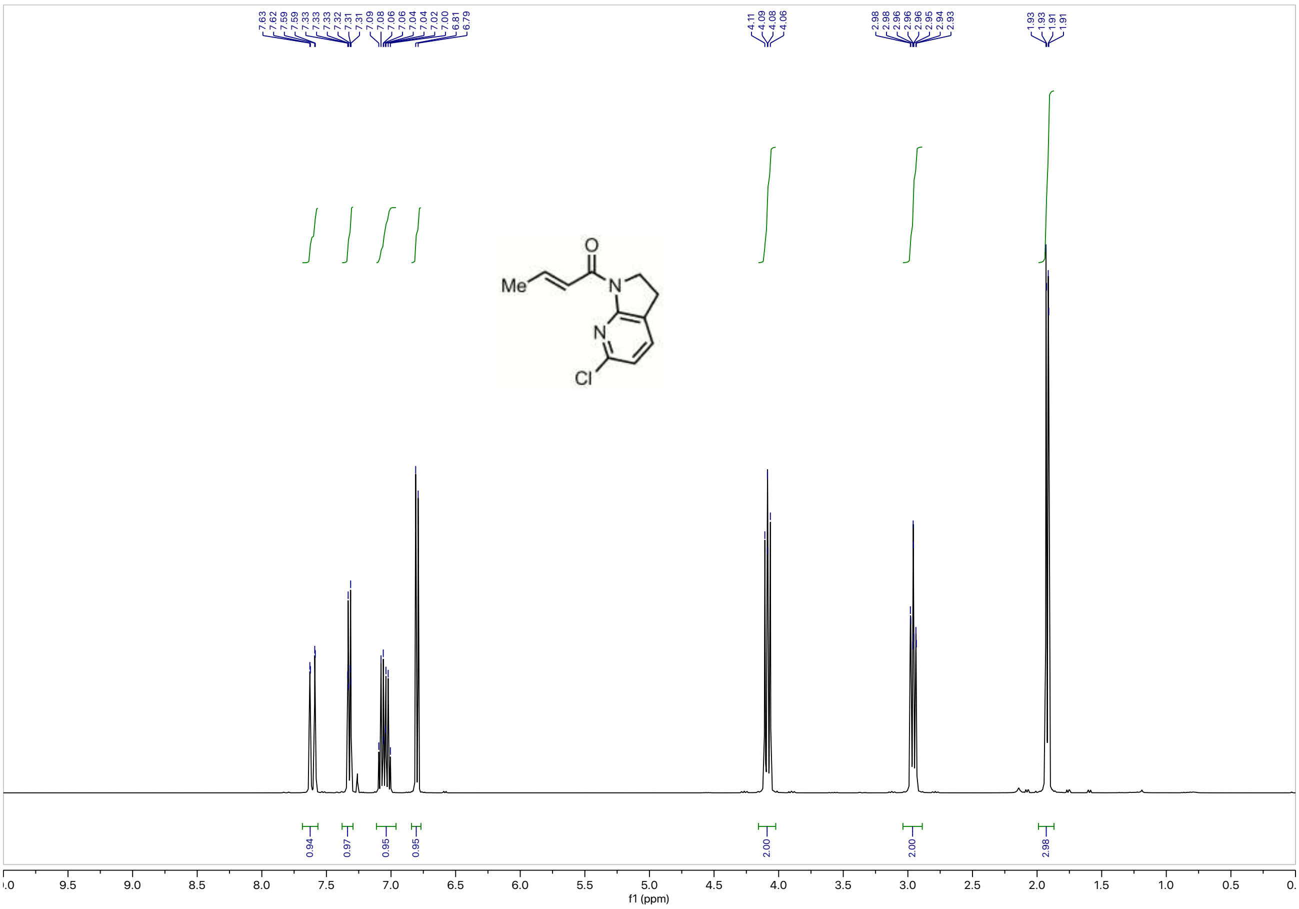

^{13}C NMR: **1e**

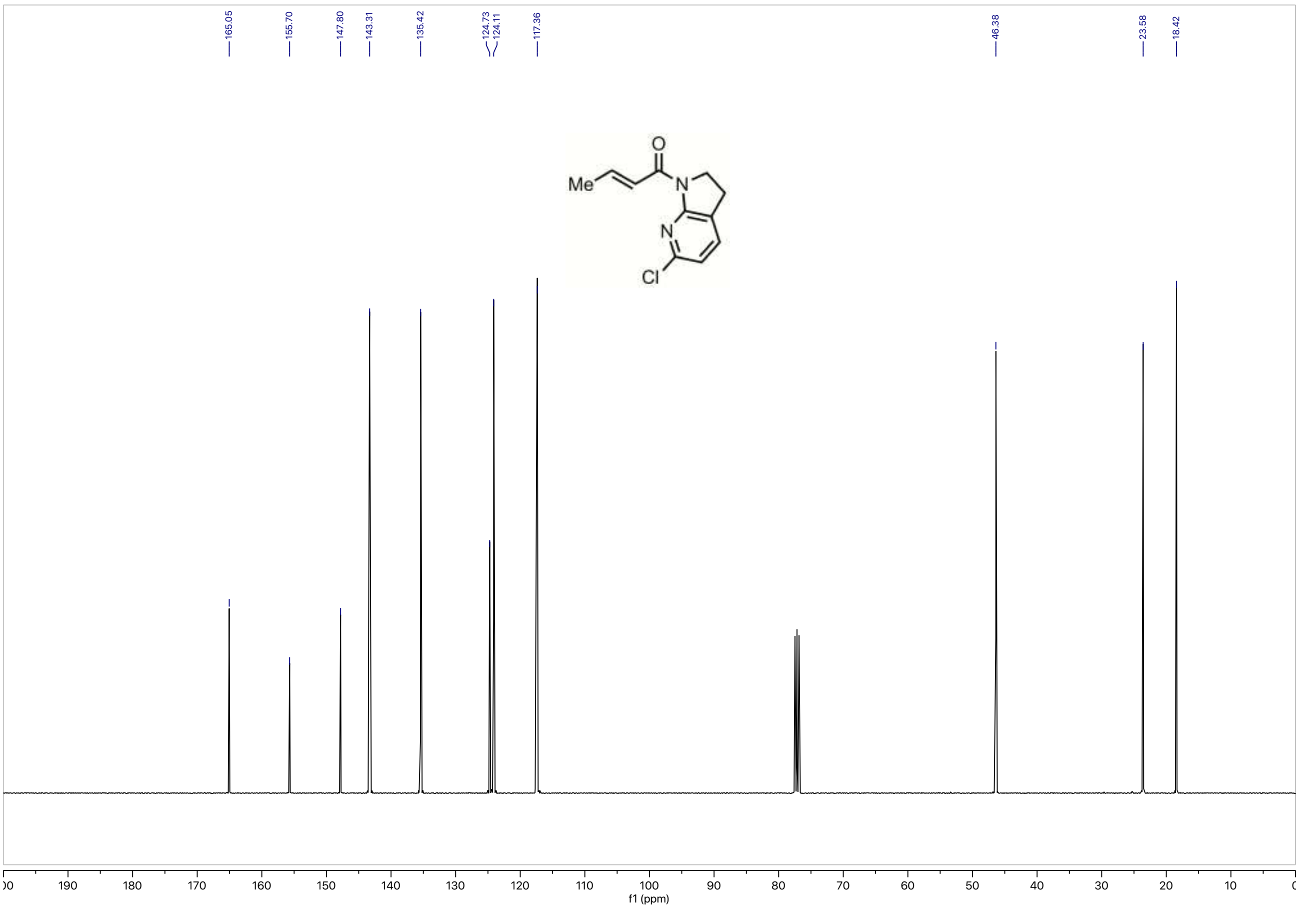

^{1}H NMR: **1f**

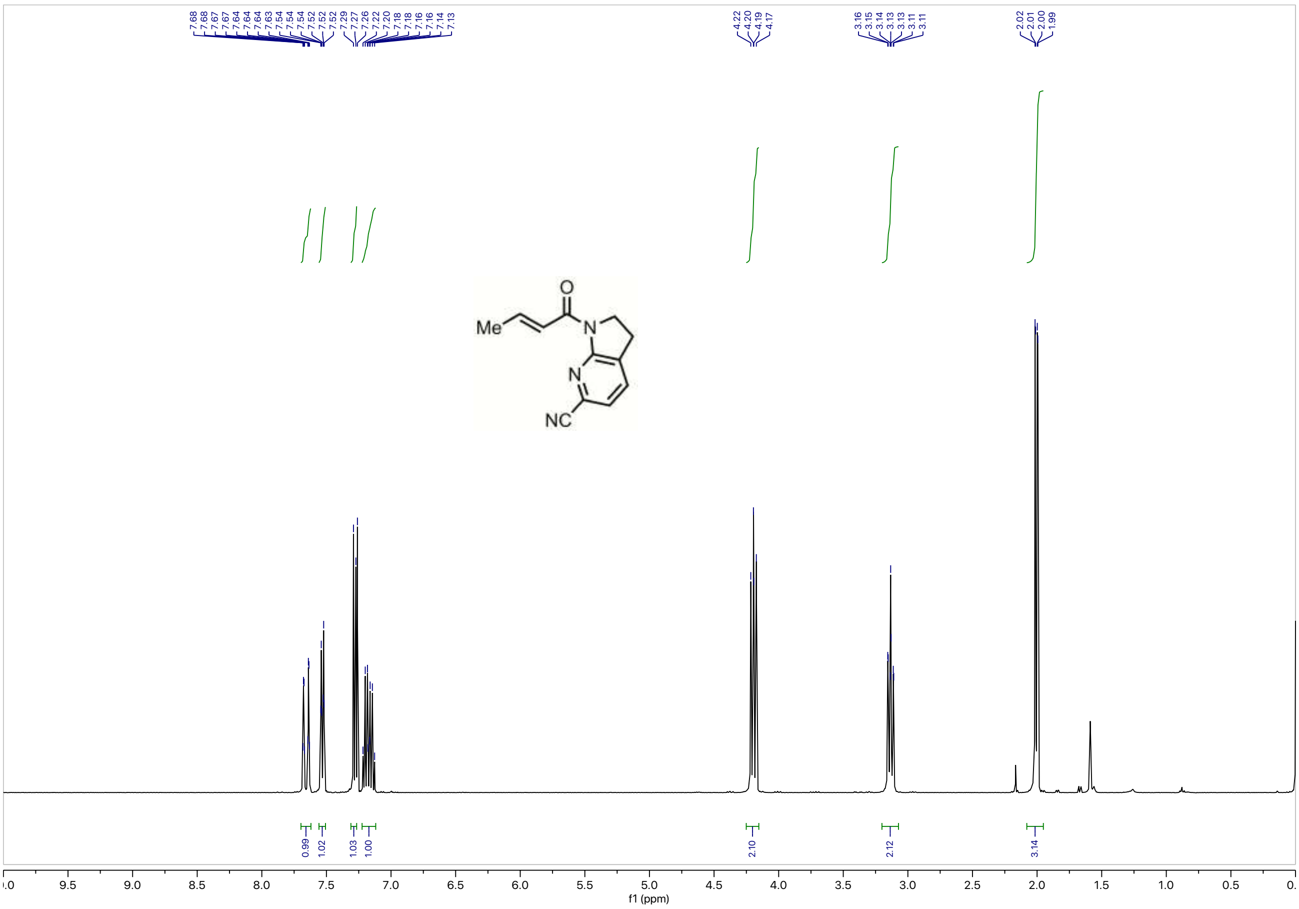

^{13}C NMR: **1f**

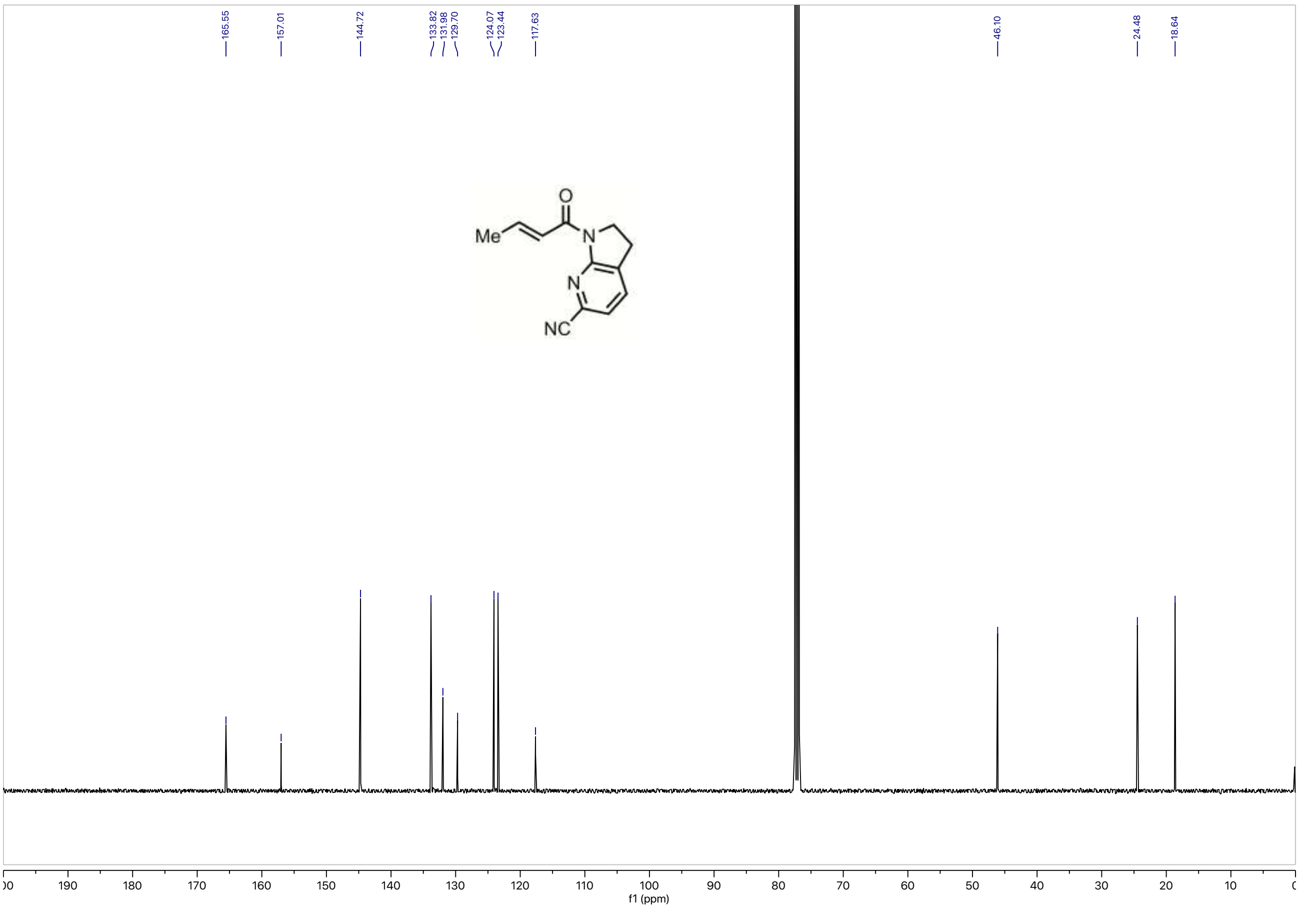

^{1}H NMR: **1g**

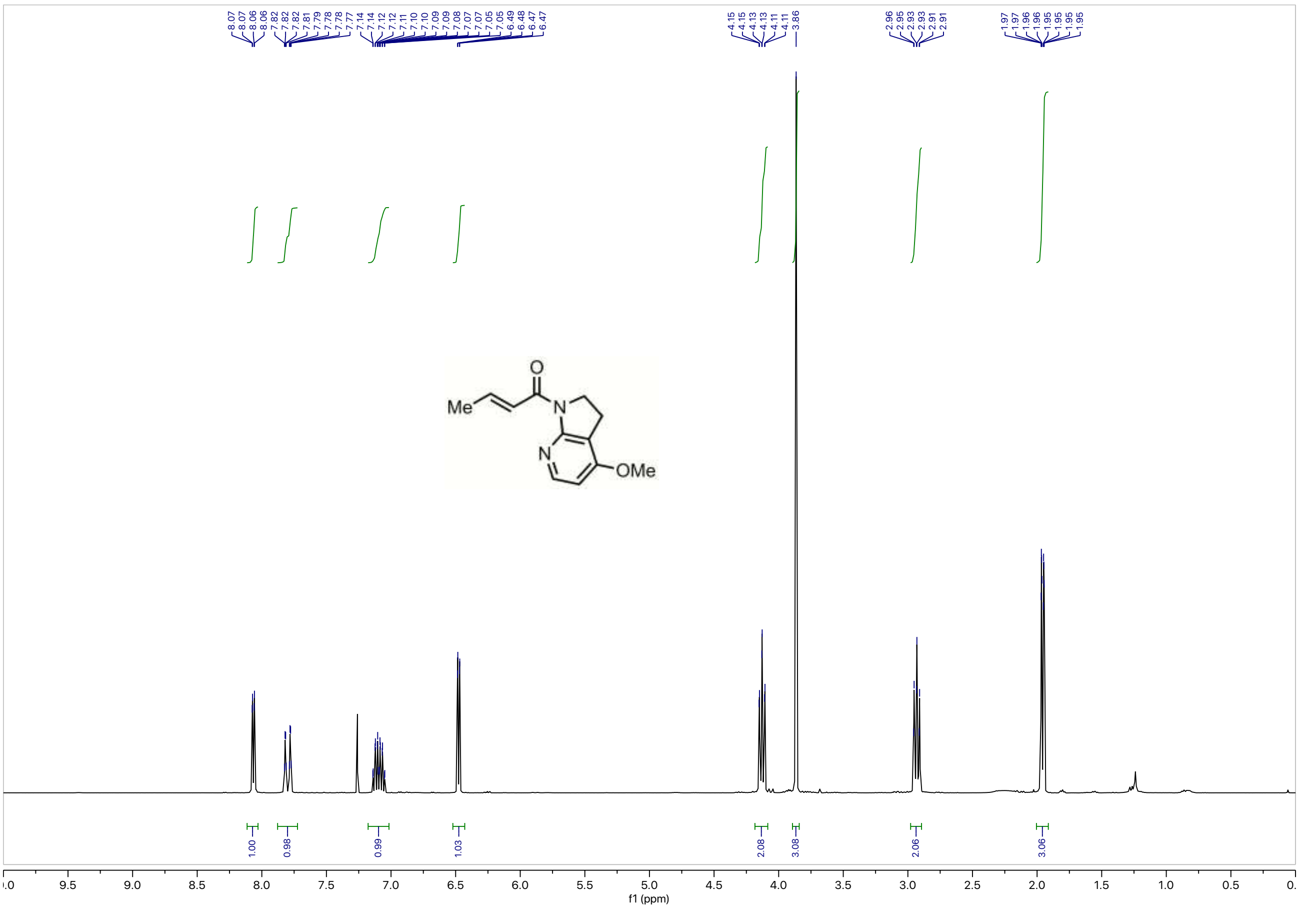

^{13}C NMR: **1g**

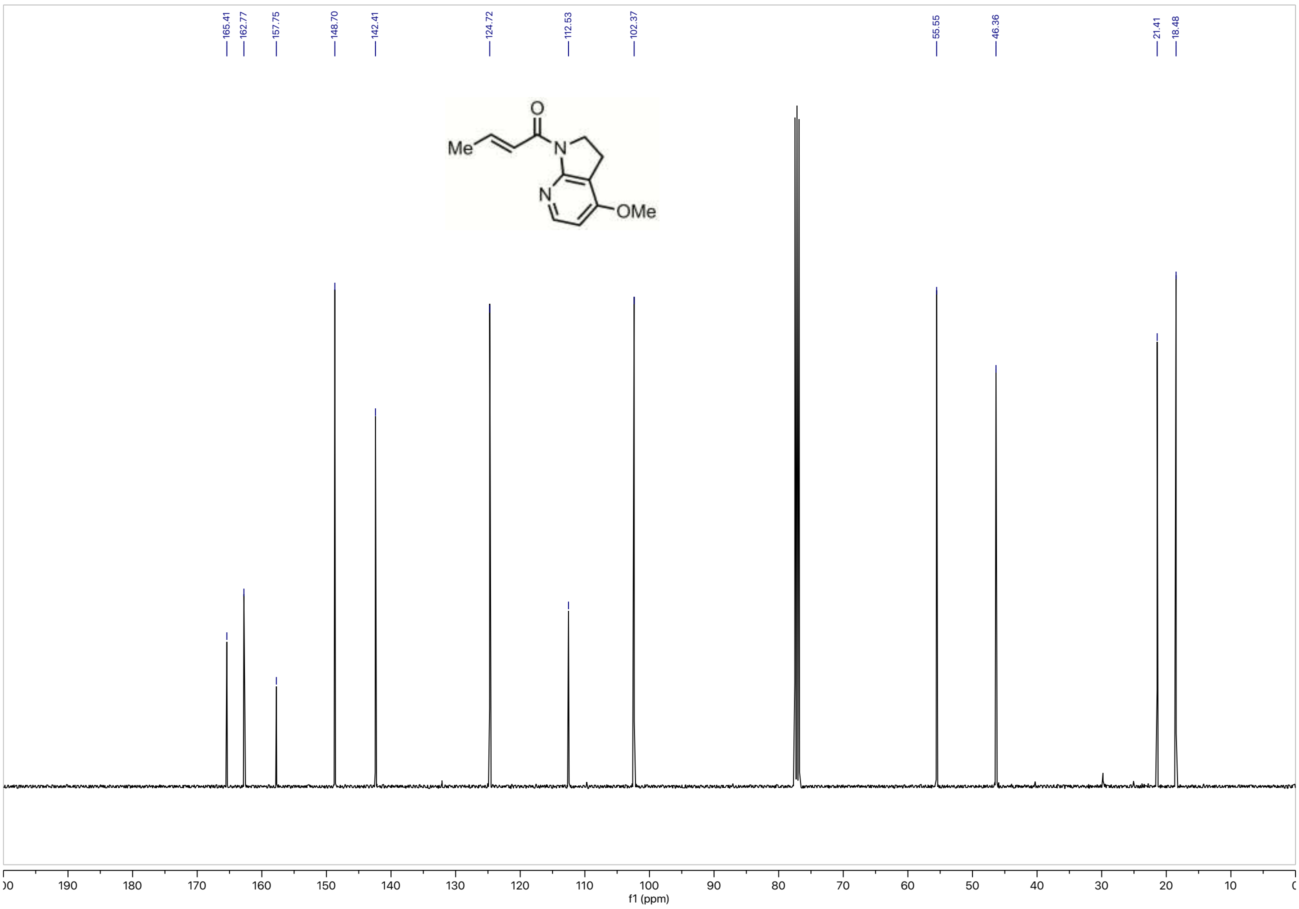

^{1}H NMR: **1h**

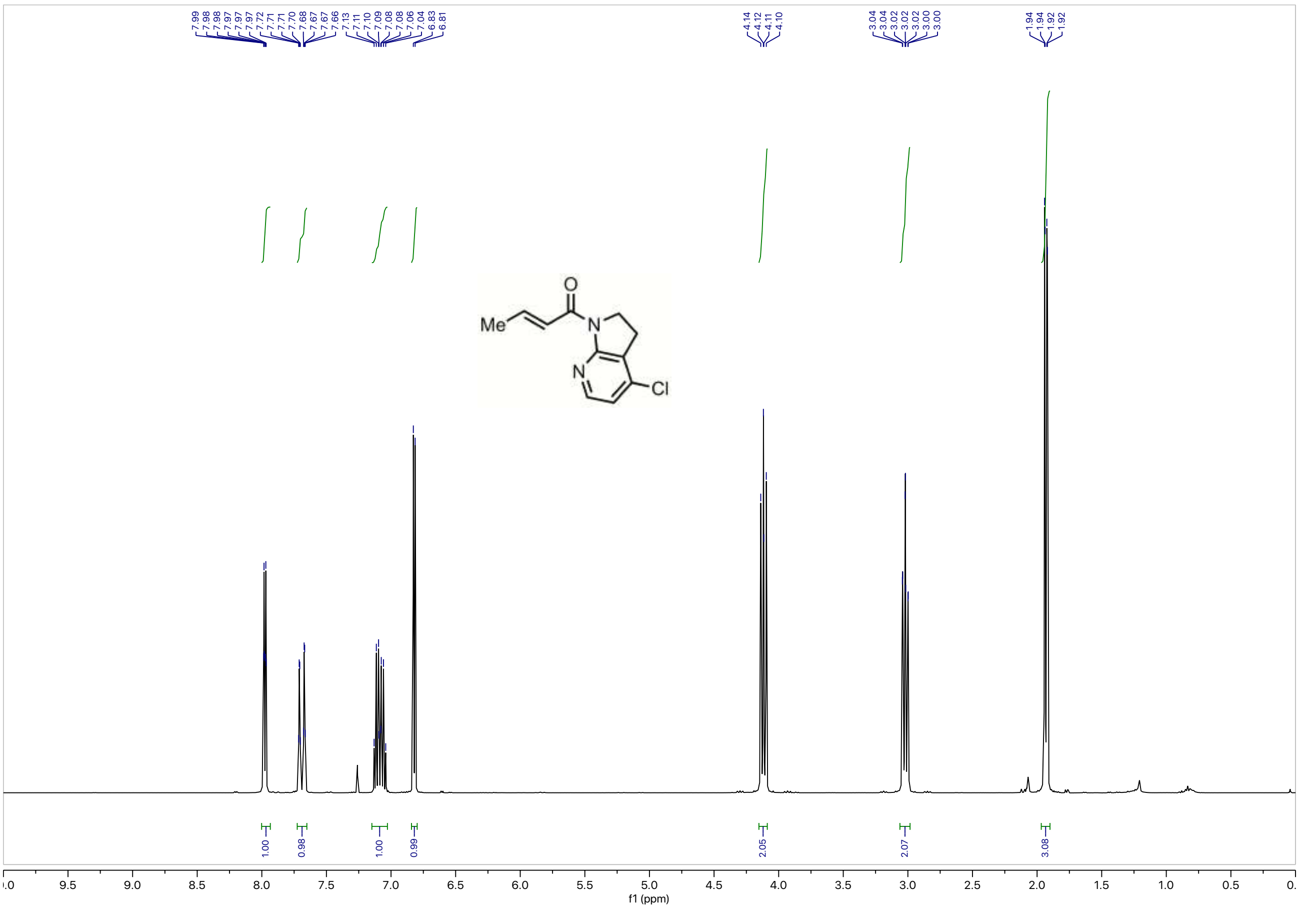

^{13}C NMR: **1h**

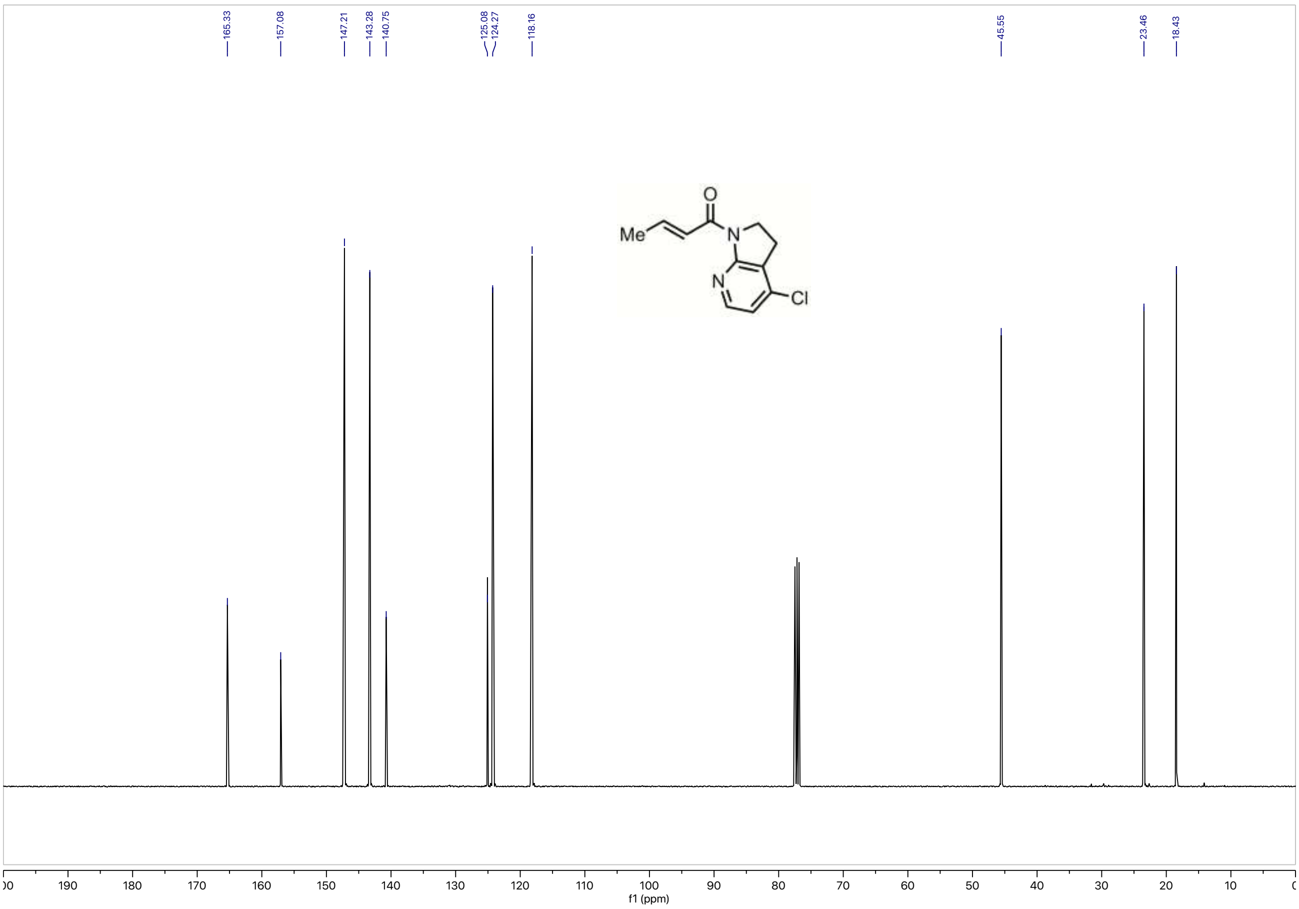

^{1}H NMR: **1i**

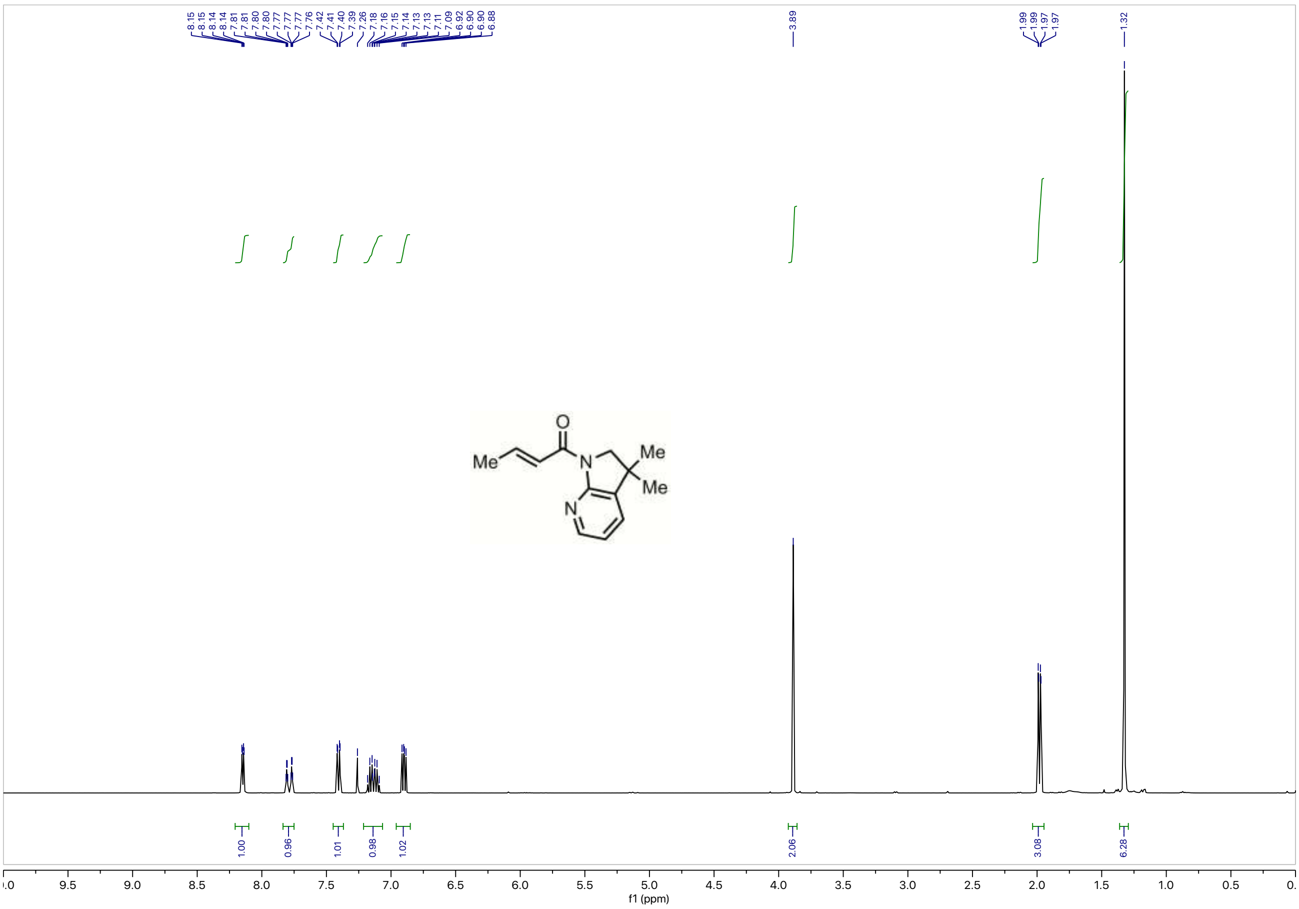

^{13}C NMR: **1i**

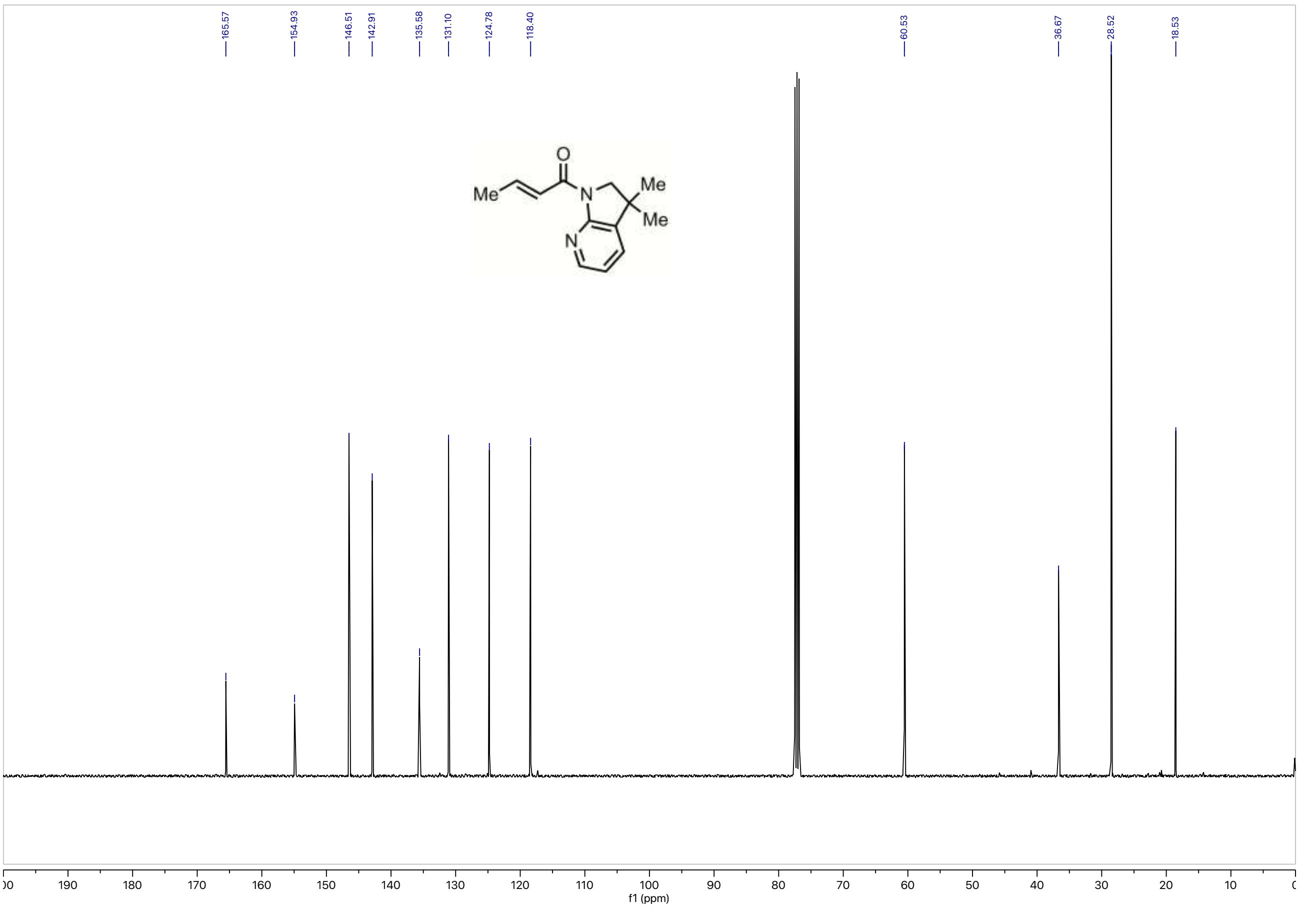

^{1}H NMR: **1j**

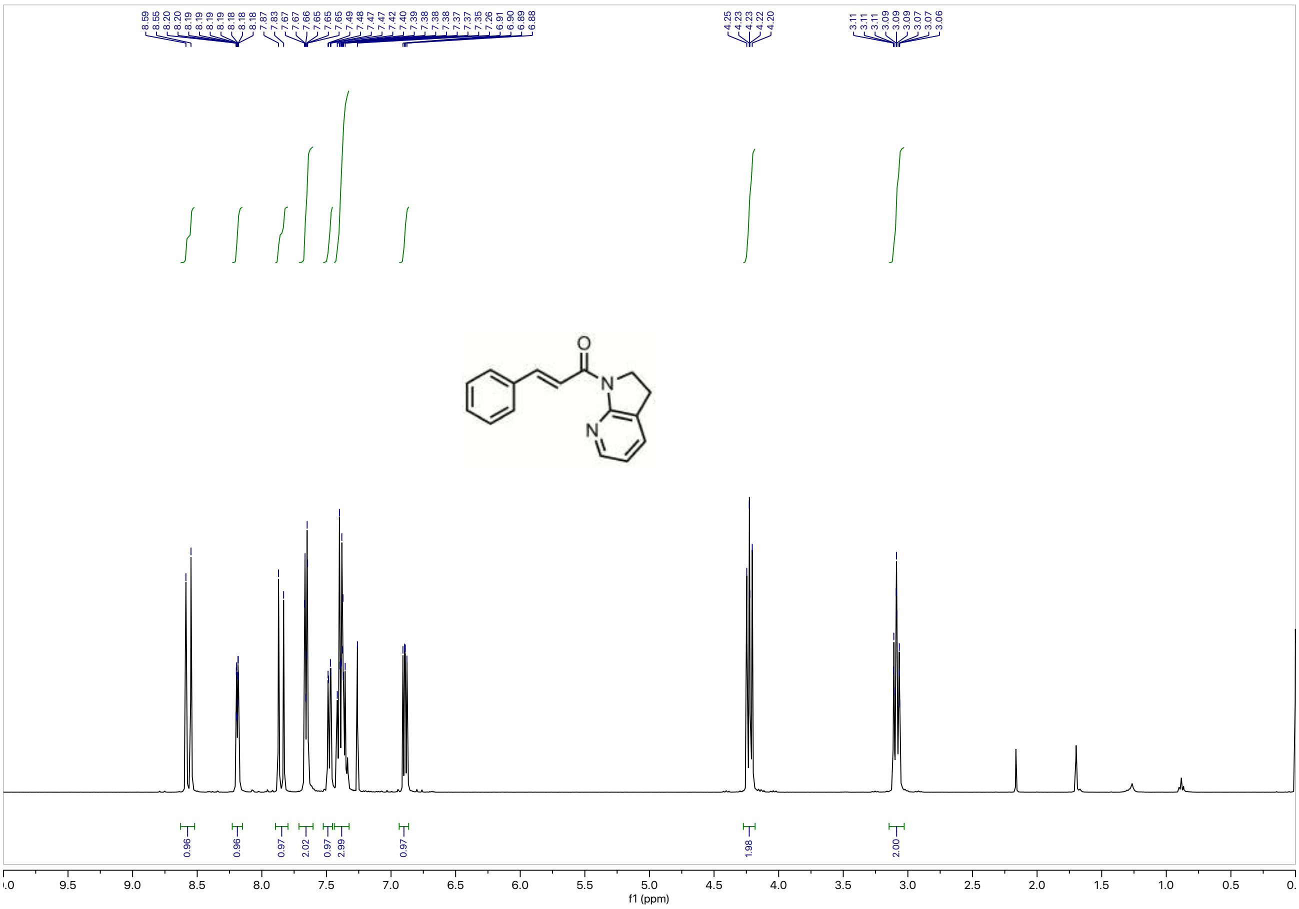

^{13}C NMR: **1j**

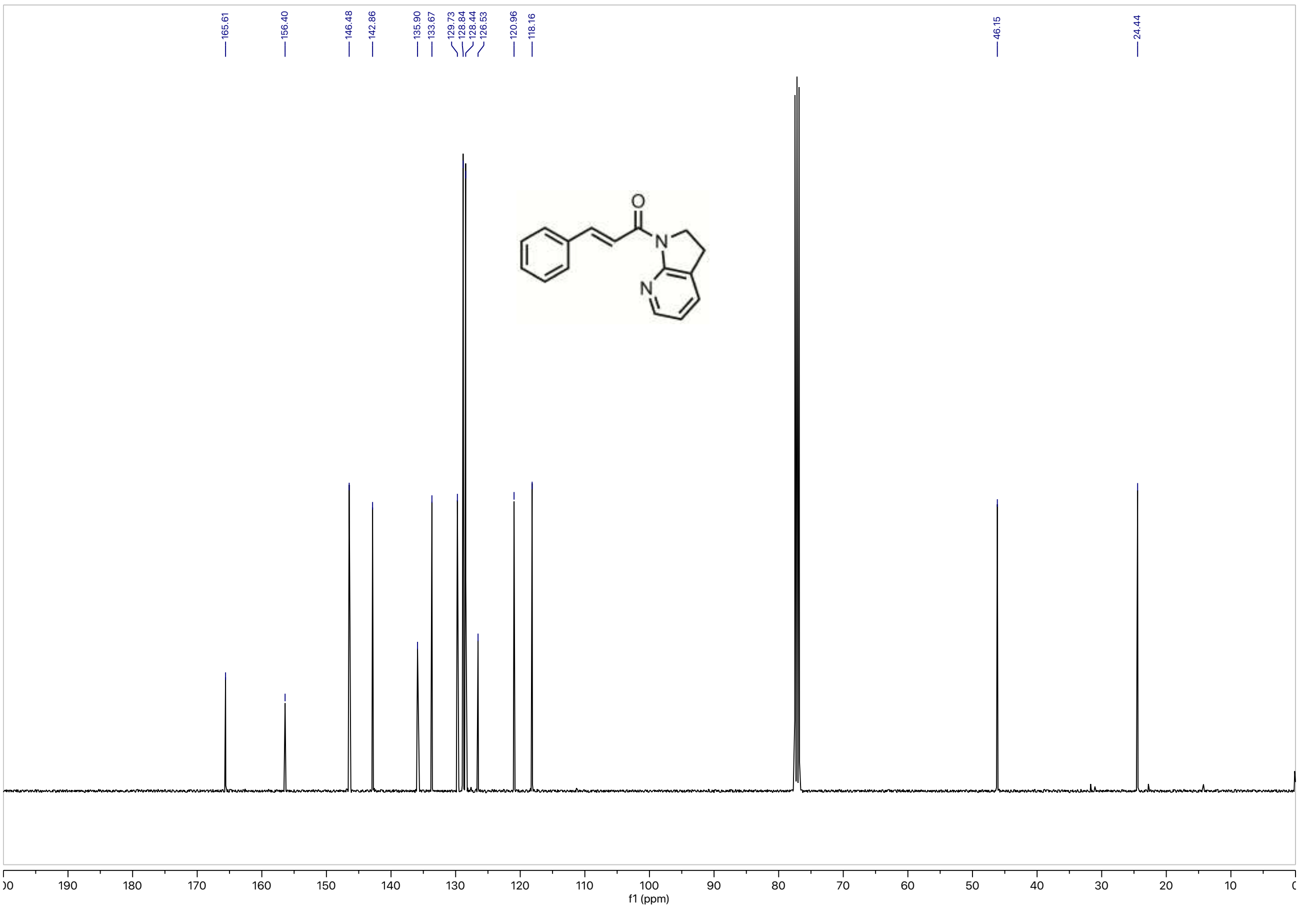

^{1}H NMR: **1k**

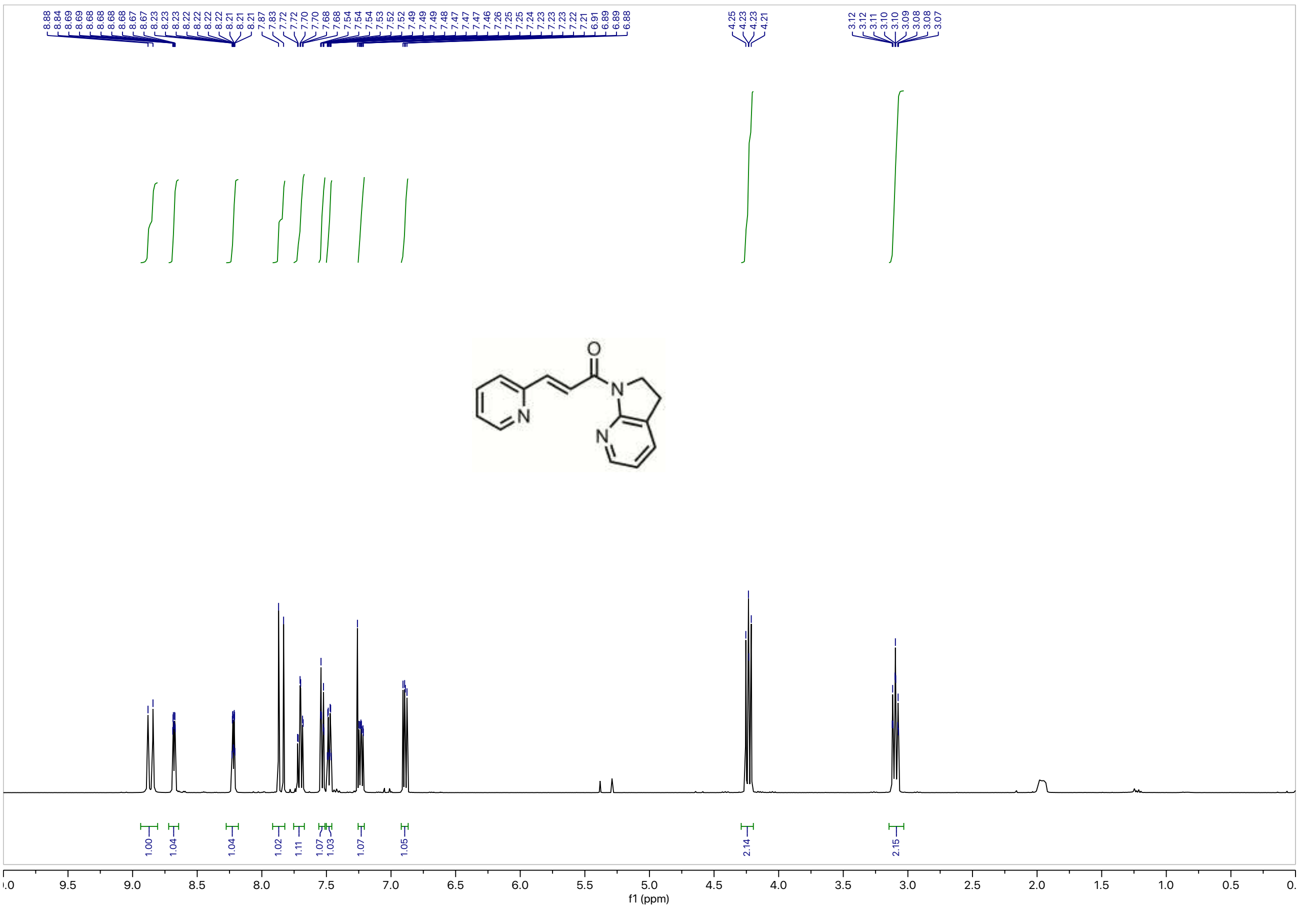

^{13}C NMR: **1k**

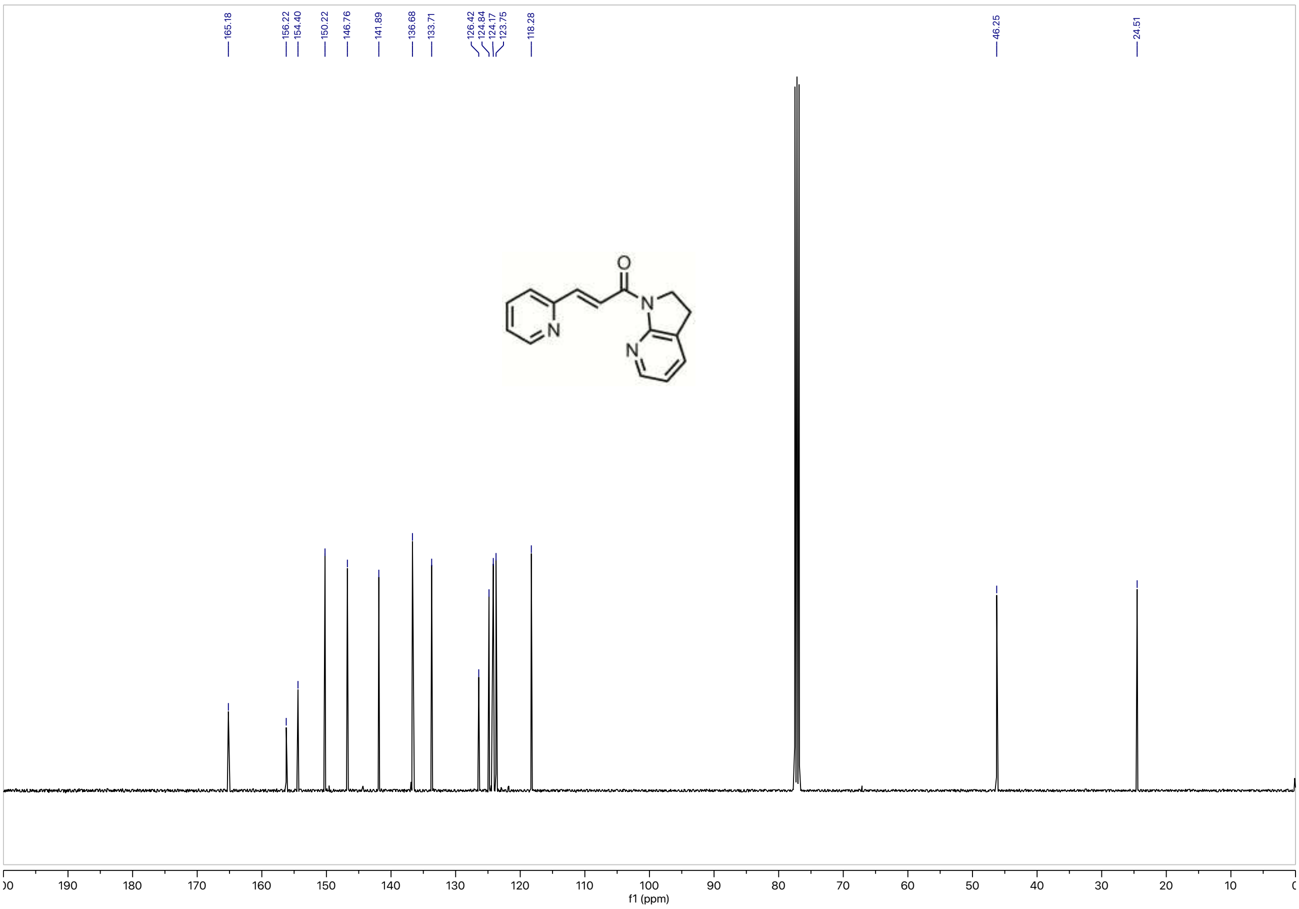

¹H NMR: **1l**

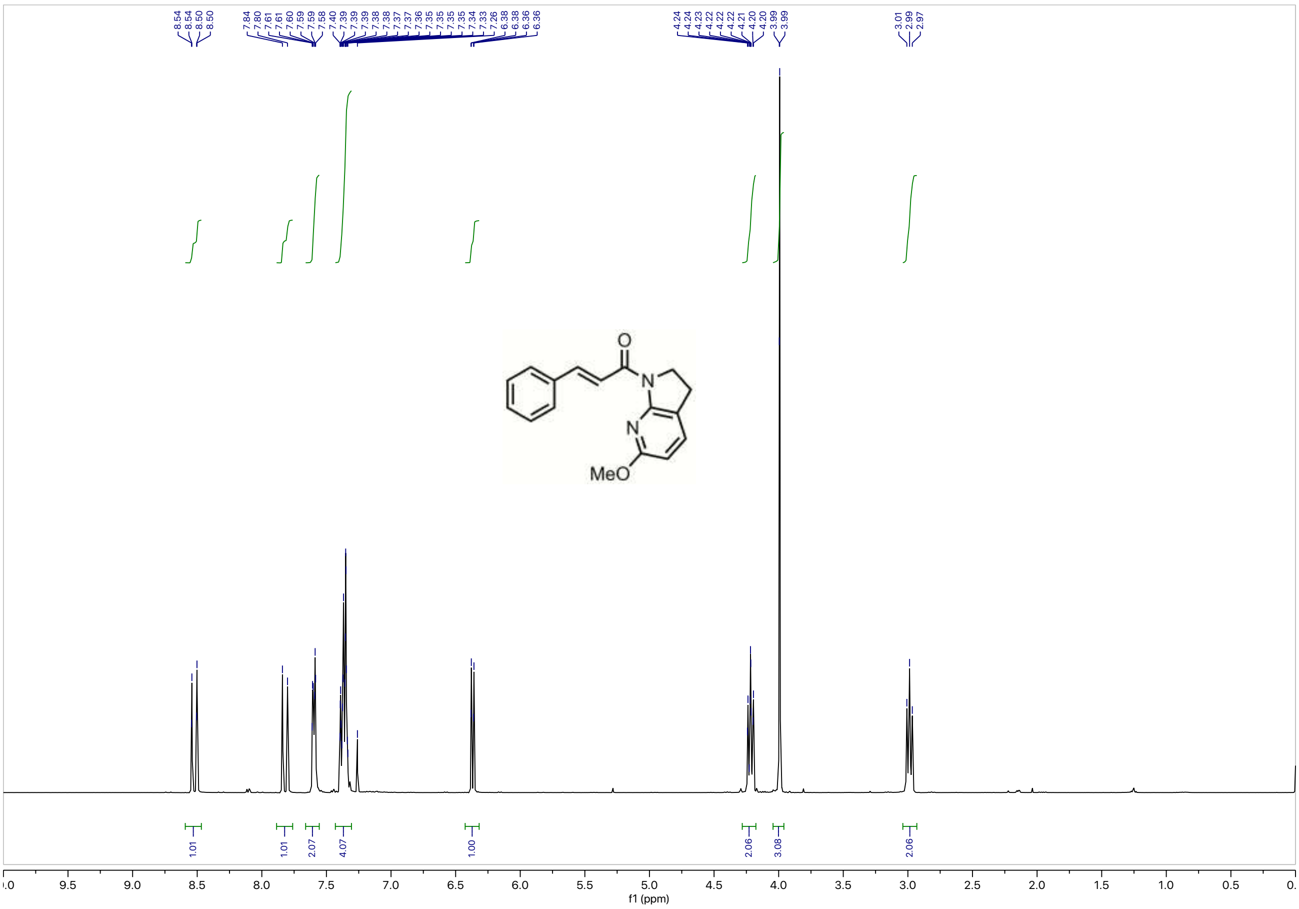

¹³C NMR: **1l**

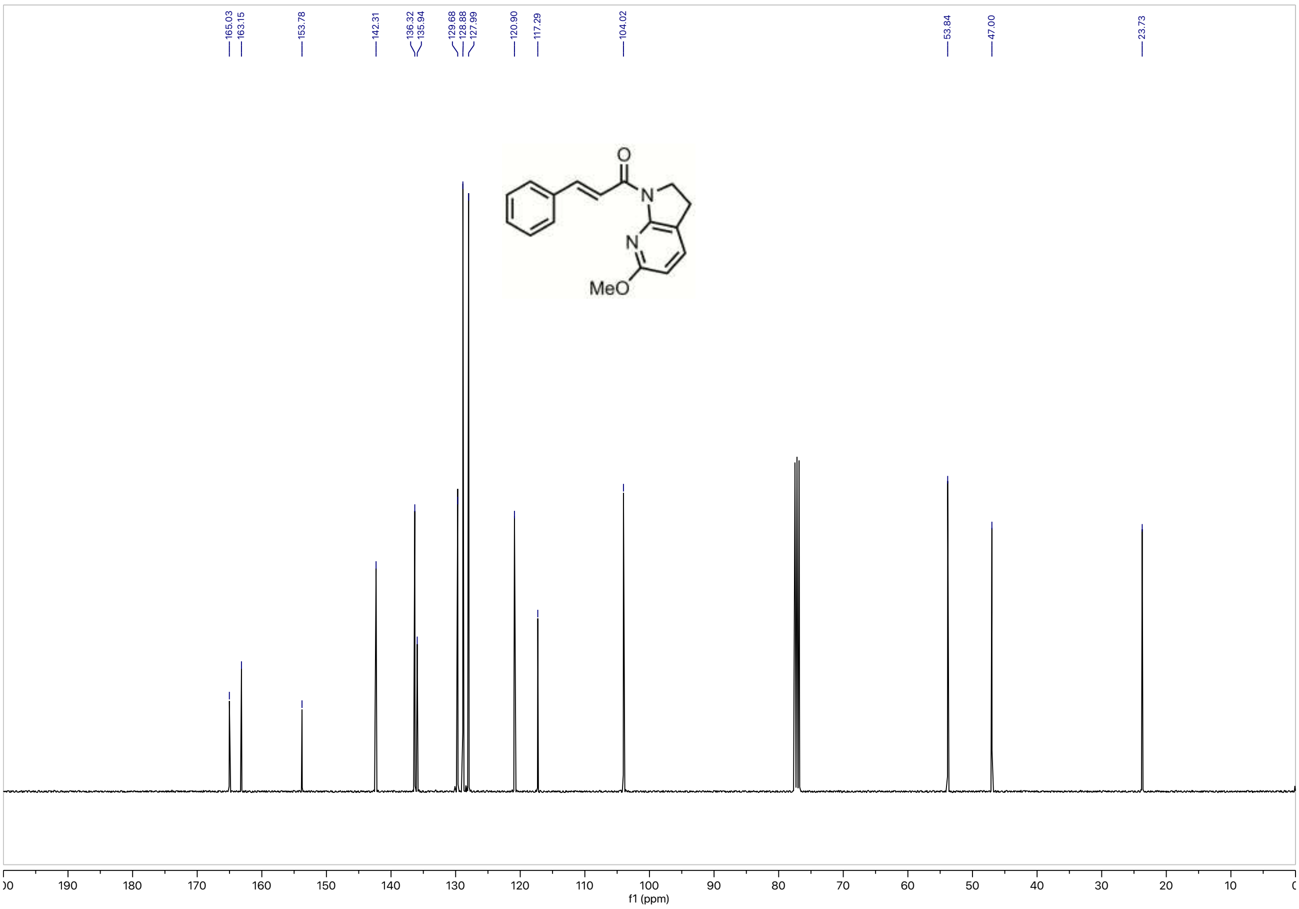

^{1}H NMR: **1m**

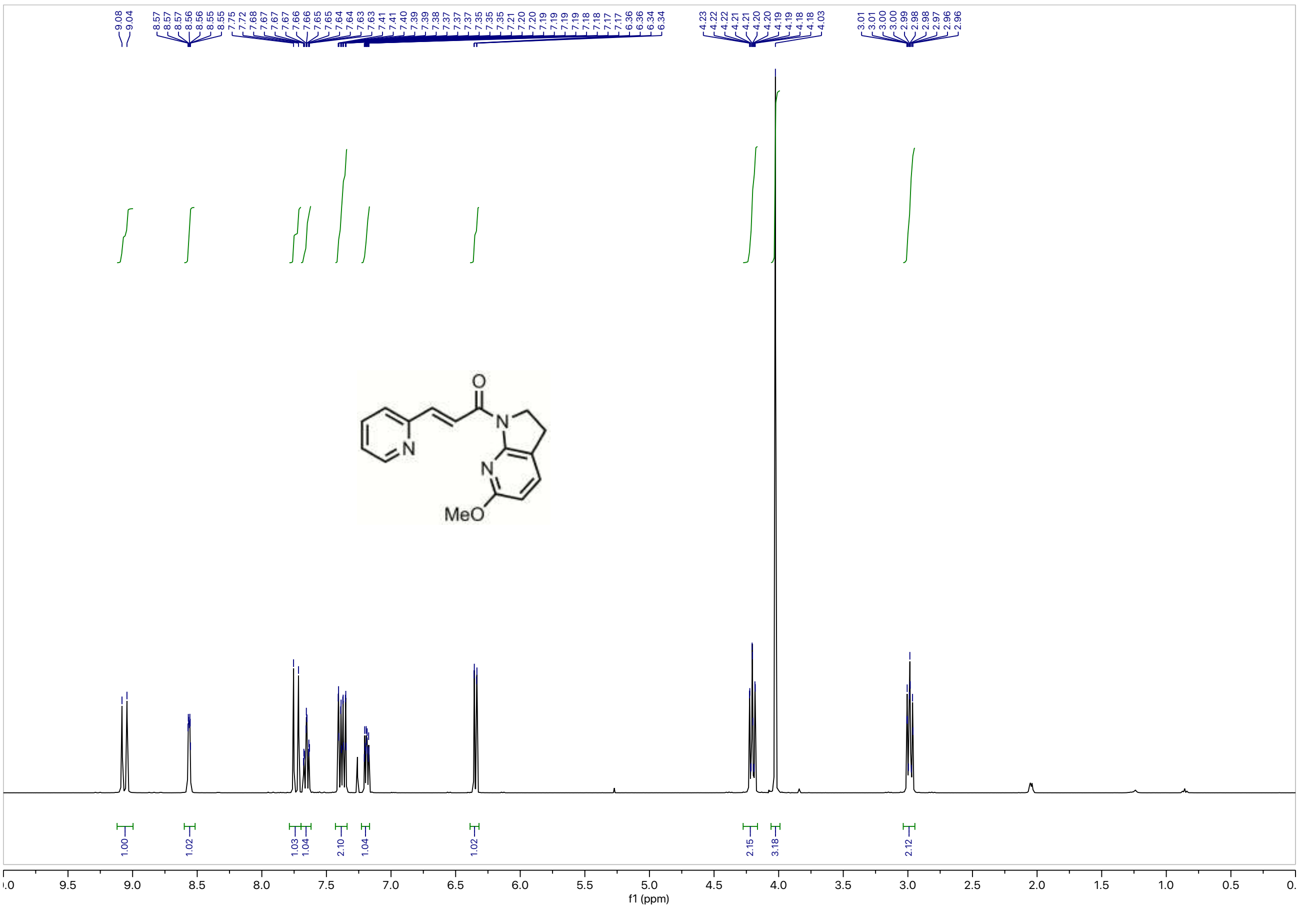

^{13}C NMR: **1m**

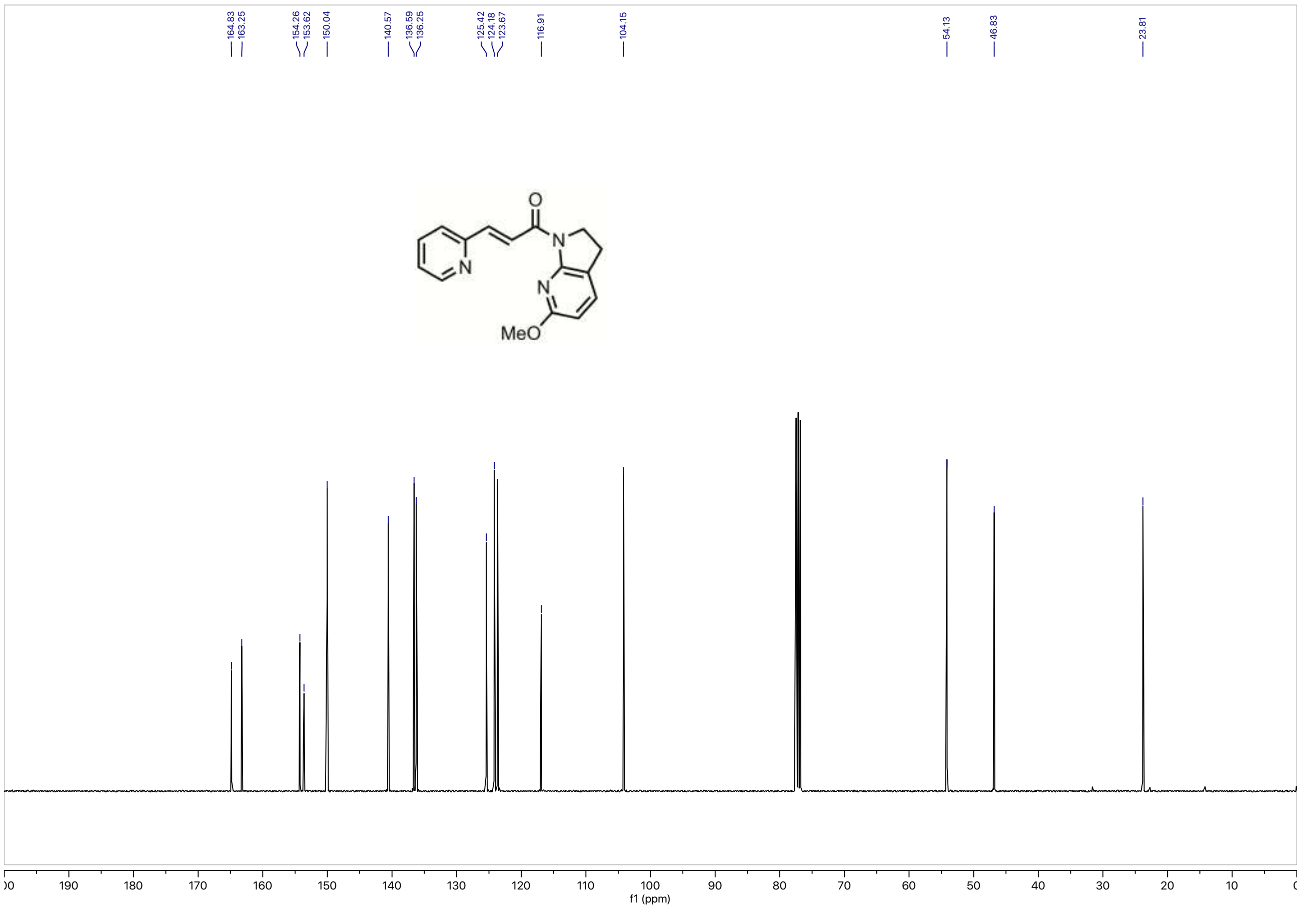

^{1}H NMR: **1n**

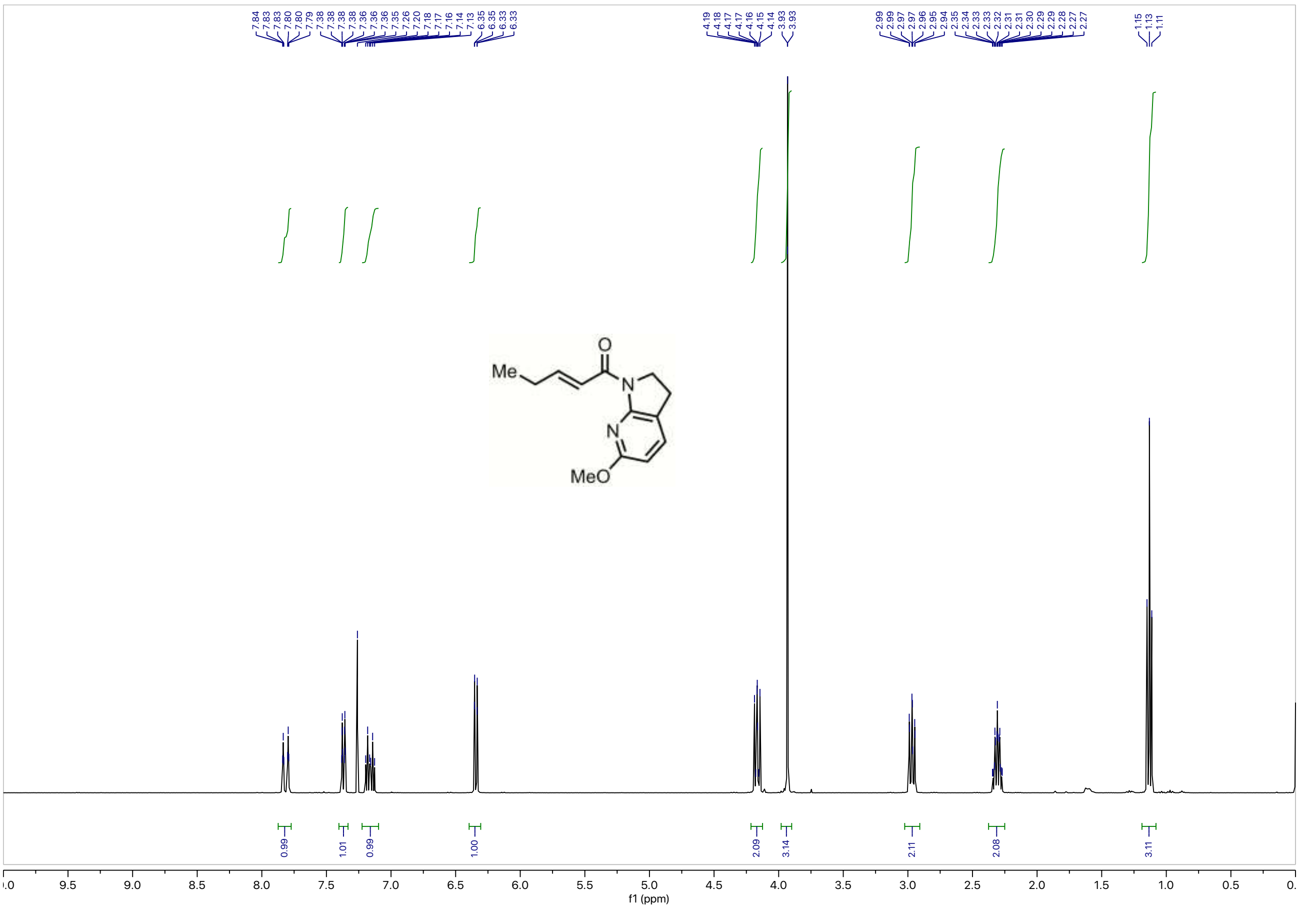

^{13}C NMR: **1n**

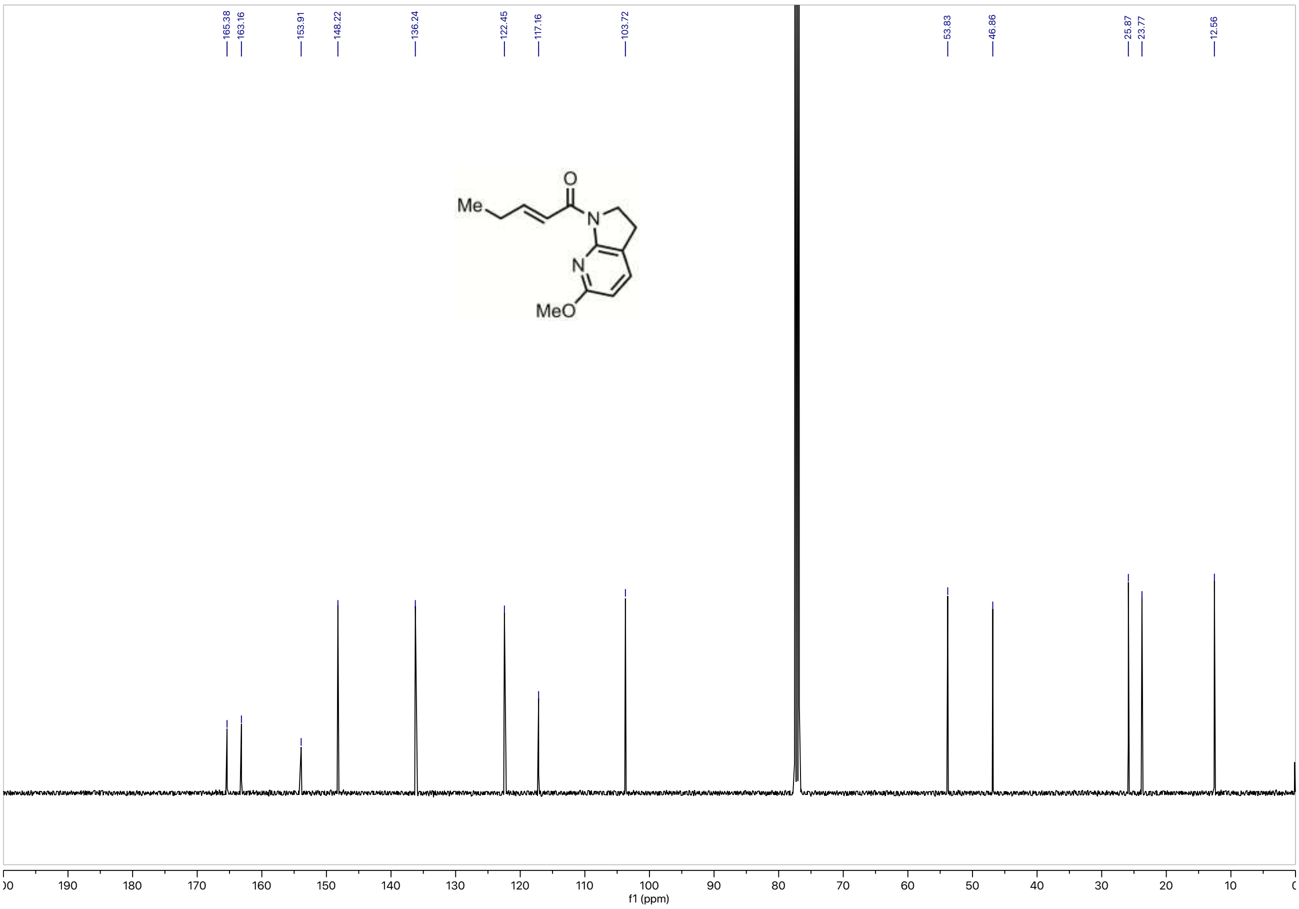

^{1}H NMR: **1o**

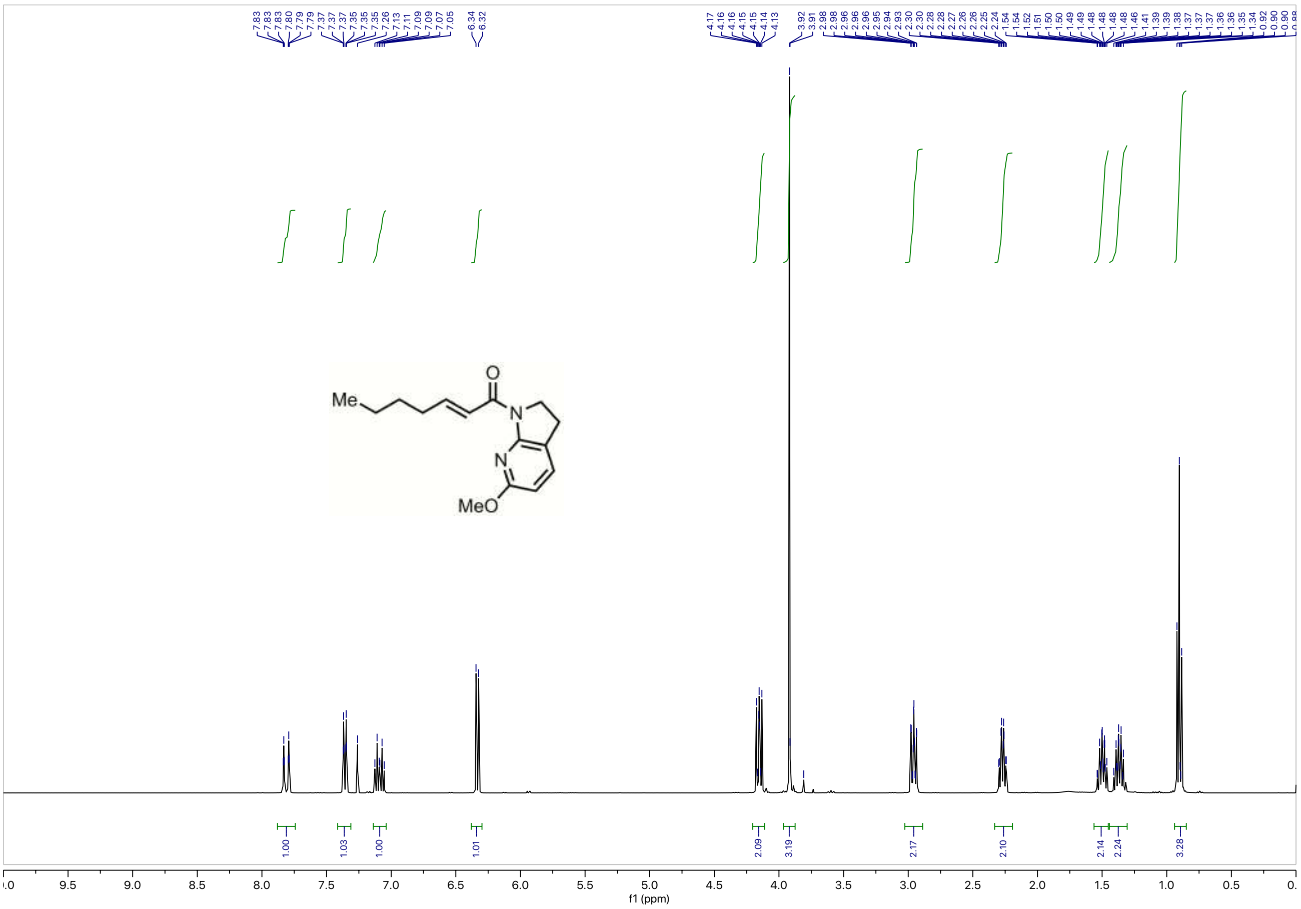

^{13}C NMR: **1o**

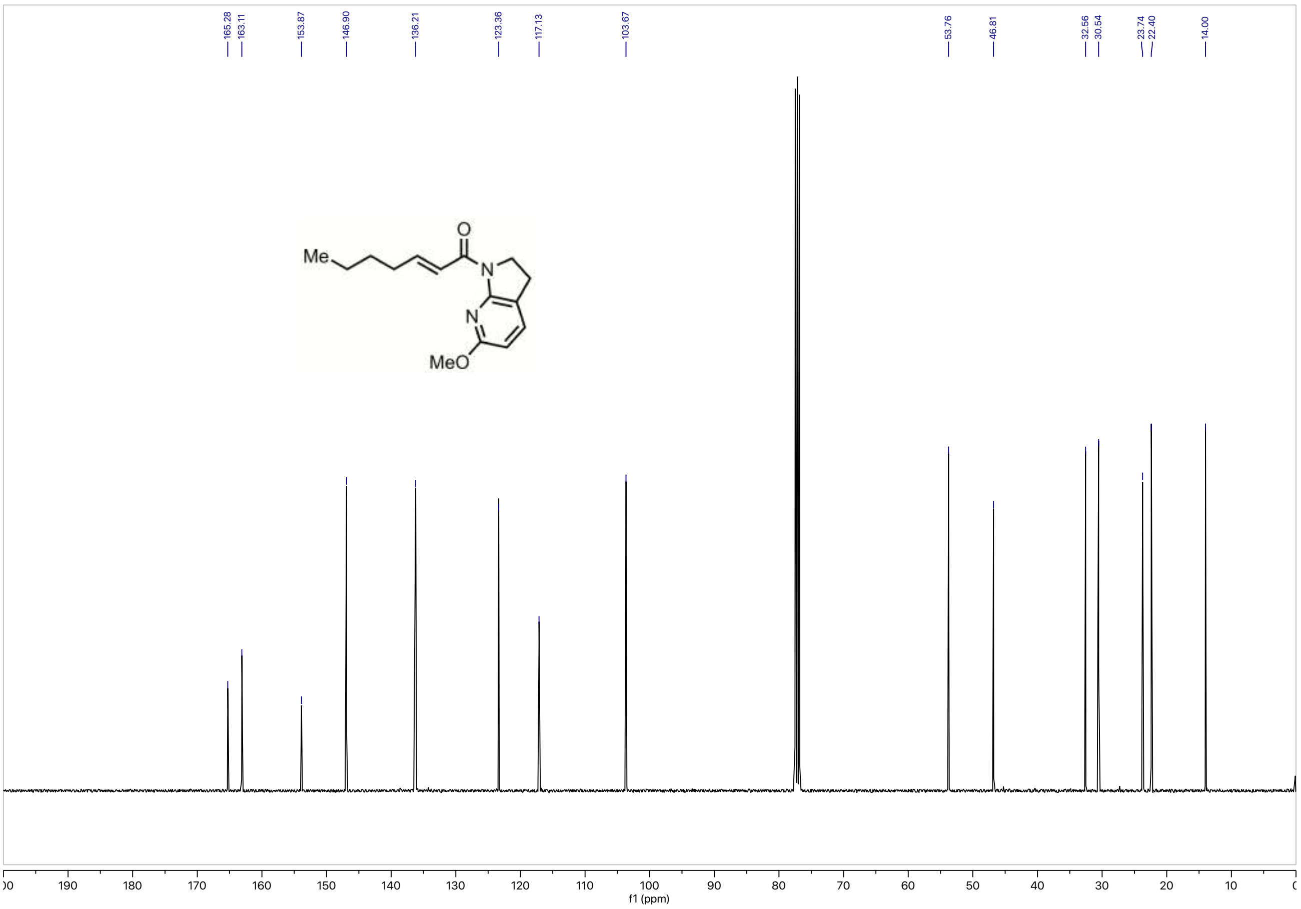

^{1}H NMR: **1p**

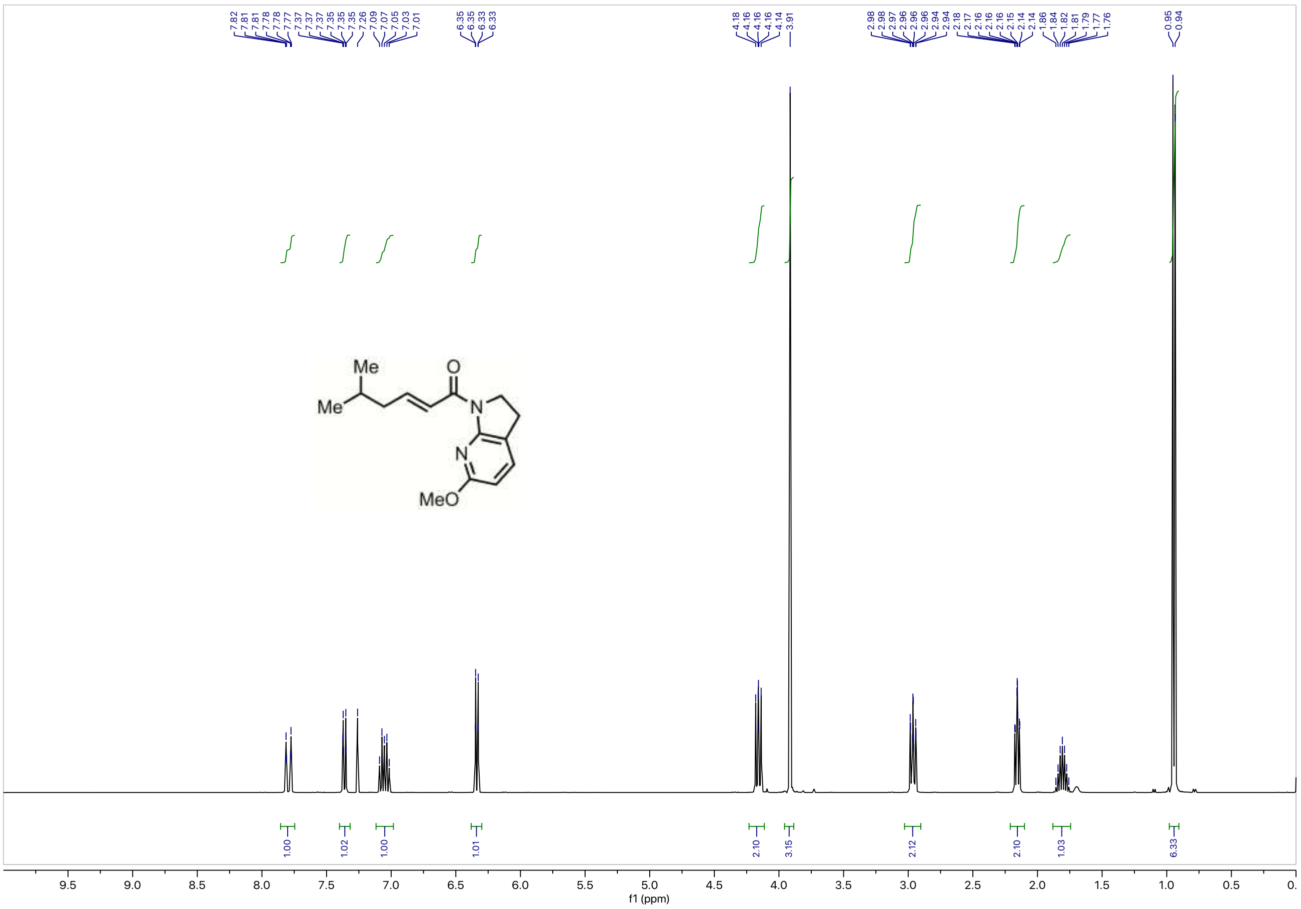

^{13}C NMR: **1p**

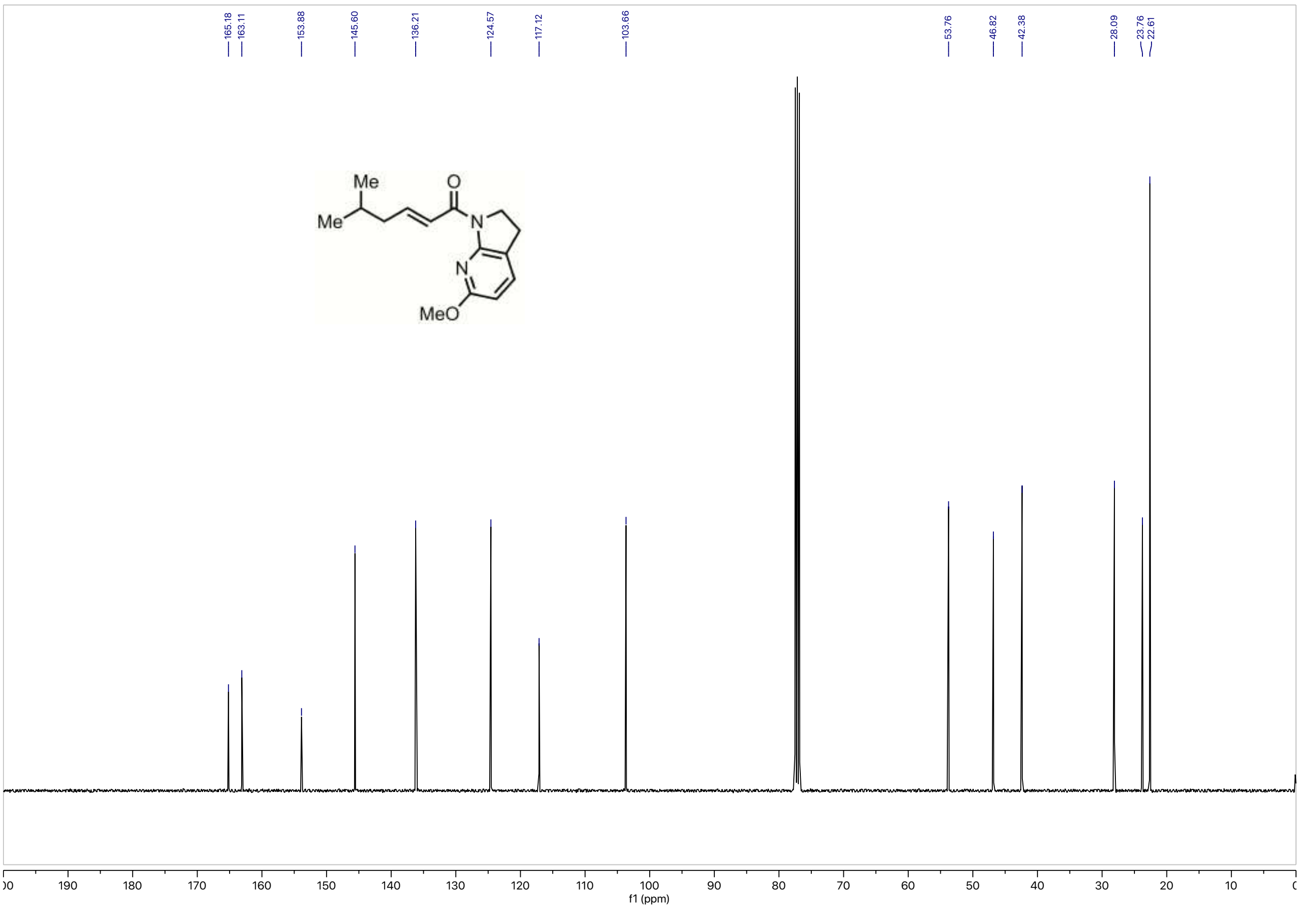

^{1}H NMR: **1q**

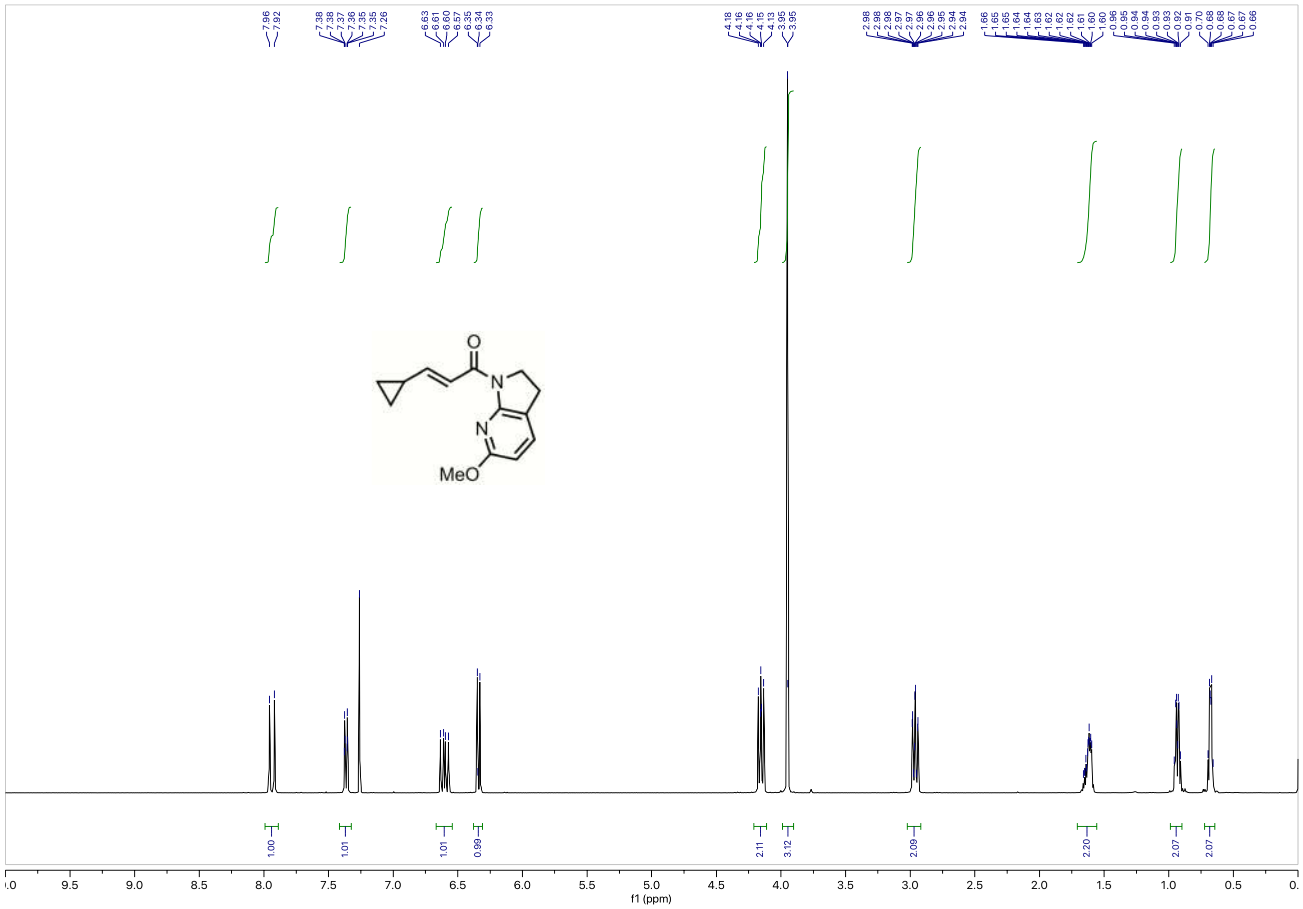

^{13}C NMR: **1q**

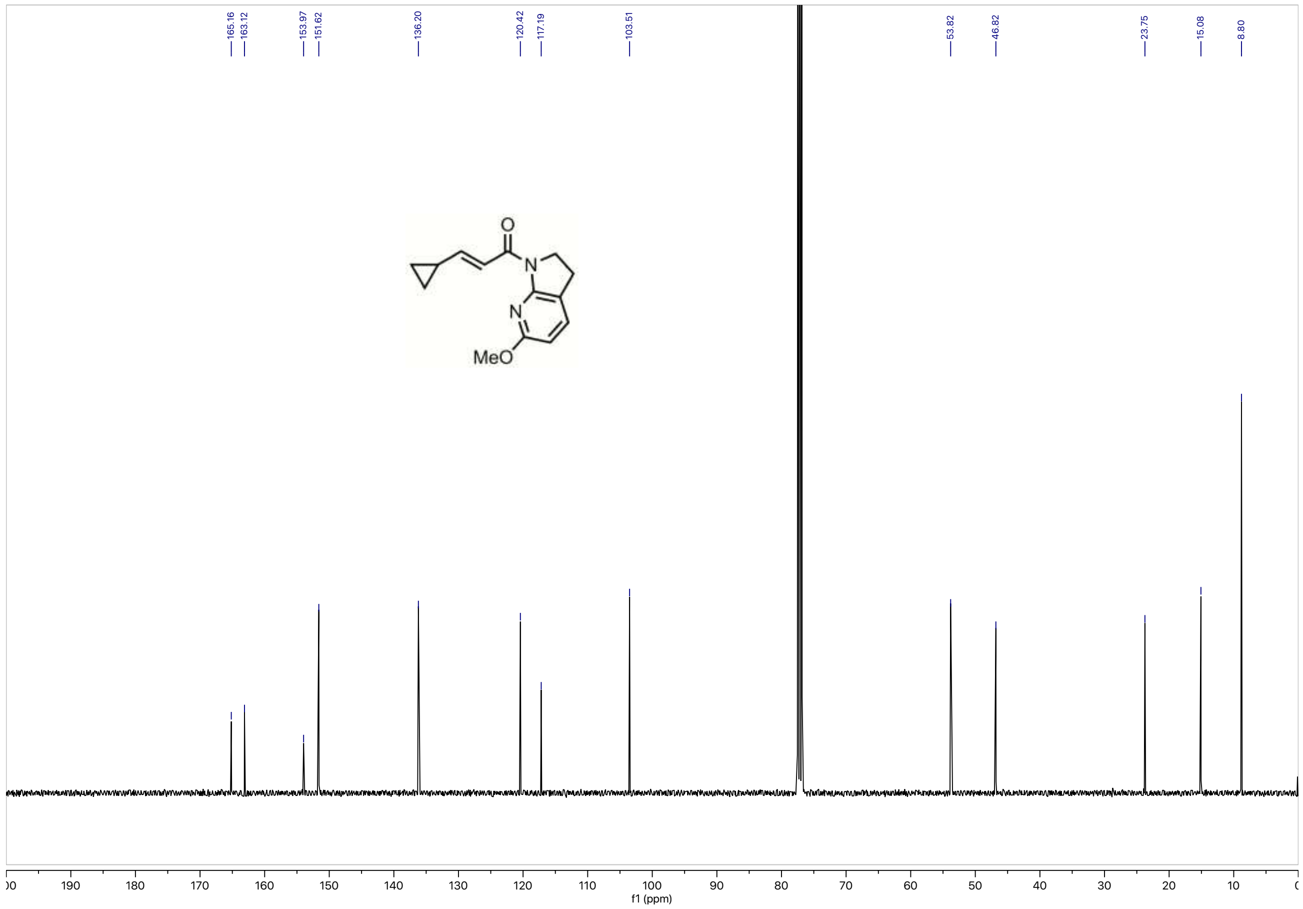

¹H NMR: **1r**

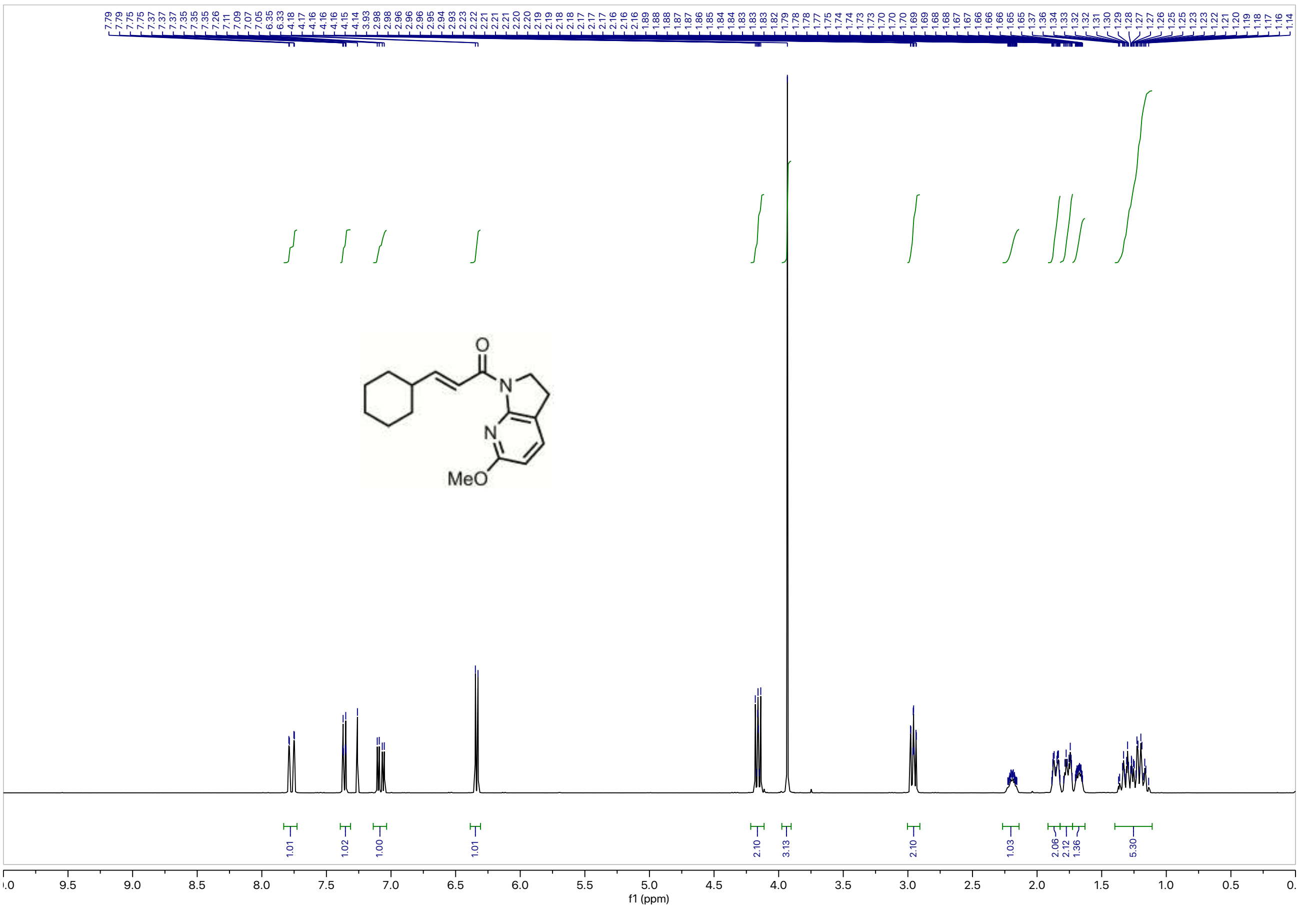

¹³C NMR: **1r**

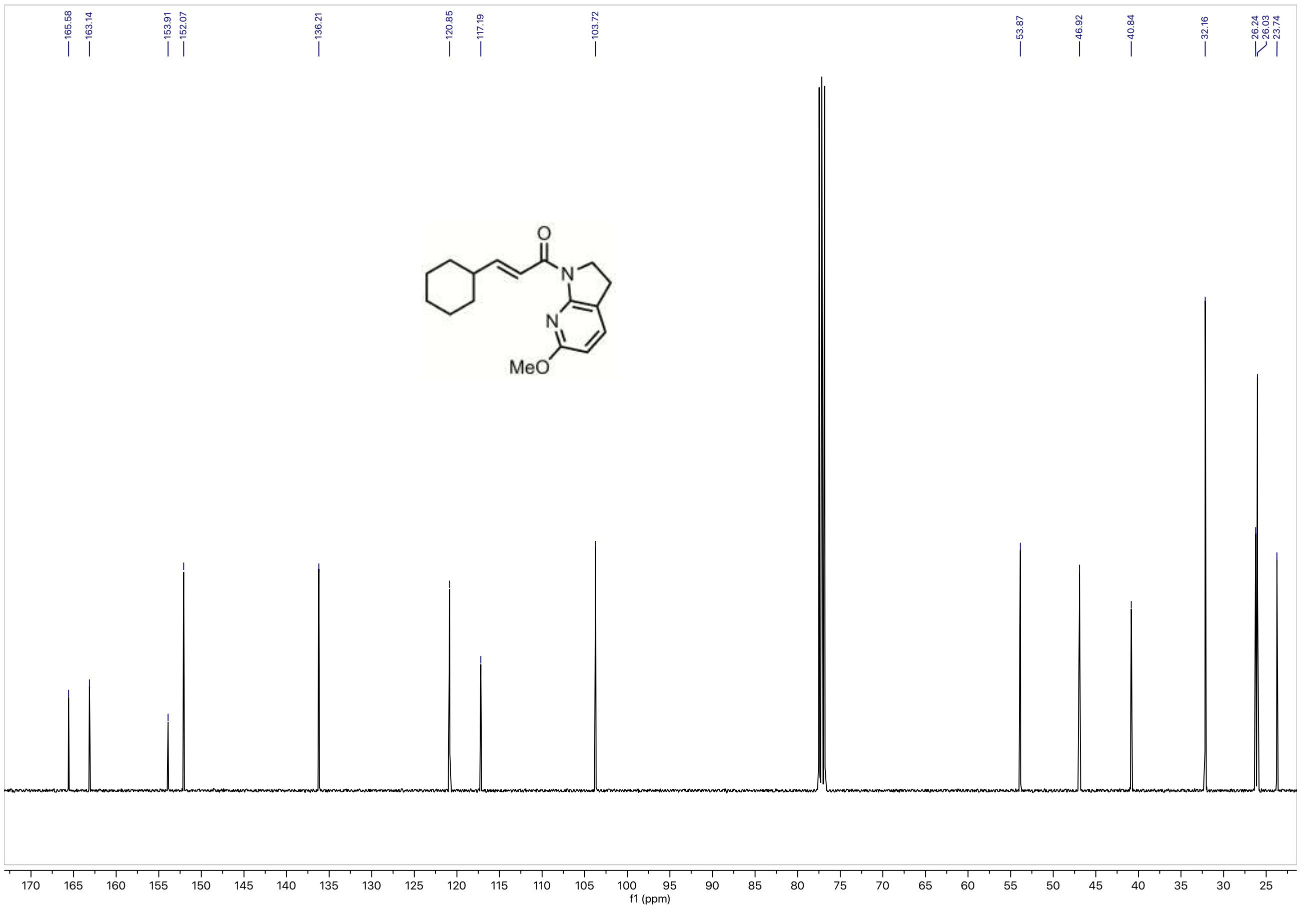

^{1}H NMR: **1s**

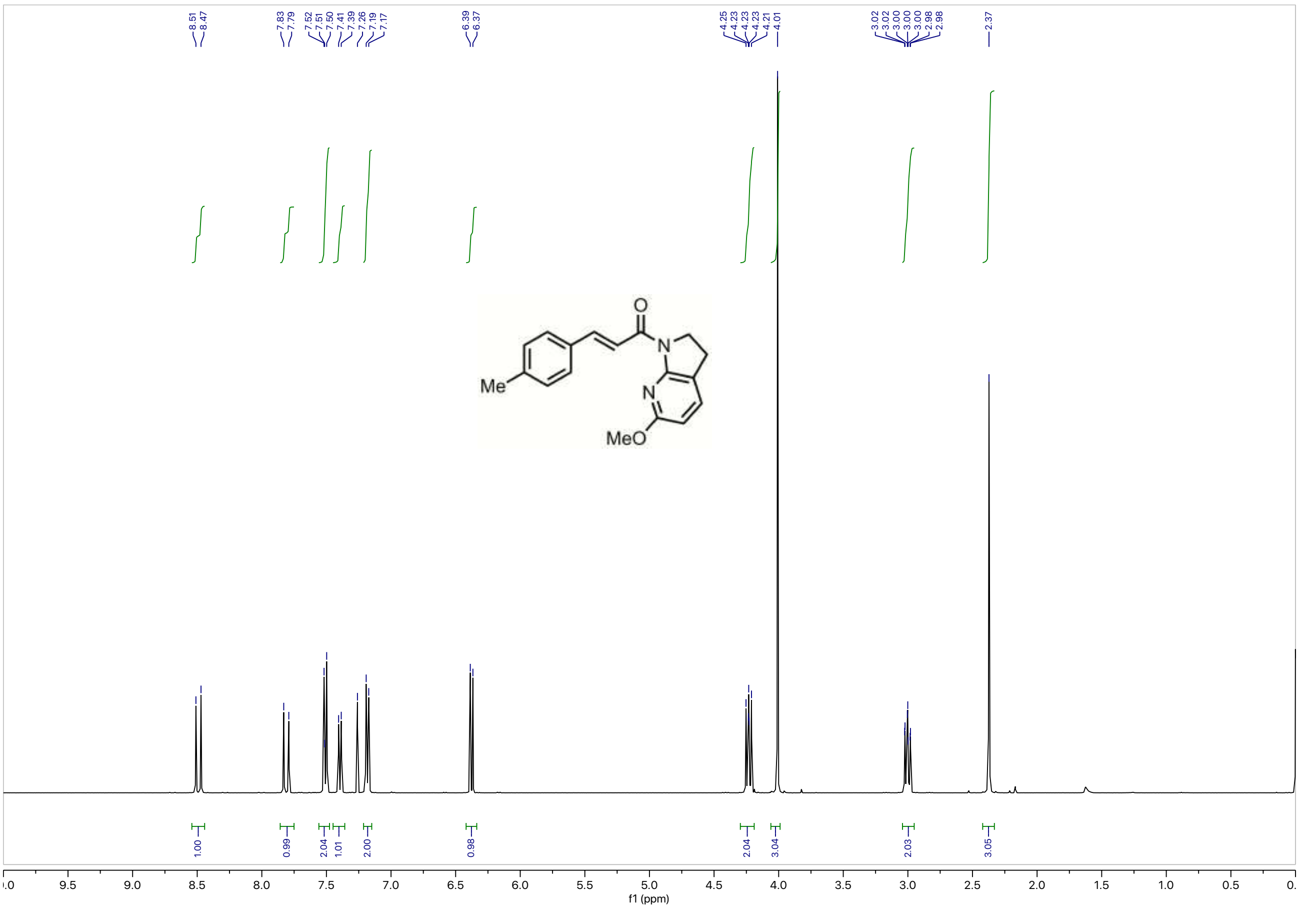

^{13}C NMR: **1s**

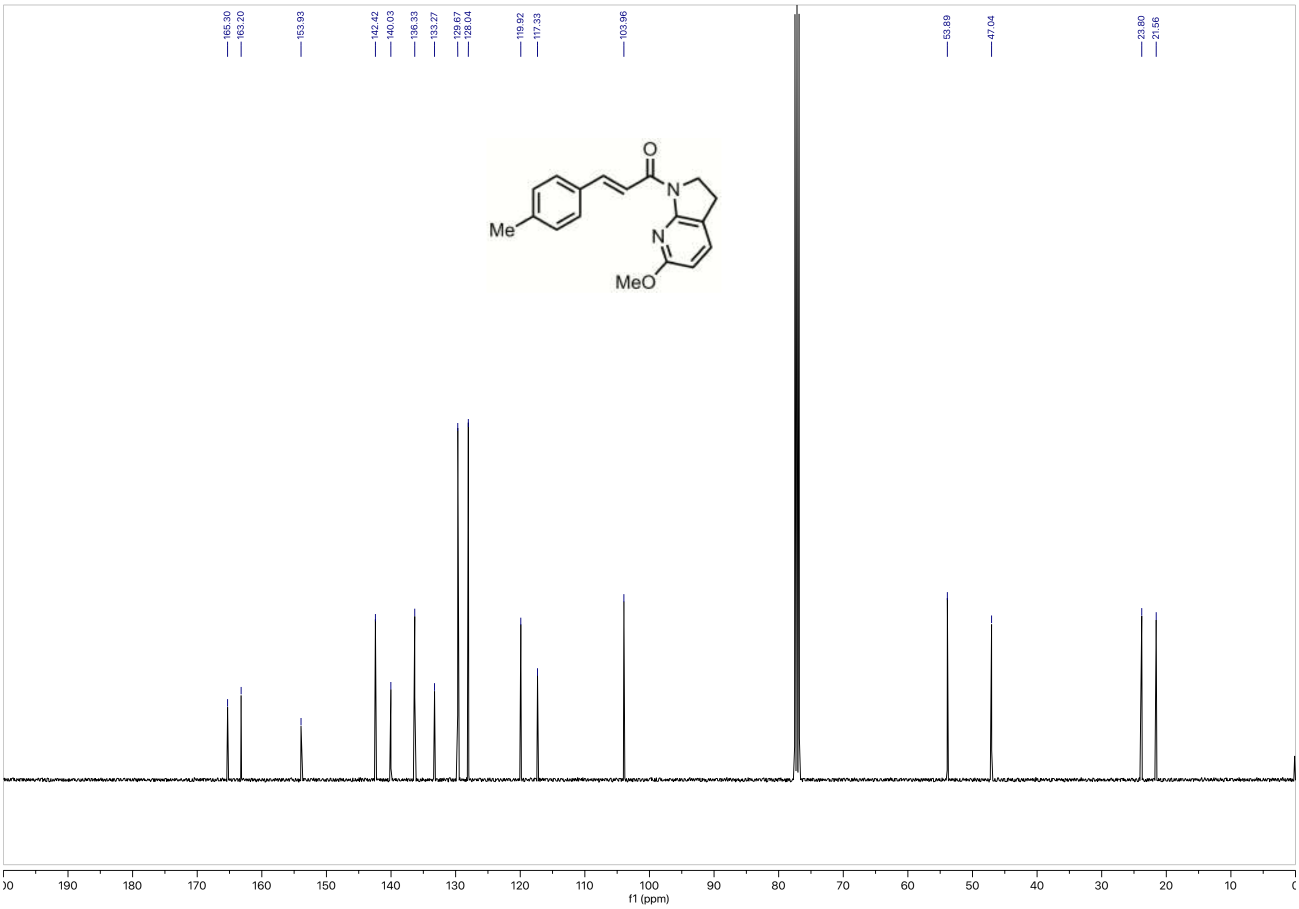

^{1}H NMR: **1t**

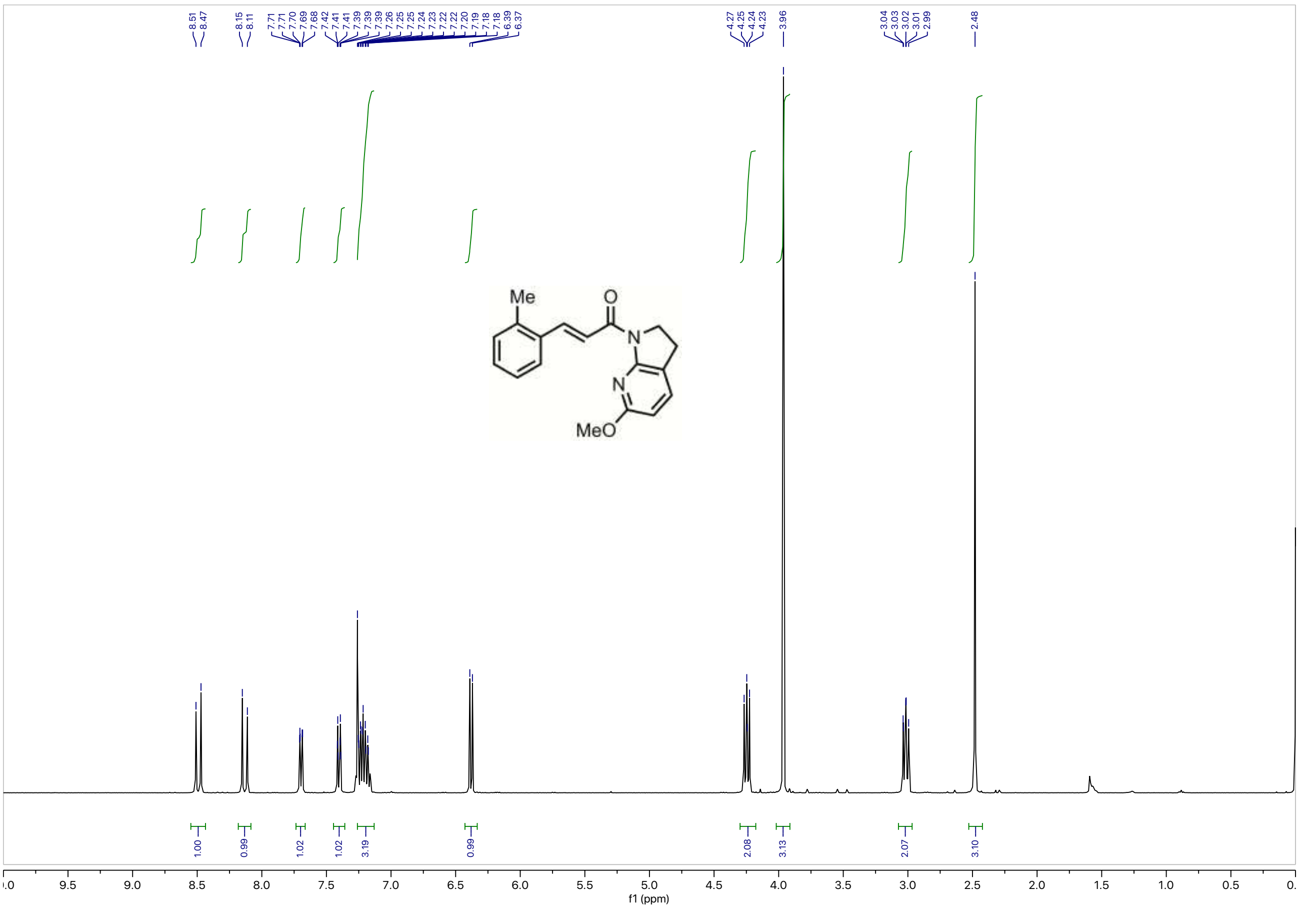

^{13}C NMR: **1t**

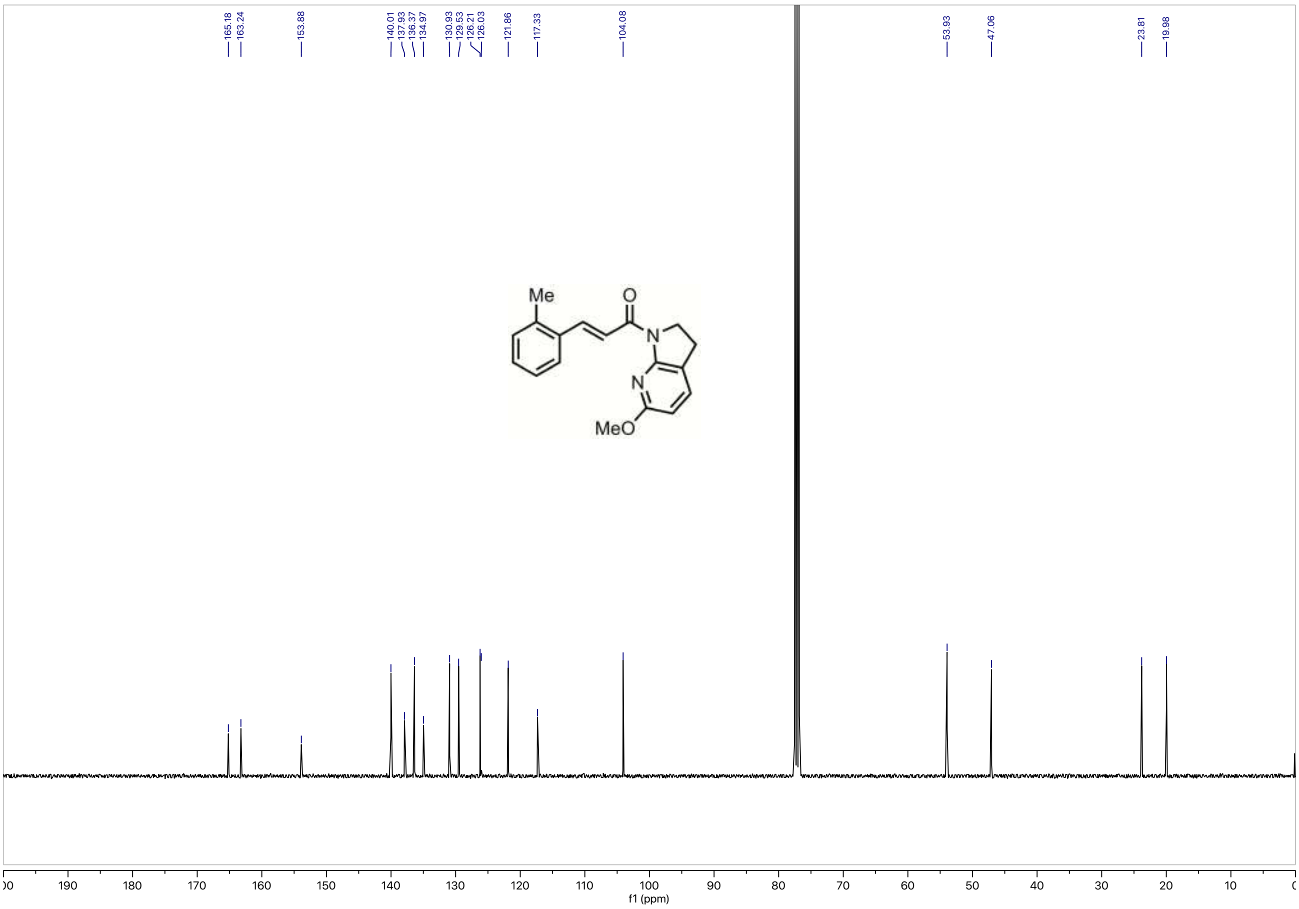

^{1}H NMR: **1u**

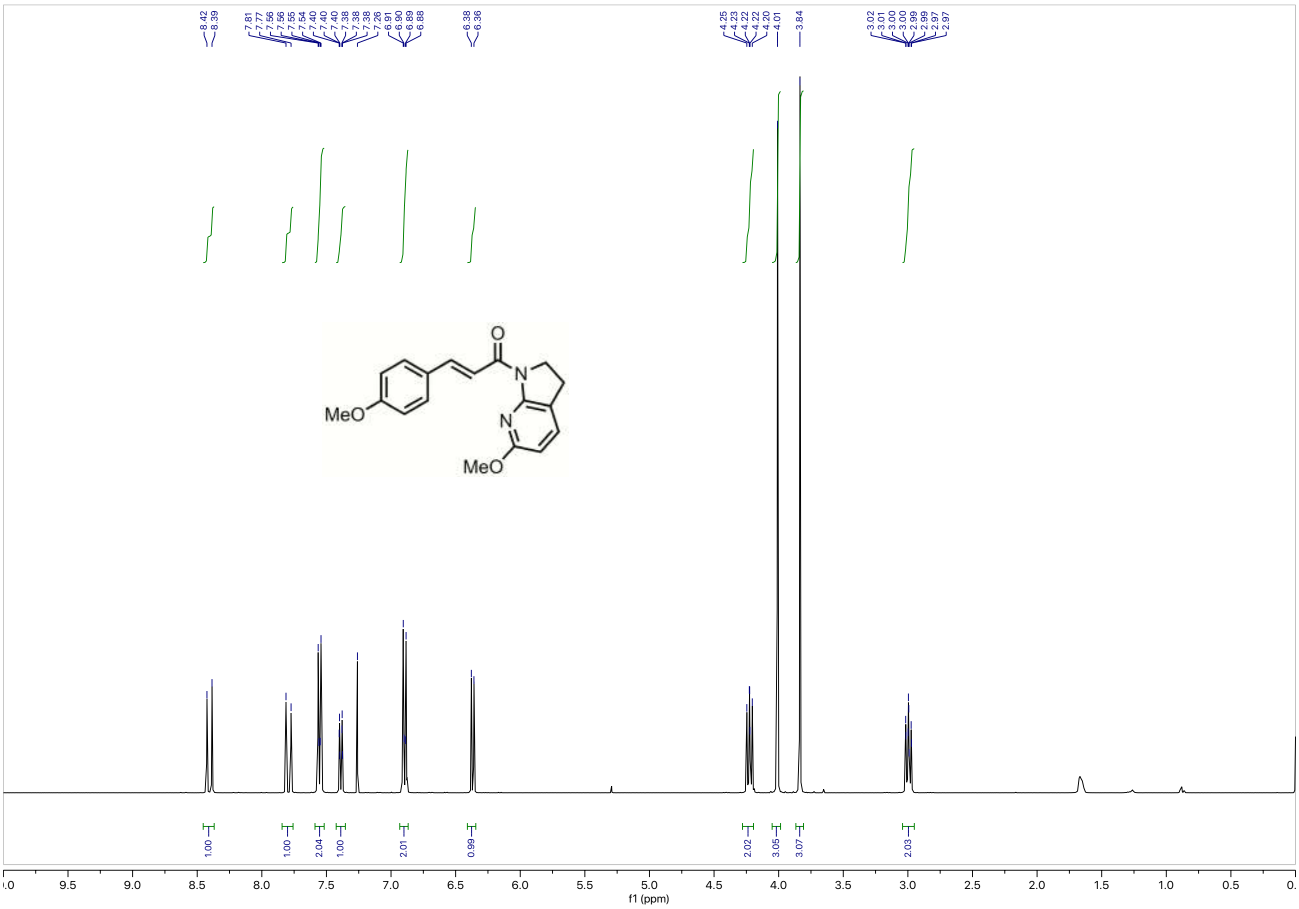

^{13}C NMR: **1u**

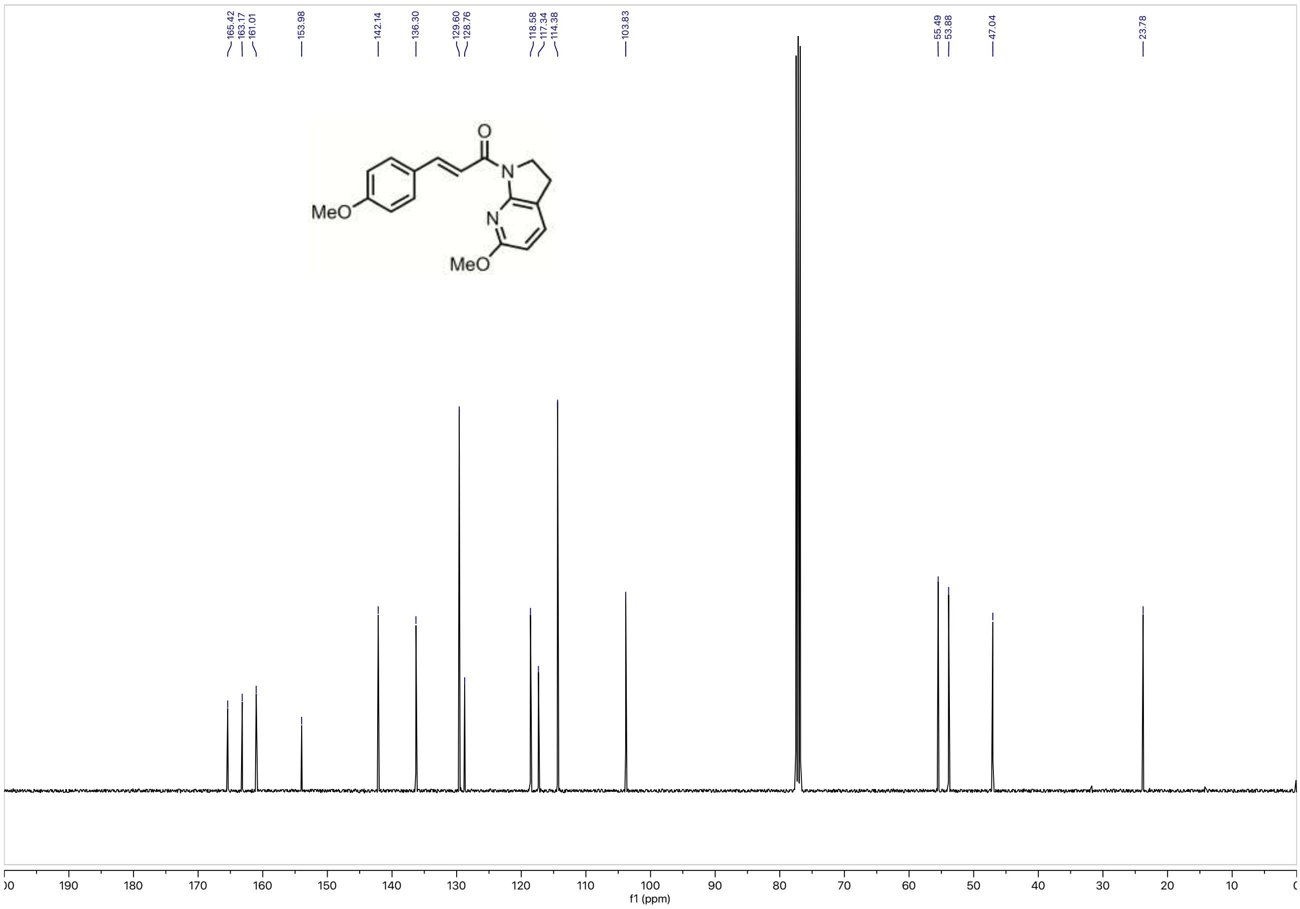

^{1}H NMR: **1v**

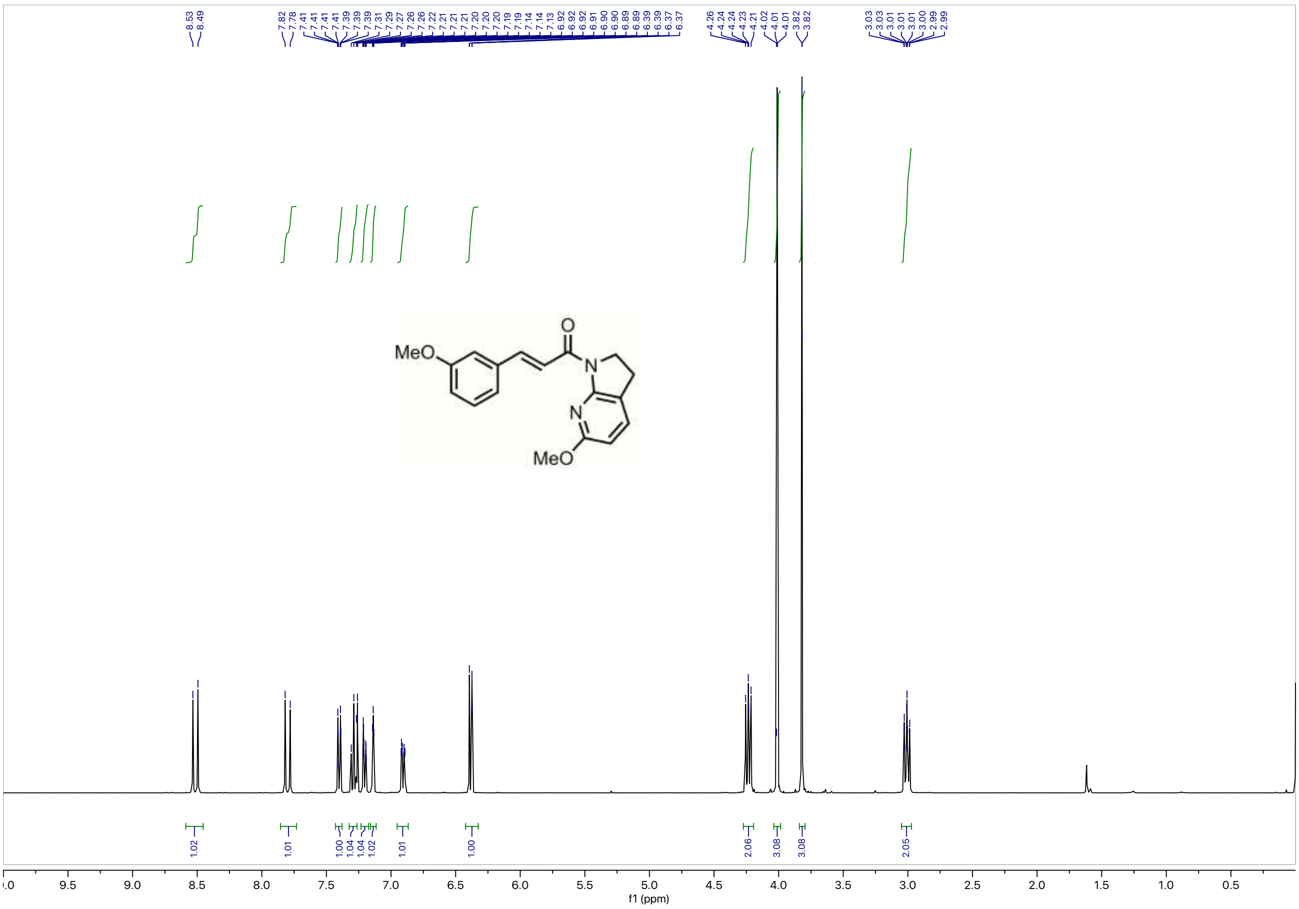

^{13}C NMR: **1v**

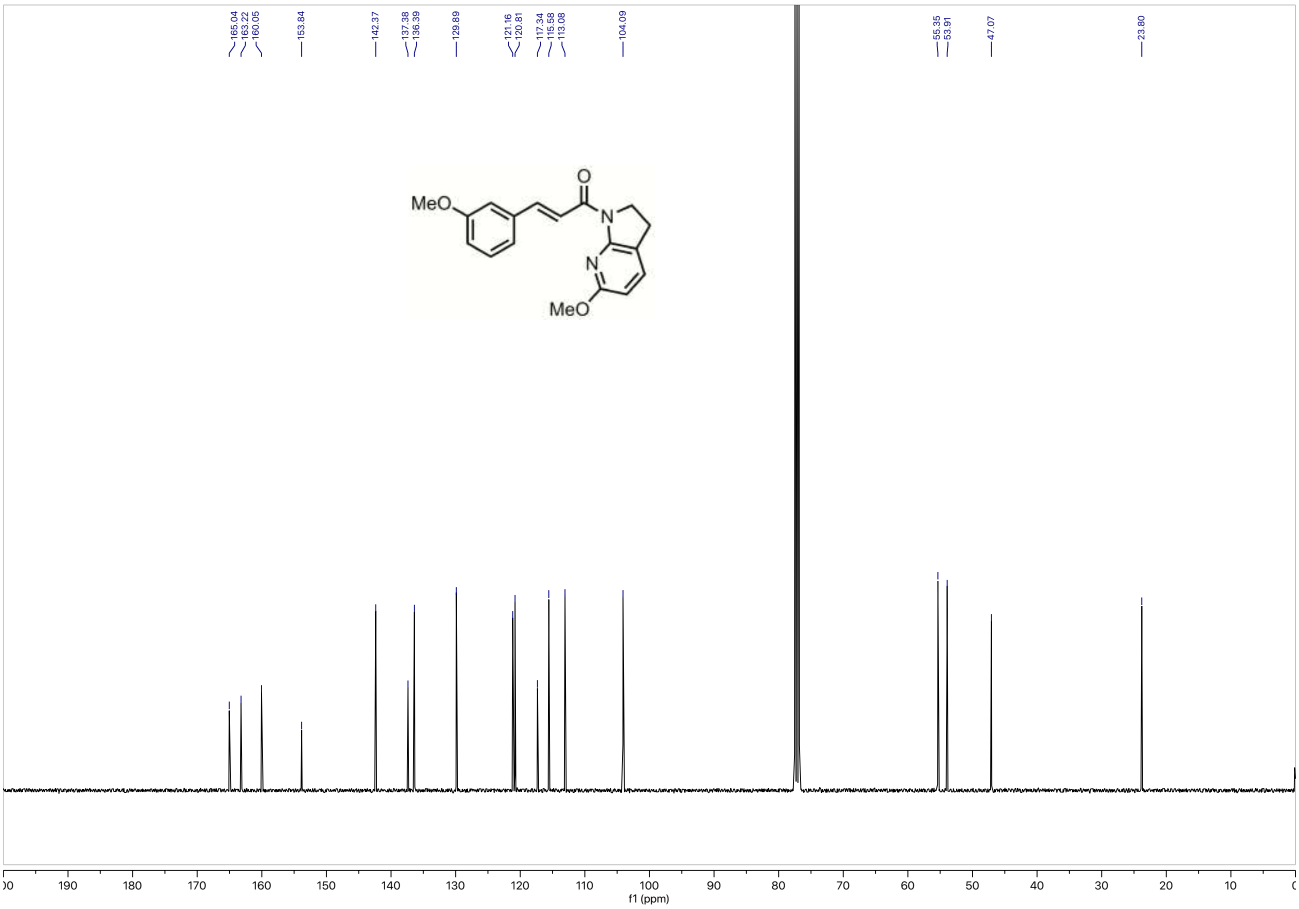

^{1}H NMR: **1w**

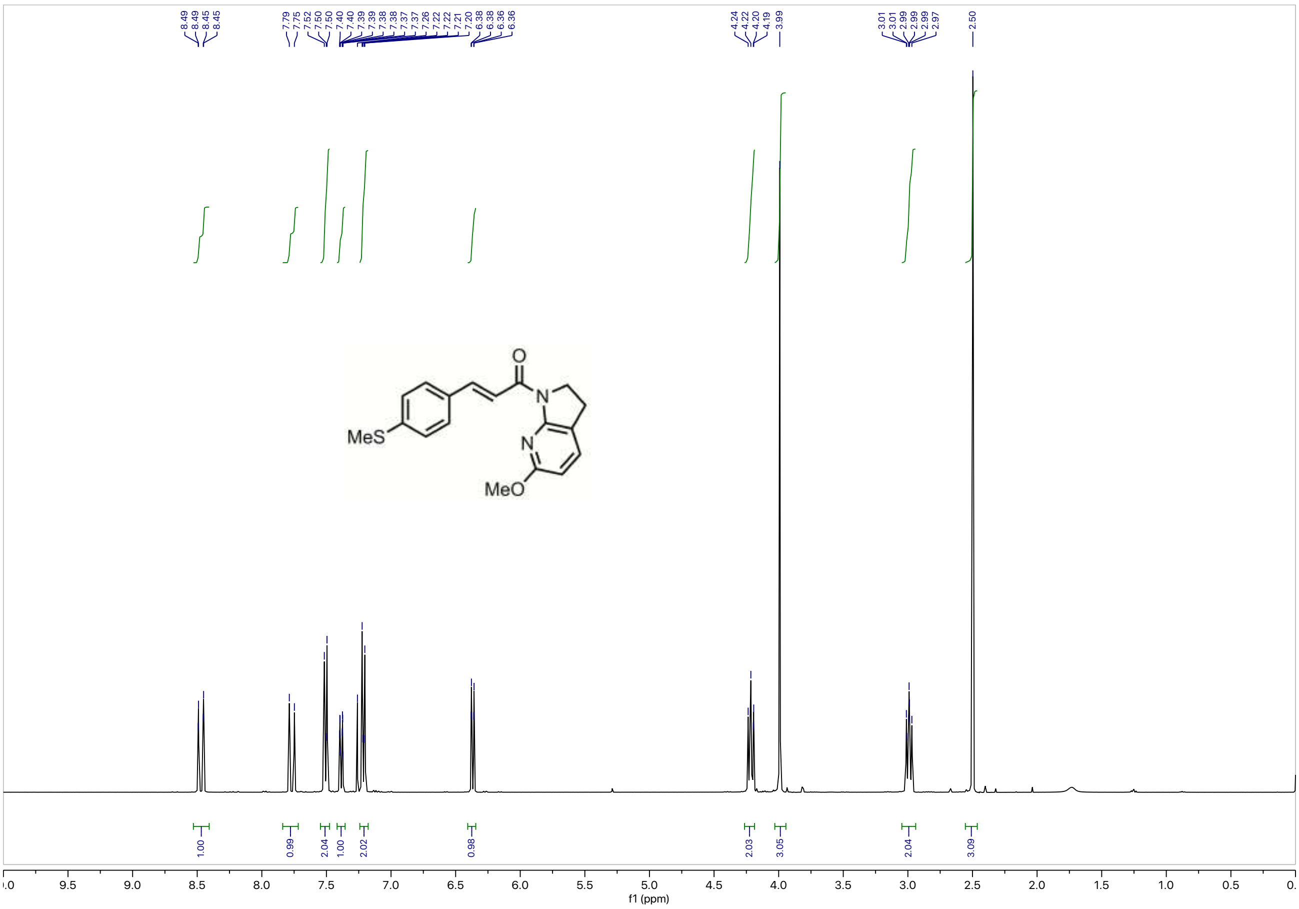

^{13}C NMR: **1w**

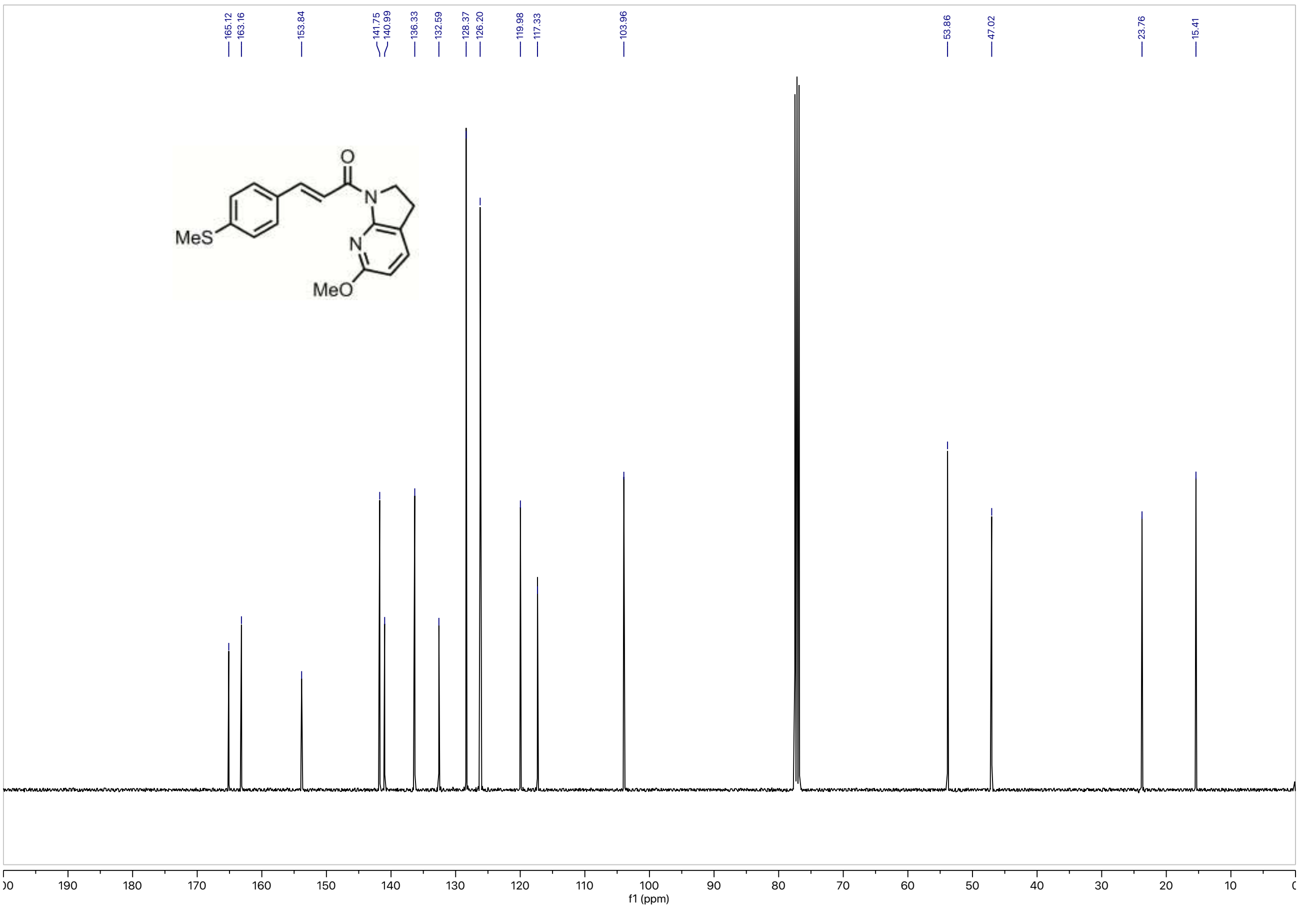

^{1}H NMR: **1x**

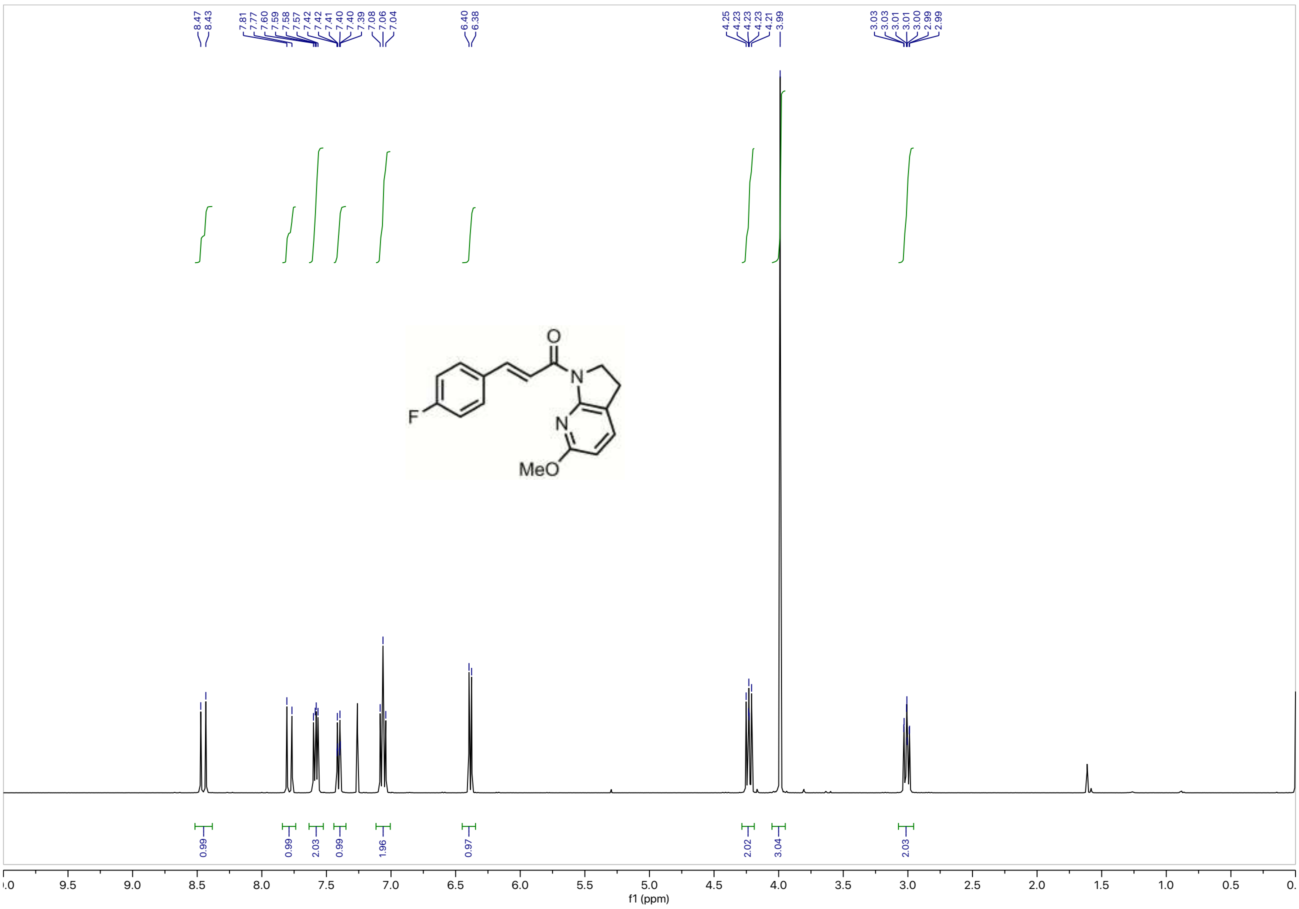

^{13}C NMR: **1x**

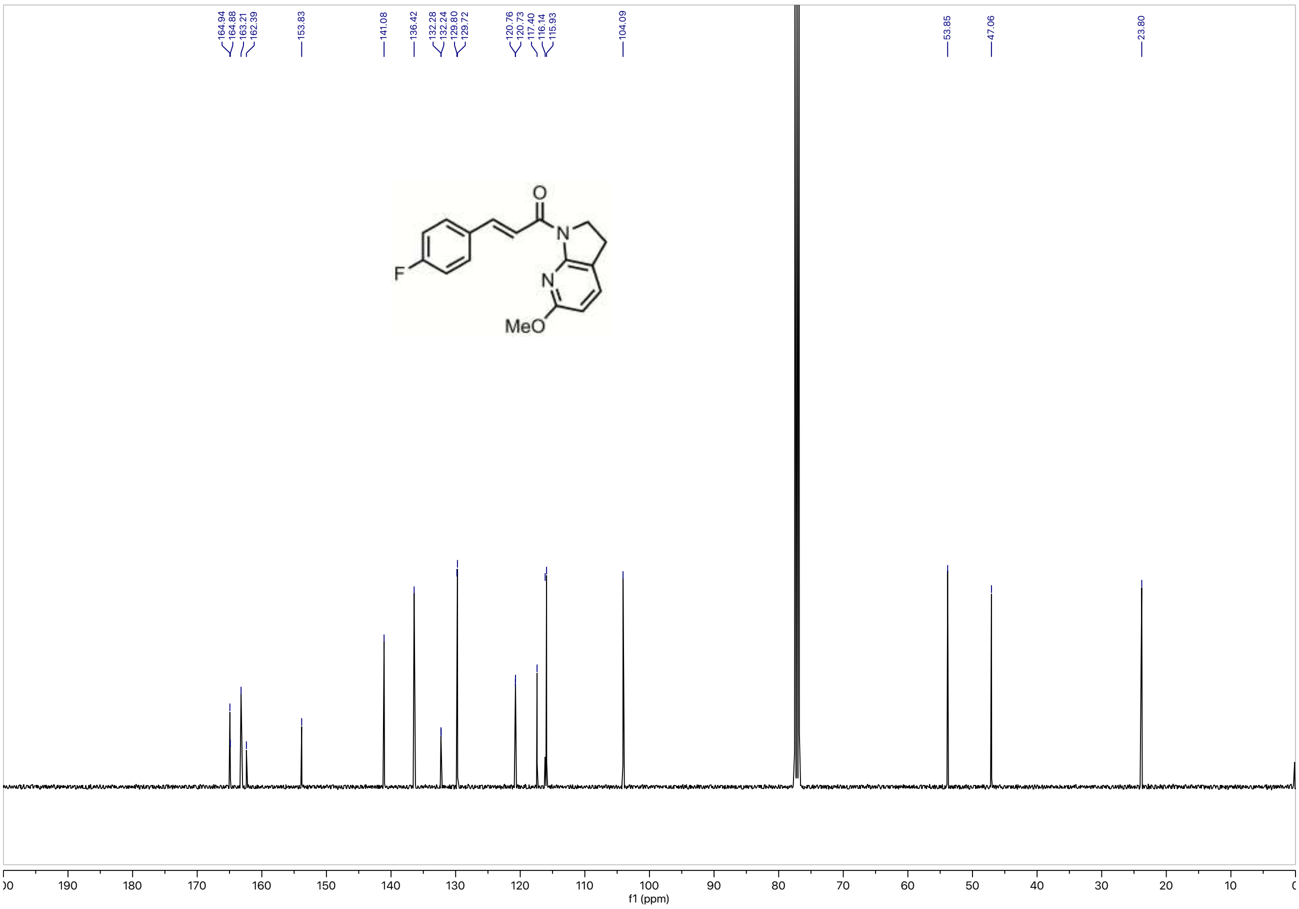

^{19}F NMR: **1x**

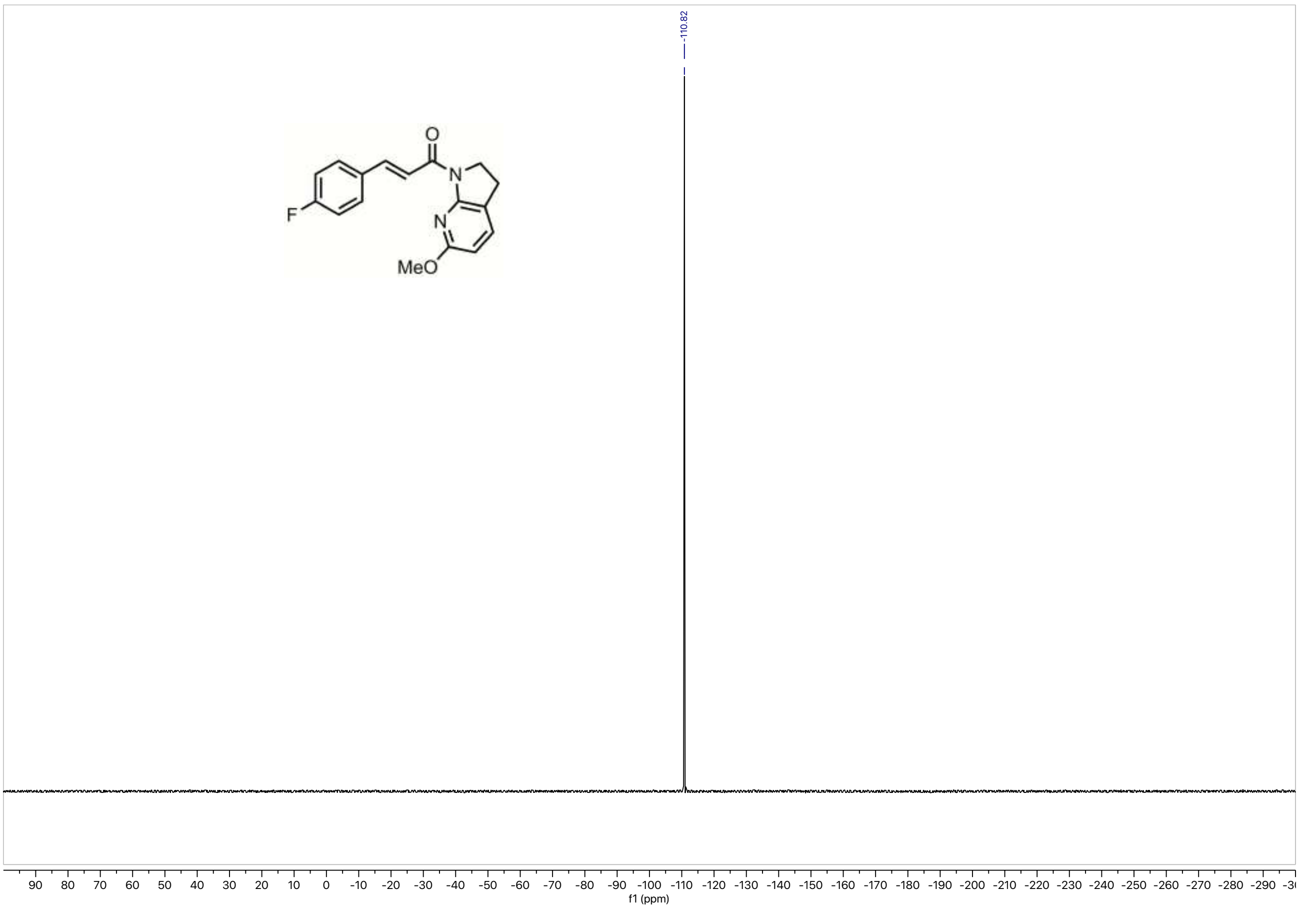
-110.82
F
N
O
N
MeO
90 80 70 60 50 40 30 20 10 0 -10 -20 -30 -40 -50 -60 -70 -80 -90 -100 -110 -120 -130 -140 -150 -160 -170 -180 -190 -200 -210 -220 -230 -240 -250 -260 -270 -280 -290
f1 (ppm)

^{1}H NMR: **1y**

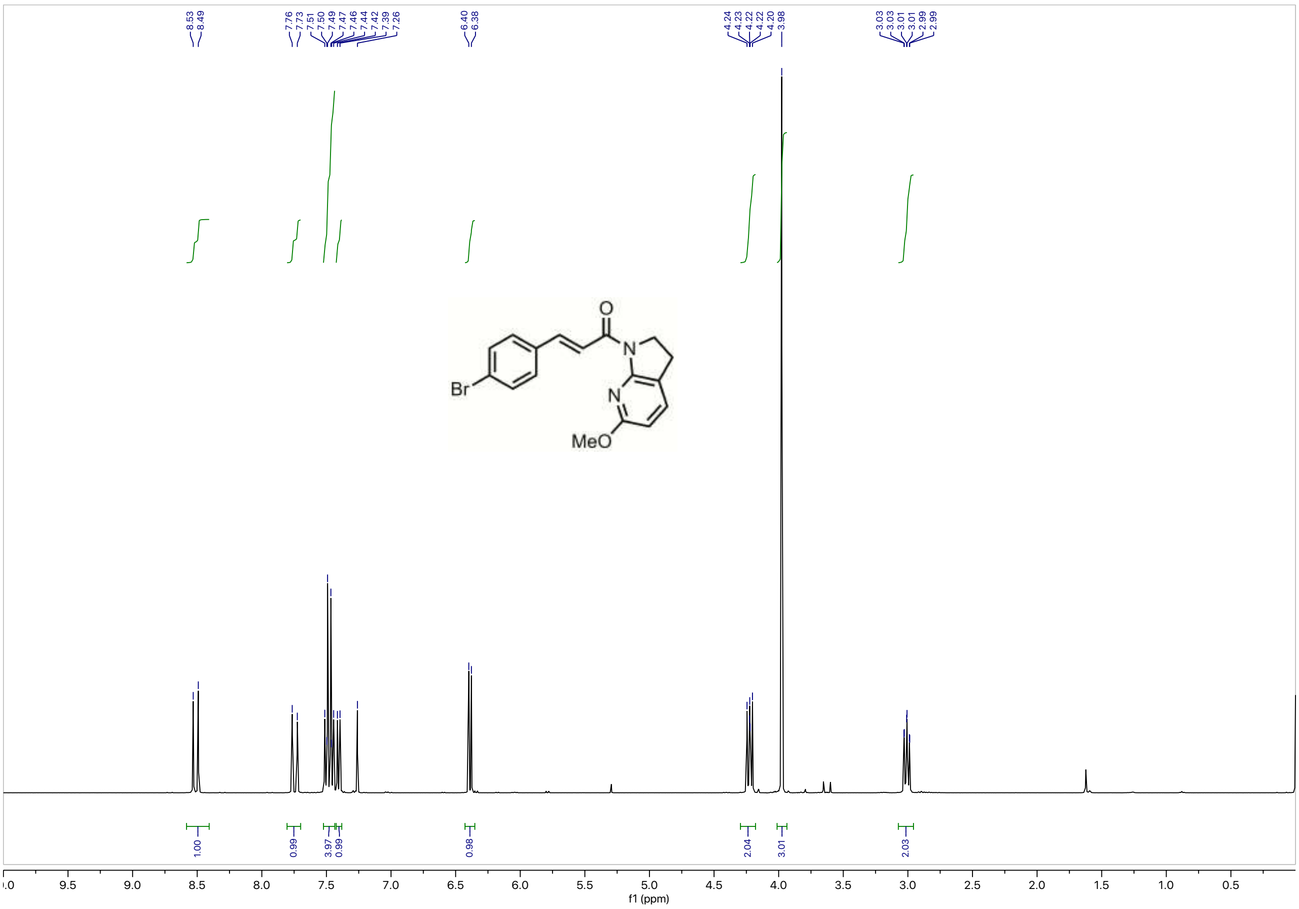

^{13}C NMR: **1y**

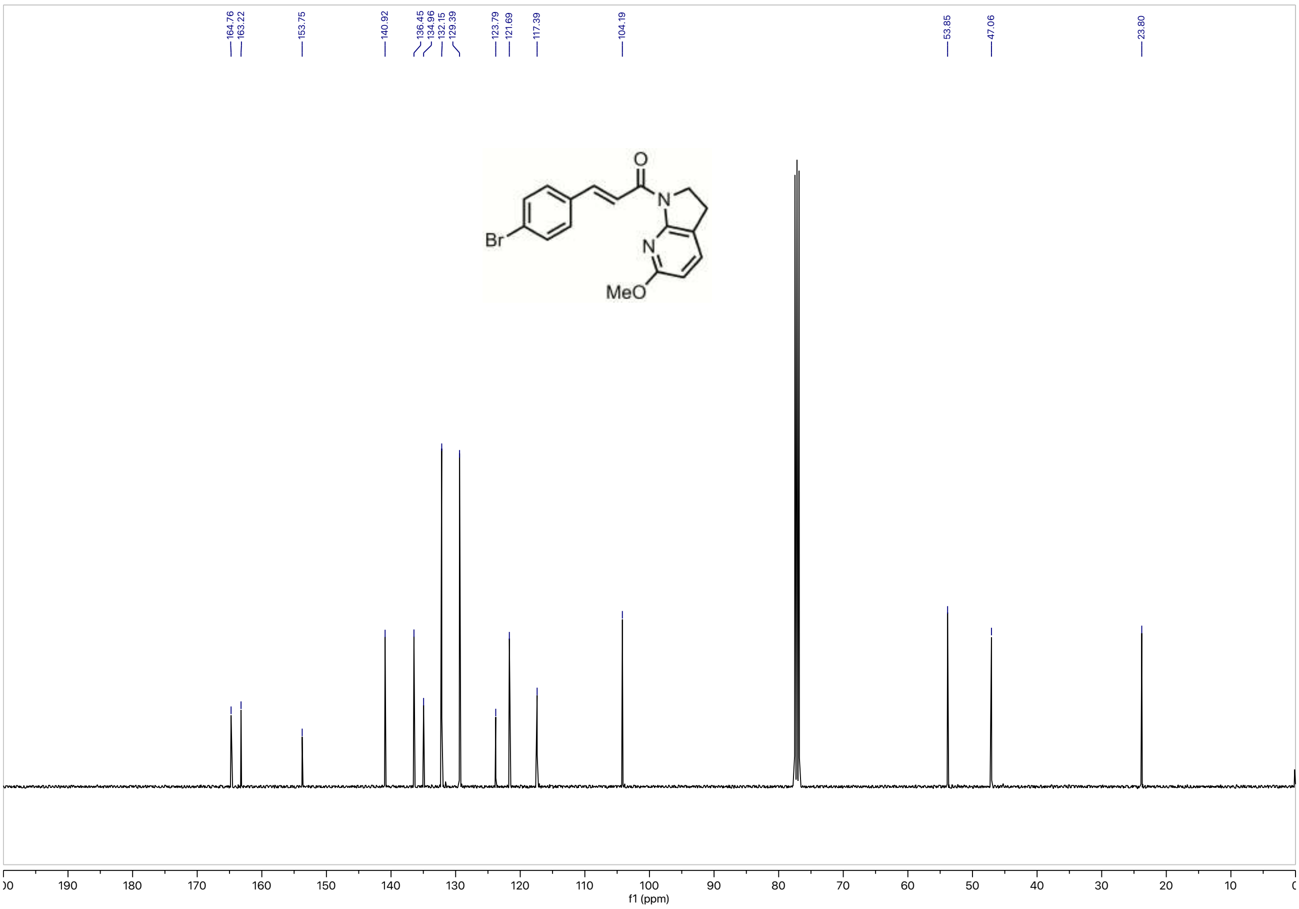

^{1}H NMR: **1z**

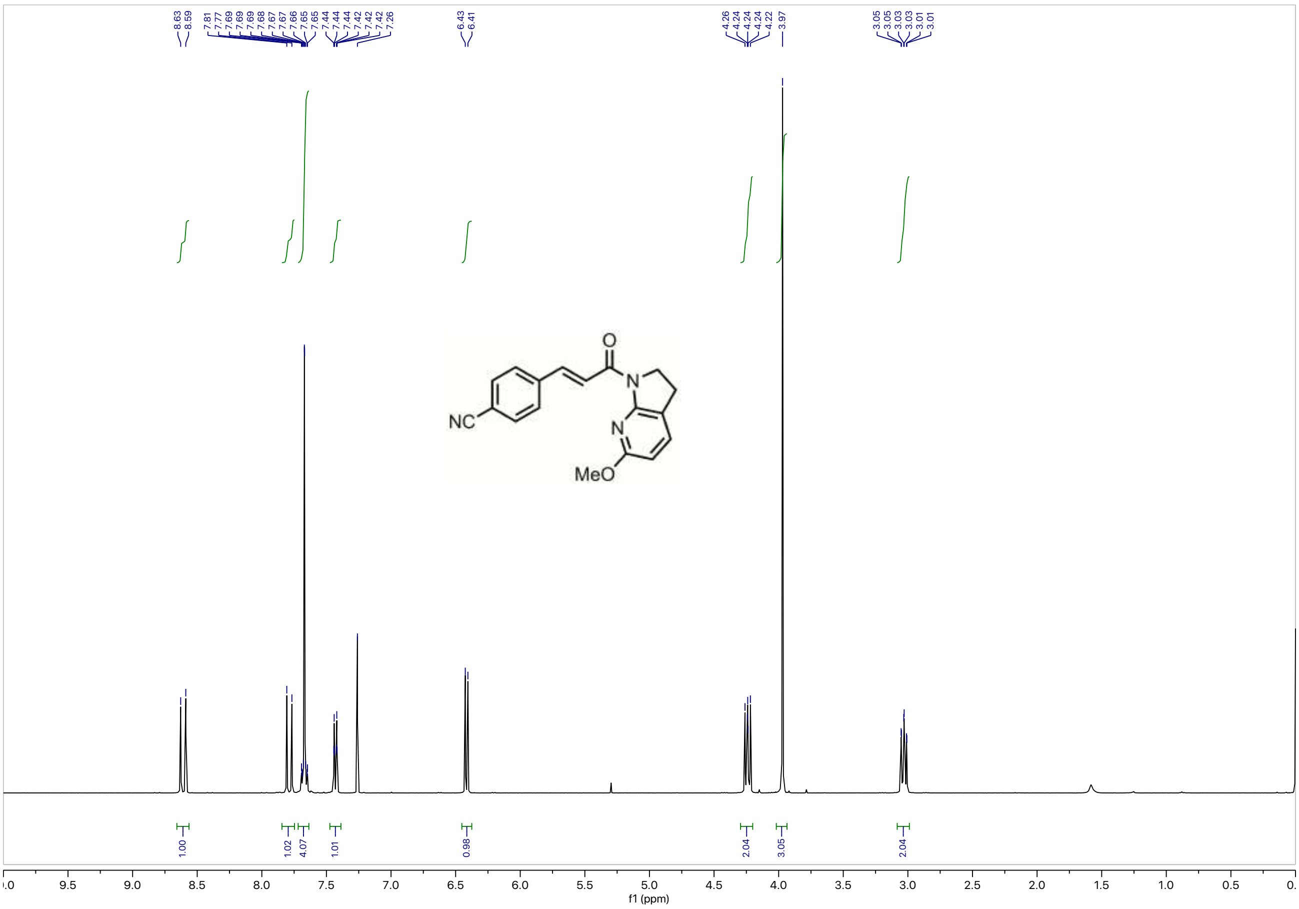

^{13}C NMR: **1z**

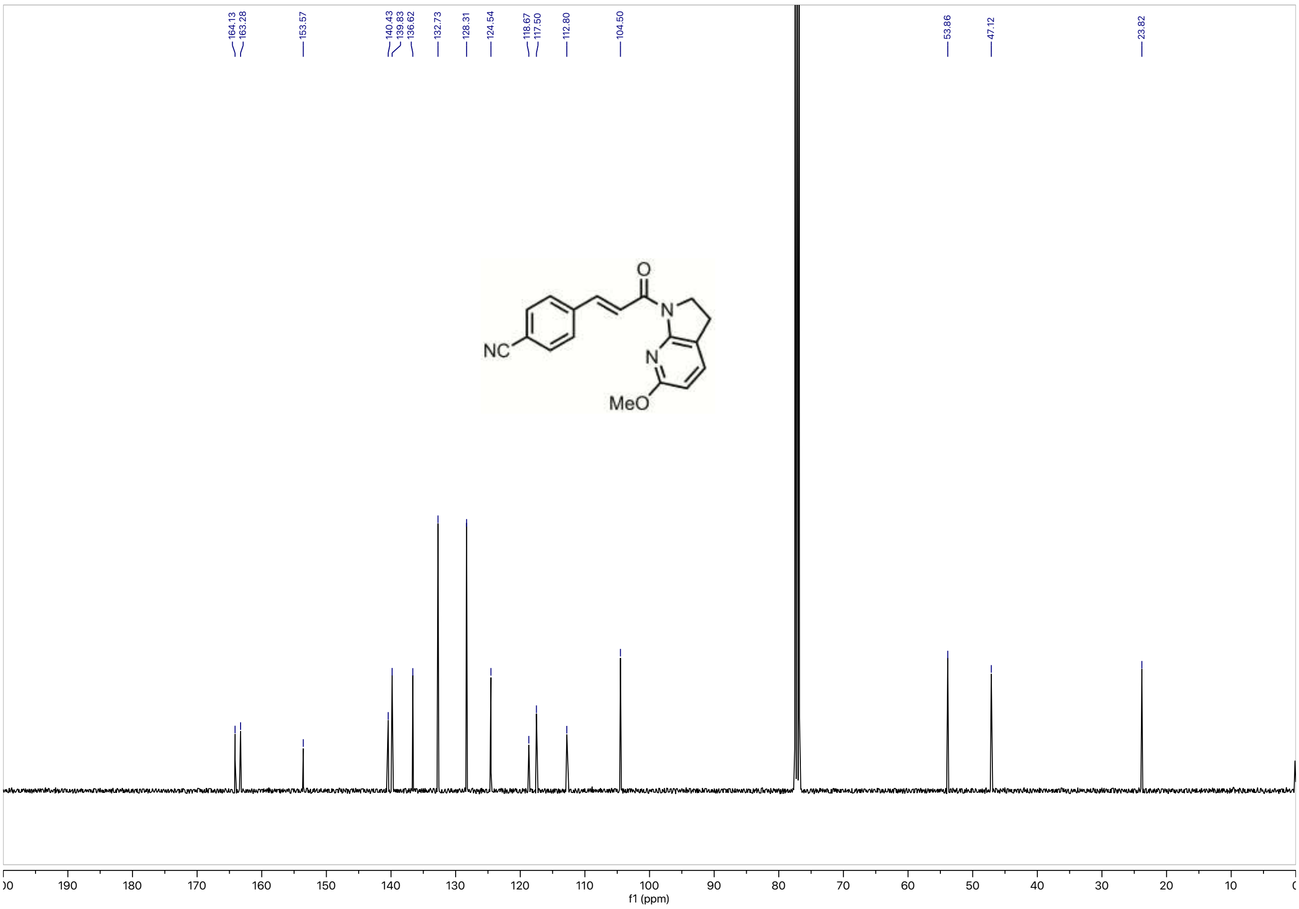

^{1}H NMR: **1aa**

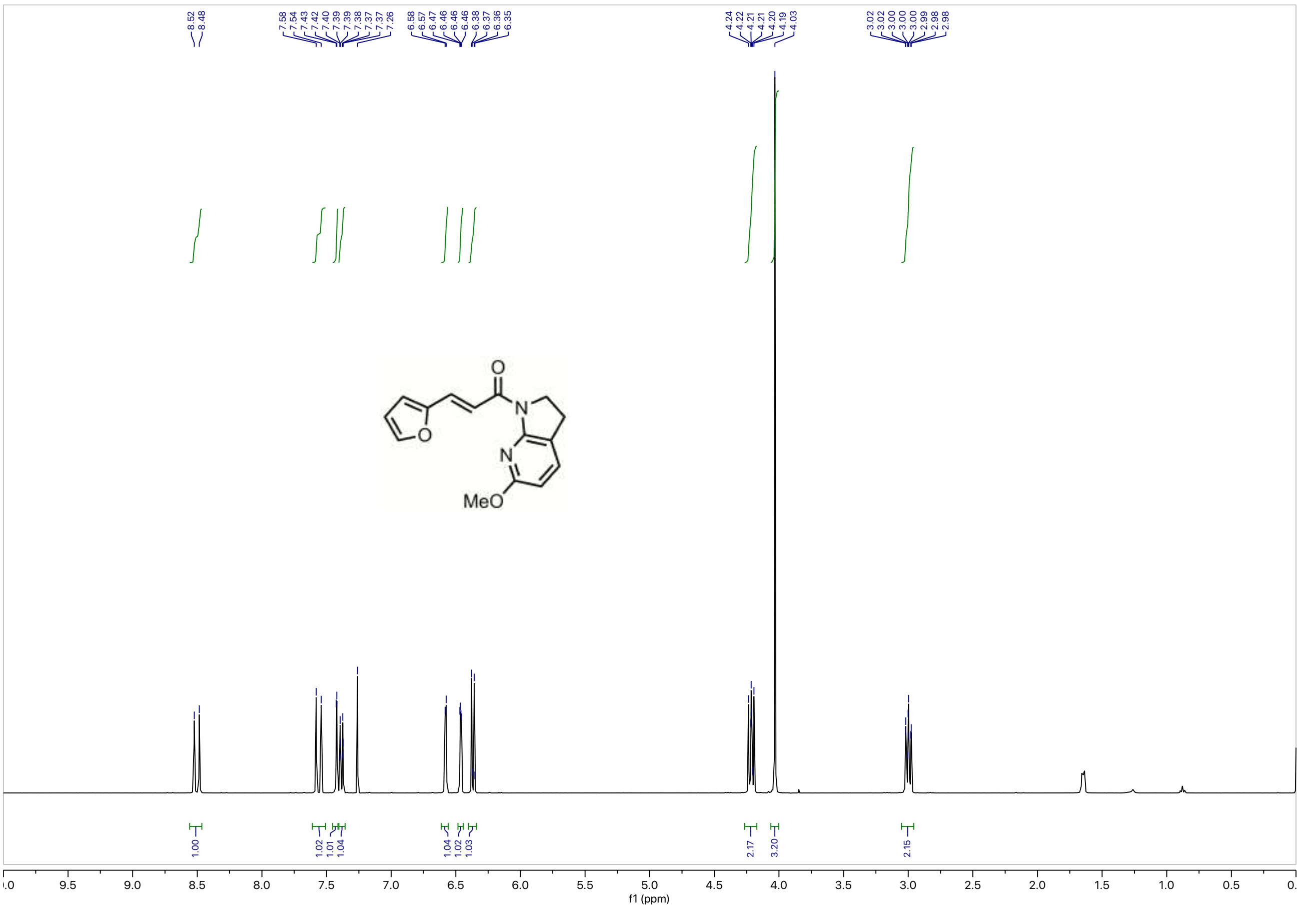

^{13}C NMR: **1aa**

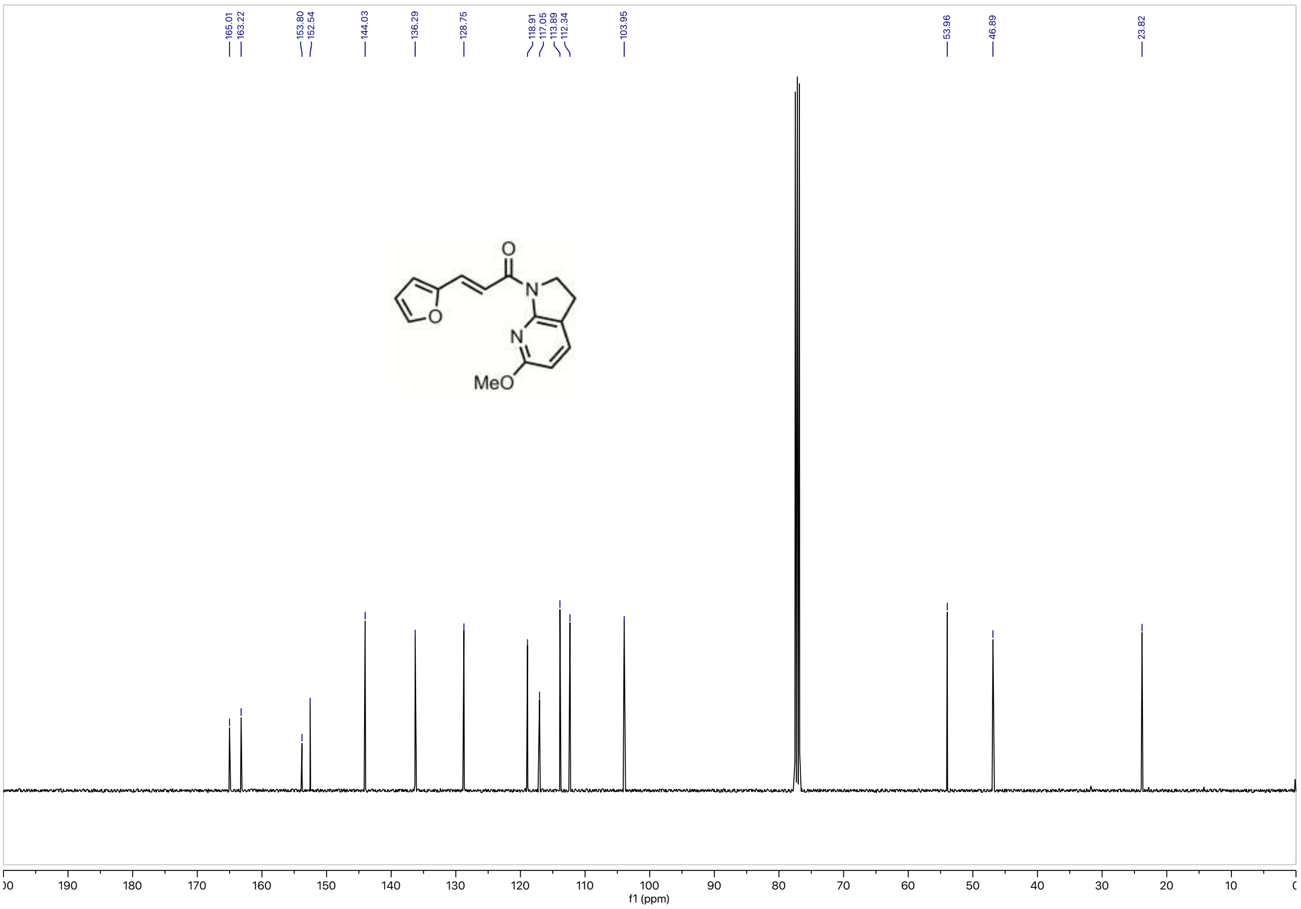

^{1}H NMR: **1ab**

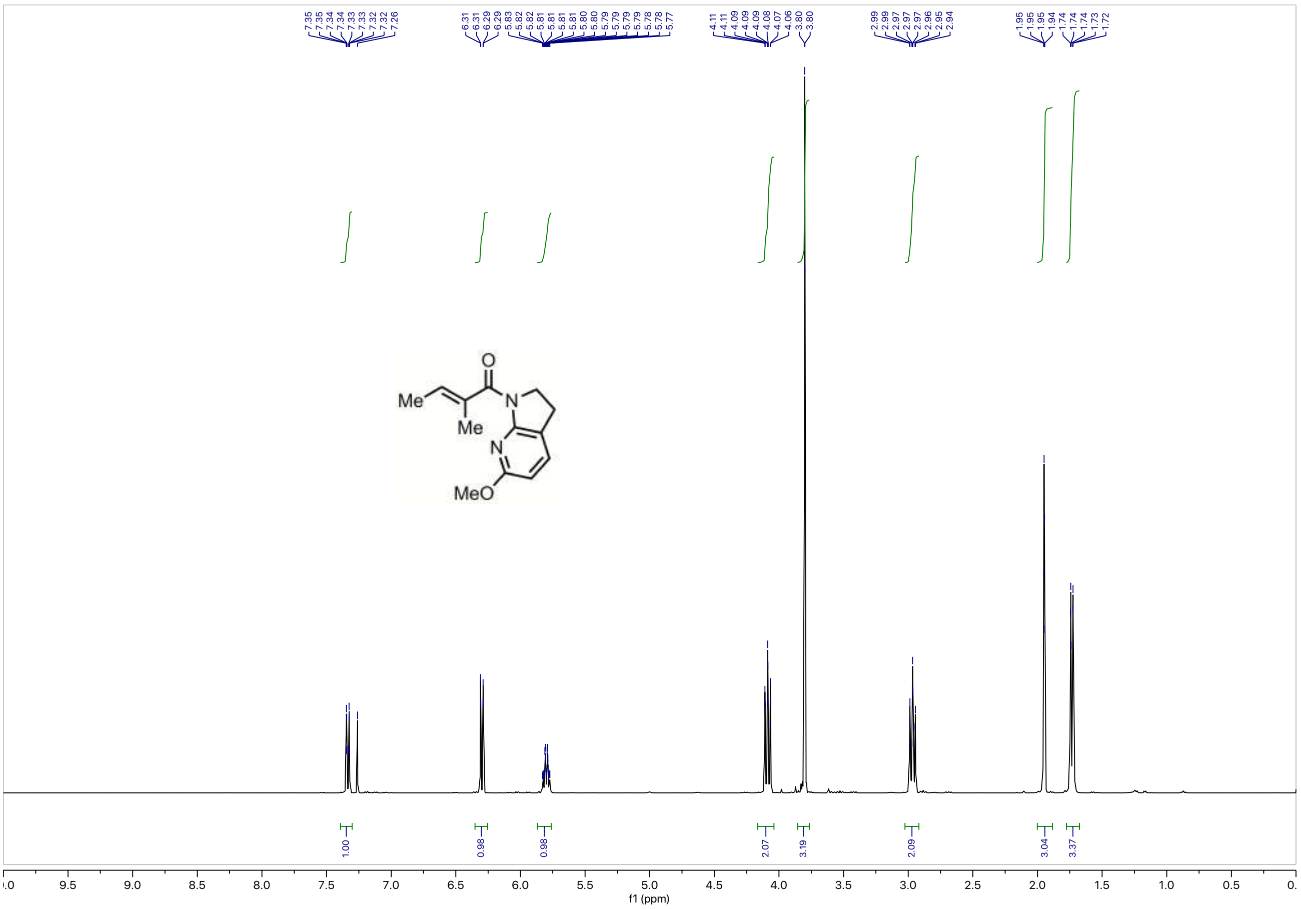

^{13}C NMR: **1ab**

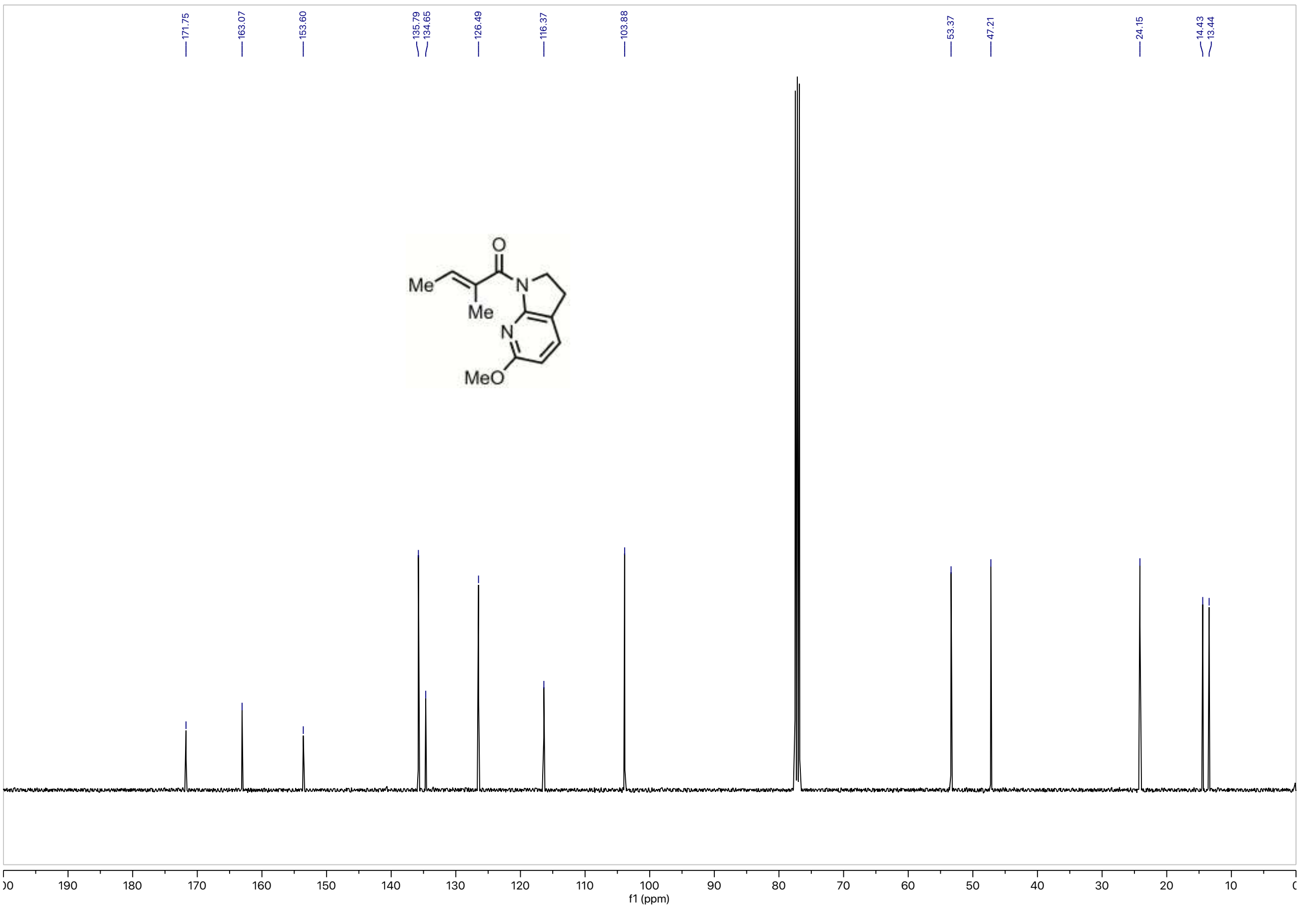

^{1}H NMR: **4**

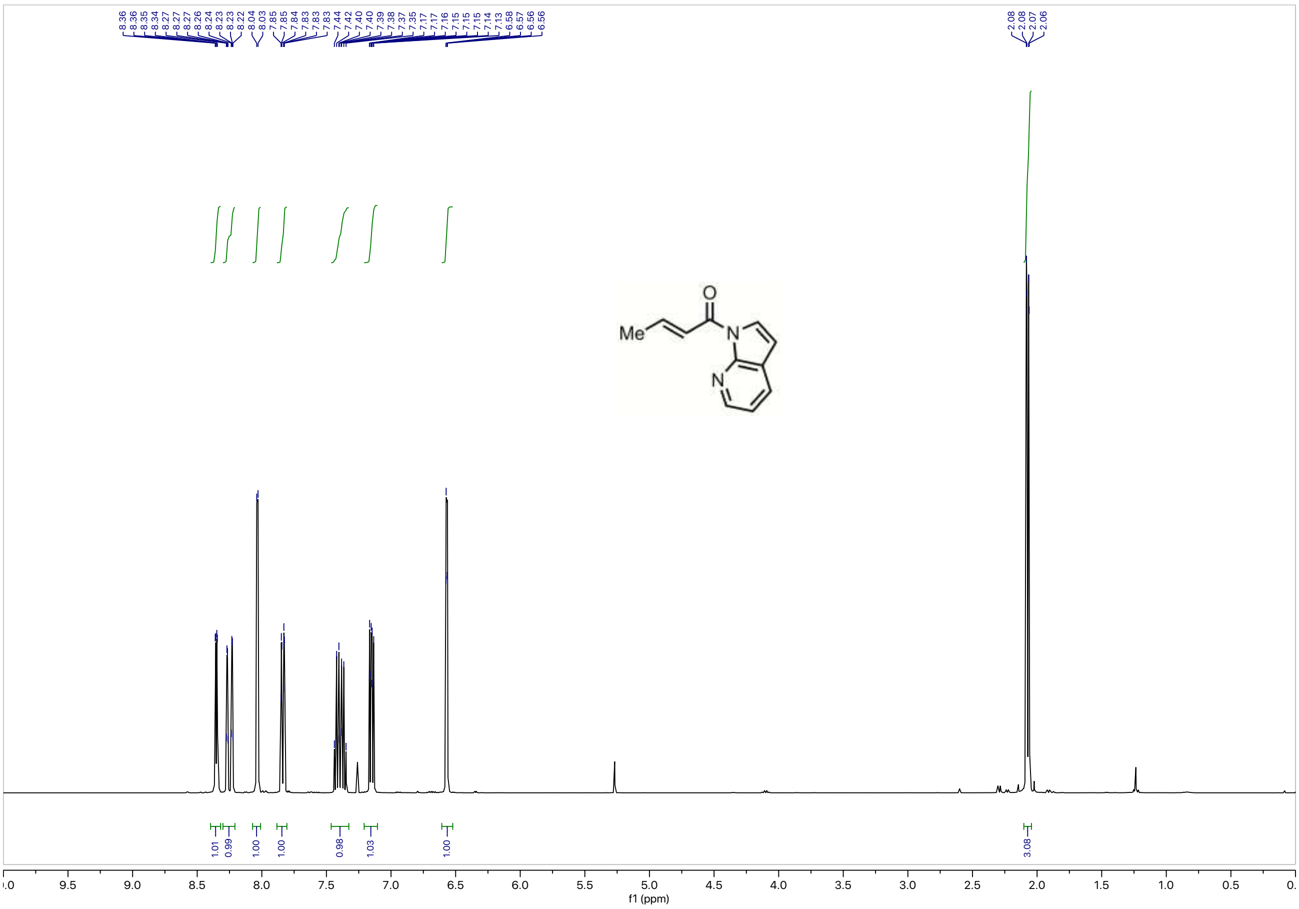

^{13}C NMR: **4**

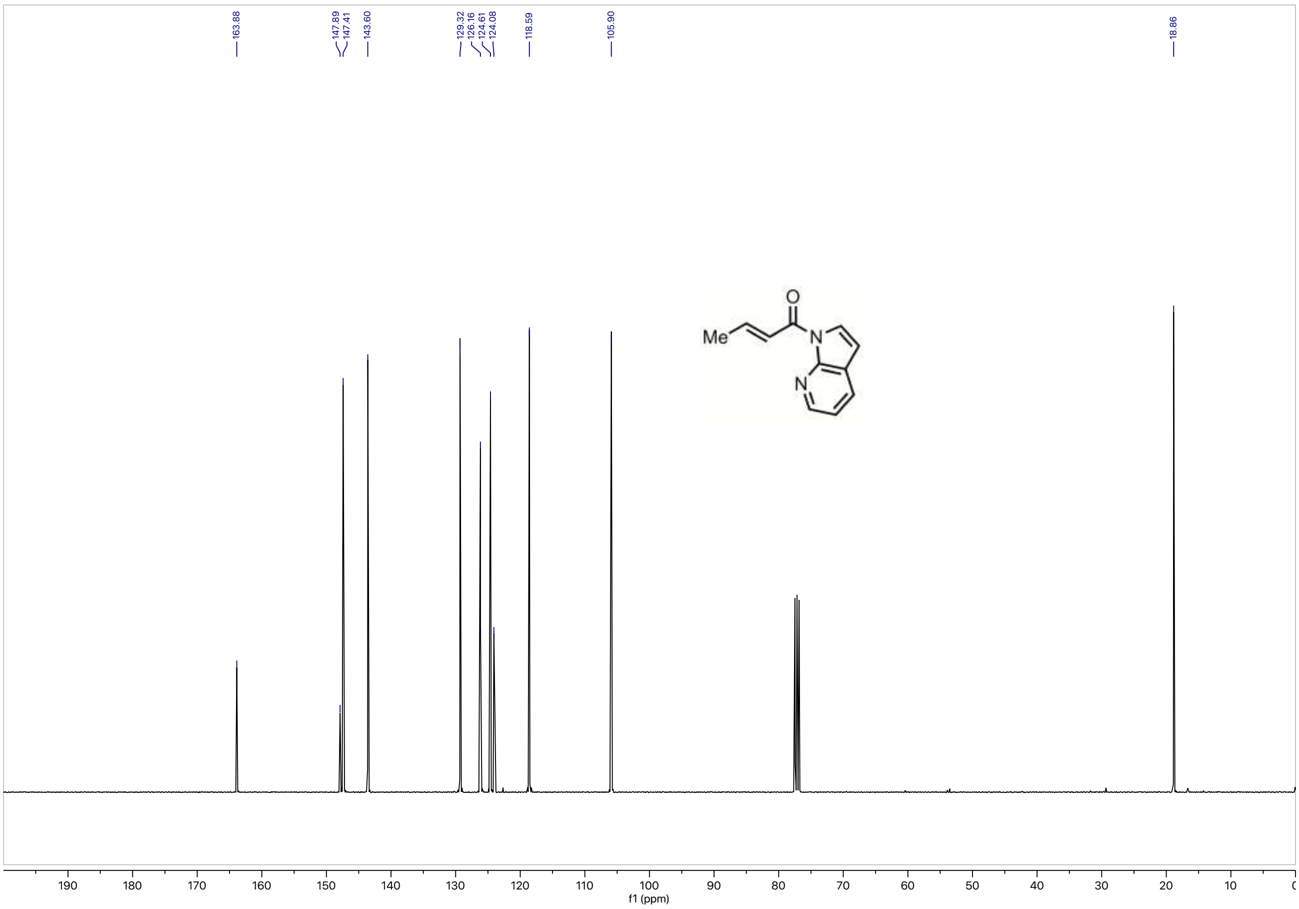

^{1}H NMR: **5** (DMSO-d_6)

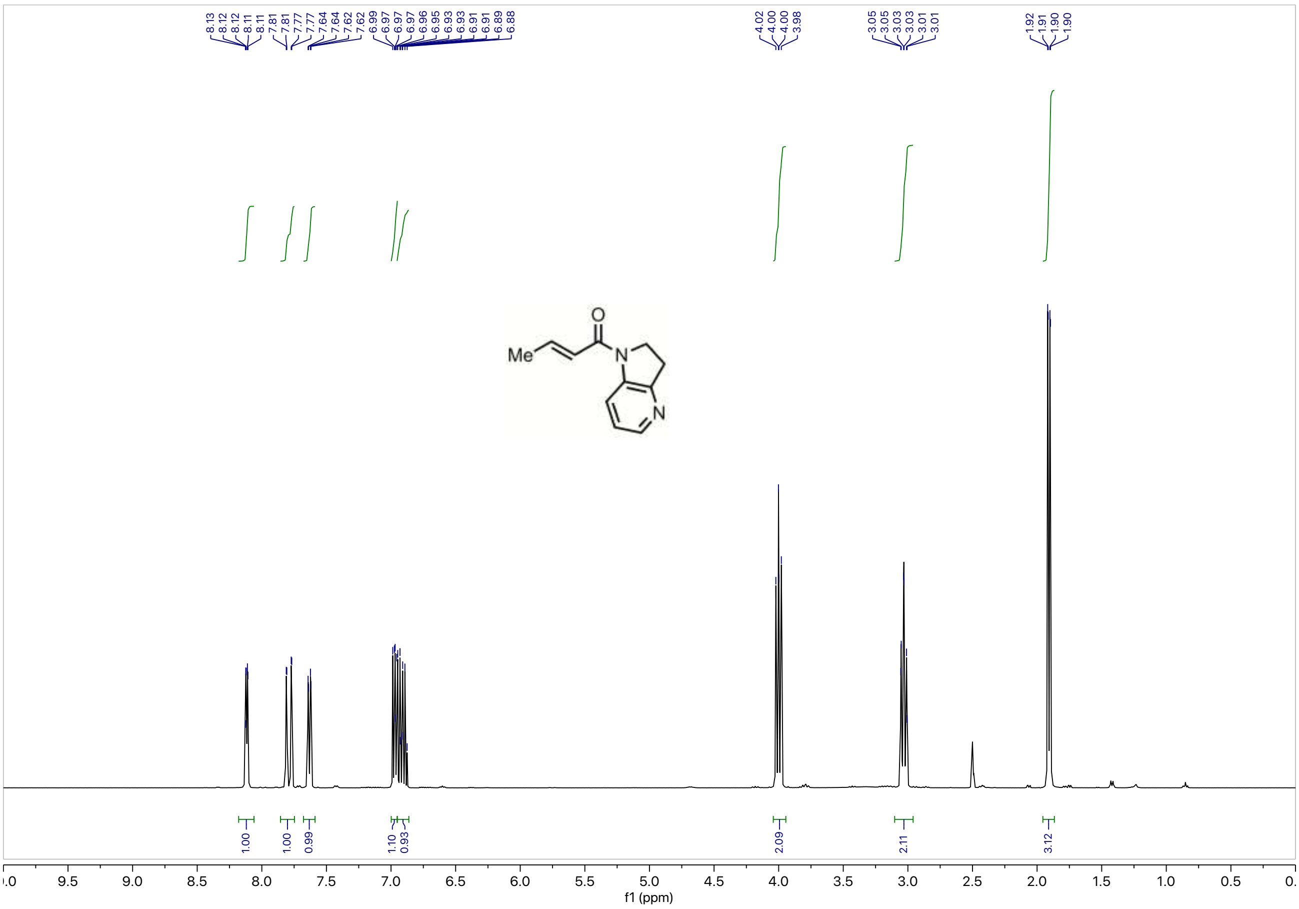

^{13}C NMR: **5** (DMSO-d_6)

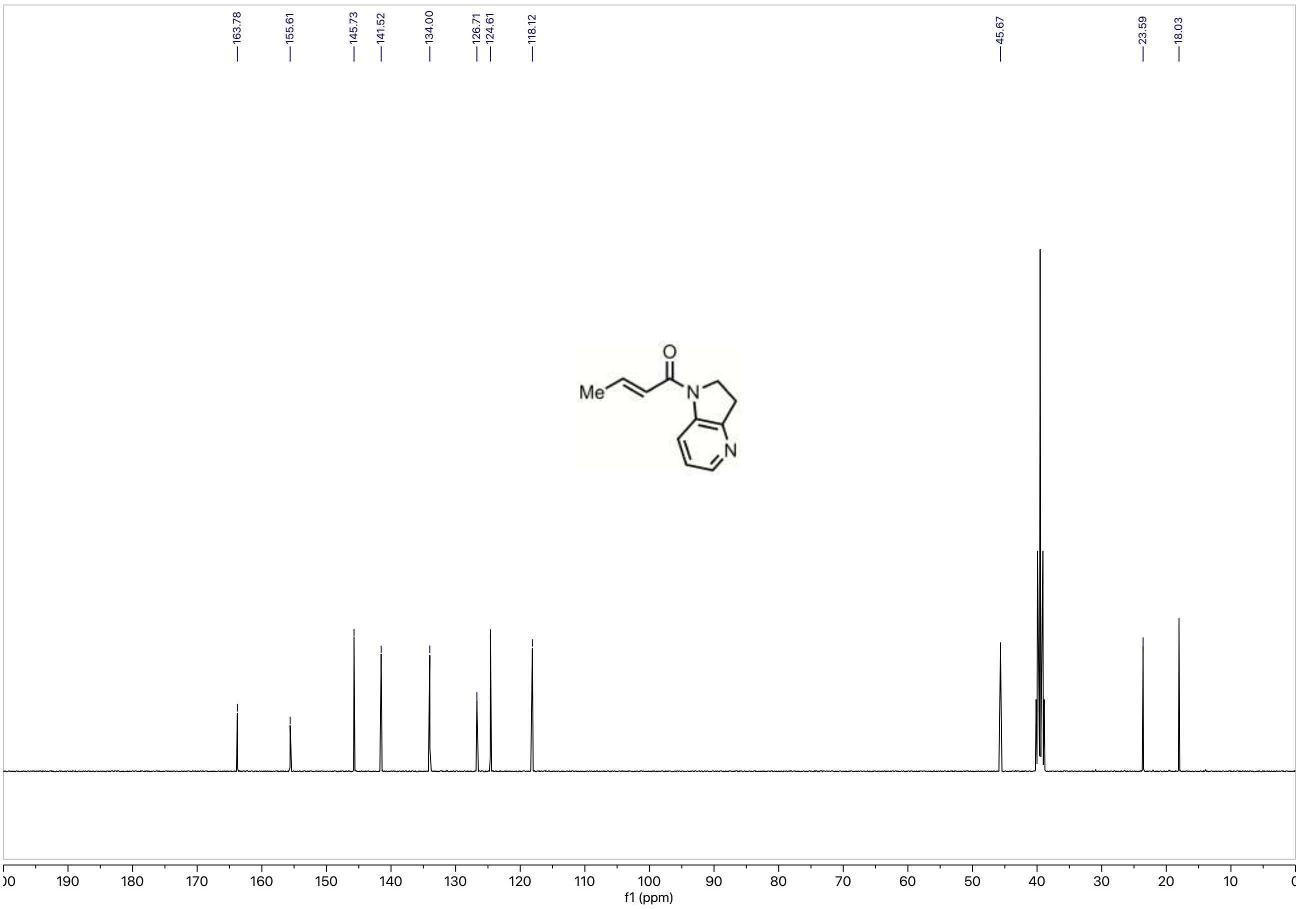

^{1}H NMR: **6**

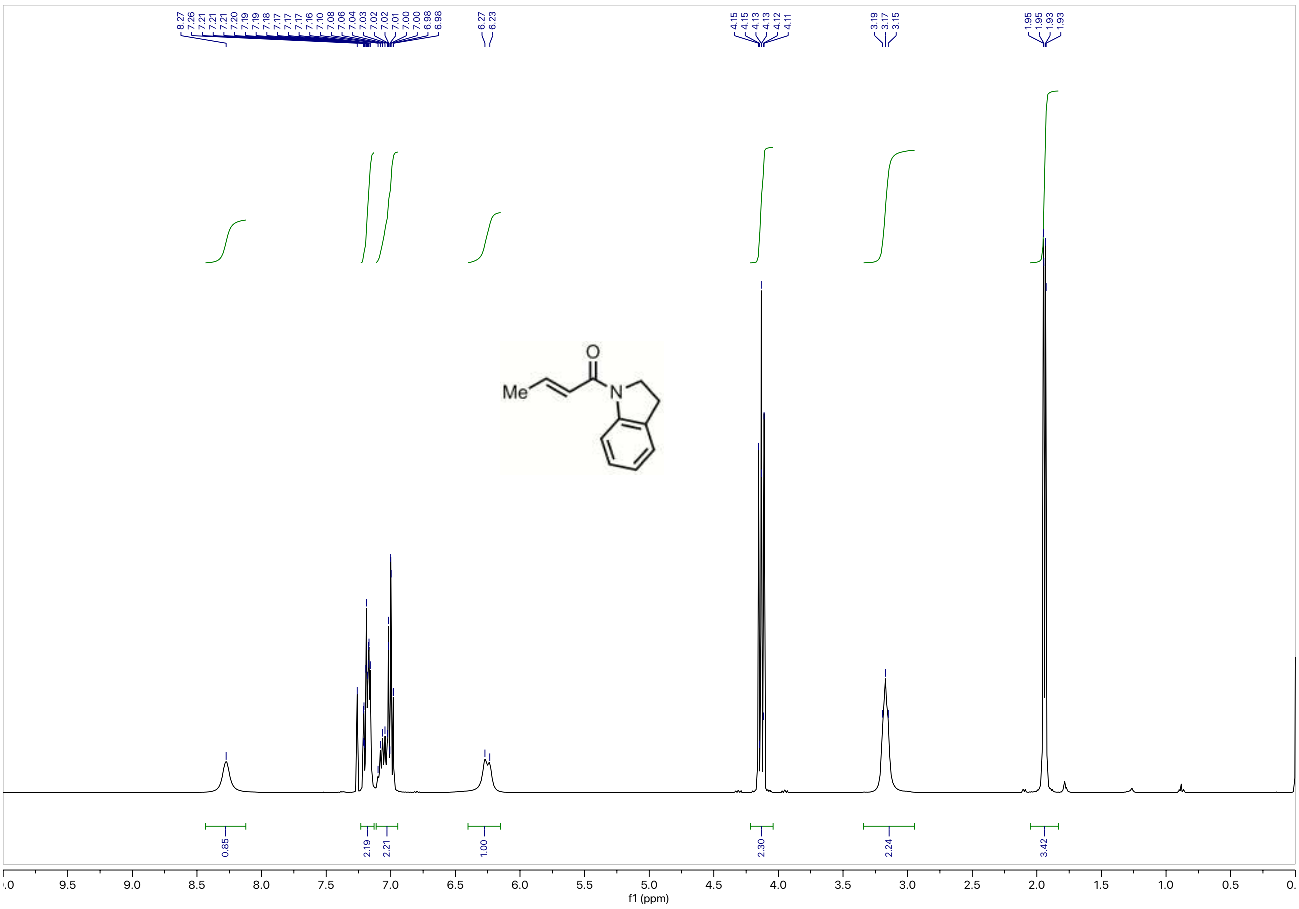

^{13}C NMR: **6**

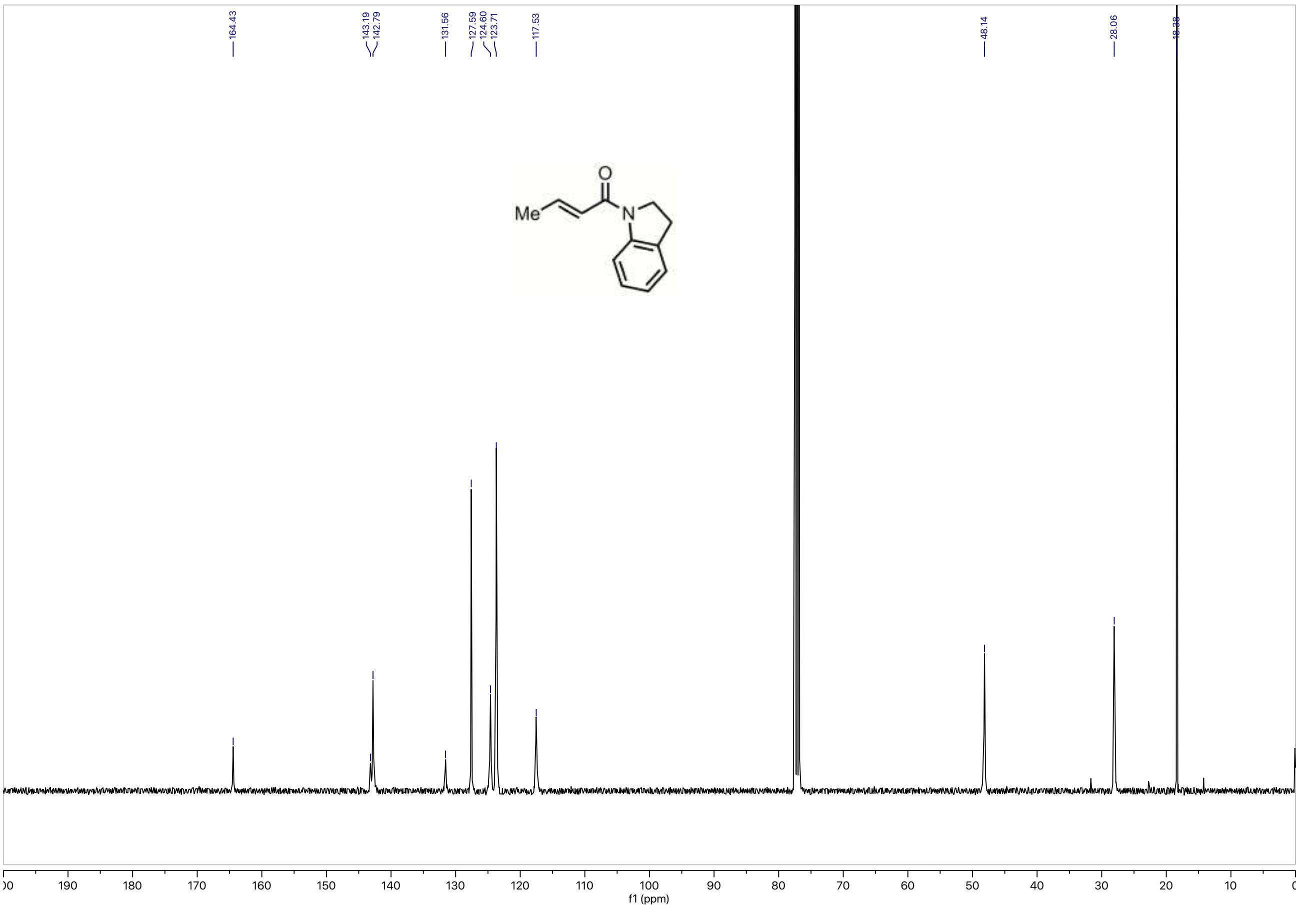

^{1}H NMR: **7**

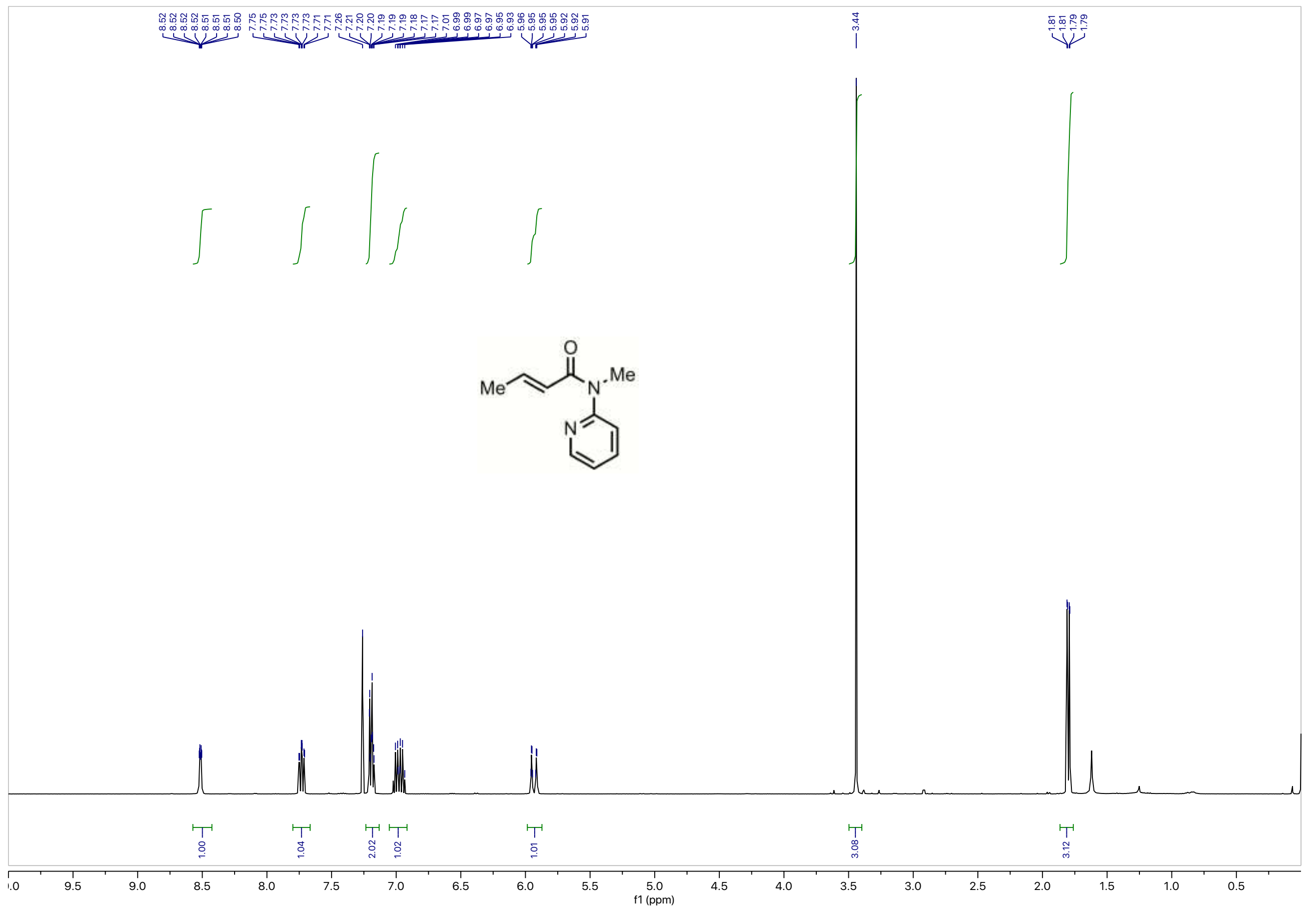

^{13}C NMR: **7**

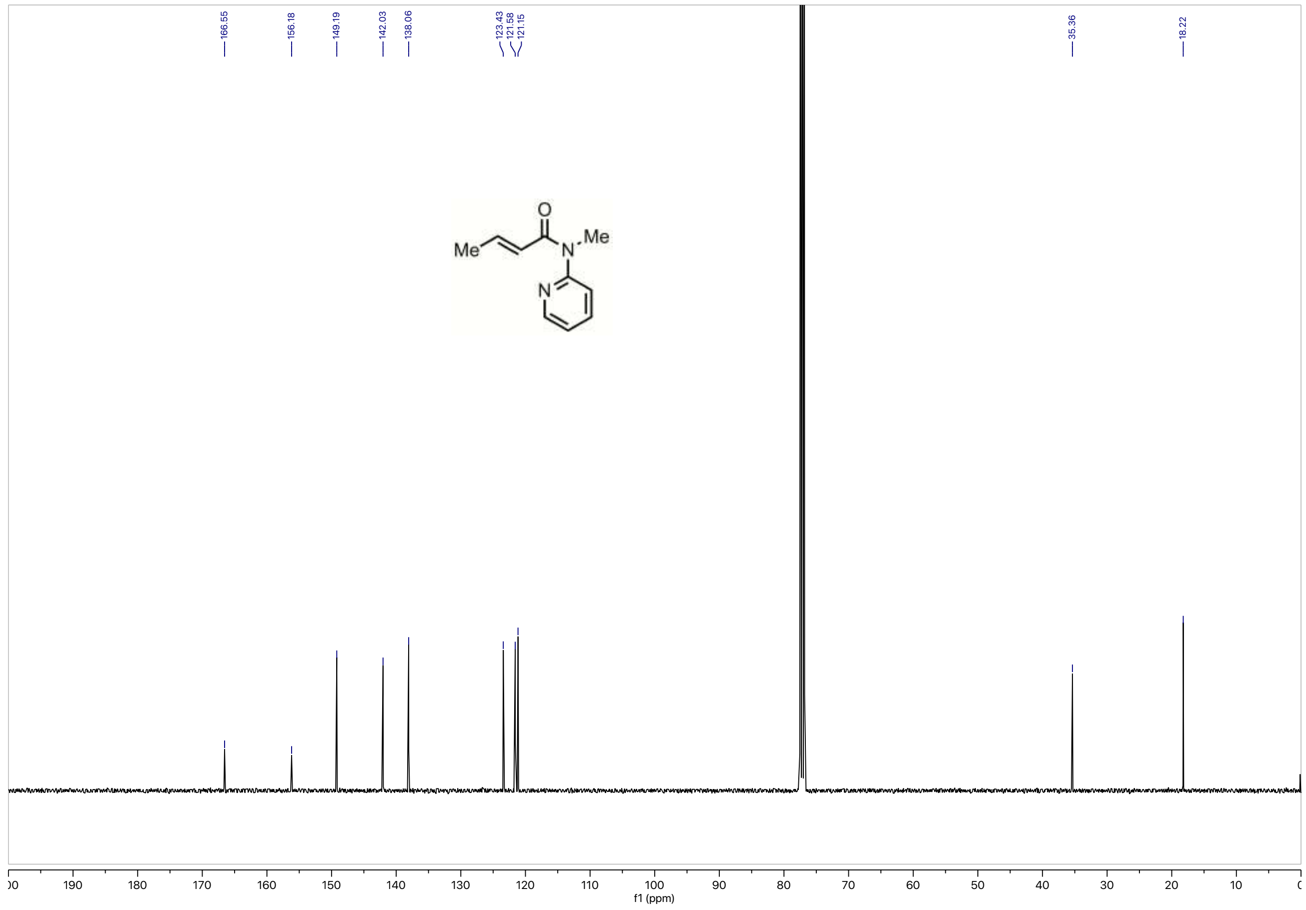

^{1}H NMR: **8**

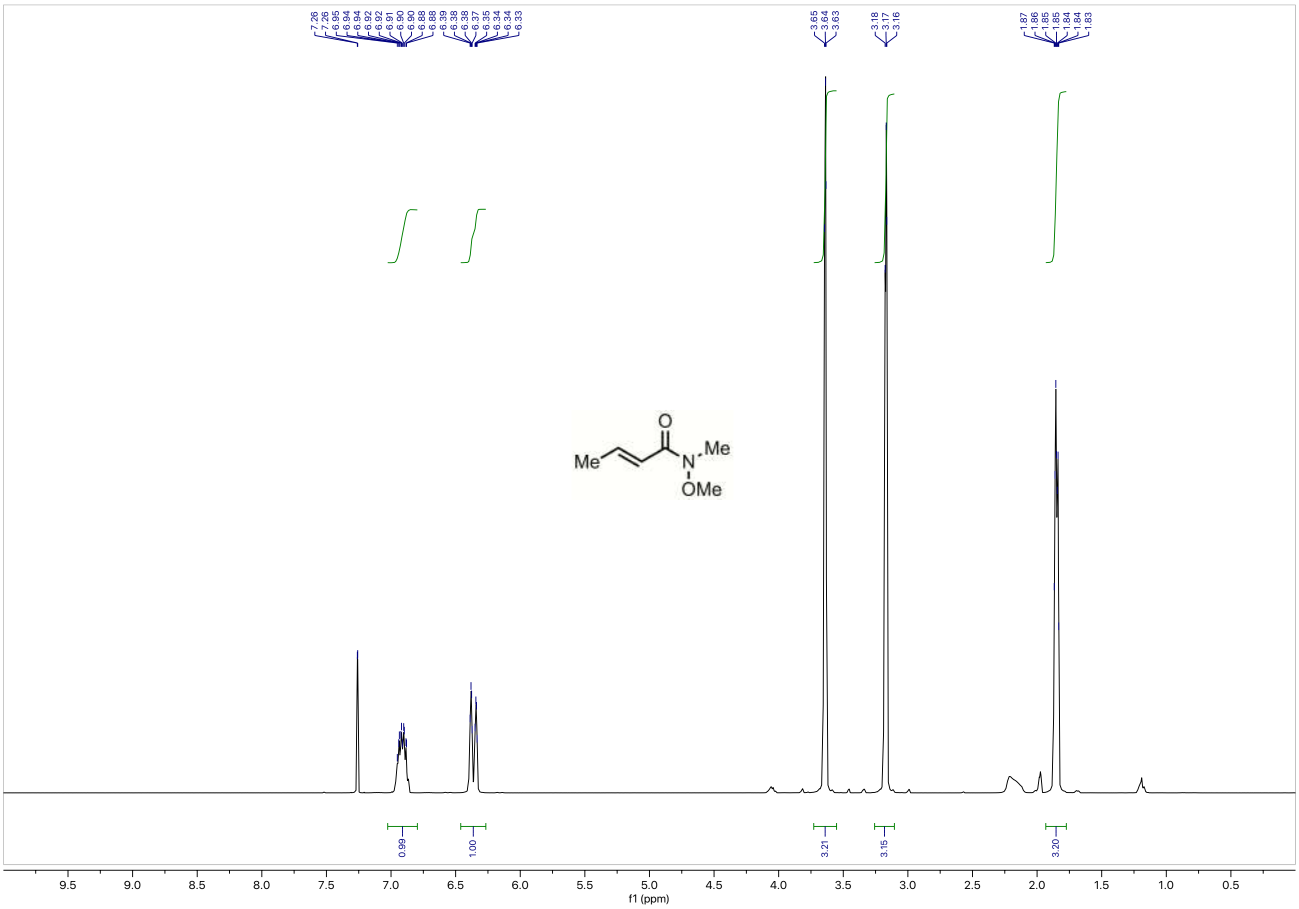

^{13}C NMR: **8**

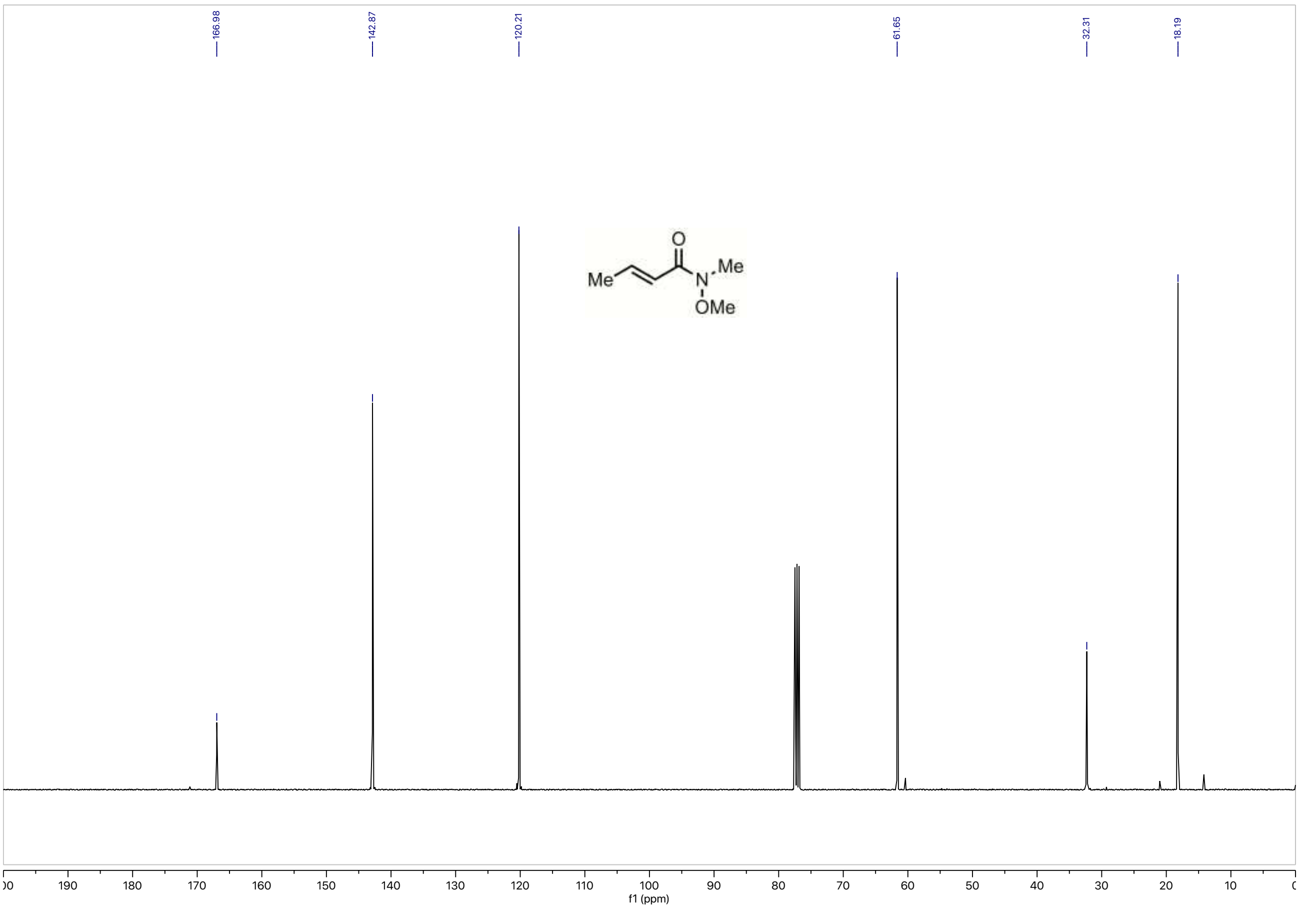

^{1}H NMR: **9**

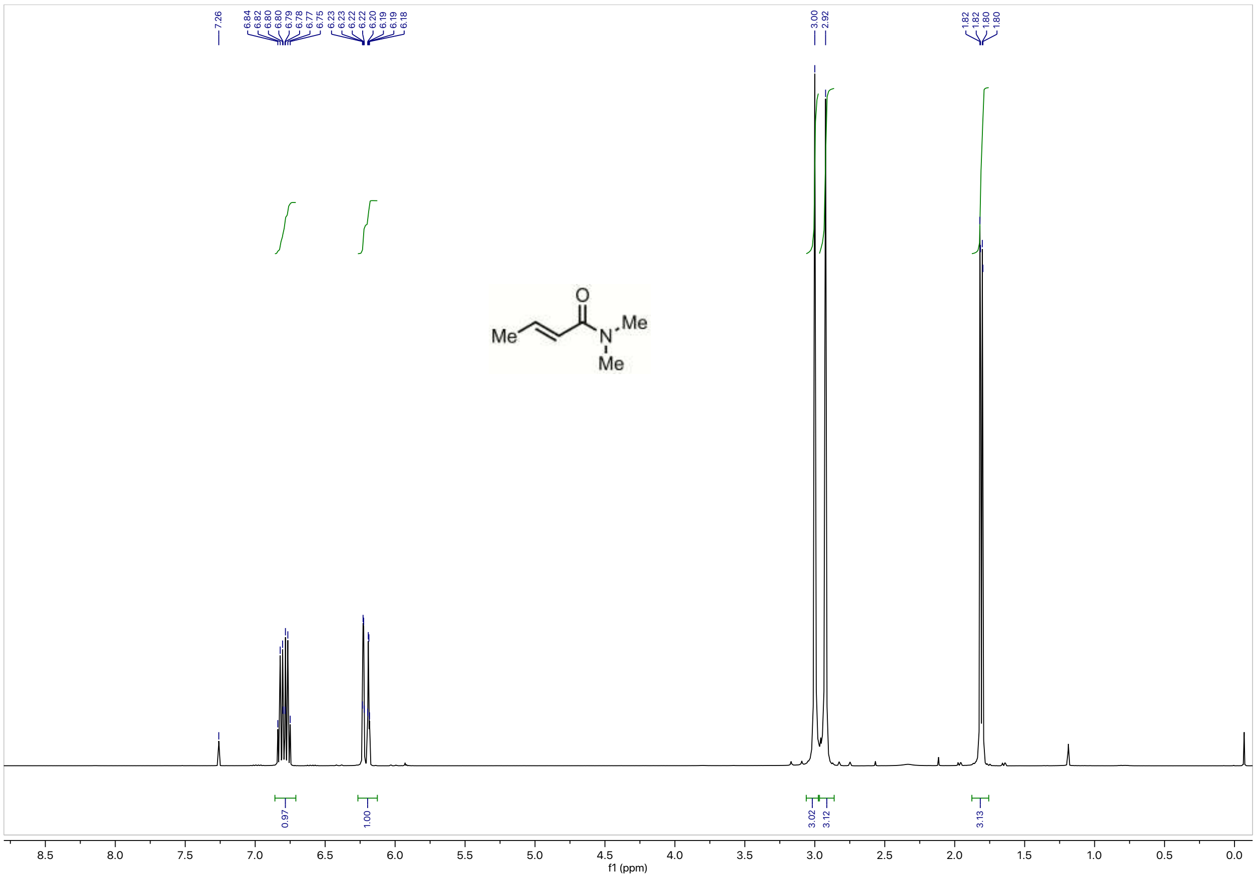

^{13}C NMR: **9**

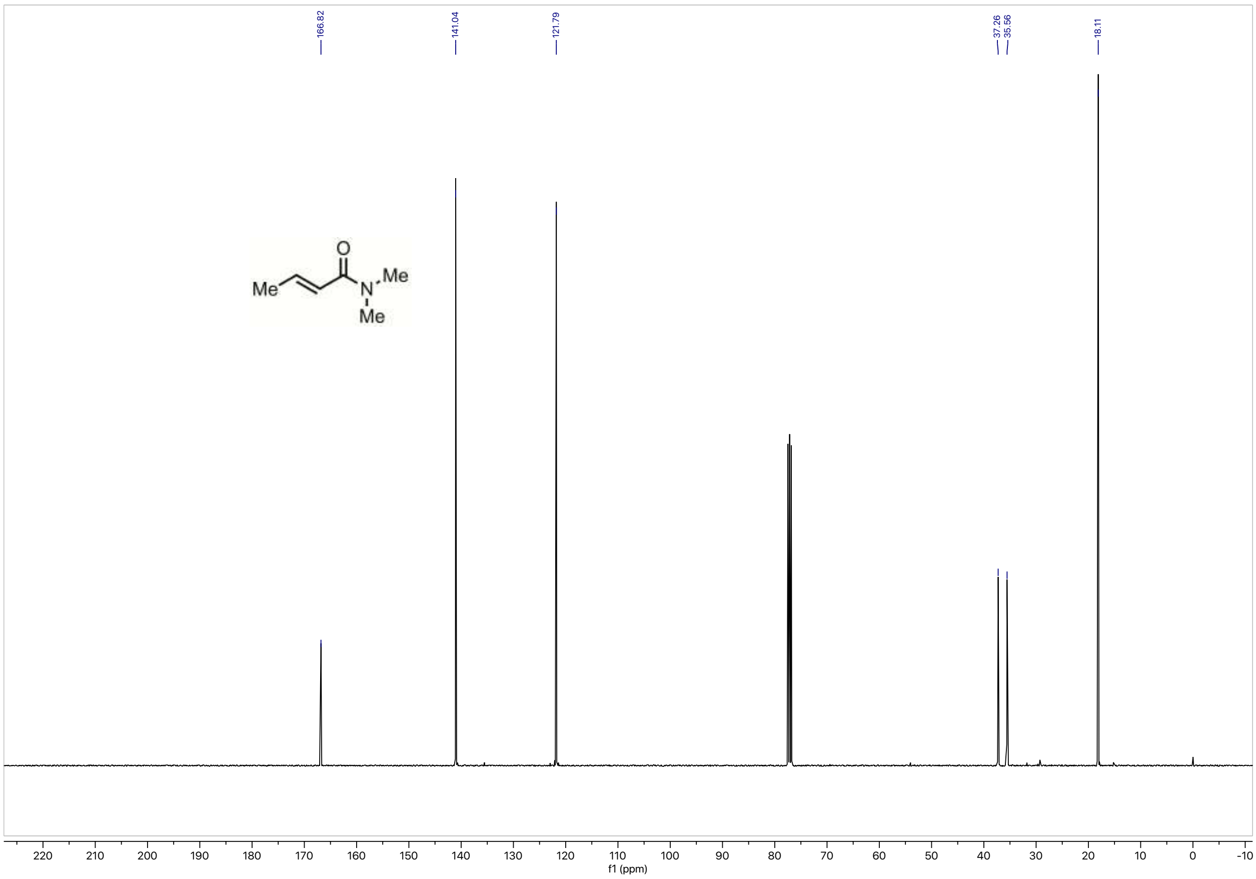

^{1}H NMR: **10**

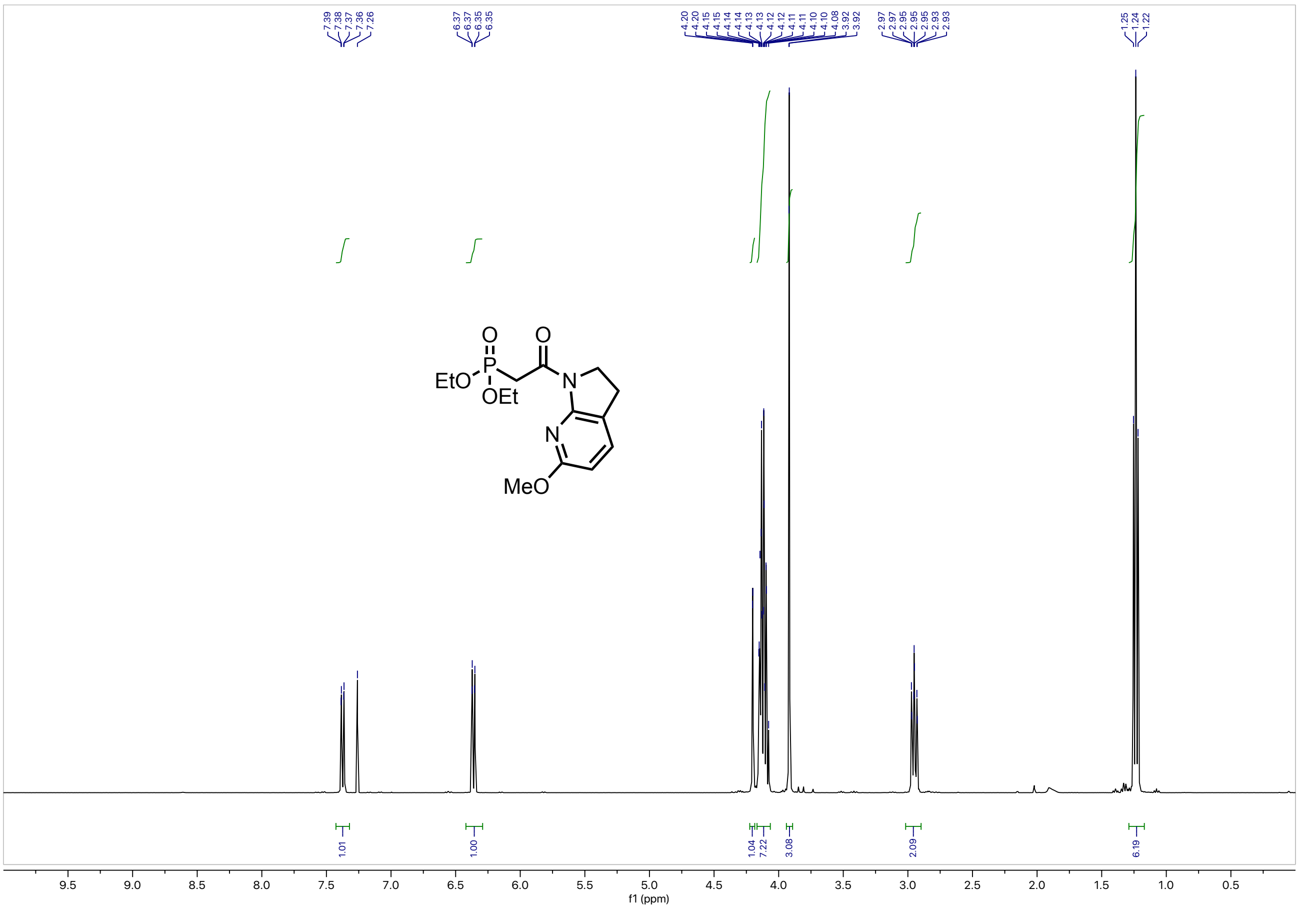

^{13}C NMR: **10**

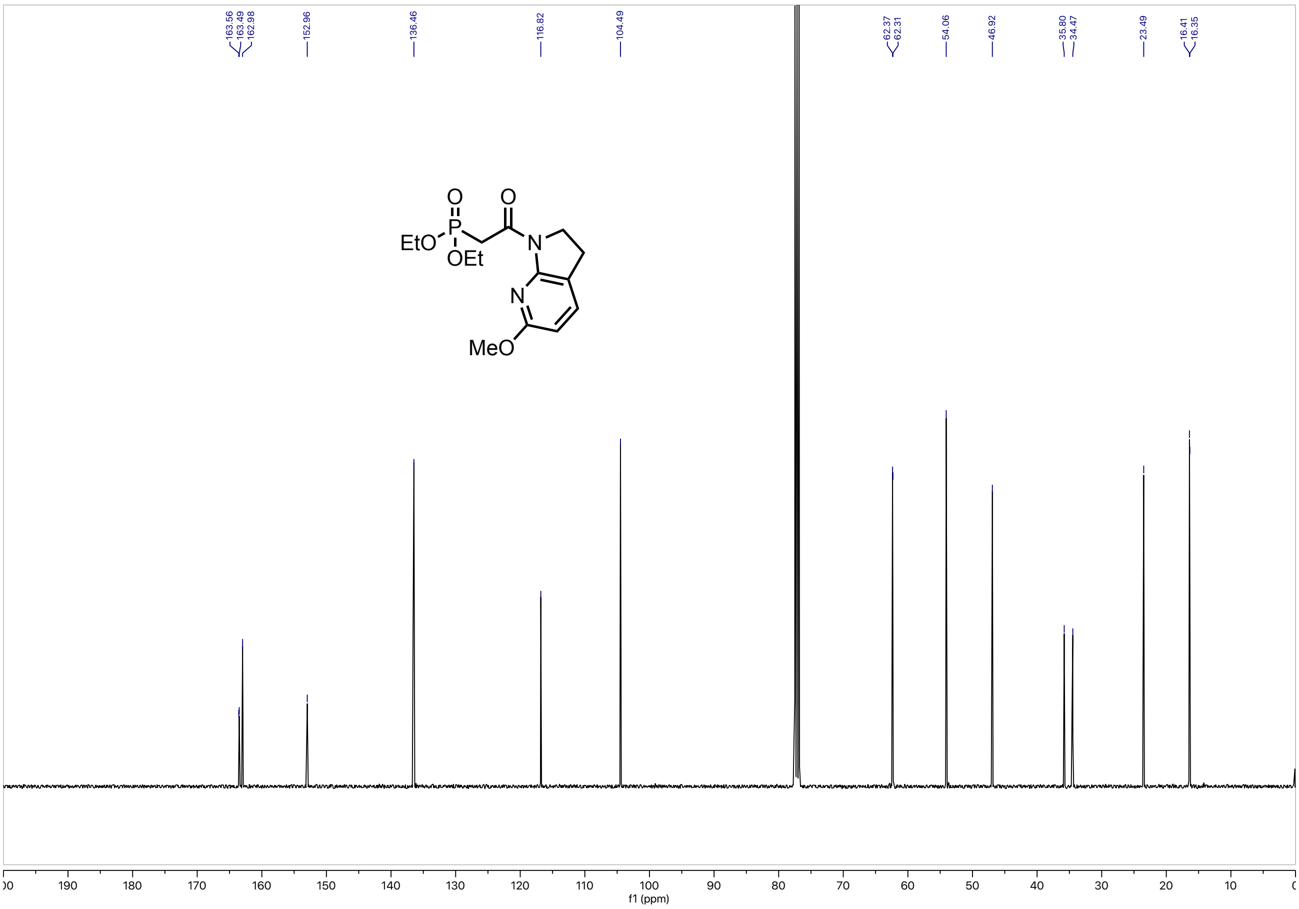

^{31}P NMR: **10**

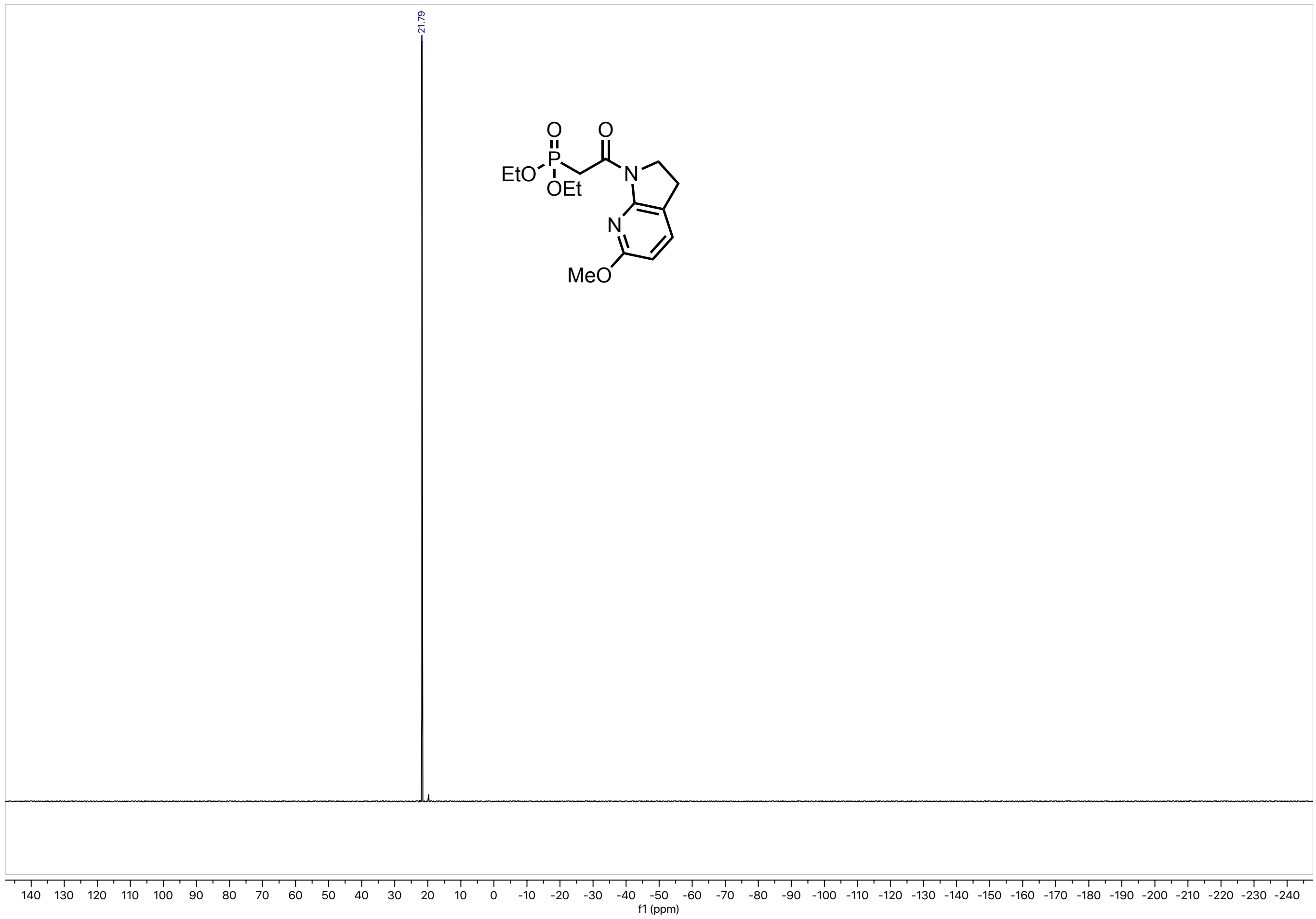
21.79
EtO
OEt
MeO
140
130
120
110
100
90
80
70
60
50
40
30
20
10
0
-10
-20
-30
-40
-50
-60
-70
-80
-90
-100
-110
-120
-130
-140
-150
-160
-170
-180
-190
-200
-210
-220
-230
-240
f1 (ppm)

^{1}H NMR: **2a**

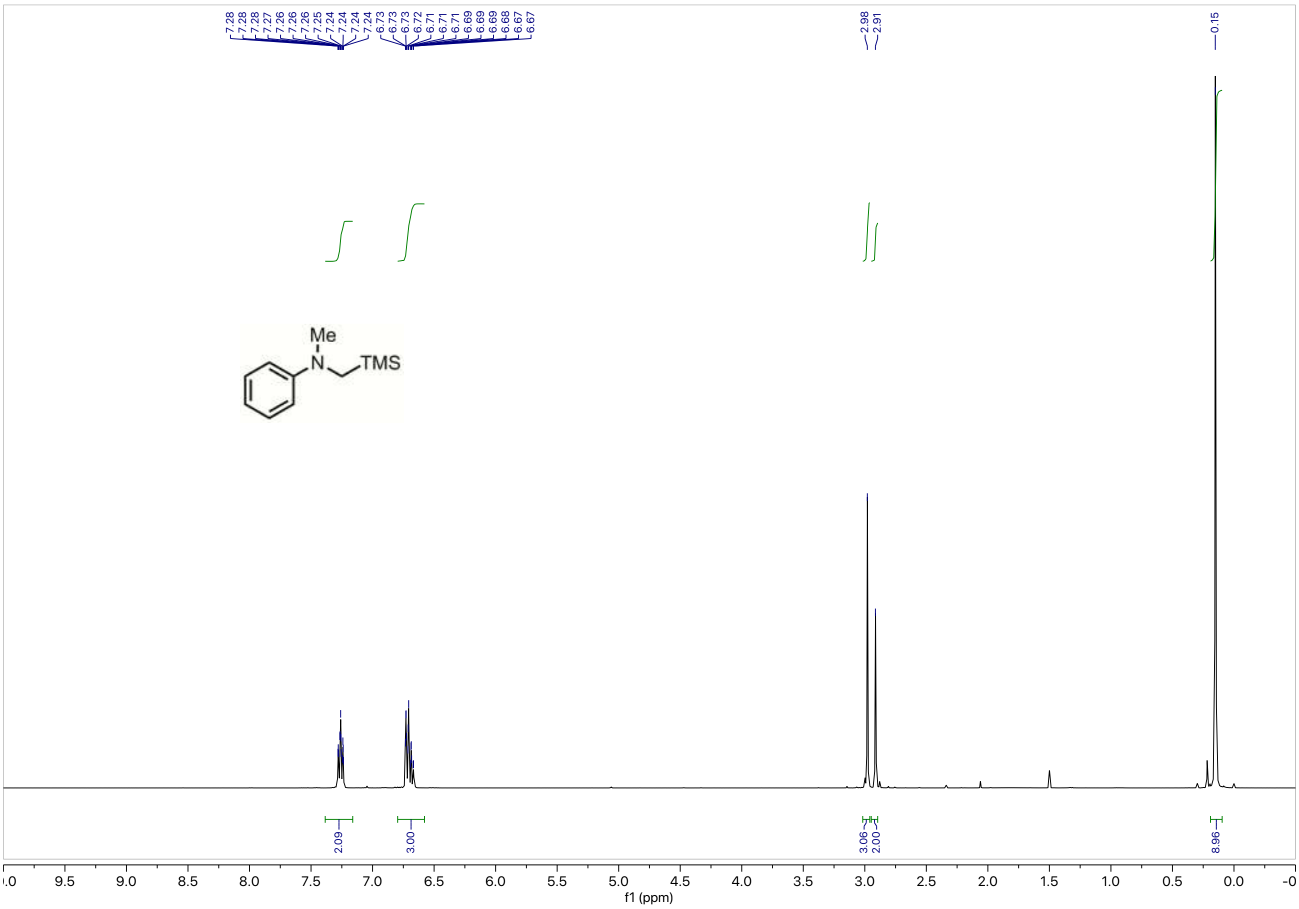

^{13}C NMR: **2a**

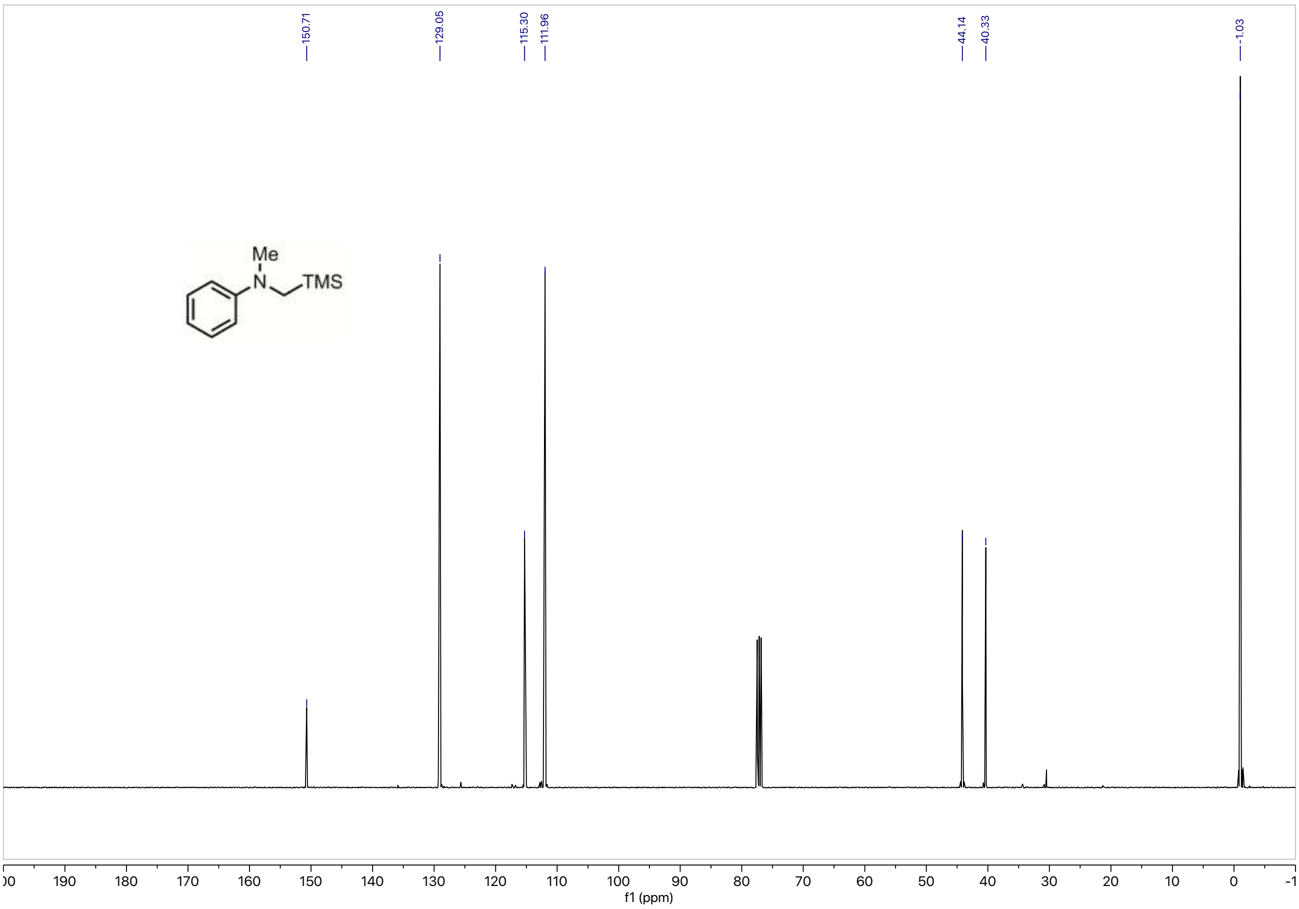

^{1}H NMR: **2b**

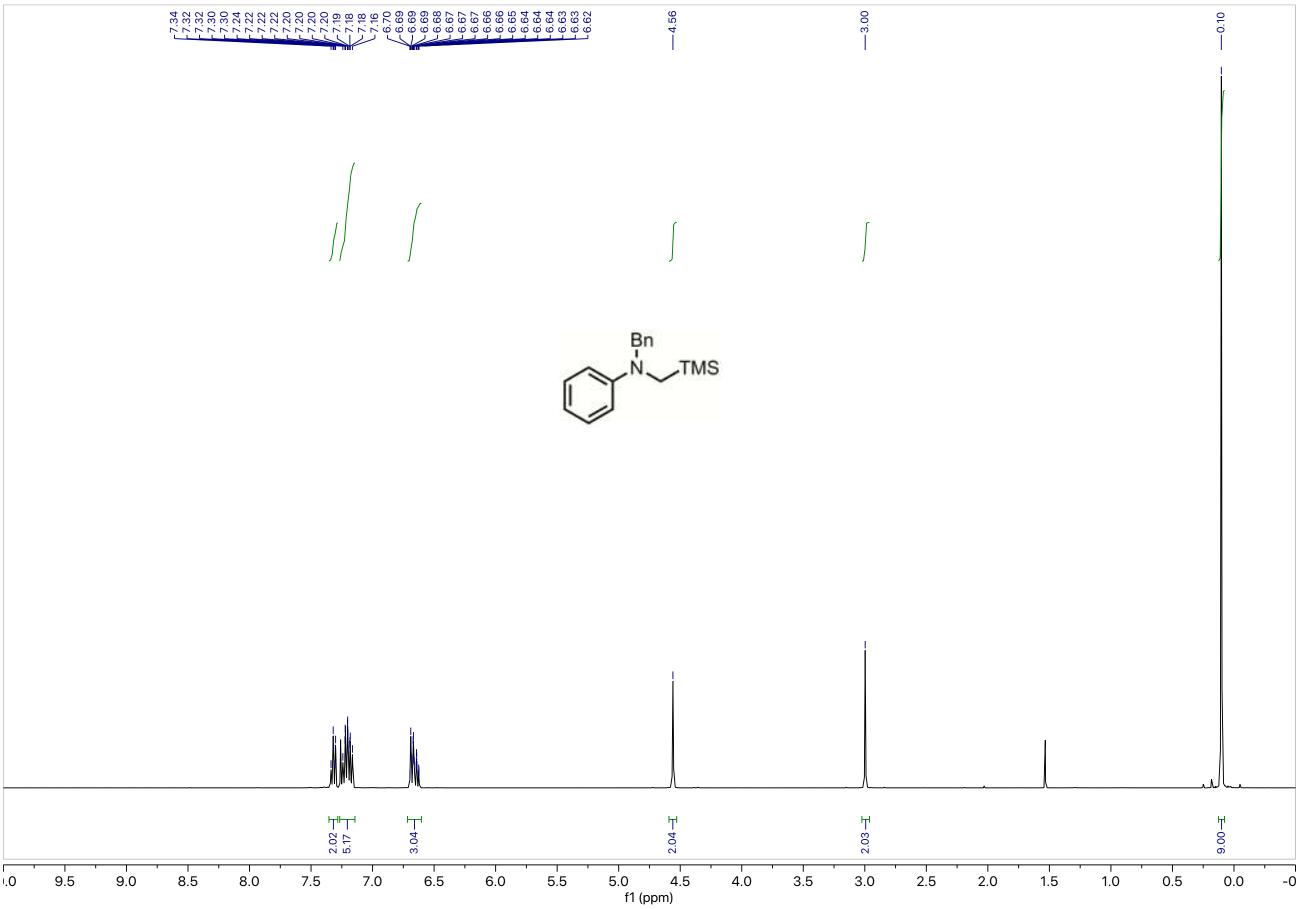

^{13}C NMR: **2b**

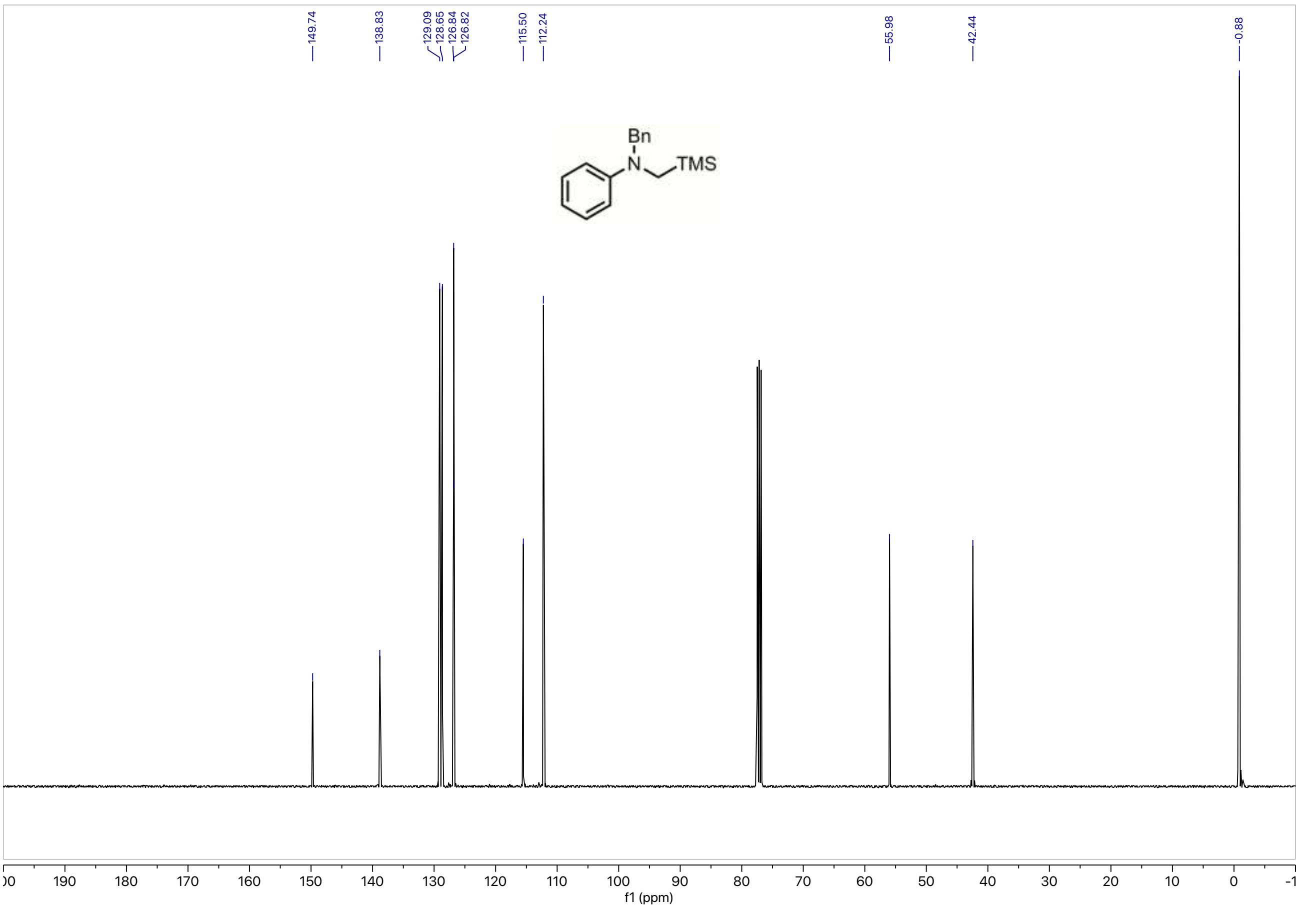

^{1}H NMR: **2c**

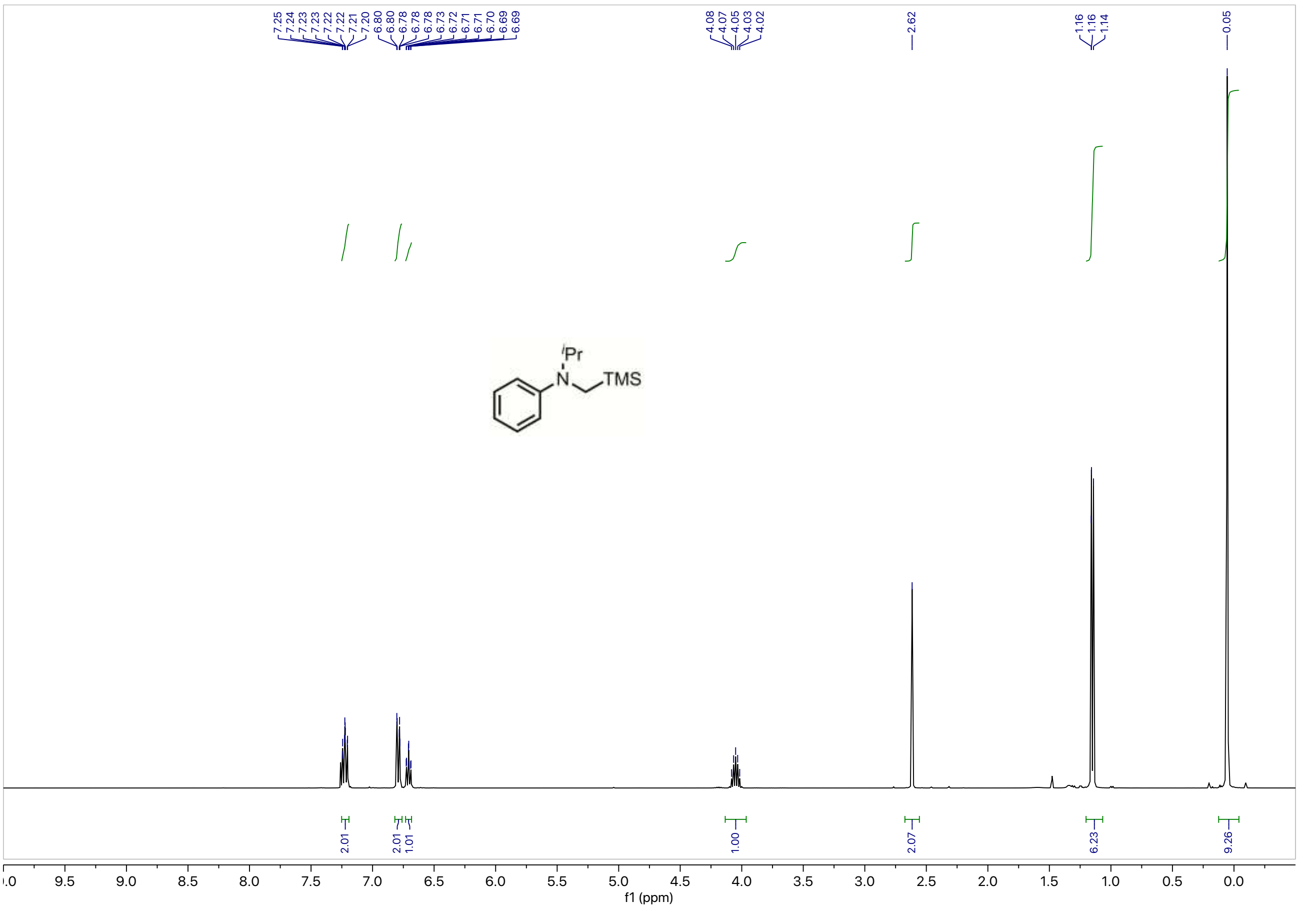

^{13}C NMR: **2c**

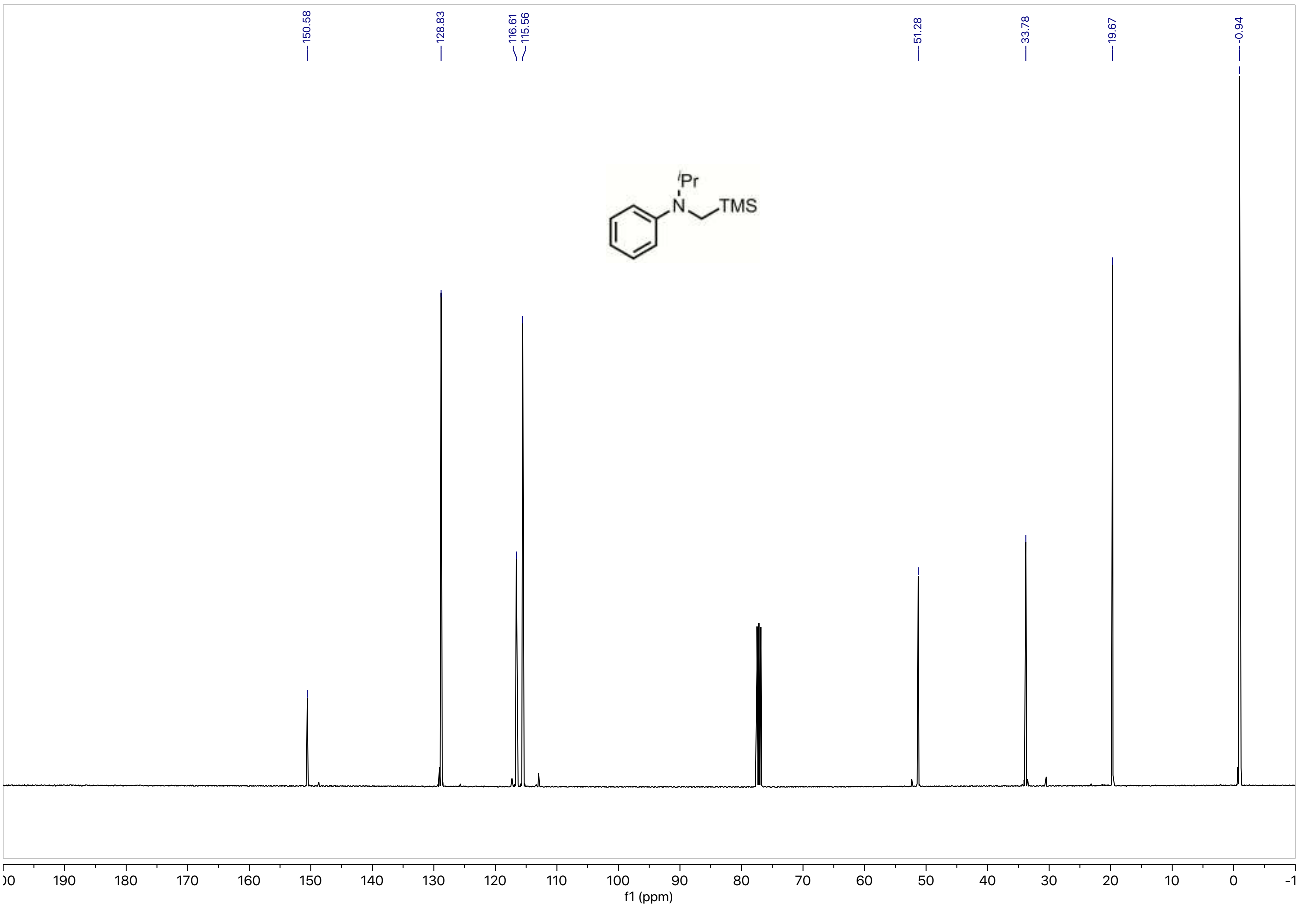

^{1}H NMR: **2d**

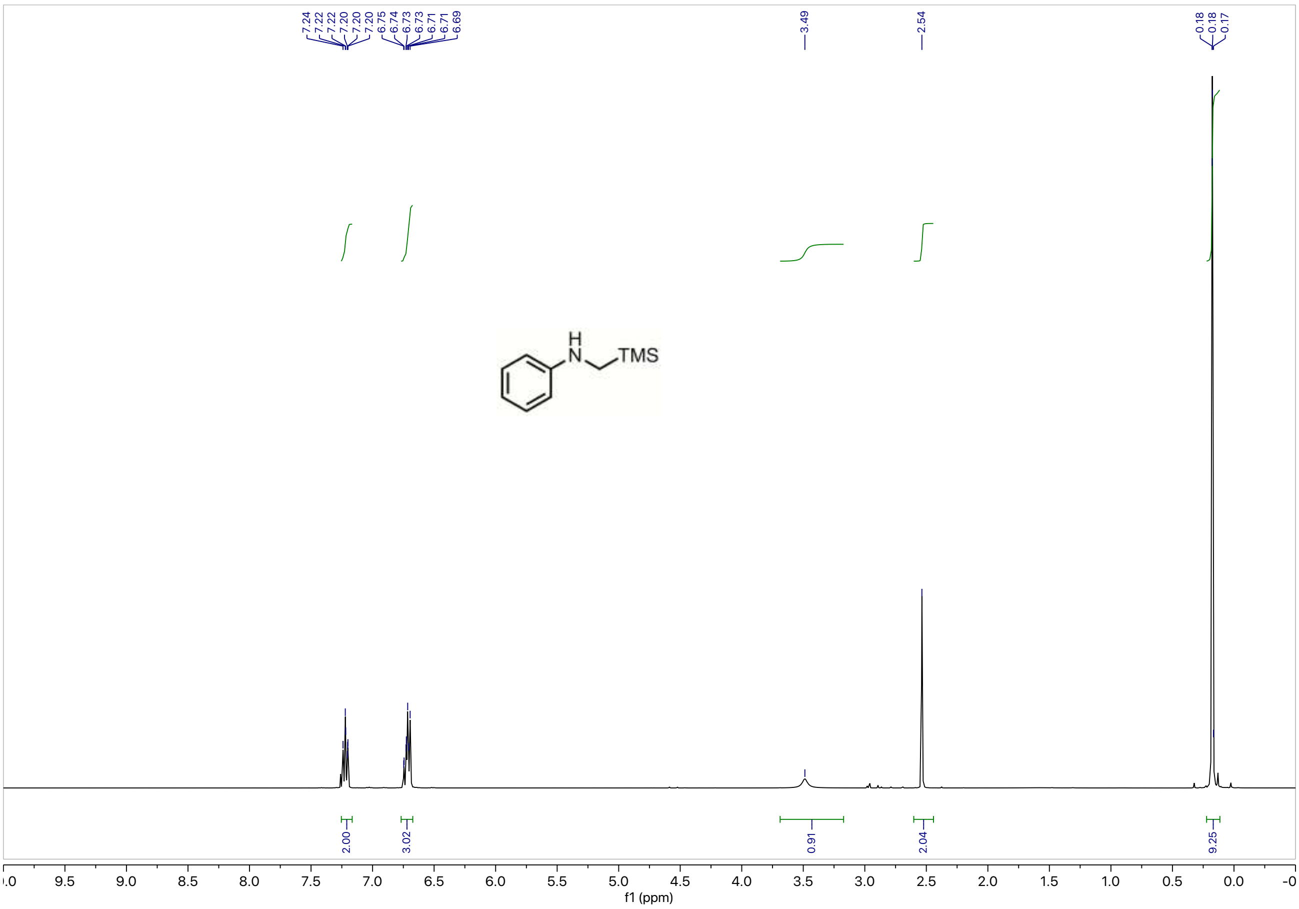

^{13}C NMR: **2d**

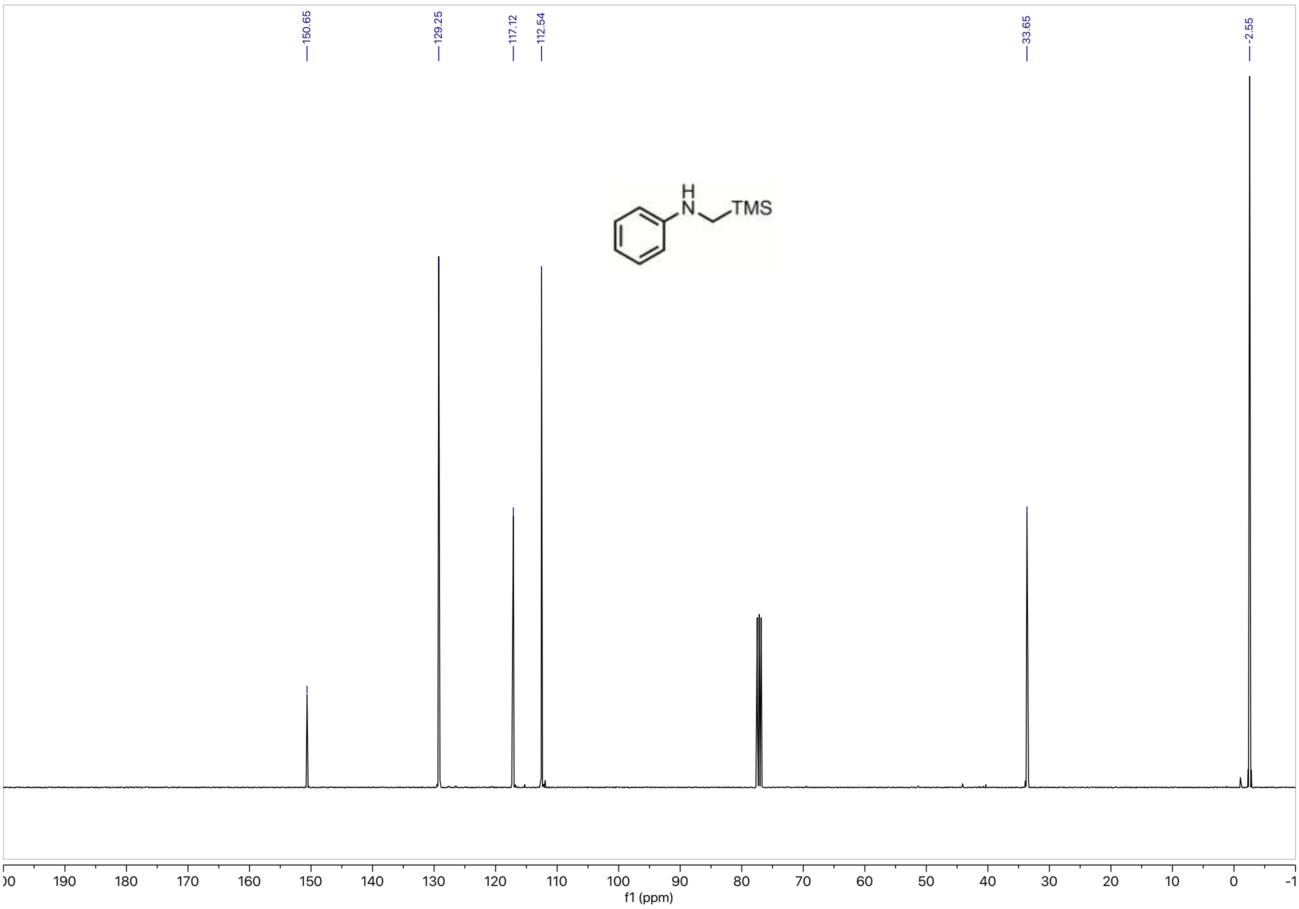

^{1}H NMR: **2e**

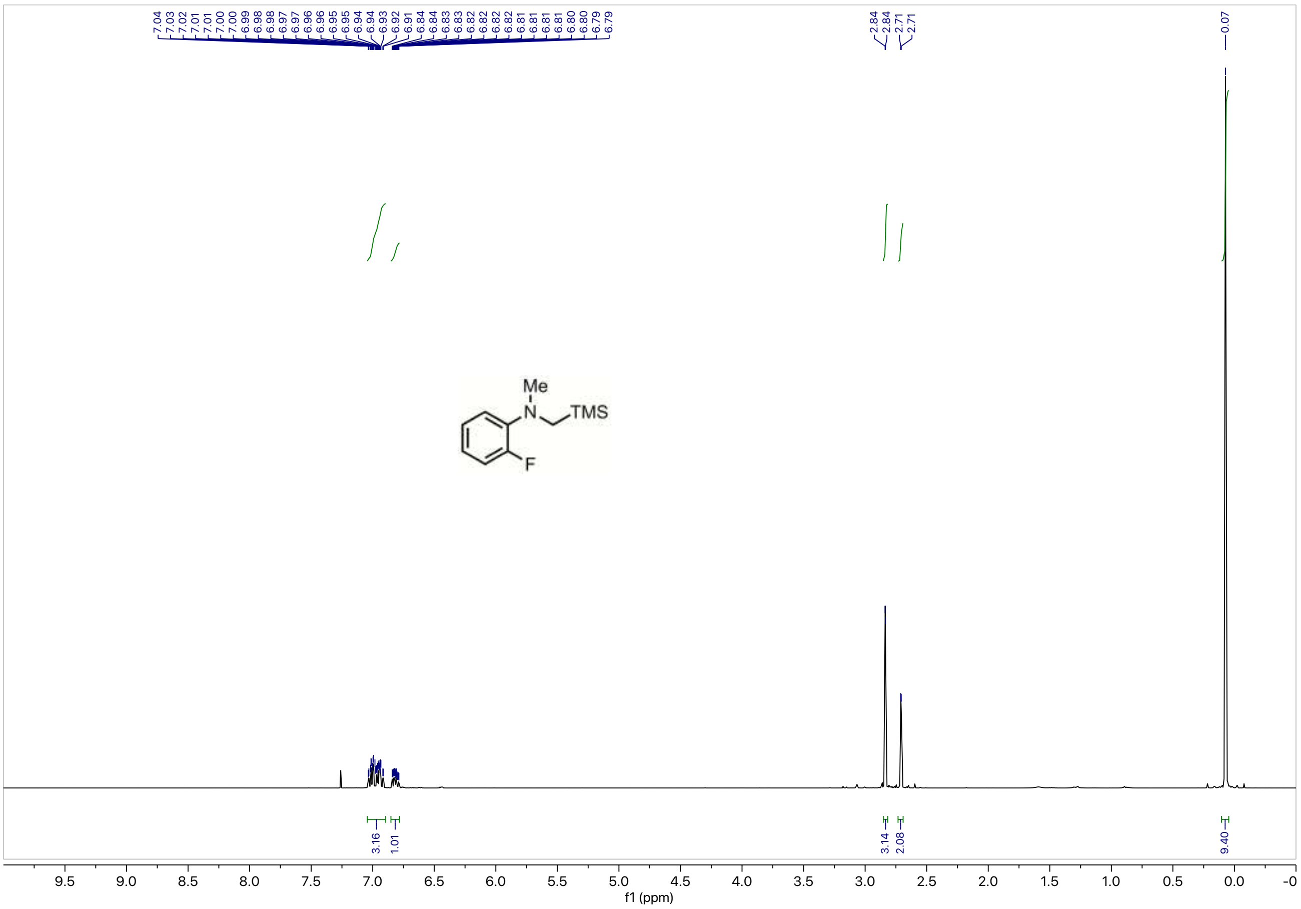

^{13}C NMR: **2e**

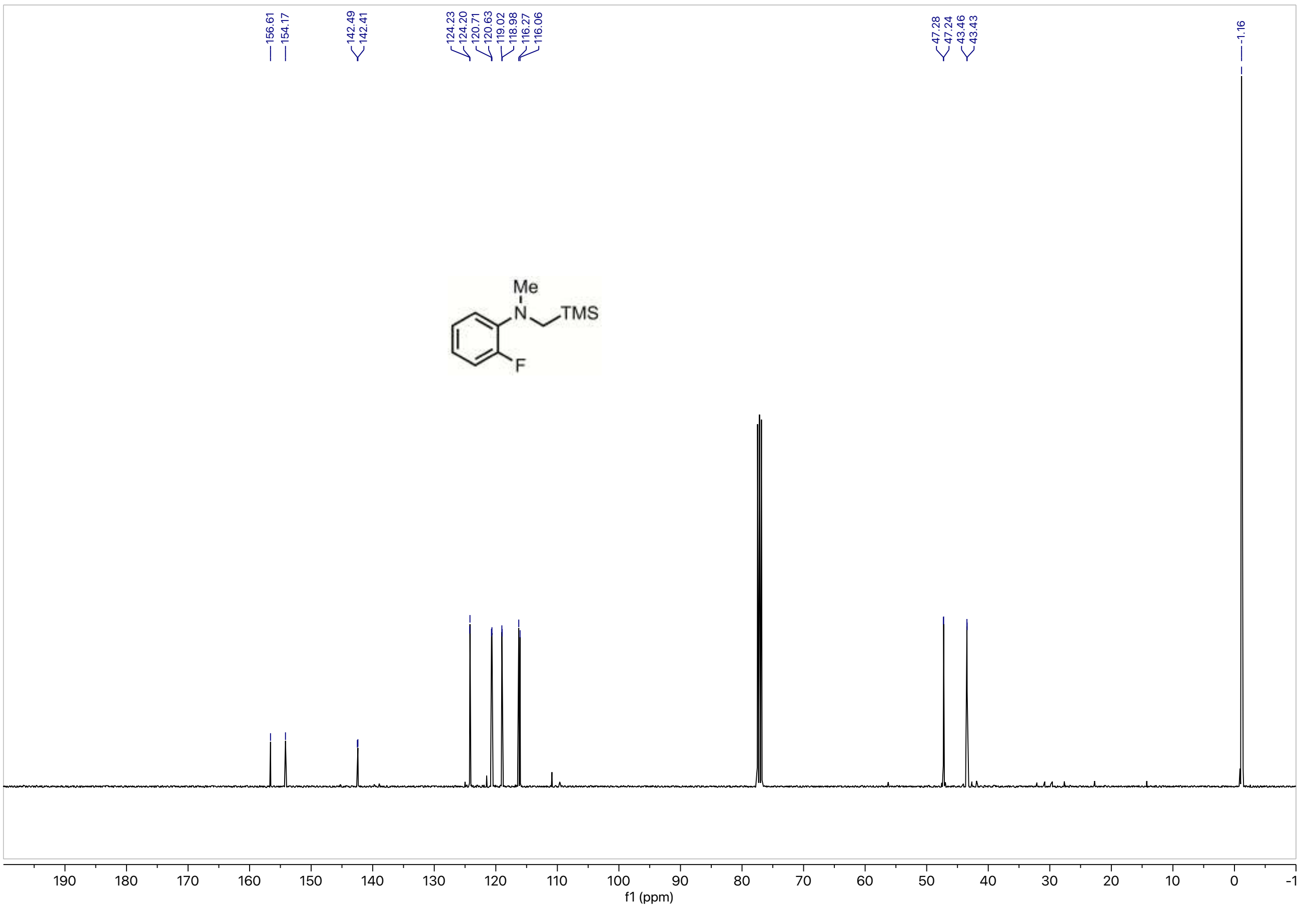

^{19}F NMR: **2e**

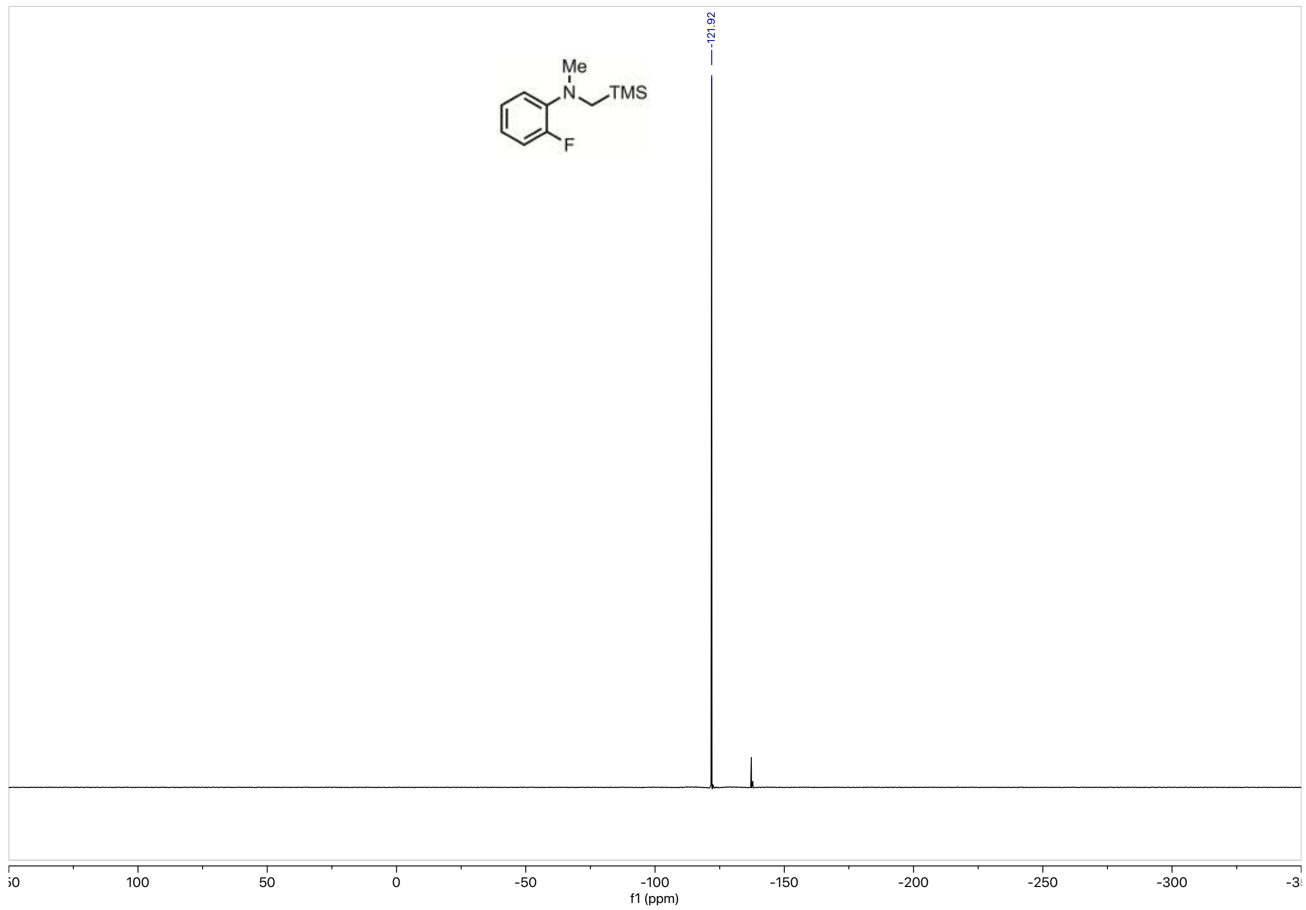

^{1}H NMR: **3aa**

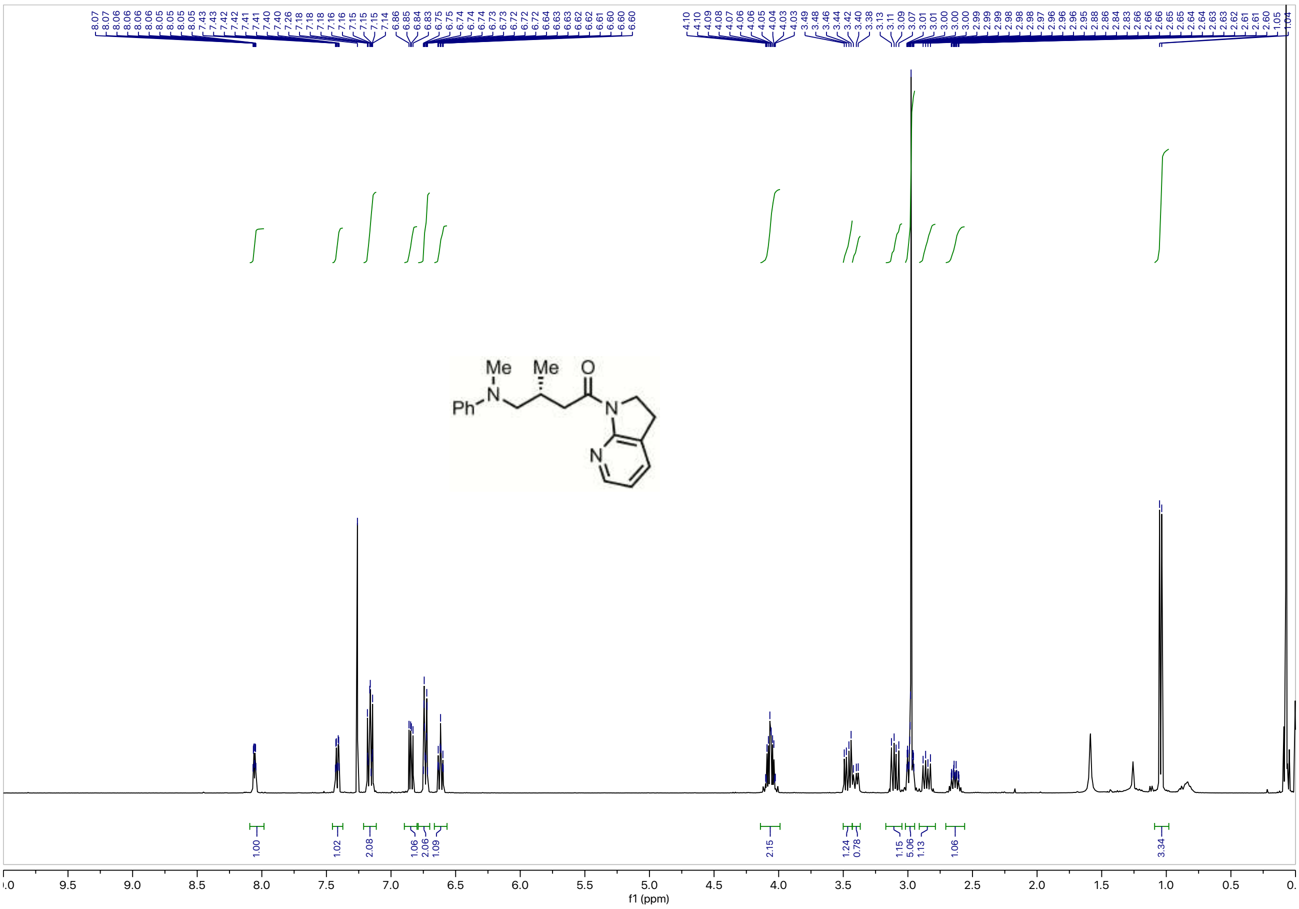

^{13}C NMR: **3aa**

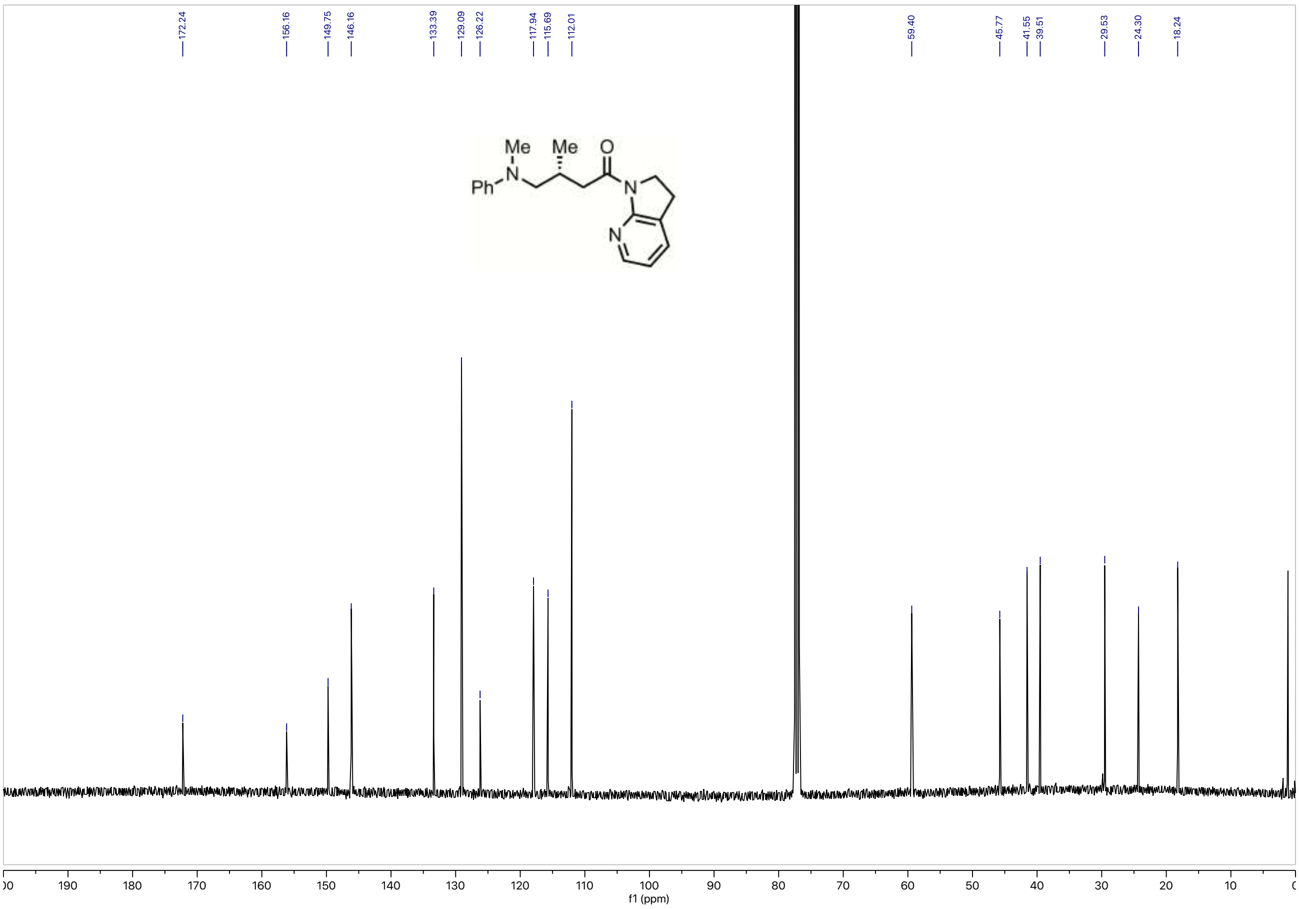

^{1}H NMR: **3ba**

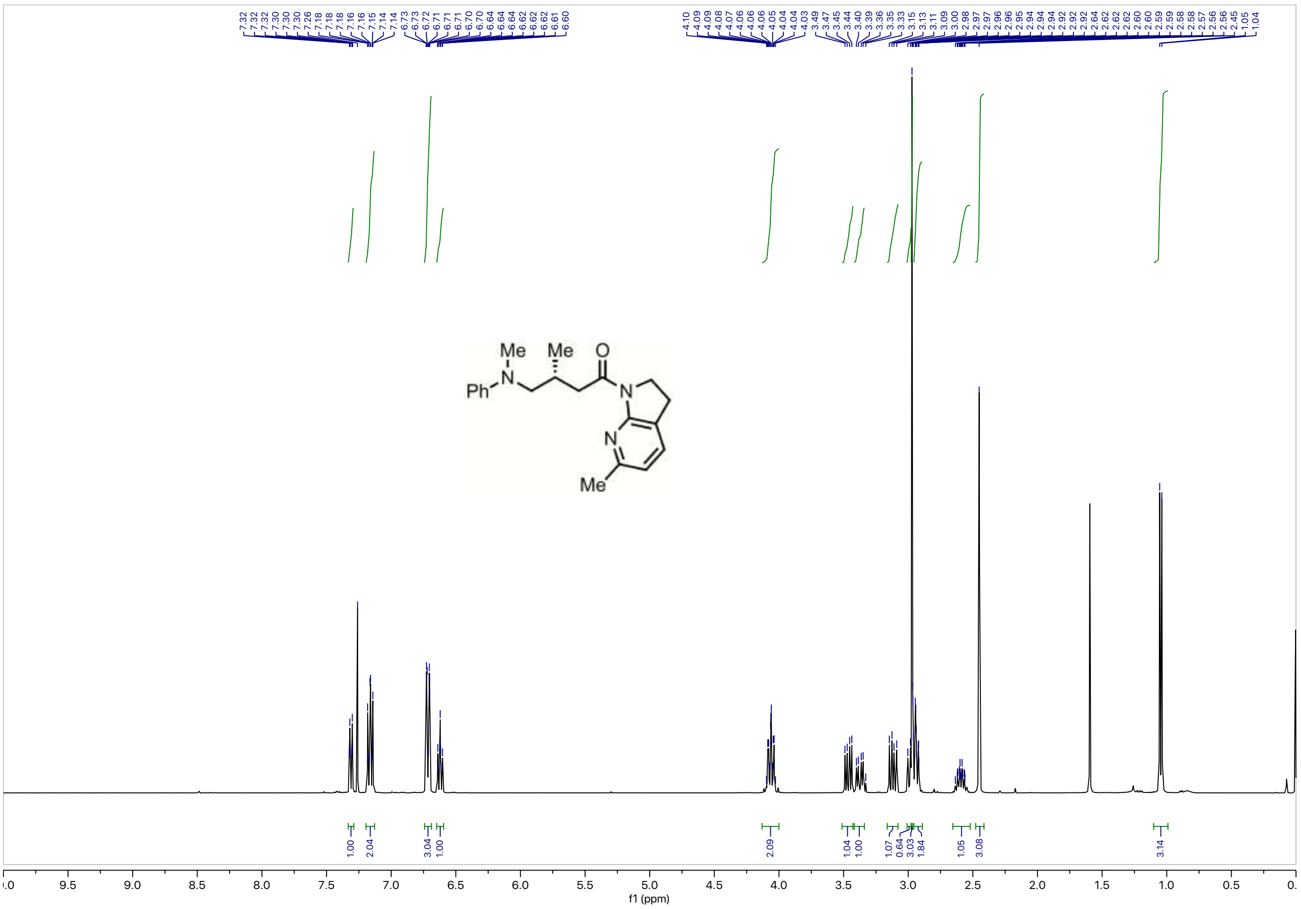

^{13}C NMR: **3ba**

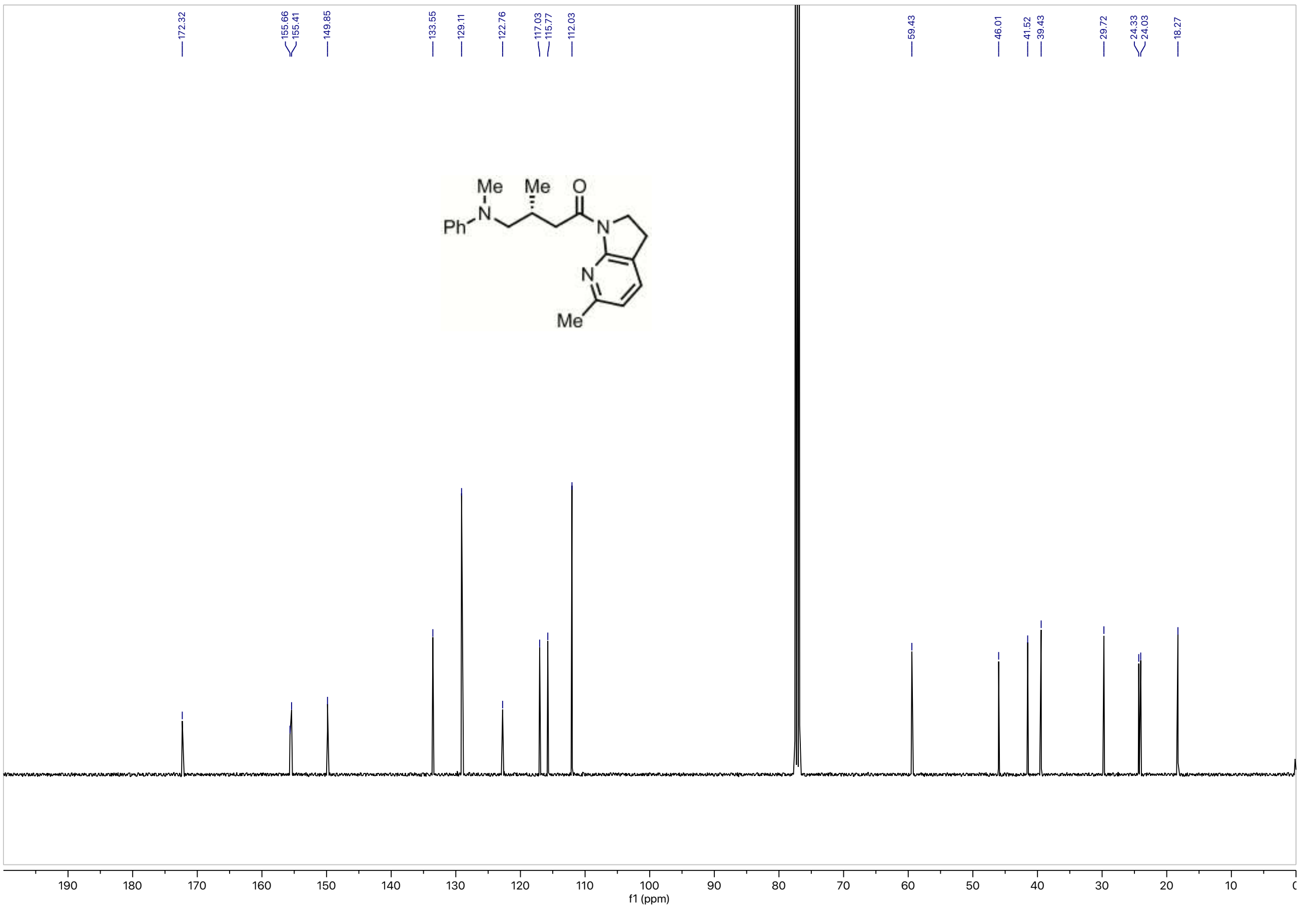

^{1}H NMR: **3ca**

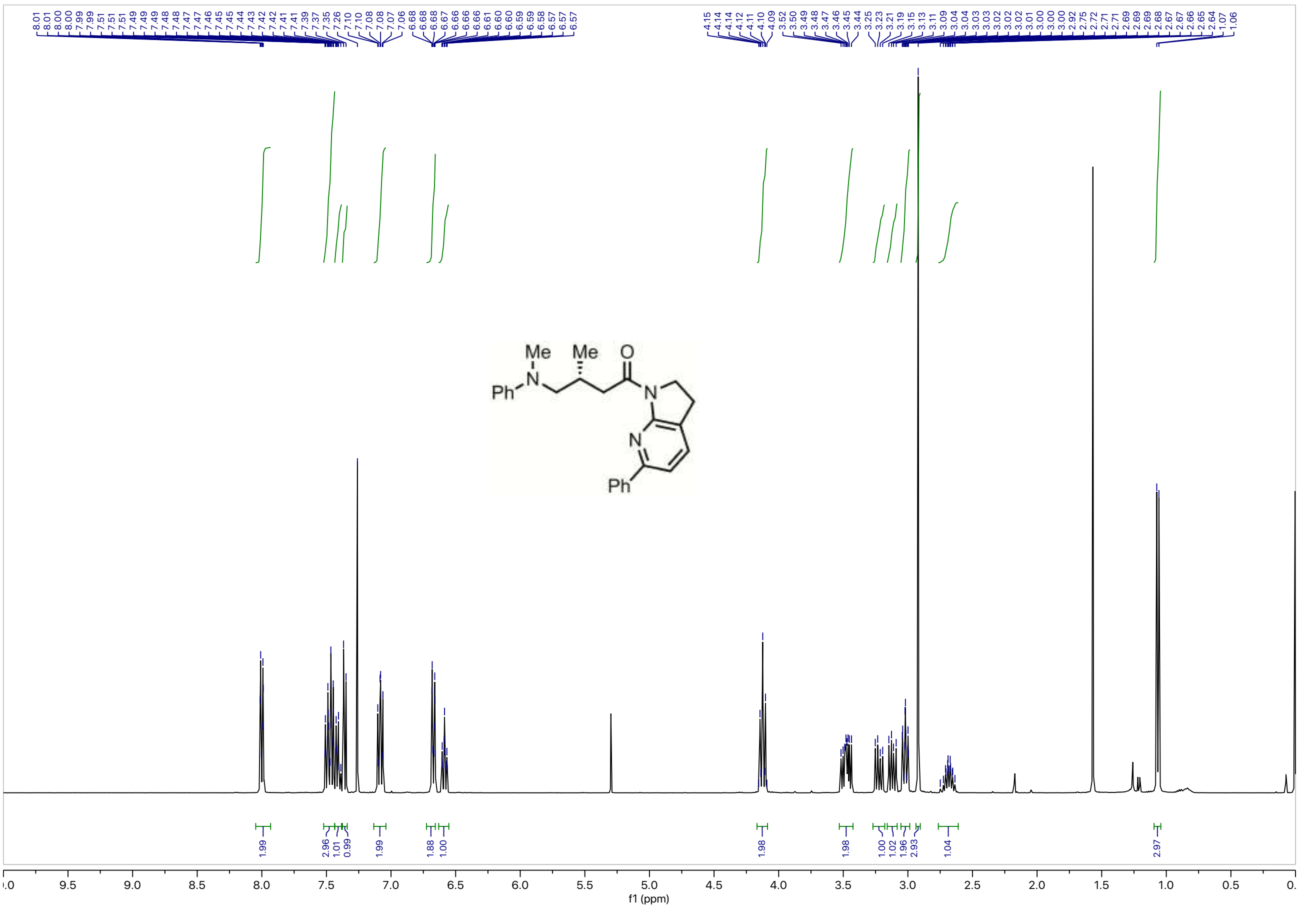

^{13}C NMR: **3ca**

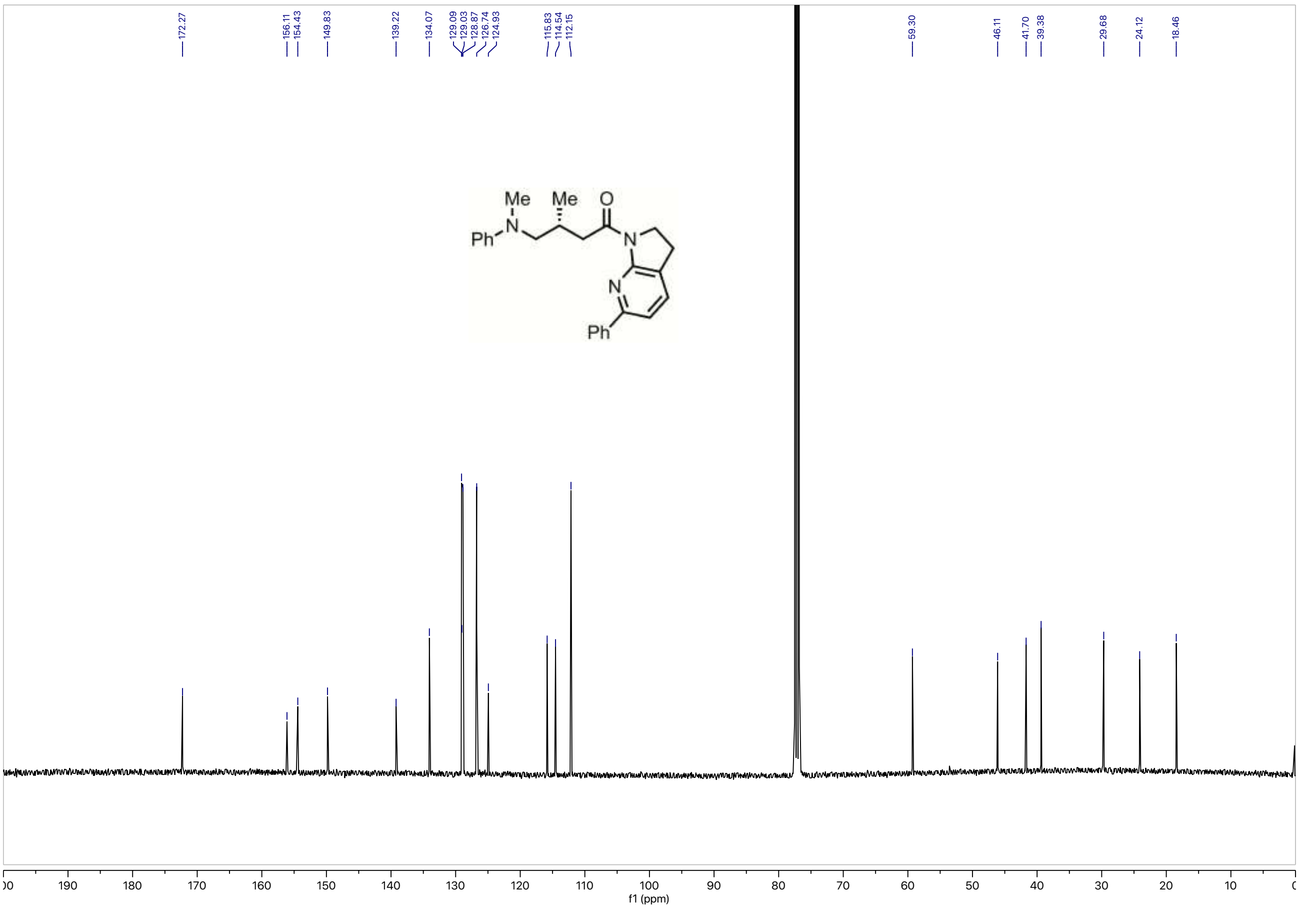

^{1}H NMR: **3da**

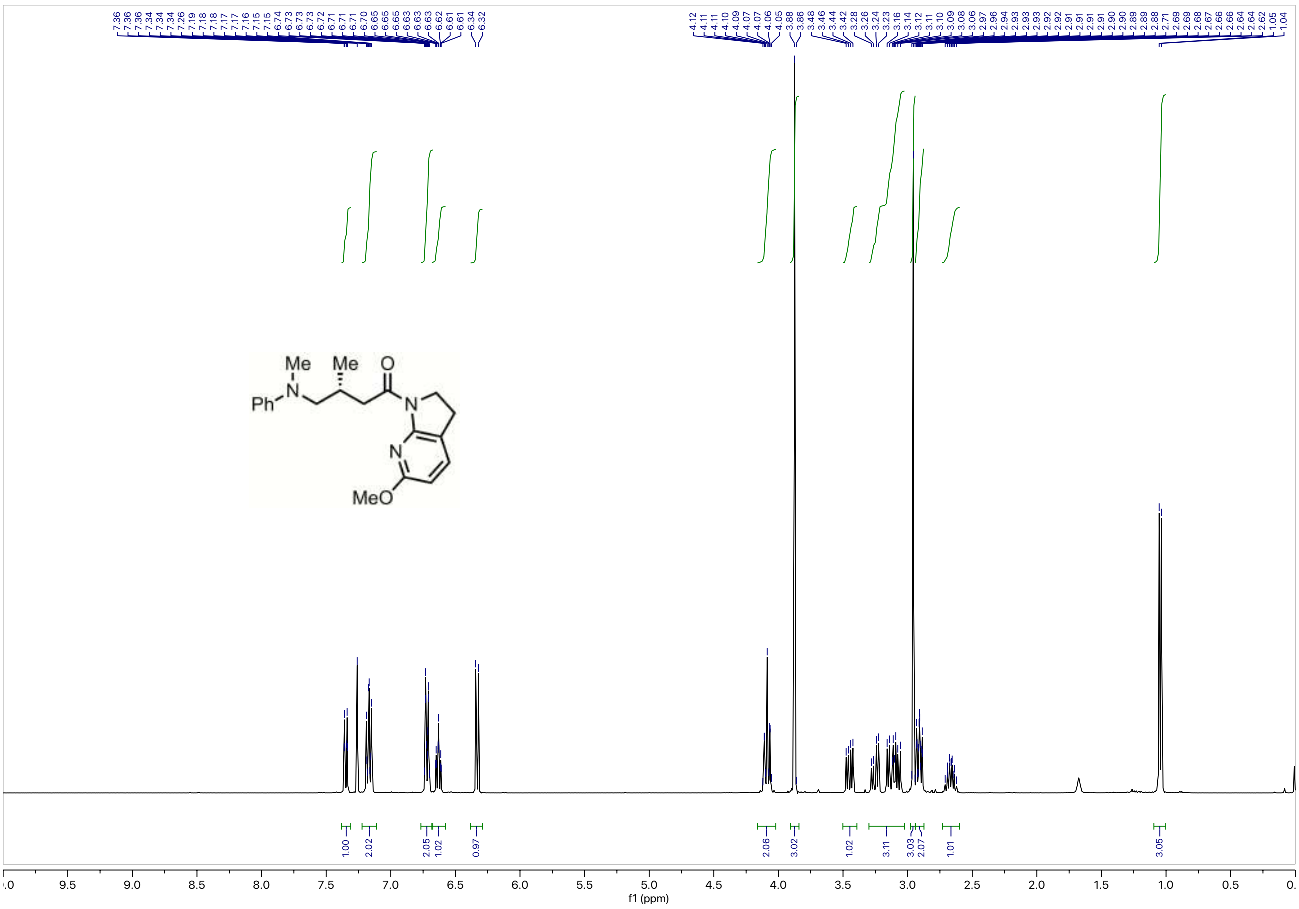

^{13}C NMR: **3da**

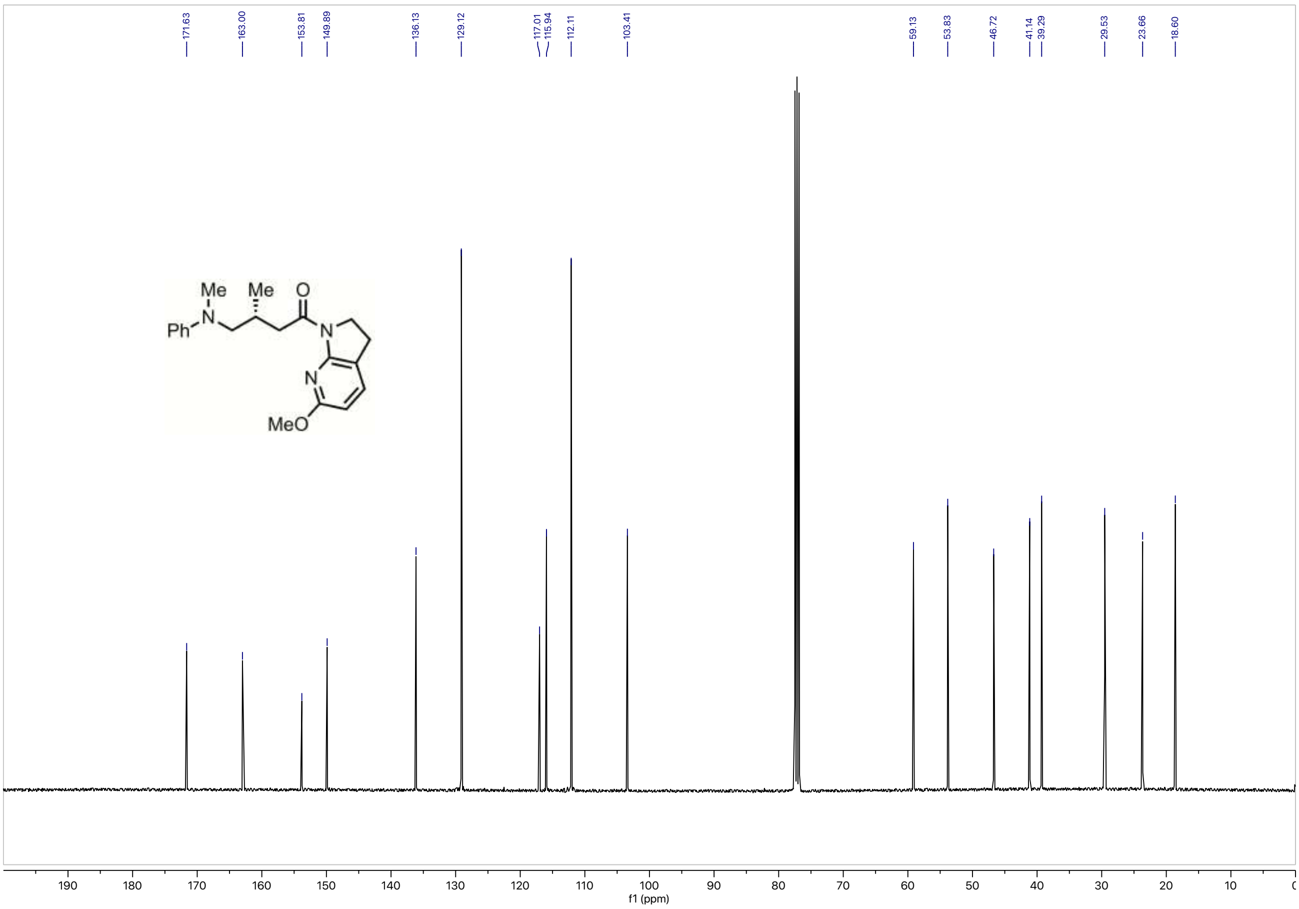

^{1}H NMR: **3ea**

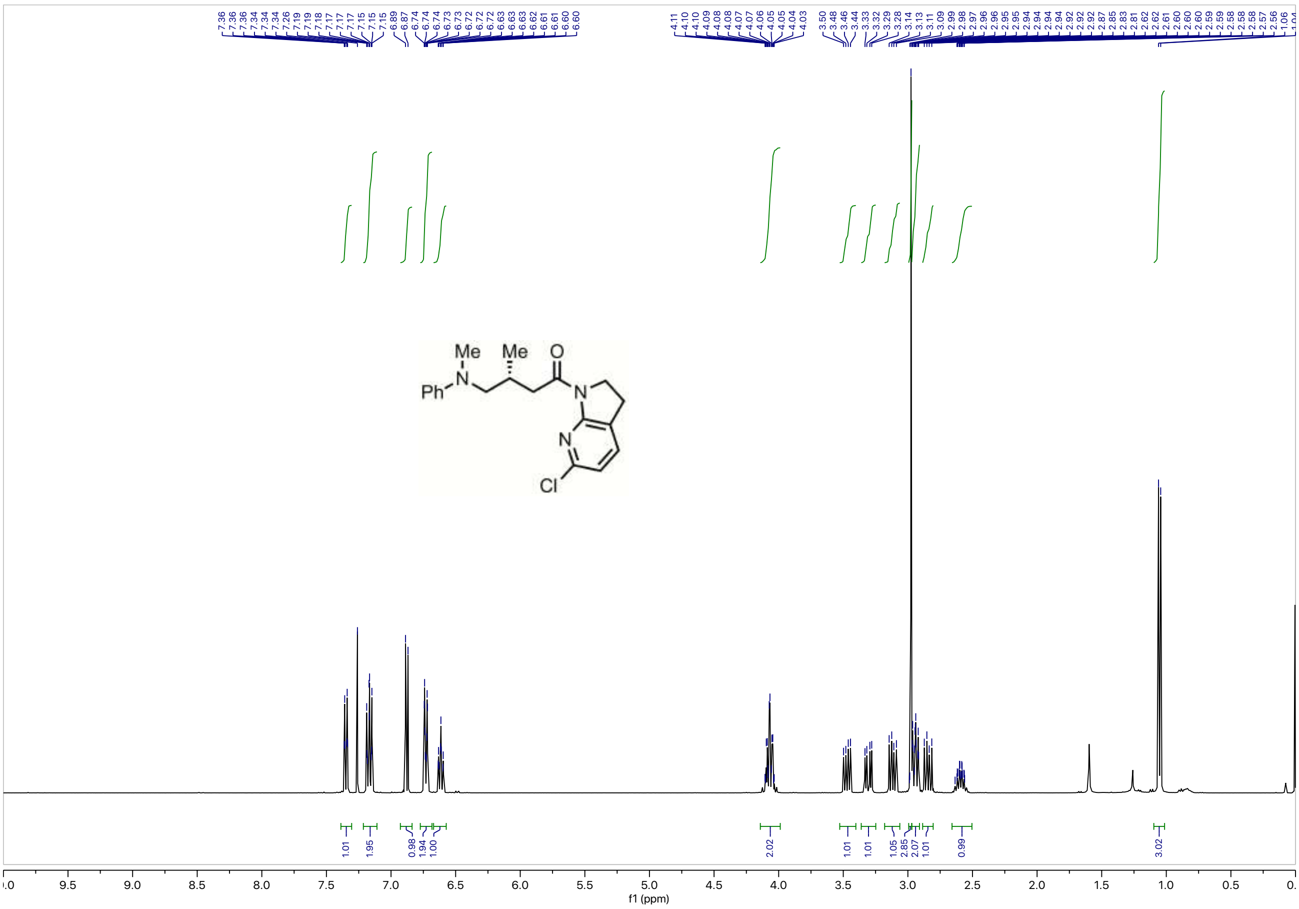

^{13}C NMR: **3ea**

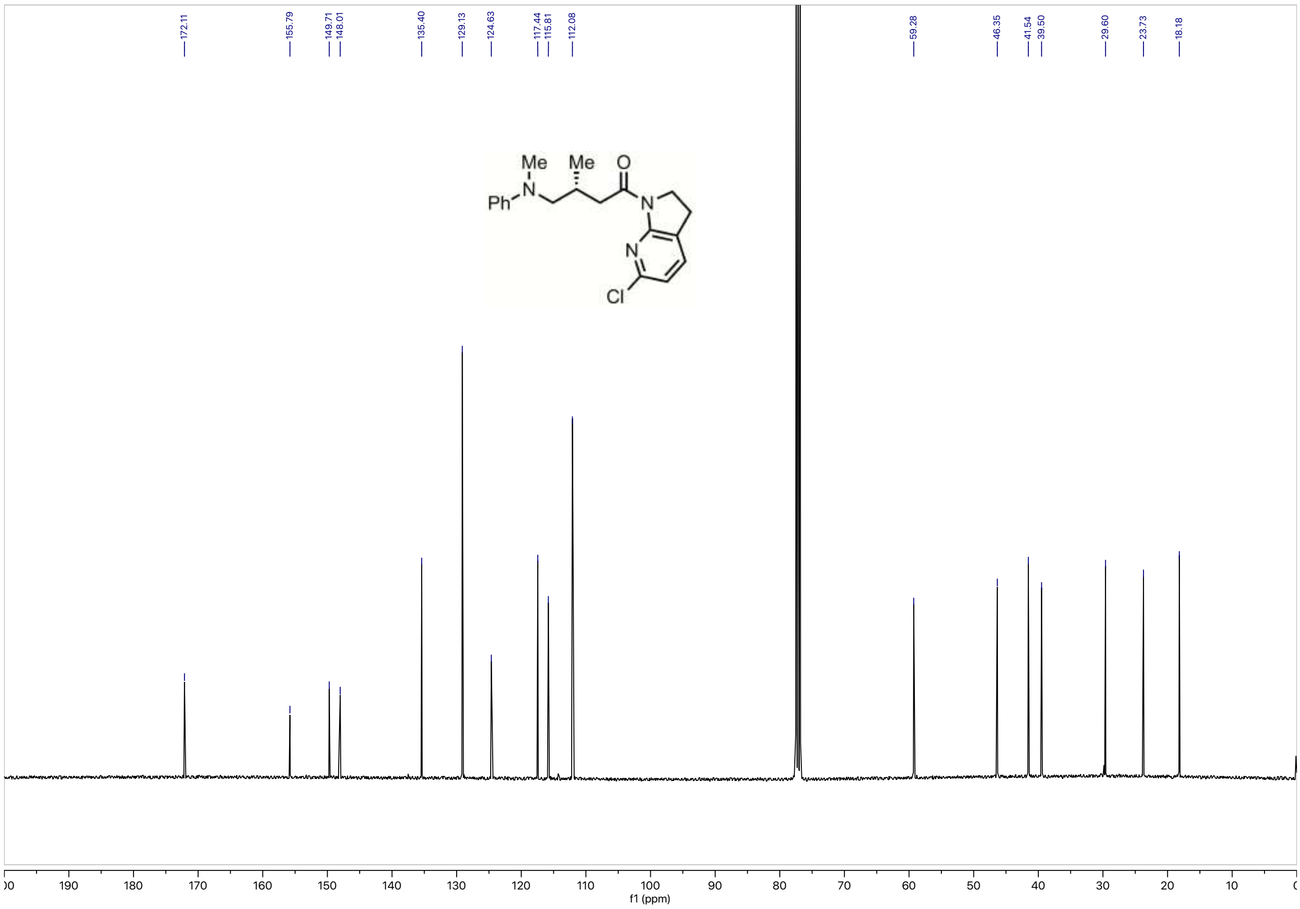

^{1}H NMR: **3fa**

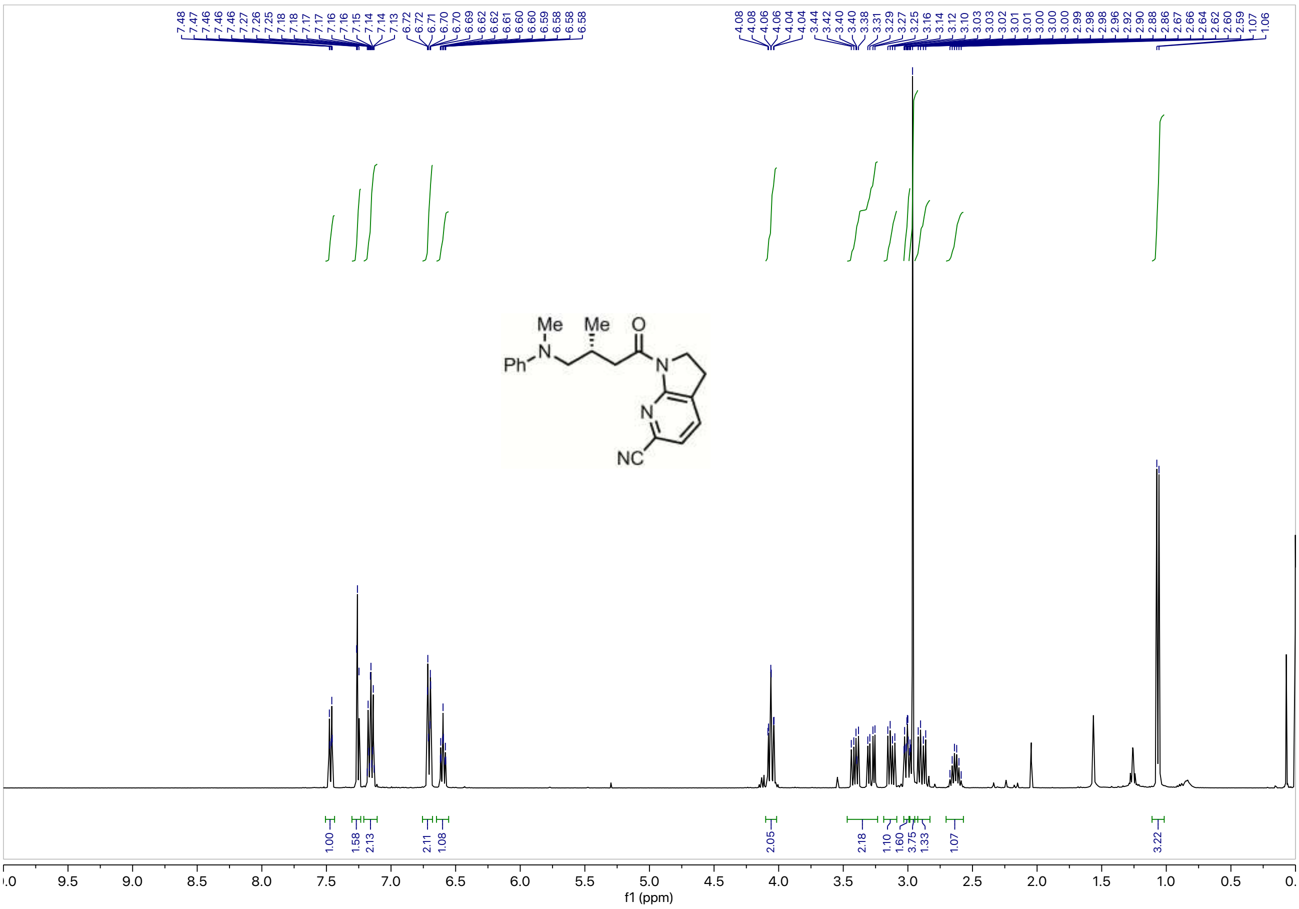

^{13}C NMR: **3fa**

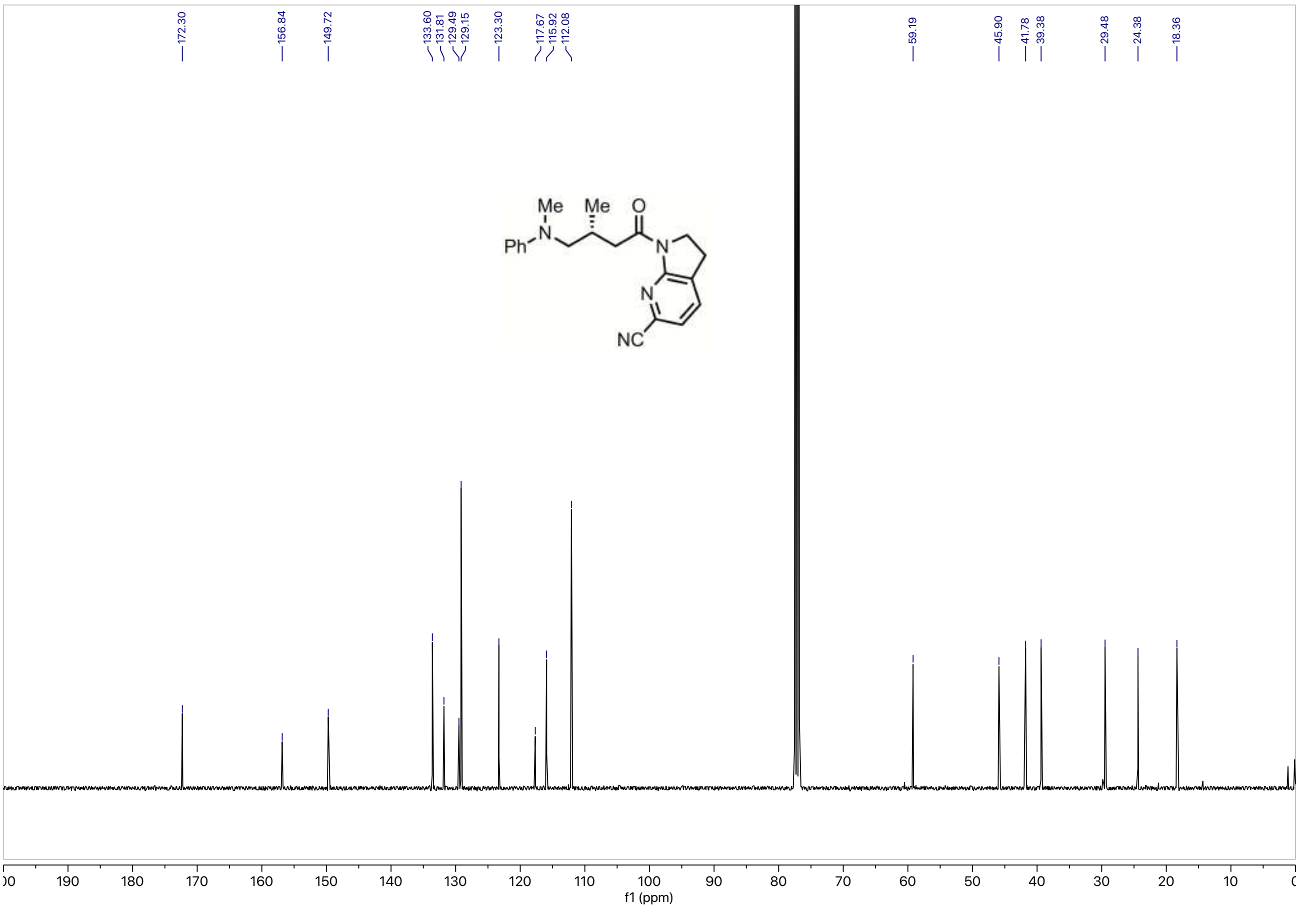

^{1}H NMR: **3ga**

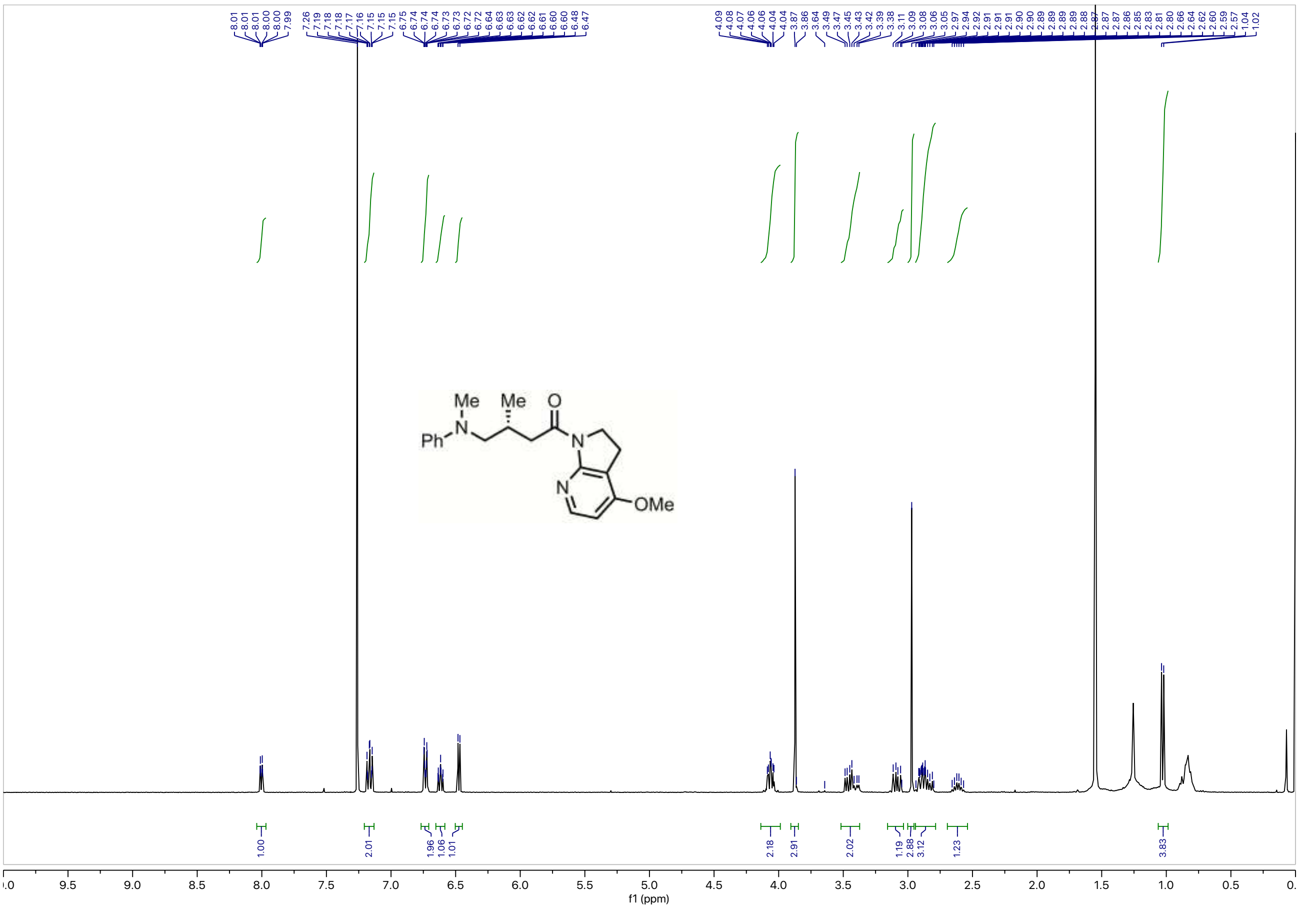

^{13}C NMR: **3ga**

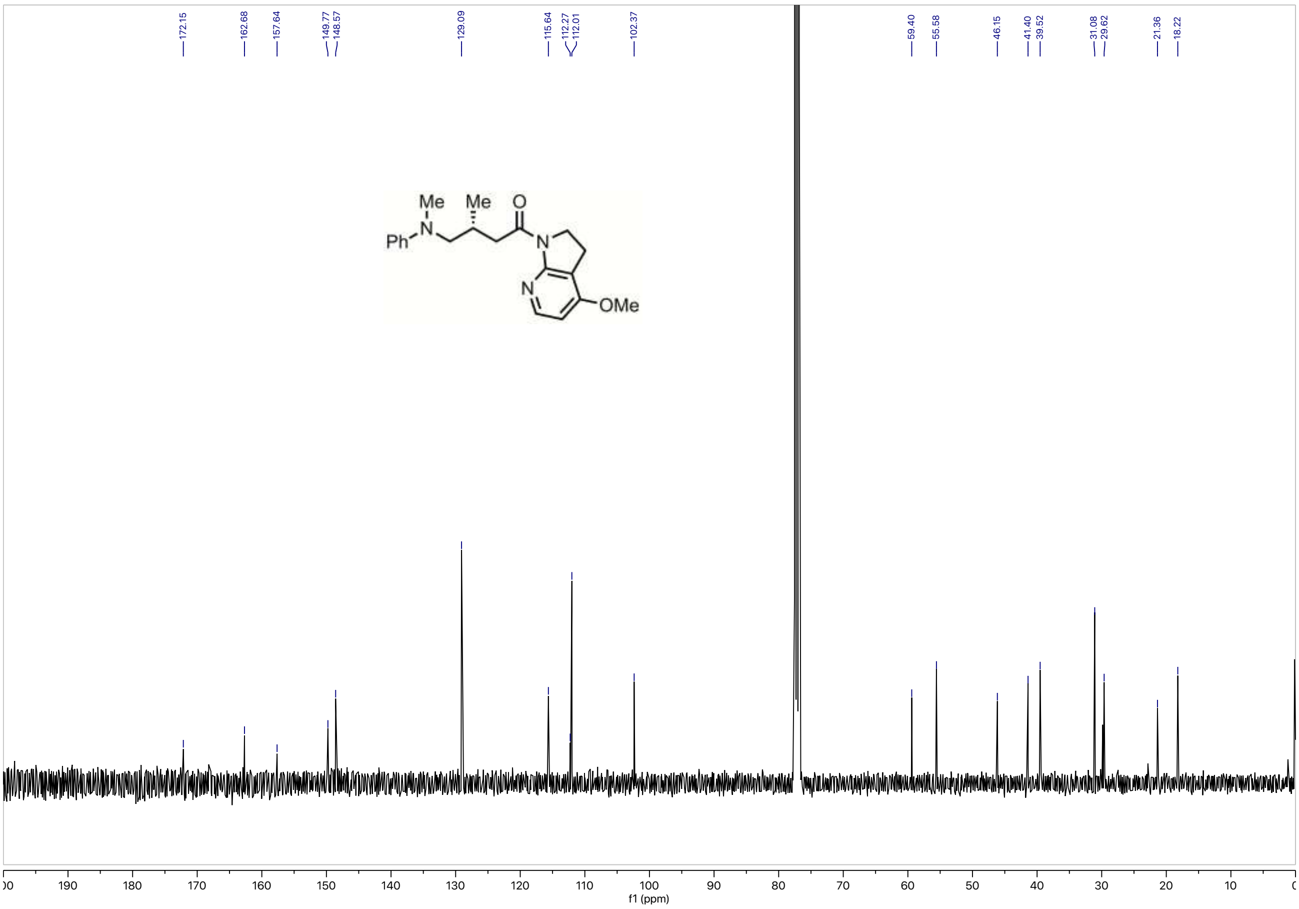

^{1}H NMR: **3ha**

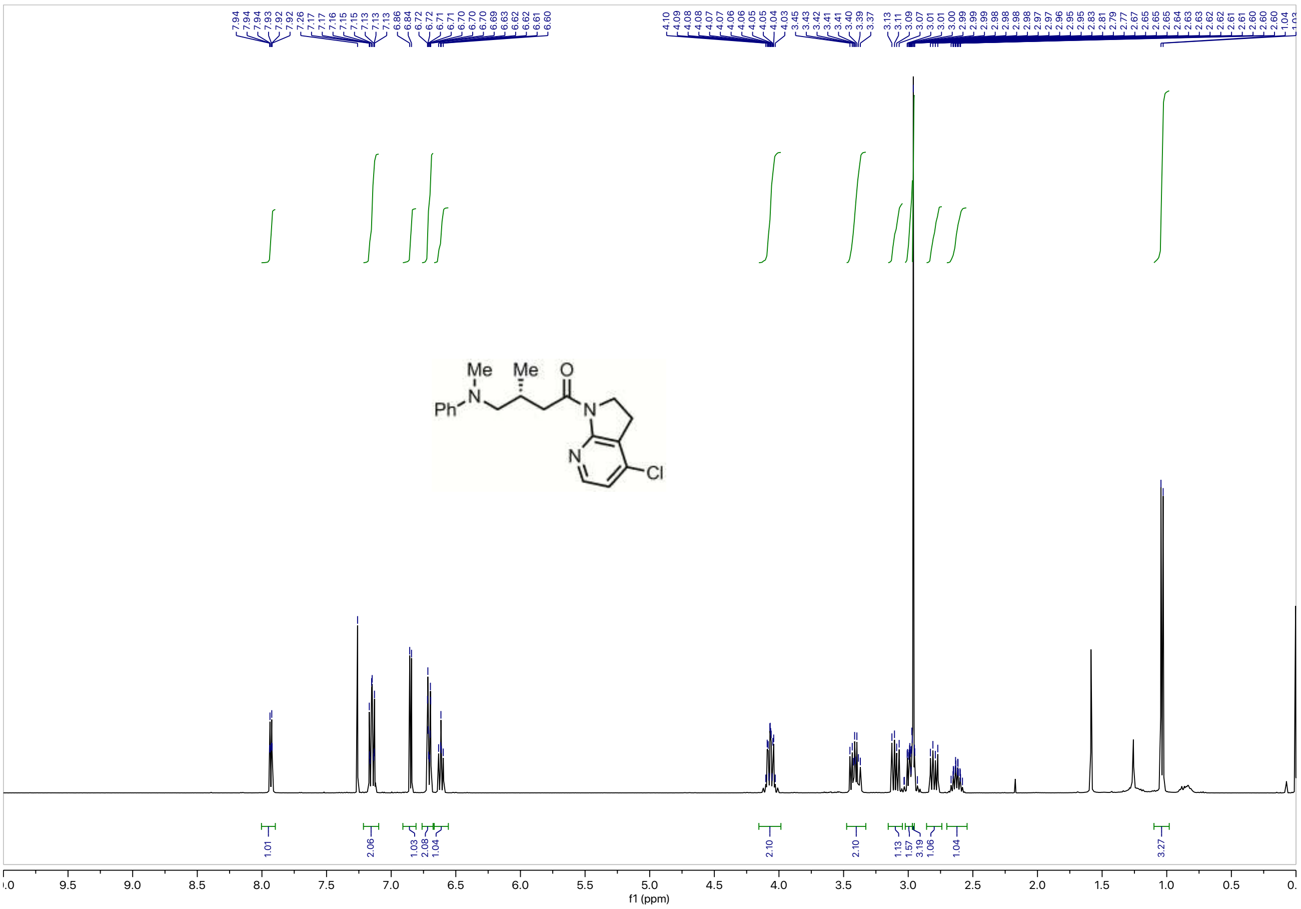

^{13}C NMR: **3ha**

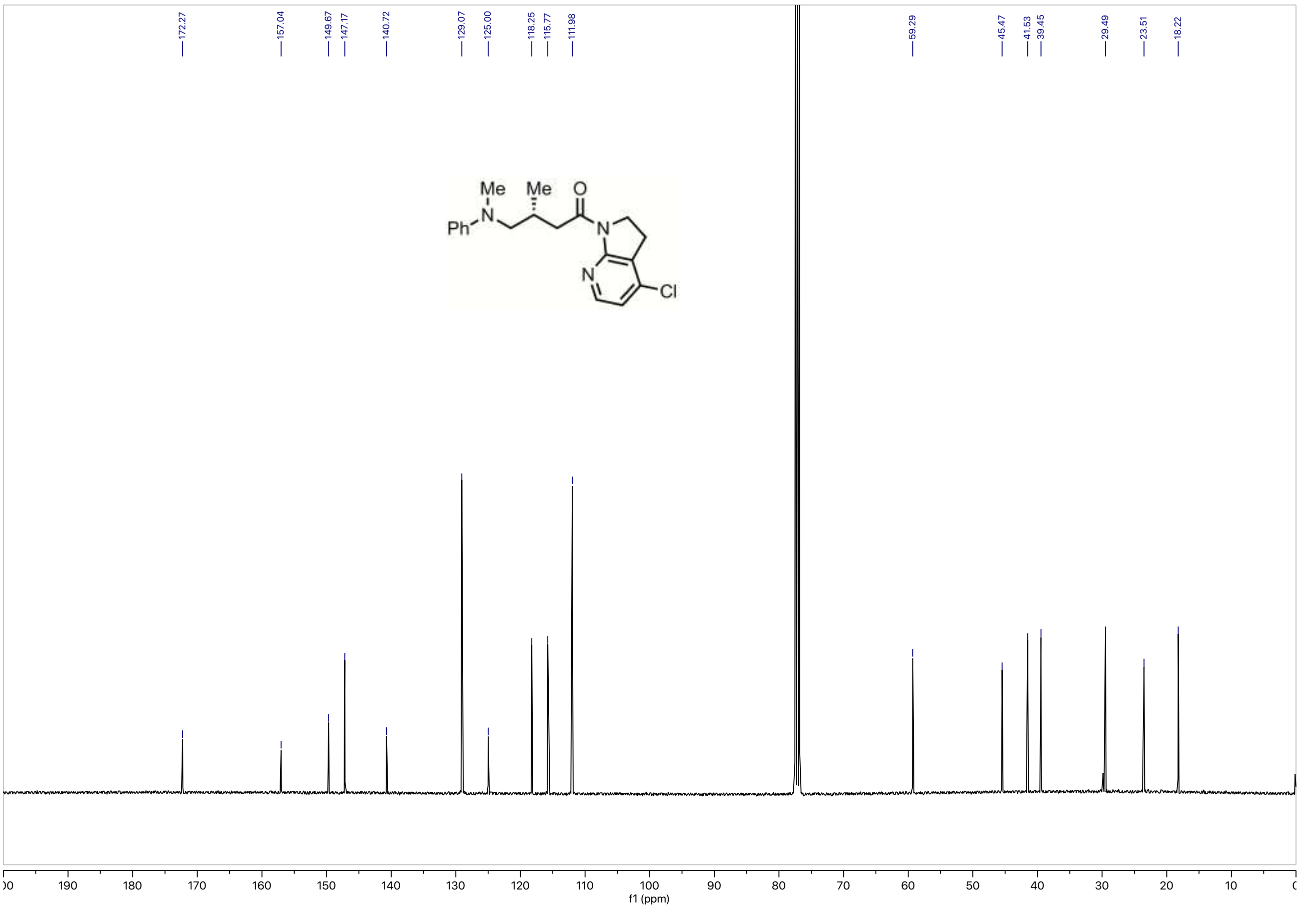

^{1}H NMR: **3ia**

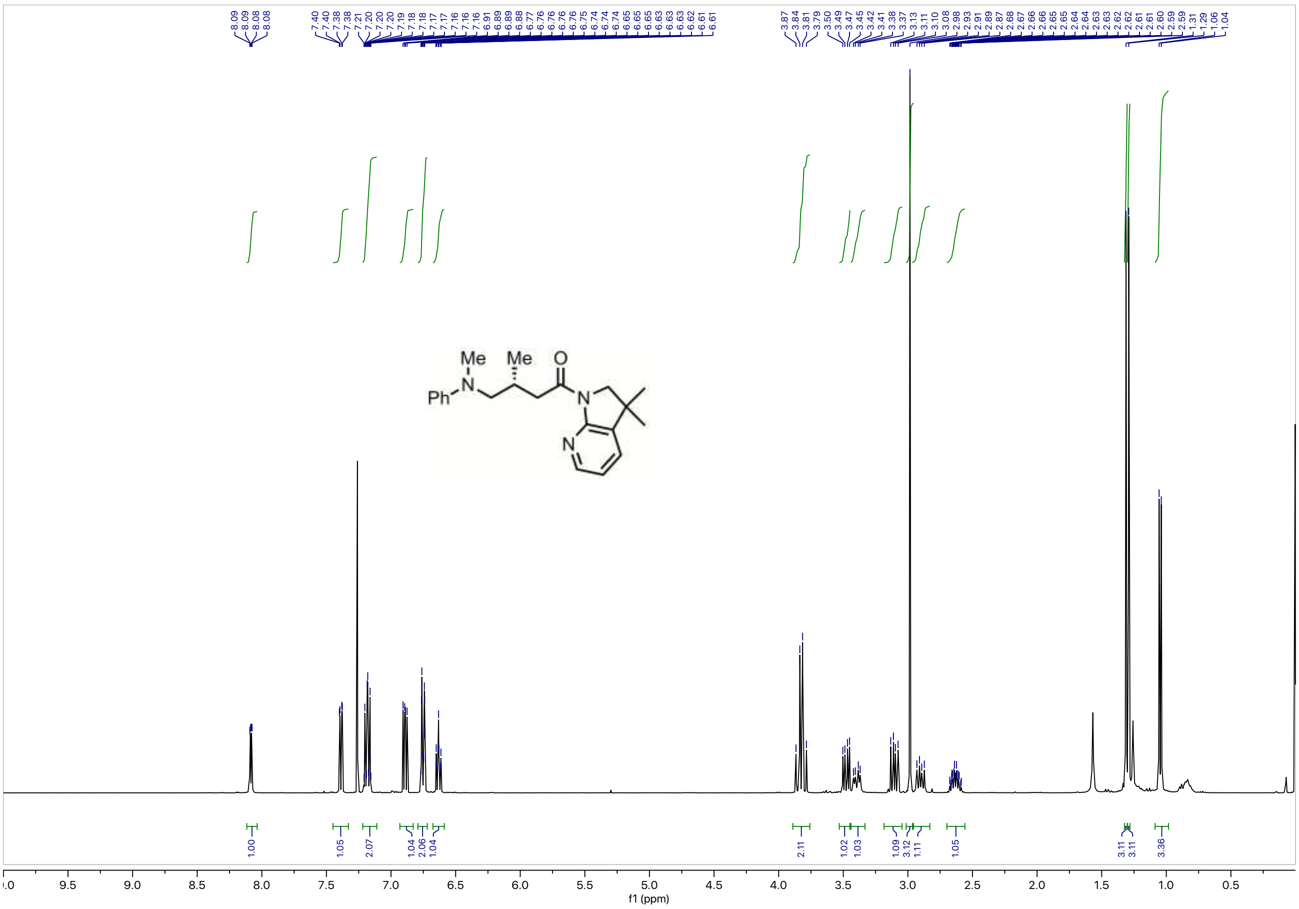

^{13}C NMR: **3ia**

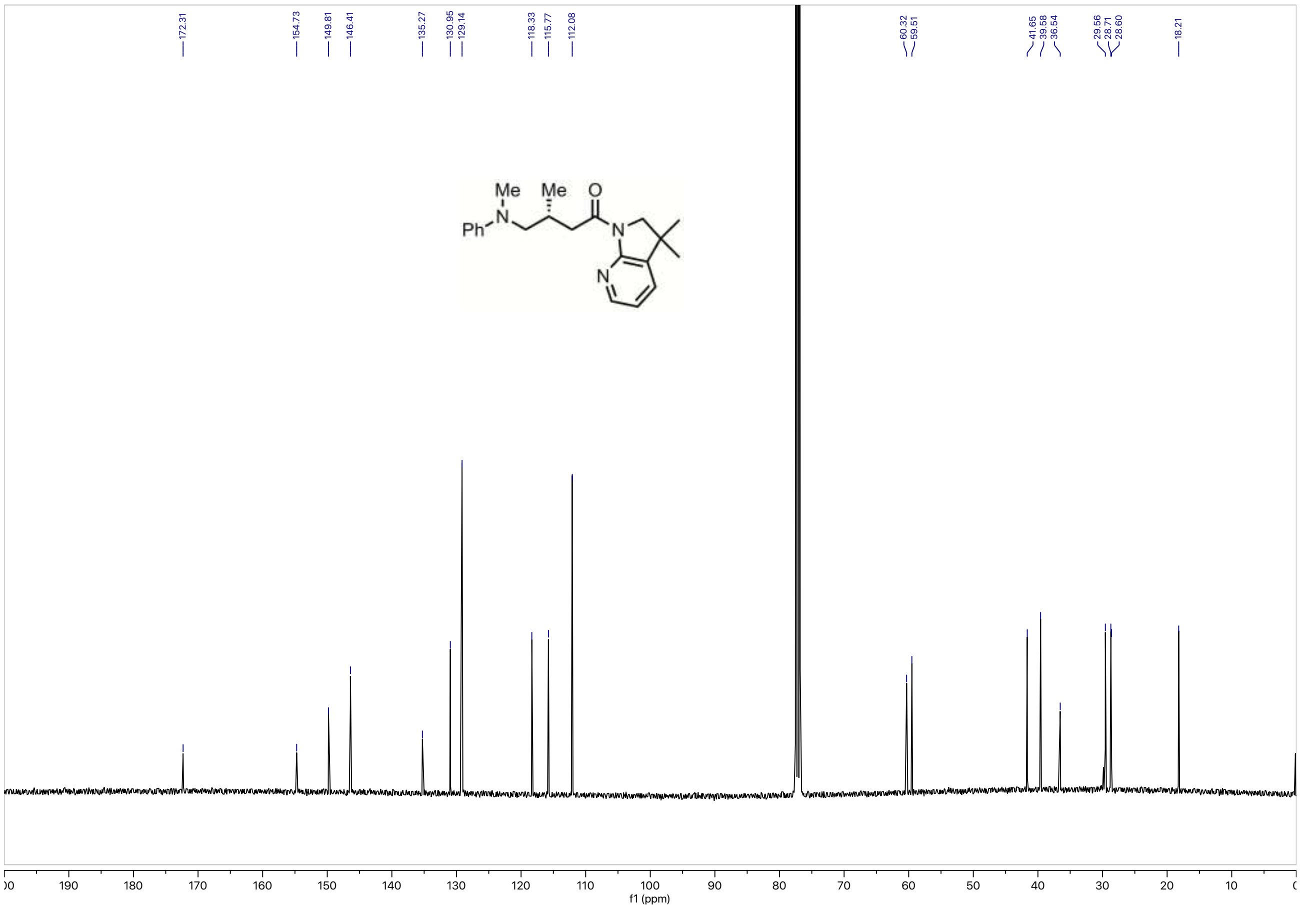

^{1}H NMR: **3ja**

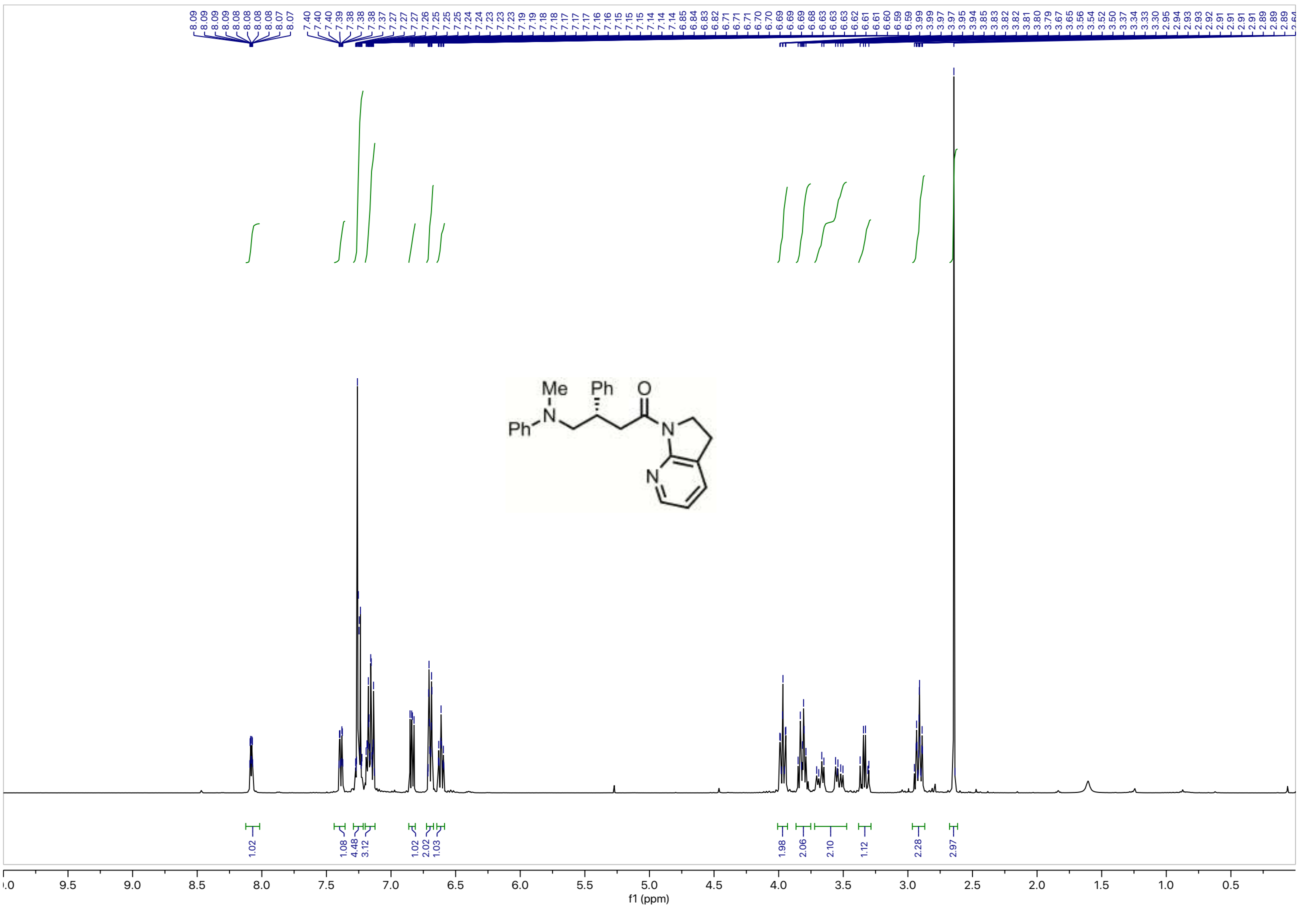

^{13}C NMR: **3ja**

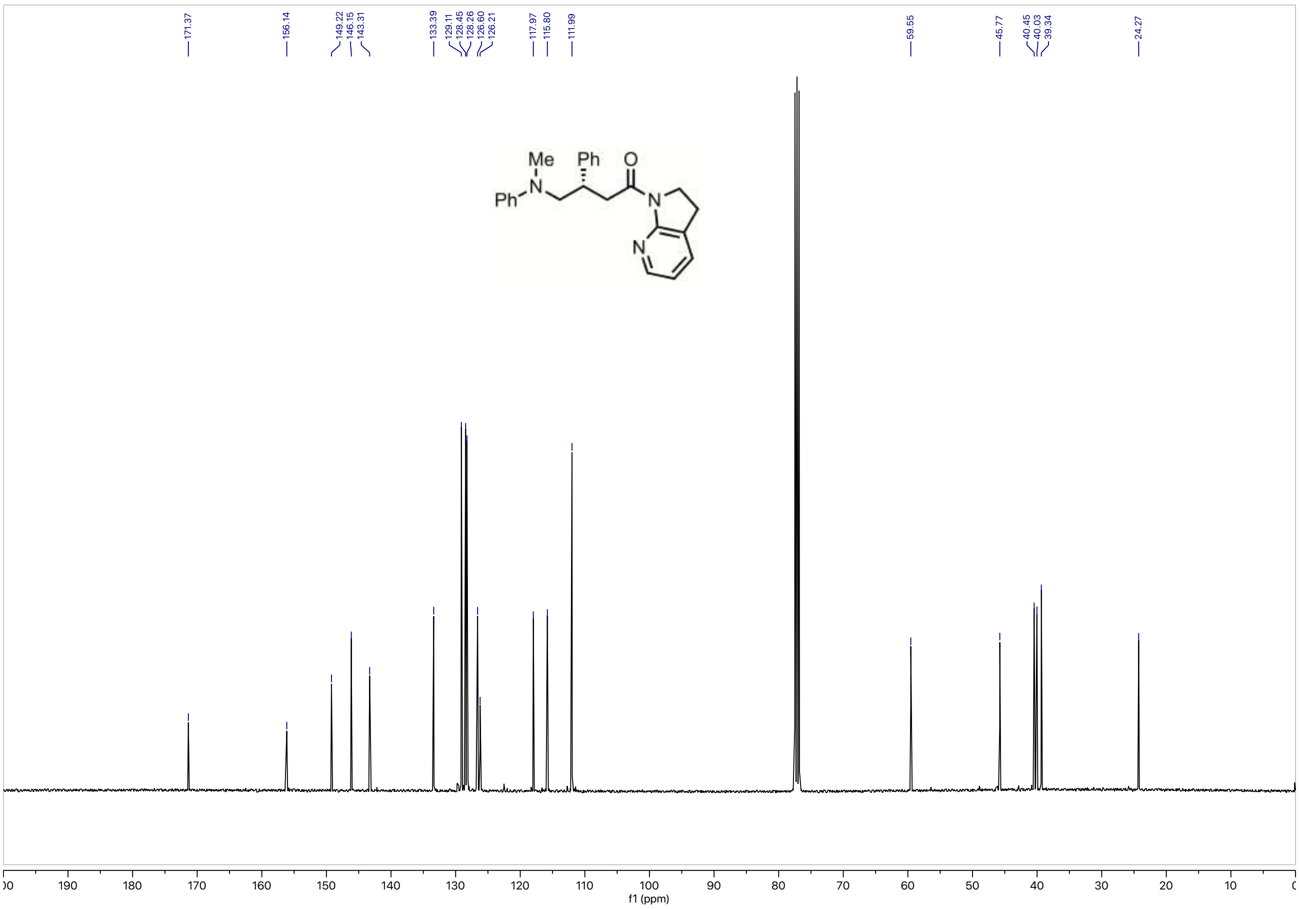

^{1}H NMR: **3ka** (Note: this compound is very unstable and difficult to purify)

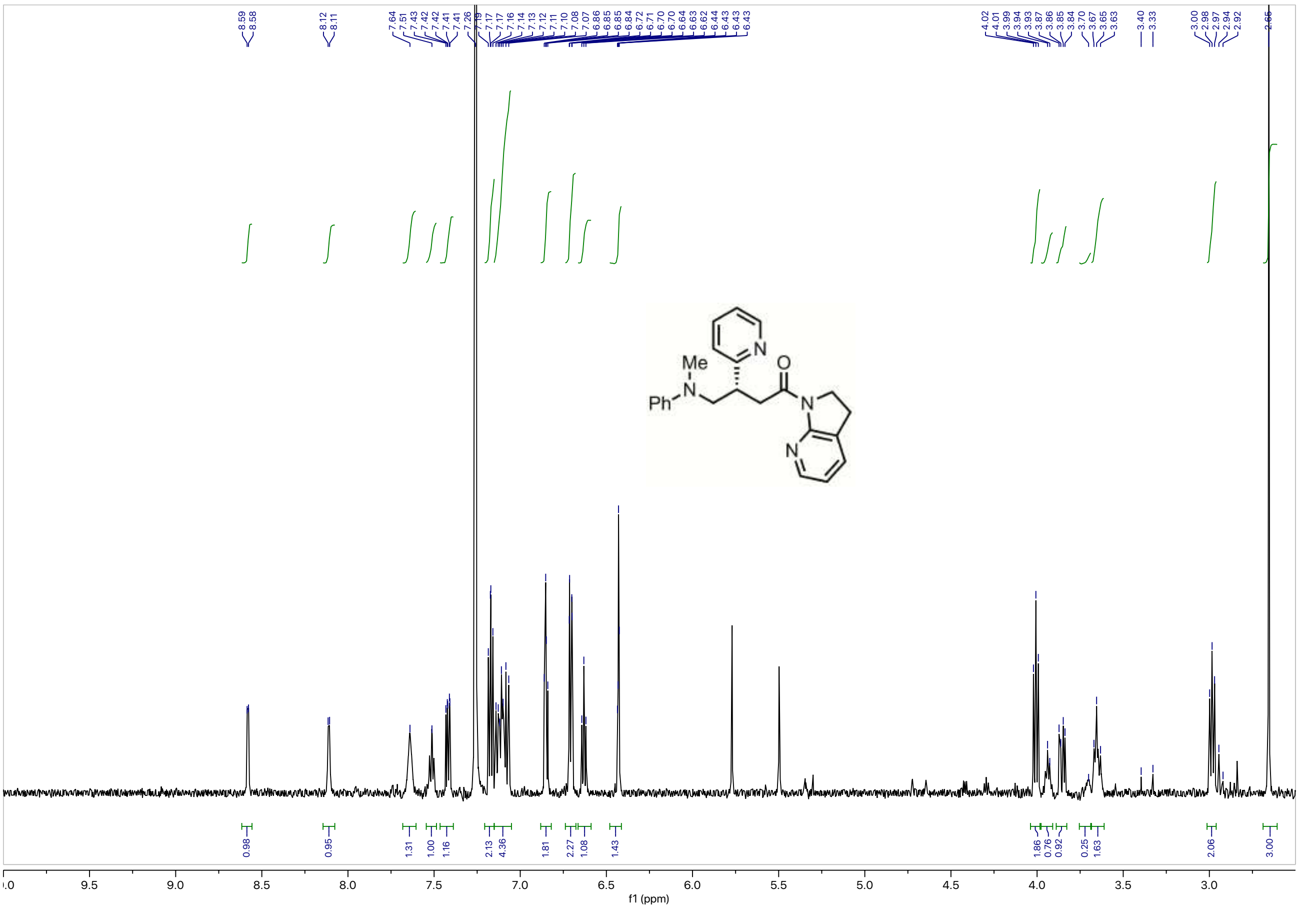

^{13}C NMR: **3ka** (Note: this compound is very unstable and difficult to purify)

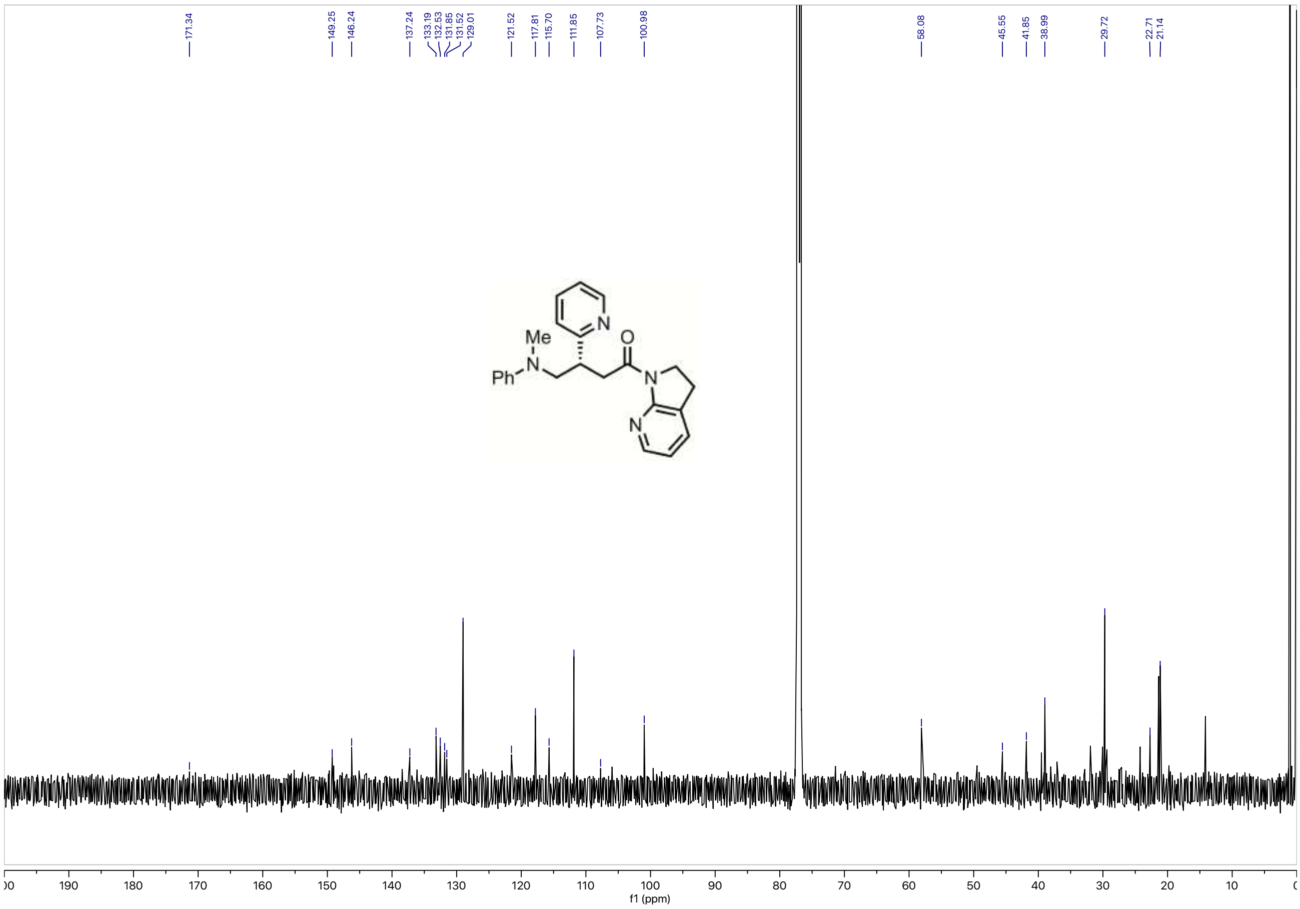

^{1}H NMR: **3la** (from **1l**, *E*-isomer, and (*R*)-DM-Segphos)

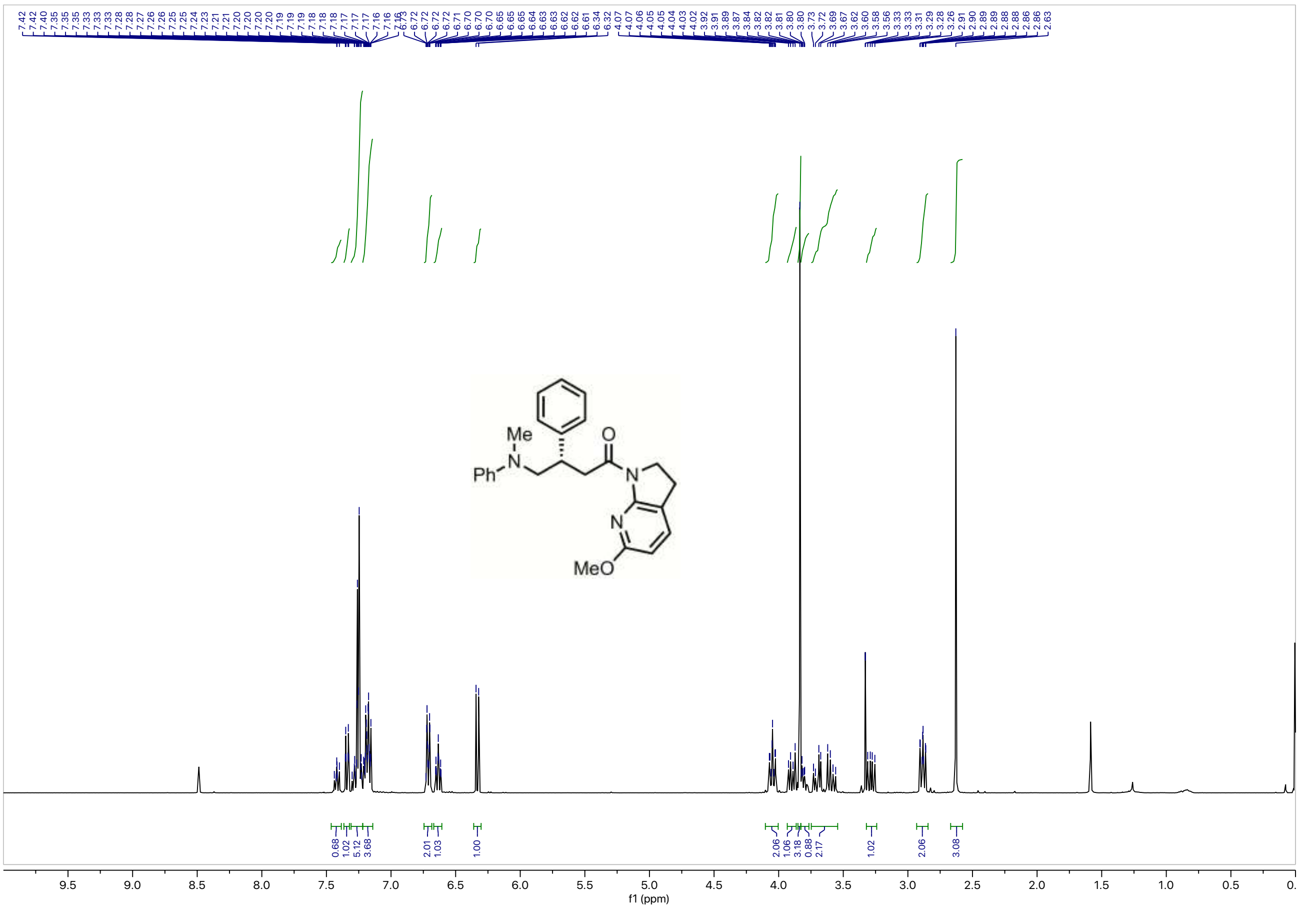

^{13}C NMR: **3la** (from **1l**, *E*-isomer, and (*R*)-DM-Segphos)

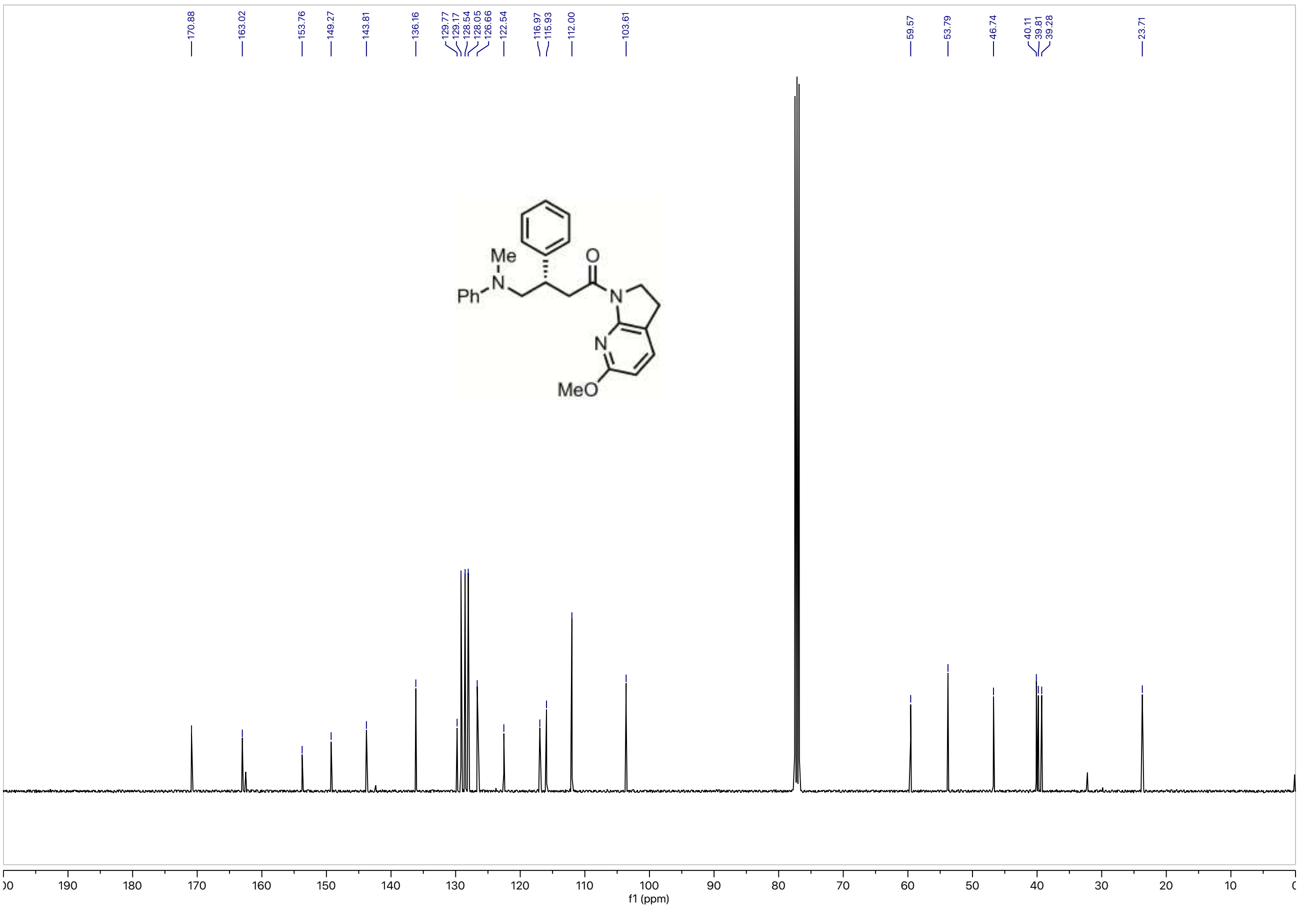

^{1}H NMR: **3la'** (from **1l**, *E*-isomer, and (*S*)-DM-Segphos)

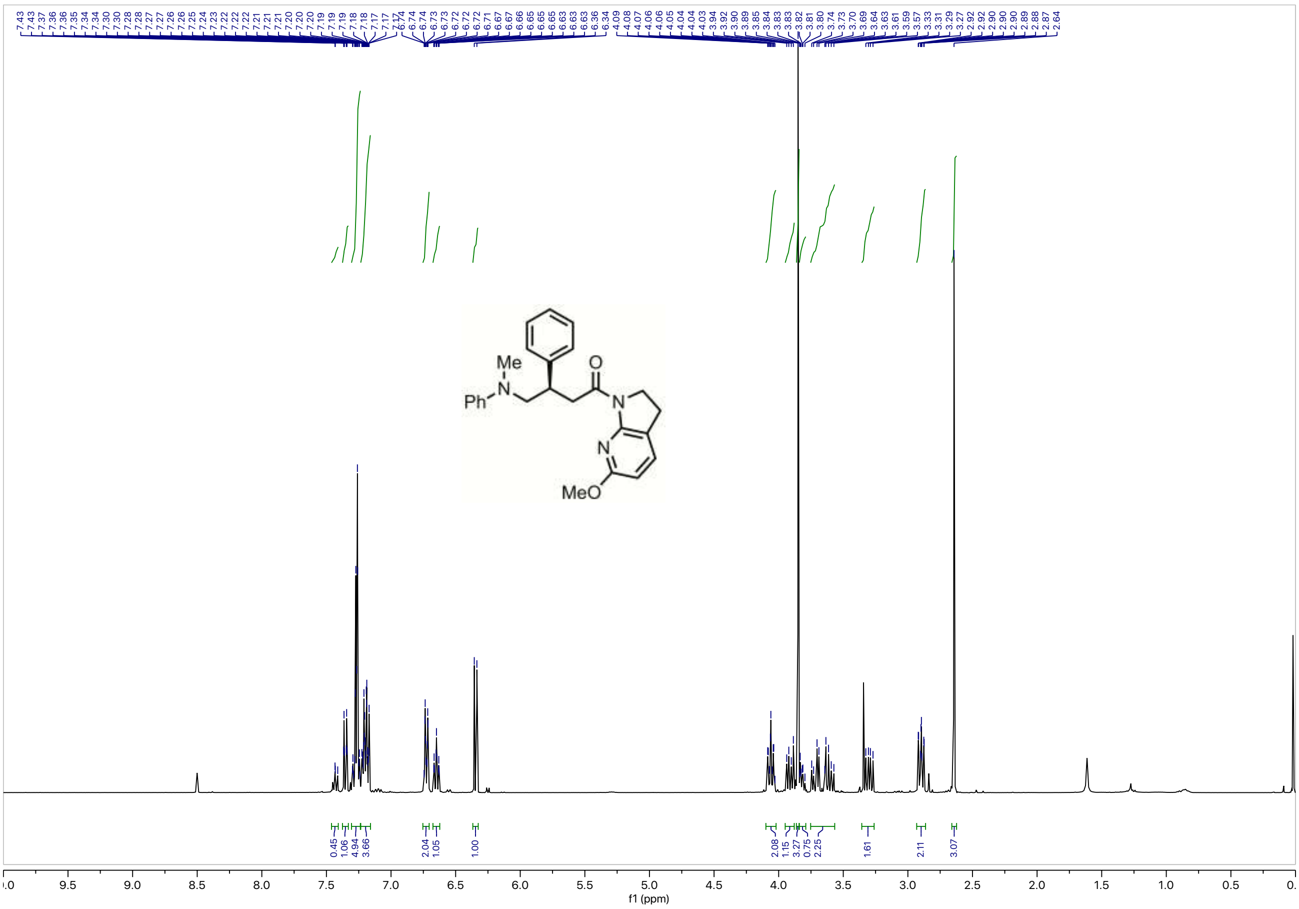

^{13}C NMR: **3la'** (from **1l**, *E*-isomer, and (*S*)-DM-Segphos)

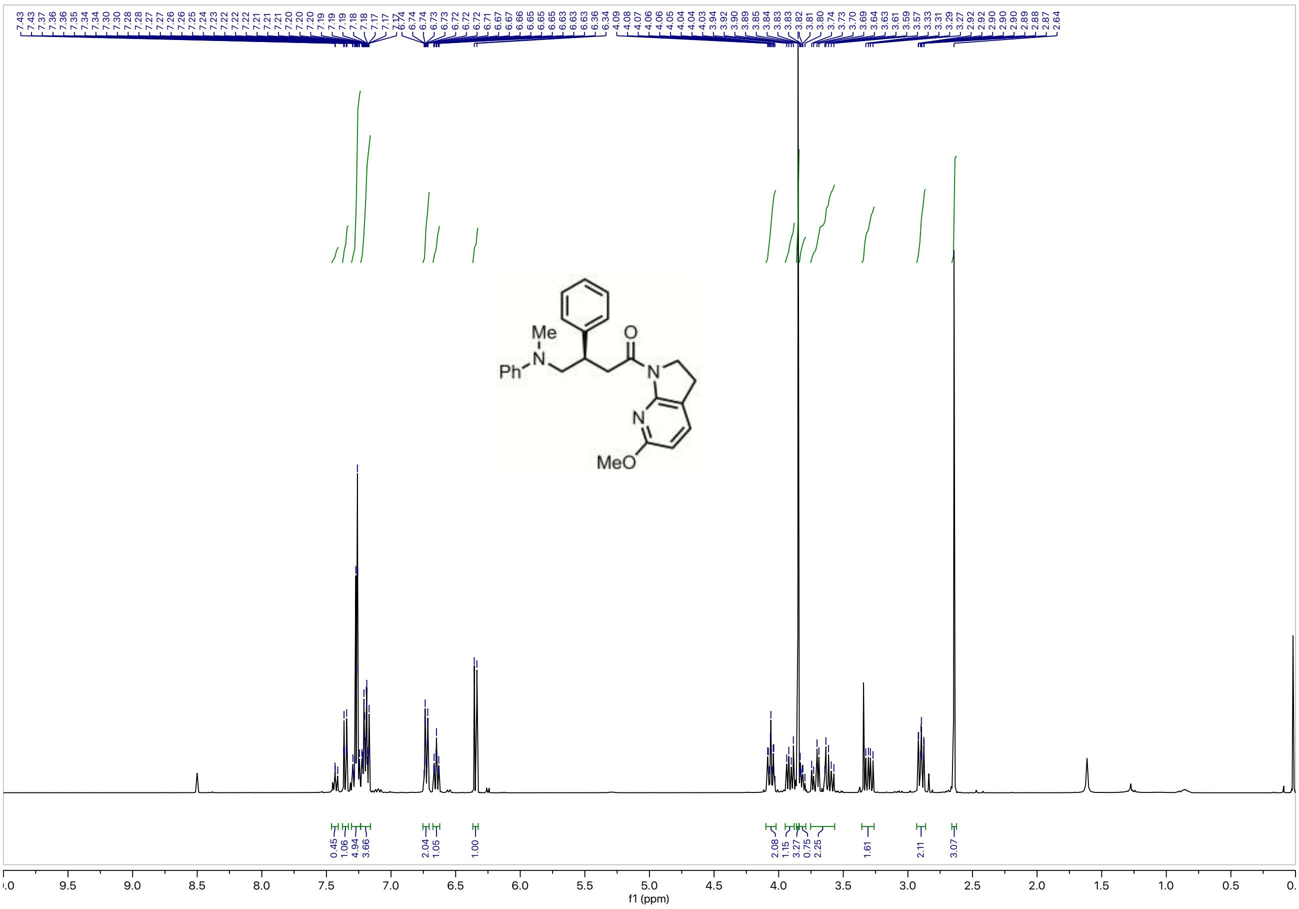

^{1}H NMR: **3ma**

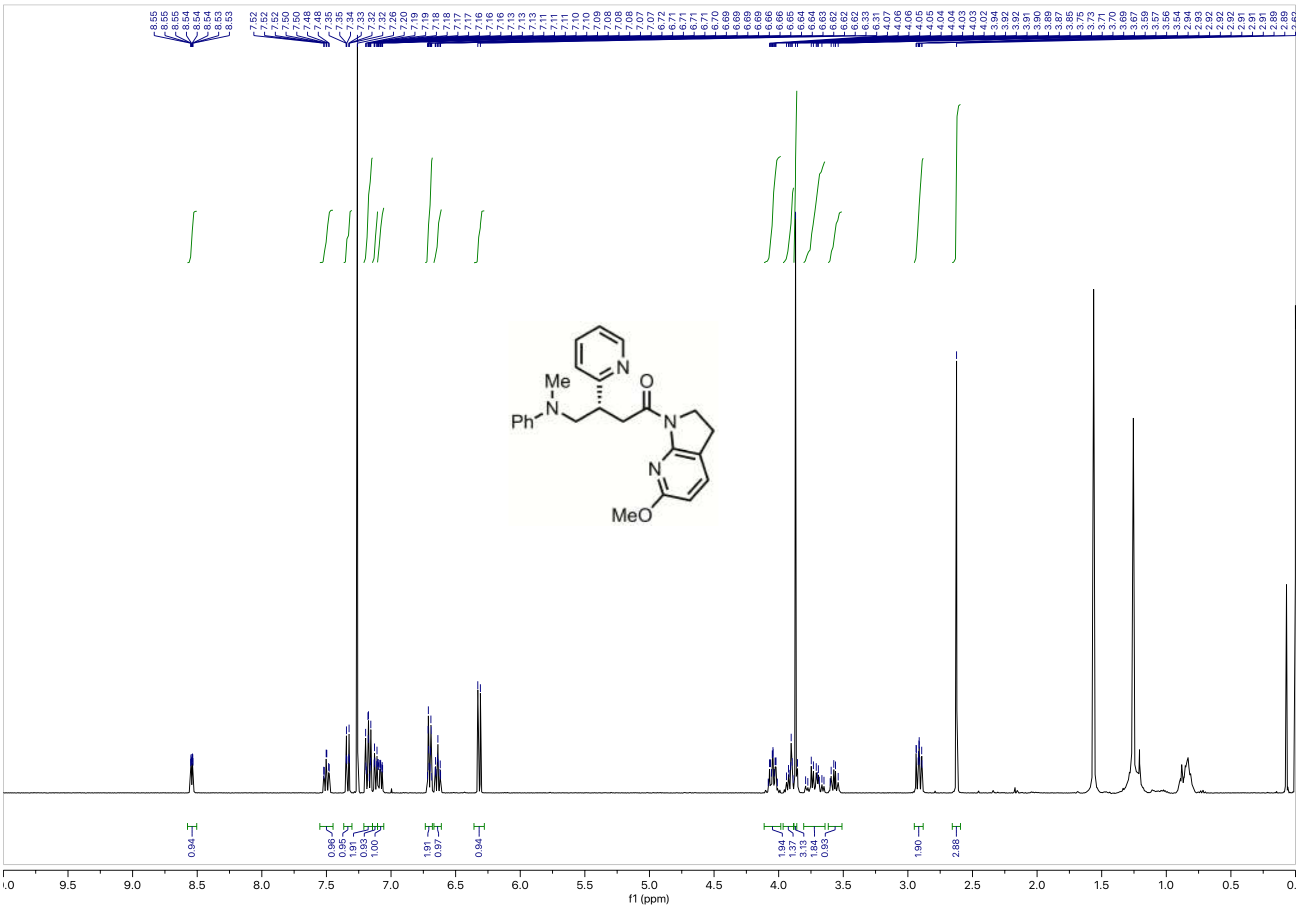

^{13}C NMR: **3ma**

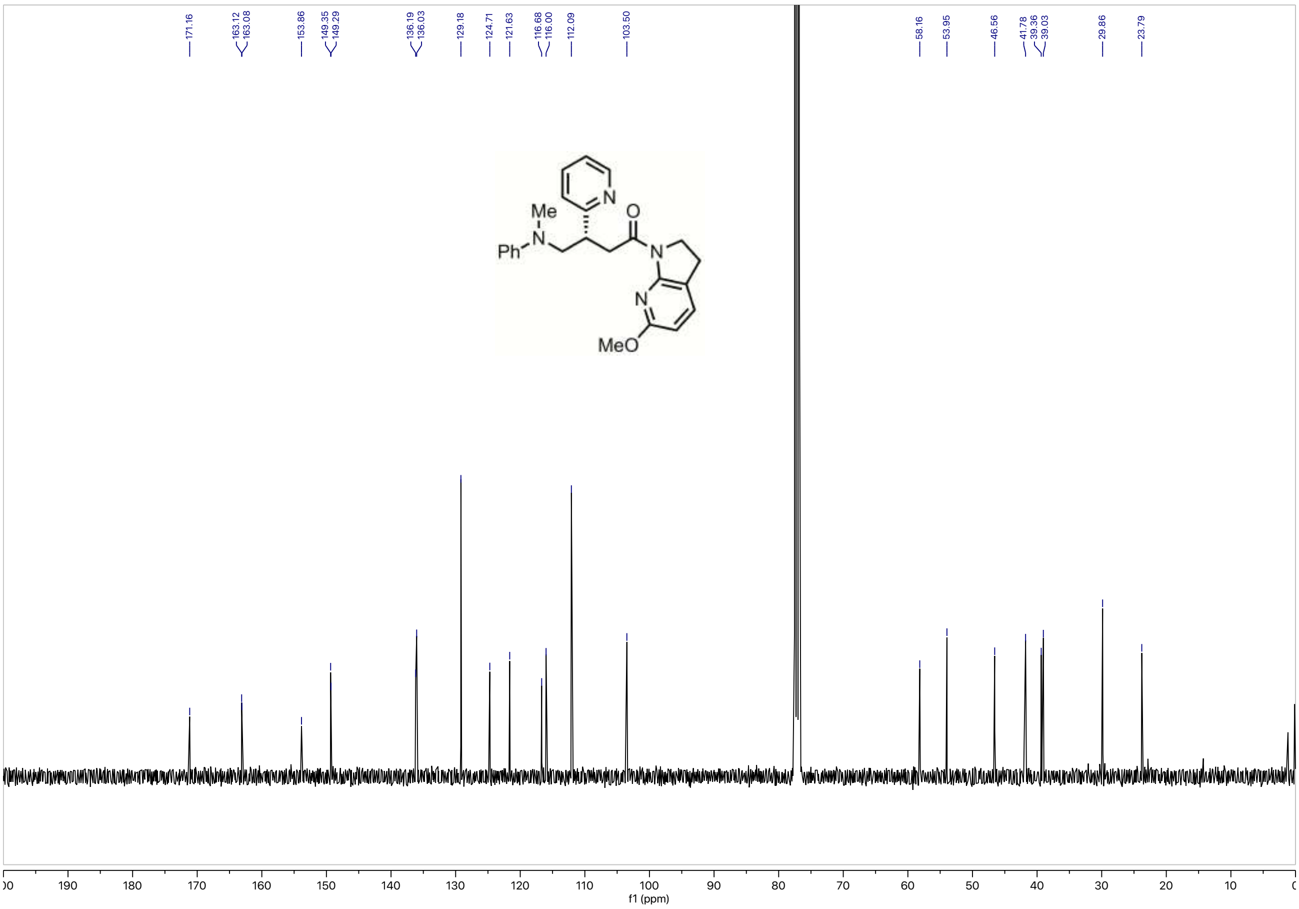

^{1}H NMR: **3na**

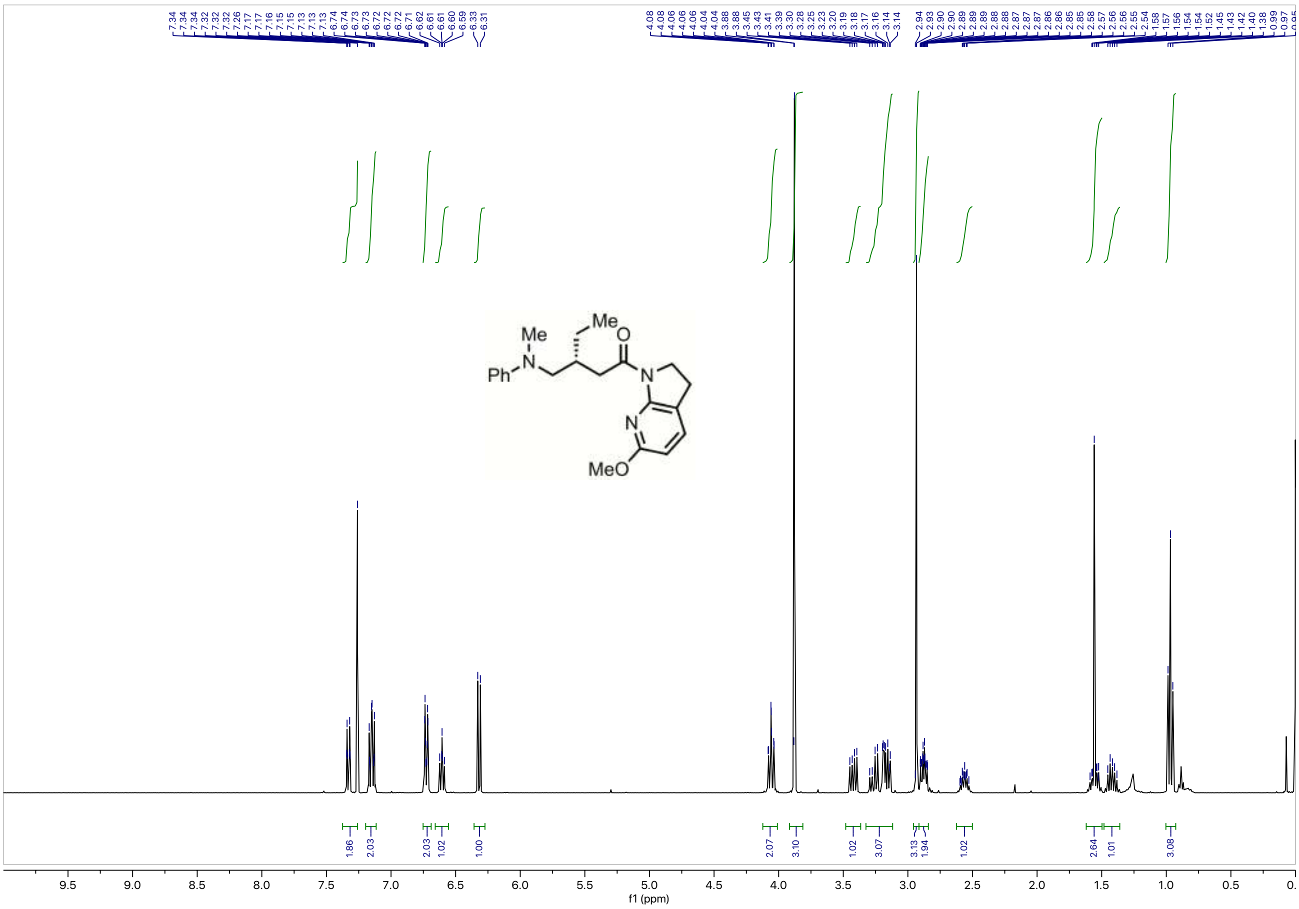

^{13}C NMR: **3na**

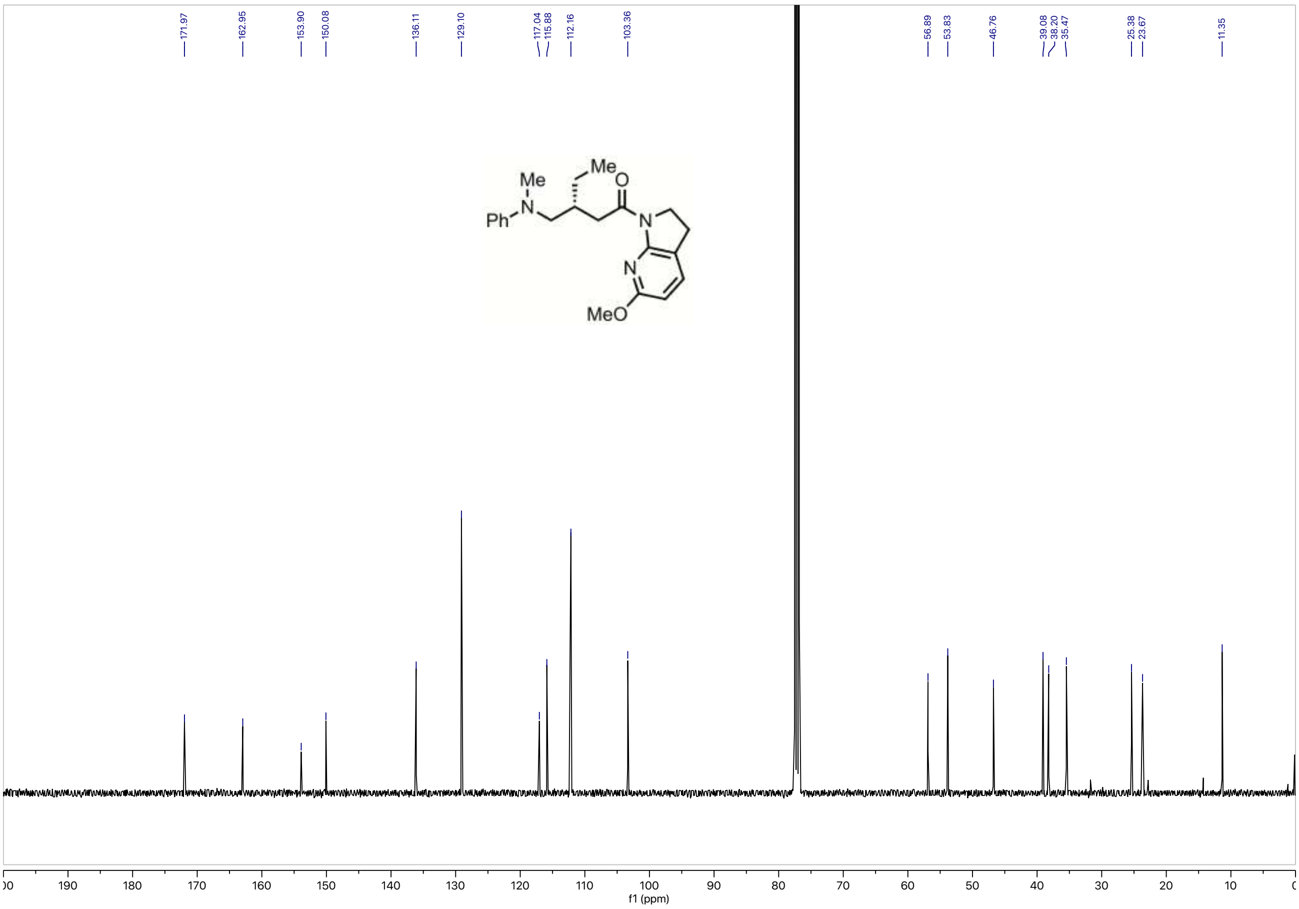

^{1}H NMR: **3oa**

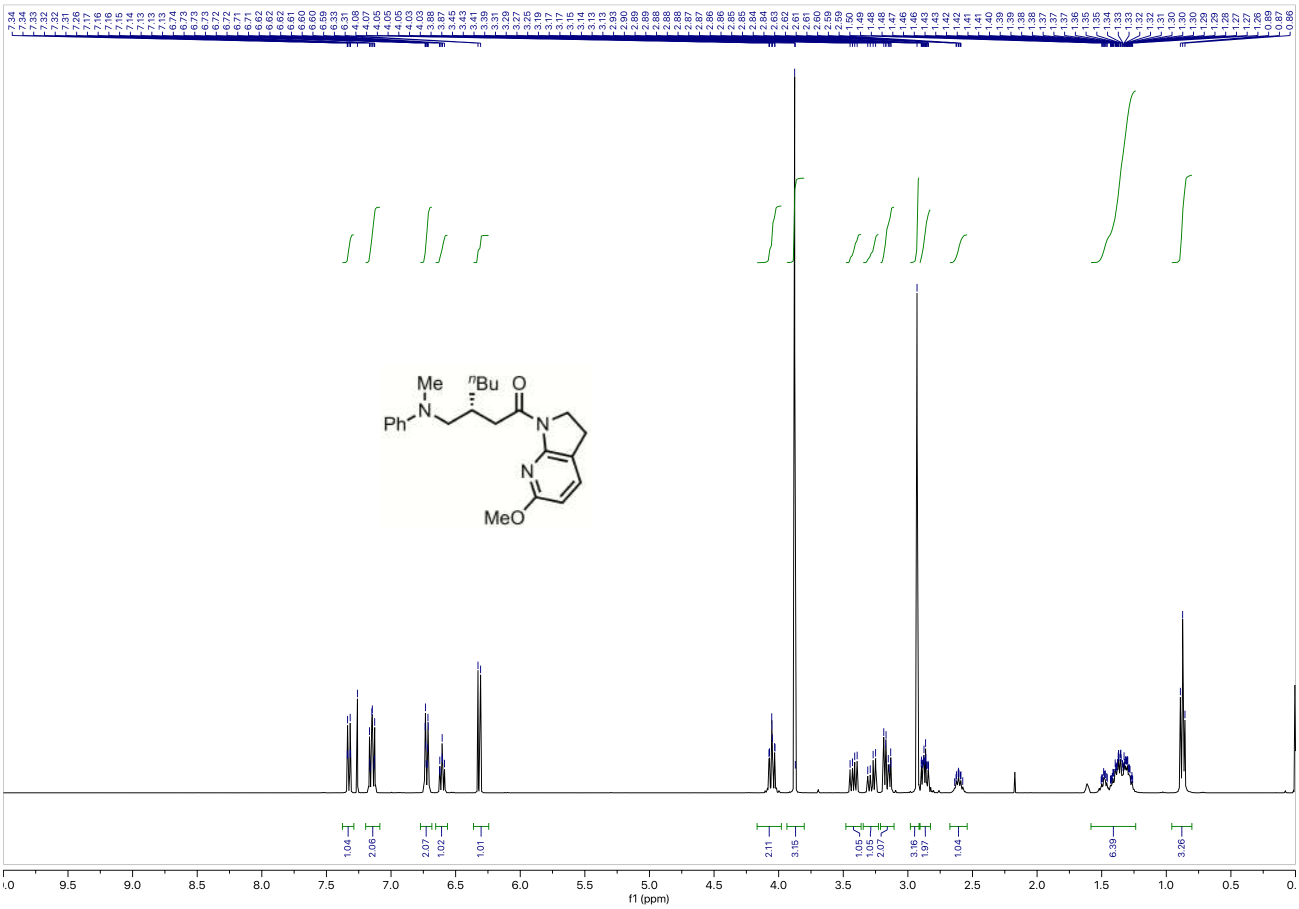

^{13}C NMR: **3oa**

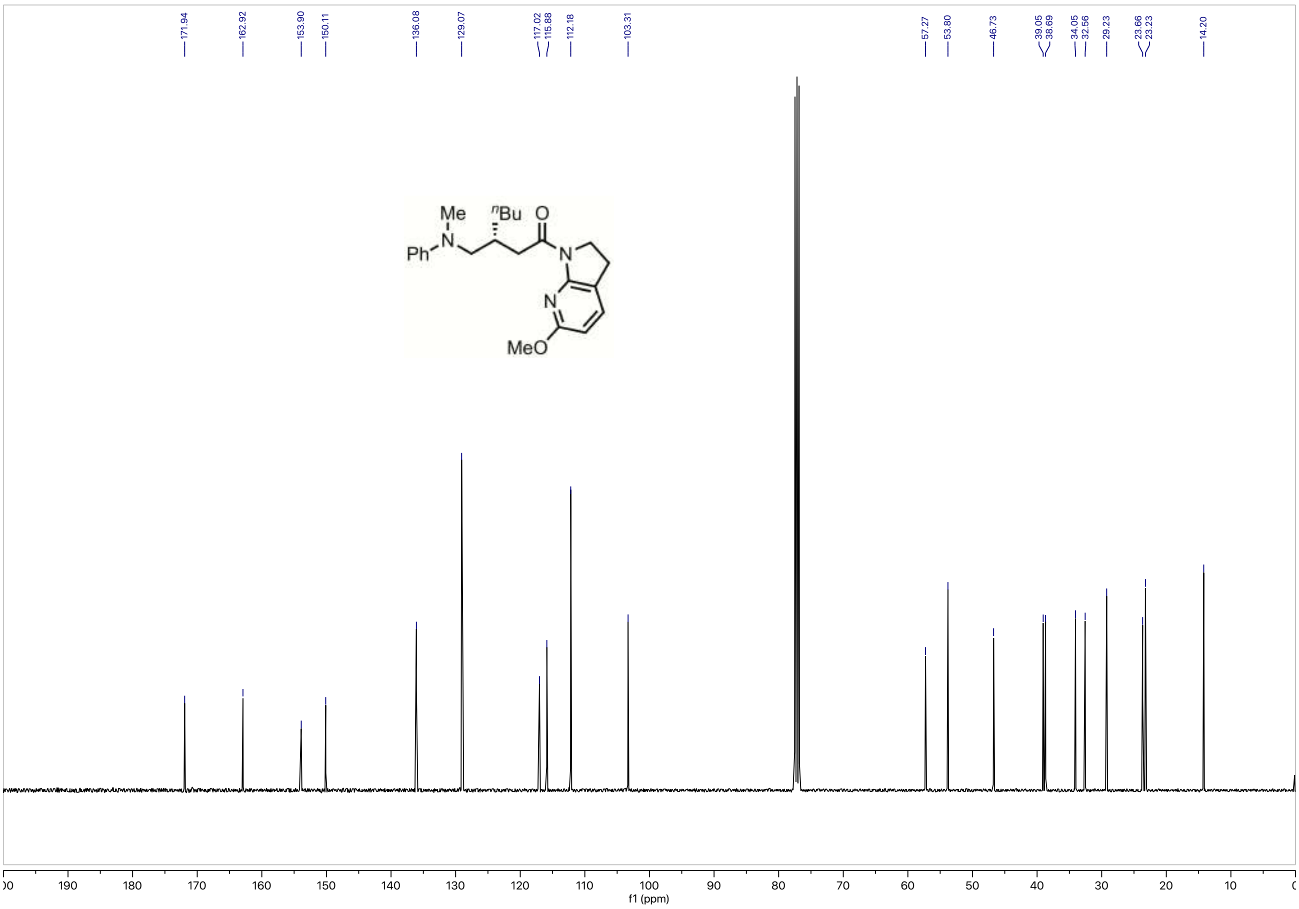

^{1}H NMR: **3pa**

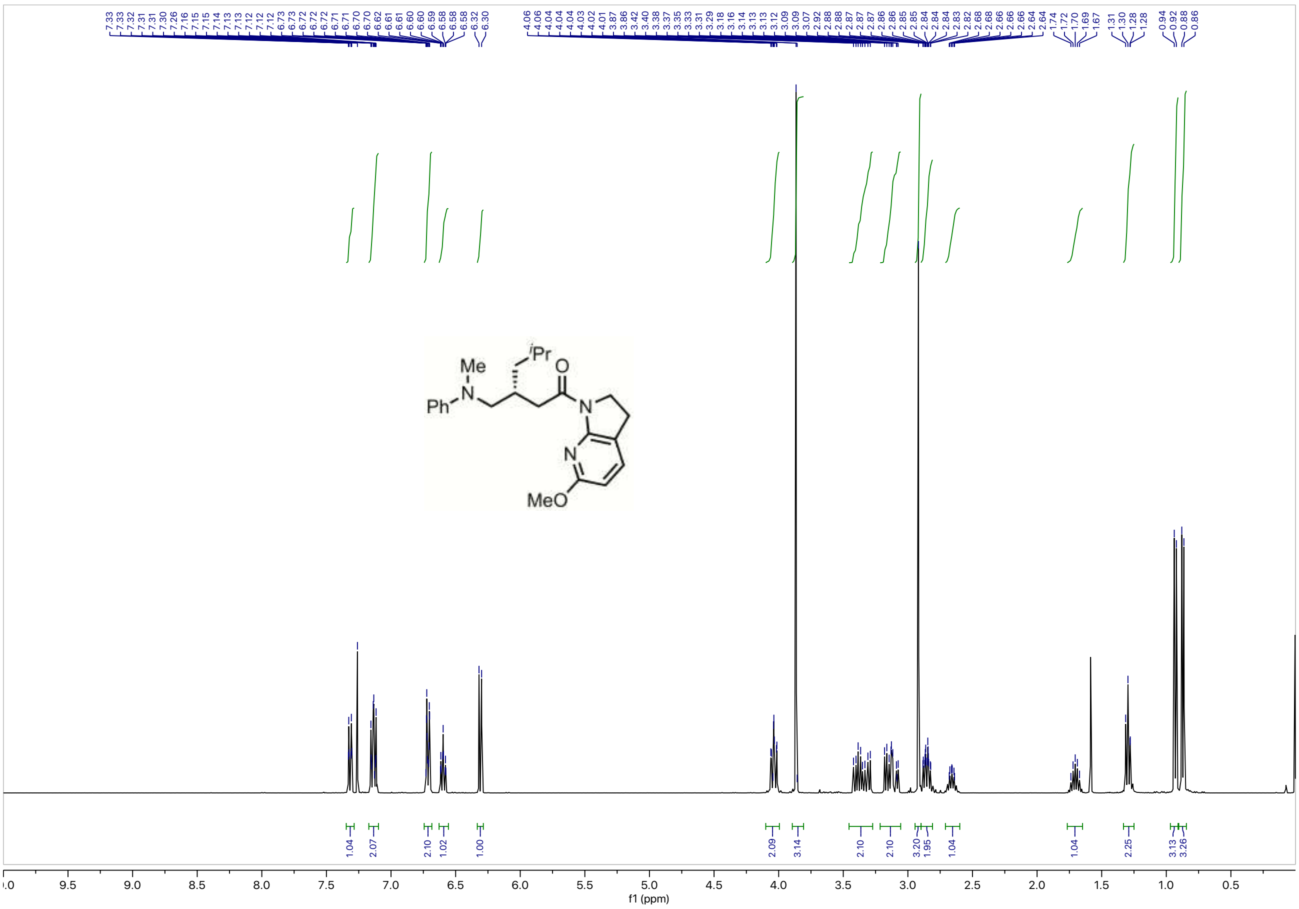

^{13}C NMR: **3pa**

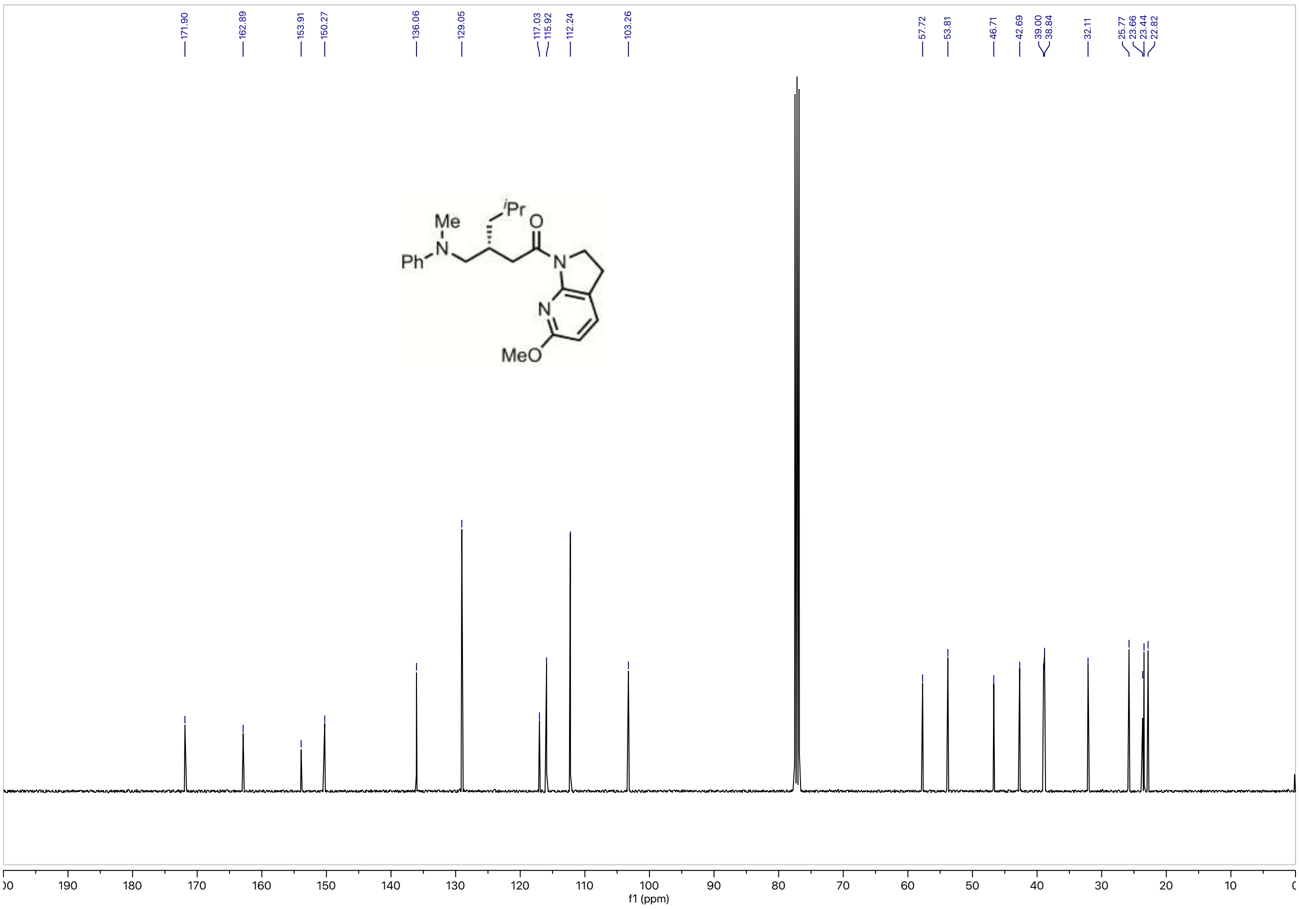

^{1}H NMR: **3qa**

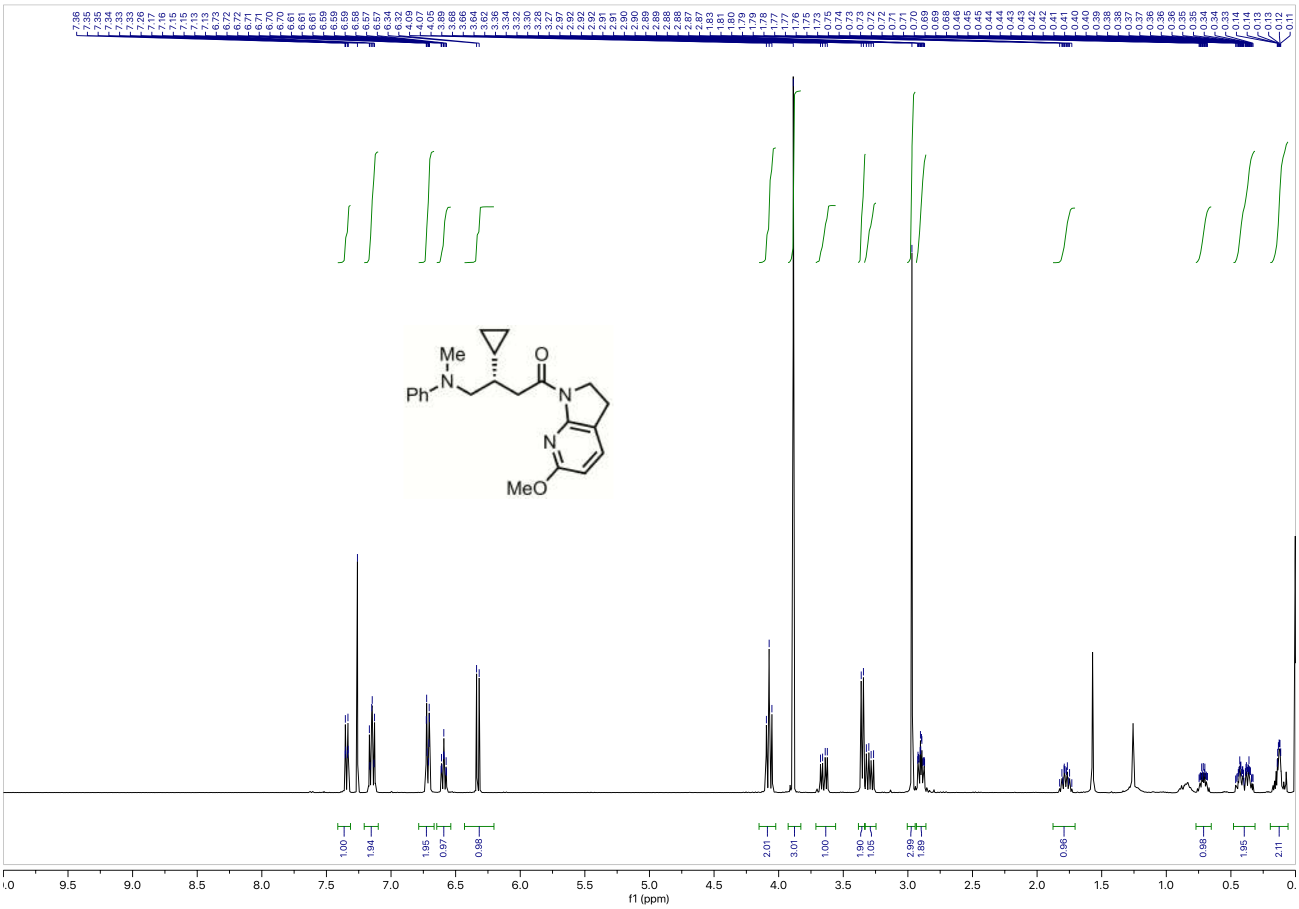

^{13}C NMR: **3qa**

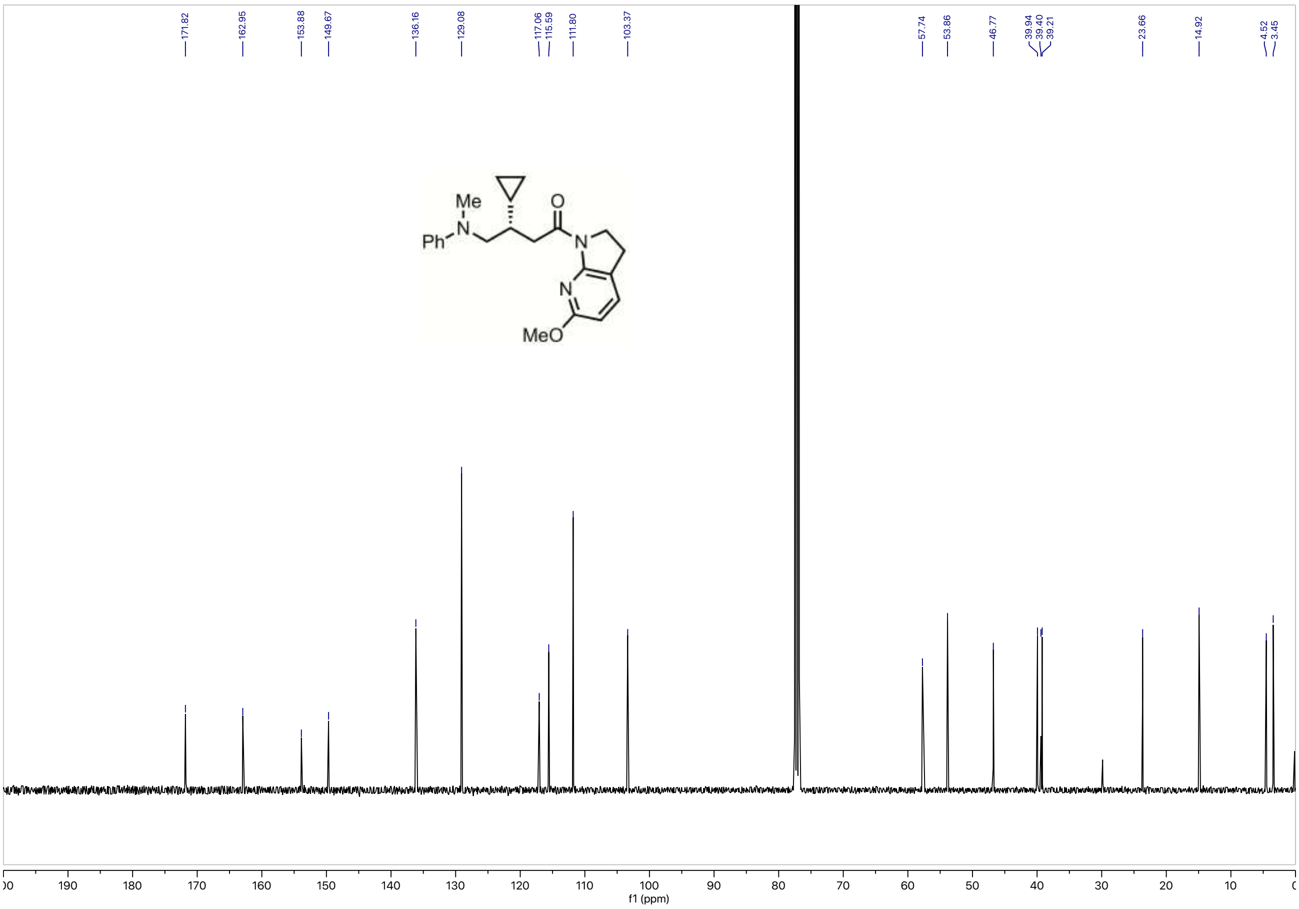

^{1}H NMR: **3ra**

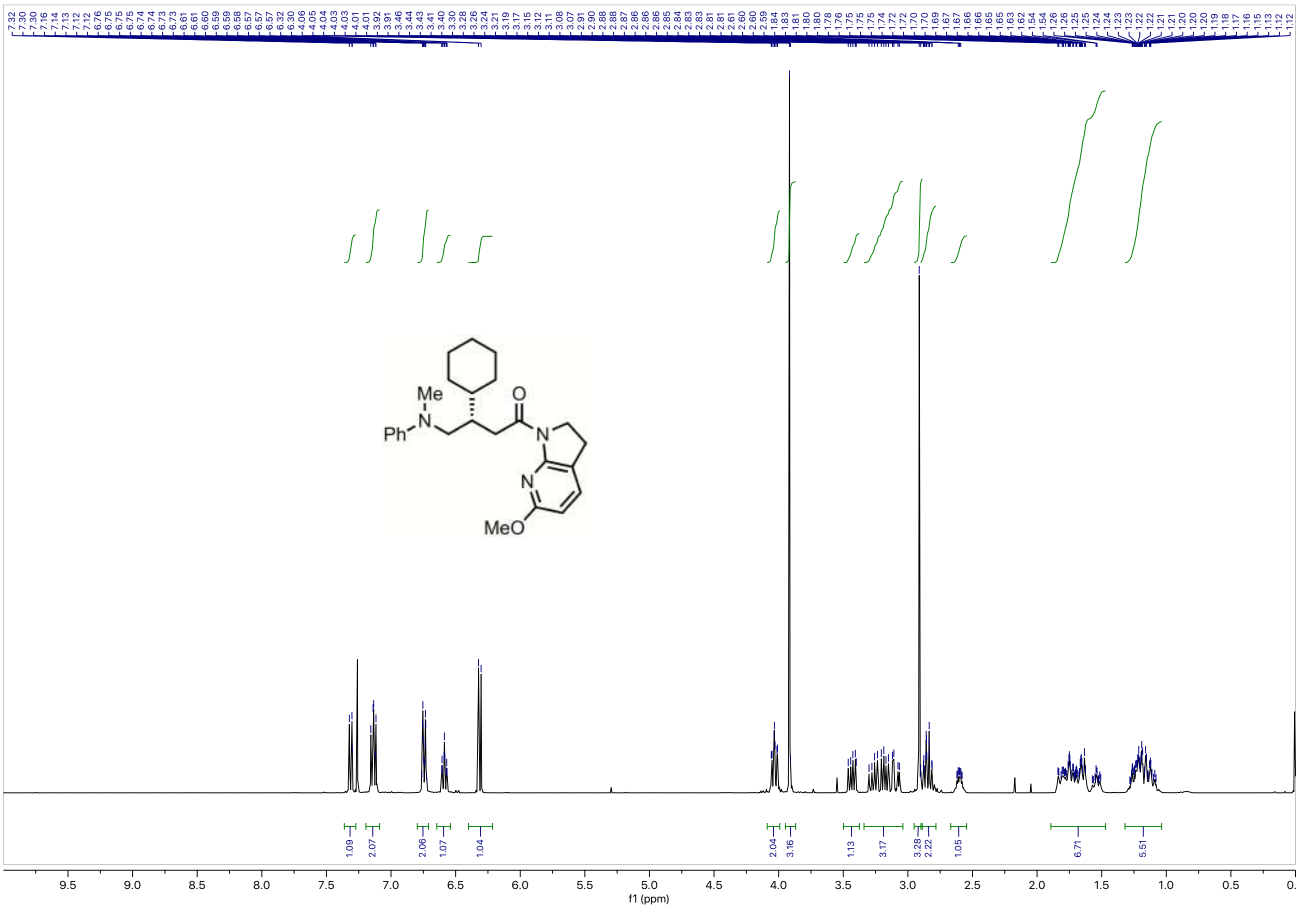

^{13}C NMR: **3ra**

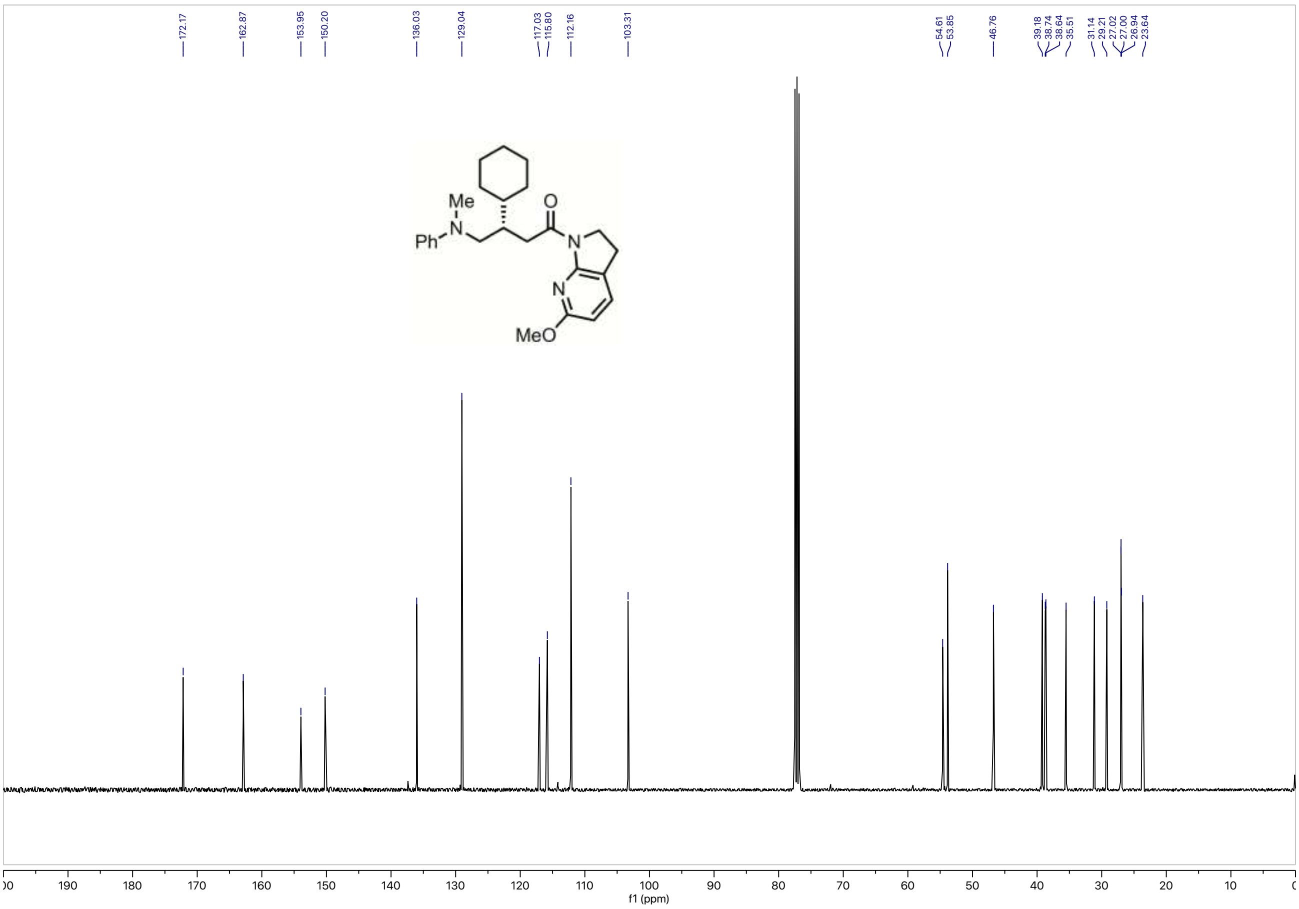

^{1}H NMR: **3sa**

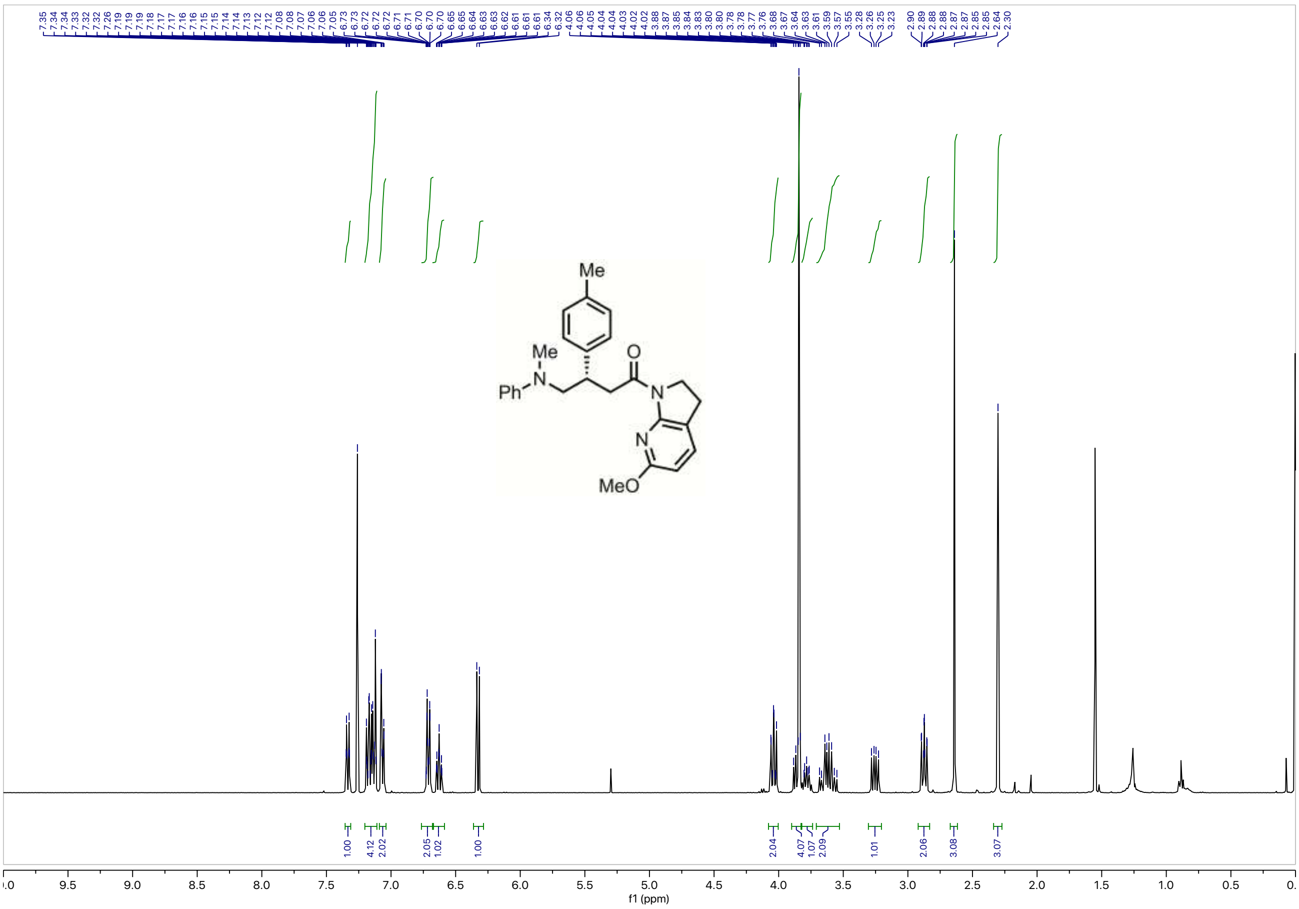

^{13}C NMR: **3sa**

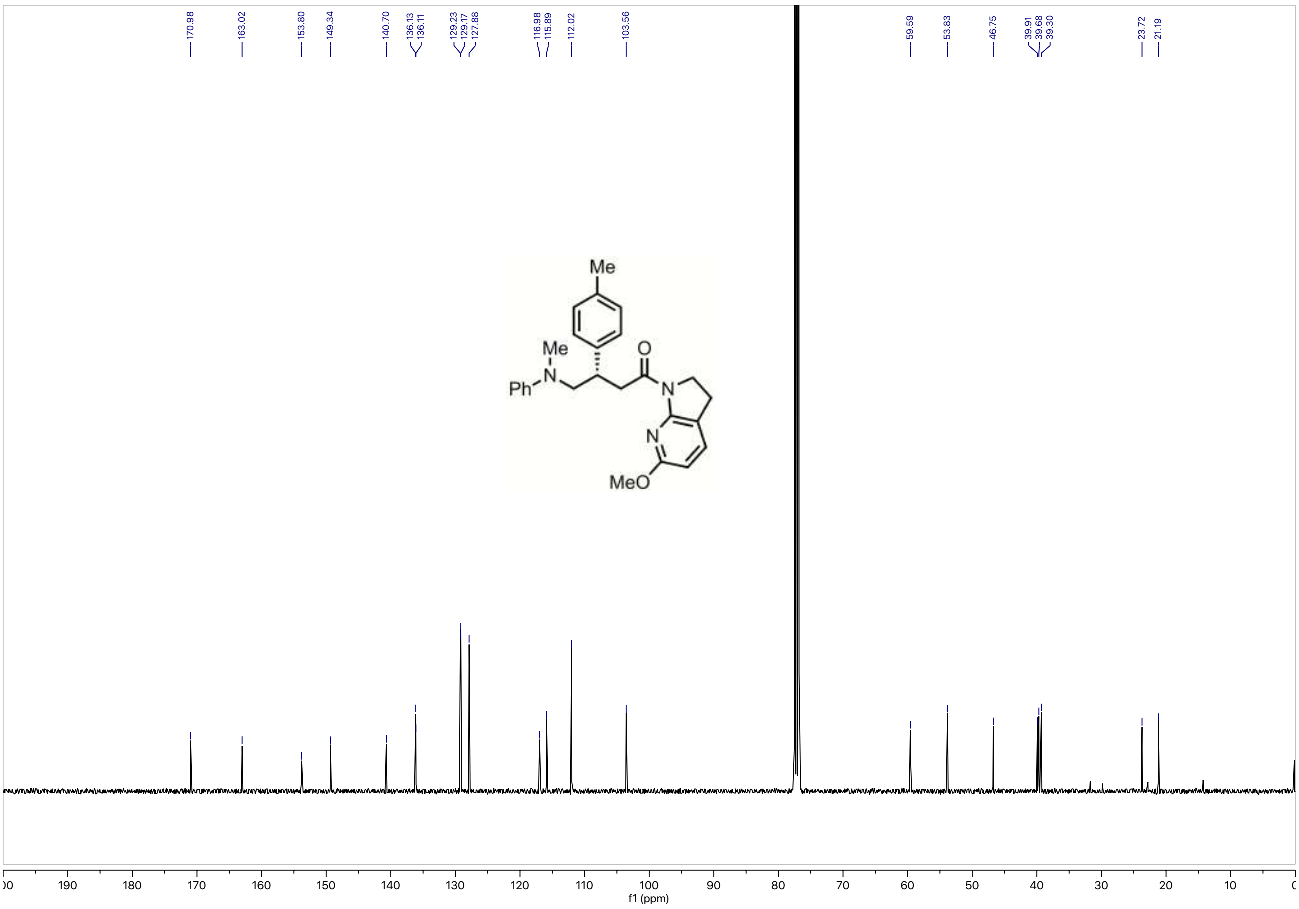

^{1}H NMR: **3ta**

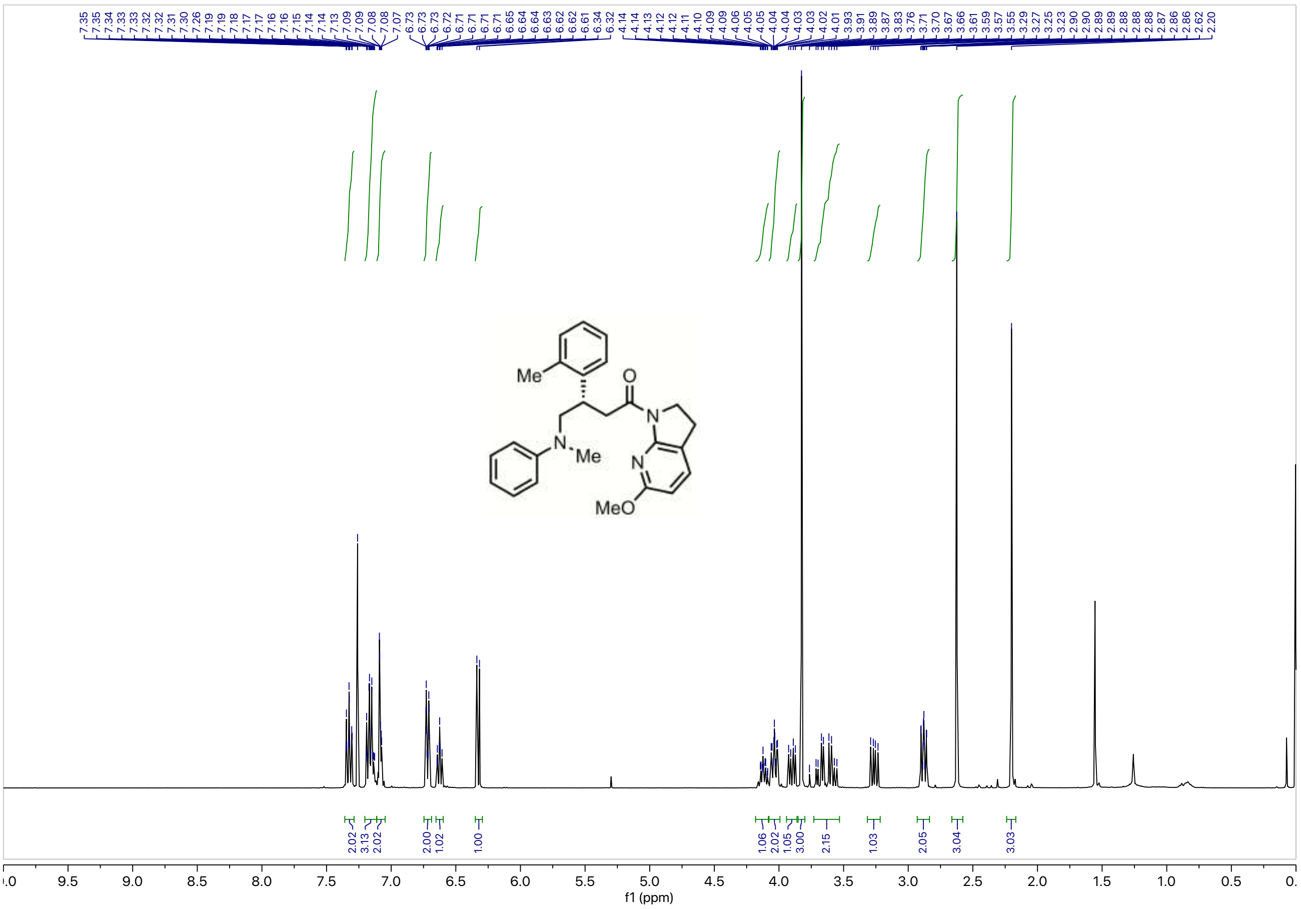

^{13}C NMR: **3ta**

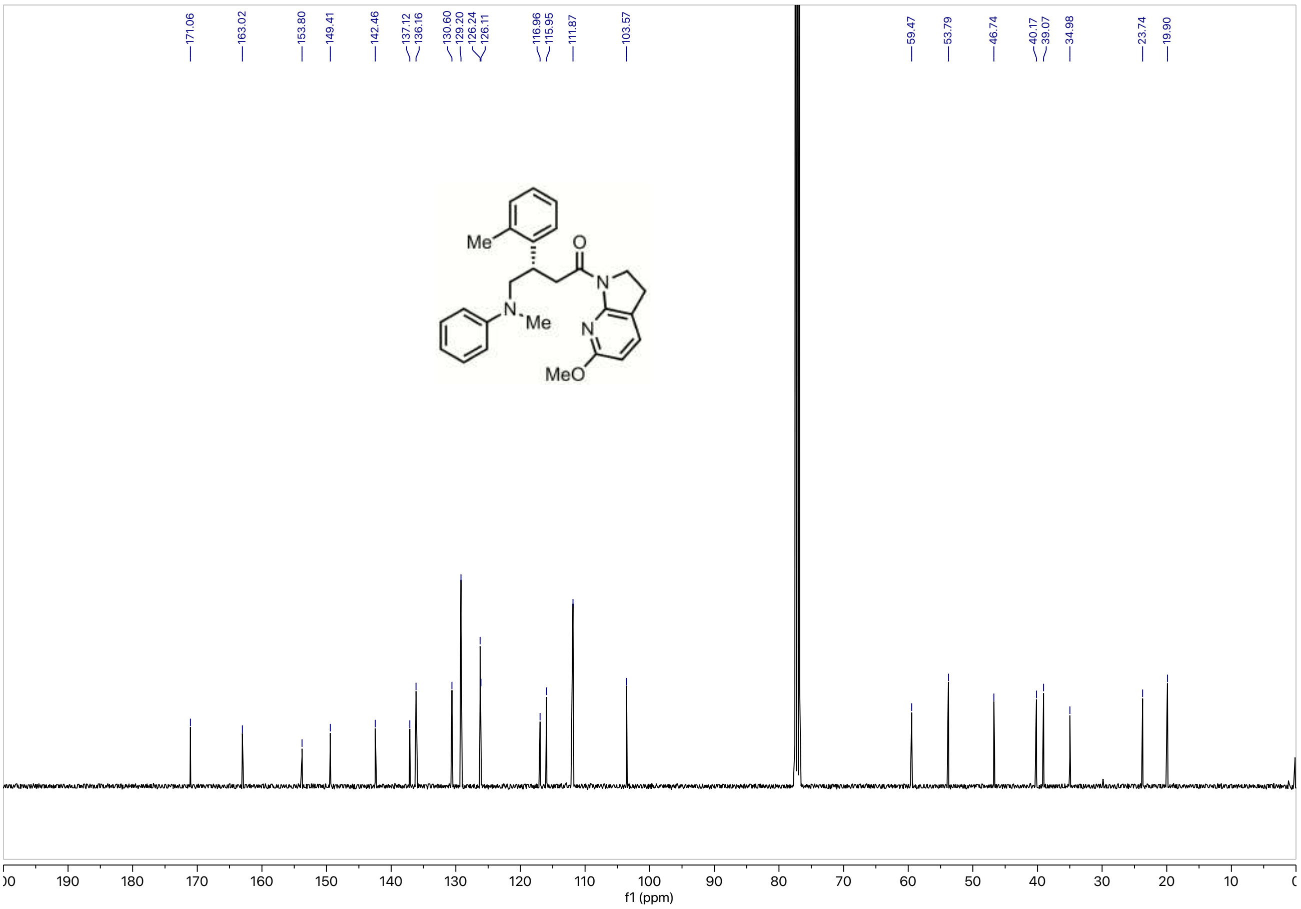

^{1}H NMR: **3ua**

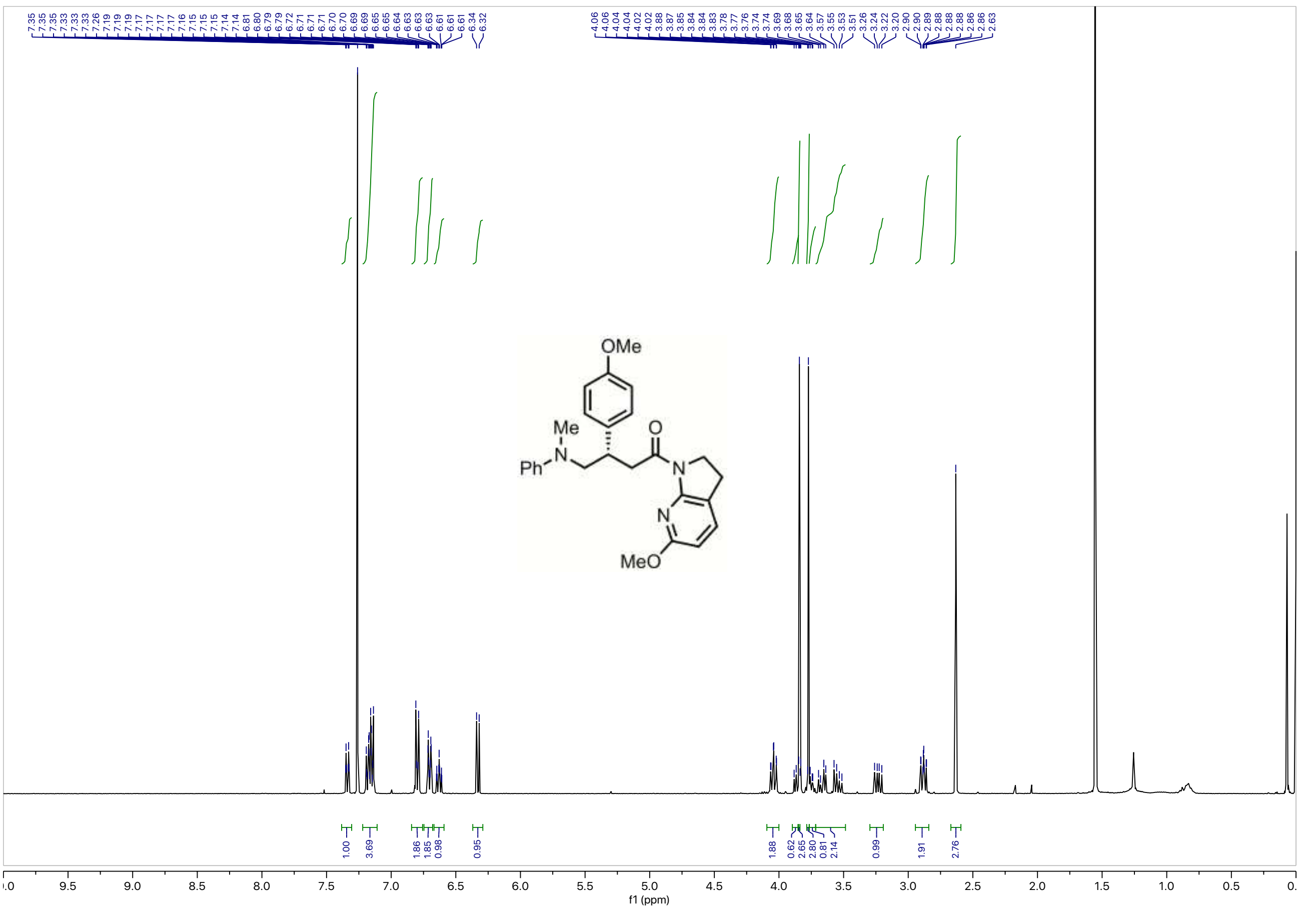

^{13}C NMR: **3ua**

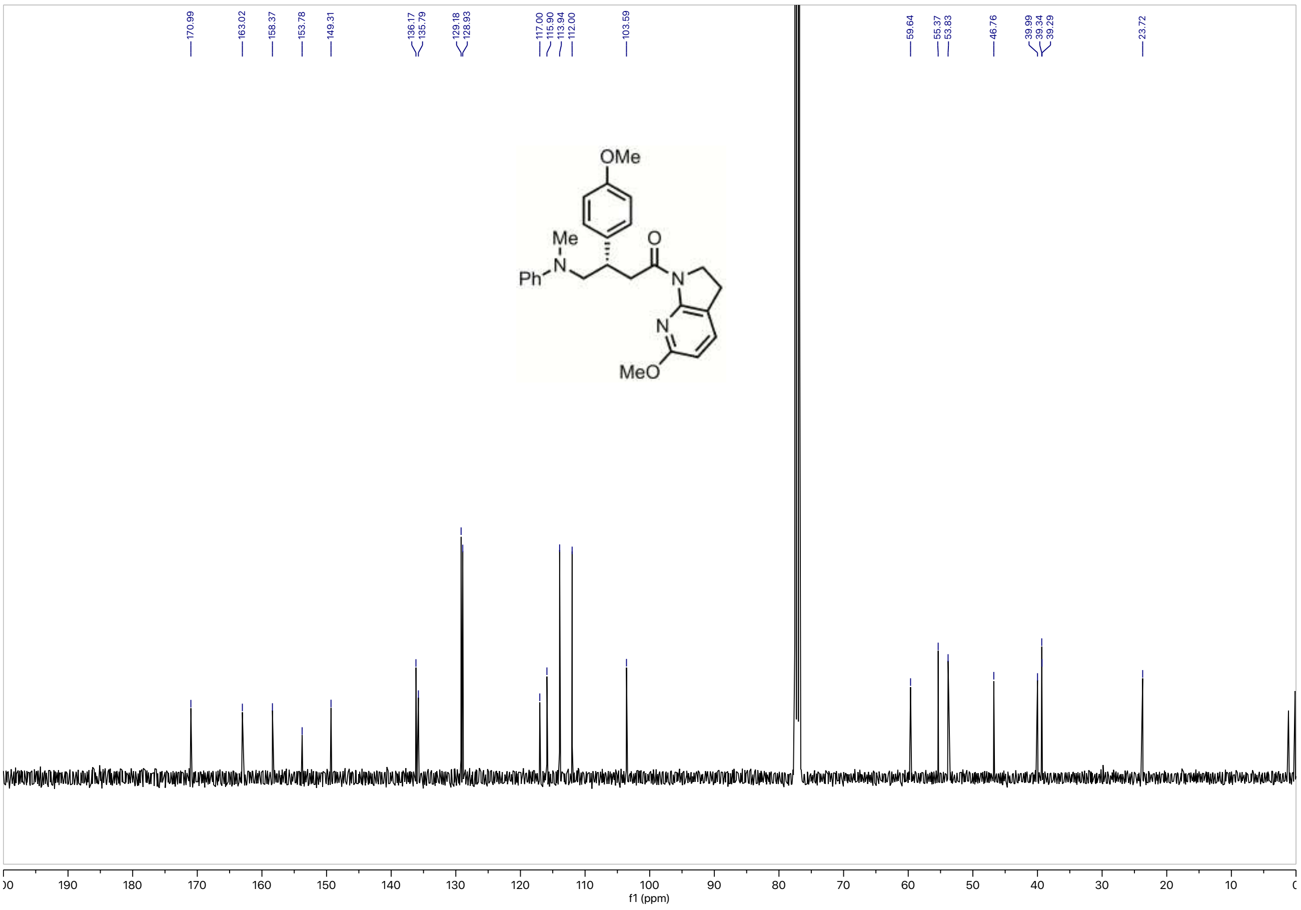

^{1}H NMR: **3va**

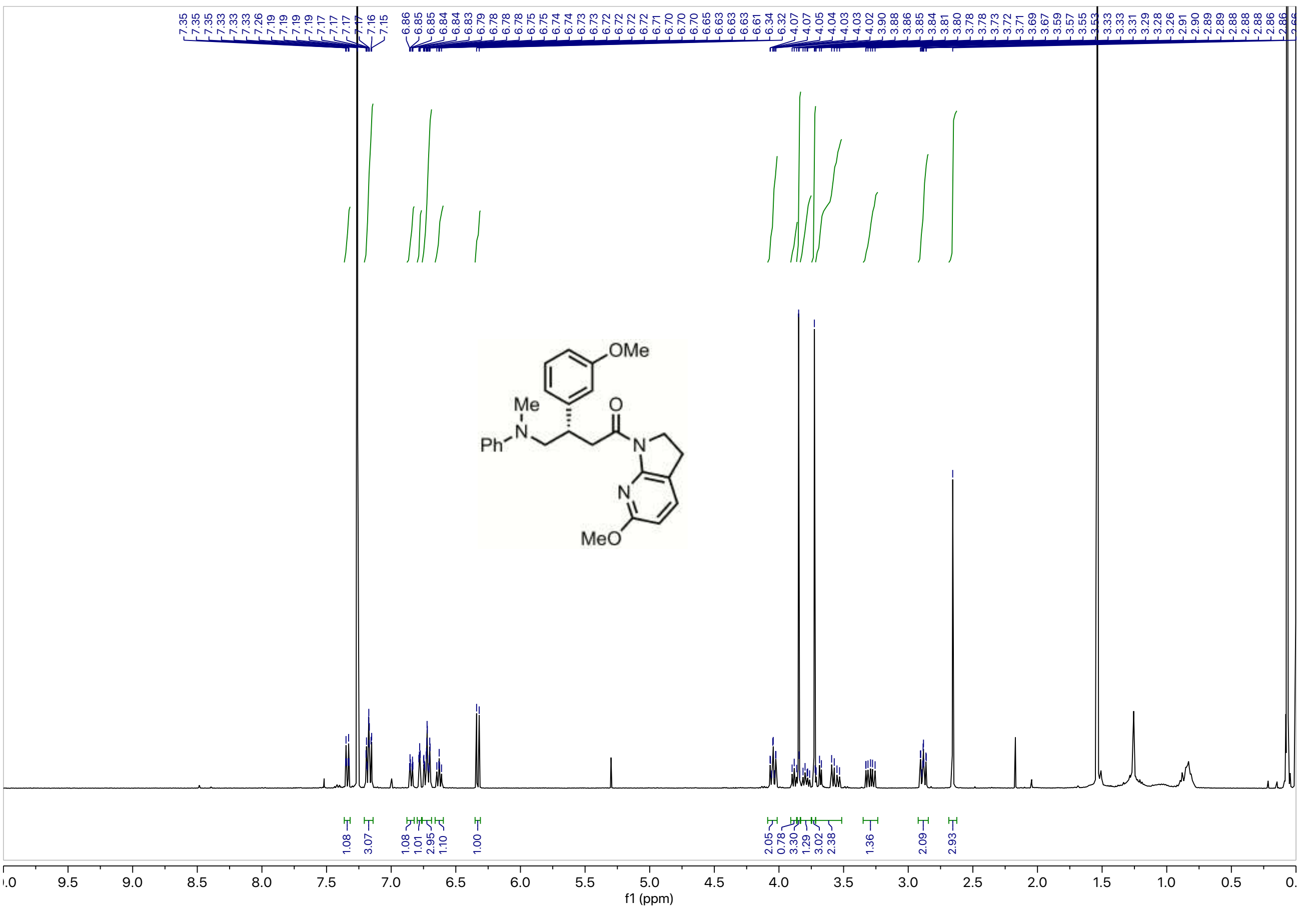

^{13}C NMR: **3va**

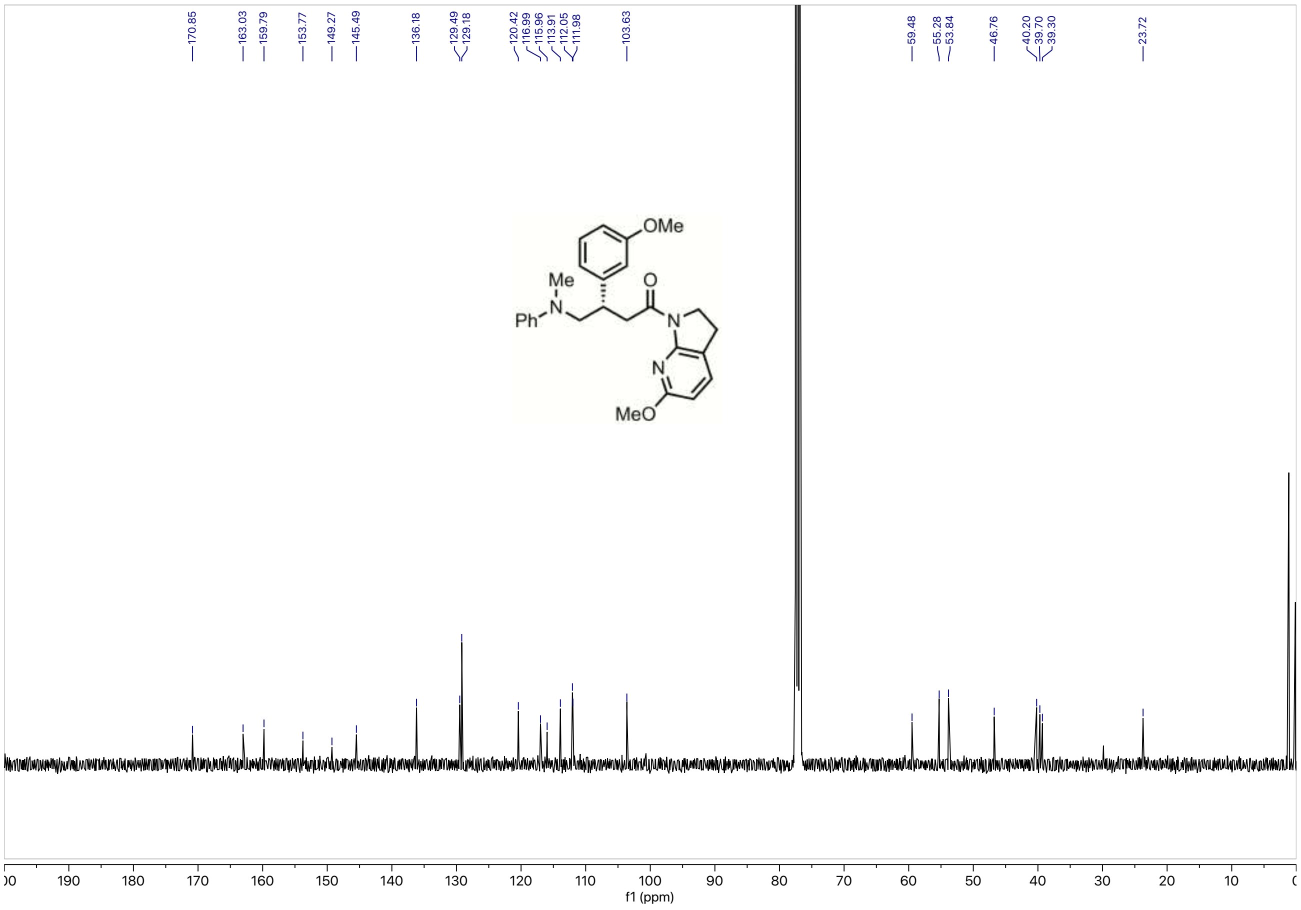

^{1}H NMR: **3wa**

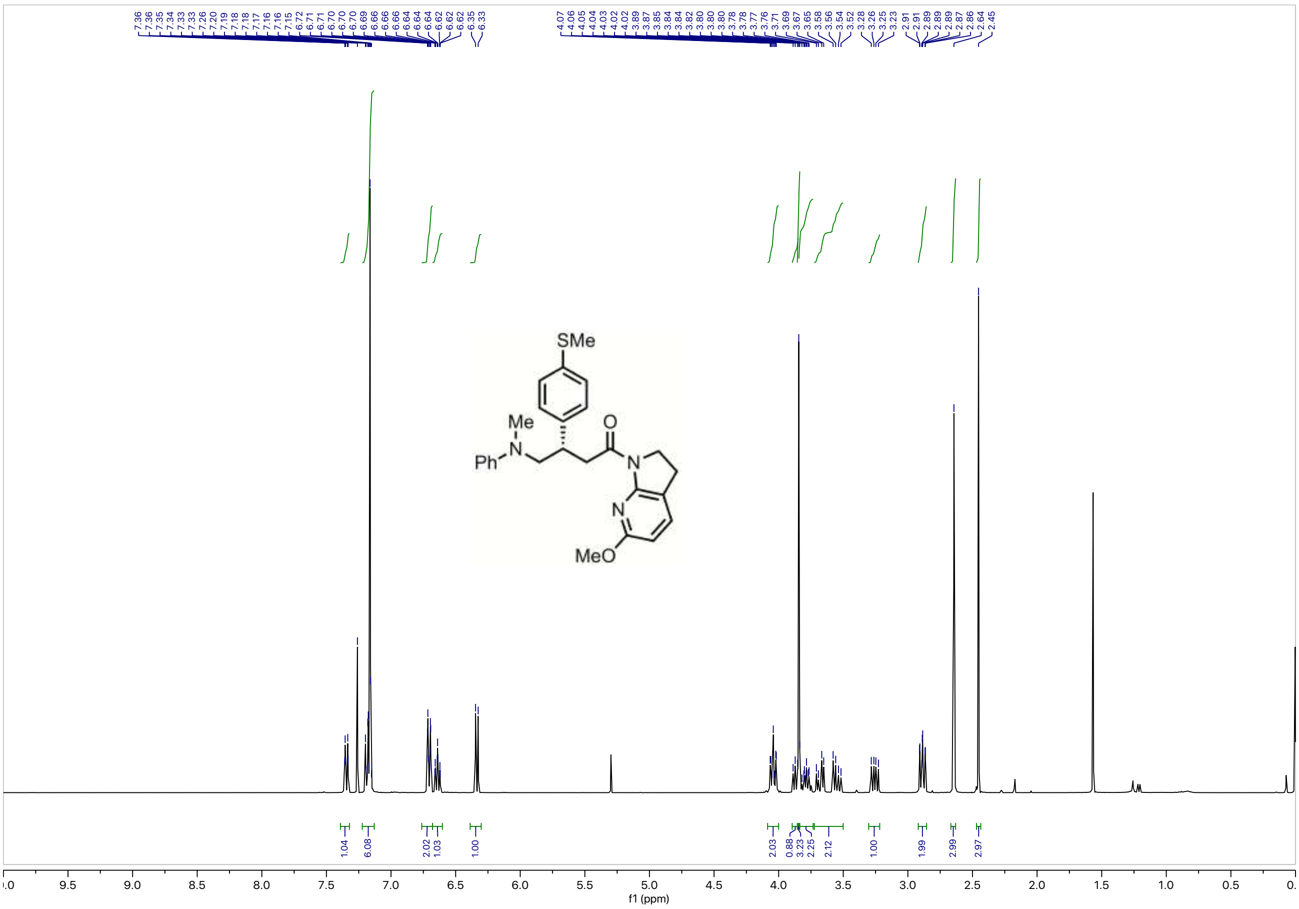

^{13}C NMR: **3wa**

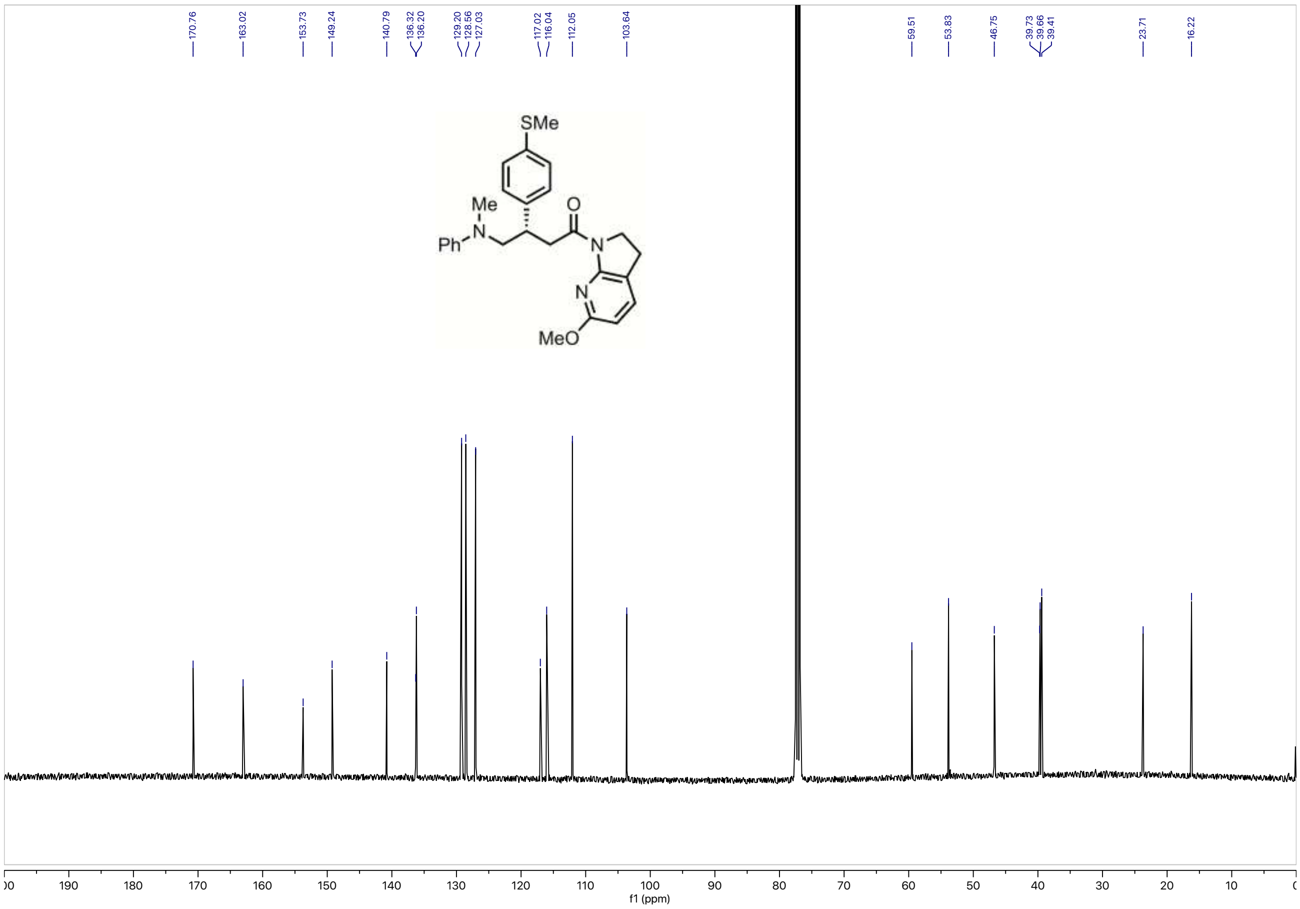

^{1}H NMR: **3xa**

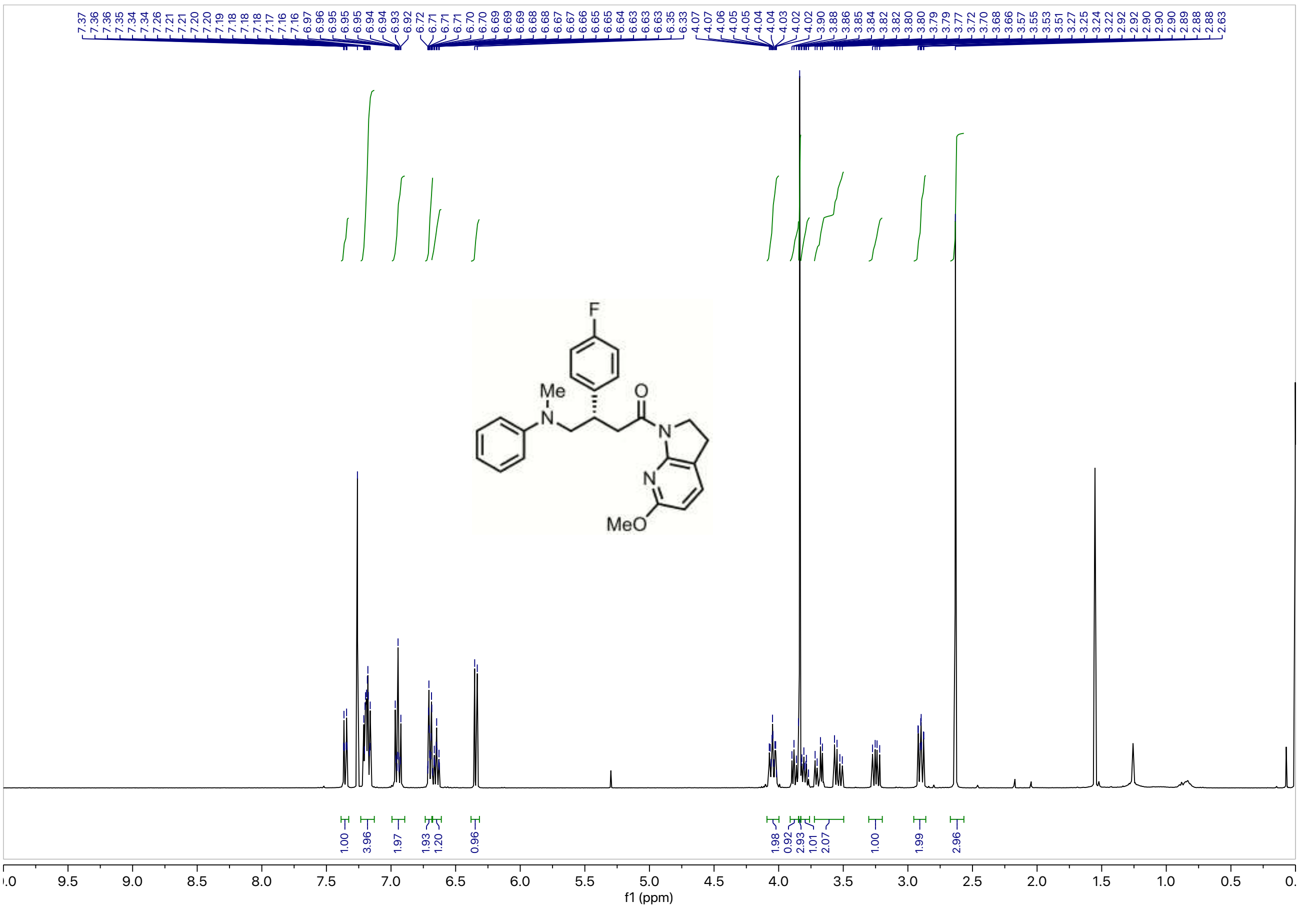

^{13}C NMR: **3xa**

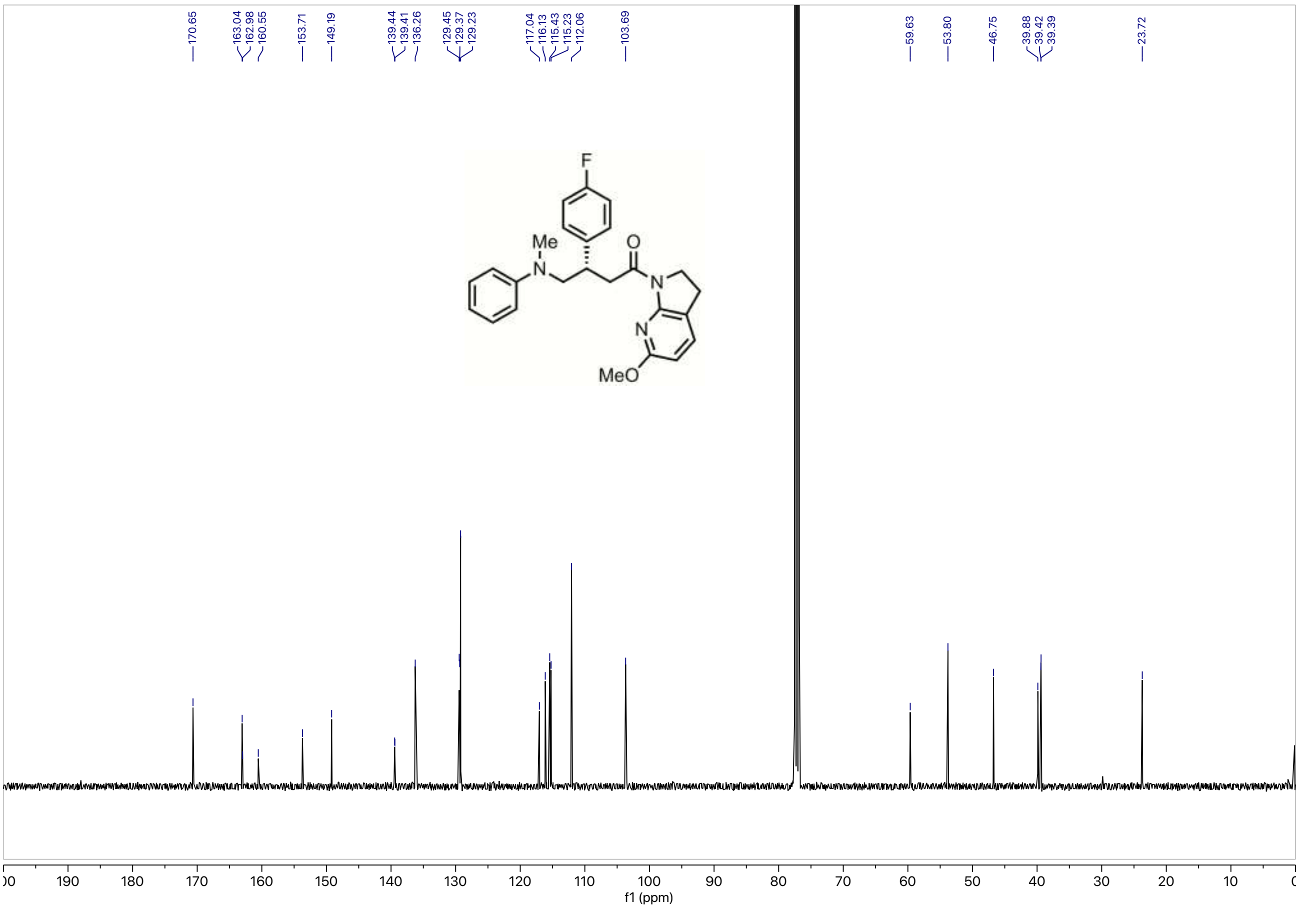

^{19}F NMR: **3xa**

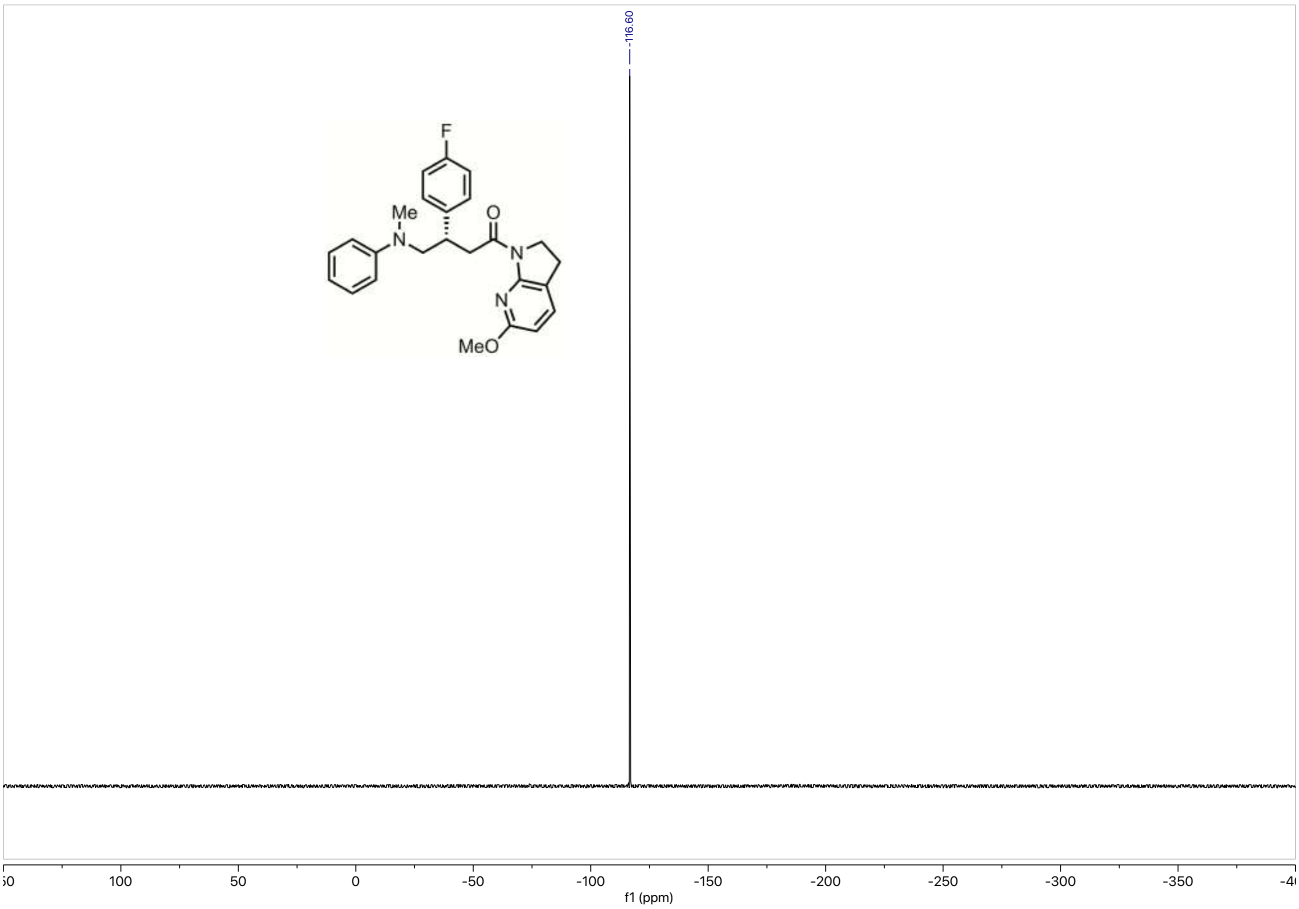

^{1}H NMR: **3ya**

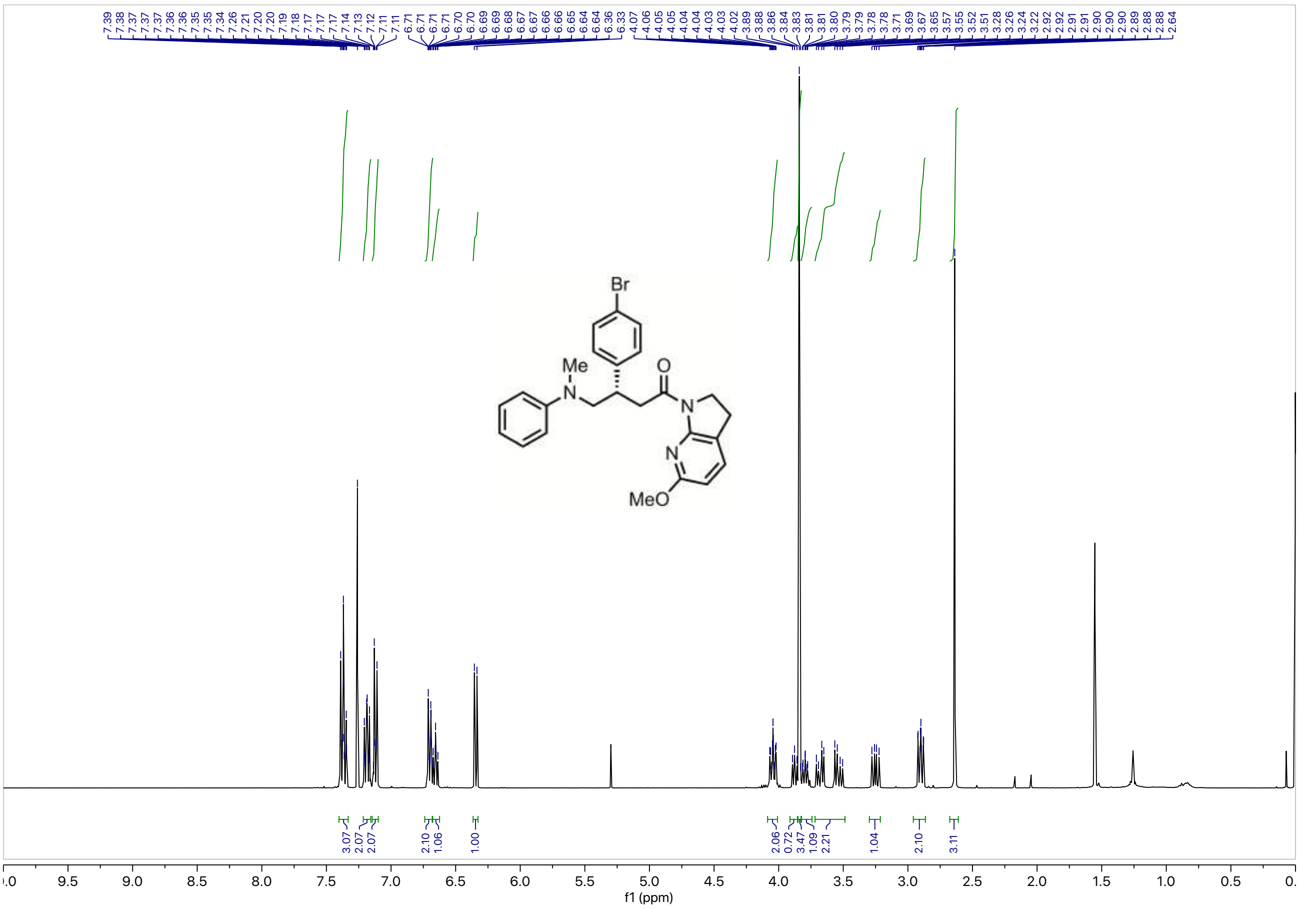

^{13}C NMR: **3ya**

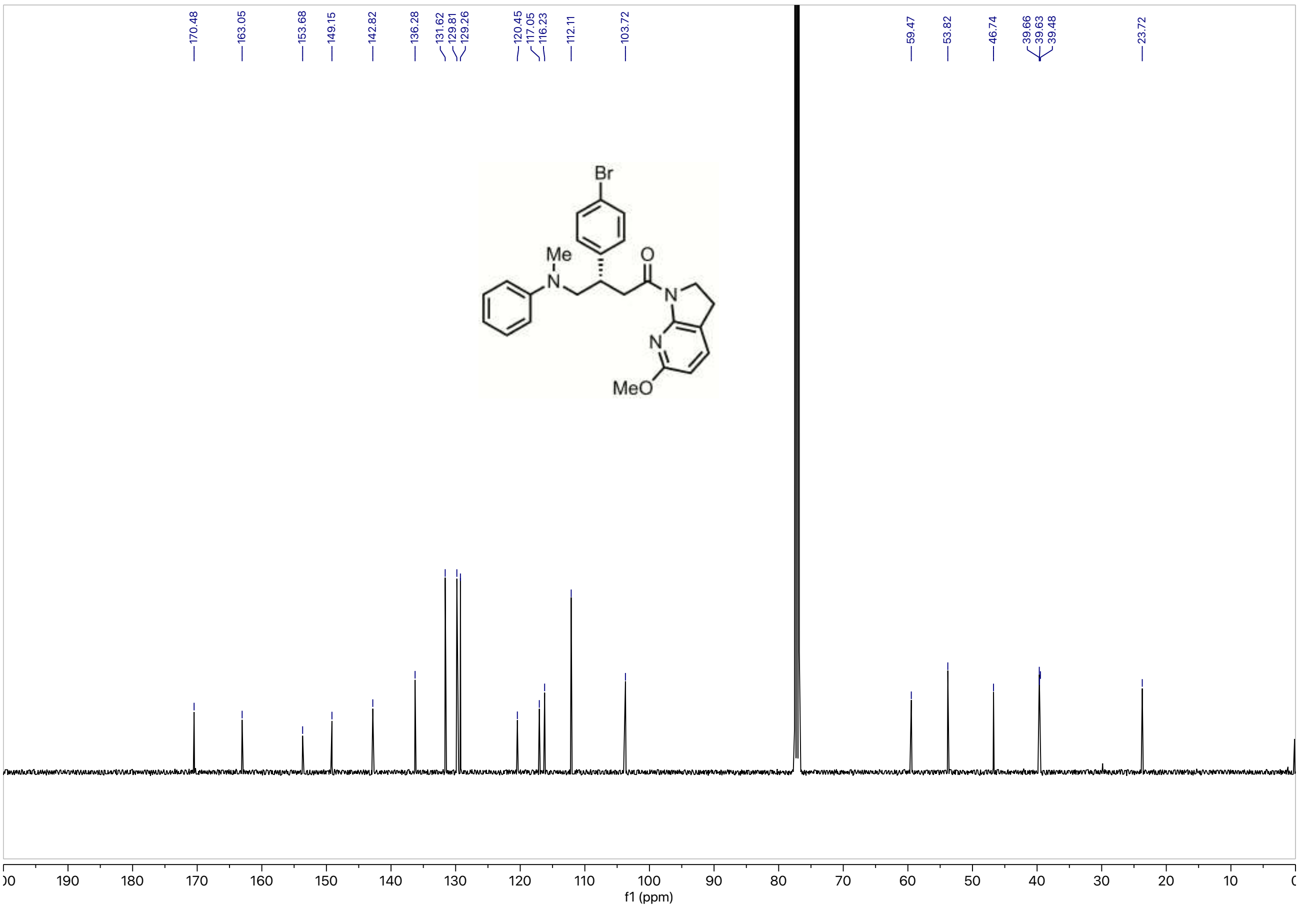

^{1}H NMR: **3za**

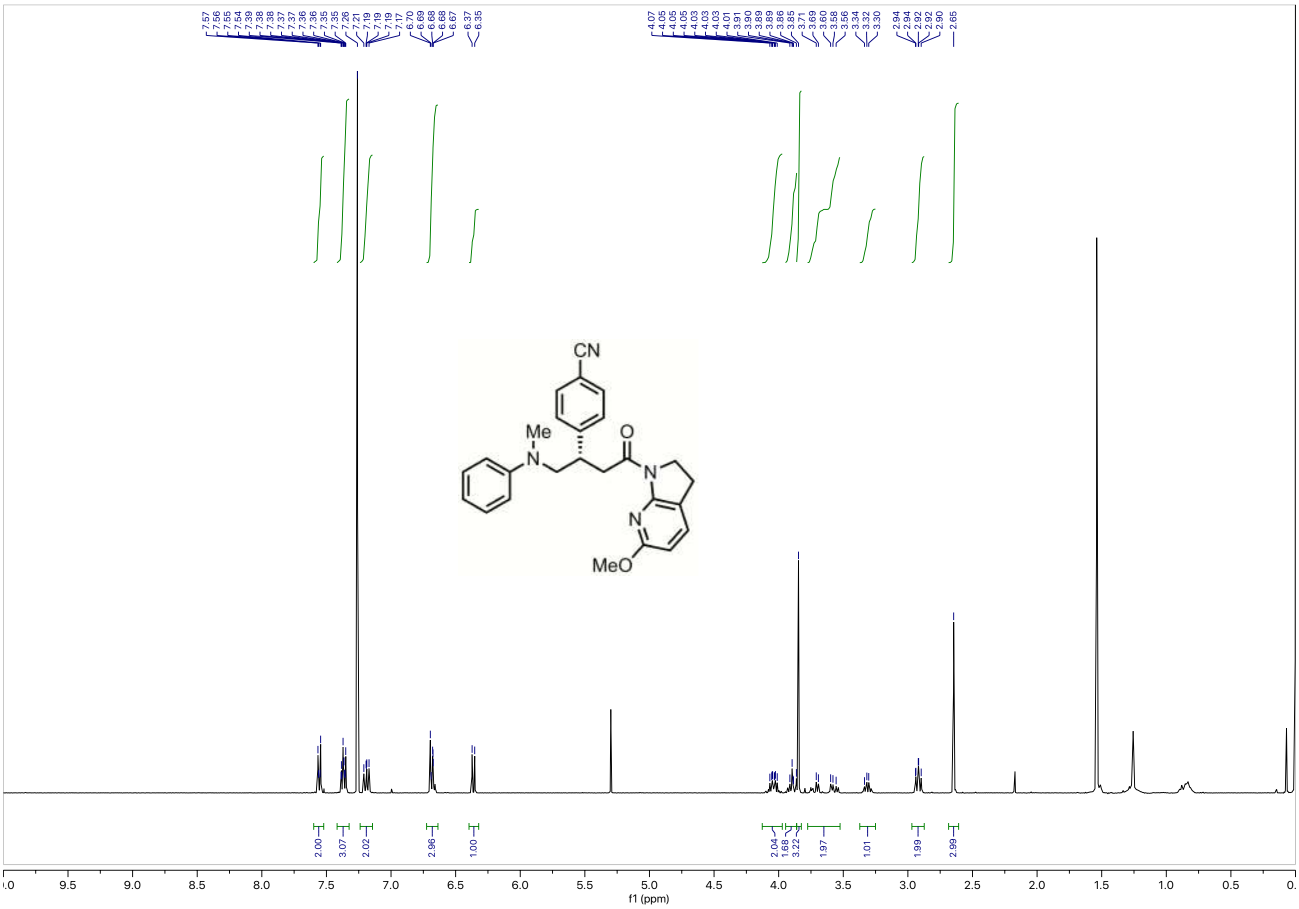

^{13}C NMR: **3za**

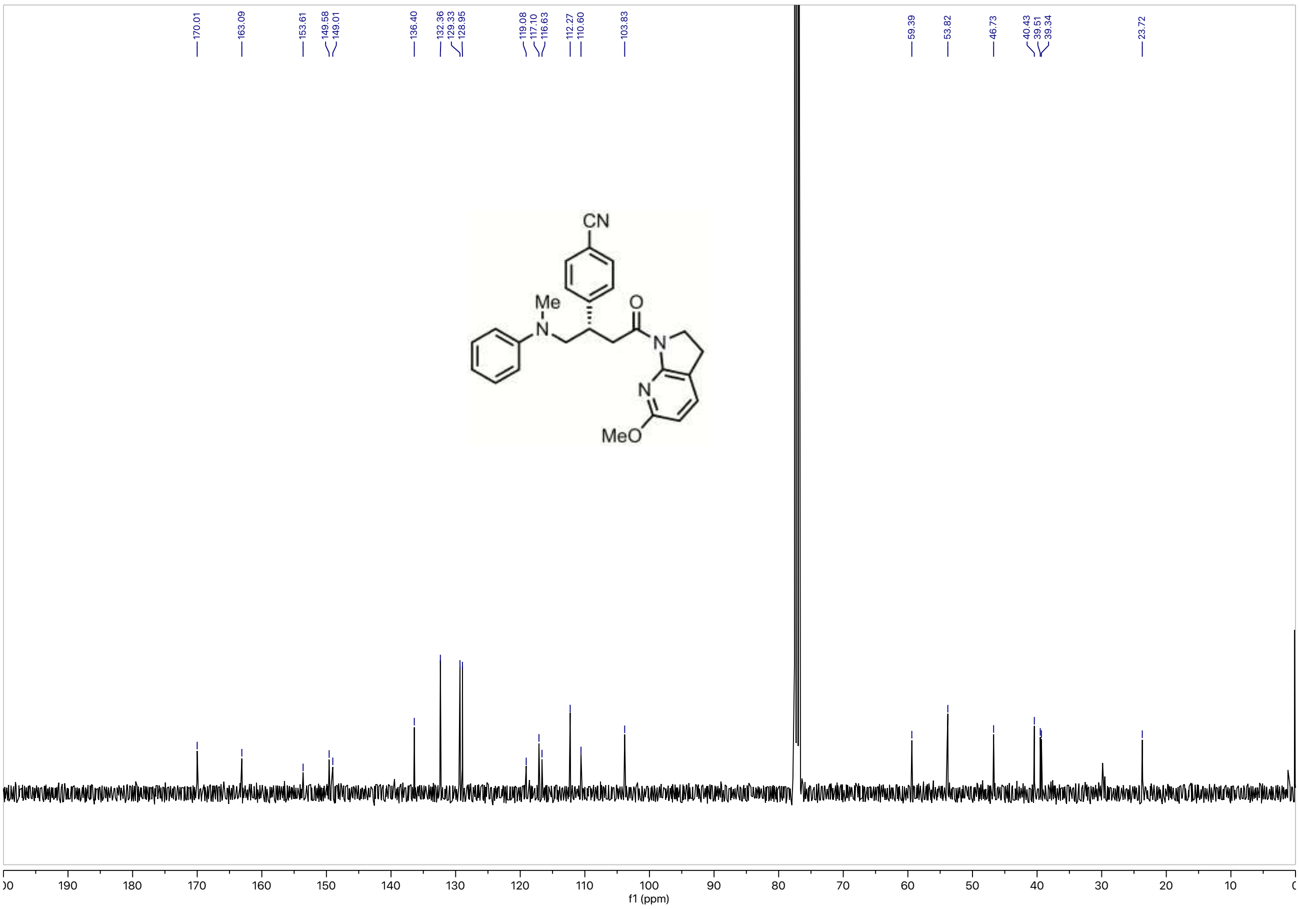

^{1}H NMR: **3aaa**

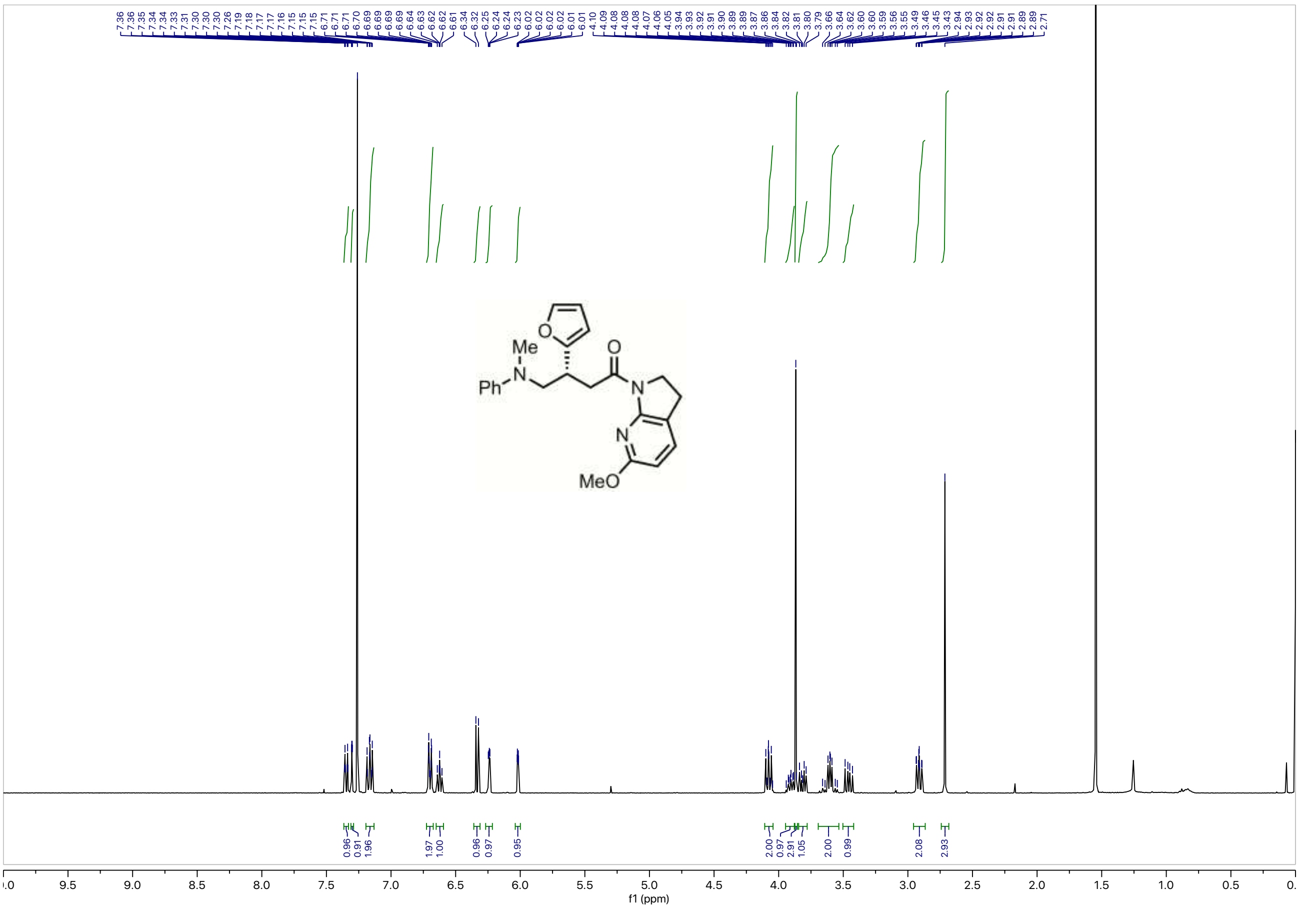

^{13}C NMR: **3aaa**

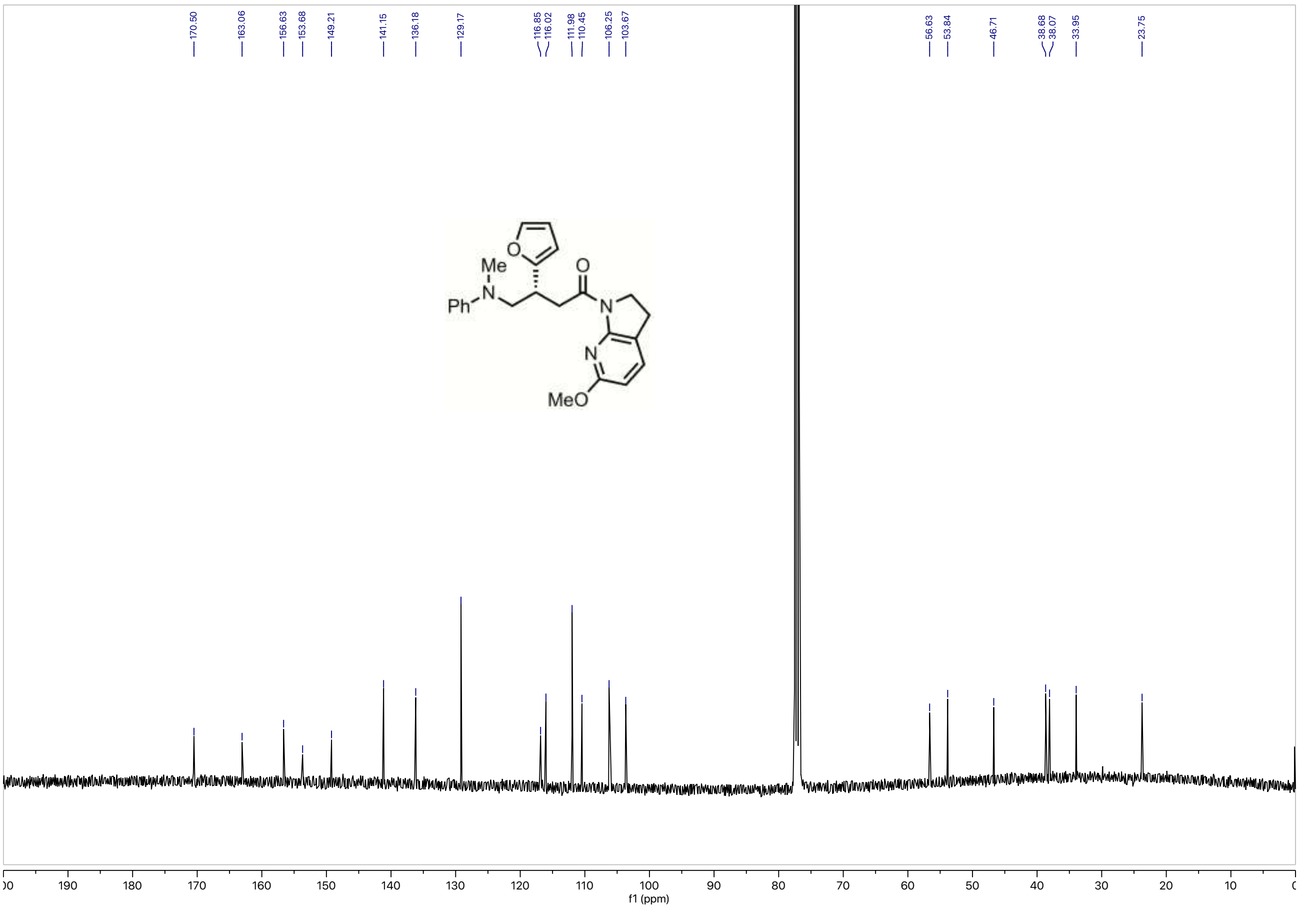

^{1}H NMR: **3aba**

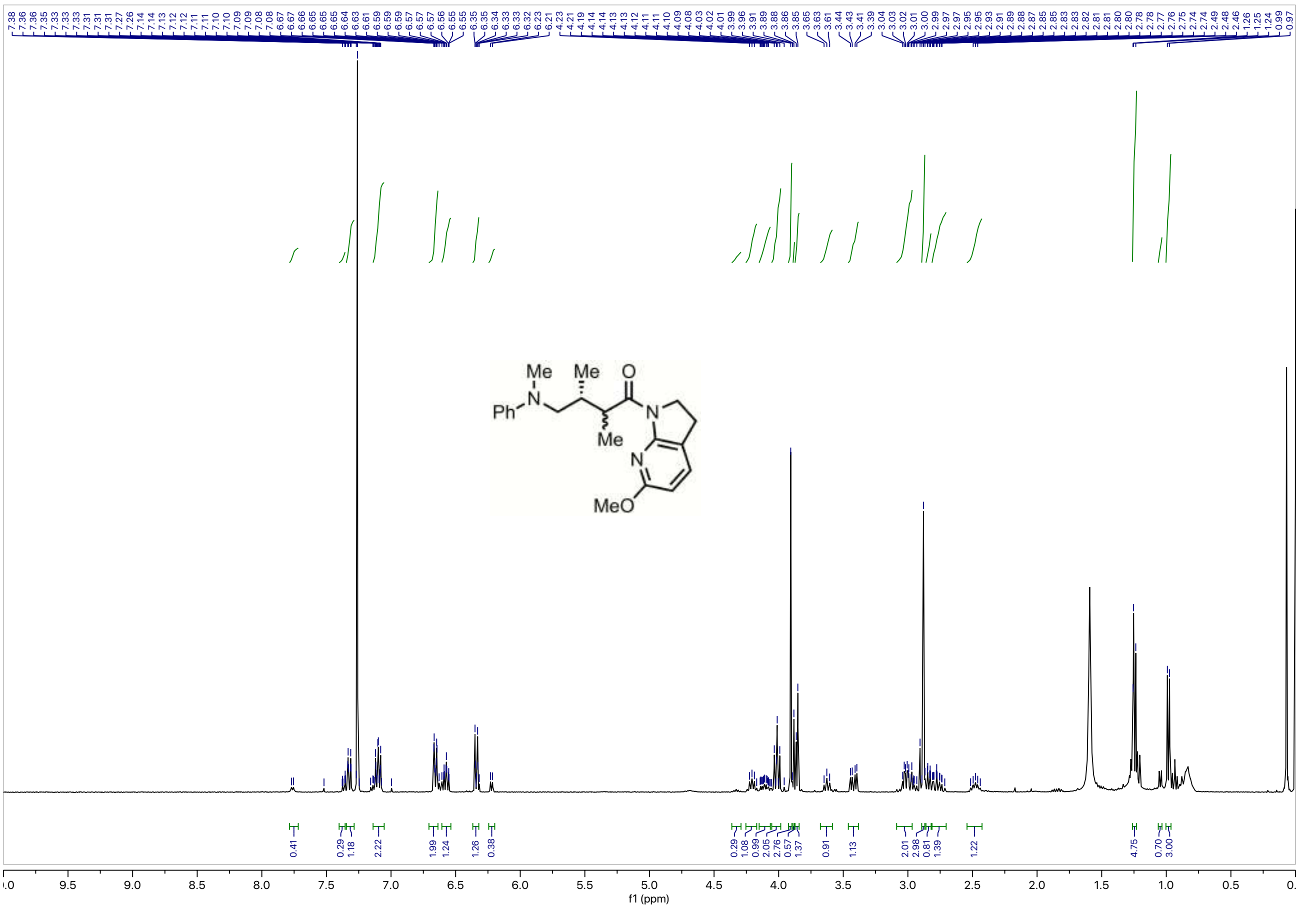

^{13}C NMR: **3aba**

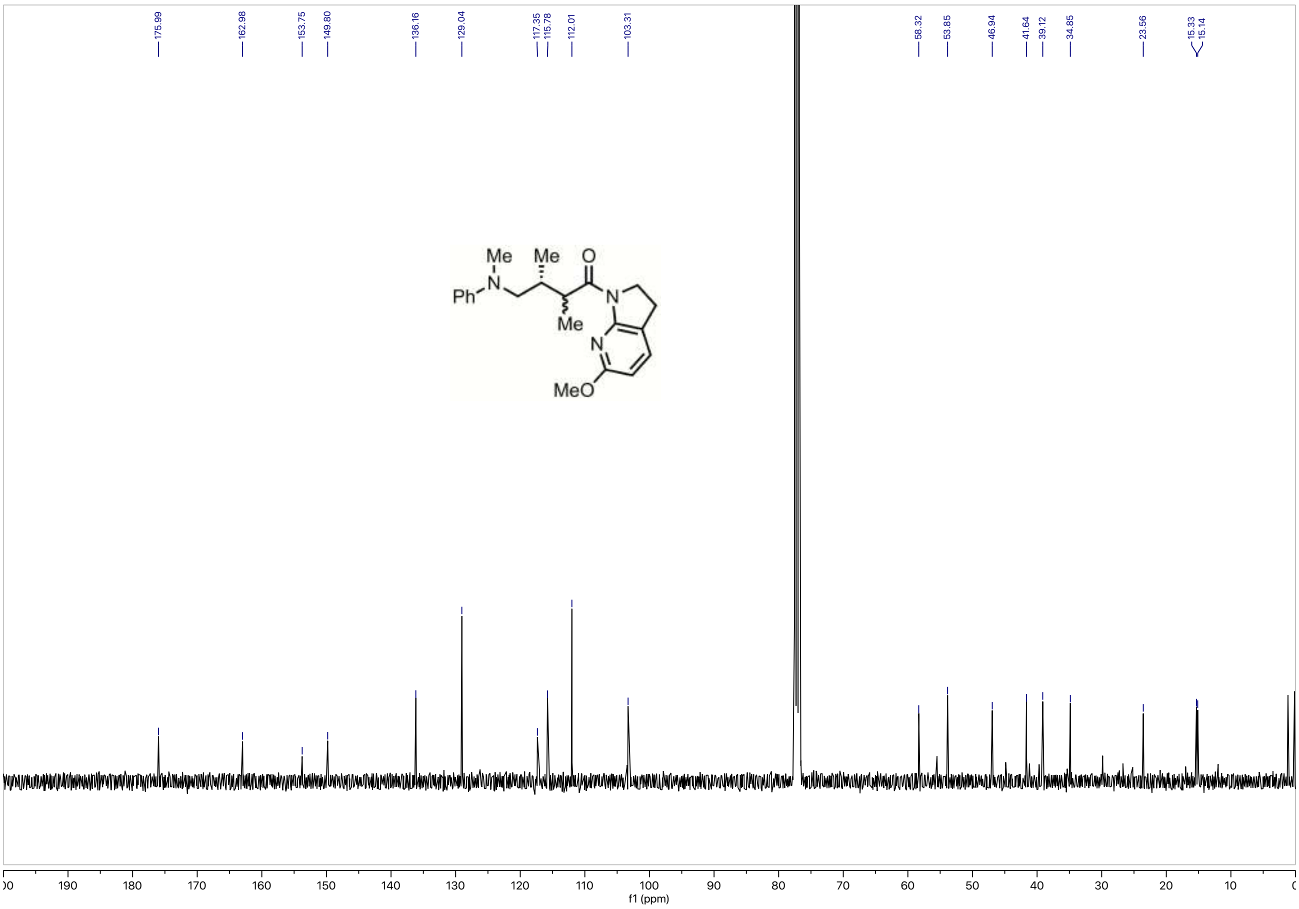

^{1}H NMR: **3db**

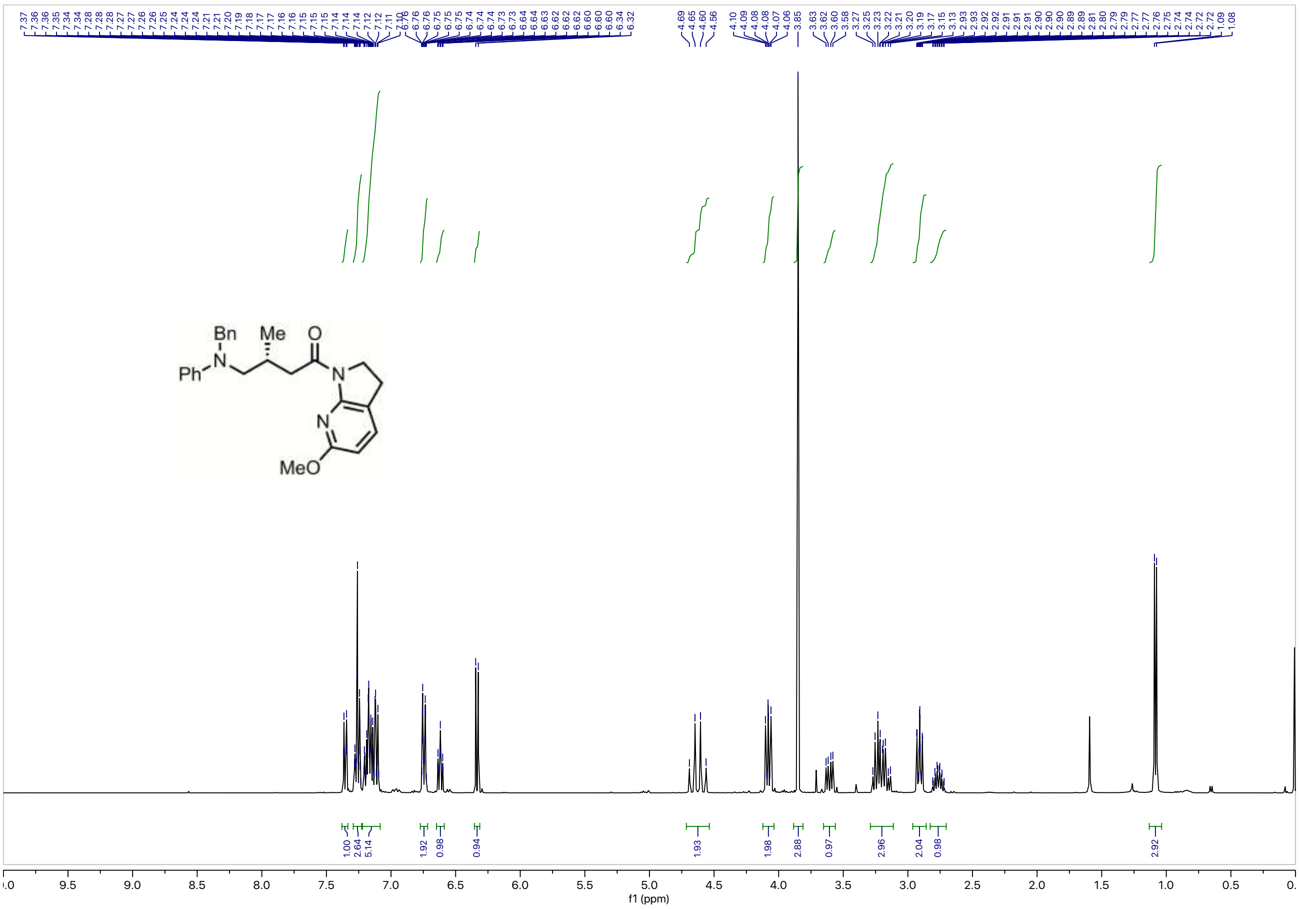

^{13}C NMR: **3db**

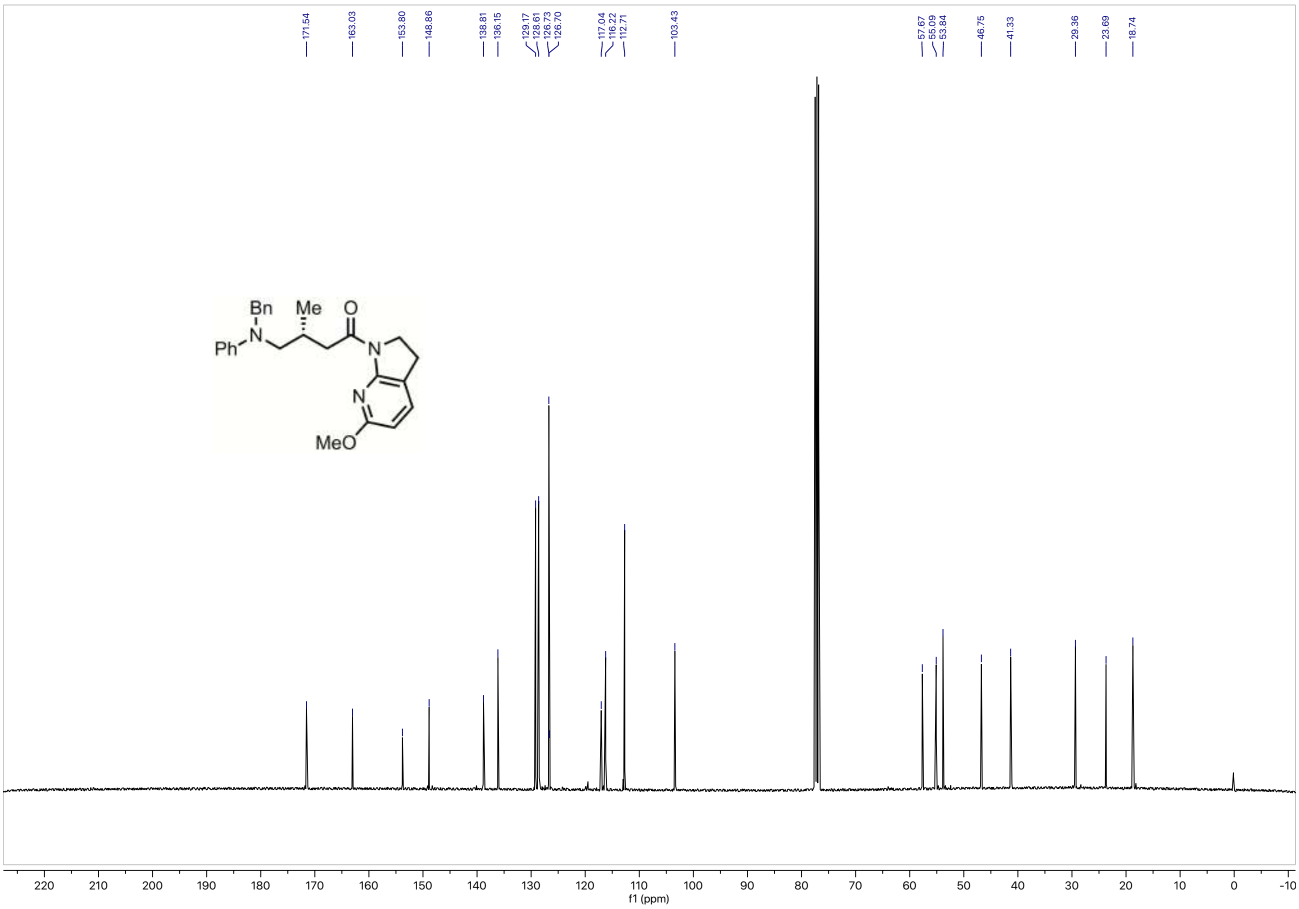

^{1}H NMR: **3dc**

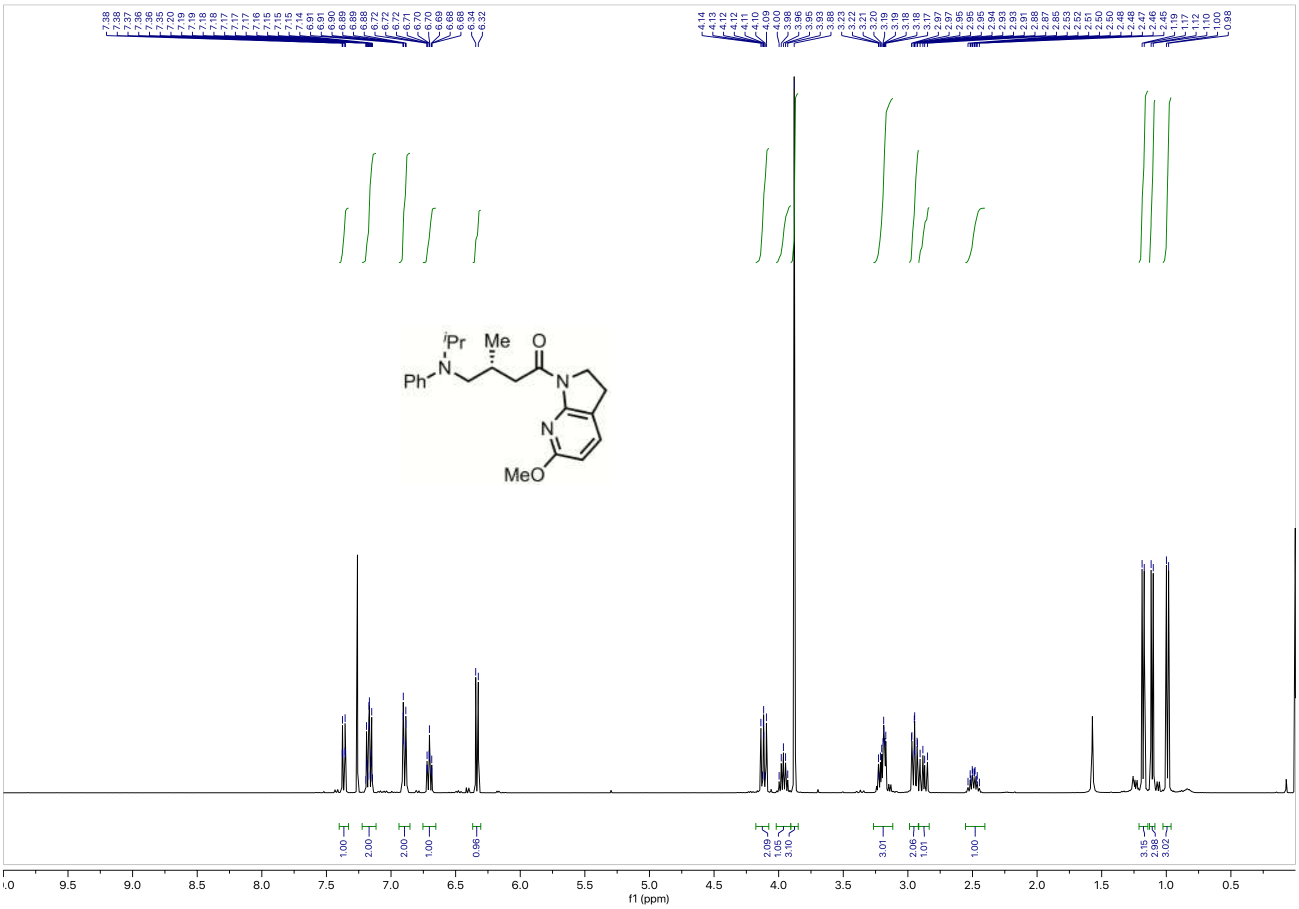

^{13}C NMR: **3dc**

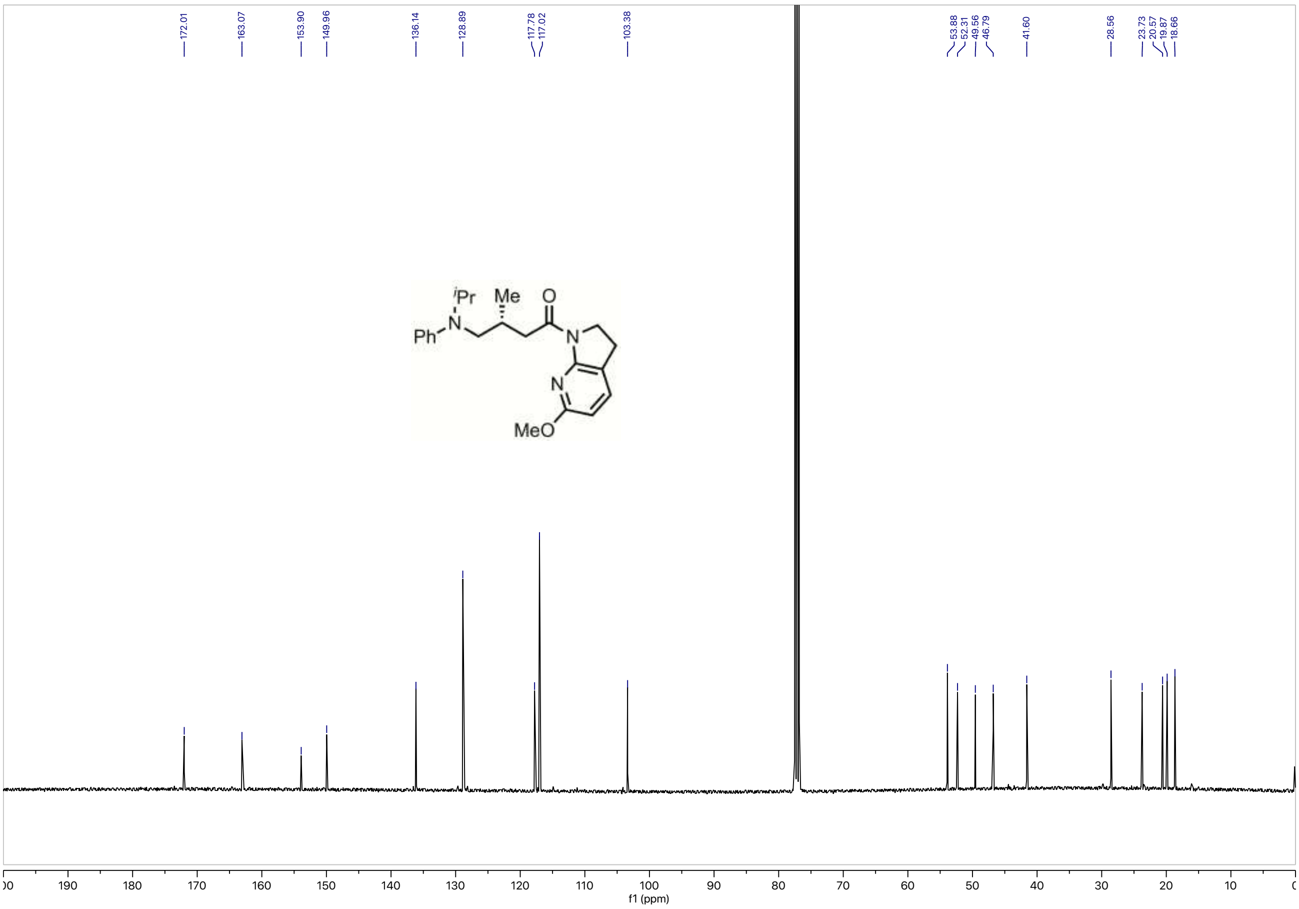

^{1}H NMR: **3dd**

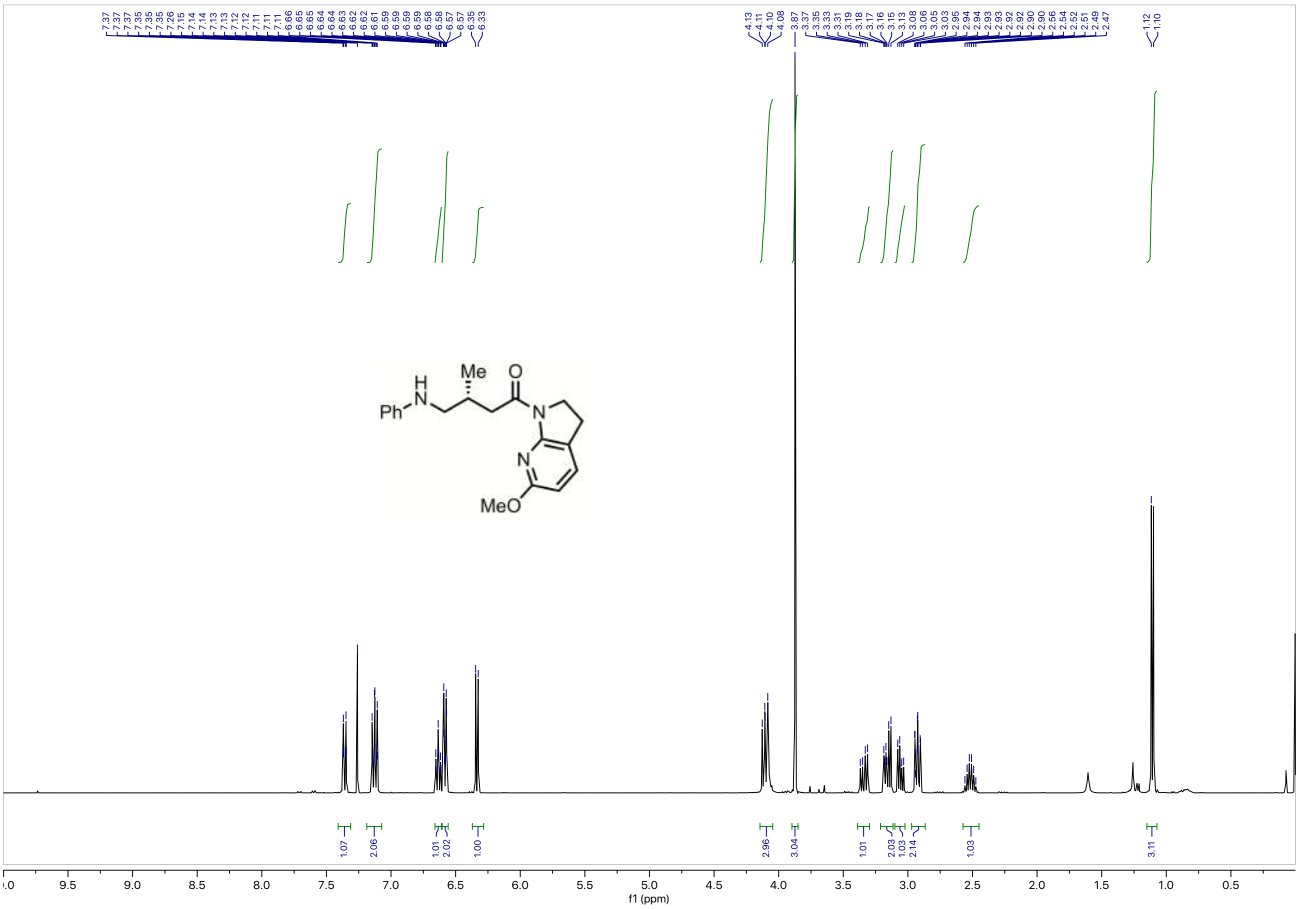

^{13}C NMR: **3dd**

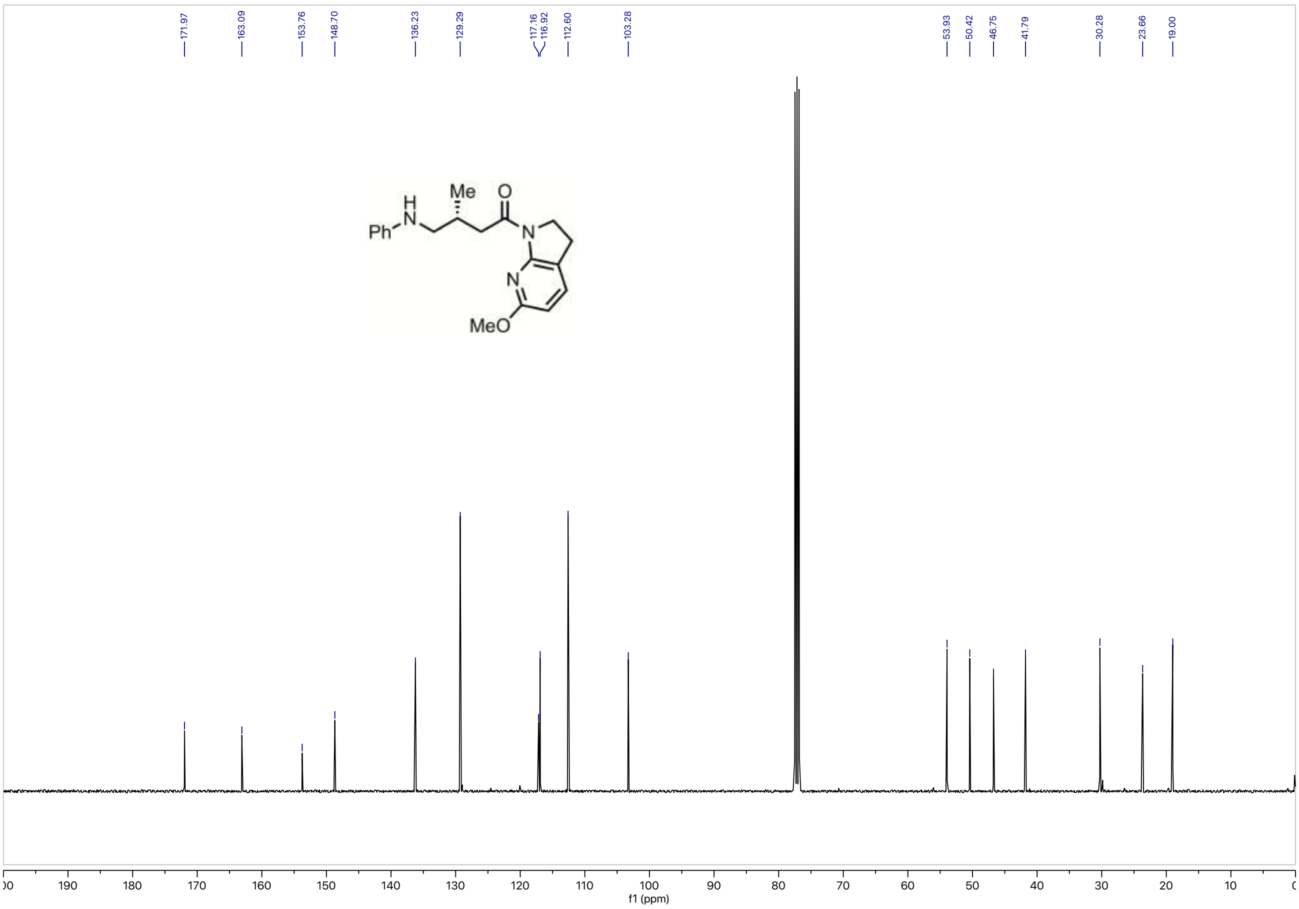

^{1}H NMR: **3de**

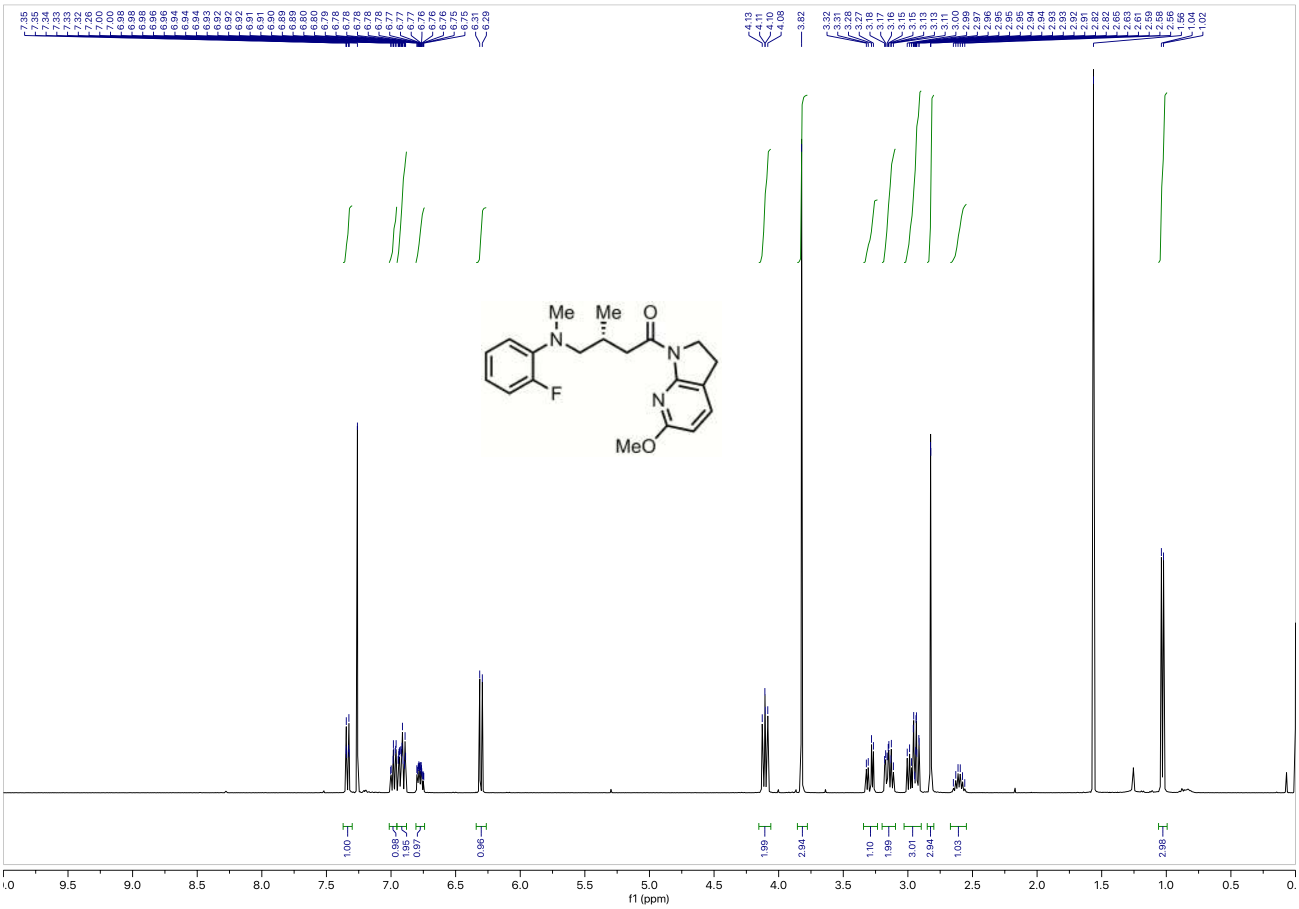

^{13}C NMR: **3de**

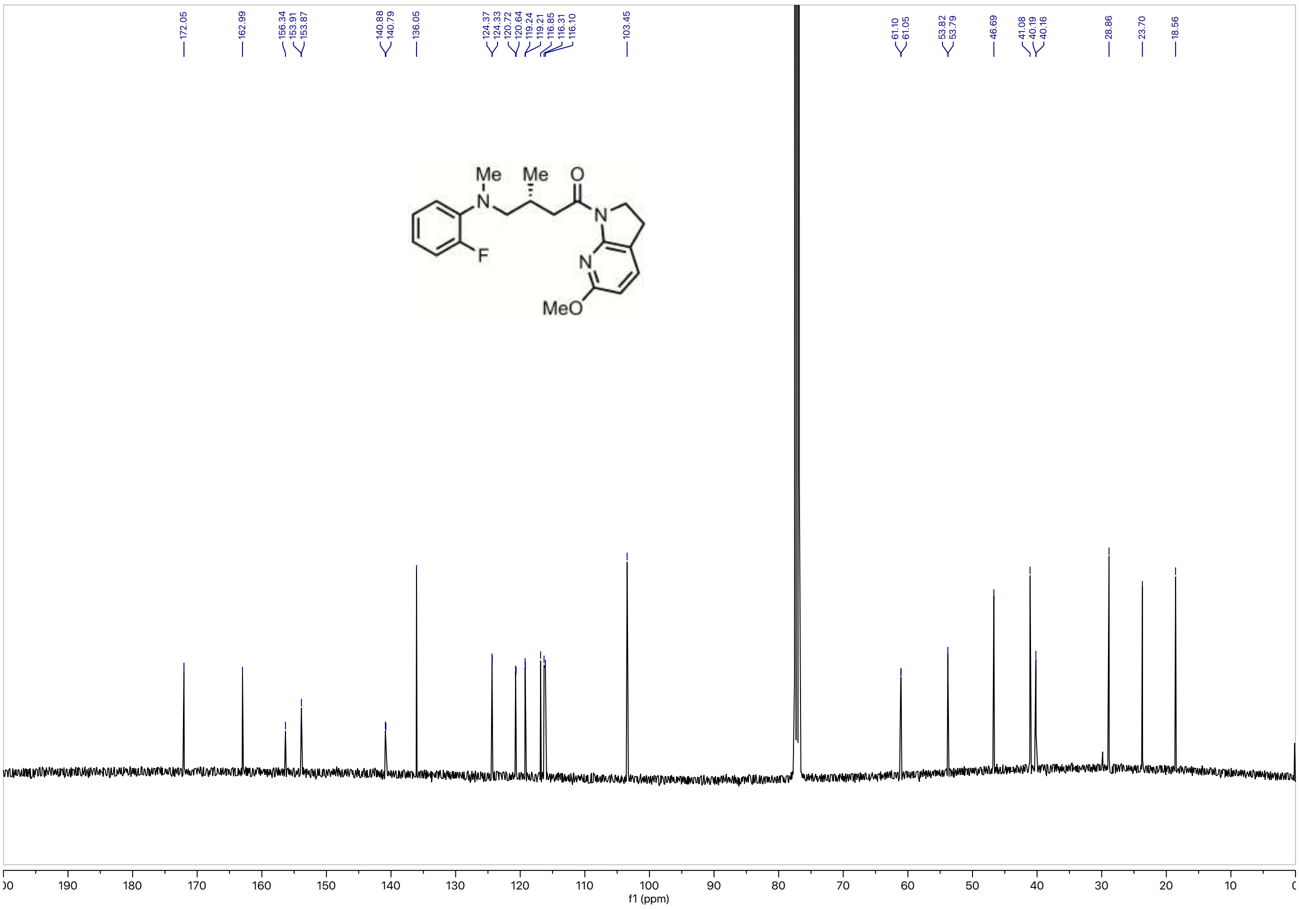

^{19}F NMR: **3de**

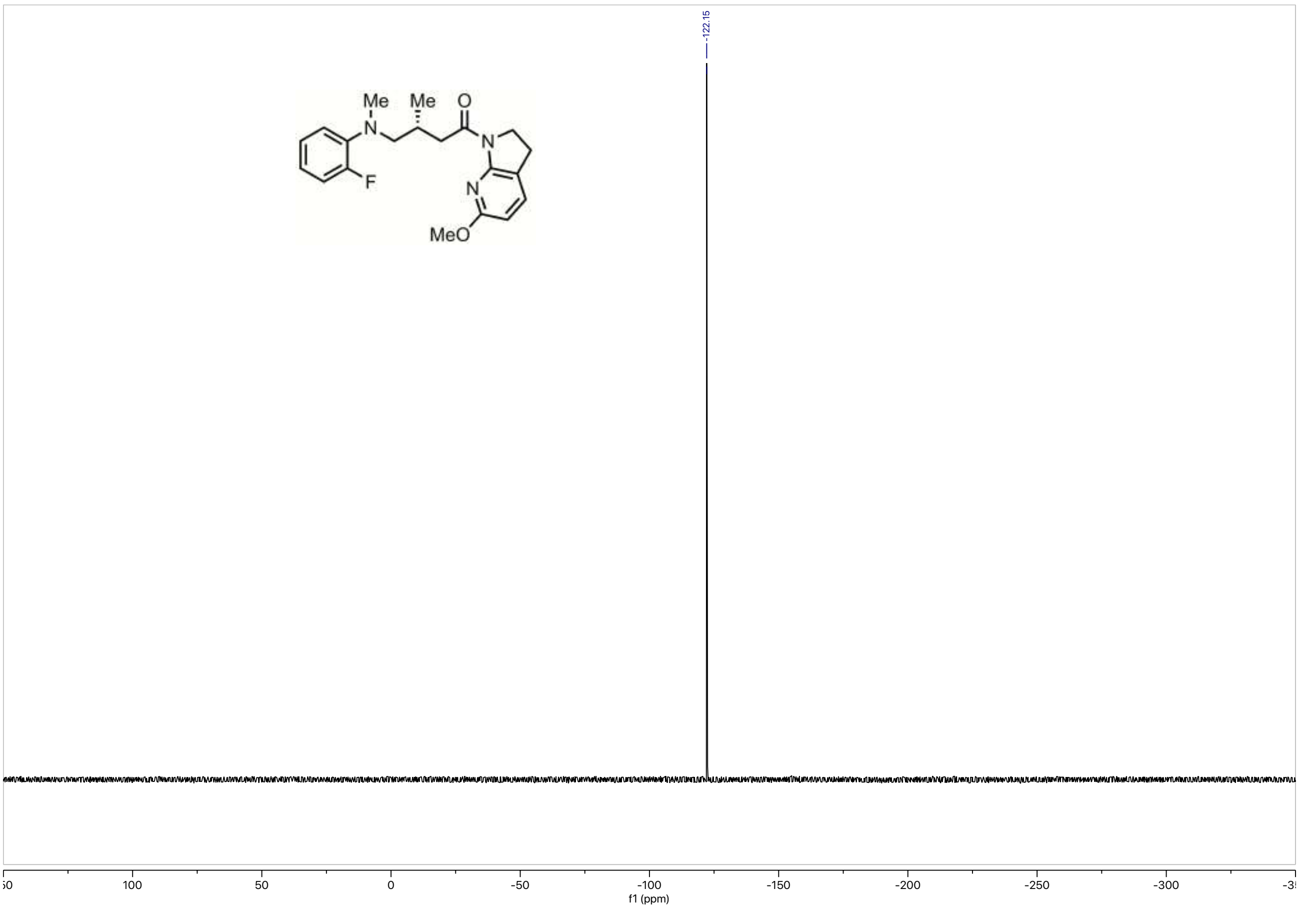

^{1}H NMR: **11**

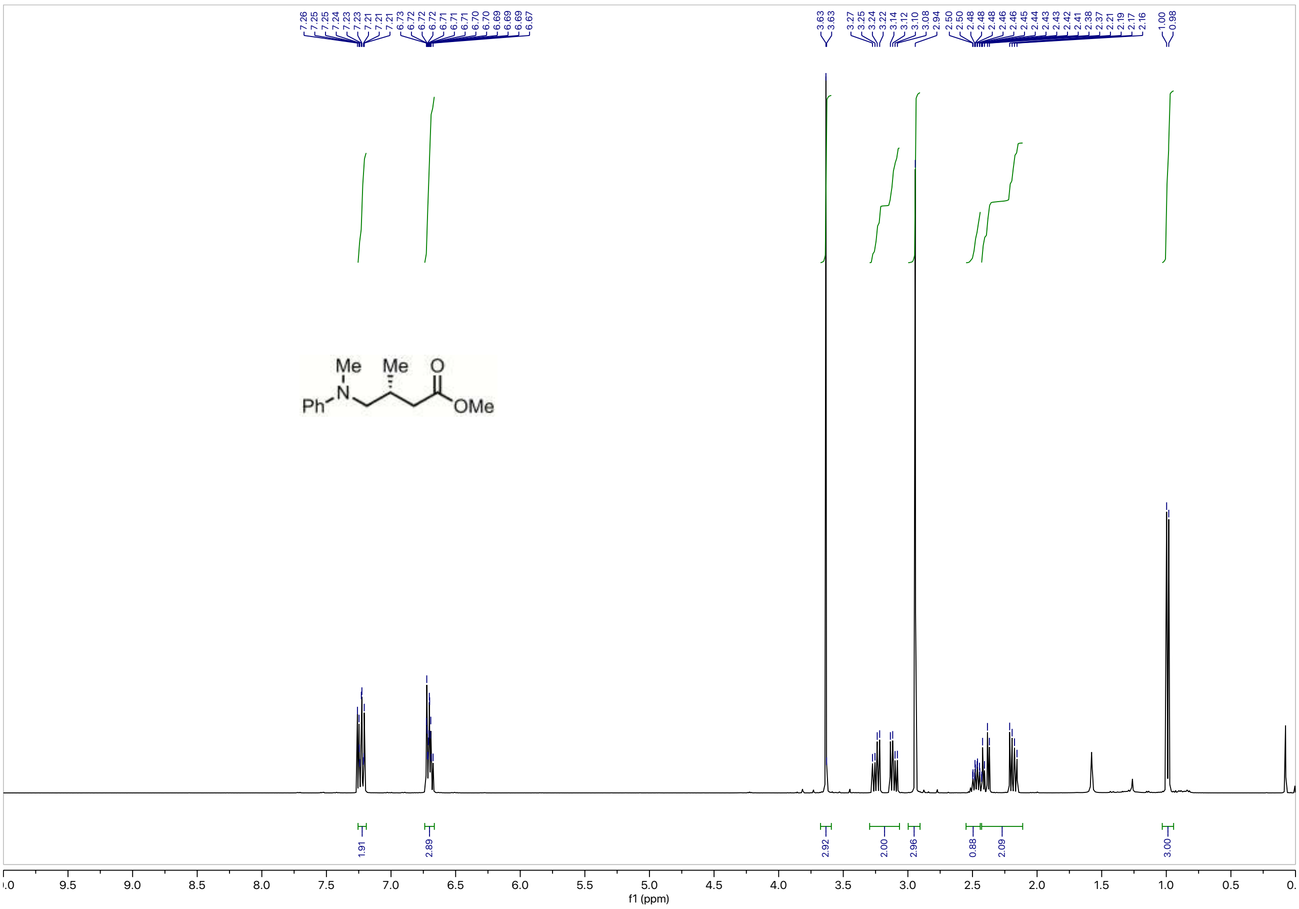

^{13}C NMR: **11**

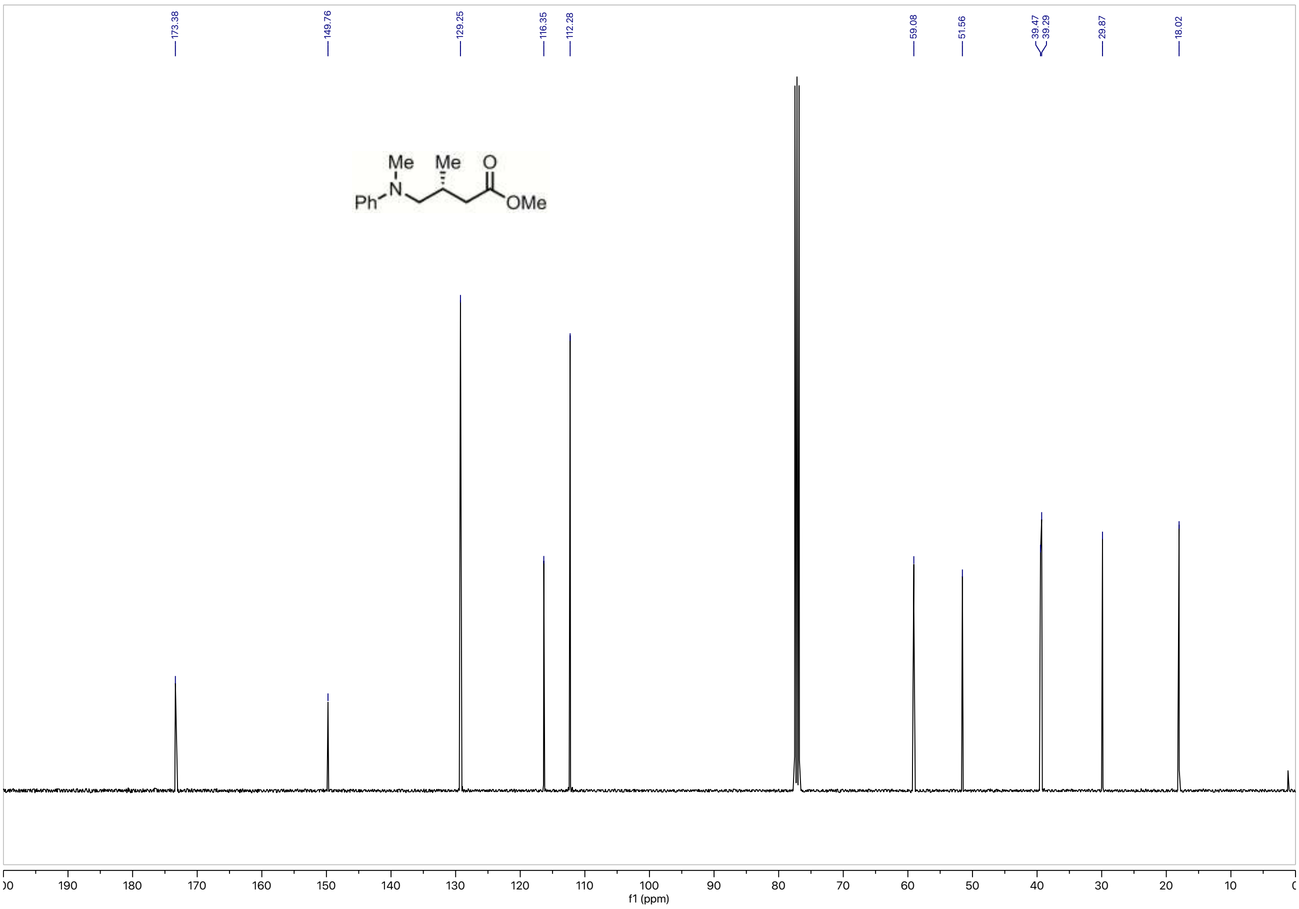

^{1}H NMR: **12** (MeOD)

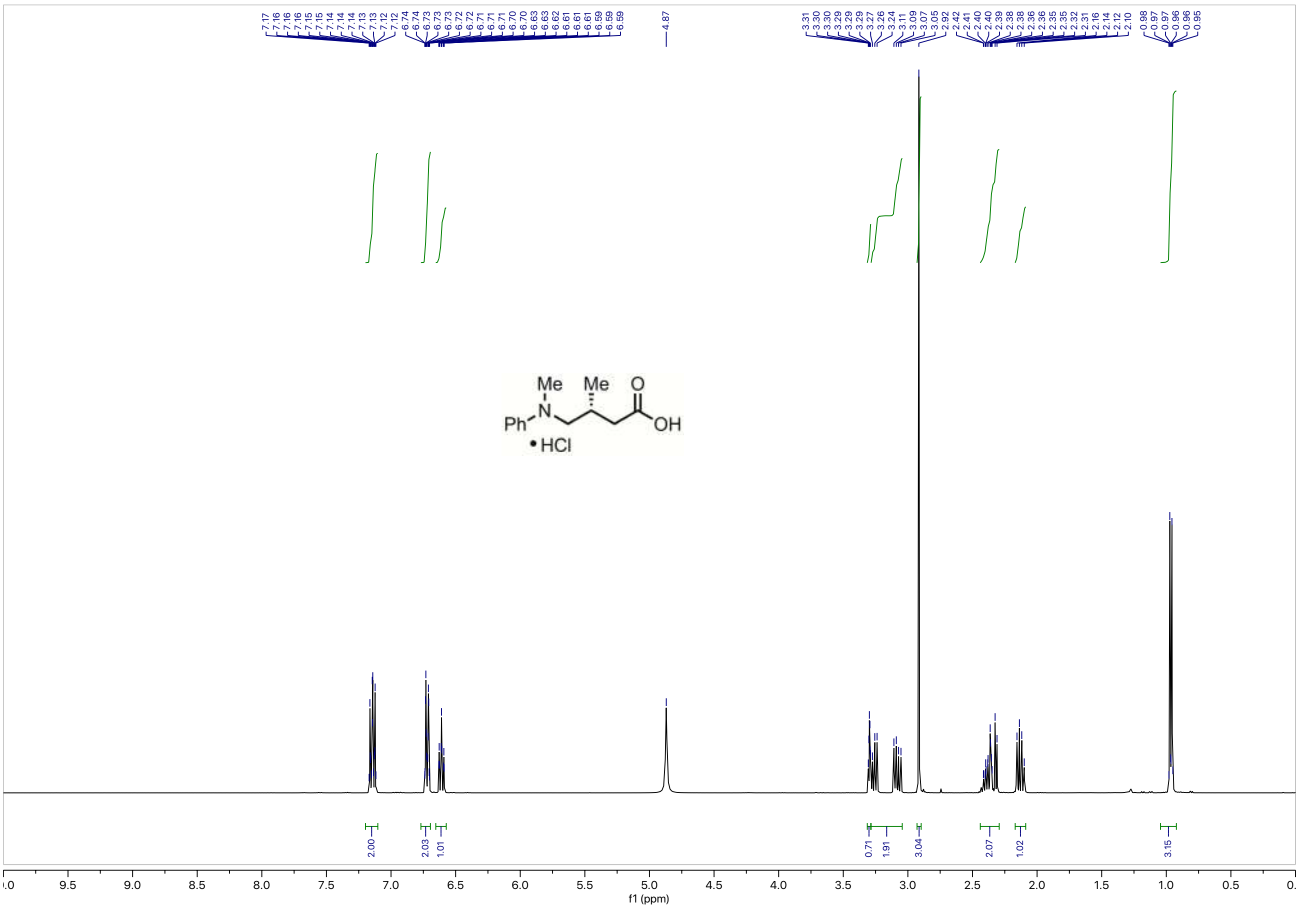

^{13}C NMR: **12** (MeOD)

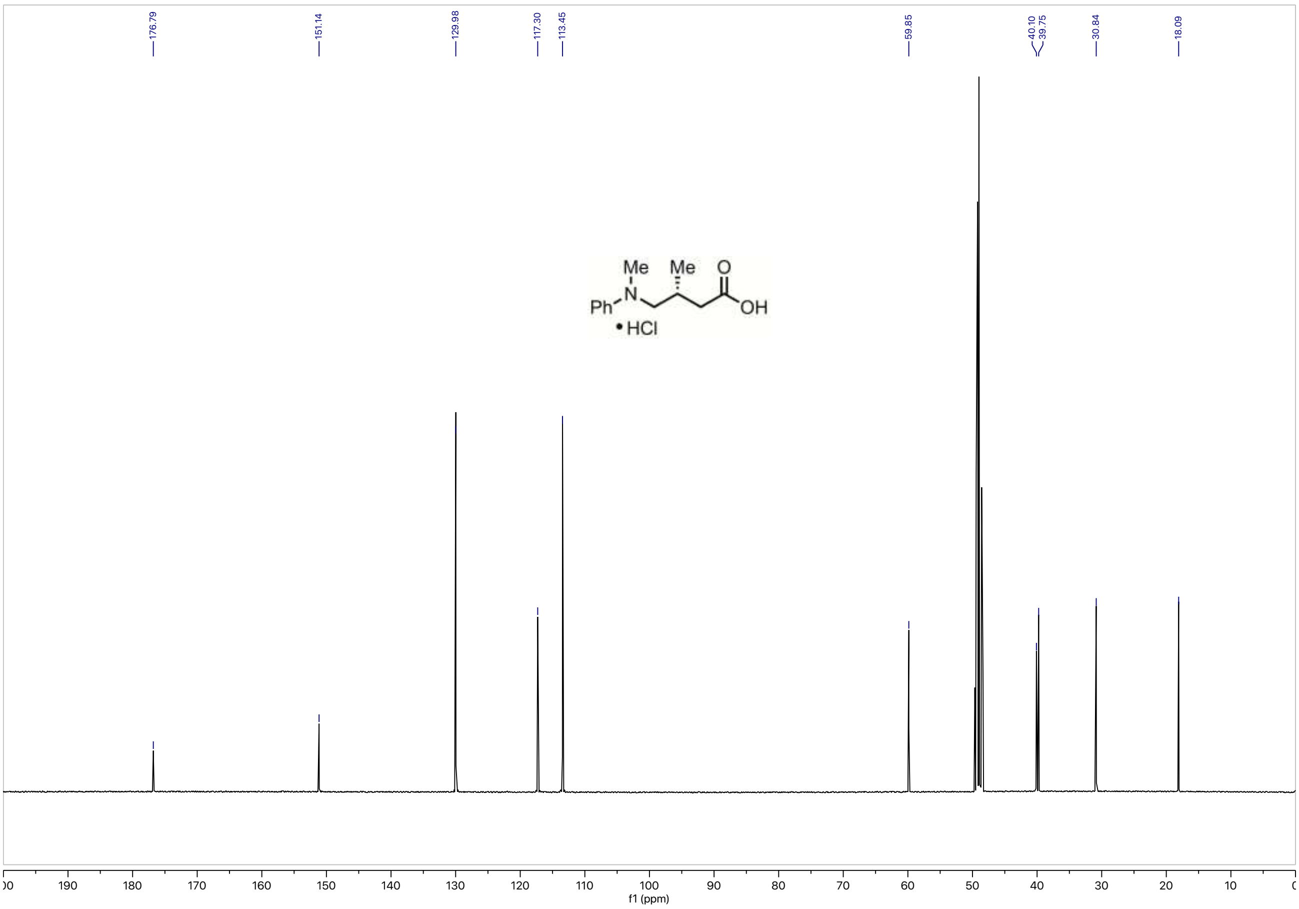

^{1}H NMR: **13**

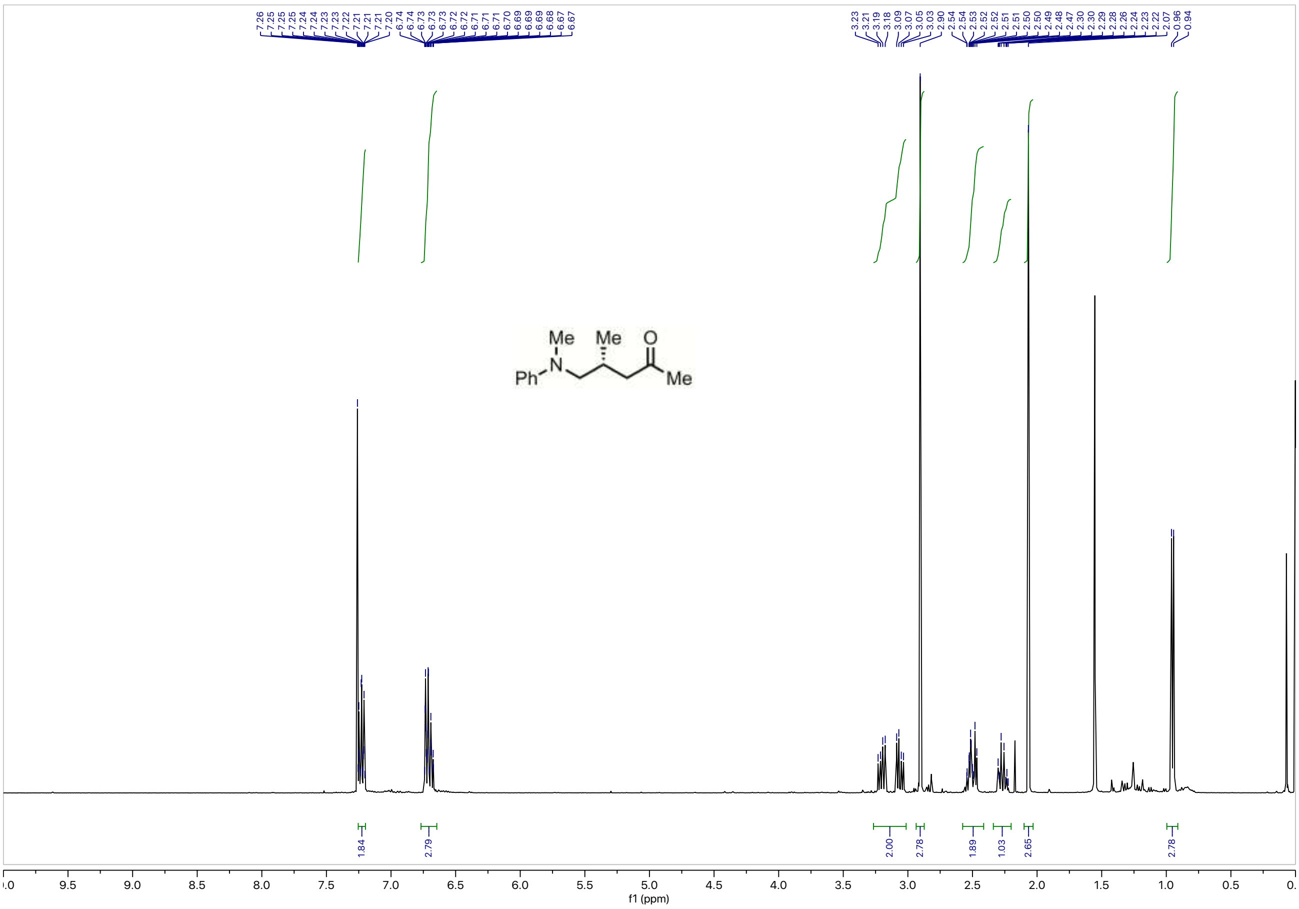

^{13}C NMR: **13**

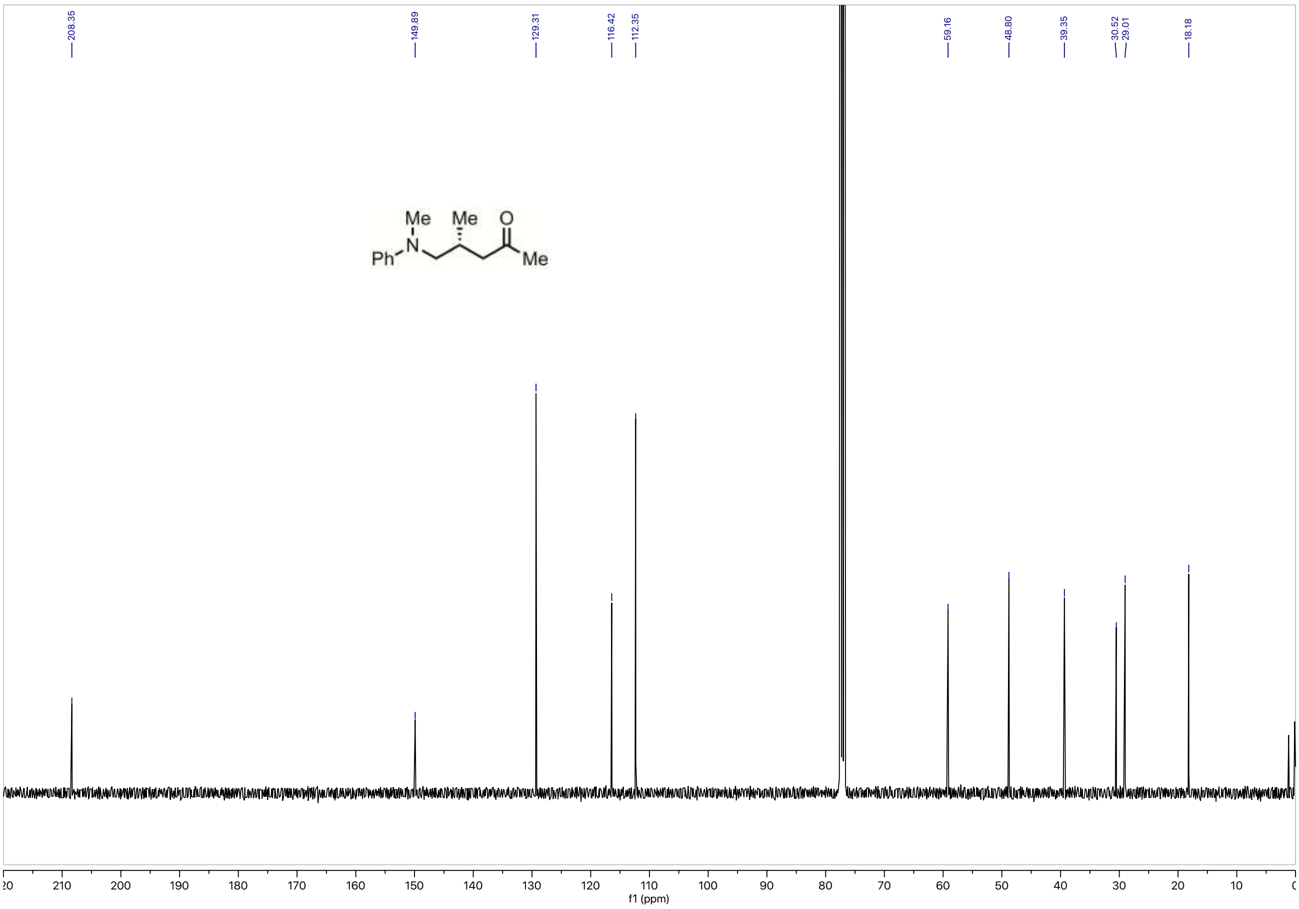

^{1}H NMR: **14**

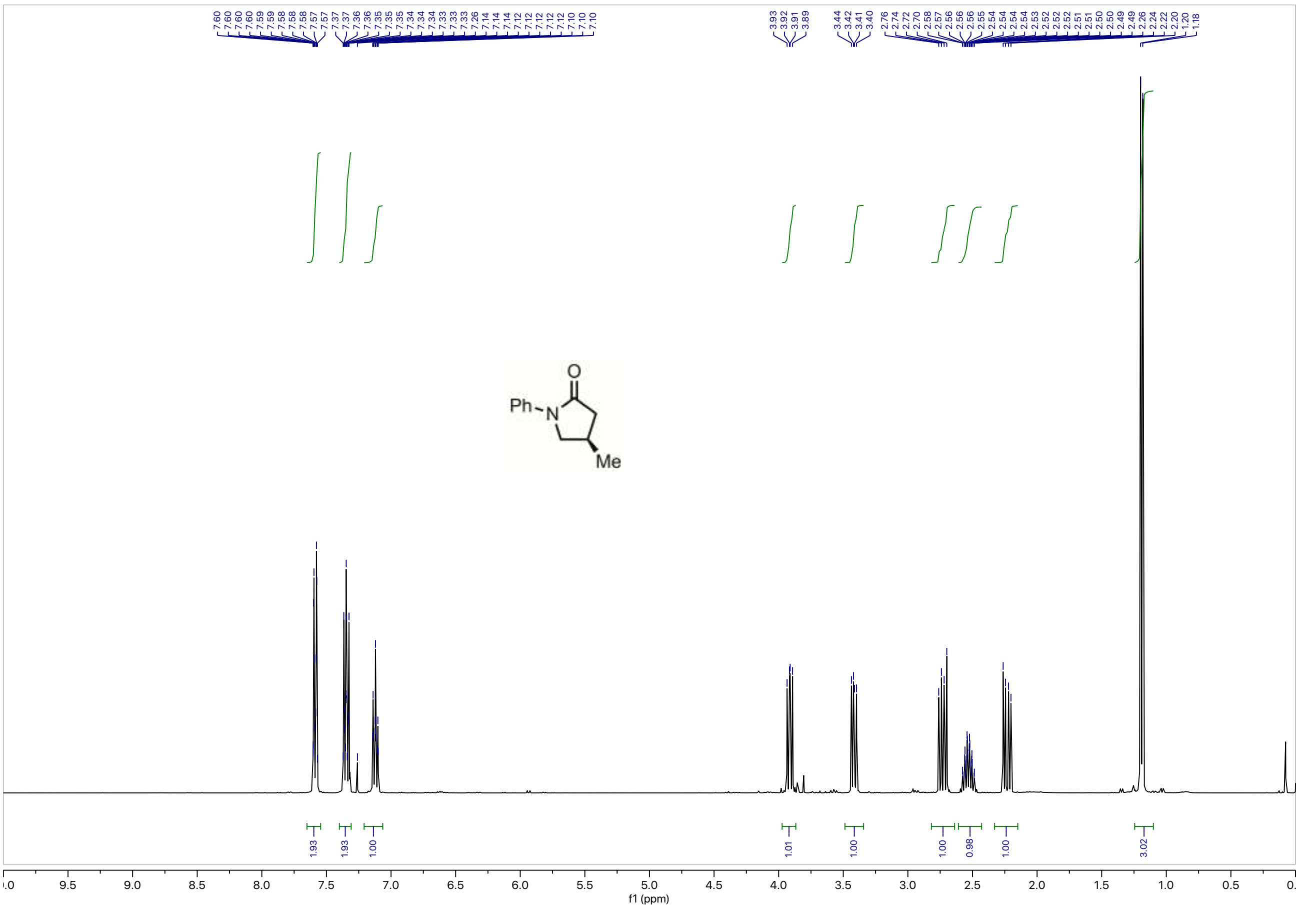

^{13}C NMR: **14**

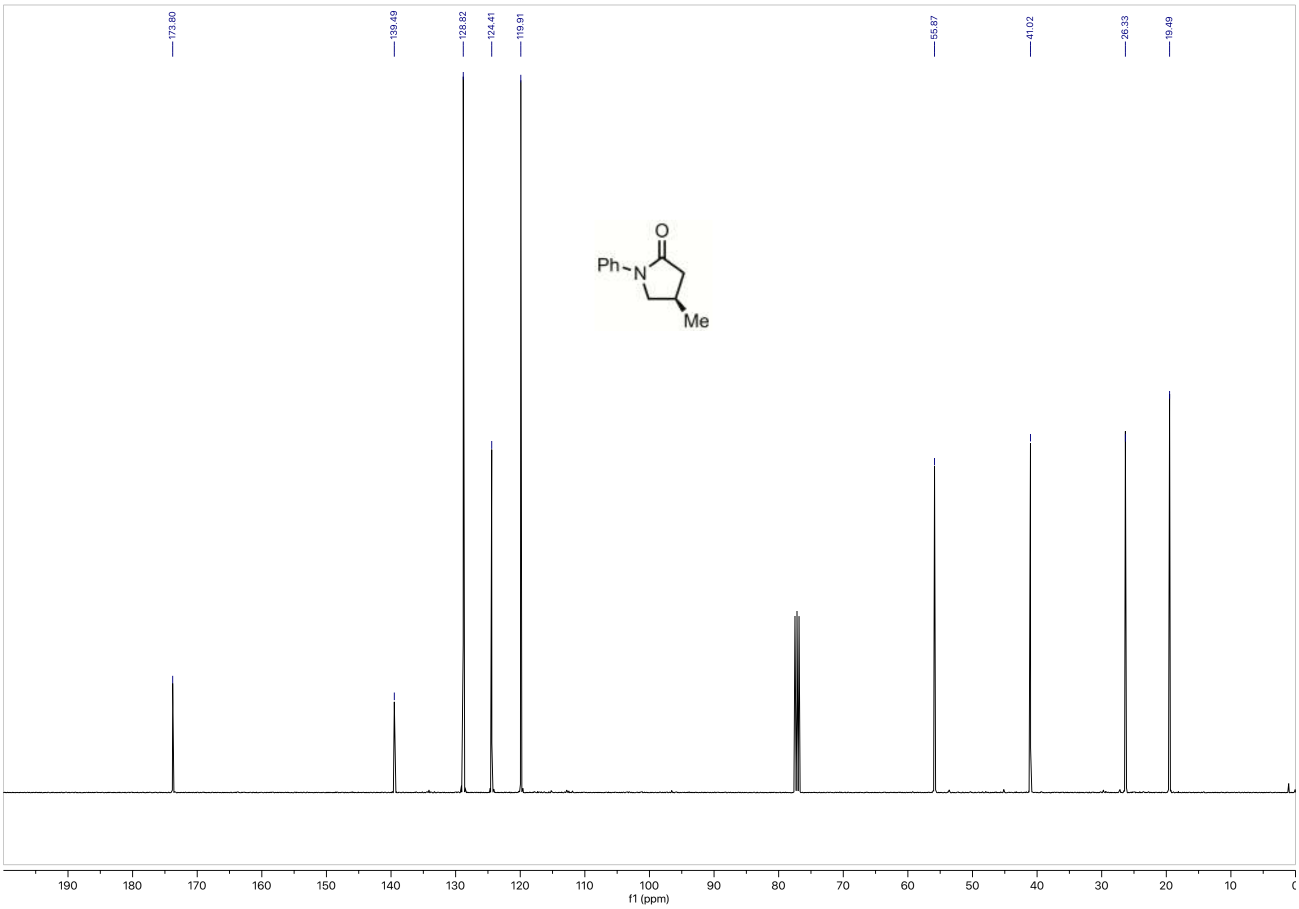

Supplement: SC-011-D0SC01890B-s001 [file SC-011-D0SC01890B-s001.pdf]
